# A Route to Potent, Selective, and Biased Salvinorin Chemical Space

**DOI:** 10.1021/acscentsci.3c00616

**Published:** 2023-07-12

**Authors:** Sarah J. Hill, Nathan Dao, Vuong Q. Dang, Edward L. Stahl, Laura M. Bohn, Ryan A. Shenvi

**Affiliations:** † Department of Chemistry, Scripps Research, La Jolla, California 92037, United States; ‡ Graduate School of Chemical and Biological Sciences, Scripps Research, La Jolla, California 92037, United States; § Department of Molecular Medicine, The Herbert Wertheim UF Scripps Institute for Biomedical Innovation & Technology, Jupiter, Florida 33458, United States

## Abstract

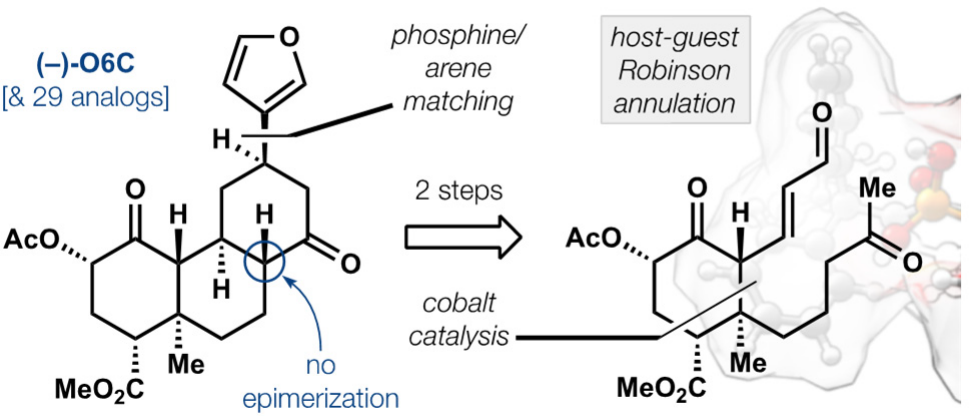

The salvinorins serve as templates for next generation analgesics, antipruritics, and dissociative hallucinogens via selective and potent agonism of the kappa-opioid receptor (KOR). In contrast to most opioids, the salvinorins lack basic amines and bind with high affinity and selectivity via complex polyoxygenated scaffolds that have frustrated deep-seated modification by synthesis. Here we describe a short asymmetric synthesis that relies on a sterically confined organocatalyst to dissociate acidity from reactivity and effect Robinson annulation of an unactivated nucleophile/unstable electrophile pair. Combined with a cobalt-catalyzed polarized diene-alkyne cycloaddition, the route allows divergent access to a focused library of salvinorins. We appraise the synthesis by its generation of multiple analogs that exceed the potency, selectivity, stability, and functional bias of salvinorin A itself.

## Abstract 2

A 10-step asymmetric and diversifiable route to stabilized salvinorin A analogs uncovers new reactivity and delivers superior pharmacological profiles.

## Introduction

**Figure 1:**
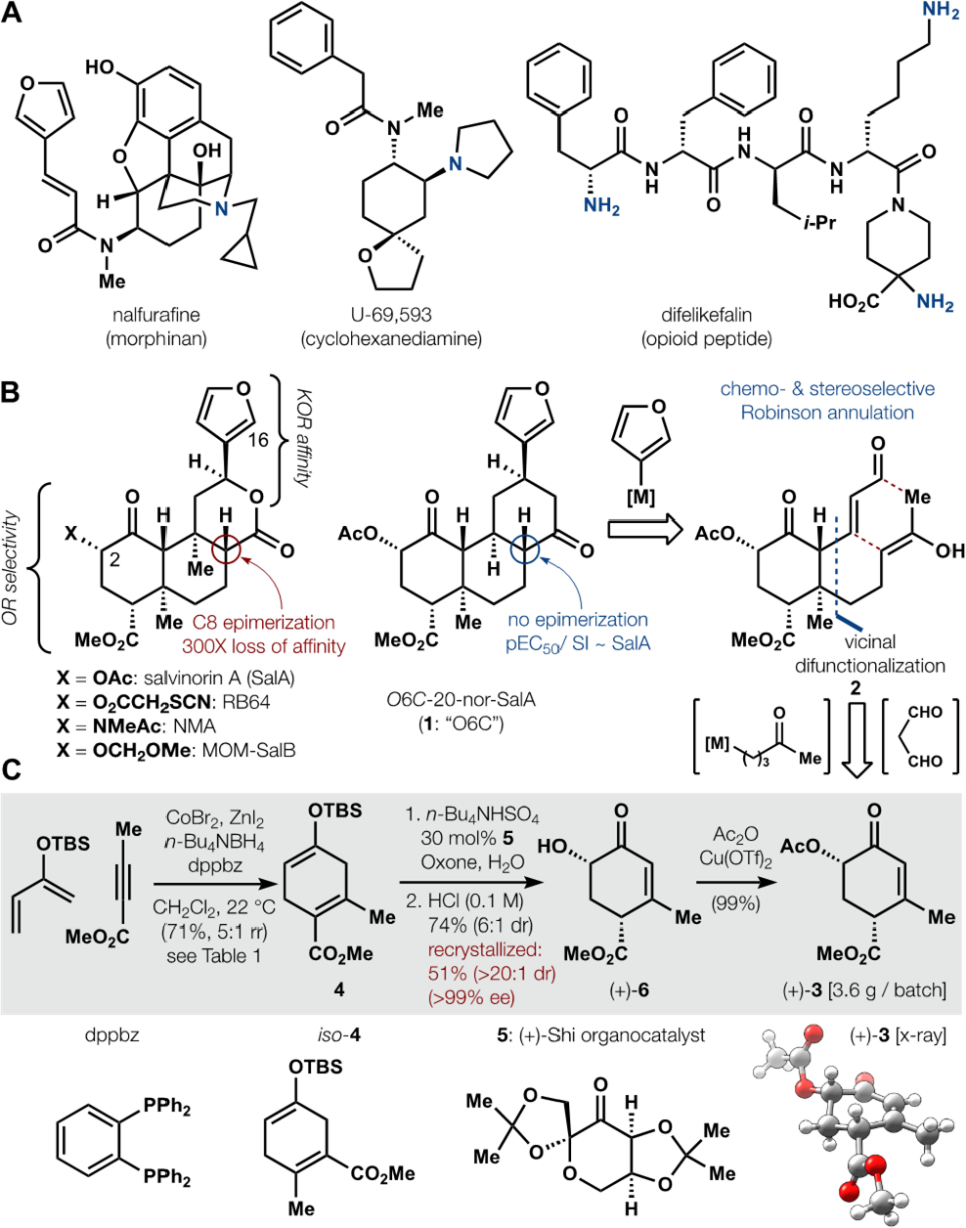
Salvinorin A, a KOR-selective hallucinogen, differs from common opioids. (A) KOR-selective opioid ligands that contain nitrogenous Brønsted bases. (B) Promising salvinorin A analogs, including the configurationally stable O6C and plan to effect Robinson annulation. (C) Synthesis entry that relies on control of regioselectivity in a cobalt-catalyzed cycloaddition.

Evolutionary selection pressures endow natural products with properties that are advantageous to drug development.^1^ Many structures, however, frustrate optimization campaigns due to their high complexity and low modularity. Salvinorin A, for example (Figure 1), is a potent and selective KOR agonist (EC_50_ = 0.9 nM, cAMP inhibition; selectivity index >5000 vs mu-opioid receptor, MOR).^2^ It exhibits a high lipophilic ligand efficiency (LLE = 8; ClogP = 1.6)^3^ and rapidly penetrates mammalian brains to cause psychoactive effects at low concentration (est. 10 μg total human brain content).^4^ Its complexity, however, has not allowed diversification and optimization by a total synthesis. Instead, the salvinorins have been subject to extensive semisynthetic campaigns, which limit diversity (see below).^5^ These studies have targeted next-generation analgesics and antipruritic agents that rely on the unique physicochemical properties of the salvinorins, which differ markedly from other KOR-selective agonists (Figure 1A), in no small part due to the absence of a basic amine. Since pain relief via agonism of the related MOR leads to dependency and addiction, significant effort has focused on selective targeting of KOR. This alternative opioid receptor has not been associated with addiction and therefore represents a critical research area for pharmaceutical development. More recent recognition that GPCR ligands can mediate “biased” or “functionally selective” signaling has stimulated a search for new KOR-targeting chemotypes with pliable pharmacology that preferentially activate G protein pathways over β-arrestin recruitment.^6,7^

Semisynthetic modifications of isolated SalA have produced analogs with extended blood half-life,^8,9^ increased oral bioavailability,^10^ and expanded receptor pharmacology.^11^ Structural diversity and multisite exploration, however, remain limited: only C2 and C16 analogs have met or exceeded the potency of SalA.^5^ For example, the equipotent C2 thiocyanatoacetate analog, RB64, exhibited G protein-biased signaling over β-arrestin via proposed covalent adduction at the site of binding.^12^ C16-bromo-salvinorin A, a noncovalent analog, showed functional bias for G protein signaling over β-arrestin2 recruitment and promise as an analgesic (bias factor 7.7); translational potential was attenuated, however, by lower efficacy than balanced analogs in pain-related behavioral models, in addition to sedative effects and low metabolic stability associated with the C2 acetate.^13^ The metabolic half-lives of salvinorins can be enhanced by the C2 *N*-methylacetamide (NMA) modification, which resisted esterase degradation and increased oral bioavailability, but lost 27× KOR/MOR selectivity.^10^ A series of ethers at C2 (e.g., MOM) maintained SalA potency and selectivity while improving metabolic stability, yet did not increase oral bioavailability or brain residence time.^9^ Whereas SalA showed tolerability in five healthy patient trials, no salvinorin analog has advanced to a disease-modifying clinical trial.^13^ De novo synthesis could expand the analog search space without the limitations of SalA reactivity and stability.

Prior syntheses^14−20^ and medicinal chemistry campaigns struggled with the configurational lability of C8, which underwent epimerization (*K*_eq_ = 2.5) to generate the low potency 8*-epi*-SalA under acidic, basic, and thermal conditions.^5^ Whereas total syntheses might provide more analogs for property optimization, they have ranged from 16–29 steps (1.3–0.3% yield)^14−22^ generated no analogs and retained, by definition, the C8 configurational lability of SalA itself.^22^ As a result, two syntheses targeted stabilized SalA scaffolds that would not epimerize. One elegant effort introduced multiple scaffold changes via a stereoselective transannular Michael cascade (see Scheme S8) but resulted in >100× potency loss and no measure of receptor selectivity.^23^ Another effort relied on computational modeling^24^ to modify the B and C rings and arrive at the analog O6C (**1**, EC_50_ = 3.3 nM),^25^ which resisted epimerization and approached the potency of SalA (ref (25) and see below). Nevertheless, this effort required 13 steps to synthesize racemic material (<1% yield) and was not diversified to analogs. Poor divergency resulted from reliance of C-ring closure on an electron-rich arene that was installed 5-steps away from bioactive chemical space, i.e., **1** (see Scheme S11). Greater divergence and brevity were designed via retrosynthetic conjugate addition and Robinson annulation, if the enol of **2** could engage in chemo- and stereoselective reaction with the pendant enal, an acidic vinylogous 1,3-dicarbonyl and unprecedented partner. Intermediate **2** could derive from 2-acetoxy-Hagemann’s ester (**3**) as a scalemic building block. Here we report an asymmetric synthesis of (−)-**1** plus 29 bioactive analogs by diversification of a common scaffold (9 steps, 6.7% overall yield). New structure activity relationships that deliver increased potency, selectivity, and C8 stability, in addition to G protein-biased agonism, demonstrate the practicality of the route.

## Results and Discussion

The synthesis commenced with a Diels–Alder reaction between two electronically matched partners. Neither high temperatures nor Lewis acids, however, promoted cycloaddition (entries 2 and 3 in Table 1) and attempts to access **4** via Birch reduction^26−28^ proved unsuccessful. Only Hilt’s cobalt-catalyzed cycloaddition^29^ delivered 1,4-cyclohexadienes (72%), albeit as a ∼1:1 ratio that favored regioisomer *iso*-**4**. Whereas many diphosphine ligands failed to promote reaction or regioselectivity, inexpensive dppbz and 4,4′-di-*tert*-butylbipyridine (dtbbpy) ligands favored isomer **4**; the former provided higher yields. Zinc metal as reductant led to variable results on scale due to slow reduction rates in CH_2_Cl_2_, but tetrabutylammonium borohydride caused an immediate color change^30^ and provided 71% yield with 5:1 rr over multiple large-scale runs (ca. 55 mmol; see Table S2 for optimization). The Co•dppbz complex enabled regioselective reaction of electronically matched, yet thermally unreactive, polarized diene-dienophile pairs; precedented conditions only used dienophiles with a pendant alkene ligand.^29,31^ Selectivity may be dictated by either steric interactions in the regiochemistry-determining step by analogy to phenyl acetylene cycloadditions^32^ or enhanced polarization of the butynoate•Co complex using the narrow bite-angle diphosphine (dppbz β_n_ = 83°) by analogy to metal hydricity trends.^33^

**Table 1:** Cobalt-Catalyzed Polarized Diene Cycloaddition^a^

Deviations	4	*iso*-4
none	59%	12%
140 °C	0%	0%
AlMe_3_ not CoL_n_	0%	0%
dppe not dppbz	33%	39%
dppp not dppbz	2%	1%
dppb not dppbz	0%	0%
20 mol % not 10 mol % dppbz	28%	5%
dtbbpy not dppbz	45%	<2%
Zn not *n*-Bu_4_NBH_4_	variable yields	

a Optimized conditions: 10 mol % dppbz, 10 mol % CoBr_2_, 50 mol % ZnI_2_, 0.3 equiv. *n*-Bu_4_NBH_4_, CH_2_Cl_2_ (0.5 M), 22 °C. dtbbpy = 4,4′-di-*tert*-butylbipyridine.

Enantioselective oxidation of **4** was explored using Jacobsen epoxidation and Sharpless dihydroxylation, but these conditions failed to engage the electron-rich alkene, as suggested by the absence of cyclohexenol silyl ethers in prior scope.^34,35^ Fortunately, Shi epoxidation^36^ (l-fructose-derived catalyst) effected Rubottom oxidation in 74% yield, 91:9 enantiomeric ratio, and a 5.7:1 *cis*:*trans* diasteromeric ratio resulting from selective deconjugation of the ester. Recrystallization of this intermediate increased the er to over 99:1 and delivered **6** in 51% yield as a single diastereomer. This Hagemann’s ester analog, **6**, proved sensitive to epimerization or aromatization under basic conditions, but could be converted quantitatively to the C2 acetate with catalytic Cu(OTf)_2_ in neat acetic anhydride on multigram scale.^37^ Prior analysis had suggested **3** as an entry to the enantioselective synthesis of the salvinorins.^38^ Its synthesis, however, required 9 steps, relied on a stoichiometric ephedrine chiral auxiliary to reach scalemic intermediates, and eventually yielded *rac*-**3** only. In comparison, the entry in Figure 1 requires four steps and a chiral catalyst to produce near-enantiopure (+)-**3**.

**Figure 2:**
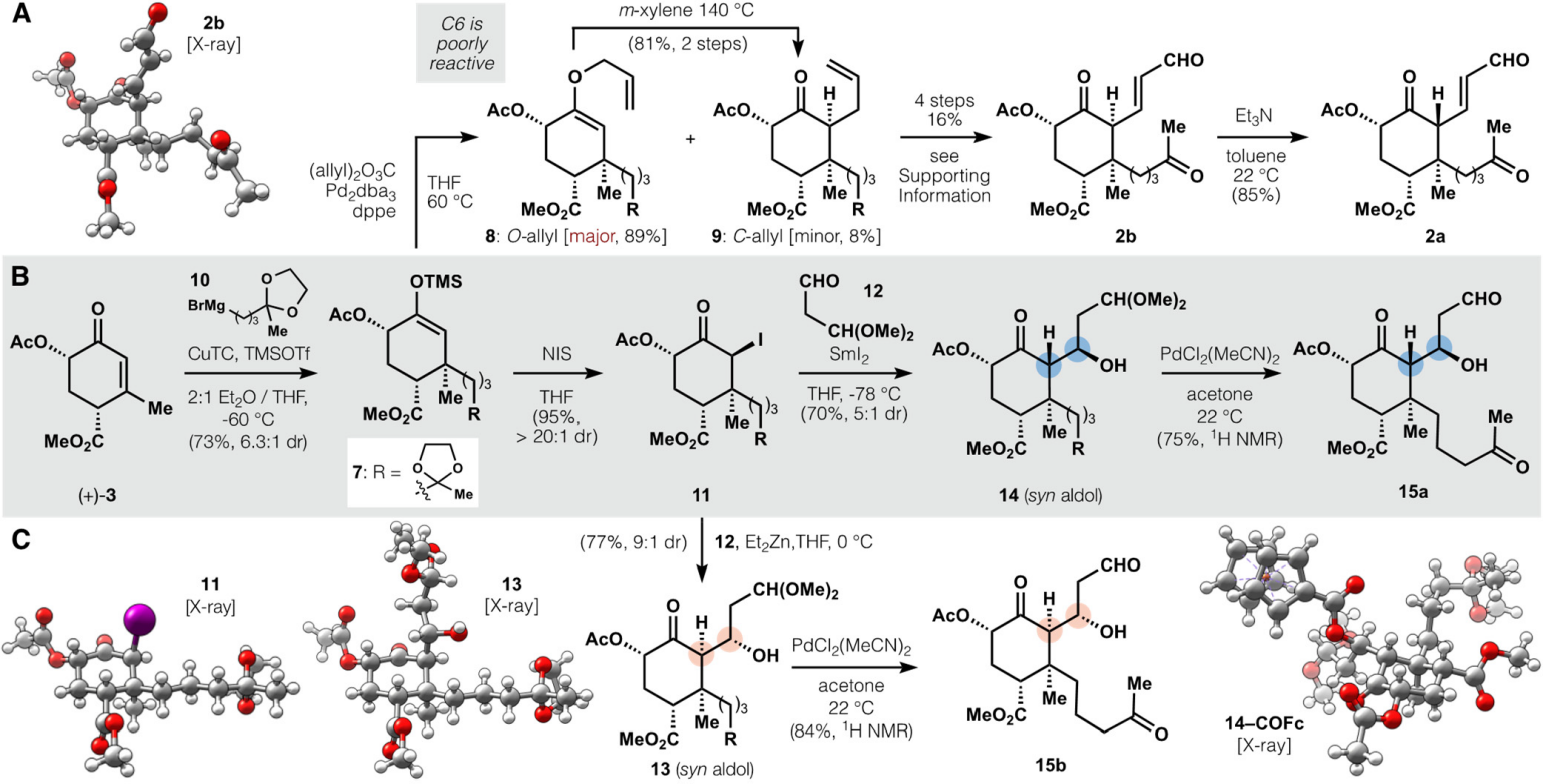
Vicinal difunctionalization of **3**. (A) The low reactivity of C6 prevents direct C–C bond formation. (B) Sm-Reformatsky aldol allows efficient side chain appendage with equatorial synaldol selectivity. (C) Zn-Reformatsky aldol yields axial syn-aldol selectivity.

Previous work had established a procedure for the vicinal difunctionalization of Hagemann’s ester,^24^ yet these conditions and numerous iterations failed in the presence of the C2–O substituent, which rendered the ketone more sensitive to strong base and deactivated the already hindered, β-trisubstituted enolate likely via π_CC_–σ*_CO_ conjugation. After extensive optimization, we found that organocopper addition to the base-sensitive, hindered enone occurred most efficiently in the presence of trimethylsilyltrifluoromethanesulfonate (TMSOTf) as a powerful activator to accelerate oxidative addition of organocopper reagents (Figure 2).^39^ Further screening identified diethyl ether as a cosolvent crucial for diastereoselectivity; in its absence, **10** added to the opposite face of the enone competitively. Diethyl ether likely affects the organocopper aggregation state, increases C2 acetate coordination of copper or both. Low-temperature quench with aqueous ammonium chloride prevented the side-reactions of excess **10** that occurred upon warming and spared the C6 enol silane, which showed stability even to chromatography on acidic silica gel, a result of its low nucleophilicity. Direct evidence of the low reactivity of the C6 position emerged during attempted *C*-allylation: treatment of enol silane **7** with diallyl carbonate, Pd_2_(dba)_3_•CHCl_3_ and dppe at 60 °C yielded the *O*-allyl ether **8** instead of the *C*-allyl ketone **9** (11.4:1 **8**:**9**), whereas similarly hindered cyclohexenyl allyl carbonates have led to *C*-allyation exclusively.^40^ This reaction sequence vividly illustrated the low reactivity of C6. Claisen rearrangement (*O*- to *C*-allyl, **8** to **9**) and multiple functional group interconversions (FGIs) could adapt the unfunctionalized allyl group to suit the synthesis (**9** to **2b**, **2b** to **2a**), but this sequence consumed time, labor, and material.

**Figure 3:**
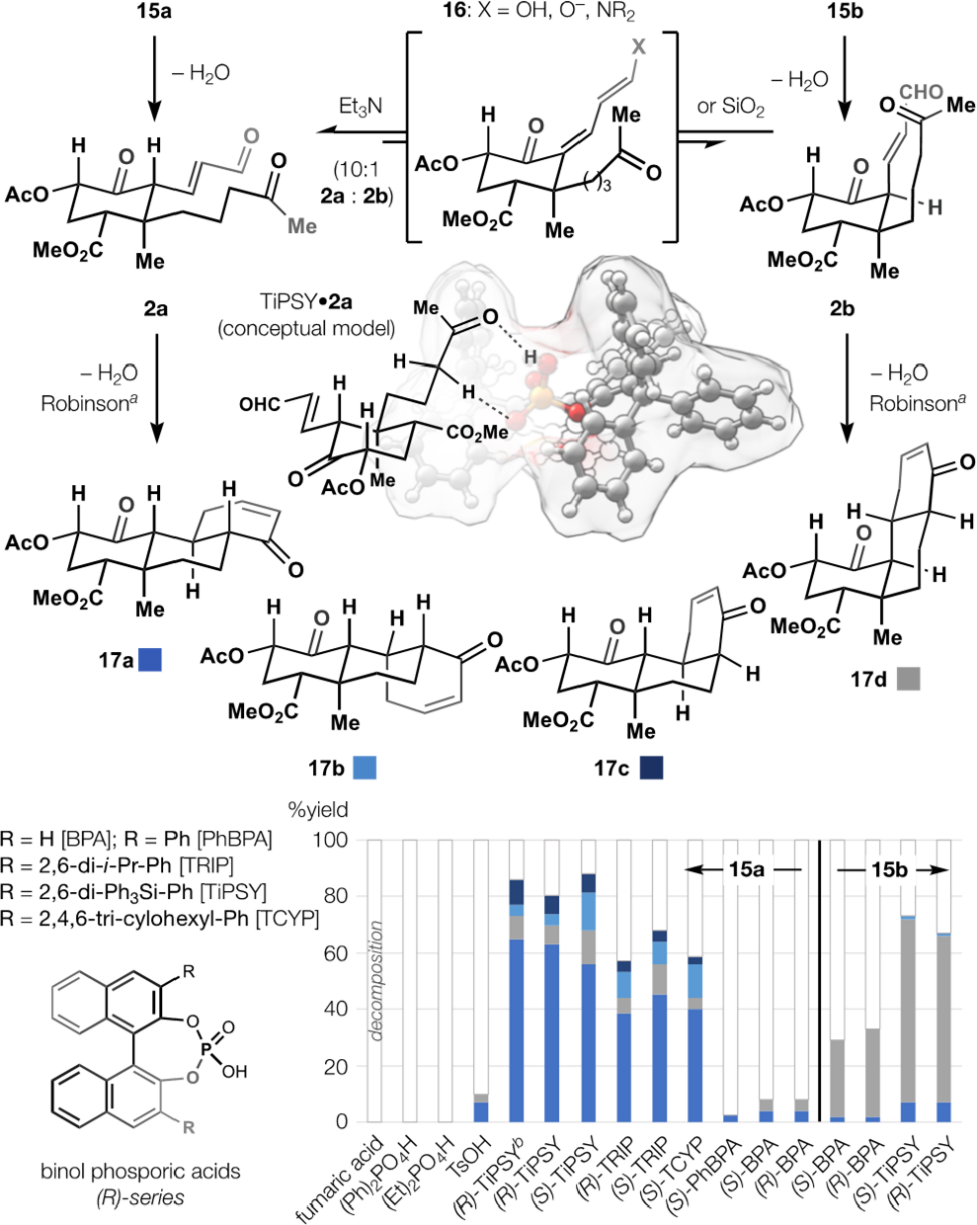
Robinson annulation by selective enolization. Chiral binolphosphoric acids with large *o*,*o*′-substituents enolize the weakly acidic, unhindered ketone but spare the highly acidic, hindered keto–enal. Empty bars indicate decomposition, not residual starting materials. ^*a*^30 mol % acid catalyst, 0.1 M **15a** or **15b**, PhCl, 85 °C; ^*b*^toluene not PhCl.

Instead, we considered Reformatsky variants recently shown to couple ketone–aldehyde pairs that were unreactive under basic or Lewis acidic conditions.^41^ Despite the low reactivity of **7**, *N*-iodosuccinimide effected high-yielding and diastereoselective α-halogenation on gram-scale (95%, >20:1 dr, 1 g per batch; see **11** X-ray); iodide **11** proved stable to thiosulfate workup, chromatography, and crystallization (Et_2_O, −20 °C). Nevertheless, **11** underwent efficient Reformatsky reaction with malondialdehyde dimethylacetal (**12**) under the action of diethylzinc (Et_2_Zn) to yield the *syn*-aldol diastereomer in an axial orientation (77%, 9:1 dr; see **13** X-ray). Whereas *E*-enolates tend to yield *syn*-aldols via open (Noyori) transition states, the absence of strong Lewis acid here suggests a closed (Zimmerman-Traxler) transition state where the aldehyde alkyl chain is forced into an axial approach (enolate *si*-face) by the enolate β-substituents (see Figure S7).^42^ The high stereoselectivity, however, proved problematic because axial diastereomer **13** could not be advanced in the synthesis (see Figure 3 below). Fortunately, SmI_2_ could replace Et_2_Zn and retain *syn*-selectivity but reverse the aldehyde approach (enolate *re*-face) to yield diastereomer **14** (70%, 5:1 dr; see **14-COFc** X-ray). The origin of this reversal may involve samarium chelation^43^ between the enolate C1 oxygen and C2 acetate to enforce aldehyde approach adjacent to the axial β-methyl. Double hydrolysis of the acetals required unusual conditions due to the sensitivity of aldehydes **15a** and **15b** to silica gel, aqueous workup, and residual acid: PdCl_2_(MeCN)_2_ in acetone^44^ delivered keto-aldehydes after filtration through activated carbon, followed by evaporation. Thus, enone (+)-**3** could be stereoselectively functionalized in 4 steps with side chains in the correct oxidation state for a tandem cyclization of both B and C rings.

Attempts to effect Robinson annulation of **15a** or **2a** met initially with failure due to the competitive reaction of their multiple carbonyls in preference to the methyl ketone. Standard reagents—amines, acids, and bases—first engaged α,β-unsaturated aldehydes **2a** and **2b** (Figure 3), produced from **15a** or **15b** by loss of water (identified *in situ* by comparison to the products of Figure 2A). Increased temperatures or reagent concentrations did not allow cyclization into structures such as **16** and instead caused extensive decomposition using either **15a** or **2a** generated independently. The low rate of cyclization and the tendency toward decomposition presented a dilemma: either the synthesis would require multiple steps to replace the linear methyl ketone with a superior nucleophile or the linear methyl ketone would require selective enolization. Unfortunately, the enal side chains could enolize with great ease, as demonstrated by the epimerization of **2b** to **2a** by Et_3_N or SiO_2_ at 22 °C (17:1, **2b**:**2a**). We turned to commercially available chiral phosphoric acids, whose large substituents can dissociate reactivity from acidity^45,46^ via noncovalent interactions^47^ in flexible binding pockets.^48^ Selective ketone binding and enolization, comparable to TRIP-AcOH host–guest binding,^47^ might avoid reaction of bulkier Lewis basic motifs prone to decomposition, like the enolizable C6 ketone or ionizable C2 acetate. Phosphoric acids with no or minimal ortho-substitution—BPA (p*K*_a(DMSO)_ = 3.37^49^) and *o,o′*-diphenyl-BPA (p*K*_a(DMSO)_ = 3.86^49^)—led to only trace product. However, 2,4,6-tricylohexylphenyl- (TCYP), 2,4,6-tri-*iso*-propylphenyl- (TRIP, p*K*_a(DMSO)_ = 4.22^49^) and triphenylsilyl- ((*R*)- or (*S*)-TiPSY, p*K*_a(DMSO)_ = est. 4.42^46^) substituents provided increasing amounts of Robinson annulation, ultimately delivering an 85% yield (18:1:2:2 dr) favoring **17a** over 3 other diastereomers (**17b**–**d**). All phosphoric acids were solubilized completely under the reaction conditions and led to full consumption of starting materials. Small acids with lower, similar and higher p*K*_a(DMSO)_ values (TsOH, 0.9;^50^ Ph_2_PO_4_H, 3.88;^49^ fumaric acid, 9.0^51^) led to decomposition. To distinguish between selective methyl ketone enolization versus capture of the enal–enol equilibrium (i.e., **2a** ⇋ **16** ⇋ **2b**), we subjected **15b** to identical conditions—(*R*)- or (*S*)-TiPSY or BPA, 30 mol %, PhCl, 85 °C. Isomer **2b** proved less sensitive to decomposition than **2a** (see BPA entries) but cyclized to **17d** only, despite the ability of **2b** to enolize with Et_3_N and SiO_2_. That the phosphoric acid enantiomeric series had only a small effect on both reaction efficiency and stereoselectivity suggested dissociation from the enol prior to cyclization.^52^ Selective enolization of ketones is precedented in special cases where A(1,3)-strain reduces the competitive, kinetic acidity of low p*K*_a_ 1,3-dicarbonyls;^53^ that situation does not appear relevant here, and the selectivity imparted by bulky phosphoric acids, as monomers or aggregates,^54^ appears unprecedented.

**Figure 4:**
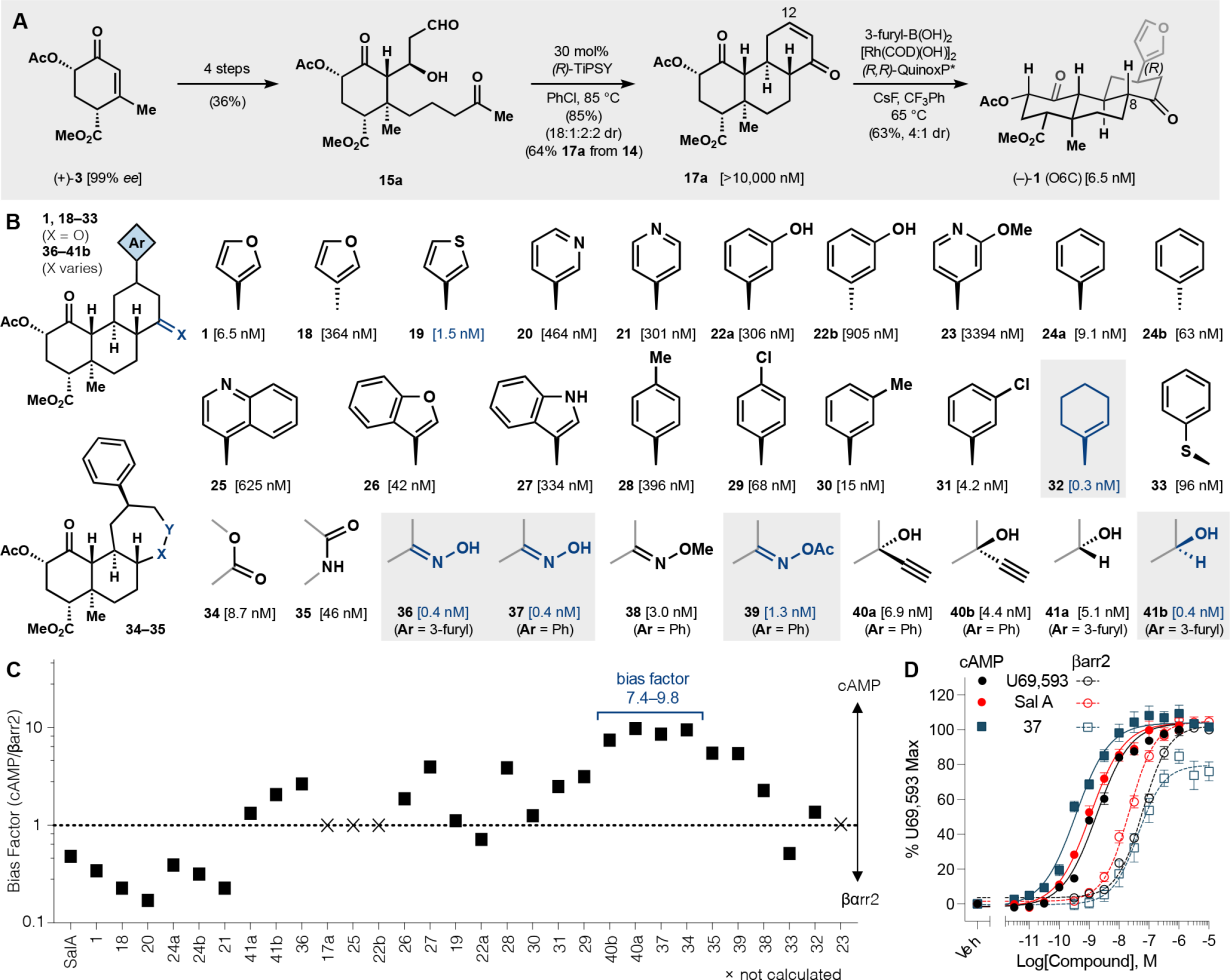
Pharmacology measures synthesis design and practicality. The synthesis could be judged by its ability to generate multiple analogs that exceeded the potency, stability, selectivity, and functional bias of SalA. (A) Overview of the synthesis and stereoselective 1,4-addition of 3-furyl. (B) Focused library to probe SAR where bracketed concentrations denote mean EC_50_ of inhibition of forskolin-stimulated cAMP accumulation via KOR; *n* ≥ 3 (Cisbio HTRF). For tables of pharmacology data and plotted curves, see Figures S1 and S2 and Table S1. (C) Bias factors [10^^(ΔΔlog (τ /KA)(cAMP-βarr2)^] for SalA and analogs. (D) Representative CRCs of select analogue **37** vs positive controls U69,593 and SalA in cAMP and β-arrestin2 (DiscoveRx PathHunter) signaling assays; data are normalized to the baseline and to the maximum response produced by 10 μM U69,593, and presented as the mean ± SEM.

To evaluate the practicality of the synthesis, we probed the ease with which we could assemble a focused library of bioactive congeners. Robinson annulation product **17a** could be advanced to O6C (**1**, Figure 4A) by diastereoselective Hayashi conjugate addition using [Rh(COD)(OH)]_2_ with (*R*,*R*)-QuinoxP* to disfavor the C12 epimer, which predominated with other phosphines or organocopper nucleophiles via a proposed Fürst-Plattner model of stereoselectivity (see Figure S11). Selective access to the C12(*R*) configuration using (*R*,*R*)-QuinoxP* was restricted to electron-rich heterocyclic boronic acids, whereas electron-neutral phenyls required (*R*)-BINAP and electron-deficient aryls relied on (*R*,*R*)-BenzP* in *t*-BuOH for high diastereoselectivity (Table S8); these optimizations benefited from ready access to enone **17a**. The final conjugate addition completed a 10-step synthesis of O6C and accessed 29 other unprecedented analogs, including heteroaromatics and substituted phenyls. No conditions produced C8 epimers, by virtue of the designed scaffold stability of O6C (**1**) itself.^24,25^

Seminal work by Prisanzano established the importance of the C12 furan and its potential replacement with a series of aryl ketones via oxidation and Liebeskind-Srogl coupling.^55^ Nevertheless, this C12 series led only to losses in potency, in contrast to widely explored C2 analogs,^12,56^ and left many questions about important binding site contacts. We assayed^7^ diverse carbocycle substitutions to determine whether hydrogen bonding, cation−π interactions, or lipophilic contacts played important roles (Figure 4B). Prior binding models suggested a hydrogen bond in this region as necessary for high potency;^57^ a recent furan-to-phenyl replacement retained affinity but lost potency.^24^ To probe in-plane hydrogen bonding, we replaced the 3-furyl with strong acceptors^58^ like 3- and 4-pyridyl (**20**-**21**), but these led to potency losses (Figure 4C). Instead, 3-thiophenyl (**19**) and phenyl (**25a**) led to increased potency, which might have suggested important cation−π or π–π interactions^59^ perpendicular to the plane of the electron-rich rings. Investigation of this possibility was challenged by the sensitivity of potency to steric substitution (**23**, **25**–**27**), so methyl-/chloro- isosteres (**28**–**31**) were used to make small, polar perturbations. Like the electron-rich furan/thiophene rings, the electron-deficient 3-chlorophenyl (**31**) exhibited high potency; this apparent contradiction suggested that lipophilic interactions—not H-bonds or π-interactions—likely played a dominant role. Indeed, replacement of the 3-furyl with a 1-hexenyl substituent (**32**) dramatically increased potency 20-fold (EC_50_ = 0.3 nM) and surpassed that of SalA itself. Thiophenol 1,4-addition led to a thioether (**33**, EC_50_ = 96 nM) that indicated a facile entry to diversity via commercially available thiols.

The C11 ketone showed a greater tolerance for variation. Ring-expanded lactone and lactam analogs (**34**, **35**) retained good potency, as did oximes (**36**–**38**), easily accessible via condensation. Tertiary and secondary alcohols also proved potent and selective, especially **41b** (EC_50_ = 0.4 nM), which also exceeded the SalA potency. The alkynylated analogs may prove useful for the recently reported CATCH protocol,^60^ which requires active alkyne analogs for visualization of cellular and subcellular binding sites. Both alcohols and oximes offer prodrug functionalization sites to tune pharmacokinetics without alteration A-ring motifs (C2 acetate, ethers, or *N*-methyl-acetamide) that govern affinity, selectivity, and metabolism. SalA is highly selective for KOR over MOR and the analogs preserve, or in some cases improve upon, this selectivity profile, apparently a general feature of the O6C scaffold (see Figure S2 for MOR activity comparisons). Remarkably, multiple analogs display G protein signaling biased agonism, especially **34**, **37**, and **40a**,**b** (bias factor 7.4–9.8, comparable to 16-Br-SalA), measured as potency of G_ai_ protein-mediated inhibition of forskolin-stimulated cAMP accumulation versus β-arrestin2 recruitment, both normalized to U69,593. Notably, compound **37** (cAMP pEC_50_ = 9.38 vs βarr2 pEC_50_ = 7.37) relied on both the C12 phenyl and C11 oxime to expand its G protein bias; high selectivity for KOR over MOR was also preserved (Figure S2). The relative scarcity of G protein biased salvinorins in the literature suggests that this highly KOR-selective set represents a privileged area of chemical space to explore.

## Conclusion

The salvinorins represent unique chemotypes for development of KOR-selective analgesics and antipruritics, most notably for their nonbasic, high F_sp_3 scaffolds. The recreational use of *S. divinorum*, however, has led to restrictions in 32 US states and 20 countries, limiting access to plant-sourced materials for broad development.^61^ The synthesis reported here quickly enters the chemical space of salvinorin A and accesses new analogs at C11 and C12 that exceed the stability, potency, selectivity, and G protein bias of the natural product itself. Whereas an early salvinorin analog, herkinorin, exhibited the first example of MOR-selective G protein bias,^62^ corresponding KOR-selective biased agonists have proven harder to develop; the prevalence of functional bias in Figure 4C bodes well for further discovery. Key to this discovery platform were (1) a cobalt-catalyzed cycloaddition to a scalemic Hagemann’s ester derivative, (2) a stereoselective Reformatsky aldol reaction to overcome low enolate reactivity, and (3) the enolization of an unactivated ketone in the presence of an unstable electrophile to effect a stereoselective Robinson annulation. Diverse salvinorins can now be accessed by a concise total synthesis, placing a rational medicinal chemistry campaign within reach.

## Supporting Information Available

All data is made available in the main text or the Supporting Information, including experimental procedures, copies of NMR spectra, X-ray structure reports, full outlines of prior syntheses and all pharmacological protocols. Structural parameters for X-ray crystal structures are available from the Cambridge Crystallographic Data Centre (**3**: CCDC2202073; **6**: CCDC2195935; **11**: CCDC2211512; **S5**: CCDC2241886; **13**: CCDC2245372; **17b**: CCDC2250953; **17c**: CCDC2217612; **1**: CCDC2249326; **S6**: CCDC2127086; **2b**: CCDC2166577, **24a**: CCDC2263493) (PDF)
