# Supplementary material for: A Route to Potent, Selective, and Biased Salvinorin Chemical Space
Source: ACS Cent Sci. 2023 Jul 12;9(8):1567–74. doi: 10.1021/acscentsci.3c00616 (PMC10450872; doi:10.1021/acscentsci.3c00616)
Supplement: Supplementary file 1 — oc3c00616_si_001.pdf [file oc3c00616_si_001.pdf]

Supplementary Information

A route to potent, selective and biased salvinorin chemical space

Sarah J. Hill[1, 2, †], Nathan Dao[1, 2, †], Vuong Q. Dang,[3] Edward L. Stahl,[3], Laura M. Bohn,[3]* Ryan A. Shenvi[1]*
[1]Department of Chemistry, Scripps Research, 10550 North Torrey Pines Road, La Jolla, California 92037, United States [2]Graduate School of Chemical and Biological Sciences, Scripps Research, La Jolla, California 92037, United States, [3]The Herbert Wertheim UF Scripps Institute for Biomedical Innovation & Technology, Jupiter, Florida 33458, United States, 130 Scripps Way, Jupiter, FL 33458, United States
† These authors contributed equally to this work.
*Email: rshenvi@scripps.edu, laura.bohn@ufl.edu

Contents

Materials and Methods (Biology) S3
Figure S1. Concentration response curves in the cAMP and βarrestin2 signaling assays S4
Figure S2. Selectivity against hMOR........ S5
Table S1. Pharmacological parameters S6
Materials and Methods (Chemistry) S8
Scheme S1. Evans's salvinorin A synthesis[14]........ S9
Scheme S2. Hagiwara's 1st generation salvinorin A synthesis[15] S11
Scheme S3. Hagiwara's 2nd generation salvinorin A formal synthesis[16] S12
Scheme S4. Forsyth's salvinorin A synthesis[17] S13
Scheme S5. Metz's 1st generation salvinorin A synthesis[18]........ S14
Scheme S6. Maier's salvinorin A formal synthesis[19] S15
Scheme S7. Metz's 2nd generation salvinorin A formal synthesis[20] S17
Scheme S8. Prisinzano's stabilized salvinorin A scaffold[23] S18
Scheme S9. Rook's approach[38]........ S19
Scheme S10. Perlmutter's approach[21] S19
Scheme S11. Synthesis of *rac*-20-nor-SalA[24] and *rac*-O6C-20-nor-SalA[25] S20
Scheme S12. This work: enantioselective synthesis of (−)-O6C-20-nor-SalA S21
Diene **S1**........ S22
Alkyne **S2** S23
Bromo ketal **S3** S24
Aldehyde **12**........ S25
ent-Shi catalyst **5**........ S26
Cycloadduct **4 and iso 4** S27
Figure S3. TLC of Diels-Alder reaction S28

Hydroxy Hagemann's ester, **6** S29
Figure S4. Chiral SFC trace for (+)-**6** S31
Figure S5. Chiral SFC trace for *rac*-**6** S32
Acetate (+)-**3** S33
Grignard reagent **10** S34
Silyl enol ether **7** S35
Figure S6. Crude TLC of conjugate addition S37
Iodide **11** S38
SmI_2 Reformatsky adduct **14** S39
Ferrocene carboxylate **S5** S40
Et_2Zn Reformatsky adduct **13** S41
Figure S7. Discussion of Reformatsky stereoselectivity S42
Enone **17a, b, c, d** S43
Figure S8. TLC analysis of deprotection and Robinson annulation protocol S43
Deprotected aldehydes **15a** and **15b** S44
Figure S9. Enone stereochemical assignment S47
Figure S10. Representative crude spectra of Robinson annulation. S48
Scheme S13. Prior allylation approach S49
O-allyl **8** and *C*-allyl **9** S50
Allyl **S6** S52
Aldehyde **S7** S53
Enal **2a** S55
(−)-O6C-20nor-salvinorin A (**1**) S56
Analog procedures **18-42b** S57
Figure S11. Discussion of arylation stereoselectivity S59
Table S2. Optimization of cycloadditions and Birch reductions to access **4** S86
Table S3. Optimization of protection/acetylation of (+)-**6** S87
Table S4. Optimization of conjugate addition to access silyl enol ether **7** S88
Table S5. Optimization of Reformatsky reaction S89
Table S6. Optimization of deketalization of **14** S90
Table S7. Attempted optimization of amine/acid catalyzed Robinson annulation S91
Table S8. Optimization of arylation of **17a** S92
References S95
NMR Characterization S100
X-ray crystallographic data S172

Materials and Methods (Biology)

Our drugs: U-69593 (Sigma-Aldrich, U103), salvinorin A (Sigma-Aldrich, S8071), PDE4 inhibitor (Sigma-Aldrich, B8279), forskolin (Toronto Research Chemicals, F701800), DAMGO (Tocris, 1171), Buprenorphine (the NIDA Drug Supply Program).

Forskolin-stimulated cAMP accumulation assay: The HTRF® cAMP Gs HiRange assay (PerkinElmer) was performed using CHO cells stably expressing 3HA-tagged-hKOR (OPRK1) or hMOR (OPRM1) as previously described.[7, 63] Briefly, cells were passaged into 384-well low-volume white plates (Greiner Bio-One) at 4000 cells/well with Opti-MEM media (Gibco) containing 1% FBS for 3 hours at 37°. Cells were then treated with 25 µM 4-(3-butoxy-4-methoxybenzyl)imidazolidin-2-one (PDE4 inhibitor) and 20 µM forskolin for 30 minutes at room temperature, followed by a 1 hour incubation of detection reagent provided by the manufacturer at room temperature. Fluorescence was measured at 620 and 665 nm using an Infinite M1000Pro (Tecan) and the cAMP levels were determined based on the ratio of absorbance.

βArrestin2 recruitment assay. The PathHunter hKOR βarrestin assay was performed according to the manufacturer's protocol (DiscoveRx) and as described previously.[7] Briefly, U2OS-β-arrestin2-EFC-hKOR cells were plated in 384-well white plates (Greiner Bio-One) at 5000 cells/well with Opti-MEM media (Gibco) containing 1% FBS overnight at 37°C. The next day, cells were treated with compounds for 90 min at 37°C, followed by a 1-hour incubation of detection reagent at room temperature. Luminescence values were determined by using an Infinite M1000Pro (Tecan).

Data Analysis. Concentration dose response curves (CRCs) were analyzed using Graphpad Prism v.9.0.; they were fit to 3 parameter nonlinear regression analysis to determine potency (EC50) and efficacy (Emax). Compounds that did not converge to regression analysis due to failure to plateau are designated as nc (not converged). Bias analysis was applied using the operational model to determine the $\Delta\Delta\text{Log}(\tau/\text{KA})_{(\text{cAMP}-\beta\text{arr2})}$ as previously described.

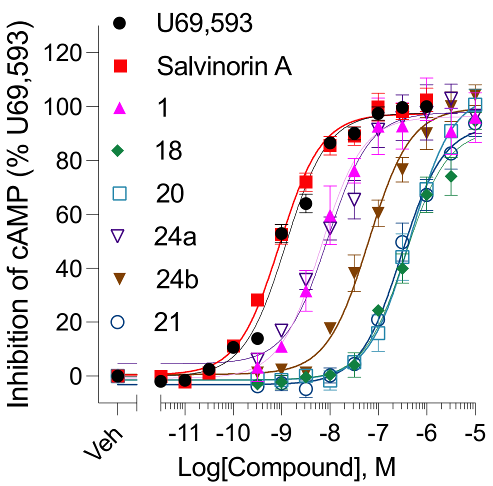

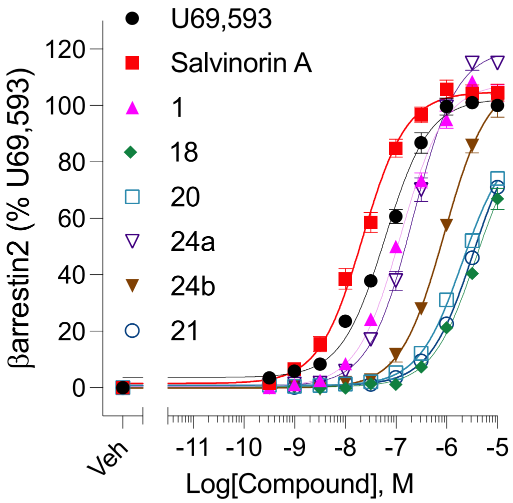

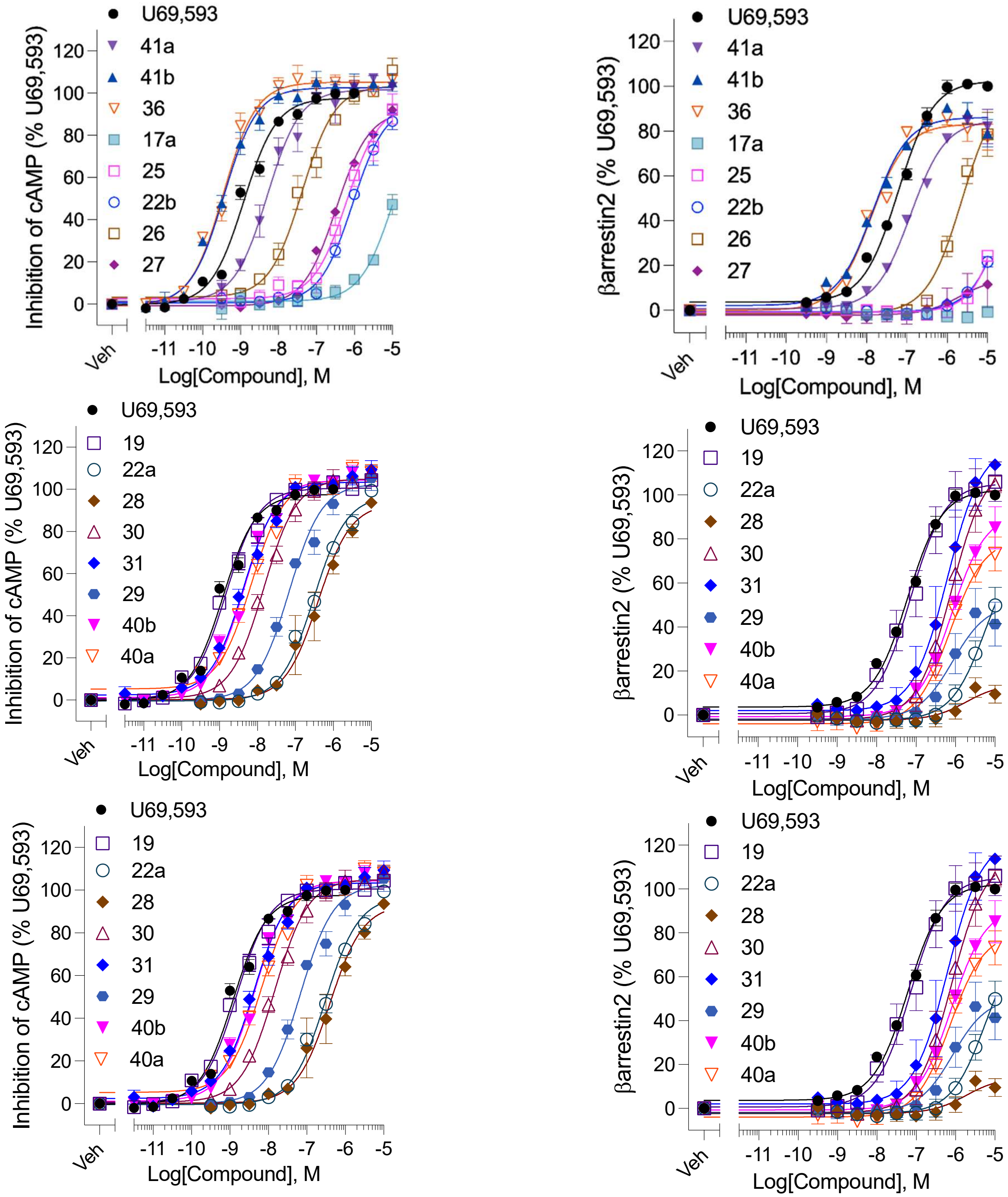

Figure S1. Concentration response curves in the cAMP and βarrestin2 signaling assays. A. Compounds were tested in CHO cells stably expressing hKOR for their ability to inhibit forskolin-stimulated cAMP accumulation (Cisbio HTRF); or B. their ability to stimulate the recruitment of βarrestin2 to hKOR in U2OS cells in the enzyme fragment complementation assay (DiscoveRx PathHunter).[7] Data are normalized to the baseline and the maximum response produced by 10 μM DAMGO and are presented as the mean ± SEM.

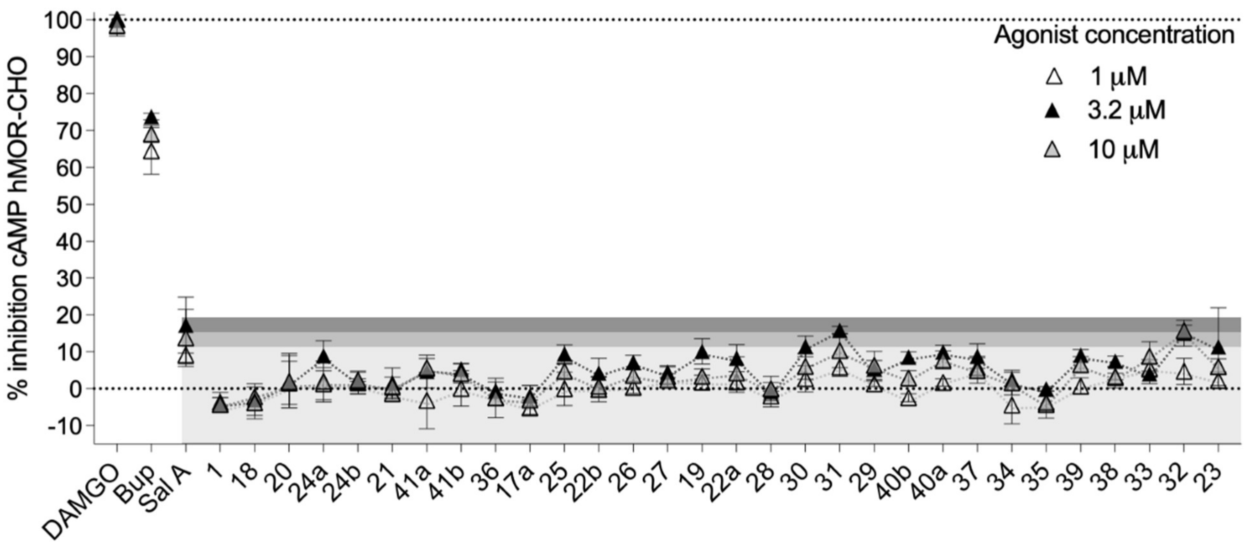

Figure S2. Selectivity against hMOR is demonstrated using a forskolin-stimulated cAMP accumulation assay in CHO cells stably expressing human MOR (OPRM1) as previously described.[63] The % stimulation of MOR is normalized to the baseline and the maximum obtained with 10 μM of the full agonist, DAMGO (D-Ala^2, N-MePhe^4, Gly-ol]-enkephalin) for each compound at 1, 3.2 and 10 μM. Buprenorphine is used as a control to demonstrate the response of a partial agonist in the assay. Data are shown as mean ± SEM, n=3 independent experiments performed in duplicate for all but buprenorphine (n=2).

Table S1. Pharmacological parameters derived from the nonlinear regression analysis of the cAMP and βarrestin2 recruitment assays fit to the curve replicates (n≥3 independent curves). nc: not converged.

	cAMP					βarrestin2					ΔΔLogτ/KA	
	**pEC50**	**SEM**	**Emax**	**SEM**		**pEC50**	**SEM**	**Emax**	**SEM**		**(cAMP/βarr2)**	**Bias Factor**
**U69,593**	8.92	0.036	100			7.22	0.033	100			reference	
**Salvinorin A**	9.04	0.052	97	2		7.66	0.042	105	1		2.073	0.48
**1**	8.19	0.111	96	3		6.90	0.031	108	1		2.921	0.34
**18**	6.44	0.075	91	4		5.42	0.047	91	4		4.392	0.23
**20**	6.33	0.070	105	4		5.69	0.027	88	2		5.896	0.17
**24a**	8.04	0.098	98	3		6.66	0.027	120	1		2.554	0.39
**24b**	7.20	0.066	100	3		6.05	0.030	109	2		3.153	0.32
**21**	6.52	0.066	93	3		5.48	0.030	94	2		4.409	0.23
**41a**	8.30	0.077	102	2		6.91	0.048	85	2		0.754	1.33
**41b**	9.44	0.057	103	2		7.85	0.060	86	2		0.486	2.06
**36**	9.46	0.062	105	2		7.89	0.070	83	2		0.375	2.66
**17a**	4.97	0.226	96	28		nc		(-1)	(1)		–	
**25**	6.20	0.090	95	5		nc		(24)	(1)		–	
**22b**	6.04	0.063	95	4		nc		(22)	(2)		–	
**26**	7.38	0.070	104	3		5.63	0.092	100	7		0.536	1.87
**27**	6.48	0.036	92	2		5.69	0.671	14	8		0.252	3.96
**19**	8.83	0.038	101	1		7.14	0.104	105	4		0.898	1.11
**22a**	6.51	0.046	97	2		5.23	0.182	81	16		1.397	0.72
**28**	6.40	0.101	93	5		5.73	0.413	14	5		0.258	3.88
**30**	7.84	0.051	105	2		6.10	0.098	115	7		0.797	1.26
**31**	8.37	0.043	103	1		6.20	0.120	123	8		0.399	2.50
**29**	7.17	0.063	103	3		6.07	0.204	51	6		0.316	3.16
**40b**	8.36	0.039	104	1		6.10	0.064	92	4		0.134	7.44
**40a**	8.16	0.058	105	2		6.13	0.084	80	4		0.102	9.83
**37**	9.38	0.056	105	2		7.37	0.103	80	3		0.116	8.60
**34**	8.06	0.051	107	2		6.22	0.070	72	3		0.105	9.52
**35**	7.34	0.041	107	2		5.74	0.087	67	4		0.182	5.48
**39**	8.90	0.034	105	1		7.01	0.060	83	2		0.184	5.43
**38**	8.53	0.040	100	1		6.61	0.062	98	3		0.439	2.28
**33**	7.02	0.061	92	2		5.37	0.072	131	9		1.946	0.51

**32**	9.58	0.039	99	1		7.70	0.075	106	3		0.732	1.37
**23**	5.47	0.104	102	10		nc		(9)	(1)		–	
**nc: not converged to nonlinear regression; values in parentheses are the maximum stimulation seen at 10 μM.**												

Materials and Methods (Chemistry)

All reactions were carried out under a positive pressure of nitrogen or argon unless otherwise noted. Glassware was oven-dried at 120 °C for a minimum of 12 hours or flame-dried with a propane torch under high vacuum (<1 torr). Commercially available chemicals were used without further purification unless otherwise noted. ZnI_2 was dried briefly under vacuum with a heat gun prior to use. Solutions of TBSOTf and TMSOTf were purchased from Oakwood chemicals and used within 6 months of opening. Anhydrous dichloromethane (DCM, CH_2Cl_2) was purchased from Sigma-Aldrich stored over molecular sieves or argon-sparged and passed through an activated alumina column. Tetrahydrofuran, chlorobenzene, trifluorotoluene, chloroform (contains amylenes as stabilizer, ≥99%), and 1,4-dioxane were purchased from Sigma-Aldrich and used as received. Deuterated chloroform ($CDCl_3$) and benzene (C_6D_6) and acetone were purchased from Cambridge Isotope Laboratories. $CDCl_3$ was filtered through oven-dried basic alumina prior to use. Reactions were monitored by thin layer chromatography (TLC) using precoated silica gel plates from EMD Chemicals (TLC Silica gel 60 F254, 250 μm thickness) and monitored by UV light (254 nm) or stained with anisaldehyde or potassium permanganate. Silica gel column chromatography (SGC) was performed over silica gel 60 (particle size 0.04-0.063 mm) from EMD Chemicals. Specific optical rotations were measured on a Rudolph Research Analytical Autopol® III automatic polarimeter in a cuvette of 50 mm optical pathlength with sodium D line (589 nM) and concentrations (c) given in grams of solute per 100 mL of solution. Melting points were measured on a Laboratory Devices Mel-Temp II in open glass capillaries and are uncorrected. Routine liquid chromatography-mass spectra (LC-MS) were obtained on an Agilent 6120 Single Quadrupole System with an electron-spray ionization (ESI) probe. Gas chromatography-mass spectra (GC-MS) were obtained on an Agilent 7820A/5975 GC/MSD system with helium as the carrier gas. Mass measurements for high-resolution mass spec (HRMS) were performed on a Waters Xevo G2-XS TOF calibrated against sodium formate clusters and using a LeuEnk lockmass. Expected monoisotopic masses were calculated using MassLynx 4.1 and the m/z values for calibrant and lockmass were MassLynx-default values. Enantiomeric excess of chiral samples were analyzed on a Waters Waters UPC2 SFC with a Diacel IH column (3 um, 4.6x250 mm) under isocratic conditions (3.3 mL/min, 3% MeOH / CO_2, 1600 psi backpressure) at 30 °C. The enantiomers were detected by UV light (226 nm). The enantiomers were detected by 212 nm UV light. NMR spectra were recorded on Varian-400, Bruker DPX-400, DRX-500, and DRX-600 (equipped with a CryoProbeTM) spectrometers using residual solvent as an internal standard ($CDCl_3$ at 7.26 ppm ^1H NMR, 77.16 ppm ^{13}C NMR; C_6D_6 at 7.16 ^1H NMR, 128.06 ppm ^{13}C NMR; acetone-d^6 at 2.09 ppm ^1H NMR, 205.87 ppm & 30.60 ppm ^{13}C NMR). The following abbreviations (or combinations thereof) were used to denote multiplicities: s = singlet, d = doublet, t = triplet, q = quartet, quint = quintet, m = multiplet (complex pattern), br = broad signal. Raw FID files were processed and analyzed using MestReNova v14.2.0 from Mestrelab Research S.L.

Abbreviations: THF = tetrahydrofuran, TBSOTf = *tert*-butyldimethylsilyl trifluoromethanesulfonate, TMSOTf = trimethylsilyl trifluoromethanesulfonate, TBAF = tetrabutylammonium fluoride, KHMDS = potassium bis(trimethylsilyl)amide, NIS = *N*-iodosuccinimide, Na_2SO_4 = sodium sulfate, $Cu(OTf)_2$ = copper(II) trifluoromethanesulfonate, TLC = thin layer chromatography, SGC = silica gel chromatography, MeOH = methanol, EtOH = ethanol, H_2O = water, tol = toluene, PTSA = *para*-toluene sulfonic acid, H_2SO_4 = 18 M sulfuric acid, RBF = round-bottom flask, TMB = trimethoxy benzene, HBr = hydrobromic acid, H_3PO_4 = phosphoric acid, TsCl = *para*-toluene-sulfonyl chloride, Et_3N = triethyl amine, Ac_2O = acetic anhydride, Et_2O = diethyl ether, EtOAc= ethyl acetate, Pd_2dba_3 = tris(dibenzylideneacetone)dipalladium(0), $Pd(PhCN)_2Cl_2$ = palladium(II) bisbenzonitrile dichloride, MTBE = methyl *tert*-butyl ether, CuTC = copper(I) thiophene carboxylate.

Scheme S1. Evans's salvinorin A synthesis[14]

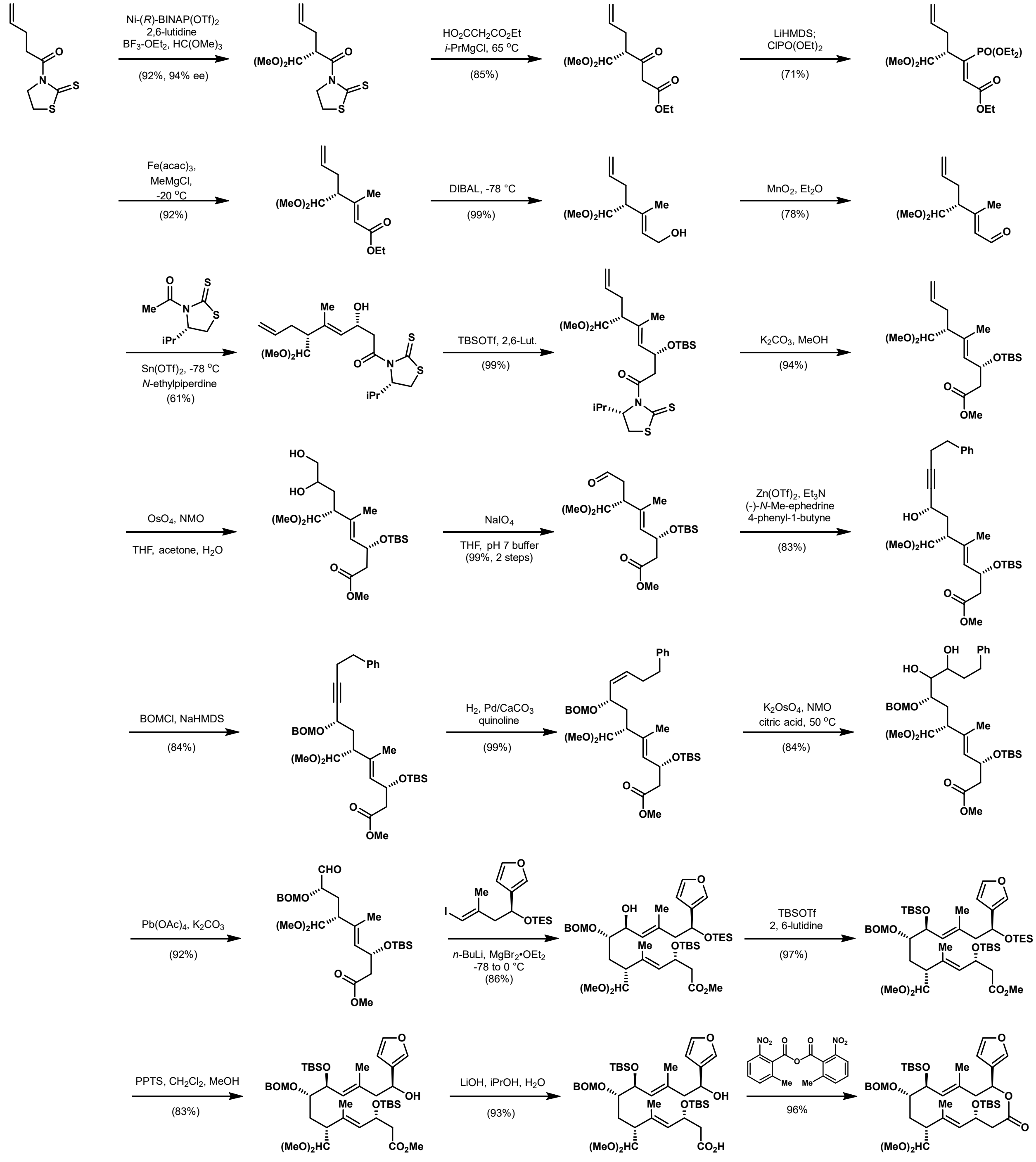

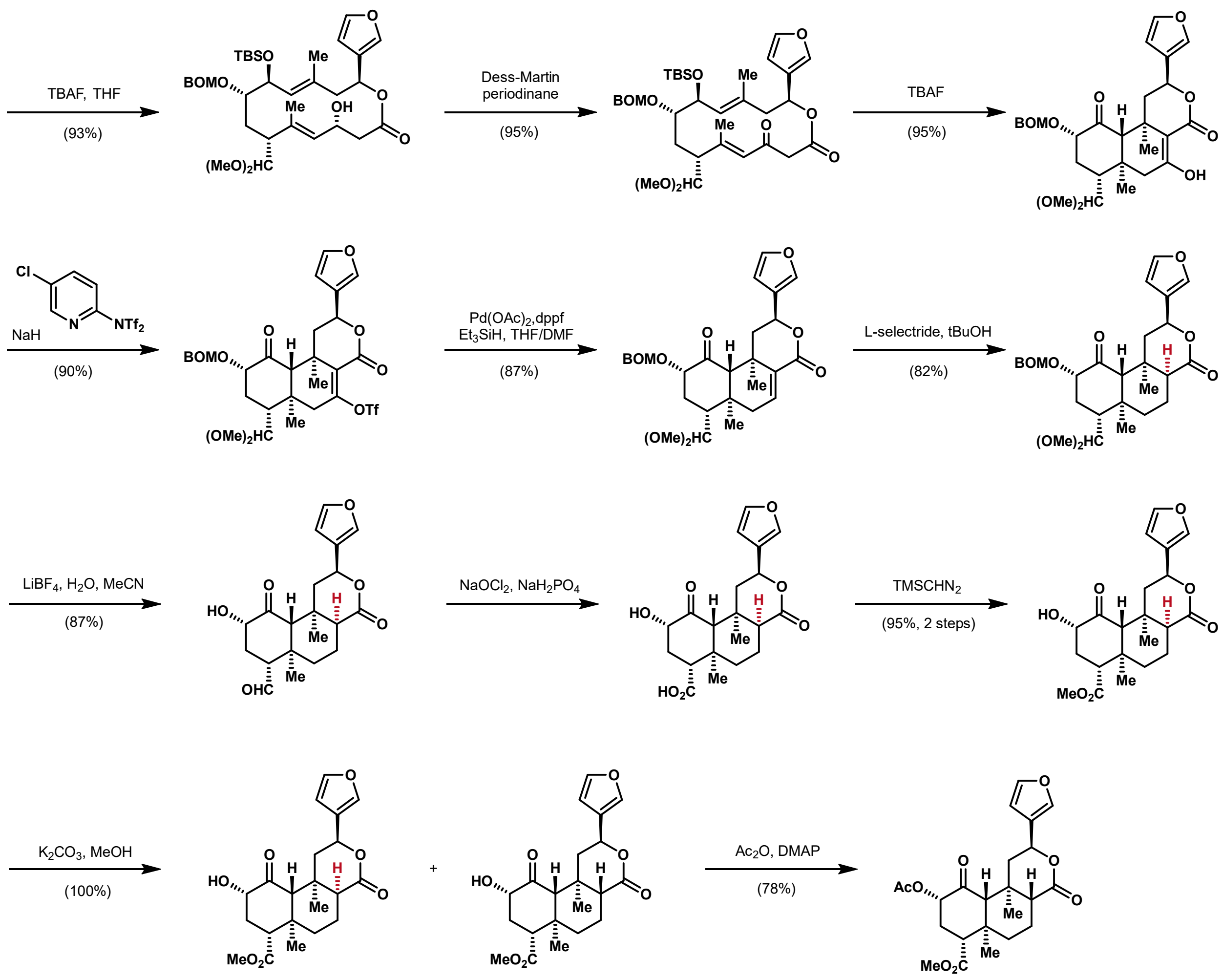

8-*epi*-SalB 2.5:1 SalB SalA

Scheme S2. Hagiwara's 1st generation salvinorin A synthesis[15]

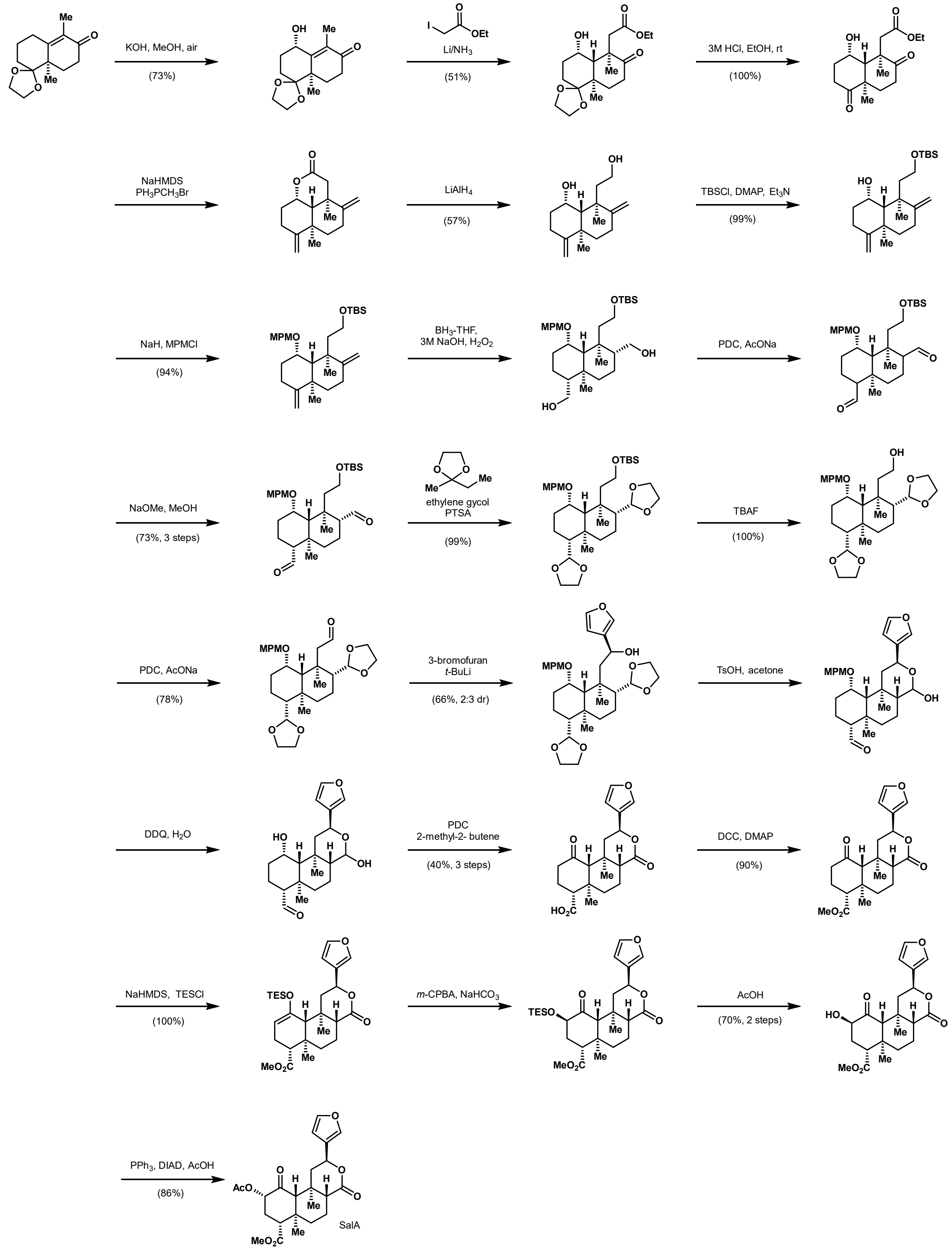

Scheme S3. Hagiwara's 2nd generation salvinorin A formal synthesis[16]

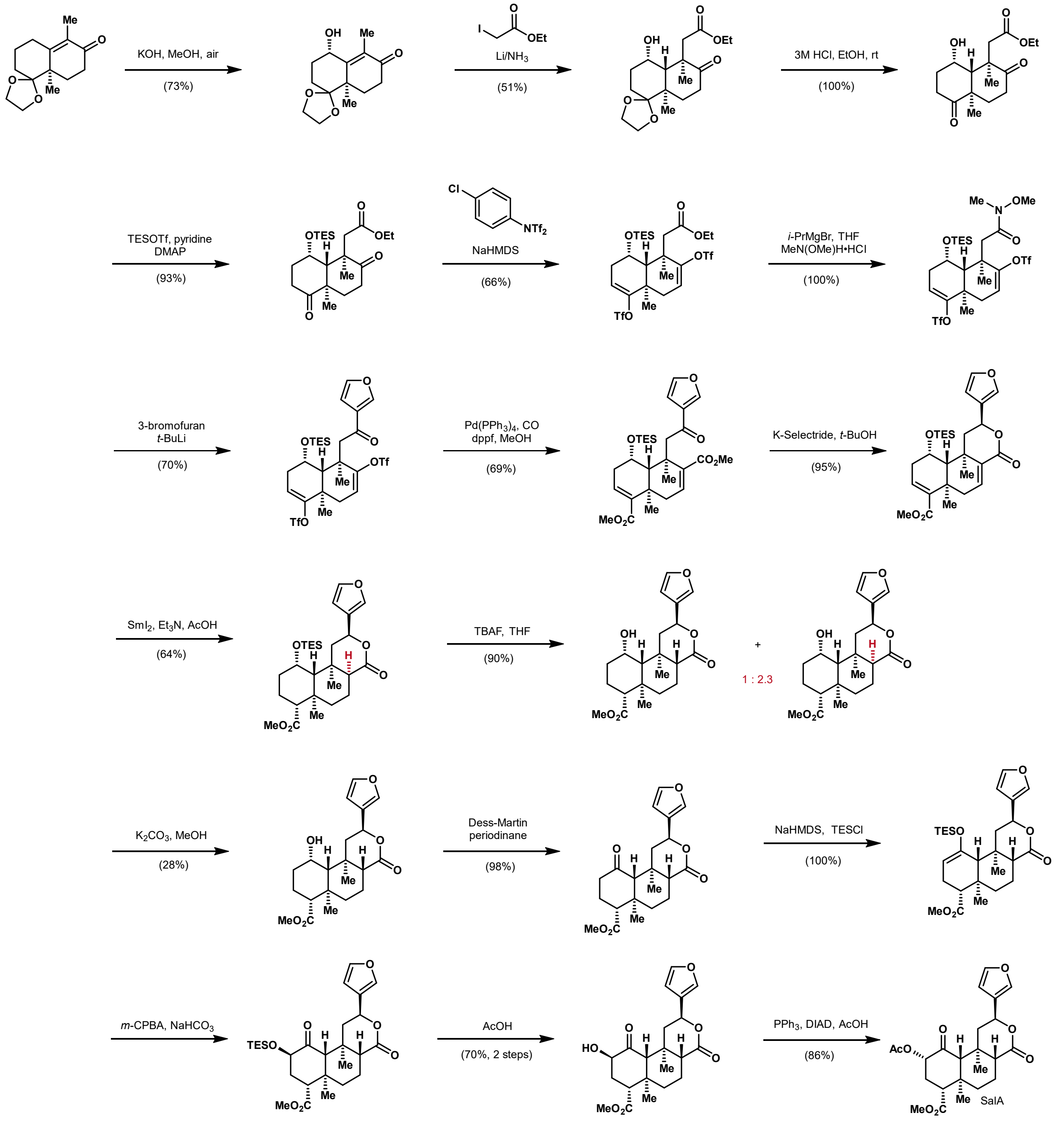

Scheme S4. Forsyth's salvinorin A synthesis[17]

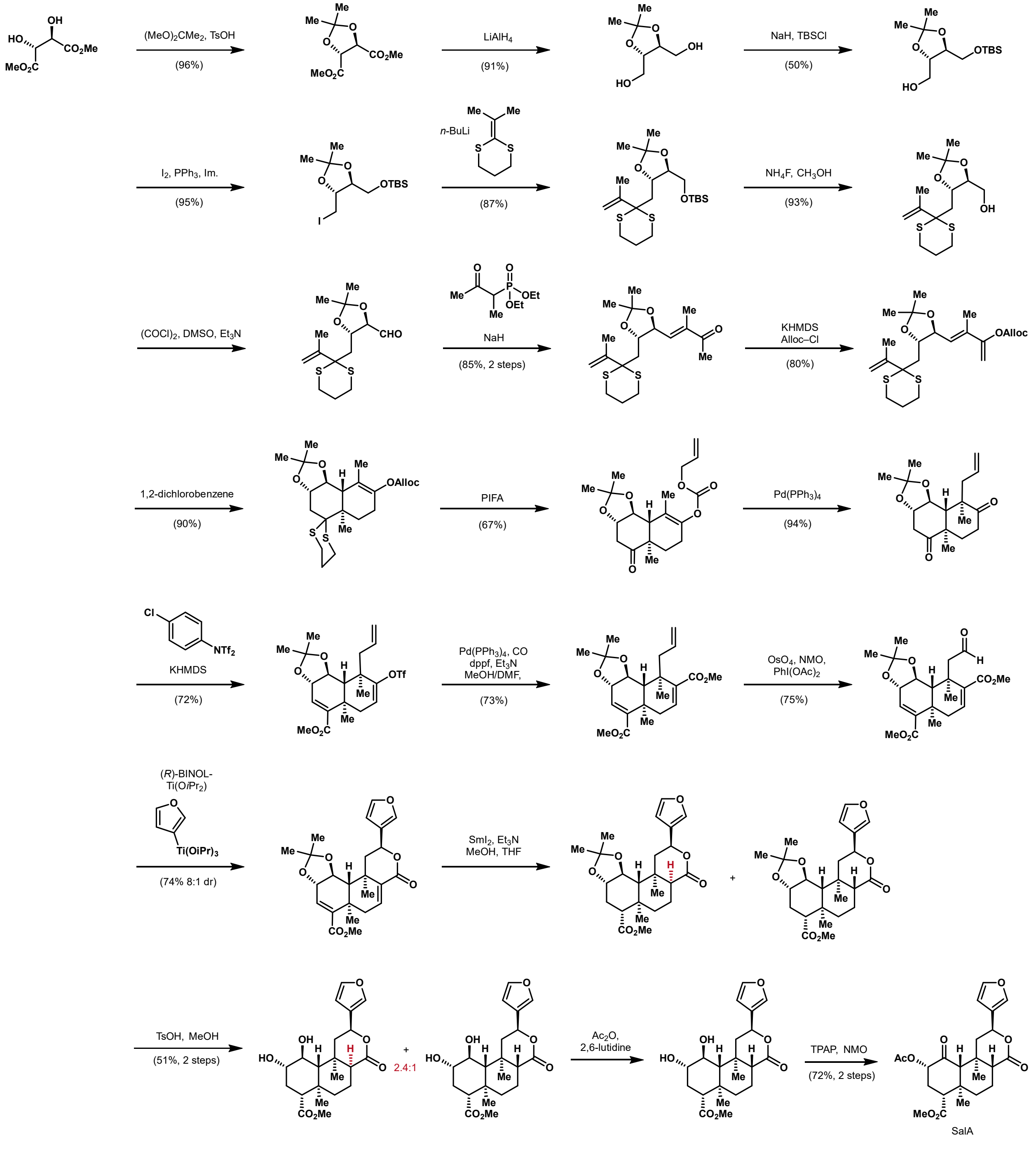

Scheme S5. Metz's 1st generation salvinorin A synthesis[18]

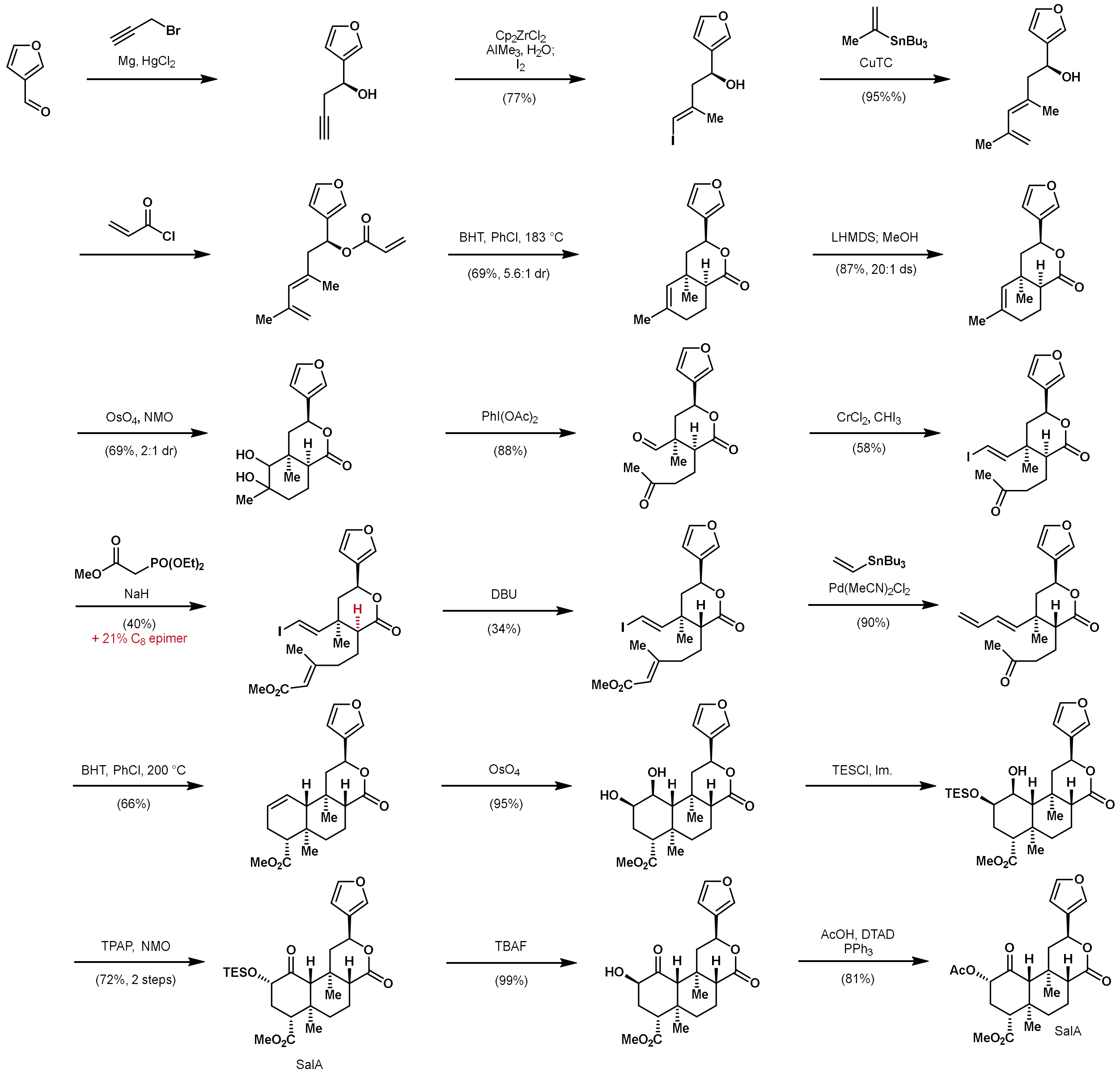

Scheme S6. Maier's salvinorin A formal synthesis[19]

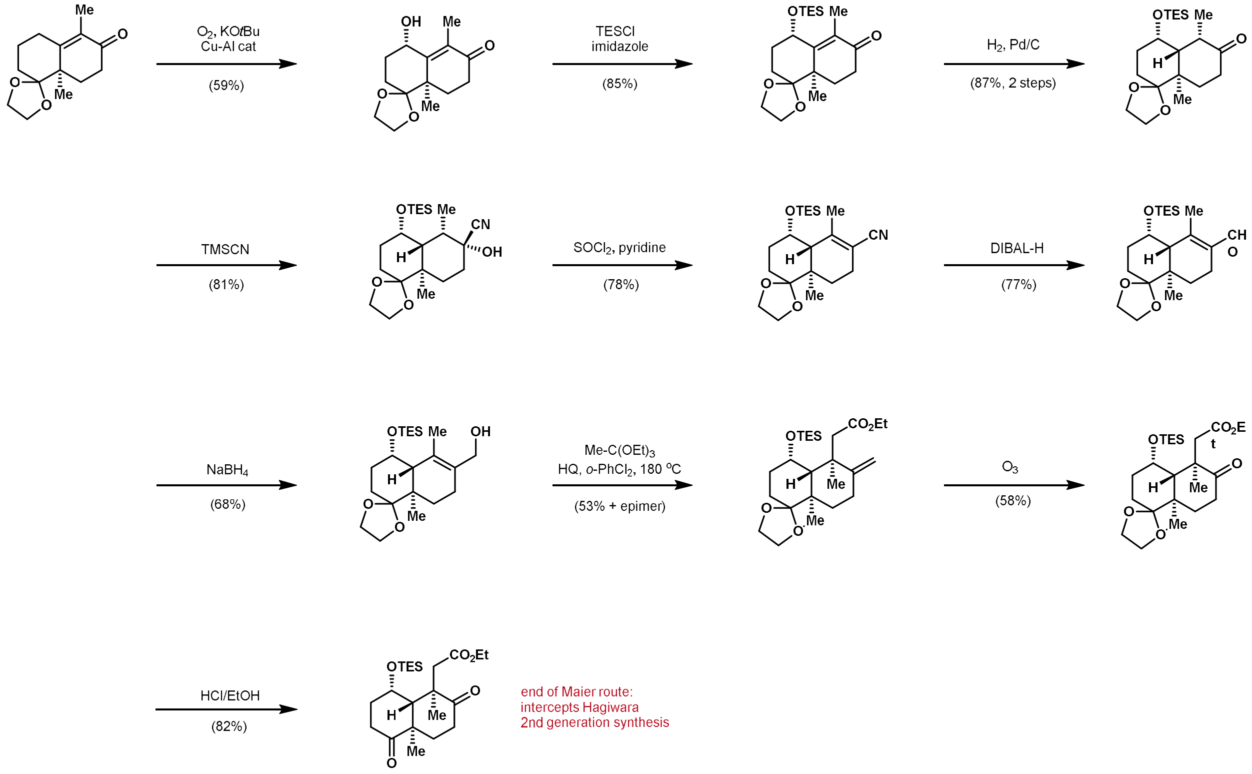

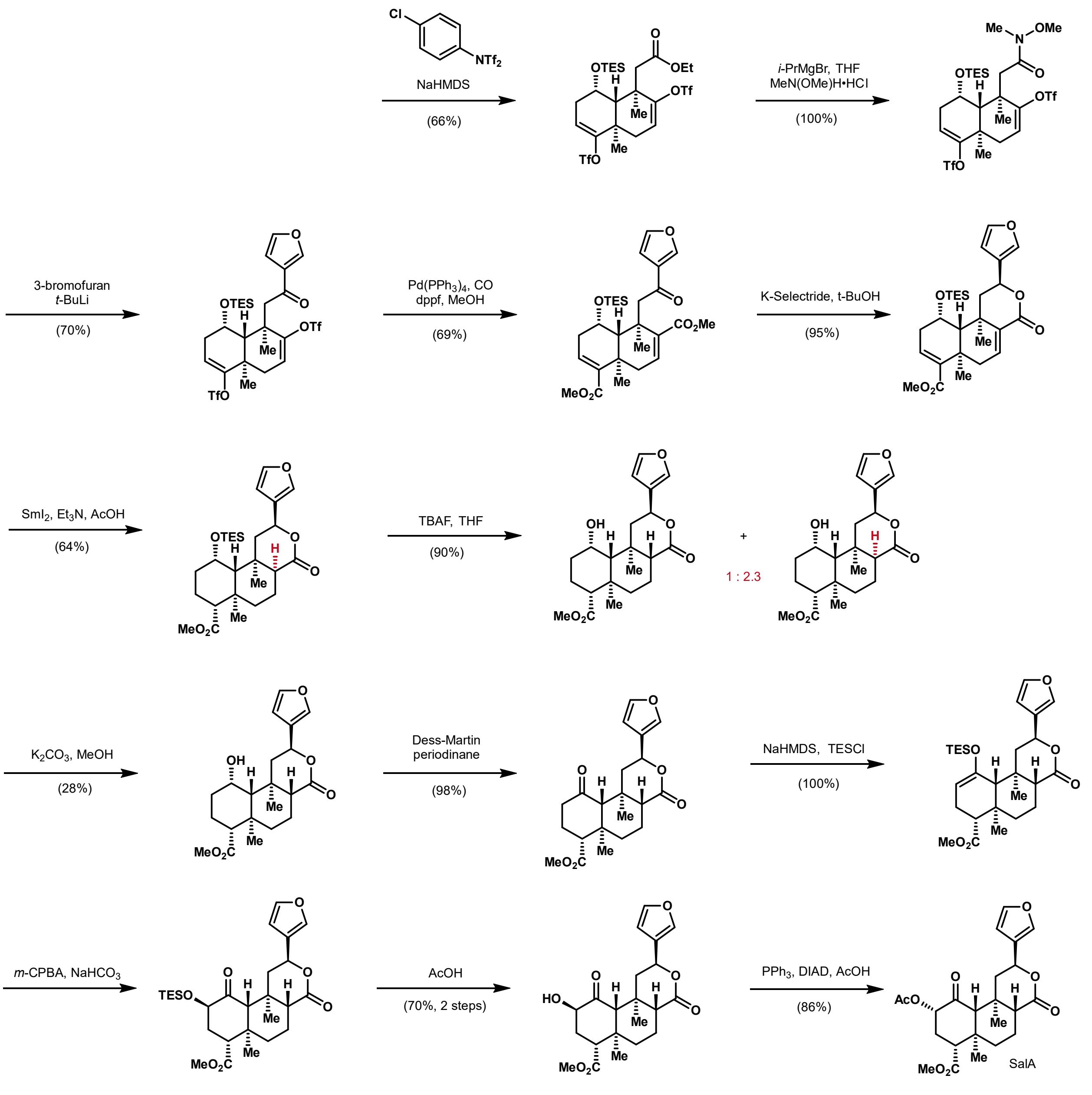
Cl
NTf2
NaHMDS
(66%)
OTES
H
O
OEt
OTf
Me
Me
TfO
i-PrMgBr, THF
MeN(OMe)H•HCl
(100%)
Me
N
OMe
3-bromofuran
t-BuLi
(70%)
Pd(PPh3)4, CO
dppf, MeOH
(69%)
CO2Me
MeO2C
K-Selectride, t-BuOH
(95%)
SmI2, Et3N, AcOH
(64%)
TBAF, THF
(90%)
OH
+
1 : 2.3
K2CO3, MeOH
(28%)
Dess-Martin
periodinane
(98%)
NaHMDS, TESCl
(100%)
TESO
m-CPBA, NaHCO3
AcOH
(70%, 2 steps)
HO
PPh3, DIAD, AcOH
(86%)
AcO
SalA

Scheme S7. Metz's 2nd generation salvinorin A formal synthesis[20]

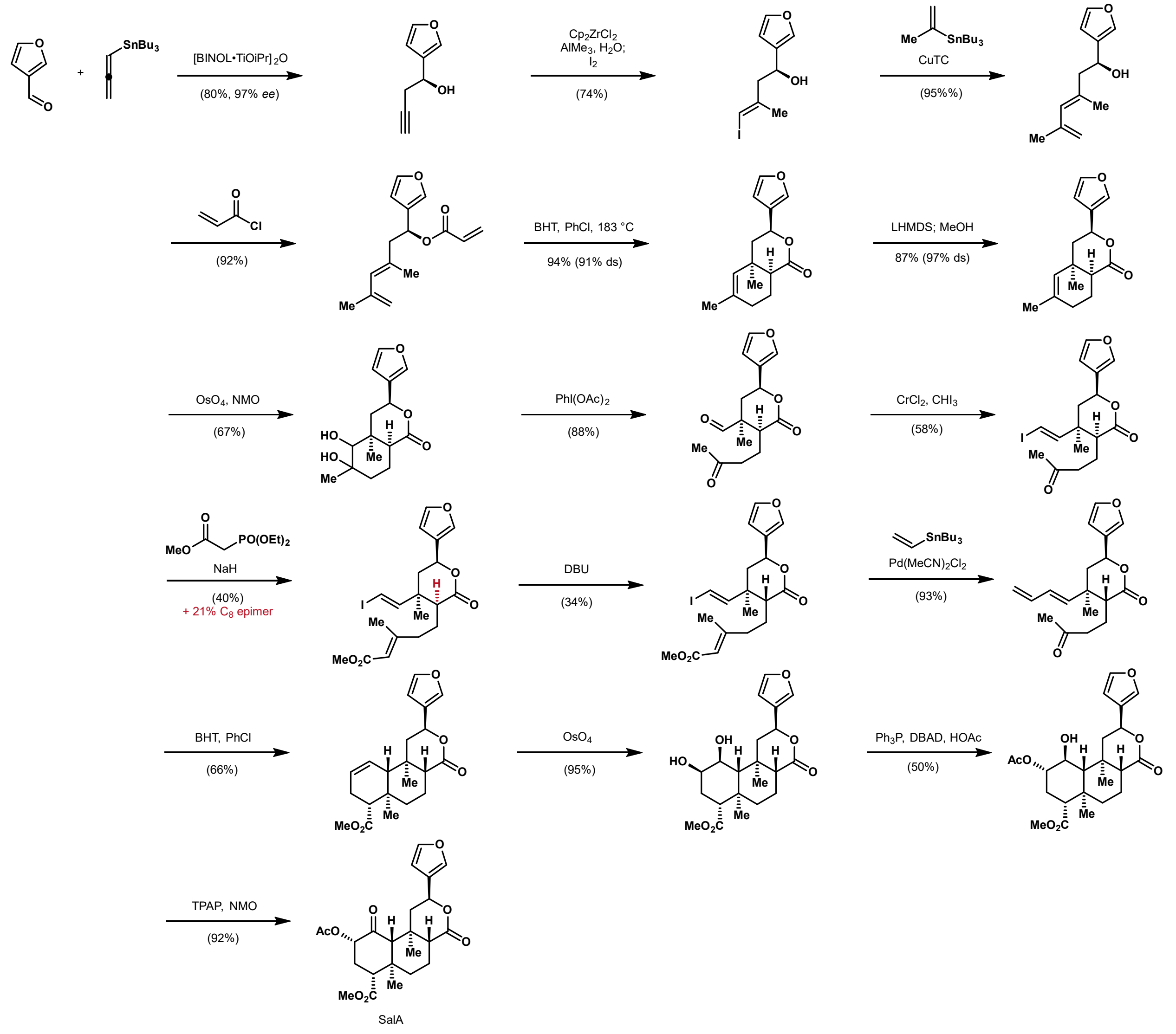

Scheme S8. Prisinzano's stabilized salvinorin A scaffold[23]

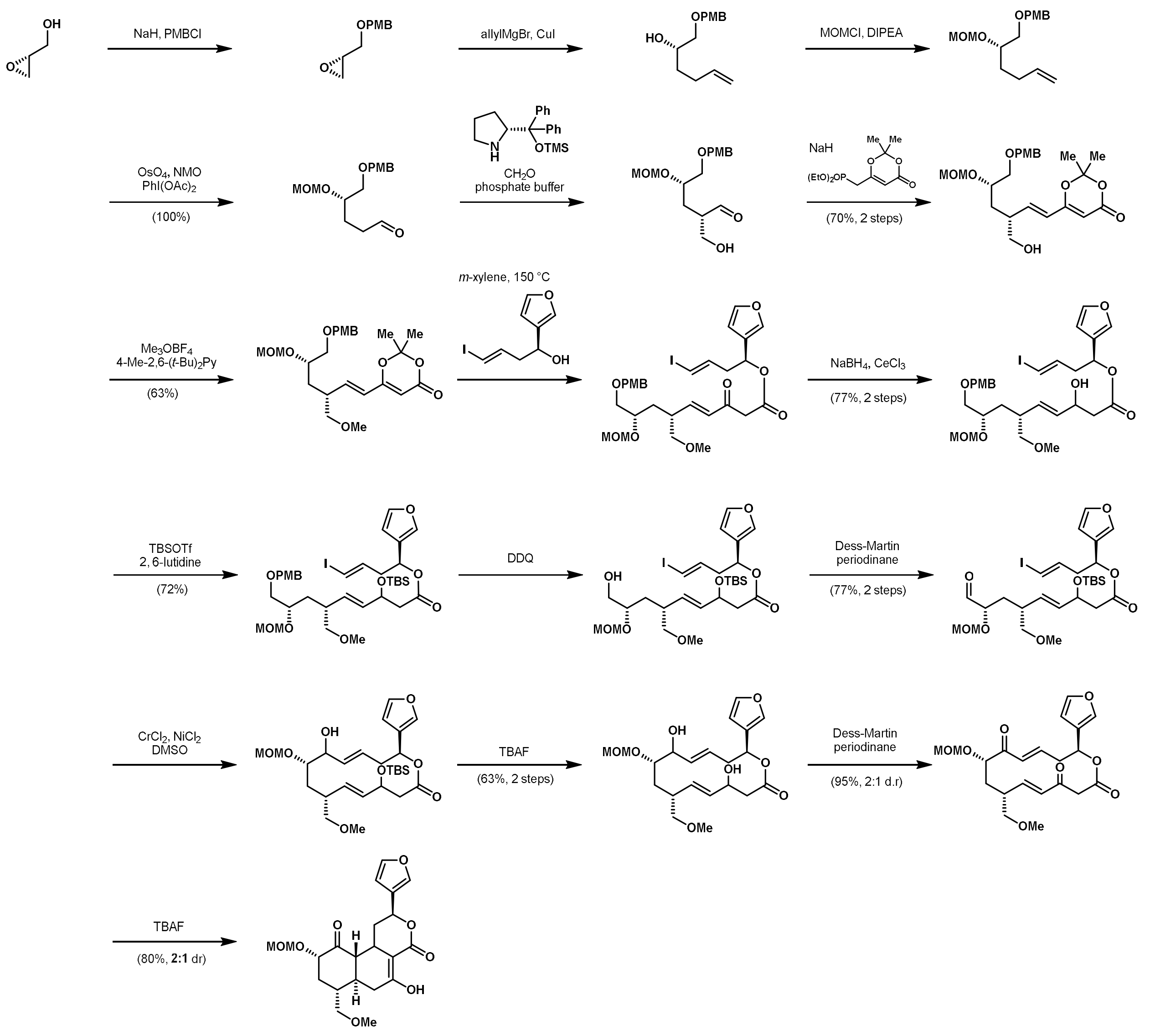

Scheme S9. Rook's approach[38]

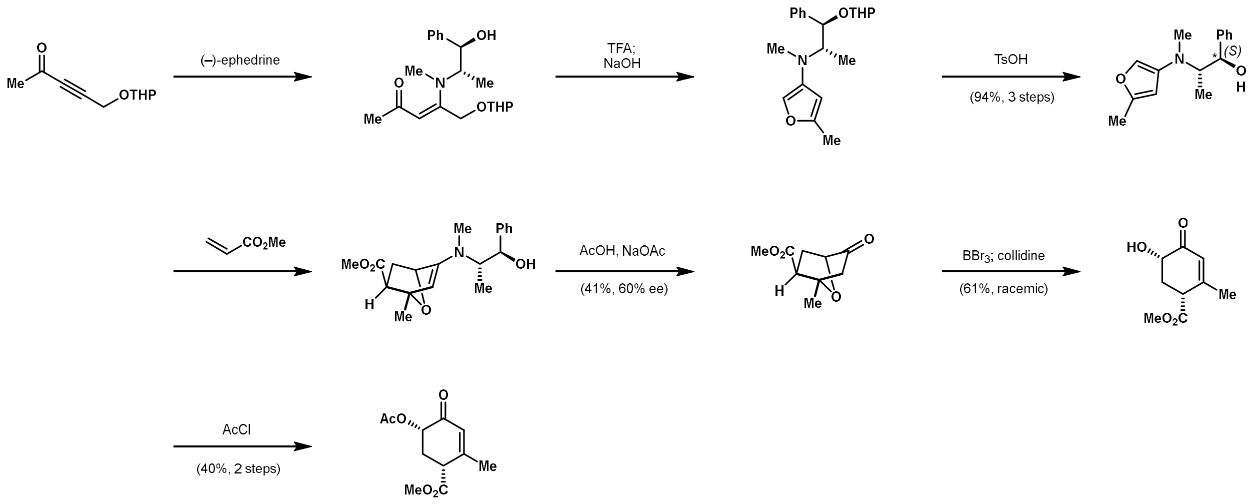

Scheme S10. Perlmutter's approach[21]

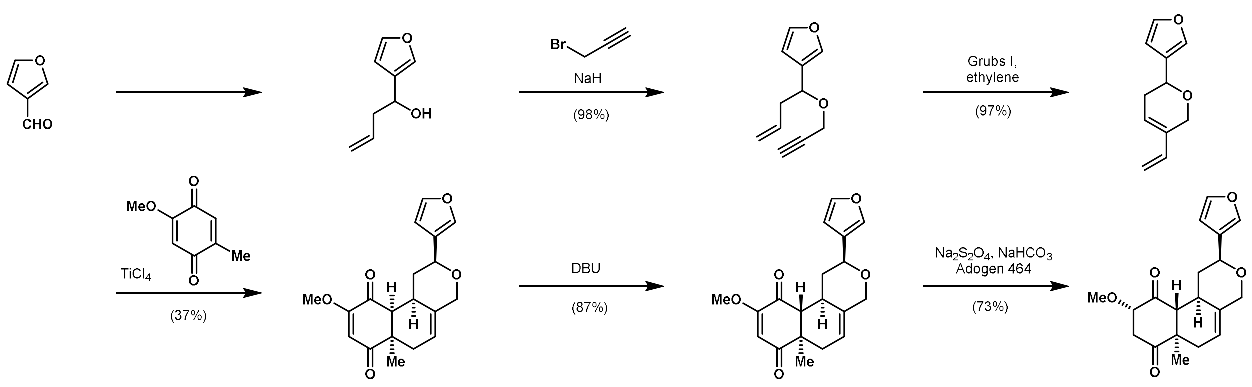

Scheme S11. Synthesis of *rac*-20-nor-SalA[24] and *rac*-O6C-20-nor-SalA[25]

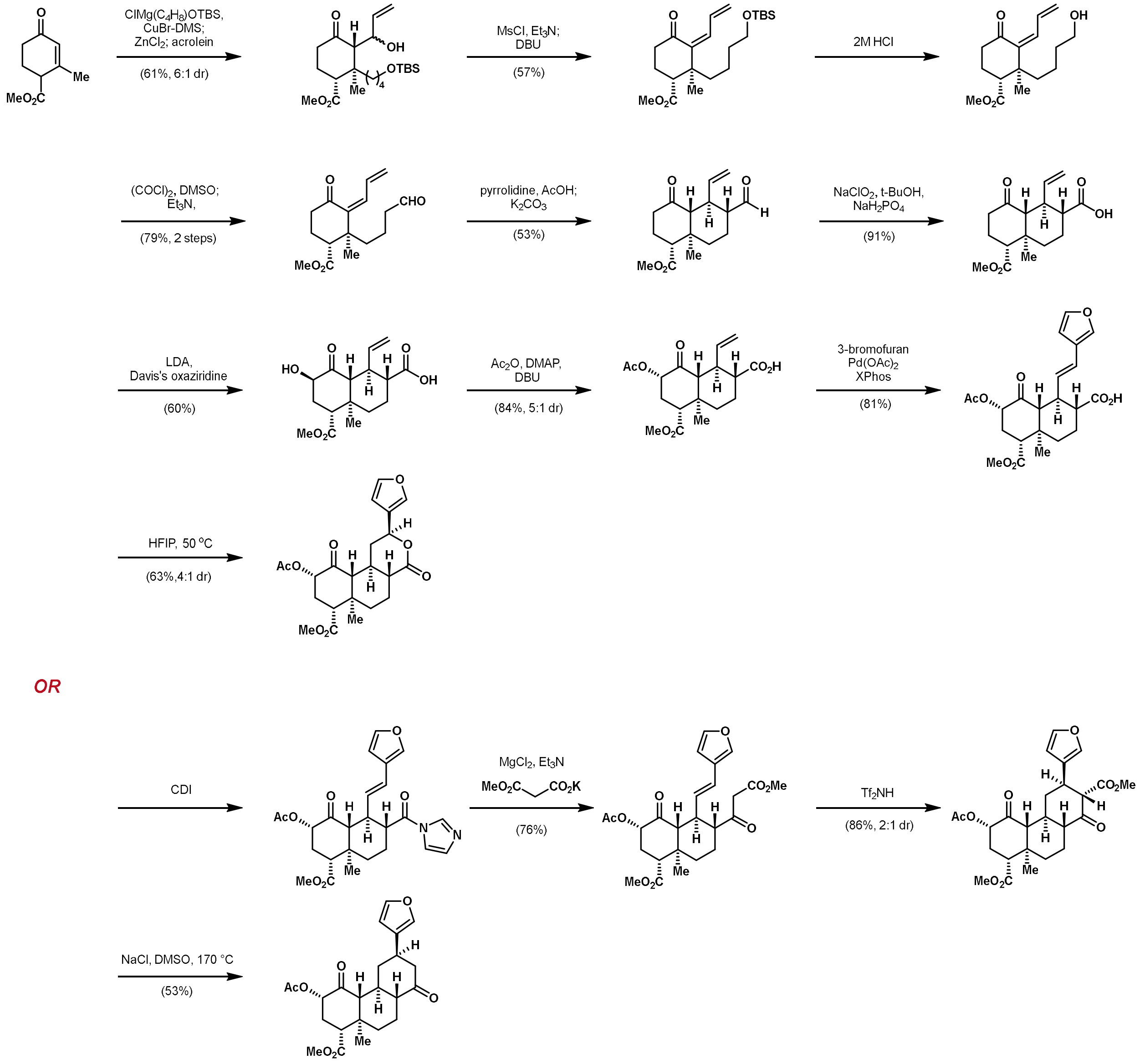

Scheme S12. This work: enantioselective synthesis of (−)-O6C-20-nor-SalA

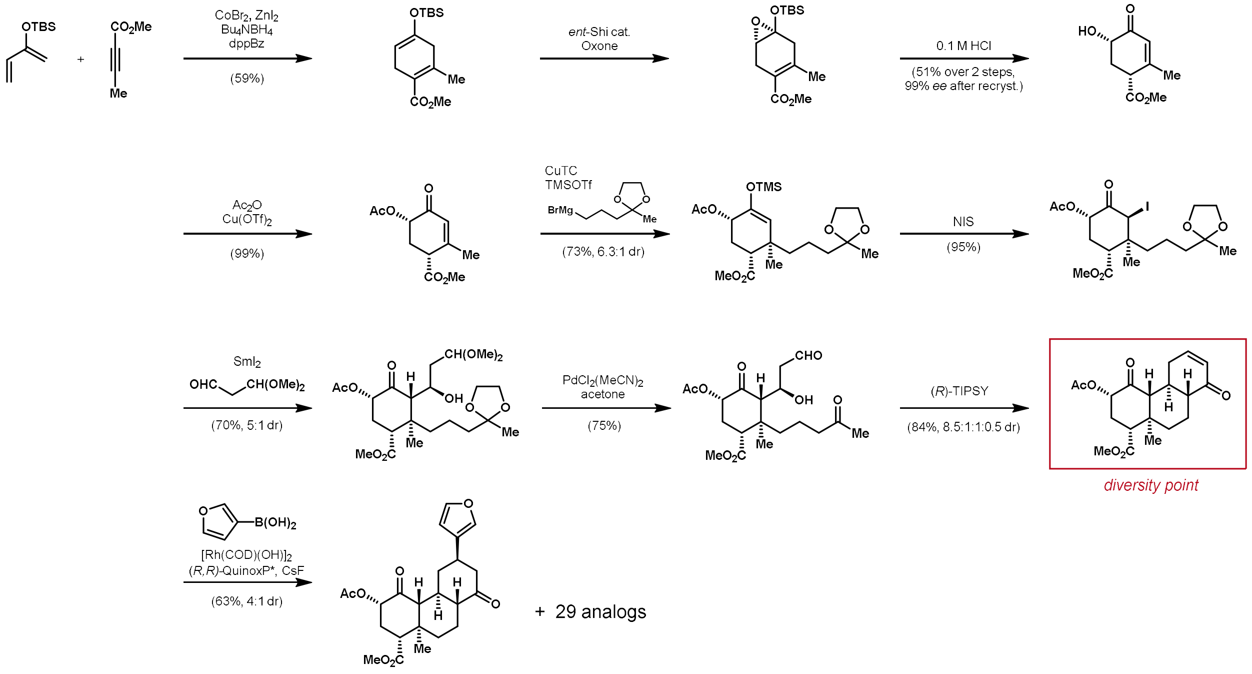

Diene **S1**

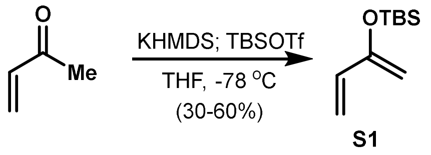

To a flame-dried 1-L RBF, KHMDS (100 mL of 1 M solution in THF, 1.05 equiv., 100.0 mmol) was added to THF (200 mL) under Ar atmosphere and the solution was cooled to –78 ºC. Freshly distilled methyl vinyl ketone (6.63 g, 7.88 mL, 1 equiv., 94.5 mmol) was then added at the rate of 0.26 mL/min. Upon completion of the addition, the mixture was left to stir for 0.5 h. Subsequently, TBSOTf (26.2 g, 22.8 mL, 1.05 equiv., 99.3 mmol) was added to the solution at the rate of 0.76 mL/min, followed by 0.5 h of vigorous stirring. Afterwards, Et_3N (18 mL) was added at –78 ºC. The reaction was then removed from the cooling bath and H_2O (300 mL) was added immediately. The mixture was warmed up to 22 ºC and extracted 3 times with pentane (approx. 200 mL each). The combined organic layers were washed with brine and dried over Na_2SO_4. The mixture was concentrated to a light-yellow oil. The crude concentrate was purified via flash column chromatography (200 mL of silica) eluting with pentane. The fractions containing the product were concentrated, the yield of **S1** varied from 30-60% depending on the quality of the TBSOTf and THF used. The NMR spectra of the isolated material match literature report.[64] **S1** is also listed commercially.

Note 1: Impurities of di-*tert*-butyldimethylsilyl ether and another unidentified silylated impurity were sometimes present in the combined fractions up to 20%, these impurities could be carried through the subsequent reaction and removed upon purification of cycloadduct **4**.

^{1}H NMR (600 MHz, $CDCl_3$) δ 6.22 (dd, *J* = 16.9, 10.5 Hz, 1H), 5.54 (dd, *J* = 16.9, 1.9 Hz, 1H), 5.11 (m, 1H), 4.38 – 4.33 (m, 2H), 1.00 (s, 9H), 0.21 (s, 6H).

^{13}C NMR (151 MHz, $CDCl_3$) δ 155.26, 134.97, 114.63, 96.22, 25.94, 18.44, -4.53.

R_f: 0.64 in pentane (UV active, stained against $KMnO_4$)

GC-MS: (GC/MSD; HP-5MS UI; 9.785 psi; flow rate 1.2 mL/min; inlet temperature 250 °C; column temperature 50 °C at 0 min, then 25 °C/min to 100 °C, then 35 ºC/min to 350 ºC): tR = 3.033 min. (EI, 70 eV): m/z (%): 184.1 (1), 128.0 (72) , 127.0 (100), 75.0 (70).

Alkyne **S2**

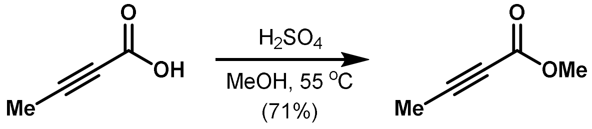

S2

To a 50 mL pressure vial was added 2-butynoic acid (5.0 g, 59.9 mmol, 1 equiv.) and dissolved in MeOH (15 mL). 18 M H_2SO_4 (500 µL) was then added, and the mixture was heated in an oil bath to 55 ºC for 12 hours. The reaction vessel was then removed from the oil bath, cooled to 22 ºC and saturated aqueous sodium bicarbonate solution (30 mL) was carefully added. This mixture was then poured into a separatory funnel; more sodium bicarbonate solution (50 mL) and pentane (50 mL) were added. The layers were shaken together and then separated. The aqueous layer was extracted two more times with pentane (2 × 50 mL) and then the combined organic layers were washed with brine (100 mL) and dried over Na_2SO_4. The Na_2SO_4 was removed by filtration and the organic layer was carefully concentrated under reduced pressure to yield methyl 2-butynoate **S2** (4.2 g, 42.8 mmol, 71%) as a colorless oil. The ^{1}H NMR and ^{13}C NMR spectra of this material matched literature characterization.[65] **S2** is also listed commercially.

Note: This compound has an extremely unpleasant smell and should be kept inside a ventilated fumehood.

^{1}H NMR (500 MHz, $CDCl_3$) δ 3.75 (s, 3H), 1.99 (s, 3H).

^{13}C NMR (100 MHz, $CDCl_3$) δ 154.31, 85.89, 72.26, 52.71, 3.89.

R_f: 0.45 in 10% Et_2O in hexanes (stained with $KMnO_4$)

GC-MS: (GC/MSD; HP-5MS UI; 9.785 psi; flow rate 1.2 mL/min; inlet temperature 250 °C; column temperature 50 °C at 0 min, then 25 °C/min to 100 °C, then 35 ºC/min to 350 ºC): tR = 4.020 min. (EI, 70 eV): m/z (%): 98.0 (2), 70.0 (15), 67.0 (100)

Bromo ketal **S3**

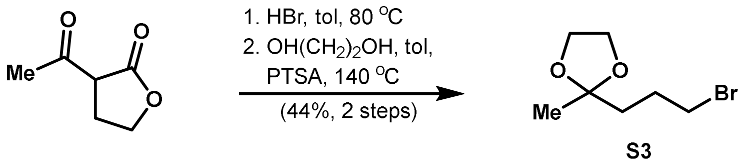

To a 500 mL RBF 15 mL acetyl butyro lactone (17.82 g, 139.1 mmol, 1 equiv.) was added followed by hydrobromic acid 48 wt% by H_2O (25 mL) and toluene (60 mL). The reaction mixture was then placed in an 80 ºC oil bath and stirred with ample outlet for the carbon dioxide evolution for 1.5 hours. The RBF was then removed from the oil bath, quenched by addition of H_2O (100 mL) and extracted with Et_2O (4 × 100 mL). The organic layers were combined and washed with brine (200 mL) and dried over Na_2SO_4. The solvent was evaporated to yield an orange oil that was used immediately without purification. The oil from the reaction was then transferred to a two-necked 2 L RBF and dissolved in toluene (450 mL). Ethylene glycol (34.5 g, 30 ml, 556 mmol, 4 equiv.) was then added to the flask followed by *p*-toluene sulfonic acid (2.4 g, 13.9 mmol, 0.1 equiv.). The flask was fitted with a Dean-Stark apparatus and a reflux condenser, insulated by covering with aluminum foil, and placed in a pre-heated 135 ºC oil bath. The solution was refluxed for 12 hours, draining the Dean-Stark of H_2O as needed. The reaction flask was then removed from the oil bath, quenched with H_2O (300 mL) and diluted with Et_2O (200 mL). The layers were separated and aqueous layer was extracted twice with Et_2O (2 x 200 mL). The combined organic layers were washed with brine (300 mL) and dried over Na_2SO_4. After filtration, the organic solution was then concentrated under reduced pressure to yield a brown oil. The crude mixture was purified by flash column chromatography with a gradient of 0 → 20% EtOAc in hexanes. The fractions containing product were collected and concentrated to yield bromo ketal **S3** (12.7 g, 60.7 mmol, 44% over two steps) as a colorless oil. The ^{1}H NMR and ^{13}C NMR spectra of the isolated material matched literature characterization.[66] **S3** is also listed commercially.

Note 1: This product slowly turns brown even when stored in the freezer. For the best results in the synthesis of the Grignard reagent **10**, this product should be used immediately or passed through a silica plug prior to use.

^{1}H NMR (400 MHz, $CDCl_3$) δ 4.00 – 3.88 (m, 4H), 3.44 (t, *J* = 6.8 Hz, 2H), 2.04 – 1.92 (m, 2H), 1.83 – 1.75 (m, 2H), 1.32 (s, 3H).

^{13}C NMR (100 MHz, $CDCl_3$) δ 109.66, 64.85, 37.72, 34.16, 27.62, 24.11.

R_f: 0.45 in 20% Et_2O in hexanes (stained red with anisaldehyde)

GC-MS : (GC/MSD; HP-5MS UI; 9.785 psi; flow rate 1.2 mL/min; inlet temperature 250 °C; column temperature 50 °C at 0 min, then 25 °C/min to 100 °C, then 35 ºC/min to 350 ºC): tR = 4.101 min. (EI, 70 eV): m/z (%): 195.0 (20), 193.0 (20), 122.9 (4), 120.9 (4), 87.0 (100)

Aldehyde **12**

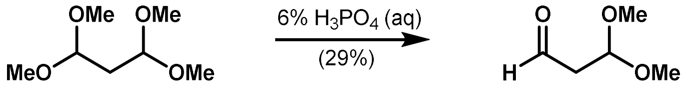

12

1,1,3,3-tetramethoxy propane (3 mL, 18.4 mmol, 1 equiv.) was combined with 6% aqueous phosphoric acid (1.5 mL) in four different vials (12 mL in total). These vials were then stirred vigorously overnight at 22 ºC. Approximately 12 hours later, TLC indicated product formation but starting material remained. These vials were combined and diluted with Et_2O (100 mL). Solid Na_2CO_3 was added until the acid was neutralized, measured by pH or visually by the cessation of gas evolution. This yellow mixture was then filtered and carefully concentrated to an oil as aldehyde **12** is volatile. The crude oil is purified by silica gel flash column chromatography eluting with 10 → 40% Et_2O in pentane. The fractions containing the product were combined and concentrated to yield **12** (2.5 g, 21.2 mmol, 29% yield) as a clear oil. The ^1H NMR and ^{13}C NMR matched literature characterization.[67] **12** is also listed commercially.

^1H NMR (400 MHz, $CDCl_3$) δ 9.74 (t, J = 2.3 Hz, 1H), 4.85 (t, J = 5.5 Hz, 1H), 3.38 (s, 6H), 2.72 (dd, J = 5.5, 2.2 Hz, 2H).

^{13}C NMR (100 MHz, $CDCl_3$) δ 199.62, 100.47, 53.74, 47.18.

R_f: 0.73 in 70% EtOAc in hexanes (stained with PMA)

GC-MS : (GC/MSD; HP-5MS UI; 9.785 psi; flow rate 1.2 mL/min; inlet temperature 250 °C; column temperature 50 °C at 0 min, then 25 °C/min to 100 °C, then 35 ºC/min to 350 ºC): tR = 4.222 min. (EI, 70 eV): m/z (%): 117.0 (4), 87.0 (25), 75.1 (100), 59.1 (68), 58.1 (33)

ent-Shi catalyst **5**

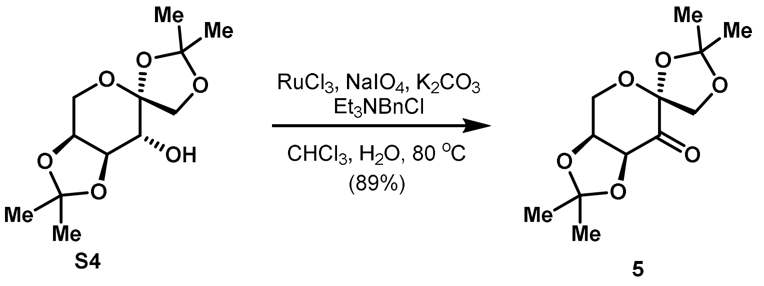

S4 was prepared according to protocols described by Shi.[68] The oxidation to afford *ent*-Shi catalyst was performed as follows: Shi-catalyst precursor **S4** (4.4 g, 16.9 mmol, 1 equiv.) was dissolved in $CHCl_3$ (15.6 mL) and H_2O (15.6 mL). To this solution was added triethyl benzyl ammonium chloride (195 mg, 0.86 mmol, 0.051 equiv.), K_2CO_3 (362 mg, 2.61 mmol, 0.155 equiv.), and ruthenium trichloride (129 mg, 0.57 mmol, 0.033 equiv.). These were then stirred vigorously and sodium periodinate (5.45 g 25.5 mmol, 1.5 equiv.) was added to the reaction mixture and the mixture was heated to 70 °C until complete consumption of the starting material was observed by TLC (typically 1 h). At this point isopropanol (3 mL) was added to the mixture and the mixture was left to stir at 22 °C for 2 hours (Note 1). The mixture was then filtered through a pad of celite. H_2O and DCM (50 mL) were then added to the reaction and the aqueous layer was separated and extracted twice more with DCM (2 x 50 mL). The combined organic layers were washed with saturated sodium thiosulfate solution (100 mL) and then brine (100 mL). The organic layer was then dried with Na_2SO_4, filtered and concentrated to yield **5** as a white solid that was confirmed by ^1H NMR to be *ent*-Shi catalyst which was used without further purification (3.9 g, 15.1 mmol, 89%) (Note 2). This procedure was taken from the literature precedent and all characterization matched.[69] The characterization obtained is listed below for convenience.

Note 1: The addition of isopropanol and stirring at 22 °C is crucial to remove the ruthenium from the reaction, without this addition the work up and extraction proves impossible.

Note 2: Occasionally the solid was contaminated with a small impurity (less than 5%, presumed mono-deketalization). This was found to not be detrimental to the subsequent Shi-epoxidation

^{1}H NMR (600 MHz, $CDCl_3$) δ 4.72 (d, J = 5.6 Hz, 1H), 4.61 (dd, J = 9.5, 0.7 Hz, 1H), 4.54 (ddd, J = 5.7, 2.2, 1.0 Hz, 1H), 4.38 (dd, J = 13.5, 2.2 Hz, 1H), 4.12 (dt, J = 13.5, 0.8 Hz, 1H), 3.99 (dd, J = 9.5, 0.7 Hz, 1H), 1.55 (s, 3H), 1.46 (s, 3H), 1.39 (s, 6H).

^{13}C NMR (151 MHz, $CDCl_3$) δ 197.08, 113.97, 110.78, 104.26, 78.06, 76.02, 70.14, 60.21, 27.29, 26.65, 26.20, 26.15.

R_f: 0.80, 50% EtOAc in hexanes (blue – anisaldehyde) (Shi catalyst **5** decomposes on silica)

$[\alpha]_D^{29.1}$ = +116.6 (c = 1.0, $CHCl_3$)

HRMS: [M+H]+ calcd. 259.1176, found 259.1177

Cycloadduct **4 and iso 4**

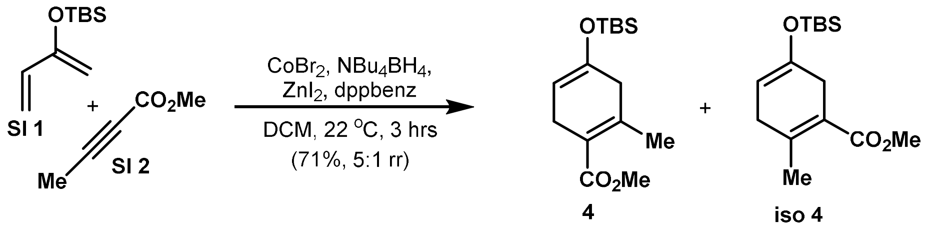

To a flame-dried 1 L RBF, 1,2-bis(diphenylphosphino)benzene (2.5 g, 5.6 mmol, 0.1 equiv.), anhydrous $CoBr_2$ (1.23 g, 5.6 mmol, 0.1 equiv.), $^{n}Bu_4NBH_4$ (4.3 g, 16.8 mmol, 0.3 equiv.) and anhydrous ZnI_2 (9.0 g, 28.0 mmol, 0.50 equiv.) were successively added. The flask was then evacuated and back filled with argon three times. Anhydrous DCM (112 mL) was added, and the reaction mixture was stirred at 22 °C for 20 min. Diene **S1** (10.9 g, 58.9 mmol, 1.05 equiv.) was subsequently added followed by alkyne **S2** (5.50 mL, 56.1 mmol, 1.00 equiv.). The reaction was stirred for 3 hrs and TLC indicated complete consumption of alkyne **S2** (Note 1). The reaction was quenched by adding Et_2O (300 mL) under rigorous stirring, resulting in a cloudy suspension. The suspension was filtered through a plug of basic alumina (approx. 300 mL, pre-packed with Et_2O) to give a dark yellow/black solution. The solution was concentrated to a neat black oil, which was subsequently loaded onto a silica gel column (approx. 400 mL, pre-packed with hexanes). Gradient elution (0% to 10% Et_2O in hexanes) afforded the product **4** as a 5:1 mixture with **iso 4** as a yellow oil (11.2 g, 39.7 mmol, 71%, 5:1 rr).

Note 1: The reaction typically has a long induction period of 1.5 hours, and an exothermic event is observed once the product starts forming; the reaction can be heated at 40 °C to shorten the induction period and similar yields are observed.

Major Regioisomer - 4:

^{1}H NMR (600 MHz, C_6D_6) δ 4.86 – 4.83 (m, 1H), 3.41 (s, 3H), 3.14 (tdt, J = 7.9, 3.7, 1.8 Hz, 2H), 2.66 – 2.61 (m, 2H), 2.00 – 1.96 (m, 3H), 0.98 (s, 9H), 0.11 (s, 6H).

^{13}C NMR (151 MHz, C_6D_6) δ 167.90, 146.53, 143.34, 122.16, 100.39, 50.79, 38.99, 29.03, 25.87, 21.25, 18.20, -4.33

R_f: 0.58 in 10% Et_2O in hexanes (UV, red/brown with anisaldehyde)

HRMS: [M+H]+ calcd. 283.1729, found 283.1730

The NMR shifts for the Minor Regioisomer (iso 4) below (R_f and mass are identical)

^{1}H NMR (600 MHz, C_6D_6) δ 4.72 – 4.67 (m, 1H), 3.38 (d, J = 0.7 Hz, 3H), 3.14 (tdt, J = 7.9, 3.7, 1.8 Hz, 2H), 2.66 – 2.61 (m, 2H), 2.04 (s, 3H), 0.97 (s, 9H), 0.13 (s, 6H).

^{13}C NMR (151 MHz, C_6D_6) δ 167.45, 148.61, 144.91, 121.35, 99.07, 50.79, 35.97, 32.58, 25.87, 21.01, 18.20, -4.33.

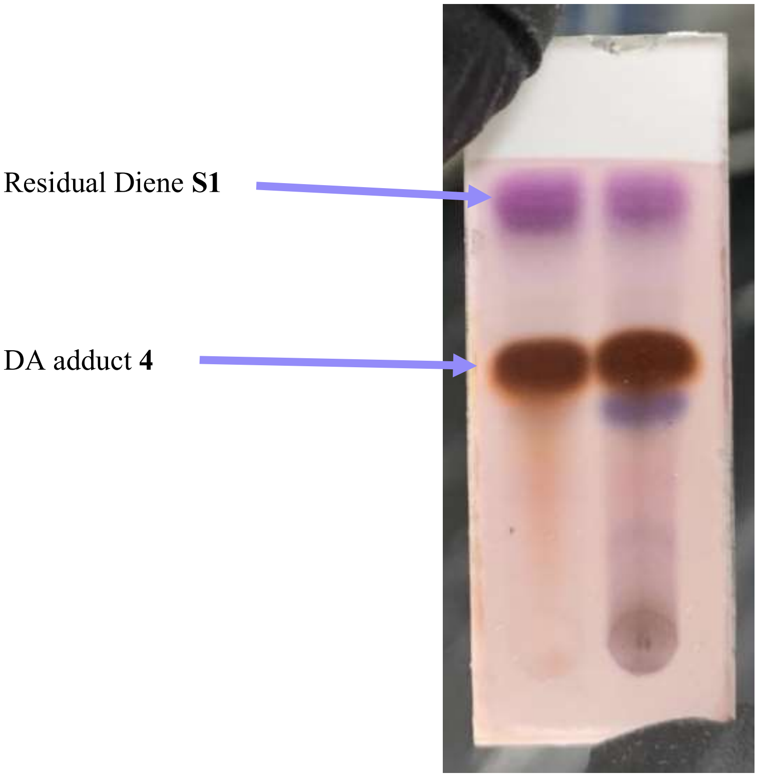

Figure S3. TLC of Diels-Alder reaction (crude - right lane) and TLC of Diels-Alder reaction after filtration through basic alumina (left lane), run in 5% Et_2O in hexanes stained with anisaldehyde. Please note that to confirm alkyne **S2** consumption, it is necessary to stain with $KMnO_4$.

Hydroxy Hagemann's ester, **6**

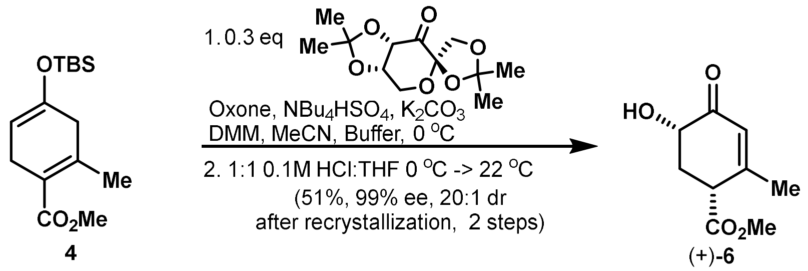

To a 5 L 3 necked RBF equipped with an overhead stirrer, cycloadduct **4** (9.0 g, 31.7 mmol, 1 equiv.) (10.8 g **4** + **Iso-4,** 38.1 mmol total), *ent*-Shi catalyst **5** (3.0 g, 12.8 mmol, 0.3 equiv.), and *tert*-butyl ammonium bisulfite (579 mg ,1.70 mmol, 0.04 equiv.) were added and dissolved in acetonitrile (200 mL), dimethoxy methane (400 mL), and Shi catalyst buffer (400 mL) (Note 1). The flask was submerged in a bath with the temperature maintained between -5 ºC and 0 ºC. Two addition funnels were fitted to the flask. To one addition funnel K_2CO_3 (53.3 g) was added in H_2O (250 mL). To the other addition funnel was added Oxone® (53.3 g) in H_2O (250 mL) with Na_2EDTA (98 mg) as buffer. The mixture was stirred vigorously (avoiding excess splashing) and the solutions of Oxone and K_2CO_3 were added at the same rate over 1.5 hours. Upon complete addition the reaction was stirred for an additional 1 hour. EtOAc and H_2O (500 mL each) were then added to quench the reaction. The organic and aqueous layers were separated and the aqueous layer was extracted two more times with EtOAc (2 x 500 ml). The organic layer was washed with brine (500 mL) and dried over Na_2SO_4. The organic layer was filtered and concentrated to a clear oil, which was directly subjected to the hydrolysis. In the same flask the oil was concentrated in, THF (150 mL) and 0.1 M HCl (150 mL) were added (Note 2) and the mixture was stirred at 22 ºC until consumption of the mass 299.2 (intermediate epoxidation mass) was observed by LCMS. This typically required 1.25-1.5 hours. H_2O and EtOAc (200 mL each) were added to quench the hydrolysis and the aqueous and organic layers were separated. The aqueous layer was extracted two more times with EtOAc (2 x 200 mL) and the organic layer was washed with brine (400 mL) and dried with Na_2SO_4. The organic layer was filtered and concentrated to a yellow oil which was stored at -20 ºC overnight. The oil was then filtered through a silica plug (approximately 200 mL) first eluting with 70% Et_2O in hexanes to remove non-polar by-products and then 80% → 90% Et_2O to elute product (collected over approximately 14-18 fractions). All fractions containing product were collected and concentrated to a crude white powder. ^{1}H NMR analysis (using trimethoxy benzene as an internal standard) indicated 23.3 mmol of product prior to recrystallization (74% yield, 5.7:1 dr, from **4**). The white powder obtained was recrystallized from boiling diisopropyl ether (Note 3) to yield **6** (2.99 g, 16.2 mmol, 51% yield from **4**) as a white solid with >99:1 er (Note 4) and > 20:1 dr determined by chiral SFC. X-ray quality crystals were grown from slow diffusion of pentane into MTBE.

^{1}H NMR (600 MHz, $CDCl_3$) δ 6.04 (s, 1H), 4.13 (dd, J = 13.4, 5.6 Hz, 1H), 3.76 (s, 3H), 3.69 (br s, 1H), 3.59 – 3.49 (m, 1H), 2.57 (dt, J = 12.5, 5.1 Hz, 1H), 2.12 (q, J = 12.6 Hz, 1H), 1.97 (s, 3H).

^{13}C NMR (151 MHz, $CDCl_3$) δ 198.31, 171.95, 158.81, 125.88, 71.11, 52.62, 47.33, 34.34, 22.15.

[α]$_D^{23.6}$ = +33.4 (c = 1.0, $CHCl_3$)

HRMS: [M+H]+ calcd.185.0814, found 185.0806

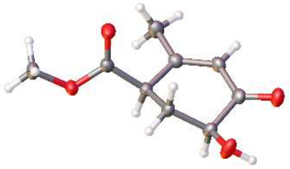

R_f: 0.43, 90% Et2O in hexanes (UV, pink – anisaldehyde)

Melting Point: 102-104 ºC

Note 1: Shi catalyst buffer is prepared by combining sodium perborate decahydrate (19.07 g), of sodium EDTA (135 mg) and H_2O (1 L).

Note 2: 0.1 M HCl was found to be the optimal acid/concentration for the hydrolysis. More concentrated HCl (i.e. 1 M) lead to significant amount of dehydration and aromatization to yield the phenol by-product. Weaker acids such as AcOH were significantly slower at the hydrolysis/isomerization procedure.

Note 3: It is important to only cool the recrystallization down to 22 ºC followed by 10 minutes in an ice bath. Cooling down the recrystallization to -20 ºC yielded samples of 83% ee. Further recrystallization of the mother liquor obtained after filtration of the crystals yields nearly racemic samples.

Note 4: It is important to prepare samples for ee measurement in non-protonated solvents, such as MeCN. Preparation of samples in MeOH lead to slow epimerization of the C4 position and slow racemization of the material. 'A_Ent1' and 'B_Ent1' (see SFC traces) were observed in samples prepared in MeOH (up to 10%) compared to samples from the same batch in MeCN (less 0.5% of 'A_Ent1' and 'B_Ent1').

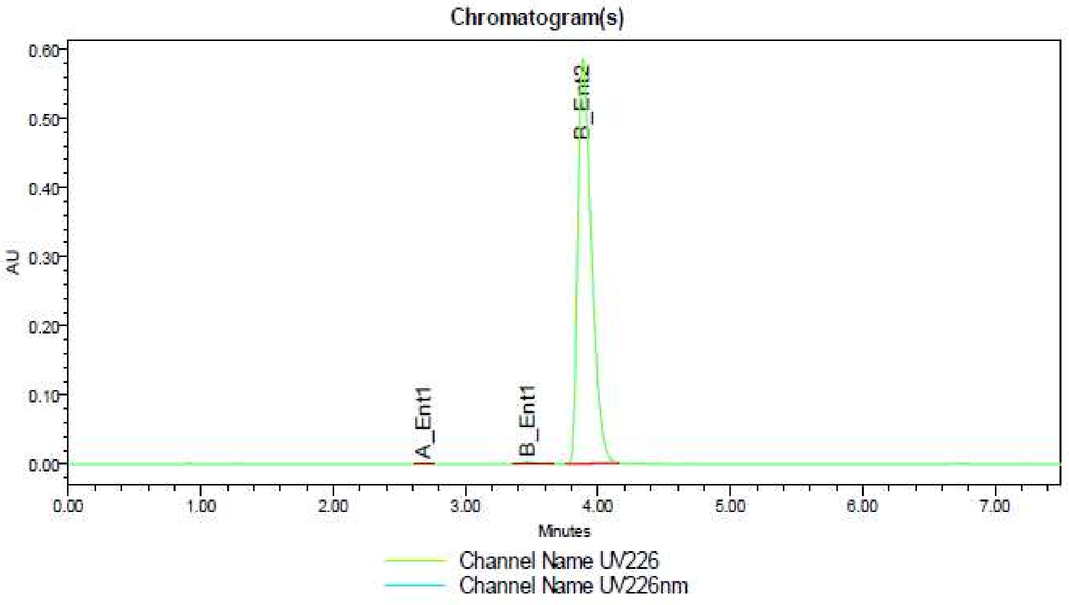

Peak Info

	Channel Name	Name	RT	Area	Height (µV)	ent1	ent2	ee
1	UV226	A_Ent1	2.69	3108	694	100.00		100.00
2	UV226		3.47	19080	2747	100.00		100.00
3	UV226		3.89	4208010	582876	100.00		100.00
4	UV226	A_Ent2	5.52			100.00		100.00
5	UV226nm		2.69	3108	694	0.45	99.55	-99.10
6	UV226nm	B_Ent1	3.47	19080	2747	0.45	99.55	-99.10
7	UV226nm	B_Ent2	3.89	4208010	582876	0.45	99.55	-99.10

Figure S4. Chiral SFC trace for (+)-**6** (B_Ent2), (B_Ent1 is (-)-**6** and A_Ent1 is trace of the *trans*-epimer)

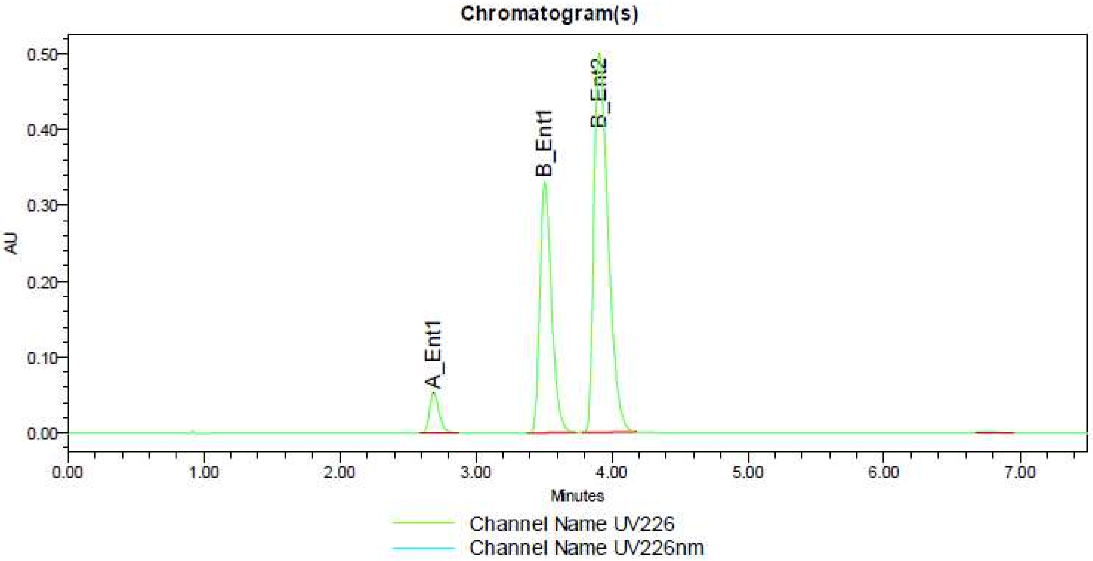

Peak Info

	Channel Name	Name	RT	Area	Height (µV)	ent1	ent2	ee
1	UV226	A_Ent1	2.69	252823	53531	100.00		100.00
2	UV226		3.51	1936543	330490	100.00		100.00
3	UV226		3.91	3569742	499526	100.00		100.00
4	UV226	A_Ent2	5.52			100.00		100.00
5	UV226		6.74	14117	1569	100.00		100.00
6	UV226nm		2.69	252823	53531	35.17	64.83	-29.66
7	UV226nm	B_Ent1	3.51	1936543	330490	35.17	64.83	-29.66
8	UV226nm	B_Ent2	3.91	3569742	499526	35.17	64.83	-29.66
9	UV226nm		6.74	14117	1569	35.17	64.83	-29.66

Figure S5. Chiral SFC trace for *rac*-**6**

Acetate (+)-**3**

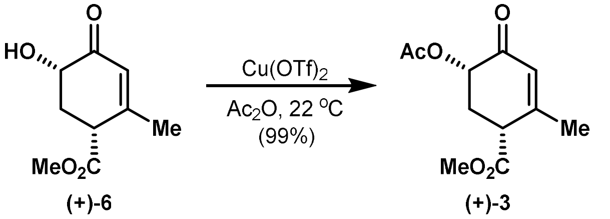

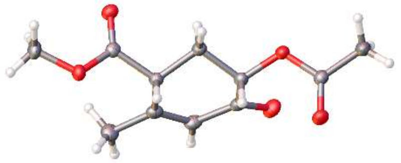

Hydroxy Hagemann's ester **6** (2.92 g, 15.9 mmol, 1 equiv.) and $Cu(OTf)_2$ (58 mg, 0.16 mmol, 0.01 equiv.) were placed in a 100 mL RBF. Acetic anhydride (30 mL) was added and the mixture was stirred at 22 °C until complete consumption of the starting material was observed by TLC (usually 10 minutes, the reaction progress can be monitored visually as the starting material was insoluble in acetic anhydride but the product completely solubilizes). 60 mL of Et_2O was then added to the reaction and this was filtered through a silica plug (60 mL of silica) and washed thoroughly with Et_2O (approximately 300 ml). The mixture was concentrated and then azeotroped 3 times with toluene (50 mL each) to remove the acetic anhydride (Note 1). Concentration on high vacuum for 12 hours yields **3** as a colorless to yellow solid (Note 2) (3.6 g, 15.9 mmol, 99%). X-ray quality crystals were grown from slow diffusion of pentane into MTBE.

^{1}H NMR (600 MHz, $CDCl_3$) δ 5.98 (dd, J = 2.6, 1.3 Hz, 1H), 5.28 (ddd, J = 12.5, 5.8, 1.2 Hz, 1H), 3.74 (d, J = 1.4 Hz, 3H), 3.57 (dddd, J = 10.7, 5.3, 2.5, 1.2 Hz, 1H), 2.44 – 2.34 (m, 2H), 2.13 (s, 3H), 1.95 (s, 3H).

^{13}C NMR (151 MHz, $CDCl_3$) δ 192.00, 171.64, 170.10, 157.00, 127.58, 71.67, 52.62, 47.00, 31.56, 22.11, 20.84.

R_f: 0.44, 80% Et_2O in hexanes (UV, Brown – $KMnO_4$)

$[\alpha]_D$ $^{23.7}$ = +67.2 (c = 1.0, $CHCl_3$)

HRMS: [M+H]+ calcd. 227.0919, found 227.0914

Melting Point: 39 – 40 °C

Note 1: If a faint blue color persists after filtration through silica and azeotroping (presumably trace $Cu(OTf)_2$), this mixture should be redissolved in Et_2O and re-filtered through silica and re-concentrated.

Note 2: If the product does not solidify under high vacuum overnight it can be placed in a -20 °C freezer overnight at which point a solid is observed.

Grignard reagent **10**

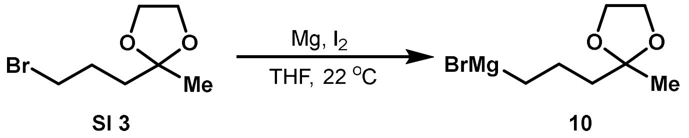

A flame-dried three-necked RBF was equipped with a reflux condenser and was evacuated and back-filled with argon three times. Magnesium turnings (5.58 g, 229.2 mmol, 1.2 equiv.), iodine (10 mg, trace), and anhydrous THF (54 mL) were combined in that flask. The iodine and magnesium turnings were stirred together until the solution turns from red to colorless (white precipitate is occasionally observed). This color change indicated activation of the magnesium. Bromide **S3** (40.0 g, 191.2 mmol, 1 equiv.) was then dissolved in anhydrous THF (54 mL) and added to the solution of magnesium turnings. The mixture was stirred at high speed and a vigorous exotherm was observed (Note 1, 2). The solution at this point turned dark brown to black and once the solution had returned to 22 °C, an aliquot was titrated with iodine (typically between 0.7-1.2 M). The solid were allowed to settle to the bottom and the Grignard reagent **10** was used as is (Note 3).

Note 1: Caution: The exotherm on larger scales requires a reflux condenser for safety

Note 2: If the bromide and magnesium have been stirred together for some time without an exotherm observed, the solution can be gently heated to initiate Grignard reagent formation or 0.05 equiv. of dibromoethane added.

Note 3: The Grignard reagent should be used immediately for best results in the silyl enol ether **7** synthesis. Storing **10** overnight showed decreased yields in the synthesis of **7**

Silyl enol ether 7

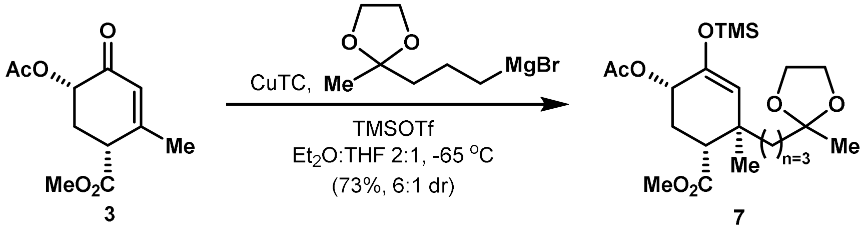

A 500 mL, 3-necked RBF was equipped with an overhead stirrer and flame-dried under vacuum. To this RBF, CuTC (9.10 g, 47.7 mmol, 3 equiv.) was added under a positive flow of N_2. The flask was then evacuated and back-filled with argon three times, followed by the addition of Et_2O (60 mL). The suspension was cooled to -60 °C and vigorous stirring was applied. At this temperature, the Grignard reagent (60 mL of 0.80 M solution in THF, 48.0 mmol, 3 equiv.) was added slowly. Upon completion, the suspension was allowed to stir for 20 minutes within the temperature range of –60 °C to –50 °C. In the meantime, a solution of the acetate **3** (3.6 g, 15.9 mmol, 1 equiv.) in a mixture of Et_2O/THF (60 mL/8.6 mL, respectively) was prepared (Note 1). The reaction flask was cooled to –65 °C. TMSOTf (5.8 mL, 31.8 mmol, 2 equiv.) and the solution of **3** were added slowly, respectively. The reaction suspension was stirred vigorously at below -60 °C for 25 mins (Note 2). To the suspension, H_2O (180 mL), Et_2O (60 mL), saturated aqueous EDTA solution (40 mL) and saturated aqueous ammonium chloride (40 mL) were then added in that order (Note 3). The organic layer was separated, and the aqueous layer was extracted twice with Et_2O (100 mL each). The combined organic layers were washed with brine (250 mL), dried over Na_2SO_4 and concentrated to a crude mixture. The mixture was purified by silica gel column chromatography (approximately 800 mL SiO_2) (Note 4). Gradient elution (5% → 10% → 15% acetone in hexanes) afforded the desired product **7** (4.93 g, 11.5 mmol, 73% yield) as a clear light yellow oil. (Note 5)

Note 1: Acetate **3** is sparingly soluble in Et_2O. To ensure all is transferred from the vessel, THF can be used to rinse the glassware. Only up to 1/7th volume of THF:Et_2O can be used to rinse otherwise diastereoselectivity of the reaction drops.
Note 2: Carefully monitoring of the reaction temperature is important as temperatures above -60 °C begin to decompose the product, likely from the excess organometallics.
Note 3: It is critical that the aqueous quench is added first before diluting with Et_2O, otherwise the temperature will be rapidly raised and the product decomposed.
Note 4: Silica gel flash columns performed on pre-packed columns for use on an automated column failed to separate the thiophene carboxylic acid from the product. It is best to thoroughly pack the silica by hand.
Note 5: Fractions were occasionally contaminated with Würtz dimer from the Grignard reagent synthesis. These fractions were collected as it did not affect the subsequent iodination and could be readily removed upon the subsequent purification of the iodide **7**.

^{1}H NMR (600 MHz, C_6D_6) δ 5.51 (ddd, J = 10.3, 6.6, 1.3 Hz, 1H), 4.72 (d, J = 1.3 Hz, 1H), 3.67 – 3.49 (m, 4H), 3.28 (s, 3H), 2.50 (dd, J = 13.5, 2.9 Hz, 1H), 2.24 (td, J = 13.2, 10.2 Hz, 1H), 2.10 (ddd, J = 13.0, 6.6, 2.9 Hz, 1H), 1.77 (s, 3H), 1.77 – 1.65 (m, 4H), 1.49 – 1.39 (m, 1H), 1.36 (s, 3H), 1.24 – 1.15 (m, 1H), 1.04 (s, 3H).

^{13}C NMR (151 MHz, C_6D_6) δ 172.76, 169.96, 147.94, 117.57, 110.13, 68.96, 64.68 (2C), 50.88, 43.76, 42.16, 40.28, 38.50, 28.76, 25.03, 24.08, 20.80, 19.38, 0.28.

R_f: 0.43, 20 % acetone in hexanes (brown – anisaldehyde)

$[\alpha]_D^{25.8}$ = +2.5 (c = 1.0, EtOAc)

HRMS: [M+Na]+ calcd. 451.2128, found 451.2125

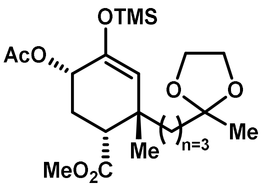

epi-**7**

Epi-**7** Characterization

^{1}H NMR (600 MHz, C_6D_6) δ 5.46 (t, *J* = 1.8 Hz, 1H), 5.05 (dd, *J* = 11.0, 3.2 Hz, 1H), 3.62 – 3.51 (m, 4H), 3.26 (s, 3H), 2.90 – 2.84 (m, 1H), 2.59 (ddd, *J* = 12.2, 10.9, 10.0 Hz, 1H), 1.96 (ddd, *J* = 12.3, 6.3, 3.2 Hz, 1H), 1.84 – 1.78 (m, 1H), 1.76 (s, 3H), 1.76-1.57 (m, 5H) 1.56 (t, *J* = 1.2 Hz, 4H), 1.34 (s, 3H), 0.28 (s, 9H).

^{13}C NMR (151 MHz, C_6D_6) δ 172.77, 170.10, 134.62, 130.29, 110.07, 74.00, 72.41, 64.71, 64.69, 51.26, 46.17, 40.78, 40.21, 27.85, 24.09, 21.42, 20.95, 19.07, 2.99.

2D NMR: HSQC, NOESY

R_f : 0.52, 10% Et_2O in DCM (brown – anisaldehyde)

$[\alpha]_D^{25.0}$ = +43.2 (c = 0.5, EtOAc)

HRMS: [M+Na]+ calcd. 451.2128, found 451.2139

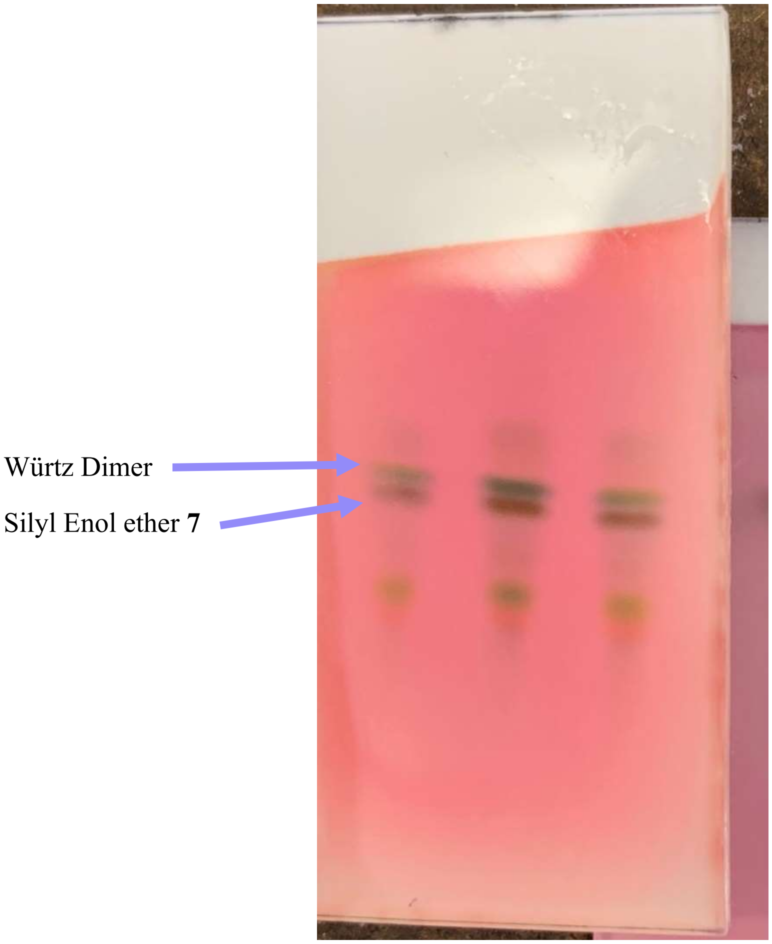

Figure S6. Crude TLC of conjugate addition (in triplicate), Würtz dimer from Grignard reagent **10** (stained green), and desired silyl enol ether **7** below (stained brown). TLC plate was run with 30% acetone in hexanes and stained with anisaldehyde.

Iodide **11**

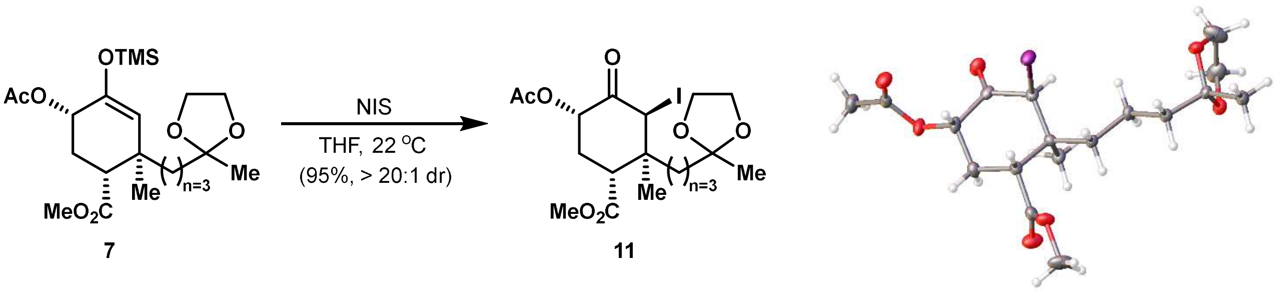

In a 100 mL RBF equipped with a stir bar, silyl enol ether **7** (1.06g, 2.48 mmol, 1 equiv.) was dissolved in 15 mL of anhydrous THF and the RBF was wrapped in aluminum foil and fumehood lights turned off. NIS (236 mg ,1.05 mmol, 0.4 equiv.) was added directly to the solution every 30 mins until complete consumption of the starting material **7** was observed by TLC (this typically required 6 to 7 additions of NIS, 2.4-2.8 equiv. total). Once complete, Et_2O (30 mL) and saturated aqueous sodium thiosulfate solution (30 mL) were added to the mixture. The mixture was shaken in a separatory funnel until the solution turned clear (initially dark brown). The layers were separated and the aqueous layer was extracted twice more with EtOAc (2 x 50 mL) and washed again with sodium thiosulfate solution if any discoloration persisted. The organic layer was then washed with brine (50 mL), dried with Na_2SO_4, filtered and concentrated to a yellow oil. The crude was purified by flash column chromatography on silica eluting with a gradient of 30 →70% EtOAc in hexanes. The purified material was concentrated to yield **11** as a white solid (1.14 g, 2.36 mmol, 95% yield). This material could be recrystallized to afford X-ray quality crystals by dissolving in minimum amount of Et_2O and leaving in a -20 °C freezer for 12 hours. This recrystallization technique is also suitable on large scale to avoid column chromatography.

^{1}H NMR (600 MHz, $CDCl_3$) δ 6.37 (dd, J = 11.0, 9.3 Hz, 1H), 4.31 (s, 1H), 4.01 – 3.90 (m, 4H), 3.72 (s, 3H) 3.25 – 3.19 (m, 1H), 2.29 – 2.23 (m, 2H), 2.19 (s, 3H), 1.75 – 1.54 (m, 3H), 1.45 (td, J = 13.0, 4.6 Hz, 1H), 1.37-1.24 (m, 2H) 1.33 (s, 3H), 1.27 (s, 3H).

^{13}C NMR (151 MHz, $CDCl_3$) δ 198.11, 172.52, 169.71, 109.64, 69.75, 64.70, 64.66, 51.95, 46.88, 41.46, 41.15, 40.89, 39.17, 30.38, 23.89, 20.60, 16.90, 14.90.

R_f: 0.66, 60% EtOAc in Hexanes (UV, brown - anisaldehyde)

$[\alpha]_D^{22.9}$ = +76.0 (c = 1.0, $CHCl_3$)

HRMS: [M+H]+ calcd. 483.0880, found 483.0875

Melting Point: 119-120 °C

SmI_2 Reformatsky adduct **14**

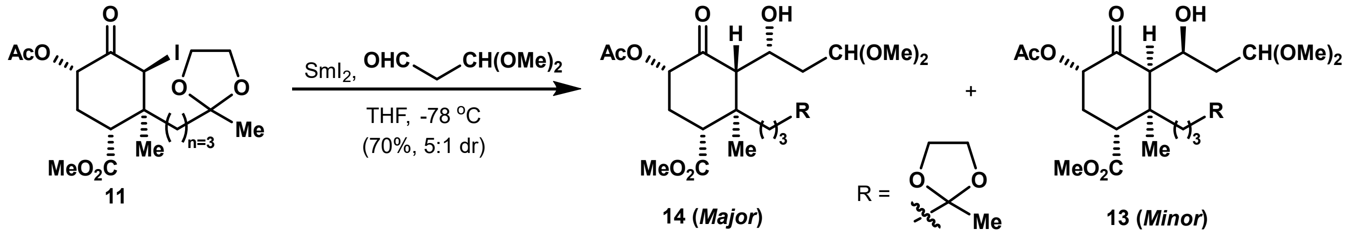

In a flame dried RBF under argon, iodide **7** (200 mg, 0.41 mmol, 1 equiv.) was dissolved in THF (50 mL) and the solution was cooled to -78 °C (Note 1). 0.1 M SmI_2 solution in THF was added dropwise to the solution. Approximately 2.1 equiv. was required based on the titre of the SmI_2 solution but it was added until a light green persisted in the solution and TLC analysis indicated complete consumption of the iodide **7** (Note 2). Neat aldehyde **12** was then added (200 µL, 1.7 mmol, 4.1 equiv.) and the mixture was allowed to stir for 20 minutes (Note 3). Acetic acid (200 µL) was then added at -78 °C followed immediately by saturated aqueous ammonium chloride solution (50 mL) and EtOAc (50 mL). The reaction mixture was brought to 22 °C and the layers were separated. The aqueous layer was extracted two more times with EtOAc (2 x 50 mL). The organic layer was then washed twice with saturated aqueous sodium thiosulfate solution (2 x 100 mL) and the brine (50 mL). The organic layer was then dried with Na_2SO_4, filtered, and concentrated under reduced pressure to yield a crude oil. This was then purified by column chromatography with an elution gradient of 40 →70% EtOAc in hexanes. The fractions containing the product were combined and concentrated to yield a mixture 5:1 of diastereomers **14** and **13** as a colorless oil (Note 4) (137 mg, 0.29 mmol, 70%, 5:1 dr).

Characterization of 14 (see below for characterization of **13**)

^{1}H NMR (600 MHz, C_6D_6) δ 4.75 (dd, J = 12.8, 6.9 Hz, 1H), 4.55 (tt, J = 9.2, 2.1 Hz, 1H), 4.40 (dd, J = 6.1, 4.4 Hz, 1H), 3.67 – 3.56 (m, 4H), 3.29 (s, 3H), 3.24 (d, J = 3.4 Hz, 1H), 3.04 (s, 3H), 3.03 (s, 3H), 2.91 (dd, J = 13.4, 3.6 Hz, 1H), 2.68 (d, J = 9.2 Hz, 1H), 2.45 – 2.35 (m, 1H), 2.30 (q, J = 13.0 Hz, 1H), 2.04 (ddd, J = 14.2, 4.4, 1.7 Hz, 1H), 1.96 – 1.79 (m, 5H), 1.78 (s, 3H), 1.77 – 1.69 (m, 1H), 1.49 (s, 3H), 1.31 – 1.23 (m, 1H), 1.16 (s, 3H).

^{13}C NMR (151 MHz, C_6D_6) δ 205.01, 172.20, 169.37, 110.38, 105.15, 75.94, 66.76, 64.60, 64.58, 58.47, 54.01, 53.00, 51.12, 47.67, 46.40, 40.38, 39.88, 39.35, 31.49, 23.93, 20.25, 19.04, 18.16.

R_f: 0.31, 70% EtOAc in Hexanes (orange – anisaldehyde)

$[\alpha]_D^{24.3}$ = -72.4 (c = 1.0, EtOAc)

HRMS: [M+ Na]+ calcd. 497.2363, found 497.2364

Note 1: The reaction dr appeared to heavily depend on running at a dilute concentration, scales larger than 200 mg seemed to diminish dr. On a 400 mg scale a 1.5:1 dr was observed. This reaction could be scaled-out in multiple runs at once to aid material throughput (4 flasks, 800 mg).
Note 2: The titre between various SmI_2 bottles varied so much; it was easier to add until the color change was apparent and monitor by TLC for complete consumption of iodide **11**.
Note 3: TLC analysis of the reaction at this point was difficult as rapid retro-aldol would occur upon warming to 22 °C.

Note 4: Exhaustive screening of solvent systems to separate diastereomers **13** and **14** proved unproductive, even several three-component solvent systems were unable to separate these diastereomers. Selective crystallization also proved unsuccessful.

Ferrocene carboxylate **S5**

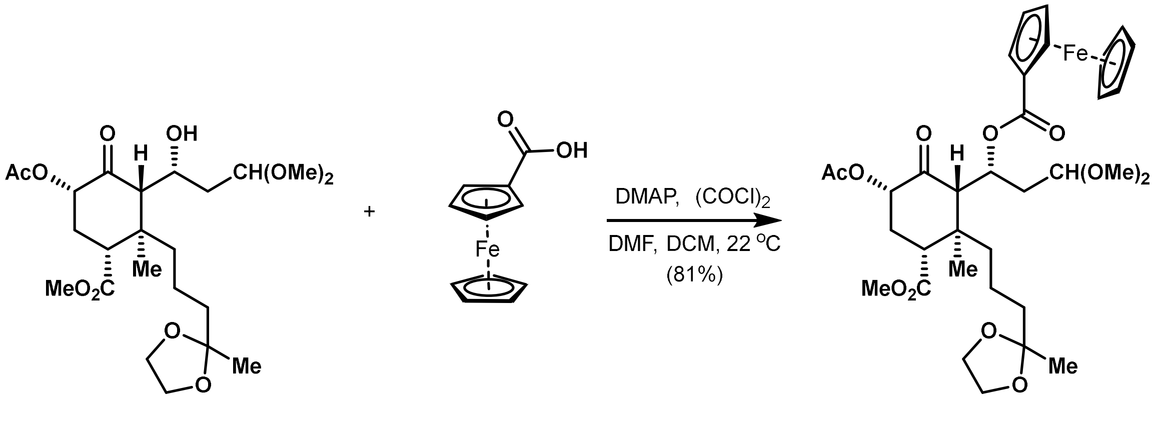

14 **S5**

Following the procedure from Magauer et al.[70]: To a flame dried 8 mL reaction tube under argon was added ferrocene carboxylic acid (12 mg, 0.052 mmol, 2 equiv.) and DCM (1 mL). 2 M Oxalyl chloride in DCM (30 μL, 0.06 mmol, 2.3 equiv.) was added followed by 1 drop of DMF. This solution was stirred for 45 minutes at 22 °C at which point toluene (1 mL) was added and the reaction was concentrated under a stream of nitrogen. To a separate 8 mL reaction tube a 5:1 mixture of **14**:**13** (12 mg, 0.0252 mmol, 1 equiv.) and DMAP (32 mg, 0.26 mmol, 10.4 equiv.) was added and these were dissolved in DCM (0.5 mL). The freshly prepared ferrocene acid chloride was then dissolved in DCM (0.5 mL) and added to the solution of **14**. This was stirred at 22 °C for 2 hours then concentrated under a stream of nitrogen and loaded directly onto a preparatory TLC plate eluting with 60% EtOAc in hexanes. The band containing **S5** was collected and concentrated to an orange solid (14 mg, 81% yield). X-ray quality crystals were grown by dissolving the product in a 3:1 mixture of MTBE:DCM and then diffusing pentane into the solution.

^{1}H NMR (600 MHz, C_6D_6) δ 6.02 (ddd, J = 8.2, 6.4, 2.7 Hz, 1H), 4.96 (ddt, J = 5.8, 2.6, 1.3 Hz, 2H), 4.88 – 4.82 (m, 1H), 4.79 (dd, J = 6.9, 4.3 Hz, 1H), 4.16 (s, 5H), 4.07 (td, J = 2.5, 1.4 Hz, 1H), 4.04 (ddd, J = 3.6, 2.5, 1.2 Hz, 1H), 3.80 – 3.70 (m, 2H), 3.70 – 3.63 (m, 2H), 3.25 (s, 3H), 3.24 (s, 3H), 3.22 (s, 3H), 3.07 (d, J = 6.4 Hz, 1H), 2.88 (ddd, J = 15.0, 8.1, 4.3 Hz, 1H), 2.82 (dd, J = 13.4, 3.6 Hz, 1H), 2.32 – 2.22 (m, 2H), 2.03 – 1.81 (m, 4H), 1.80 (s, 3H), 1.73 (dddd, J = 21.7, 13.4, 11.1, 5.4 Hz, 2H), 1.58 (s, 3H), 1.28 (ddd, J = 14.6, 11.8, 4.5 Hz, 1H), 1.11 (s, 3H).

^{13}C NMR (151 MHz, C_6D_6) δ 202.47, 171.78, 170.70, 169.24, 110.28, 102.52, 75.44, 72.26, 71.66, 71.46, 70.84, 70.70, 67.69, 64.75, 64.71, 56.74, 52.74, 52.68, 51.15, 47.69, 45.18, 39.95, 39.57, 36.86, 31.08, 24.27, 20.27, 18.94, 18.29.

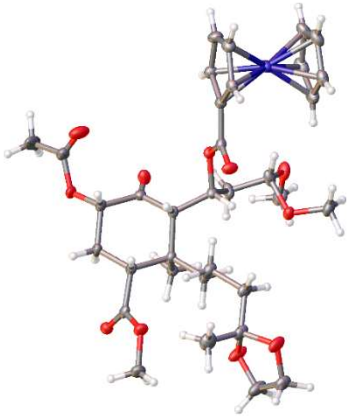

R_f: 0.44, 60% EtOAc in Hexanes (UV, green - anisaldehyde)

$[\alpha]_D^{27.4}$ = -33.1 (c = 1.0, EtOAc)

HRMS: [M-e⁻] calcd. 686.2390, found 686.2408 (presumably from oxidation to Fe^{3+})

Melting Point: 146-150 °C

Et_2Zn Reformatsky adduct **13**

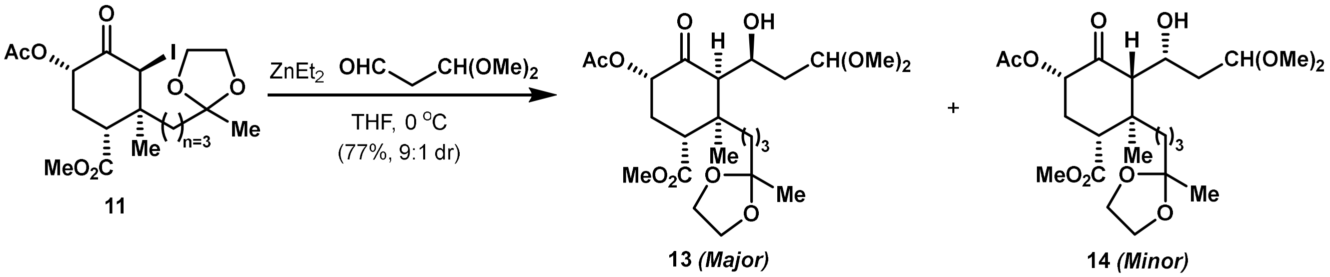

In a flame-dried 100 mL RBF, iodide **7** (100 mg, 0.21 mmol, 1 equiv.) was dissolved in anhydrous THF (25 mL) under an argon atmosphere. The solution was cooled to 0 °C, and Et_2Zn (0.21 mL of 1 M solution in THF, 0.21 mmol, 1 equiv.) was then added drop-wise (Note 1). The reaction was stirred for 5 mins before aldehyde **12** (0.2 mL, 8 equiv.) was added. Upon complete conversion of the enolate to the product, as indicated by TLC after 1 hour typically, AcOH (0.1 mL) and H_2O (25 mL) were added at 0 °C. The mixture was warmed up to 22 °C and the aqueous layer was extracted with EtOAc (3.25 mL). The combined organic layers were then washed with brine, dried over Na_2SO_4 and concentrated to afford a yellow crude oil. Column chromatography with silica gel (approximately 60 mL) on this crude material under gradient elution (40% → 50% → 60% → 70% → 80% of EtOAc in hexanes) afforded the desired products as a mixture of two diastereomers (75 mg total, 9:1 dr, 77% yield). The major diastereomer could be recrystallized to afford X-ray quality crystals by vapor diffusion of MTBE/$CHCl_3$ with pentane.

*Characterization of **13***

^{1}H NMR (500 MHz, $CDCl_3$) δ 5.03 (dd, J = 12.3, 7.9 Hz, 1H), 4.54 (t, J = 4.7 Hz, 1H), 4.35 (t, J = 9.3 Hz, 1H), 4.11 – 3.78 (m, 4H), 3.70 (s, 3H), 3.57 (s, 1H), 3.39 (s, 3H), 3.38 (s, 3H), 3.26 (dd, J = 13.0, 4.3 Hz, 1H), 2.56 (d, J = 9.2 Hz, 1H), 2.33 – 2.16 (m, 2H), 2.15 (s, 3H), 2.05 – 1.92 (m, 1H), 1.86 (ddd, J = 14.7, 4.4, 1.8 Hz, 1H), 1.73 (ddd, J = 14.5, 9.3, 5.0 Hz, 1H), 1.67 – 1.47 (m, 3H), 1.28 (s, 3H), 1.27 – 1.20 (m, 1H), 1.15 (td, J = 11.8, 4.5 Hz, 1H), 0.97 (s, 3H).

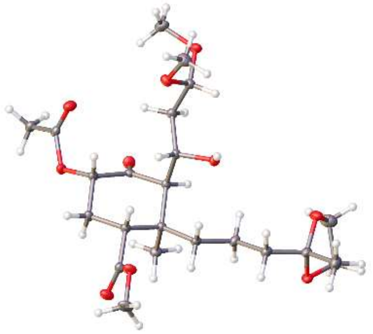

^{13}C NMR (151 MHz, $CDCl_3$) δ 203.72, 173.35, 170.24, 110.21, 104.77, 74.42, 65.87, 65.14, 64.75, 64.66, 54.99, 54.35, 51.87, 46.28, 43.04, 39.49, 38.74, 37.46, 30.62, 23.87, 20.73, 20.02, 18.45.

R$_f$: 0.31, 70% EtOAc in Hexanes (orange – anisaldehyde)

$[\alpha]_D^{27.4}$ = –14.3 (c = 1.0, $CHCl_3$)

HRMS: [M+ Na]+ calcd. 497.2363, found 497.2372

Note 1: TLC should indicate complete consumption of the starting material and the formation of the enolate

Samarium Reformatsky

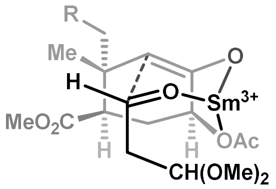

to **14**

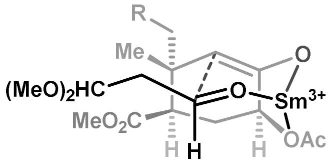

to 10-*epi*-**14**

In the case of the SmI_2 Reformatsky, we hypothesize the stereoselectivity is derived from a Zimmerman-Traxler type transition state with the aldehyde delivered from the same face as the acetate via samarium coordination to the acetate. The stereochemistry at the alcohol derives from the preference of the aldehyde side chain to sit in the axial position of the chair to avoid steric interactions with the beta substituents.

Zinc Reformatsky

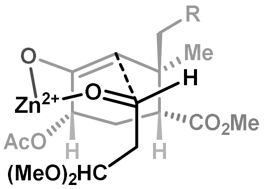

to **13**

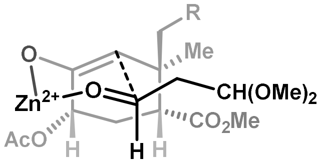

to 10-*epi*-**13**

In the case of the Et_2Zn Reformatsky, we hypothesize the stereoselectivity is derived from a Zimmerman-Traxler type transition state with the aldehyde being delivered from the opposite face since the zinc may have fewer coordination sites than samarium. The aldehyde side chain again prefers to be in the axial position to avoid the unfavorable 1,3-diaxial type interaction with the large beta substituents.

R =

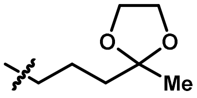

Figure S7. Discussion of Reformatsky stereoselectivity

Enone **17a, b, c, d**

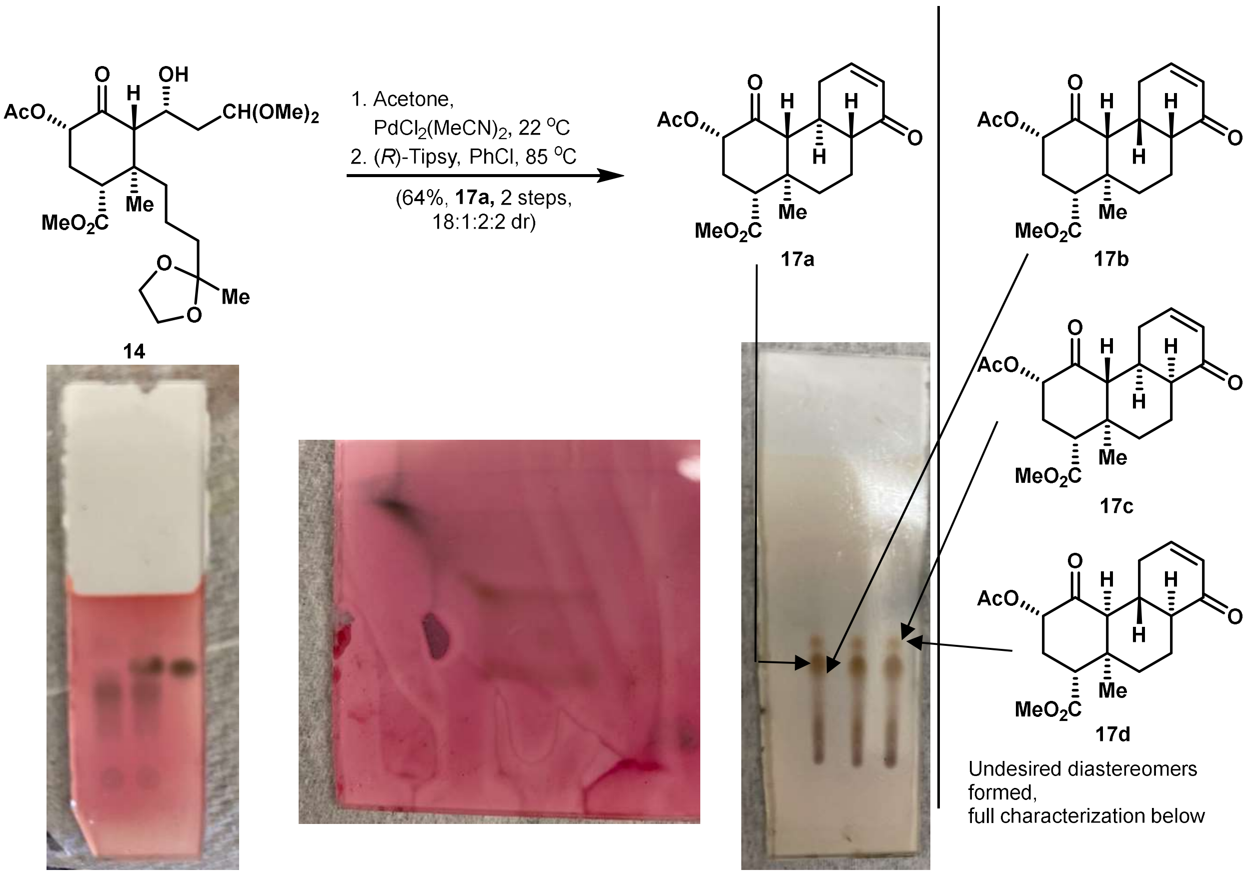

1D TLC of deprotection | 2D TLC of deprotection | Robinson annulation TLC (24 h)

Figure S8. TLC analysis of deprotection and Robinson annulation protocol
1D (left) and 2D TLC (middle) of $PdCl_2(MeCN)_2$ in acetone deprotection (50% acetone in hexanes), 1D shows presumed mono deprotection left on top of bis deprotection (lower spot that streaks), against starting material **14** on the right lane. The reaction should be run until only bis-deprotection is observed. 2D TLC (middle) shows decomposition and interconversion of intermediate aldehyde **15a** by cross peak in the 2D TLC. 1D TLC of completion of cyclization after 24 hour reaction (right (60% EtOAc in hexanes). Top orange/pink spot contains undesired isomers **17c** and **17d**. Lower orange spot is desired isomer **17a** and undesired isomer **17b** overlapping slightly, which can separated by recrystallization or chromatography. All enones are UV active.

Procedure

Reformatsky adducts **14/13** (215 mg 5:1 mixture of diastereomers, 179 mg of **14**, 0.453 mmol, 1 equiv.) were dissolved in acetone (40 mL). $PdCl_2(MeCN)_2$ (9 mg, 0.347 mmol, 0.07 equiv.) was then added and the reaction mixture stirred at 22 °C until complete consumption of starting material was observed and complete disappearance of a another less polar product on TLC (presumably mono-deprotection of the starting material, see Figure **S6**, usually 1.5 hours). The solution was then diluted with Et_2O (40 mL) and decolorizing carbon (720 mg, 1.6 g/mmol) was then added to the reaction mixture. The vial was shaken to

mix the carbon well. This solution was then filtered through a small pad of celite to completely remove decolorizing carbon and the flask and carbon rinsed several times with a mixture of acetone and Et_2O. The solution is then concentrated (Note 1), and the aldehyde **15a** transferred directly to the vessel with DCM (approximately 10 mL) (oven dried and equipped with a stir bar) for the Robinson annulation (Note 2). On this scale a 50 mL pressure tube with a small diameter (Note 3) was used. The DCM used to transfer was then concentrated by a constant flow of nitrogen and degassed chlorobenzene (4.5 mL) is then added (Note 4) followed by 115 mg of (*R*)-Tipsy catalyst (0.133 mmol, 0.3 equiv.). The reaction is then heated to 85 ºC for 24 hrs. After 24 hours of heating, the vessel is removed from the oil bath and the chlorobenzene is concentrated inside a fumehood via a constant flow of nitrogen gas. ^{1}H NMR analysis indicated 70% total yield over two steps (55% **17a**, 3% **17b**, 6% **17c**, 6% **17d**) using dibromomethane as an internal standard. The crude is then directly loaded onto a silica gel column eluting with 30 → 35 → 40 - > 50 → 60 → 70% EtOAc in hexanes. The fractions containing the desired diastereomer (see above) are combined and concentrated to yield enone **17a** (Note 5) as a white solid (84 mg, 64% over two steps, based on 179 mg of **14** that can lead to product).

Note 1: The concentration of the first reaction should be monitored carefully, if any color persists or is visible at any point during concentration more decolorizing carbon should be added and the reaction filtered again. We found decolorizing carbon to be the most effective way to remove palladium and prevent decomposition of the aldehyde intermediate which is highly unstable. Filtration through silica failed to remove palladium and the aldehyde intermediate clearly decomposed on silica upon analysis by 2D TLC (above)
Note 2: As stated above the aldehyde intermediate is highly unstable and should be used immediately.
Note 3: Liquid can be seen condensing at the top on the sides of the glass vessel (presumably an azeotrope of H_2O and chlorobenzene). For this reason vials that were long and had a narrow diameter were used. Addition of molecular sieve to remove the H_2O seemed to be detrimental to the reactivity
Note 4: Chlorobenzene was degassed by bubbling argon through for 45 minutes prior to the reaction.
Note 5: If diastereomer **17b** contaminates **17a** after column chromatography it can be removed by recrystallization from minimal amount of Et_2O. Filtration and concentration of the mother liquor then yields pure **17a**. **17a** and **17b** also separate in DCM/Et_2O eluents.

Deprotected aldehydes **15a** and **15b**

Due to aldehyde instability on silica gel, and slow decomposition over time, optical rotations were not obtained.

Aldehyde 15a

^{1}H NMR (600 MHz, C_6D_6) δ 9.13 (t, *J* = 0.8 Hz, 1H), 4.71 (dd, *J* = 12.8, 6.9 Hz, 1H), 4.61 (d, *J* = 9.9 Hz, 1H), 3.34 (s, 3H), 2.93 (d, *J* = 5.4 Hz, 1H), 2.81 (dd, *J* = 13.4, 3.7 Hz, 1H), 2.58 (dd, *J* = 18.2, 2.3 Hz, 1H), 2.52 (d, *J* = 9.3 Hz, 1H), 2.22 (q, *J* = 13.0 Hz, 1H), 2.11 – 2.01 (m, 3H), 2.01 – 1.95 (m, 1H), 1.92 (ddd, *J* = 13.0, 7.0, 3.7 Hz, 1H), 1.80 (s, 3H), 1.79 – 1.76 (m, 1H), 1.69 (s, 3H), 1.17 – 1.09 (m, 1H), 0.99 (s, 3H).

^{13}C NMR (151 MHz, C_6D_6) δ 207.18, 204.54, 202.33, 171.95, 169.48, 75.84, 64.92, 57.59, 51.32, 50.59, 47.33, 46.20, 43.59, 39.01, 31.36, 29.43, 20.21, 18.20, 17.87.

R_f = 0.80, 50% acetone in hexanes (brown – anisaldehyde)

LRMS: [M+Na]+ calcd. 407.2, found 407.2

Aldehyde 15b

^{1}H NMR (600 MHz, C_6D_6) δ 9.18 (s, 1H), 4.44 (dd, *J* = 12.6, 7.5 Hz, 1H), 4.32 – 4.25 (m, 1H), 3.35 (s, 3H), 2.82 (dd, *J* = 13.3, 3.8 Hz, 1H), 2.67 (dd, *J* = 18.5, 2.1 Hz, 1H), 2.58 (d, *J* = 9.6 Hz, 1H), 2.28 – 2.22 (m, 2H), 1.95 – 1.78 (m, 4H), 1.78 (s, 3H), 1.69 – 1.62 (m, 1H), 1.61 (s, 3H), 1.27 (qt, *J* = 13.2, 5.6 Hz, 1H), 1.15 (td, *J* = 12.8, 5.1 Hz, 1H), 0.97 (s, 3H).

^{13}C NMR (151 MHz, C_6D_6) δ 207.13, 202.12, 201.93, 172.65, 169.80, 74.66, 64.55, 64.47, 51.27, 48.72, 46.45, 43.61, 42.72, 38.10, 30.53, 29.25, 20.20, 19.93, 18.29.

R_f = 0.80, 50% acetone in hexanes (brown – anisaldehyde)

LRMS: [M+Na]+ calcd. 407.2, found 407.2

Enone 17a

^{1}H NMR (600 MHz, $CDCl_3$) δ 6.90 (ddd, *J* = 10.0, 6.2, 2.1 Hz, 1H), 6.01 (dd, *J* = 10.1, 2.9 Hz, 1H), 5.17 (dd, *J* = 12.3, 7.6 Hz, 1H), 3.72 (s, 3H), 2.79 (dd, *J* = 13.1, 4.3 Hz, 1H), 2.75 (ddd, *J* = 3.30, 6.45, 18.42, 1H) 2.41 – 2.26 (m, 4H), 2.17 (s, 3H), 2.12 (dq, *J* = 14.2, 3.5 Hz, 1H), 2.02 (td, *J* = 12.2, 4.1 Hz, 1H), 1.84 (ddt, *J* = 18.5, 9.6, 2.6 Hz, 1H), 1.78 (dt, *J* = 13.2, 3.4 Hz, 1H), 1.52 (dd, *J* = 13.5, 3.8 Hz, 1H), 1.44 – 1.34 (m, 1H), 0.86 (s, 3H).

^{13}C NMR (151 MHz, $CDCl_3$) δ 202.48, 199.88, 171.86, 170.17, 149.25, 129.25, 75.32, 60.71, 52.29, 52.04, 49.56, 42.68, 37.65, 33.76, 31.63, 31.31, 20.77, 20.01, 13.70.

R_f: 0.48, 60% EtOAc in hexanes (UV, light brown – anisaldehyde)

$[\alpha]_D^{23.0}$ = -17.9 (c =0.1, $CHCl_3$)

HRMS: [M+H]+ calcd. 349.1651, found 349.1649

Melting Point: 68 – 70 °C

Enone 17b

^{1}H NMR (600 MHz, $CDCl_3$) δ 6.98 (ddd, *J* = 10.1, 6.2, 2.2 Hz, 1H), 6.00 (dd, *J* = 10.1, 2.7 Hz, 1H), 5.21 (ddd, *J* = 10.9, 8.5, 1.0 Hz, 1H), 3.72 (s, 3H), 3.25 (dt, *J* = 19.7, 5.8, 1H), 2.83 – 2.73 (m, 1H), 2.70 (dt, *J* = 12.0, 4.5 Hz, 1H), 2.52 (ddt, *J* = 19.5, 12.0, 2.6 Hz, 1H), 2.45 – 2.26 (m, 3H), 2.23 (dt, *J* = 11.8, 4.4 Hz, 1H), 2.17 (s, 3H), 1.83 – 1.74 (m, 1H), 1.74 – 1.59 (m, 3H), 1.14 (s, 3H).

^{13}C NMR (151 MHz, $CDCl_3$) δ 203.26, 201.61, 171.88, 170.04, 151.00, 128.15, 74.65, 57.45, 53.38, 52.05, 49.53, 41.41, 38.40, 33.52, 30.67, 25.21, 20.96, 20.76, 17.02.

$[\alpha]_D^{23.4}$ = -84.0 (c = 0.1, $CHCl_3$)

R_f: 0.41, 15% Et2O in DCM (UV, orange – anisaldehyde)

HRMS: [M+H]+ calcd. 349.1651, found 349.1649

Enone 17c

^{1}H NMR (600 MHz, $CDCl_3$) δ 6.81 – 6.75 (m, 1H), 6.03 (dd, *J* = 10.1, 3.0 Hz, 1H), 5.07 (dd, *J* = 11.8, 8.1 Hz, 1H), 3.67 (s, 3H), 2.78 (dt, *J* = 11.5, 5.5 Hz, 1H), 2.71 (dd, *J* = 12.9, 4.5 Hz, 1H), 2.68 – 2.57 (m, 2H), 2.51 (d, *J* = 11.8 Hz, 1H), 2.49 – 2.40 (m, 2H), 2.37 – 2.20 (m, 2H), 2.16 (s, 3H), 1.52 – 1.39 (m, 3H), 0.89 (s, 3H).

^{13}**C NMR** (151 MHz, $CDCl_3$) δ 203.39, 199.29, 172.43, 170.14, 148.28, 129.39, 75.33, 54.04, 52.10, 51.87, 45.69, 43.00, 34.56, 31.69, 30.90, 28.59, 20.78, 20.03, 13.53.

$[\alpha]_D^{23.4}$ = -5.7 (c = 0.1, $CHCl_3$)

R_f: 0.56, 60% EtOAc in hexanes (UV, pinkish brown – anisaldehyde)

HRMS: [M+H]+ calcd. 349.1651, found 349.1645

Enone 17d

^{1}**H NMR** (600 MHz, $CDCl_3$) δ 6.91 (ddd, J = 10.3, 5.8, 2.1 Hz, 1H), 6.05 (dd, J = 10.1, 2.9 Hz, 1H), 5.31 (dd, J = 12.8, 7.5 Hz, 1H), 3.72 (s, 3H), 3.38 (dd, J = 13.1, 4.2 Hz, 1H), 2.39 – 2.23 (m, 4H), 2.23 – 2.18 (m, 2H), 2.17 (d, J = 1.1 Hz, 3H), 2.10 (ddt, J = 14.0, 11.4, 5.9 Hz, 1H), 1.99 (td, J = 12.2, 3.9 Hz, 1H), 1.93 (dt, J = 14.5, 3.4 Hz, 1H), 1.69 (tdd, J = 14.8, 11.8, 3.5 Hz, 1H), 1.22 (td, J = 14.2, 3.8 Hz, 1H), 0.98 (s, 3H).

^{13}**C NMR** (151 MHz, $CDCl_3$) δ 204.12, 199.08, 172.31, 170.35, 148.34, 129.50, 72.77, 66.01, 52.04, 49.52, 41.65, 40.01, 38.01, 36.17, 30.63, 30.47, 23.69, 20.77, 20.37.

R_f: 0.52, 20% EtOAc in toluene (UV, pinkish brown – anisaldehyde)

$[\alpha]_D^{23.1}$ = +3.0 (c = 0.1, $CHCl_3$)

HRMS: [M+H]+ calcd. 349.1651, found 349.1643

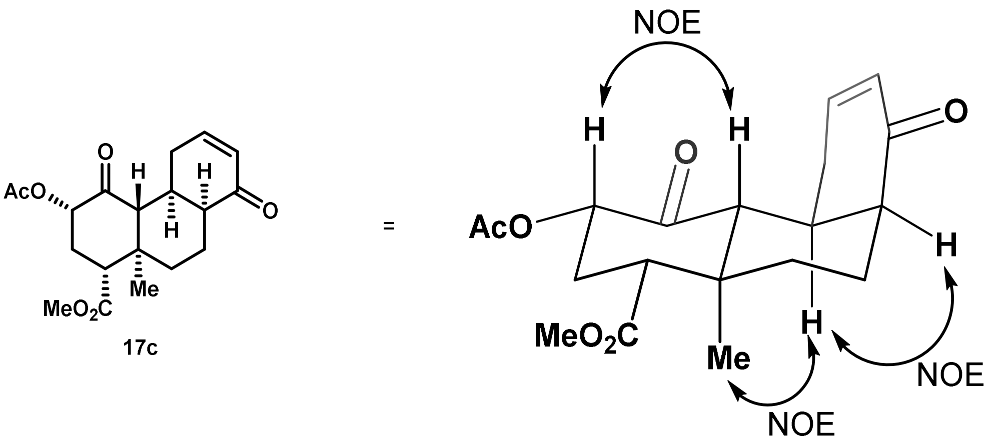

17c was assigned by NOE between the C2 proton and C6 protons confirming the *trans*-ring fusion of the A-B ring system and NOE between the C19 methyl proton, C9 proton and C8 proton confirming the *cis*-ring fusion at the B,C ring.

17b and **17d** were confirmed by X-ray crystallography

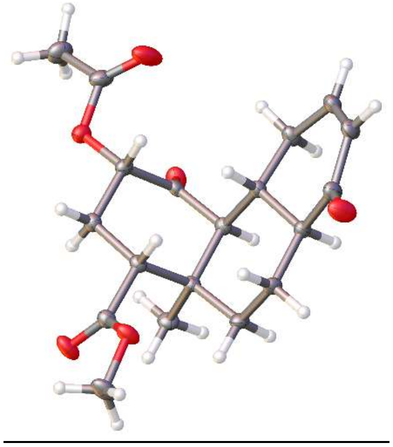

17d X-ray crystal structure

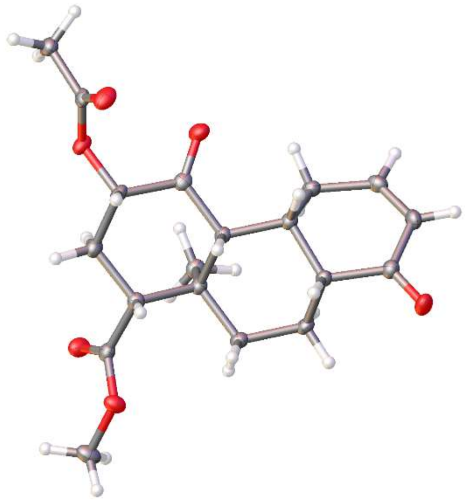

17b X-ray crystal structure

X-ray quality crystals of **17d** were grown by dissolving **17d** in MTBE and diffusing pentane into the solution. X-ray quality crystals of **17b** were grown by dissolving **17b** in minimal amount Et_2O and storing at -20 ºC overnight.

17a was confirmed by X-ray crystallographic analysis of **1**, 2D NMR spectra (COSY, HSQC, NOESY, and HMBC) are also available below for **17a, 17c,** and **17d.** COSY and NOESY are available for **17b**

Figure S9. Enone stereochemical assignment

R-Tipsy in chlorobenzene at 85 ºC (top), pyrrolidine/acetic acid in MeOH/THF at 60 ºC (middle), PTSA in chlorobenzene at 85 ºC (bottom). Crude NMRs with pyrrolidine/acetic acid and PTSA clearly show significant decomposition and broadening in the NMR spectra presumably from enolization of the enal and possible polymerization. Dibromomethane is present in each NMR at 4.90 ppm as an internal standard. Some of the diagnostic signals from **17a** are highlighted in grey.

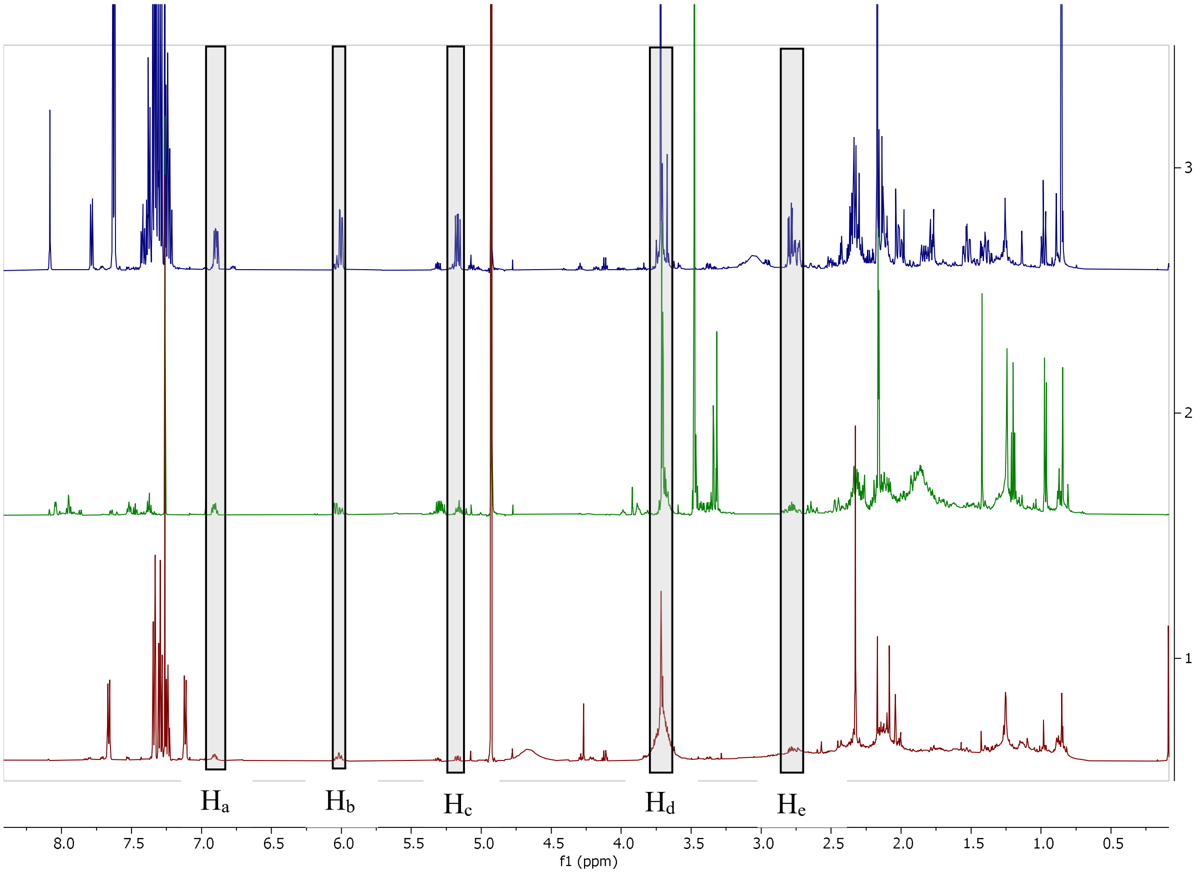

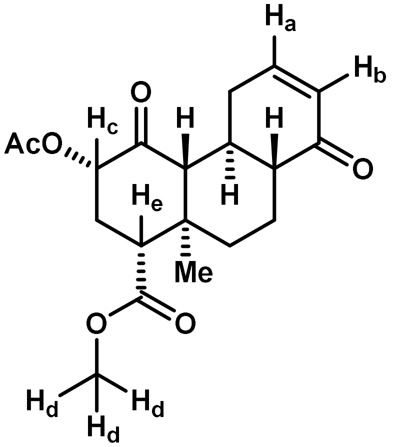

17a with diagnostic protons labeled according to NMR spectra above.

Figure S10. Representative crude spectra of Robinson annulation.

Scheme S13. Prior allylation approach

A key enal intermediate that could undergo Robinson annulation. Oxidation of the allyl unit was laborious and ultimately low yielding which led to the Reformatsky reaction. Detailed procedures and characterizations of the intermediates follow.

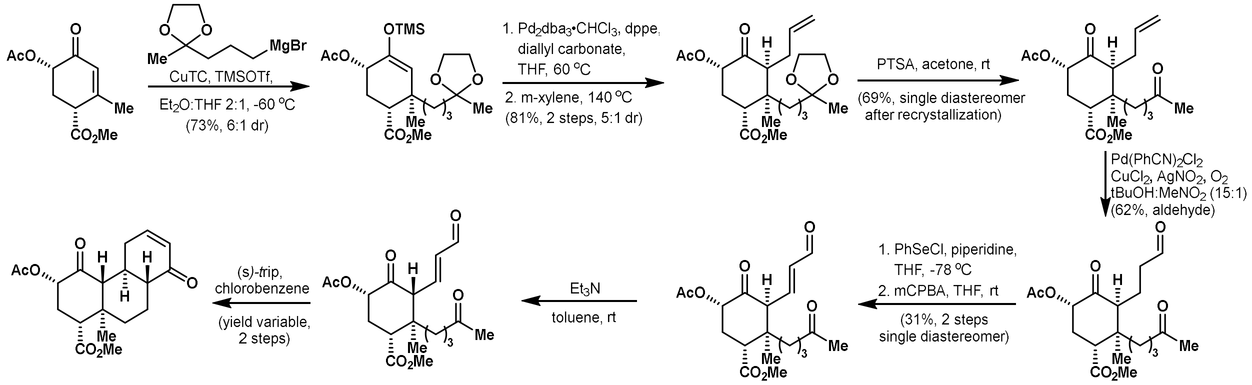

O-allyl **8** and *C*-allyl **9**

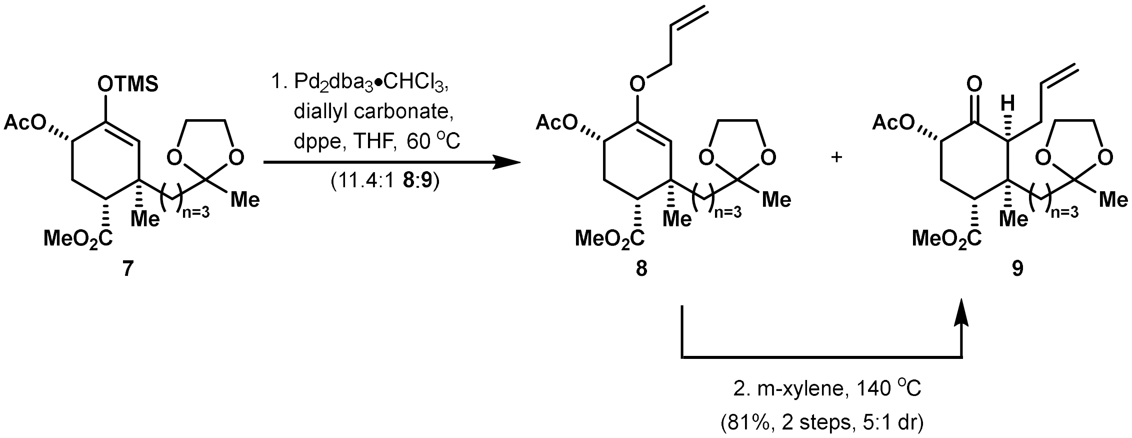

In a flamed dried 250 mL RBF that was backfilled with argon, silyl enol ether **7** (2.44 g, 5.69 mmol, 1 equiv.) was dissolved in anhydrous THF (28 mL). Pd_2dba_3•$CHCl_3$ (294 mg, 0.253 mmol, 0.05 equiv.) and diphenylphosphino ethane (226 mg, 0.56 mmol, 0.11 equiv.) were added to the mixture. Diallyl carbonate (1.63 mL, 11.4 mmol, 2 equiv.) was then added neat. The RBF was submerged in a 60 °C oil bath and stirred until TLC indicated complete consumption of starting material. (Note 1) The solution was cooled to 22 °C and diluted with equal volume Et_2O and filtered through a silica pad. The crude mixture was concentrated to dryness and *m*-xylenes (5 mL) was added and stirred at 140 °C until TLC indicated complete consumption of the *O*-allylated product (typically 3 hours). The reaction was concentrated to remove the xylenes and loaded directly on a silica column eluting with 20→30→40→50% EtOAc in hexanes. The fractions containing the product were collected and concentrated to yield **9** as a colorless oil in a 5:1 mixture of diastereomers (1.83 g, 81%, 2 steps). ^{1}H NMR analysis of the crude indicated a 11.4:1 mixture of compounds **8**:**9** in a separate run of the allylation.

Note 1: Different bottles of Pd_2dba_3•$CHCl_3$ exhibited significantly different rates of reactivity, some requiring overnight reaction and some reaching completion in 1 hour. If significant amounts of TMS removal is observed without allylation, it is recommended a shorter reaction time is tried. The de-allylated product presumably arises from reversible O-allylation and protonation from a trace proton source.

O-allyl 8

^{1}H NMR (600 MHz, $CDCl_3$) δ 5.90 (ddt, *J* = 17.3, 10.3, 5.0 Hz, 1H), 5.49 (ddd, *J* = 10.3, 6.6, 1.1 Hz, 1H), 5.28 (dq, *J* = 17.2, 1.8 Hz, 1H), 5.18 (dq, *J* = 10.6, 1.5 Hz, 1H), 4.46 (d, *J* = 1.1 Hz, 1H), 4.23 – 4.13 (m, 2H), 3.98 – 3.88 (m, 4H), 3.67 (s, 3H), 2.69 (dd, *J* = 13.4, 3.0 Hz, 1H), 2.17 (ddt, *J* = 13.8, 7.2, 3.6 Hz, 1H), 2.12 – 2.04 (m, 1H), 2.08 (s, 3H), 1.71 – 1.55 (m, 3H), 1.54 – 1.43 (m, 1H), 1.37 – 1.23 (m, 2H), 1.31 (s, 3H) 1.01 (s, 3H).

^{13}C NMR (151 MHz, $CDCl_3$) δ 173.38, 170.93, 150.48, 133.18, 116.81, 110.09, 108.48, 68.08, 67.94, 64.75 (2C), 51.61, 44.02, 42.24, 39.78, 38.01, 28.19, 25.14, 23.90, 21.29, 18.75.

R_f: 0.56, 70% EtOAc in hexanes (brown – anisaldehyde)

HRMS: [M+Na]+ calcd. 419.2046, found 419.2046

$[\alpha]_D^{25.3}$ = +0.3 (c = 1.0, $CHCl_3$)

C-allyl 9

^{1}H NMR (600 MHz, $CDCl_3$) δ 5.63 (ddt, *J* = 17.0, 10.0, 7.1 Hz, 1H), 5.41 (dd, *J* = 10.8, 9.3 Hz, 1H), 5.12 (d, *J* = 17.1 Hz, 1H), 5.05 (d, *J* = 10.8 Hz, 1H), 3.99 – 3.85 (m, 4H), 3.69 (s, 3H), 3.02 (dd, *J* = 9.9, 7.4 Hz, 1H), 2.45 – 2.27 (m, 3H), 2.26 – 2.21 (m, 2H), 2.14 (s, 3H), 1.60 – 1.51 (m, 2H), 1.28 – 1.17 (m, 4H), 1.27 (s, 3H) 1.00 (s, 3H).

^{13}C NMR (151 MHz, $CDCl_3$) δ 205.11, 172.89, 170.05, 133.55, 118.10, 109.77, 72.58, 64.84, 64.80, 59.28, 51.90, 46.17, 42.00, 39.71, 37.82, 31.86, 30.84, 24.06, 20.81, 19.39, 17.09.

R_f: 0.44, 70% EtOAc in hexanes (orange – anisaldehyde)

HRMS: [M+H]+ calcd. 397.2226, found 397.2215

$[\alpha]_D^{24.3}$ = -17.5 (c = 1.0, $CHCl_3$)

Allyl **S6**

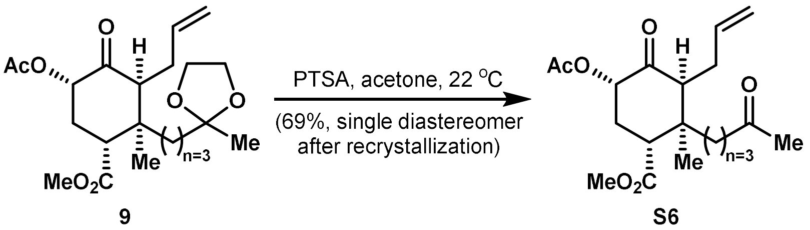

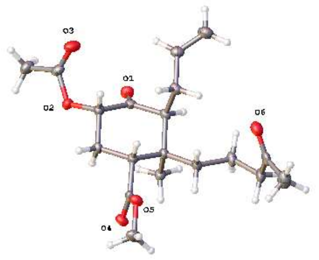

In an RBF C-allyl **9** (1.47 g, 4.6 mmol, 1 equiv.) was dissolved in acetone (50 mL). *p*-toluenesulfonic acid (142 mg, 0.46 mmol, 0.2 equiv.) was added and the mixture was stirred at 22 °C until TLC indicated complete consumption of **9** (typically 2-3 hours). At this point the mixture was concentrated and loaded directly on a silica gel column (120 mL of silica) eluting with 10 →20 →30 →40 % acetone in hexanes. The fractions containing the major diastereomer were concentrated. This mixture was then recrystallized from hot diisoproyl ether to yield a single diastereomer of **S6** as colorless crystals (900 mg, 69%). X-ray quality crystals were obtained by diffusion of pentane into MTBE.

---

^{1}H NMR (600 MHz, $CDCl_3$) δ 5.62 (ddt, *J* = 17.1, 9.9, 7.0 Hz, 1H), 5.40 (dd, *J* = 11.7, 8.5 Hz, 1H), 5.14 (d, *J* = 17.0 Hz, 1H), 5.05 (d, *J* = 9.8 Hz, 1H), 3.68 (s, 3H), 3.01 (dt, *J* = 10.1, 4.2 Hz, 1H), 2.57 – 2.50 (m, 1H), 2.50 – 2.27 (m, 4H), 2.27 – 2.17 (m, 2H), 2.13 (s, 3H), 2.12 (s, 3H), 1.62 – 1.52 (m, 1H), 1.44 (ddt, *J* = 13.5, 9.4, 5.4 Hz, 2H), 1.14 (dddd, *J* = 22.1, 8.3, 6.5, 2.2 Hz, 1H), 1.00 (s, 3H).

^{13}C NMR (151 MHz, $CDCl_3$) δ 208.06, 204.94, 172.80, 170.00, 133.49, 118.13, 72.51, 59.14, 51.94, 46.16, 43.51, 41.92, 37.04, 31.71, 30.84, 30.08, 20.80, 19.35, 16.75.

R_f: 0.26, 30% acetone in hexanes (brown – anisaldehyde)

HRMS: [M+Na]+ calcd. 375.1784, found 375.1781

$[\alpha]_D^{22.5}$ = -10.2 (c = 0.1, $CHCl_3$)

Melting Point: 105-110 °C

Aldehyde **S7**

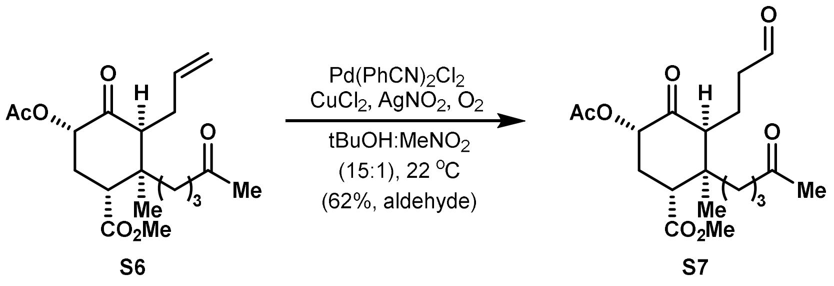

To a RBF containing *t*BuOH (32 mL) and $MeNO_2$ (2 mL) was added $Pd(PhCN)_2Cl_2$ (93 mg, 0.328 mmol, 0.12 equiv.), $CuCl_2$ (64 mg, 0.426 mmol, 0.24 equiv.), and $AgNO_2$ (18 mg, 0.17 mmol, 0.06 equiv.). Oxygen was then bubbled through this solution for 15 minutes with vigorous stirring (avoiding splashing of the solids on the flask). The oxygen bubbling was then stopped but an oxygen balloon was left on the flask. The contents were then stirred for a further 15 minutes at 22 °C. Allyl **S6** (29.1 mg) was then added every 30 minutes for 2 hours (350 mg total). The reaction was then stirred for one hour and these 29.1 mg additions every 30 minutes were then repeated for 2 more hours (an additional 350 mg, 700 mg total, 1.98 mmol, 1 equiv.). The reaction was then stirred for an additional 12 hours (overnight) and analyzed by TLC the following morning to verify consumption of allyl **S6**. The reaction was then quenched with addition of H_2O (75 ml) and EtOAc (50 ml). The layers were separated and the aqueous layer was then extracted twice more with EtOAc (50 ml). The combined organic layers were then washed with brine and dried with Na_2SO_4. The organic layer was filtered and concentrated to yield crude material. This material was purified via silica gel flash column chromatography eluting with a gradient of 40 → 50 → 60 →70% EtOAc in hexanes. The fractions containing product were combined and concentrated to yield **S7** as a colorless oil (454 mg, 62% yield).

^{1}H NMR (600 MHz, $CDCl_3$) δ 9.75 (s, 1H), 5.28 (dd, *J* = 12.4, 7.6 Hz, 1H), 3.69 (s, 3H), 3.02 (dd, *J* = 12.7, 4.4 Hz, 1H), 2.54 (ddd, *J* = 19.0, 9.2, 4.9 Hz, 1H), 2.49 – 2.38 (m, 3H), 2.35 (dd, *J* = 13.1, 4.4 Hz, 1H), 2.31 – 2.18 (m, 2H), 2.15 (s, 3H), 2.07-2.15 (m, 1H) 2.13 (s, 3H), 1.83 (tdd, *J* = 13.7, 8.7, 5.0 Hz, 1H), 1.64 – 1.56 (m, 1H), 1.47 (p, *J* = 6.5 Hz, 2H), 1.19 (ddd, *J* = 13.6, 11.2, 6.1 Hz, 1H), 0.99 (s, 3H).

^{13}C NMR (151 MHz, $CDCl_3$) δ 208.20, 205.56, 200.68, 172.75, 170.31, 72.57, 57.91, 51.98, 46.06, 43.62, 42.16, 40.79, 36.99, 30.56, 30.13, 20.75, 19.44, 19.17, 16.80.

R_f: 0.18, 50% EtOAc in Hexanes (yellow - anisaldehyde)

HRMS: [M+Na]+ calcd. 391.1733, found 391.1731

$[\alpha]_D^{22.4}$ = -31.2 (c = 0.1, $CHCl_3$)

Enal **2b**

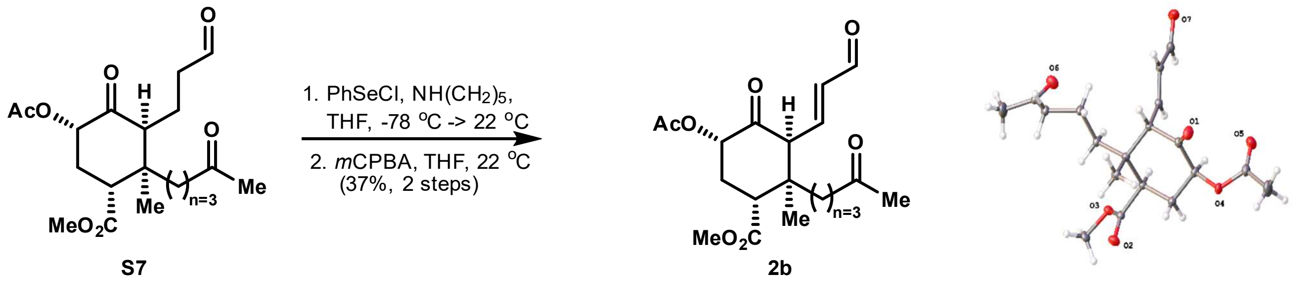

Aldehyde **S7** (50 mg, 0.136 mmol, 1 equiv.) was dissolved in THF (1.2 mL) and piperidine (14.2 µL, 0.190 mmol, 1.4 equiv.) was added. This was stirred at 22 ºC for 10 minutes and then cooled to -78 ºC. Phenyl selenium chloride (32 mg, 0.163 mmol, 1.2 equiv.) was dissolved in THF (0.6 mL) and added dropwise to this solution. The solution was then removed from the cold bath and allowed to warm to 22 ºC. The solution was stirred until complete consumption of aldehyde **S7** was observed by TLC (typically by the time the flask reached 22 ºC). The reaction was quenched by the addition of H_2O (2 mL) and EtOAc (2 mL). The organic layer was separated and the aqueous layer back extracted two more times with EtOAc (2 x 2 mL). The combined organic layers were washed with brine (5 mL) and dried with Na_2SO_4. The crude mixture was vacuum filtered through a silica plug (approximately 10 ml) first with hexanes to remove selenium by-products, followed by 70% EtOAc in hexanes flush to obtain the crude product which was concentrated and used immediately without further purification. To the crude mixture was added 1.2 mL of THF. *m*CPBA (31 mg, 0.18 mmol, 0.75 equiv.) was added at 22 ºC, then a second portion of *m*CPBA (31 mg, 0.18 mmol, 0.75 equiv.) was added to the solution and stirred until dissolved. The reaction was then quenched with sat. $NaHCO_3$ solution (5 ml) and EtOAc (5 mL). The organic layer was separated and the aqueous layer was extracted twice more with EtOAc (2 x 5 mL). The organic layer was washed with brine (10 mL) and dried with Na_2SO_4. The organic layer was then concentrated under a stream of nitrogen until approximately 500 µl (Note 1) remained and immediately loaded on a 5 mL silica plug. This plug was flushed 3x with 30% EtOAc in hexanes and collected as fraction 1. The plug was then flushed 1x with 30% EtOAc in hexanes and collected as fraction 2. The plug was then flushed 4x with 100% EtOAc and collected as fraction 3. The fractions were analyzed by TLC to confirm fraction 3 contained product and fraction 1 and 2 contained non-polar by-products. Fraction 2 was then concentrated and enal **2b** was crystallized from the concentrate with Et_2O to yield **2b** as a white powder (20 mg, 37%, 2 steps). X-ray quality crystals were grown from vapor diffusion of pentane into MTBE.

Note 1: If the crude from the *m*CPBA oxidation is completely concentrated enal **2b** rapidly decomposes in the presence of the non-polar by products.

^{1}H NMR (600 MHz, $CDCl_3$) δ 9.60 (d, J = 7.3 Hz, 1H), 6.97 (dd, J = 15.7, 8.2 Hz, 1H), 6.27 (ddd, J = 15.7, 7.3, 1.3 Hz, 1H), 5.32 (dd, J = 10.3, 9.2 Hz, 1H), 3.70 (s, 3H), 3.40 (dd, J = 8.2, 1.4 Hz, 1H), 3.07 – 2.97 (m, 1H), 2.45 – 2.27 (m, 4H), 2.13 (s, 3H), 2.09 (s, 3H), 1.53 (td, J = 12.8, 3.7 Hz, 1H), 1.46 (dqd, J = 12.8, 6.2, 3.8 Hz, 1H), 1.42-1.33 (m, 1H),1.25 (td, J = 12.7, 3.7 Hz, 1H), 1.09 (s, 3H).

^{13}C NMR (151 MHz, $CDCl_3$) δ 207.73, 200.35, 192.31, 172.13, 169.79, 146.97, 136.88, 73.06, 62.62, 52.15, 46.67, 43.23, 43.18, 37.37, 30.71, 30.05, 20.62, 18.93, 16.61.

R_f: 0.23, 60% EtOAc in hexanes (UV, orange – anisaldehyde)

HRMS: [M+Na]+ calcd. 389.1570, found 389.1570

$[\alpha]_D^{29.3}$ = +5.6 (c = 0.2, $CHCl_3$)

Enal **2a**

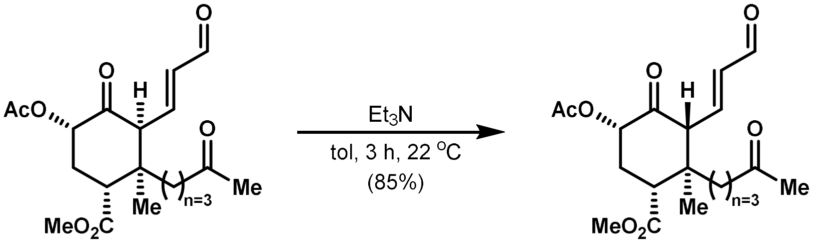

2b 2a

Enal **2b** (55 mg, 0.15 mmol, 1 equiv.) was added to a vial equipped with a stir bar and dissolved in toluene (2 mL). Et_3N (10 µL, 0.14 mmol, 0.9 equiv.) was then added. The reaction was stirred for 3 hours and at this point sat. NH_4Cl solution (3 ml) and EtOAc (3 ml) were added. The layers were separated and the aqueous layer extracted twice more with EtOAc (2 x 3mL). The combined organic layers were washed with brine (5 ml) and dried with Na_2SO_4, filtered and concentrated. ^1H NMR analysis with dibromomethane as an internal standard indicated > 90% consumption of enal **2b** and enal **2a** present (0.13 mmol, 85%) and the crude material was used without further purification. Due to instability on silica gel, enal **2b** was not purified further and optical rotation was not obtained on the crude material.

^{1}H NMR (600 MHz, $CDCl_3$) δ 9.59 (d, *J* = 7.7 Hz, 1H), 6.90 (dd, *J* = 15.9, 9.8 Hz, 1H), 6.16 (dd, *J* = 15.9, 7.7 Hz, 1H), 5.20 (dd, *J* = 12.7, 7.2 Hz, 1H), 3.74 (s, 3H), 3.48 (d, *J* = 9.8 Hz, 1H), 3.17 (dd, *J* = 13.1, 3.9 Hz, 1H), 2.43 – 2.27 (m, 4H), 2.15 (s, 3H), 2.12 (s, 3H), 1.94 – 1.84 (m, 1H), 1.47 (ddq, *J* = 12.5, 10.6, 6.6 Hz, 1H), 1.34 (td, *J* = 13.7, 12.9, 4.1 Hz, 1H), 1.21 – 1.12 (m, 1H), 0.99 (s, 3H).

^{13}C NMR (151 MHz, $CDCl_3$) δ 207.71, 201.31, 193.02, 171.61, 169.88, 148.24, 136.98, 74.48, 57.90, 52.27, 46.14, 45.77, 43.40, 38.09, 30.88, 30.09, 20.68, 18.14, 16.82.

R_f: 0.53, 6% MeOH in DCM (UV, orange – anisaldehyde)

LRMS: [M+H]+ calcd. 367.2, found 349.2 ([M+H]+ -H_2O)

(−)-O6C-20nor-salvinorin A (**1**)

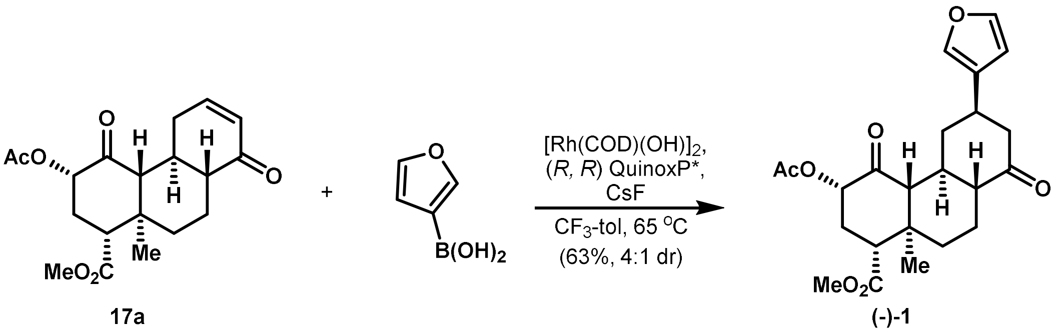

To a flame dried 27 mL reaction tube equipped with a stir bar was added enone **17a** (15 mg, 0.431 mmol, 1 equiv.), 3-furan boronic acid (28.9 mg, 0.258 mmol, 6 equiv.), $[Rh(COD)(OH)]_2$ (9.8 mg, 0.215 mmol, 0.5 equiv.), CsF (39 mg, 0.258 mmol, 6 equiv.), and (*R, R*) Quinox P* (17.3 mg, 0.052 mmol, 1.2 equiv.). The tube was evacuated and back-filled with argon three times and then degassed trifluoro toluene (1.3 mL) was added. The reaction mixture was immediately submerged in a 65 °C oil bath and stirred for 2 hours at which point TLC indicated complete consumption of enone **17a**. The mixture was removed from the oil bath and filtered through a silica plug. Crude NMR analysis indicated a 4:1 diastereomeric ratio of **1** : **18**. The reaction was purified by silica gel chromatography with a gradient of 30 → 40 → 50 → 60% EtOAc in hexanes. **1** was isolated as a white powder (9.2 mg, 51%) and 12-*epi*-**1** (**18**) was isolated as a white film (2.2 mg, 12%). X-ray quality crystals were grown by dissolving the compound in a 3:1 mixture of MTBE:DCM and diffusing pentane into the solution via vapor diffusion.

^{1}H NMR (600 MHz, $CDCl_3$) δ 7.33 (t, *J* = 1.7 Hz, 1H), 7.20 (dt, *J* = 1.5, 0.9 Hz, 1H), 6.27 (dd, *J* = 1.8, 0.9 Hz, 1H), 5.17 (dd, *J* = 12.1, 7.7 Hz, 1H), 3.71 (s, 3H), 3.00 (tt, *J* = 12.7, 4.0 Hz, 1H), 2.78 (dd, *J* = 13.2, 4.1 Hz, 1H), 2.68 – 2.61 (m, 1H), 2.41 – 2.25 (m, 5H), 2.15 (s, 3H), 2.04 (ddd, *J* = 9.8, 5.2, 2.3 Hz, 2H), 1.78 – 1.71 (m, 1H), 1.57 – 1.45 (m, 2H), 1.29 – 1.20 (m, 2H), 0.85 (s, 3H).

^{13}C NMR (151 MHz, $CDCl_3$) δ 209.61, 202.43, 171.88, 170.09, 143.27, 138.02, 128.35, 109.20, 75.28, 60.94, 53.01, 52.33, 52.03, 48.26, 42.83, 37.51, 37.19, 36.42, 34.81, 31.73, 20.77, 19.79, 13.82.

R_f: 0.56, 60% EtOAc in hexanes (blue – anisaldehyde)

$[\alpha]_D^{26.0}$ = -17.9 (c = 0.1, $CHCl_3$)

HRMS: [M+H]+ calcd. 417.1913, found 417.1920

2D NMR: COSY, HSQC, NOESY, HMBC

See below for characterization of **18**

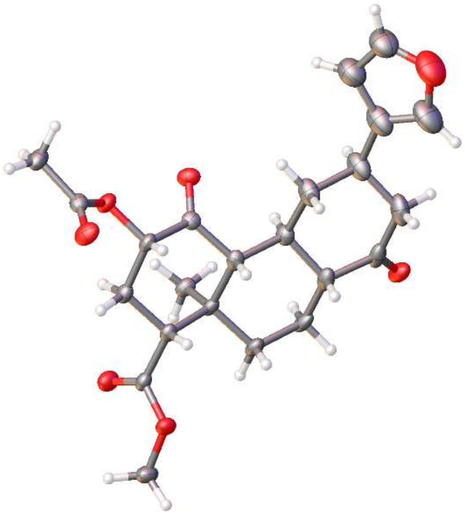

Analog procedures **18-42b**

General arylation procedures are written below. Further functionalization of analogs is also discussed in detail with characterization in the following pages

General Procedure A: To an 8 mL reaction tube $[Rh(COD)(OH)]_2$ (3.3 mg, 0.0073 mmol, 0.5 equiv.) and (*R*)-BINAP (13 mg, 0.0219 mmol, 2 equiv.) were added and dissolved in toluene (300 μL). This mixture was stirred for 15 minutes at 22 °C until all rhodium and BINAP had dissolved and the reaction had turned visually dark red (occasionally sonication was required to complete this process). Then phenyl boronic acid (2.7 mg, 0.0219 mmol, 2 equiv.) and boric acid (1.3 mg, 0.0219 mmol, 2 equiv.) were added to the reaction mixture with minimal air exposure. The solution at this point turns a dark brown color. The solution is then inserted into a 100 °C oil bath and enone **17a** (5 mg, 0.014 mmol, 1 equiv.) in toluene (300 μL) was added to the mixture quickly. The reaction mixture was then stirred for 1 hour and analyzed by TLC for complete consumption of **17a**. The mixture was then cooled to 22 °C and filtered through a silica plug. ^{1}H NMR analysis of the crude showed a 10:1 dr favoring the *nat*-epimer. The crude filtrate was concentrated and purified on preparatory TLC eluting with 5% DCM in Et_2O. **24a** was isolated as a white solid (2.4 mg, 0.0056 mmol, 39% yield). This reaction with phenyl boronic acid was also run on a 30 mg scale which showed an improvement in yield (30 mg isolated, 81%).

General Procedure B: To an 8 mL reaction tube **17a** (5 mg, 0.014 mmol, 1 equiv.), $[Rh(COD)(OH)]_2$ (3.3 mg, 0.0073 mmol, 0.5 equiv.), *(R, R)* QuinoxP* (7.3 mg, 0.0219 mmol , 2 equiv.), 3-thiophene boronic acid (2.8 mg, 0.0219 mmol, 2 equiv.), and boric acid (1.3 mg, 0.0219 mmol, 2 equiv.) were added. The reaction vessel was evacuated and backfilled with argon three times (waiting 5 minutes on high vacuum with each evacuation). Then CF_3-toluene (800 μL) was added to the reaction mixture and the vessel was immediately submerged in a 65 °C oil bath and stirred for three hours. At this point the reaction was analyzed by TLC for consumption of **17a**. The solution was then cooled to 22 °C and filtered through silica. ^{1}H NMR analysis of the crude showed a 1.3:1 dr favoring the *nat*-epimer. The crude filtrate was purified by prepatory TLC eluting with 60% EtOAc. **19** was isolated as a white solid (3.3 mg, 0.0069 mmol, 49% yield).

General Procedure C: To an 8 mL reaction tube **17a** (5 mg, 0.014 mmol, 1 equiv.), $[Rh(COD)(OH)]_2$ (3.3 mg, 0.0073 mmol, 0.5 equiv.), *(R, R)* BenzP* (5 mg, 0.018 mmol , 1.6 equiv.), and 4-pyridyl boronic acid (2 mg, 0.016 mmol, 1.2 equiv.) was dissolved in *t*BuOH (400 μL) and submerged in a 60 °C oil bath (Note 1). The mixture was stirred for 1 hour at which point the mixture was analyzed by TLC to confirm consumption of **17a**. The *t*BuOH was then removed from the reaction tube via a constant stream of nitrogen in the 60 °C oil bath and the reaction redissolved in EtOAc. The reaction was then filtered through a silica plug, concentrated and purified by preparatory TLC eluting with 100% EtOAc in hexanes. ^{1}H NMR analysis of the crude showed a greater than 20:1 dr favoring the nat-epimer. **21** was isolated as a white solid (2.8 mg, 0.0066 mmol, 47% yield).

Note: All solvents used in the Hayashi conjugate additions were degassed prior to use by bubbling argon through the solvent, while sonicating, for 45 minutes.

Analog **18**

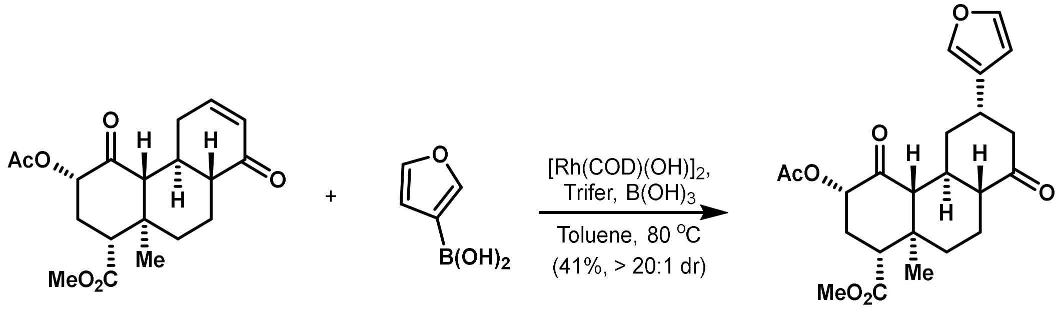

17a **18**

To a vial was added **17a** (8mg, 0.023 mmol, 1 equiv.), $[Rh(COD)(OH)]_2$ (2 mg, 0.0046 mmol, 0.2 equiv.), (*S*)-Trifer (4.2 mg, 0.0092 mmol, 0.4 equiv.), 3-furan boronic acid (5 mg, 0.046 mmol, 2 equiv.), and boric acid (2.8 mg, 0.046 mmol, 2 equiv.). Toluene (300 µl) was added to the vial and the mixture was stirred at 80 °C. After 3 hours the reaction was analyzed by TLC (starting material was still present) and the reaction was quenched by filtration through silica. The product was purified by preparatory TLC 20% Et_2O in DCM, yielding **18** as a white foam (3.9 mg, 0.0094 mmol, 41% yield).

^{1}H NMR (600 MHz, $CDCl_3$) δ 7.38 (d, *J* = 2.2 Hz, 1H), 7.34 (s, 1H), 6.42 (s, 1H), 5.17 (dd, *J* = 12.4, 7.7 Hz, 1H), 3.70 (s, 3H), 3.40 (s, 1H), 2.76 (d, *J* = 13.0 Hz, 1H), 2.73 – 2.62 (m, 2H), 2.41 (d, *J* = 13.5 Hz, 1H), 2.34 (m, 2H), 2.27 (q, *J* = 13.0 Hz, 1H), 2.17 (s, 3H), 2.07-1.97 (m, 2H), 1.76 (d, *J* = 14.3 Hz, 1H), 1.69 (d, *J* = 13.1 Hz, 1H), 1.56-1.50 (m, 1H), 1.46 (t, *J* = 13.9 Hz, 1H), 1.37 (q, *J* = 13.5, 13.0 Hz, 1H), 0.74 (s, 3H).

^{13}C NMR (151 MHz, $CDCl_3$) δ 210.04, 202.37, 171.89, 170.21, 143.44, 139.98, 126.83, 110.05, 75.32, 61.00, 53.67, 52.36, 52.00, 45.49, 42.74, 37.58, 34.30, 32.88, 32.77, 31.65, 20.84, 19.72, 13.82.

R_f: 0.55, 20% Et_2O in DCM (UV, blue - anisaldehyde)

$[\alpha]_D^{23.6}$ = -23.7 (c = 0.1, $CHCl_3$)

HRMS: [M+H]+ calcd. 417.1913, found 417.1907

2D NMR: COSY, HSQC, NOESY, HMBC

We hypothesize that the strong preference for axial addition of the aryl group (see Table **S8** for full optimization) is due to a Fürst-Plattner like effect where the axial approach allows for a chair-like transition state in the bond formation, while an equatorial approach forces a twist-boat transition state. The use of chiral ligands was able to override this selectivity likely through a match/mismatch scenario.

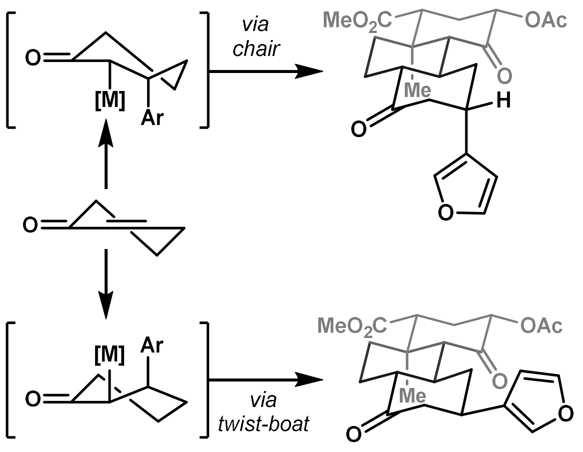

Figure S11. Discussion of arylation stereoselectivity

Analog **36**

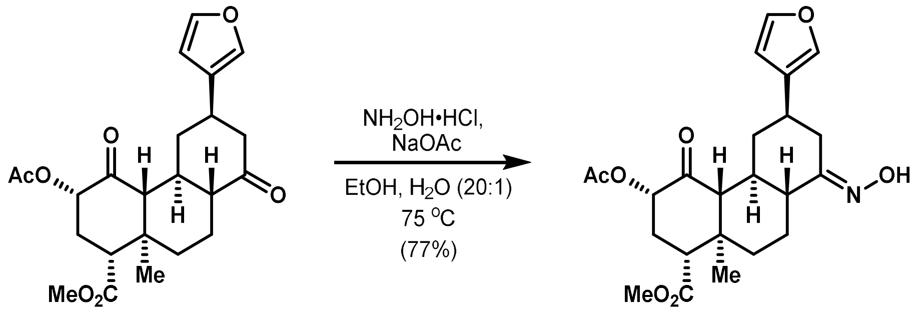

1 36

1 (5 mg, 0.012 mmol, 1 equiv.) was dissolved in EtOH (1 mL) and H_2O (50 µL). NaOAc (10 mg, 0.12 mmol, 10 equiv.) and NH_2OH•HCl (10 mg, 0.14 mmol, 12 equiv.) were added. This mixture was stirred at 75 °C for 1 hour at which point it was analyzed by TLC to confirm consumption of **17a**. The mixture was then concentrated under a constant flow of nitrogen. The crude material was suspended in minimal amount of DCM and loaded directly onto a silica gel preparatory TLC plate. Elution with 60% EtOAc in hexanes followed by collection of the product containing band afforded **36** (4.0 mg, 0.0093 mmol, 77% yield) as a white film.

^{1}H NMR (600 MHz, $CDCl_3$) δ 7.33 (q, *J* = 1.7 Hz, 1H), 7.22 (s, 1H), 6.30 (s, 1H), 5.16 (dd, *J* = 12.3, 7.8 Hz, 1H), 3.71 (s, 3H), 3.67 (ddd, *J* = 13.9, 4.7, 2.1 Hz, 1H), 2.78 (dd, *J* = 13.2, 4.2 Hz, 1H), 2.74 (tt, *J* = 12.5, 3.9 Hz, 1H), 2.35 (ddt, *J* = 12.2, 8.7, 4.0 Hz, 1H), 2.31 – 2.20 (m, 3H), 2.15 (s, 3H), 1.95-1.92 (m, 1H), 1.87 – 1.80 (m, 2H), 1.74 (dt, *J* = 13.0, 3.0 Hz, 1H), 1.66 – 1.48 (m, 3H), 0.99 (td, *J* = 12.5, 10.8 Hz, 1H), 0.87 (s, 3H).

^{13}C NMR (151 MHz, $CDCl_3$) δ 202.58, 172.01, 170.08, 160.51, 143.00, 138.09, 129.01, 109.43, 75.32, 60.88, 52.38, 51.99, 46.04, 42.91, 38.19, 37.67, 36.72, 33.47, 31.81, 31.07, 21.21, 20.79, 13.95.

$[\alpha]_D^{27.6}$ = -6.8 (c = 0.1, $CHCl_3$)

R_f: 0.53, 60% EtOAc in hexanes (purple – anisaldehyde)

HRMS: [M+H]+ calcd. 432.2017, found 432.2017

Analogs **41a** and **41b**

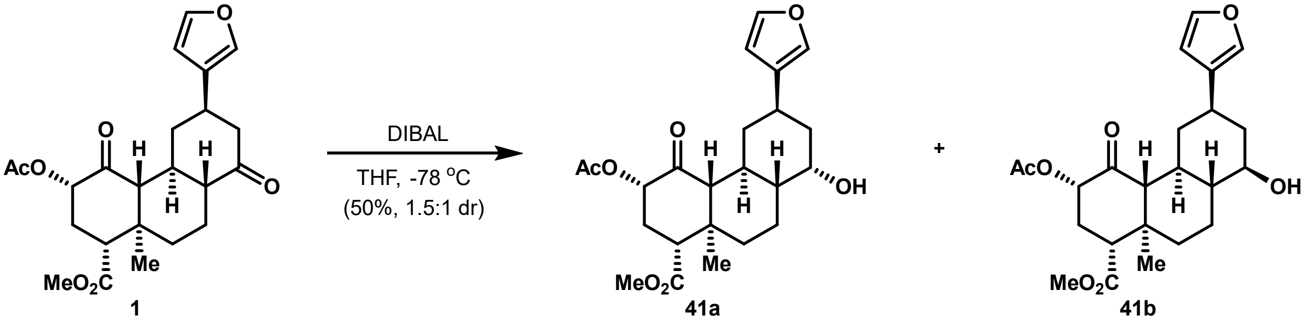

In a 8 mL reaction tube, **1** (5 mg, 0.012 mmol, 1 equiv.) was dissolved in THF (500 µL) and cooled to -78 °C. DIBAL 1 M in hexanes (15 µL, 0.015 mmol, 1.25 equiv.) was added to the solution. The mixture was stirred for 20 minutes and then analyzed by TLC to confirm consumption of starting material **1**. AcOH (5 µL) was then added to quench the reaction, and the reaction was loaded directly onto a preparatory TLC plate (diluting with a small amount of DCM). The preparatory TLC plate was eluted with 60% EtOAc in hexanes and the bands containing the diastereomers were collected yielding **41b** (1.5 mg, 0.0036 mmol, 30% yield) and **41a** (1.0 mg, 0.0024 mmol, 20% yield) both as white solids. Stereochemistry of the alcohols were assigned by coupling and NOESY, see NMR section below.

41b

^{1}H NMR (600 MHz, $CDCl_3$) δ 7.30 (t, J = 1.7 Hz, 1H), 7.17 (dt, J = 1.7, 0.9 Hz, 1H), 6.26 (dd, J = 1.9, 0.9 Hz, 1H), 5.18 – 5.10 (m, 1H), 3.71 (s, 3H), 3.43 (td, J = 10.3, 4.3 Hz, 1H), 2.77 (dd, J = 13.2, 4.1 Hz, 1H), 2.70 – 2.62 (m, 1H), 2.35 (ddd, J = 13.2, 7.6, 4.2 Hz, 1H), 2.28 (td, J = 13.3, 12.3 Hz, 1H), 2.25 – 2.17 (m, 2H), 2.15 (s, 3H), 2.12 – 2.03 (m, 2H), 1.75 – 1.70 (m, 2H), 1.51 (td, J = 13.4, 3.8 Hz, 1H), 1.36 (td, J = 12.4, 11.0 Hz, 1H), 1.15 (qd, J = 13.5, 3.5 Hz, 1H), 1.05 – 0.96 (m, 1H), 0.87 (s, 3H), 0.74 (q, J = 12.1 Hz, 1H).

^{13}C NMR (151 MHz, $CDCl_3$) δ 203.08, 172.14, 170.05, 142.82, 137.90, 129.40, 109.59, 75.37, 73.89, 60.31, 52.45, 51.93, 48.72, 42.94, 42.52, 38.22, 37.32, 33.87, 32.05, 31.92, 23.28, 20.79, 14.03.

R_f: 0.35, 60% EtOAc in hexanes (blue – anisaldehyde)

$[\alpha]_D^{27.3}$ = -29.7 (c = 0.1, $CHCl_3$)

HRMS: [M+H]+ calcd. 419.2070, found 419.2090

41a

^{1}H NMR (600 MHz, $CDCl_3$) δ 7.30 (t, J = 1.7 Hz, 1H), 7.16 (dt, J = 1.7, 0.9 Hz, 1H), 6.25 (dd, J = 1.9, 0.9 Hz, 1H), 5.18 – 5.15 (dd, J = 12.3, 7.4 Hz, 1H), 3.94 – 3.88 (m, 1H), 3.70 (s, 3H), 2.99 (tt, J = 12.7, 3.5, 1H), 2.76 (dd, J = 13.1, 4.2 Hz, 1H), 2.38 – 2.24 (m, 2H), 2.22 – 2.10 (m, 3H), 2.15 (s, 3H), 2.09 – 2.03 (m, 1H), 1.72 (dt, J = 12.7, 3.0 Hz, 1H), 1.68 – 1.50 (m, 3H), 1.42 (dd, J = 13.0, 3.5 Hz, 1H), 1.14 (t, J = 10.9 Hz, 1H), 0.89 (s, 3H), 0.78 – 0.69 (m, 1H).

^{13}C NMR (151 MHz, $CDCl_3$) δ 202.87, 172.17, 170.09, 142.73, 137.95, 129.99, 109.69, 75.37, 70.35, 60.96, 52.52, 51.90, 45.67, 43.03, 40.90, 38.71, 37.93, 31.84, 29.11, 28.00, 24.14, 20.82, 14.03.

R_f: 0.52, 60% EtOAc in hexanes (blue – anisaldehyde)

$[\alpha]_D^{23.6}$ = -27.6 (c = 0.1, $CHCl_3$)

HRMS: [M+H]+ calcd. 419.2070, found 419.2082

Analog **24a**

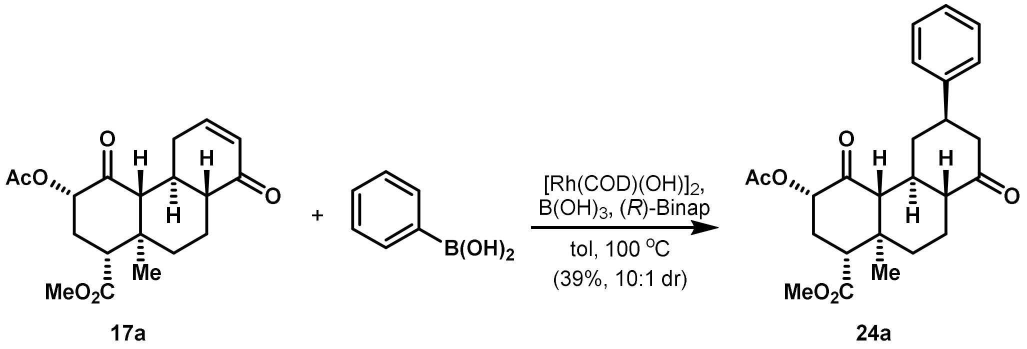

See General Procedure A

^{1}H NMR (600 MHz, $CDCl_3$) δ 7.35 – 7.28 (m, 2H), 7.23 – 7.15 (m, 3H), 5.16 (dd, J = 12.3, 7.6 Hz, 1H), 3.72 (s, 3H), 3.06 (t, J = 13.0 Hz, 1H), 2.79 (dd, J = 12.9, 2.9 Hz, 1H), 2.62 (dd, J = 13.4, 4.6 Hz, 1H), 2.52 (t, J = 13.4 Hz, 1H), 2.42 – 2.24 (m, 4H), 2.13 (s, 3H), 2.10-2.05 (m, 2H), 1.82 (d, J = 12.9 Hz, 1H), 1.76 (d, J = 9.6 Hz, 1H), 1.57-1.52 (m, 2H) 1.41 (q, J = 11.8 Hz, 1H), 0.86 (s, 3H).

^{13}C NMR (151 MHz, $CDCl_3$) δ 210.01, 202.35, 171.91, 170.07, 143.82, 128.82, 126.93, 126.73, 75.28, 61.03, 53.07, 52.36, 52.03, 49.07, 44.16, 42.86, 37.77, 37.56, 36.83, 31.72, 20.73, 19.86, 13.84.

R_f: 0.68 60% EtOAc in Hex, UV, Brown (anisaldehyde)

$[\alpha]_D^{26.7}$ = -15.0 (c = 0.1, EtOAc)

HRMS: [M+H]+ calcd. 427.2121, found 427.2126

X-ray quality crystals were grown by slow evaporation from EtOAc

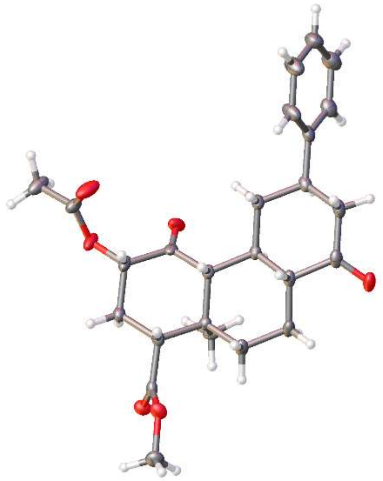

Analog **24b**

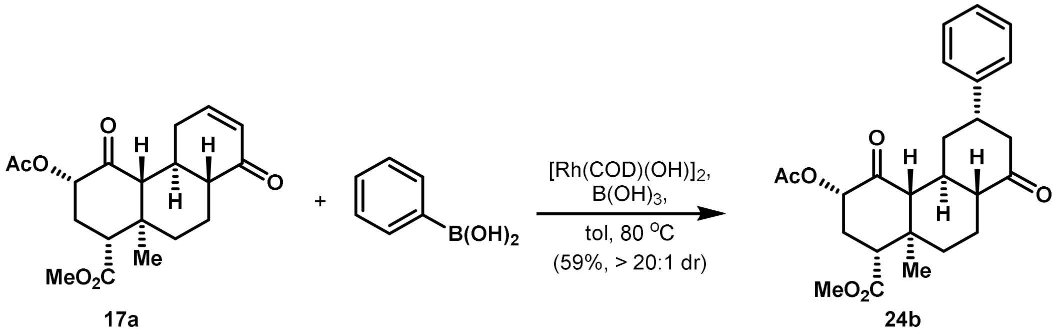

General Procedure A was followed, with the change that the reaction mixture was heated at 80 °C instead of 100 °C, without (*R*)-Binap (ligandless), completely favoring the unnatural diastereomer. The product was purified with preparatory TLC with 60% EtOAc in hexanes. **24b** was isolated as a white solid (3.6 mg, 0.0084 mmol, 59% yield).

^{1}H NMR (600 MHz, $CDCl_3$) δ 7.31 (t, *J* = 7.1 Hz, 2H), 7.26 (d, *J* = 6.3 Hz, 2H), 7.20 (t, *J* = 7.5 Hz, 1H), 5.15 (dd, *J* = 12.5, 7.4 Hz, 1H), 3.69 (s, 3H), 3.56 – 3.51 (m, 1H), 2.88 (ddd, *J* = 15.5, 4.3, 2.1 Hz, 1H), 2.76 (dd, *J* = 13.5, 3.6 Hz, 1H), 2.69 (dd, *J* = 15.4, 6.3 Hz, 1H), 2.46 (d, *J* = 13.7 Hz, 1H), 2.38 – 2.27 (m, 2H), 2.23 (q, *J* = 13.1 Hz, 1H), 2.15 (s, 3H), 2.06 (ddd, *J* = 13.9, 8.8, 3.0 Hz, 1H), 1.98 (tt, *J* = 12.5, 6.6 Hz, 1H), 1.88 (dt, *J* = 14.6, 3.2 Hz, 1H), 1.70 (dt, *J* = 12.9, 3.2 Hz, 1H), 1.67 – 1.59 (m, 1H), 1.48 (dd, *J* = 15.3, 12.1 Hz, 1H), 1.39 (q, *J* = 13.2, 12.2 Hz, 1H), 0.71 (s, 3H).

^{13}C NMR (151 MHz, $CDCl_3$) δ 211.20, 201.95, 171.92, 170.16, 143.18, 128.66, 127.44, 126.50, 75.26, 61.49, 52.93, 52.35, 51.99, 44.93, 42.73, 39.17, 37.68, 35.81, 31.64, 31.58, 20.84, 19.94, 13.79.

R_f: 0.60, 60% EtOAc in Hexanes, UV, Brown (anisaldehyde)

$[\alpha]_D^{26.3}$ = -48.8 (c = 0.1, EtOAc)

HRMS: [M+H]+ calcd. 427.2121, found 427. 2122

X-ray quality crystals were grown from slow evaporation of a mixture of $CDCl_3$, MTBE, and pentane

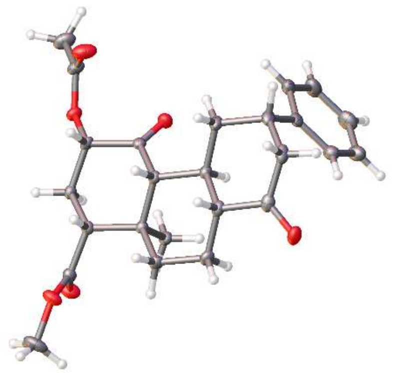

Analog **28**

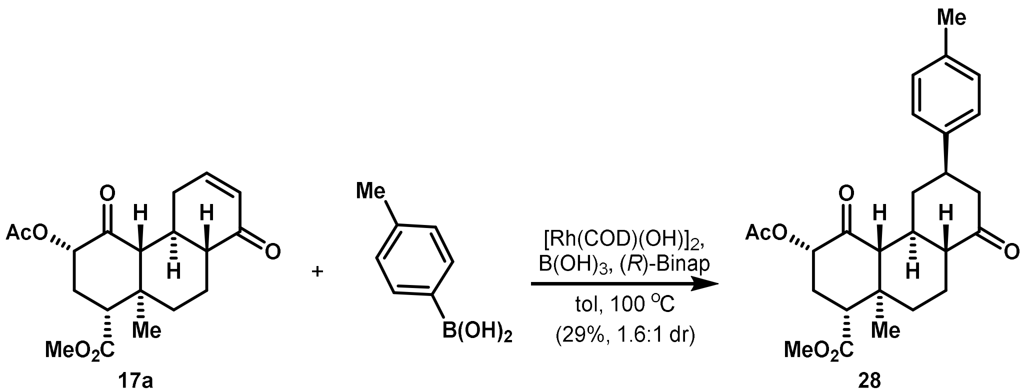

General Procedure A was followed, using 4-methyl phenyl boronic acid (8 mg, 0.059 mmol, 4.2 equiv.). The reaction mixture was purified by preparatory TLC eluting with 10% Et_2O in DCM. **28** was isolated as a white solid (1.8 mg, 0.0040 mmol, 29% yield).

^{1}H NMR (600 MHz, $CDCl_3$) δ 7.10 (d, J = 7.8 Hz, 2H), 7.06 (d, J = 8.1 Hz, 2H), 5.15 (dd, J = 12.4, 7.8 Hz, 1H), 3.72 (s, 3H), 3.03 (tt, J = 12.7, 3.9 Hz, 1H), 2.79 (dd, J = 13.1, 4.2 Hz, 1H), 2.60 (ddd, J = 13.4, 4.3, 1.9 Hz, 1H), 2.50 (dd, J = 13.9, 12.9 Hz, 1H), 2.39 – 2.25 (m, 4H), 2.30 (s, 3H), 2.12 (s, 3H), 2.10 – 2.04 (m, 2H), 1.84 – 1.79 (m, 1H), 1.79 – 1.71 (m, 1H), 1.59-1.54 (m, 1H) 1.54 – 1.47 (m, 1H), 1.38 (td, J = 12.7, 10.3 Hz, 1H), 0.86 (s, 3H).

^{13}C NMR (151 MHz, $CDCl_3$) δ 210.15, 202.34, 171.92, 170.03, 140.92, 136.49, 129.46, 126.58, 75.26, 61.05, 53.05, 52.37, 52.02, 49.18, 43.78, 42.86, 37.92, 37.57, 36.82, 31.72, 21.11, 20.73, 19.87, 13.84.

R_f: 0.68, 60% EtOAc in Hexanes, (UV, Brown - anisaldehyde)

$[\alpha]_D^{26.3}$ = -15.7 (c = 0.1, $CHCl_3$)

HRMS: [M+H]+ calcd. 441.2277, found 441.2267

Analog **30**

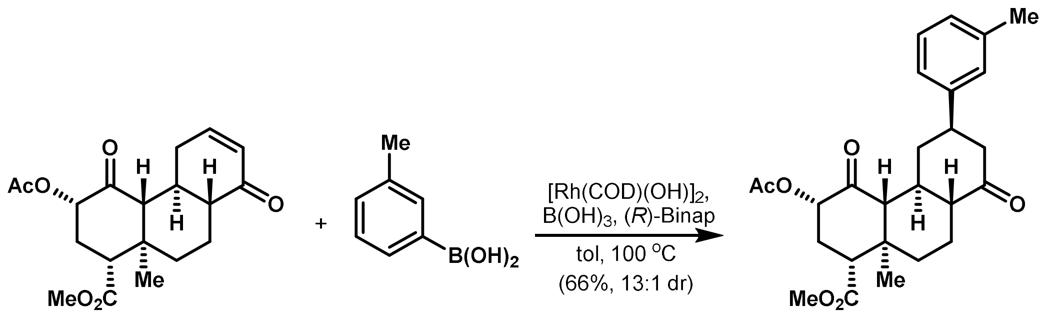

General Procedure A was followed using 3-methyl phenyl boronic acid (8 mg, 0.059 mmol, 4.2 equiv.). The reaction mixture was purified by preparatory TLC eluting with 10% Et_2O in DCM. **30** was isolated as a white solid (4.1 mg, 0.0093 mmol, 66% yield).

^{1}H NMR (600 MHz, $CDCl_3$) δ 7.18 (t, *J* = 7.5 Hz, 1H), 7.02 (ddt, *J* = 7.5, 1.8, 0.9 Hz, 1H), 6.99 – 6.93 (m, 2H), 5.16 (ddd, *J* = 12.1, 7.6, 0.9 Hz, 1H), 3.72 (s, 3H), 3.02 (tt, *J* = 12.7, 3.9 Hz, 1H), 2.79 (dd, *J* = 13.1, 4.2 Hz, 1H), 2.60 (ddd, *J* = 13.4, 4.3, 1.9 Hz, 1H), 2.52 (td, *J* = 13.4, 1.0 Hz, 1H), 2.37-2.26 (m, 4H) 2.32 (s, 3H), 2.13 (s, 3H), 2.10-2.03 (m, 2H), 1.85 – 1.79 (m, 1H), 1.78 – 1.73 (m, 1H), 1.60 – 1.48 (m, 2H), 1.40 (td, *J* = 12.6, 10.2 Hz, 1H), 0.86 (s, 3H).

^{13}C NMR (151 MHz, $CDCl_3$) δ 210.11, 202.32, 171.91, 170.06, 143.81, 138.40, 128.70, 127.67, 127.54, 123.73, 75.26, 61.04, 53.07, 52.37, 52.02, 49.12, 44.18, 42.84, 37.83, 37.57, 36.85, 31.72, 21.59, 20.73, 19.86, 13.84.

R_f: 0.68, 60% EtOAc in Hexanes, (UV, Brown - anisaldehyde)

$[\alpha]_D^{27.7}$ = -14.4 (c = 0.1, $CHCl_3$)

HRMS: [M+H]+ calcd. 441.2277, found 441.2276

Analog **31**

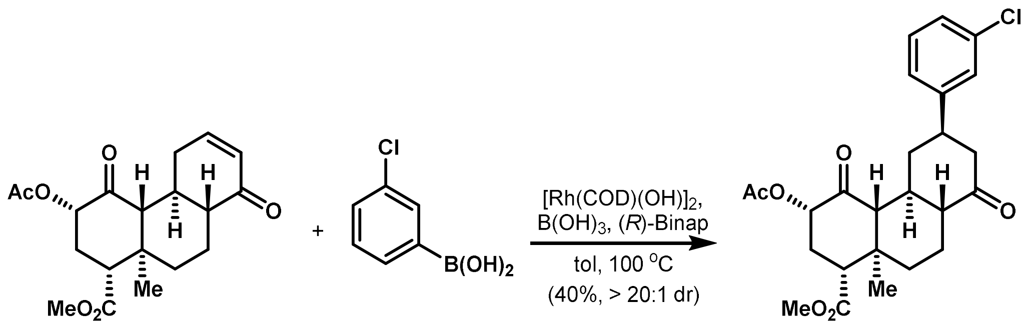

General Procedure A was followed using 3-chloro phenyl boronic acid (6 mg, 0.038 mmol, 2.7 equiv.). The reaction mixture was purified by preparatory TLC eluting with 10% Et_2O in DCM. **31** was isolated as a white solid (2.6 mg, 0.0056 mmol, 40% yield).

^{1}H NMR (600 MHz, $CDCl_3$) δ 7.25 – 7.14 (m, 3H), 7.05 (dt, *J* = 7.5, 1.5 Hz, 1H), 5.19 – 5.12 (m, 1H), 3.72 (s, 3H), 3.04 (tt, *J* = 12.9, 4.0 Hz, 1H), 2.79 (dd, *J* = 13.2, 4.2 Hz, 1H), 2.60 (ddd, *J* = 13.5, 4.3, 2.0 Hz, 1H), 2.48 (t, *J* = 13.3 Hz, 1H), 2.40 – 2.25 (m, 4H), 2.13 (s, 3H), 2.10 – 2.04 (m, 2H), 1.85-1.80 (m, 1H), 1.79 – 1.73 (m, 1H), 1.59 – 1.47 (m, 2H), 1.38 (td, *J* = 12.6, 10.2 Hz, 1H), 0.86 (s, 3H).

^{13}C NMR (151 MHz, $CDCl_3$) δ 209.37, 202.38, 171.86, 170.09, 145.80, 134.62, 130.12, 127.15, 126.89, 125.14, 75.27, 60.93, 53.03, 52.32, 52.03, 48.78, 43.83, 42.85, 37.49 (2C), 36.70, 31.68, 20.72, 19.81, 13.80.

R_f: 0.62, 10% Et_2O in DCM, (UV, Red - anisaldehyde)

$[\alpha]_D$ $^{27.2}$ = -4.8 (c = 0.1, $CHCl_3$)

HRMS: [M+H]+ calcd. 461.1731, found 461.1722

Analog **29**

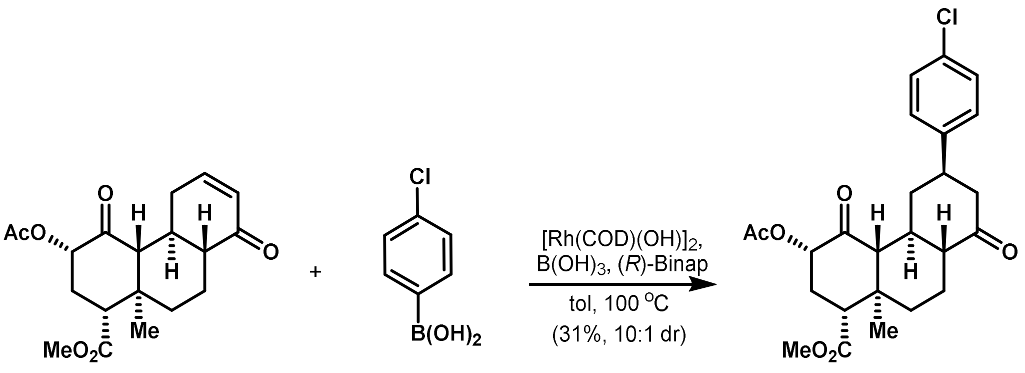

General Procedure A was followed using 4-chloro phenyl boronic acid (6 mg, 0.038 mmol, 2.7 equiv.). The product was purified by preparatory TLC eluting with 10% Et_2O in DCM. **29** was isolated as a white solid (2.0 mg, 0.0043 mmol, 31% yield).

^{1}H NMR (600 MHz, $CDCl_3$) δ 7.27-7.24 (m, 2H) 7.12-7.08 (m, 2H), 5.15 (dd, *J* = 12.4, 7.5 Hz, 1H), 3.72 (s, 3H), 3.04 (tt, *J* = 12.9, 3.8 Hz, 1H), 2.79 (dd, *J* = 13.2, 4.2 Hz, 1H), 2.59 (ddd, *J* = 13.3, 4.2, 1.9 Hz, 1H), 2.46 (t, *J* = 13.4 Hz, 1H), 2.40 – 2.25 (m, 4H), 2.13 (s, 3H), 2.09 – 2.02 (m, 2H), 1.82 (d, *J* = 12.3 Hz, 1H), 1.79 – 1.73 (m, 1H), 1.59 – 1.47 (m, 2H), 1.37 (q, *J* = 12.4 Hz, 1H), 0.86 (s, 3H).

^{13}C NMR (151 MHz, $CDCl_3$) δ 209.48, 202.40, 171.85, 170.07, 142.26, 132.60, 128.93, 128.09, 75.28, 60.97, 53.03, 52.34, 52.04, 48.93, 43.50, 42.87, 37.63, 37.51, 36.72, 31.69, 20.72, 19.82, 13.81.

R_f: 0.64, 10% Et_2O in DCM, (UV, Red - anisaldehyde)

$[\alpha]_D^{26.3}$ = -5.7 (c = 0.1, $CHCl_3$)

HRMS: [M+H]+ calcd. 461.1731, found 461.1724

Analog **32**

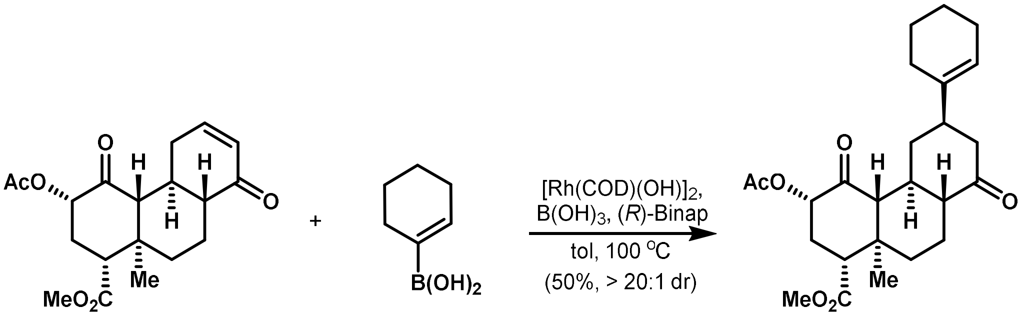

General Procedure A was followed using cyclohexenyl boronic acid (4 mg, 0.032 mmol, 2.3 equiv.). The product was purified by preparatory TLC eluting with 50% EtOAc in hexanes. **34** was isolated as a white solid (3.0 mg, 0.0070 mmol, 50% yield).

^{1}H NMR (600 MHz, C_6D_6) δ 5.29 (dt, J = 4.7, 2.6 Hz, 1H), 5.01 – 4.95 (m, 1H), 3.27 (s, 3H), 2.44 (ddd, J = 12.7, 3.7, 2.0 Hz, 1H), 2.40 – 2.26 (m, 3H), 2.12 (q, J = 15.2, 14.2 Hz, 1H), 2.08 – 1.96 (m, 2H), 1.91 – 1.79 (m, 3H), 1.77 (s, 3H), 1.78 – 1.66 (m, 4H), 1.57 – 1.34 (m, 7H), 1.15 (td, J = 13.0, 3.9 Hz, 1H), 0.80 (q, J = 11.8 Hz, 1H), 0.72 (s, 3H).

^{13}C NMR (151 MHz, C_6D_6) δ 208.51, 201.93, 171.57, 169.44, 139.79, 120.73, 75.46, 60.62, 52.69, 52.22, 51.18, 46.67, 45.47, 42.61, 37.61, 36.66, 35.30, 31.82, 26.61, 25.43, 23.22, 22.81, 20.44, 20.22, 13.65.

R_f: 0.64, 50% EtOAc in hex, (Brown – $KMnO_4$)

$[\alpha]_D^{29.0}$ = -36.9 (c = 0.1, EtOAc)

HRMS: [M+H]+ calcd. 431.2434, found 431.2439

2D NMR: HSQC, NOESY

X-ray quality crystals were grown from vapor diffusion of pentane into a 3:1 mixture of MTBE:$CDCl_3$

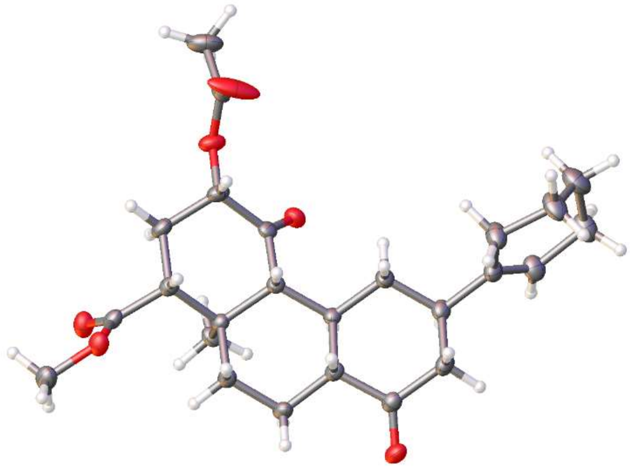

Analog **34**

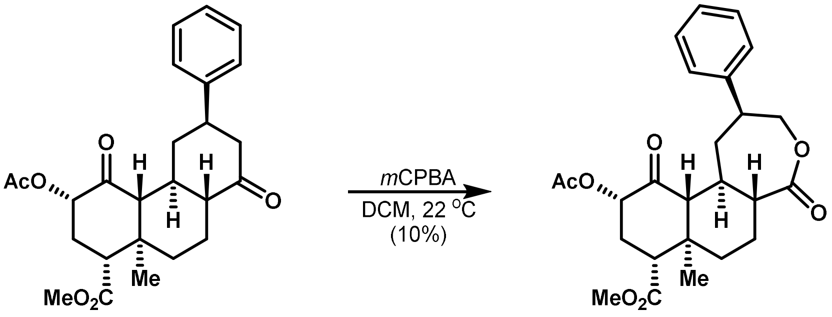

24a **34**

In a 20 ml scintallation vial, **24a** (12 mg, 0.028 mmol, 1 equiv.) was dissolved in DCM (400 μL). *m*CPBA was added to the vial (20 mg, 0.12 mmol, 4.14 equiv.) and the solution stirred for 2 hours at 22 °C. The mixture was loaded directly onto a prepatory TLC plate eluting with 60% EtOAc in hexanes. The band containing the product was collected and purified a second time by prepatory TLC eluting with 15% DCM in Et_2O. The band containing the product was collected and concentrated to yield **34** as a white solid (1.3 mg, 0.0029, 10% yield).

^{1}H NMR (600 MHz, $CDCl_3$) δ 7.29 (t, *J* = 7.6 Hz, 2H), 7.24 – 7.20 (m, 1H), 7.12 – 7.07 (m, 2H), 5.13 (dd, *J* = 12.2, 7.6 Hz, 1H), 4.40 (dd, *J* = 12.4, 9.7 Hz, 1H), 4.23 (dt, *J* = 12.3, 1.9 Hz, 1H), 3.72 (s, 3H), 3.02 (t, *J* = 11.1 Hz, 1H), 2.76 (dd, *J* = 13.2, 4.2 Hz, 1H), 2.64 (td, *J* = 11.7, 5.3 Hz, 1H), 2.42 – 2.24 (m, 3H), 2.22 – 2.12 (m, 3H), 2.11 (s, 3H), 1.77 (tt, *J* = 14.6, 3.9 Hz, 2H), 1.49 (dt, *J* = 14.1, 4.1, 1H), 1.41 – 1.32 (m, 1H), 0.88 (s, 3H).

^{13}C NMR (151 MHz, $CDCl_3$) δ 202.23, 176.04, 171.71, 169.99, 141.87, 129.12, 127.39, 126.90, 75.27, 72.41, 61.95, 52.11, 52.09, 45.25, 44.38, 43.06, 42.21, 36.33, 31.96, 30.81, 22.68, 20.74, 13.83.

R_f: 0.64, 15% Et_2O in DCM, (UV, Pink - anisaldehyde)

$[\alpha]_D^{28.1}$ = -15.6 (c = 0.1, $CHCl_3$)

LRMS: [M+H]+ calcd. 443.2, found 443.2, [M+Na]+ calcd. 465.2, found 465.2

2D NMR: HSQC, COSY

Analogs **40a** and **40b**

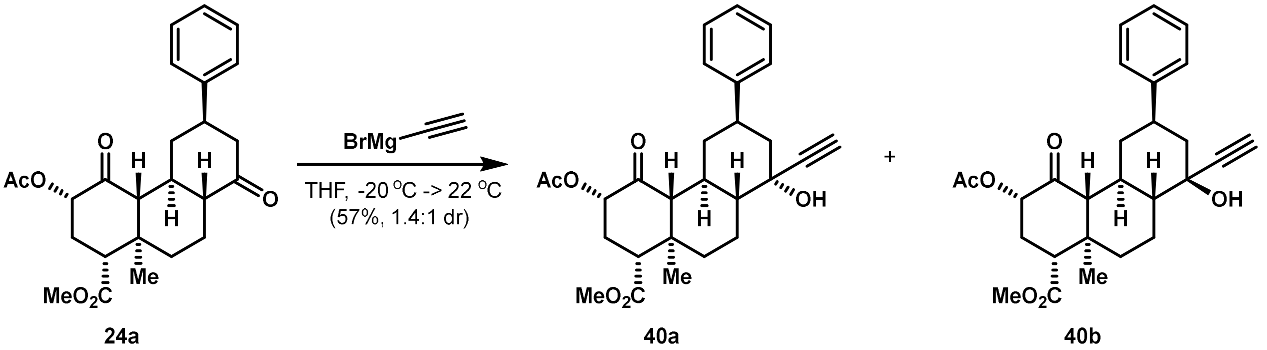

24a (5 mg, 0.012 mmol, 1 equiv.) was dissolved in anhydrous THF (200 µL) in an 8 mL reaction tube. This was cooled to -20 ºC. 0.5 M ethynyl magnesium bromide (25 µL, 0.0125 mmol, 1.05 equiv.) was added and the solution was left to stir for 10 minutes then another 25 µl of 0.5M ethynyl magnesium bromide was added. The reaction was allowed to warm to 22 ºC and analyzed by TLC to confirm consumption of starting material **24a**. AcOH (5 µL) was added to quench the reaction. The reaction was then loaded directly onto a preparatory TLC plate and eluted with 60% EtOAc in hexanes. Bands containing both diastereomers were collected and concentrated yielding **40b** (33% yield, 0.0040 mmol, 1.8 mg) and **40a** (1.3 mg, 0.0029 mmol, 24% yield) both as white solids. Stereochemistry of Grignard addition was assigned by the presence of an NOE signal in **40a** between the alcohol proton and the benzylic proton which is not present in **40b.** These NOESY spectrum were recorded in deuterated-acetone, see NMR section below.

40b

^{1}H NMR (600 MHz, Acetone) δ 7.28 (t, *J* = 7.6 Hz, 2H), 7.21 (d, *J* = 8.1 Hz, 2H), 7.16 (t, *J* = 7.3 Hz, 1H), 5.22 (dd, *J* = 12.4, 7.4 Hz, 1H), 4.56 (s, OH), 3.69 (s, 3H), 3.06 (s, 1H), 3.03 (dd, *J* = 13.3, 4.0 Hz, 1H), 2.98 (tt, *J* = 12.7, 3.5 Hz, 1H), 2.54 (d, *J* = 10.9 Hz, 1H), 2.37 (ddd, *J* = 13.0, 7.4, 4.0 Hz, 1H), 2.21 – 2.12 (m, 2H), 2.09-2.06 (m, 2H) 2.03-1.98 (m, 1H), 2.01 (s, 3H) 1.79 (t, *J* = 12.6 Hz, 1H), 1.75 (dt, *J* = 12.9, 3.2 Hz, 1H), 1.61 (td, *J* = 13.3, 3.6 Hz, 1H), 1.57 – 1.47 (m, 1H), 1.32 (td, *J* = 11.6, 3.8 Hz, 1H), 0.96 (q, *J* = 12.3 Hz, 1H), 0.84 (s, 3H).

^{1}H NMR (600 MHz, $CDCl_3$) δ 7.28-7.24 (m, 2H) 7.17 (dd, *J* = 8.2, 6.6 Hz, 3H), 5.13 (dd, *J* = 12.0, 7.8 Hz, 1H), 3.71 (s, 3H), 3.02 (tt, *J* = 12.4, 3.3, 1H), 2.77 (dd, *J* = 13.1, 4.3 Hz, 1H), 2.62 (s, 1H), 2.38 – 2.23 (m, 3H), 2.19 (t, *J* = 9.0 Hz, 1H), 2.16-2.07 (m, 3H), 2.12 (s, 3H) 1.97 (m, 1H), 1.81 – 1.74 (m, 2H), 1.55-1.50 (m, 2H), 1.26 (m, 1H), 0.90 (s, 3H).

^{13}C NMR (151 MHz, $CDCl_3$) δ 202.75, 172.14, 170.03, 144.94, 128.59, 127.07, 126.48, 84.82, 75.35, 75.14, 72.10, 60.57, 52.35, 51.96, 50.73, 48.08, 42.93, 40.62, 38.24, 38.05, 32.91, 31.90, 20.77, 20.57, 14.05.

R_f = 0.62 60% EtOAc in Hex, (UV, Brown - anisaldehyde)

$[\alpha]_D^{29.3}$ = -70.4 (c = 0.1, $CHCl_3$)

HRMS: [M+H]+ calcd. 453.2277, found 453.2285

40a

^{1}H NMR (600 MHz, Acetone) δ 7.33 – 7.23 (m, 2H), 7.23 – 7.18 (m, 2H), 7.15 (ddt, J = 8.6, 6.9, 1.4 Hz, 1H), 5.21 (ddd, J = 12.4, 7.4, 0.9 Hz, 1H), 4.40 (s, OH), 3.69 (s, 3H), 3.08 (tt, J = 12.6, 3.5 Hz, 1H), 3.02 (dd, J = 13.3, 4.0 Hz, 1H), 2.88 (d, J = 0.8 Hz, 1H), 2.51 (dd, J = 11.2, 0.9 Hz, 1H), 2.36 (ddd, J = 13.0, 7.4, 4.0 Hz, 1H), 2.27 – 2.11 (m, 3H), 2.01 (s, 3H), 2.01 – 1.97 (m, 2H), 1.91 (t, J = 13.1 Hz, 1H), 1.83 – 1.71 (m, 1H), 1.71 – 1.60 (m, 2H), 1.37 (td, J = 11.6, 3.6 Hz, 1H), 1.02 (td, J = 12.5, 11.1 Hz, 1H), 0.84 (s, 3H).

^{1}H NMR (600 MHz, $CDCl_3$) δ 7.28 – 7.22 (m, 2H), 7.16 (dd, J = 8.2, 6.6 Hz, 3H), 5.13 (dd, J = 12.2, 8.2 Hz, 1H), 3.71 (s, 3H), 3.10 – 3.02 (m, 1H), 2.77 (dd, J = 13.0, 4.3 Hz, 1H), 2.46 (s, 1H), 2.39 – 2.15 (m, 5H), 2.11 (s, 3H), 2.11-2.06 (m, 1H), 2.03 (dd, J = 12.8, 3.3 Hz, 1H), 1.91 (t, J = 13.4 Hz, 1H), 1.79 – 1.76 (m, 1H), 1.63 – 1.49 (m, 2H), 1.35 – 1.28 (m, 1H), 0.97 – 0.91 (q, J = 12.4 1H), 0.89 (s, 3H).

^{13}C NMR (151 MHz, $CDCl_3$) δ 202.62, 172.15, 170.06, 145.28, 128.55, 127.12, 126.41, 87.33, 75.37, 72.24, 69.71, 60.92, 52.44, 51.91, 49.30, 47.71, 42.99, 38.66, 38.08, 37.95, 31.86, 30.45, 21.23, 20.77, 14.04.

R_f = 0.72 60% EtOAc in Hex, (UV, Brown - anisaldehyde)

$[\alpha]_D^{29.6}$ = -28.2 (c = 0.1, $CHCl_3$)

HRMS: [M+H]+ calcd. 453.2277, found 453.2283

Analog **37**

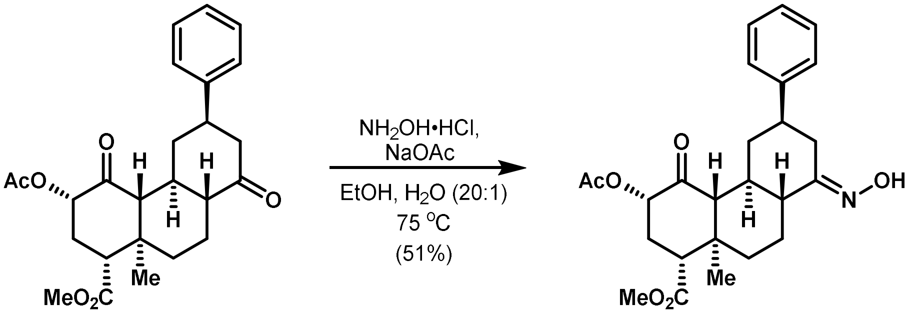

24a (5 mg, 0.012 mmol, 1 equiv.) was dissolved in EtOH (1 mL) and H_2O (50 µL). NaOAc (10 mg, 0.12 mmol, 10 equiv.) and NH_2OH•HCl (10 mg, 0.14 mmol, 12 equiv.) was added. This mixture was stirred at 75 °C for 1 hour at which point it was analyzed by TLC to confirm consumption of **24a**. The mixture was then concentrated under a constant flow of nitrogen. The crude material was suspended in minimal amount of DCM and loaded directly onto a silica gel preparatory TLC plate. Elution with 60% EtOAc in hexanes followed by collection of the product containing band afforded **37** (2.7 mg, 0.0061 mmol, 51% yield) as a white film.

Alternatively after the ethanol concentration EtOAc (1mL) and H_2O (1 mL) was added to the crude reaction and the layers were mixed. The organic layer was separated and the aqueous layer was extracted 3 more times with EtOAc (3 x 1 mL). The combined organic layers were washed with brine (1 mL) and dried over Na_2SO_4, filtered and concentrated to yield crude **37** which was used in subsequent transformations.

^{1}H NMR (600 MHz, C_6D_6) δ 7.13 – 7.07 (m, 2H), 7.09 – 6.97 (m, 3H), 5.01 – 4.95 (m, 1H), 3.85 (ddd, J = 13.5, 4.1, 1.9 Hz, 1H), 3.29 (s, 3H), 2.68 (tt, J = 12.8, 3.8 Hz, 1H), 2.43 – 2.24 (m, 3H), 2.05 (ddd, J = 12.4, 7.3, 2.8 Hz, 1H), 1.94 – 1.81 (m, 2H), 1.74 (s, 3H), 1.72 – 1.67 (m, 1H), 1.68 – 1.58 (m, 3H), 1.45 – 1.37 (m, 1H), 1.24 (td, J = 13.4, 3.7 Hz, 1H), 0.85 (td, J = 12.6, 11.0 Hz, 1H), 0.81 (s, 3H).

^{13}C NMR (151 MHz, C_6D_6) δ 201.92, 171.66, 169.32, 160.06, 145.24, 128.81, 126.98, 126.63, 75.35, 60.31, 52.19, 51.17, 45.77, 42.70, 42.65, 38.30, 38.10, 37.28, 31.91, 31.86, 21.79, 20.19, 13.78.

R_f = 0.67 60% EtOAc in Hex, (UV, Red - anisaldehyde)

$[\alpha]_D^{29.6}$ = -15.5 (c = 0.1, $CHCl_3$)

HRMS: [M+H]+ calcd. 442.2230, found 442.2240

2D NMR: HSQC, NOESY

Analog **35**

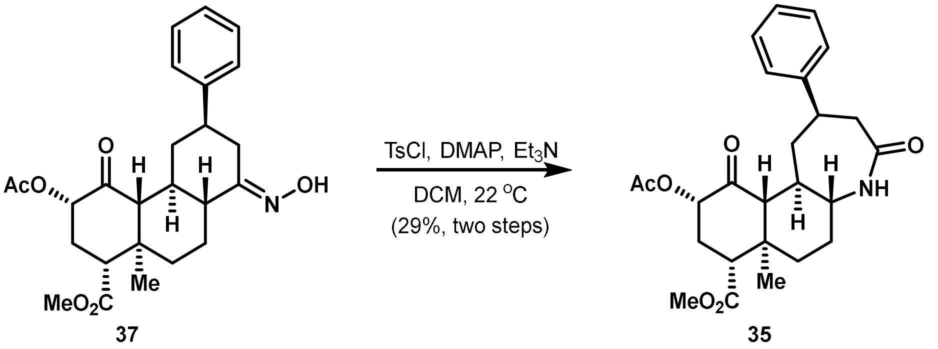

Crude **37** (see S64 for preparation) was dissolved in DCM (500 µL) and TsCl (5 mg, 0.026, 2.17 equiv.), Et_3N (5 µL, 0.036, 3 equiv.) and DMAP (1 mg, 0.008 mmol, 0.7 equiv.) was added to the vessel. The mixture was stirred for 4 hours at 22 °C and the DCM was concentrated under a stream of nitrogen. The reaction was loaded directly onto a preparatory TLC plate eluting with 60% EtOAc in hexanes. The band containing the product was collected and concentrated to yield **35** as a white solid (1.6 mg, 0.0035 mmol, 29% yield, over two steps).

^{1}H NMR (600 MHz, $CDCl_3$) δ 7.28-7.23 (m, 2H), 7.20 – 7.14 (m, 1H), 7.13 – 7.09 (m, 2H), 5.44 (s, 1H), 5.12 (dd, J = 12.1, 7.7 Hz, 1H), 3.71 (s, 3H), 3.30 (tt, J = 10.1, 5.0 Hz, 1H), 3.00 (t, J = 11.8 Hz, 1H), 2.83 (dd, J = 13.6, 12.0 Hz, 1H), 2.73 (dd, J = 13.3, 4.1 Hz, 1H), 2.59 (d, J = 13.6 Hz, 1H), 2.39 (ddd, J = 13.3, 7.6, 4.1 Hz, 1H), 2.28 (q, J = 13.1 Hz, 1H), 2.20 – 2.12 (m, 3H), 2.10 (s, 3H), 1.98 – 1.89 (m, 1H), 1.78 (dt, J = 13.5, 3.6 Hz, 1H), 1.62-1.57 (m, 1H), 1.50 – 1.41 (m, 1H), 1.28-1.24 (m, 1H), 0.89 (s, 3H).

^{13}C NMR (151 MHz, $CDCl_3$) δ 201.82, 176.45, 171.65, 169.89, 146.27, 128.89, 126.82, 126.30, 75.19, 60.10, 55.25, 52.10, 51.91, 45.66, 43.60, 42.06, 40.45, 37.15, 36.60, 31.99, 27.49, 20.75, 13.99.

R_f = 0.73 60% EtOAc in Hex, (UV, light orange - anisaldehyde)

$[\alpha]_D^{25.0}$ = -292.2 (c = 0.1, $CHCl_3$)

HRMS: [M+H]+ calcd. 442.2230, found 442.2234

2D NMR: COSY, HSQC

Analog **39**

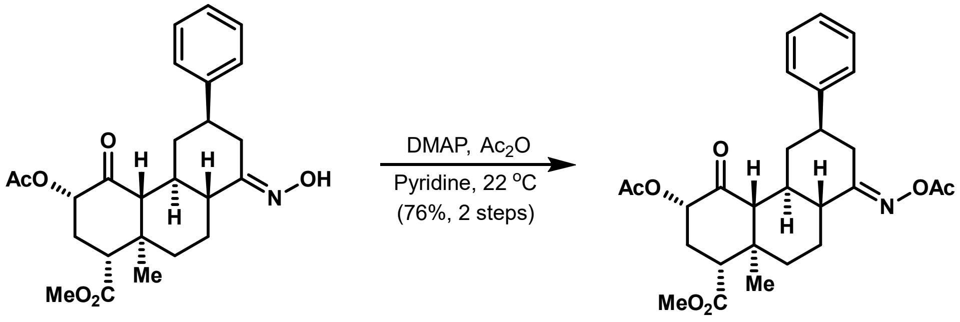

37 **39**

Crude **37** (see analog **37** for preparation) and DMAP (0.8 mg, 0.0065 mmol, 0.05 equiv.) were dissolved in pyridine (0.14 mL) under argon. To this solution, Ac_2O (3.9 µL, 0.041 mmol, 3 equiv.) was added neat. The reaction was stirred at 22 °C until complete consumption of the starting material (approximately 3 hours) as indicated by TLC. Aqueous $NaHCO_3$ solution (1 mL) was then added and the product was extracted with EtOAc (3 x 1 mL). The combined organic layers were washed with brine (1 mL), dried over Na_2SO_4 and concentrated to give a crude mixture. The product was then purified from this crude by preparative TLC, affording the **39** as a white solid (4.1 mg, 0.0085 mmol, 76%, 2 steps).

^{1}H NMR (600 MHz, $CDCl_3$) δ 7.33 – 7.27 (m, 2H), 7.24 – 7.15 (m, 3H), 5.14 (ddd, *J* = 12.2, 7.9, 0.8 Hz, 1H), 3.72 (s, 3H), 3.49 (ddd, *J* = 13.7, 4.0, 1.8 Hz, 1H), 2.85 (tt, *J* = 12.8, 3.8 Hz, 1H), 2.79 (dd, *J* = 13.0, 4.3 Hz, 1H), 2.39 – 2.22 (m, 4H), 2.14 (s, 3H), 2.12 (s, 3H), 2.09 – 1.99 (m, 3H), 1.96 (t, *J* = 13.5 Hz, 1H), 1.77 (dq, *J* = 14.1, 4.1, 3.4 Hz, 2H), 1.59 – 1.51 (m, 1H), 1.17 (td, *J* = 12.7, 10.2 Hz, 1H), 0.89 (s, 3H).

^{13}C NMR (151 MHz, $CDCl_3$) δ 202.30, 171.92, 170.06, 169.09, 168.07, 144.09, 128.81, 127.02, 126.83, 75.28, 60.96, 52.36, 52.04, 46.73, 43.14, 42.91, 38.22, 37.99, 37.21, 34.21, 31.76, 21.14, 20.73, 19.93, 13.97.

R_f = 0.71 60% EtOAc in Hex, (UV, light orange - anisaldehyde)

$[\alpha]_D^{25.0}$ = -9.2 (c = 0.1, $CHCl_3$)

HRMS: [M+H]+ calcd. 484.2335, found 484.2324

2D NMR: NOESY

Analog **38**

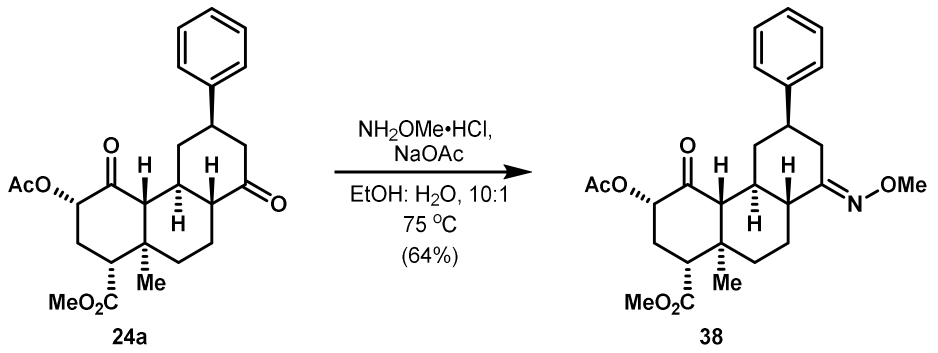

24a (5 mg, 0.012 mmol, 1 equiv.) was dissolved in EtOH (1 mL) and H_2O (0.1 mL). 10 mg of NaOAc (0.12 mmol, 10 equiv.) and 12 mg of NH_2OMe•HCl (0.14 mmol, 12 equiv.) were added. This mixture was stirred at 75 °C for 1 hour at which point it was analyzed by TLC to confirm consumption of the starting material **24a**. The mixture was then concentrated under a constant flow of nitrogen. The crude material was suspended in minimal amount of DCM and loaded directly onto a silica gel preparatory TLC plate. Elution with 50% EtOAc in hexanes followed by collection of the product containing band afforded **38** as a white film (3.5 mg, 0.0077 mmol, 64% yield).

^{1}H NMR (600 MHz, $CDCl_3$) δ 7.29 – 7.23 (m, 2H), 7.20 – 7.15 (m, 3H), 5.14 (dd, *J* = 12.2, 7.6 Hz, 1H), 3.82 (s, 3H), 3.72 (s, 3H), 3.56 – 3.49 (m, 1H), 2.79 (dq, *J* = 12.7, 4.1 Hz, 2H), 2.38 – 2.25 (m, 3H), 2.22 – 2.17 (m, 1H), 2.12 (s, 3H), 1.96 (qd, *J* = 11.0, 2.8 Hz, 1H), 1.93 – 1.82 (m, 2H), 1.78 – 1.71 (m, 2H), 1.63 (tdd, *J* = 15.8, 12.3, 3.9 Hz, 1H), 1.57 – 1.49 (m, 1H), 1.13 (td, *J* = 12.5, 10.8 Hz, 1H), 0.89 (s, 3H).

^{13}C NMR (151 MHz, $CDCl_3$) δ 202.56, 172.10, 170.03, 159.46, 144.95, 128.59, 126.87, 126.60, 75.32, 61.39, 61.01, 52.43, 51.96, 46.07, 42.98, 42.92, 38.28, 38.22, 37.20, 32.60, 31.81, 21.38, 20.75, 14.01.

R_f = 0.80 60% EtOAc in Hex, (UV, light orange - anisaldehyde)

$[\alpha]_D^{25.0}$ = -25.0 (c = 0.1, $CHCl_3$)

HRMS: [M+H]+ calcd. 456.2386, found 456.2392

Analog **20**

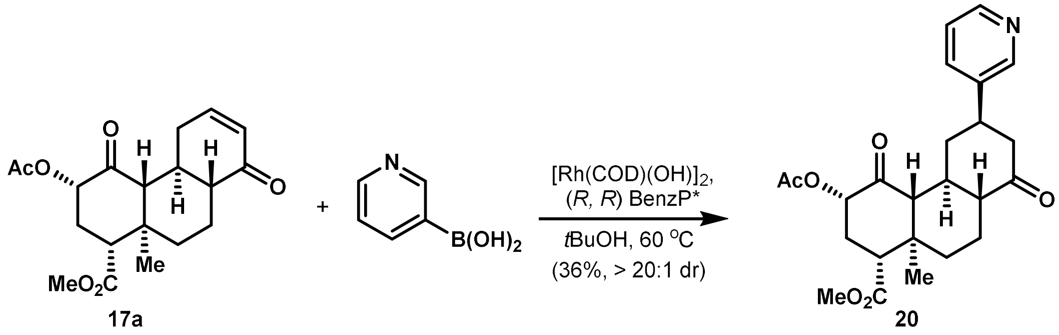

To an 8 mL reaction tube **17a** (9 mg, 0.025 mmol, 1 equiv.), $[Rh(COD)(OH)]_2$ (5.9 mg, 0.013 mmol, 0.5 equiv.), *(R, R)* BenzP* (9 mg, 0.0324 mmol , 1.6 equiv.), and 3-pyridyl boronic acid (4.5 mg, 0.029 mmol, 1.2 equiv.) were dissolved in *t*BuOH (750 µL) and submerged in a 60 °C oil bath. This mixture was stirred for 1 hour at which point it was analyzed by TLC to confirm consumption of **17a**. The *t*BuOH was then removed from the reaction via a constant stream of nitrogen in the 60 °C oil bath and the reaction redissolved in EtOAc. The reaction mixtuure was then filtered through a silica plug, concentrated and purified by preparatory TLC eluting with 100% EtOAc in hexanes. **20** was isolated as a white solid (3.8 mg, 0.0089 mmol, 36% yield).

^{1}H NMR (600 MHz, $CDCl_3$) δ 8.55 – 8.49 (m, 2H), 7.68 – 7.63 (m, 1H), 7.37 (t, *J* = 6.6 Hz, 1H), 5.15 (dd, *J* = 12.4, 7.5 Hz, 1H), 3.72 (s, 3H), 3.15 (t, *J* = 13.2 Hz, 1H), 2.80 (dd, *J* = 13.1, 4.0, 1H), 2.64 (dd, *J* = 13.3, 4.8 Hz, 1H), 2.54 (t, *J* = 13.4 Hz, 1H), 2.41-2.25 (m, 4H), 2.13 (s, 3H), 2.17 – 2.06 (m, 2H), 1.84 (d, *J* = 12.7 Hz, 1H), 1.78 (d, *J* = 9.9 Hz, 1H), 1.61 – 1.49 (m, 2H), 1.45 (q, *J* = 11.7 Hz, 1H), 0.87 (s, 3H).

^{13}C NMR (151 MHz, $CDCl_3$) δ 208.60, 202.46, 171.80, 170.10, 146.90, 146.67, 139.99, 136.22, 124.34, 75.30, 60.79, 52.96, 52.26, 52.07, 48.27, 42.88, 41.65, 37.41, 37.21, 36.70, 31.65, 20.71, 19.78, 13.78.

R_f: 0.44, 100% EtOAc, UV, Red (Dragendorf)

$[\alpha]_D^{23.5}$ = -8.5 (c = 0.1, EtOAc)

HRMS: [M+H]+ calcd. 428.2073, found 428.2085

Analog **21**

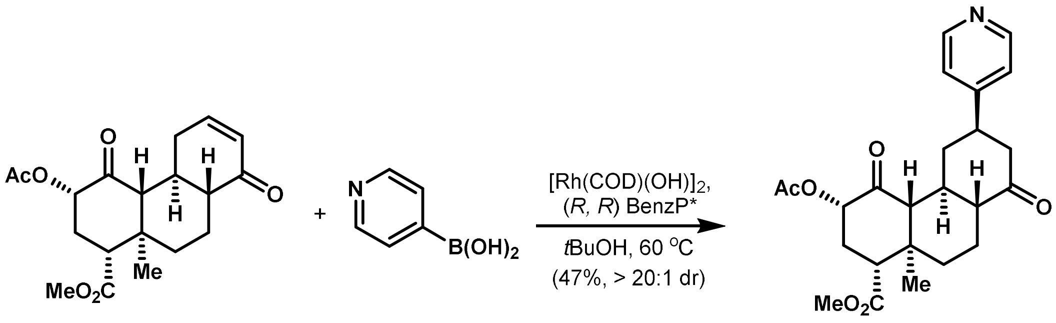

17a **21**

See General Procedure C

^{1}H NMR (600 MHz, $CDCl_3$) δ 8.53 (d, J = 4.9 Hz, 2H), 7.13 (d, J = 5.0 Hz, 2H), 5.15 (dd, J = 12.3, 7.6 Hz, 1H), 3.72 (s, 3H), 3.07 (tt, J = 13.0, 3.8 Hz, 1H), 2.79 (dd, J = 13.2, 4.1 Hz, 1H), 2.67 – 2.58 (m, 1H), 2.49 (t, J = 13.5 Hz, 1H), 2.41 – 2.25 (m, 4H), 2.14 (s, 3H), 2.12 – 2.01 (m, 2H), 1.83 (d, J = 12.7 Hz, 1H), 1.81 – 1.71 (m, 1H), 1.63 – 1.47 (m, 2H), 1.40 (q, J = 11.5 Hz, 1H), 0.86 (s, 3H).

^{13}C NMR (151 MHz, $CDCl_3$) δ 208.73, 202.41, 171.79, 170.11, 152.74, 149.92, 122.22, 75.28, 60.87, 53.05, 52.31, 52.07, 47.83, 43.23, 42.88, 37.44, 36.69, 36.67, 31.68, 20.73, 19.79, 13.79.

R_f: 0.44, 100% EtOAc, UV, Red (Dragendorf)

$[\alpha]_D^{23.5}$ = -8.6 (c = 0.1, EtOAc)

HRMS: [M+H]+ calcd. 428.2073, found 428.2076

Analog **23**

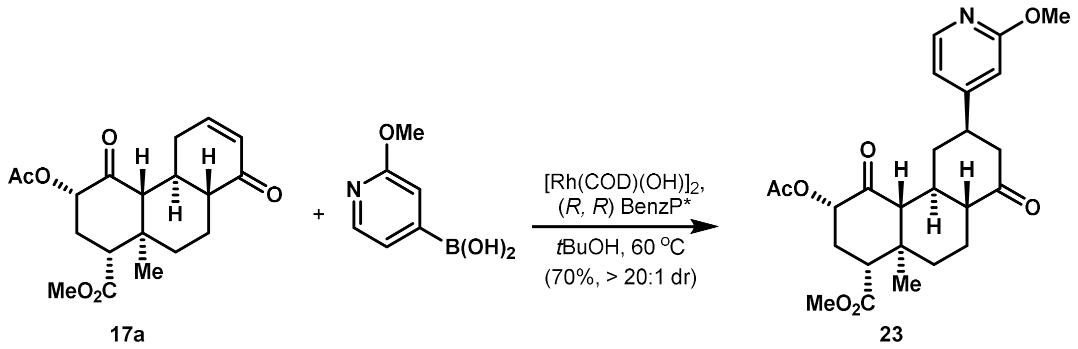

General Procedure C was followed using 2-methoxy 4-boronic acid pyridine (2.5 mg, 0.016 mmol, 1.2 equiv.). The reaction was purified on preparatory TLC eluting with 100% Et_2O. **23** was isolated as a white solid (4.5 mg, 0.0098 mmol, 70% yield).

^{1}H NMR (600 MHz, $CDCl_3$) δ 8.09 – 8.05 (m, 1H), 6.70 (dd, *J* = 5.4, 1.6 Hz, 1H), 6.54 (d, *J* = 1.5 Hz, 1H), 5.15 (dd, *J* = 12.1, 7.9 Hz, 1H), 3.92 (s, 3H), 3.72 (s, 3H), 3.01 (tt, *J* = 12.9, 3.9 Hz, 1H), 2.79 (dd, *J* = 13.2, 4.1 Hz, 1H), 2.65 – 2.56 (m, 1H), 2.47 (t, *J* = 13.5 Hz, 1H), 2.41 – 2.24 (m, 4H), 2.14 (s, 3H), 2.06 (dd, *J* = 17.5, 7.5 Hz, 2H), 1.82 (d, *J* = 12.1 Hz, 1H), 1.79 – 1.74 (m, 1H), 1.56 – 1.47 (m, 2H), 1.36 (q, *J* = 11.6 Hz, 1H), 0.86 (s, 3H).

^{13}C NMR (151 MHz, $CDCl_3$) δ 209.03, 202.37, 171.82, 170.07, 164.79, 155.24, 147.23, 115.63, 108.78, 75.28, 60.89, 53.64, 53.06, 52.31, 52.06, 47.78, 43.18, 42.87, 37.47, 36.75, 36.67, 31.69, 20.73, 19.80, 13.80.

R_f: 0.41, 100% Et_2O, UV, Red (Dragendorf)

$[\alpha]_D^{25.4}$ = -4.5 (c = 0.1, EtOAc)

HRMS: [M+H]+ calcd. 458.2179, found 458.2186

Analog **25**

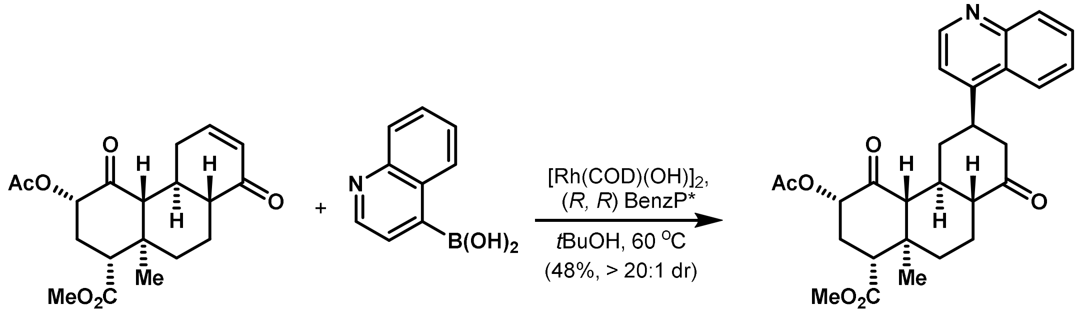

General Procedure C was followed using quinoline-4-boronic acid (2.8 mg, 0.016 mmol, 1.2 equiv.). The reaction was purified on preparatory TLC eluting with 100% EtOAc. **25** was isolated as a white solid (3.2 mg, 0.0067 mmol, 48% yield).

^{1}H NMR (600 MHz, $CDCl_3$) δ 8.85 (d, *J* = 4.5 Hz, 1H), 8.15 (d, *J* = 8.5 Hz, 1H), 8.07 (d, *J* = 8.5 Hz, 1H), 7.73 (t, *J* = 7.6 Hz, 1H), 7.62 (t, *J* = 7.9 Hz, 1H), 7.28-7.24(m, 1H) 5.17 (dd, *J* = 12.5, 7.6 Hz, 1H), 3.91 (t, *J* = 12.2 Hz, 1H), 3.73 (s, 3H), 2.82 (dd, *J* = 13.2, 3.8 Hz, 1H), 2.77 (ddd, *J* = 13.5, 4.1, 2.2 Hz, 1H), 2.59 (t, *J* = 13.5 Hz, 1H), 2.47 (dt, *J* = 13.1, 2.7 Hz, 1H), 2.43 – 2.21 (m, 4H), 2.19 (td, *J* = 11.7, 3.4 Hz, 1H), 2.10 (s, 3H), 1.88 (dt, *J* = 13.6, 3.1 Hz, 1H), 1.80 (dt, *J* = 13.2, 2.7 Hz, 1H), 1.70 – 1.48 (m, 3H), 0.91 (s, 3H).

^{13}C NMR (151 MHz, $CDCl_3$) δ 208.86, 202.38, 171.82, 170.06, 149.98, 149.66, 148.15, 130.34, 129.70, 127.28, 126.45, 122.73, 117.35, 75.22, 60.84, 53.42, 52.31, 52.08, 47.96, 42.92, 38.34, 37.46, 36.97, 36.48, 31.72, 20.71, 19.90, 13.83.

R_f: 0.22, 100% EtOAc, UV, Red (Dragendorf)

$[\alpha]_D^{23.5}$ = -51.1 (c = 0.1, EtOAc)

HRMS: [M+H]+ calcd. 478.2230, found 478.2230

Analog **25**

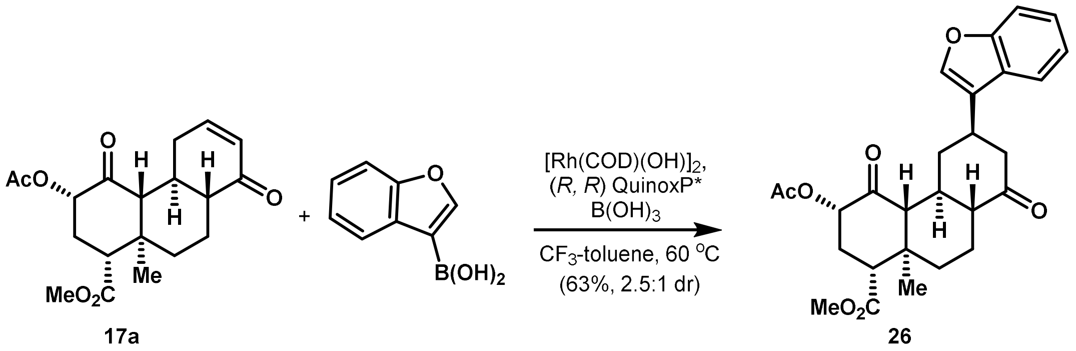

General Procedure B was followed using 3-benzofuran boronic acid (4.6 mg, 0.028 mmol, 2.7 equiv.). The reaction was purified on preparatory TLC eluting with 60% EtOAc in hexanes. **25** was isolated as a white solid (4.1 mg, 0.0088 mmol, 63% yield).

^{1}H NMR (600 MHz, $CDCl_3$) δ 7.58 (ddd, *J* = 7.7, 1.4, 0.7 Hz, 1H), 7.46 (dt, *J* = 8.2, 0.9 Hz, 1H), 7.38 (d, *J* = 0.9 Hz, 1H), 7.29 (ddd, *J* = 8.3, 7.1, 1.3 Hz, 1H), 7.24 (td, *J* = 7.5, 1.1 Hz, 1H), 5.18 (dd, *J* = 12.5, 7.3 Hz, 1H), 3.72 (s, 3H), 3.30 (ddd, *J* = 12.9, 9.0, 3.9 Hz, 1H), 2.87 – 2.77 (m, 2H), 2.63 – 2.49 (m, 2H), 2.43 – 2.34 (m, 2H), 2.35 – 2.26 (m, 1H), 2.21-2.13 (m, 2H) 2.14 (s, 3H), 1.89 – 1.82 (m, 1H), 1.82 – 1.74 (m, 1H), 1.67 – 1.44 (m, 3H), 0.89 (s, 3H).

^{13}C NMR (151 MHz, $CDCl_3$) δ 209.32, 202.38, 171.87, 170.03, 155.67, 140.31, 126.71, 124.58, 123.35, 122.65, 119.96, 111.85, 75.23, 60.90, 53.24, 52.33, 52.05, 47.53, 42.84, 37.50, 36.61, 36.22, 34.58, 31.77, 20.76, 19.86, 13.83.

R_f: 0.57, 60% EtOAc, (UV, pink – anisaldehyde)

$[\alpha]_D^{23.5}$ = -28.2 (c = 0.1, EtOAc)

HRMS: [M+H]+ calcd. 467.2070, found 467.2056

Analog **22b**

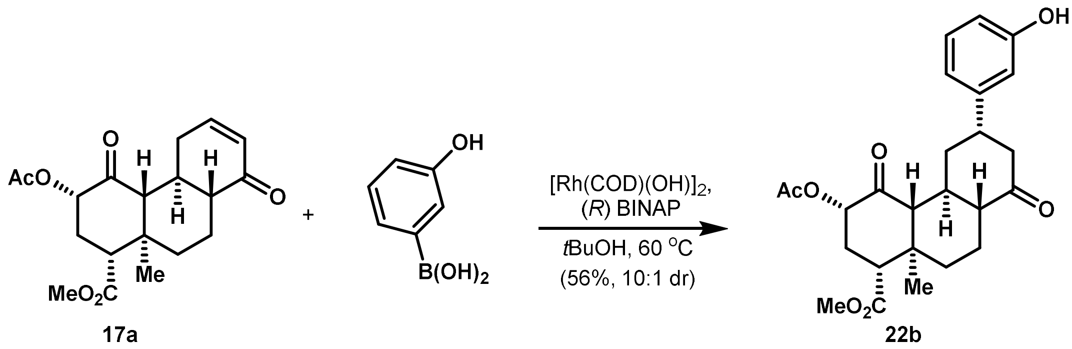

17a (5 mg, 0.014 mmol, 1 equiv.), $[Rh(COD)(OH)]_2$ (3.3 mg, 0.0073 mmol, 0.5 equiv.) and (*R*)-BINAP (13 mg, 0.0219 mmol, 2 equiv.) were added to a 8 mL reaction tube. These solids were dissolved in *t*BuOH (1 mL) and submerged in a 60 °C oil bath. The reaction mixture was stirred for 1 hour and at that point checked by TLC to confirm completion of the reaction. The *t*BuOH was then concentrated by a constant stream of nitrogen and the crude mixture was diluted with EtOAc and filtered through a silica plug. The mixture was purified by preparatory TLC eluting with 60% EtOAc in hexanes. **22b** was isolated as a white solid (3.5 mg, 0.0079 mmol, 56% yield).

^{1}H NMR (600 MHz, $CDCl_3$) δ 7.18 (t, *J* = 7.9 Hz, 1H), 6.83 (d, *J* = 7.1 Hz, 1H), 6.75 (t, *J* = 2.2 Hz, 1H), 6.69 (dd, *J* = 8.0, 2.5 Hz, 1H), 5.15 (dd, *J* = 12.4, 7.5 Hz, 1H), 3.69 (s, 3H), 3.49 (s, 1H), 2.85 (ddd, *J* = 15.2, 4.0, 1.8 Hz, 1H), 2.76 (dd, *J* = 13.3, 4.0 Hz, 1H), 2.68 (dd, *J* = 15.1, 6.5 Hz, 1H), 2.44 (d, *J* = 13.5 Hz, 1H), 2.36 – 2.29 (m, 2H), 2.24 (q, *J* = 13.3) 2.15 (s, 3H), 2.09 – 1.94 (m, 2H), 1.87 (dd, *J* = 14.3, 3.6 Hz, 1H), 1.70 (dt, *J* = 13.0, 3.4 Hz, 1H), 1.61 (ddd, *J* = 13.5, 10.6, 5.2 Hz, 1H), 1.48 (td, *J* = 13.4, 3.5 Hz, 1H), 1.44 – 1.34 (m, 1H), 0.72 (s, 3H).

^{13}C NMR (151 MHz, $CDCl_3$) δ 211.11, 202.09, 171.90, 170.18, 155.94, 144.99, 129.92, 119.86, 114.48, 113.57, 75.28, 61.45, 53.02, 52.34, 52.00, 44.90, 42.76, 39.02, 37.65, 35.66, 31.76, 31.63, 20.84, 19.91, 13.84.

R_f: 0.40, 60% EtOAc, UV, orange (anis)

$[\alpha]_D^{23.5}$ = -31.9 (c = 0.1, EtOAc)

HRMS: [M+H]+ calcd. 443.2070, found 443.2072

Analog **22a**

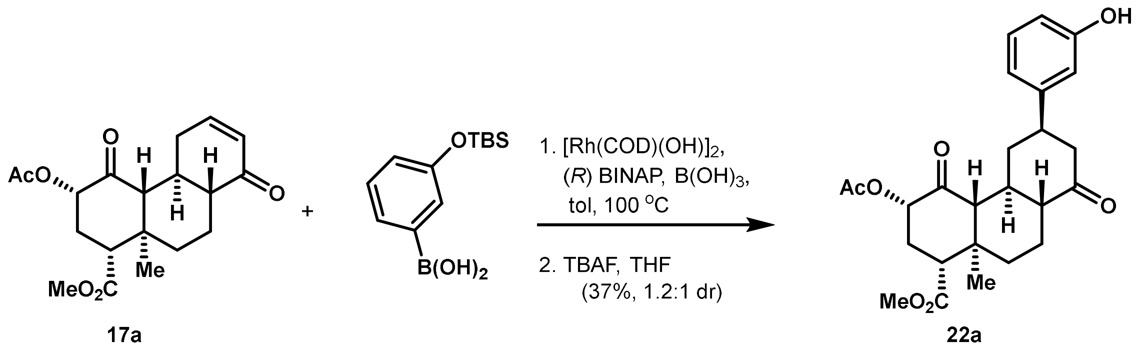

$[Rh(COD)(OH)]_2$ (2.9 mg, 0.0064 mmol, 0.25 equiv.) and (*R*)-BINAP (12 mg, 0.018 mmol, 0.7 equiv.) were dissolved in toluene (200 µL). These were stirred together at 22 °C for 15 minutes until all the Rh had dissolved and a bright red color was observed. Then aryl boronic acid (13 mg, 0.052 mmol, 2 equiv.) and $B(OH)_3$ (3 mg, 0.045 mmol, 1.75 equiv.) was added to the mixture. The solution was then submerged in a 100 °C oil bath and **17a** (9 mg, 0.0252 mmol, 1 equiv.) in toluene (200 µL) was added to the reaction. The reaction mixture was stirred for 1 hour and then removed from the oil bath and filtered through a silica plug. The crude reaction mixture was then dissolved in THF (200 µL) and 1 M TBAF in THF (5 µL, 0.05 mmol, 2 equiv.) was added at 0 °C and allowed to warm to 22 °C. The reaction was analyzed by TLC to confirm consumption of the TBS phenol and then loaded directly onto a preparatory TLC plate, eluting with 60% EtOAc in hexanes. The band containing the product was collected and concentrated to afford **23** as a white solid (4.1 mg, 0.0093 mmol, 37% yield over 2 steps).

^{1}H NMR (600 MHz, $CDCl_3$) δ 7.13 (t, *J* = 7.5 Hz, 1H), 6.73 – 6.63 (m, 3H), 5.15 (dd, *J* = 12.4, 7.7 Hz, 1H), 3.71 (s, 3H), 2.99 (t, *J* = 13.0 Hz, 1H), 2.79 (dd, *J* = 13.2, 4.0 Hz, 1H), 2.60 (dd, *J* = 12.6, 3.9 Hz, 1H), 2.49 (t, *J* = 13.5 Hz, 1H), 2.38 – 2.27 (m, 4H), 2.13 (s, 3H), 2.07 (d, *J* = 8.7 Hz, 2H), 1.99 – 1.92 (m, 1H), 1.89 (d, *J* = 14.9 Hz, 1H), 1.85 – 1.67 (m, 1H), 1.54 (q, *J* = 12.4, 11.5 Hz, 1H), 1.36 (q, *J* = 12.6, 11.6 Hz, 1H), 0.85 (d, *J* = 2.2 Hz, 3H).

^{13}C NMR (151 MHz, $CDCl_3$) δ 209.97, 202.46, 171.90, 170.15, 155.98, 145.74, 130.01, 119.09, 113.84, 113.73, 75.32, 60.99, 53.06, 52.34, 52.04, 48.89, 43.91, 42.87, 37.63, 37.54, 36.75, 31.71, 20.75, 19.85, 13.83.

R_f: 0.47, 60% EtOAc, UV, orange (anis)

$[\alpha]_D^{26.0}$ = -17.4 (c = 0.1, $CHCl_3$)

HRMS: [M+H]+ calcd. 443.2070, found 443.2068

Analog **19**

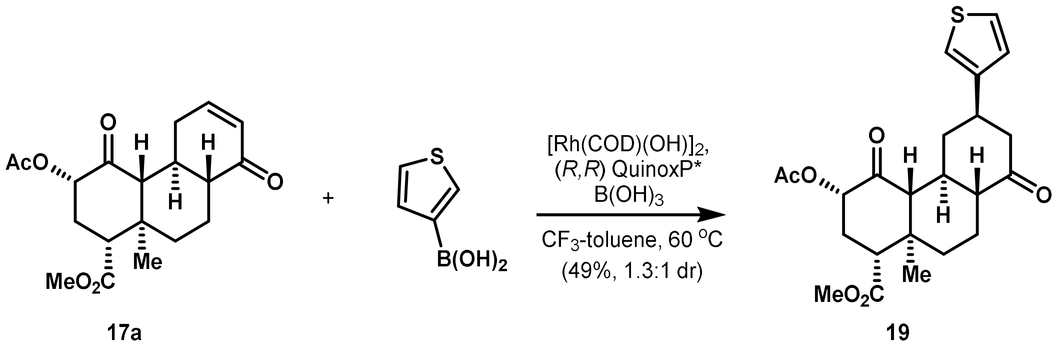

See General Procedure B

^{1}H NMR (600 MHz, $CDCl_3$) δ 7.25 (s, 1H) 6.95 (s, 1H), 6.95 (s, 1H), 5.17 (dd, J = 12.3, 7.6 Hz, 1H), 3.71 (s, 3H), 3.18 (tt, J = 12.8, 4.0 Hz, 1H), 2.79 (dd, J = 13.2, 4.2 Hz, 1H), 2.70 (ddd, J = 13.4, 4.3, 1.9 Hz, 1H), 2.47 (t, J = 13.4 Hz, 1H), 2.42 – 2.25 (m, 4H), 2.15 (s, 3H), 2.06 (ddd, J = 10.2, 6.9, 3.1 Hz, 2H), 1.81 (d, J = 12.2 Hz, 1H), 1.79 – 1.72 (m, 1H), 1.57 – 1.46 (m, 2H), 1.34 (qd, J = 12.5, 11.4, 6.8 Hz, 1H), 0.86 (s, 3H).

^{13}C NMR (151 MHz, $CDCl_3$) δ 209.71, 202.41, 171.89, 170.09, 145.00, 126.43, 126.07, 119.62, 75.29, 60.96, 53.04, 52.34, 52.03, 48.77, 42.84, 39.35, 37.61, 37.53, 36.52, 31.73, 20.76, 19.82, 13.83.

R_f: 0.58, 60% EtOAc, (UV, purple - anisaldehyde)

$[\alpha]_D^{26.5}$ = -26.6 (c = 0.1, $CHCl_3$)

HRMS: [M+H]+ calcd. 433.1685, found 433.1681

Analog **27**

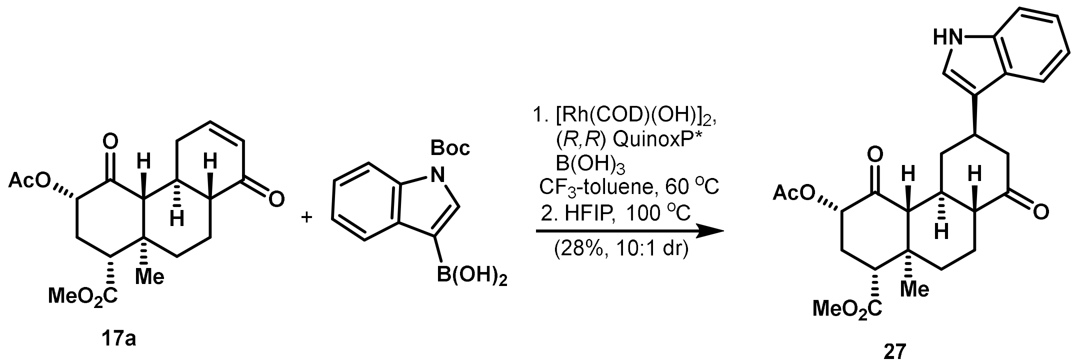

General Procedure B was followed using N-Boc indole-3-boronic acid (8.5 mg, 0.032 mmol, 3.1 equiv.). The crude material after silica plug filtration was dissolved in HFIP (500 μL) and heated to 100 °C in a microwave vial tube for 2 hours. This reaction mixture was then concentrated and the crude material loaded directly onto a preparatory TLC plate eluting with 60% EtOAc in hexanes. The band containing the product was collected and concentrated yielding **27** as a white solid (1.8 mg, 0.0039 mmol, 28% yield, 2 steps).

^{1}H NMR (600 MHz, $CDCl_3$) δ 7.96 (s, 1H), 7.64 (dt, *J* = 8.0, 1.0 Hz, 1H), 7.35 (dt, *J* = 8.1, 0.9 Hz, 1H), 7.19 (ddd, *J* = 8.2, 7.0, 1.1 Hz, 1H), 7.12 (ddd, *J* = 8.0, 7.0, 1.0 Hz, 1H), 6.95 (d, *J* = 2.1 Hz, 1H), 5.18 (dd, *J* = 12.8, 7.6 Hz, 1H), 3.72 (s, 3H), 3.40 (tt, *J* = 12.8, 3.8 Hz, 1H), 2.87 – 2.77 (m, 2H), 2.60 (t, *J* = 13.4 Hz, 1H), 2.53 (d, *J* = 12.9 Hz, 1H), 2.40 – 2.26 (m, 3H), 2.20 – 2.14 (m, 2H), 2.13 (s, 3H), 1.84 (dd, *J* = 13.7, 3.3 Hz, 1H), 1.80 – 1.74 (m, 1H), 1.64 – 1.48 (m, 3H), 0.89 (s, 3H).

^{13}C NMR (151 MHz, $CDCl_3$) δ 210.30, 202.34, 171.95, 170.04, 136.59, 126.14, 122.36, 119.98, 119.62, 119.62, 119.26, 111.41, 75.25, 61.06, 53.27, 52.40, 52.03, 48.66, 42.86, 37.61, 37.18, 36.75, 35.89, 31.80, 20.77, 19.93, 13.87.

R_f: 0.37, 60% EtOAc, UV, (brown - anisaldehyde)

$[\alpha]_D^{23.6}$ = -24.8 (c = 0.1, $CHCl_3$)

HRMS: [M+H]+ calcd. 466.2230, found 466.2232

Analog **33**

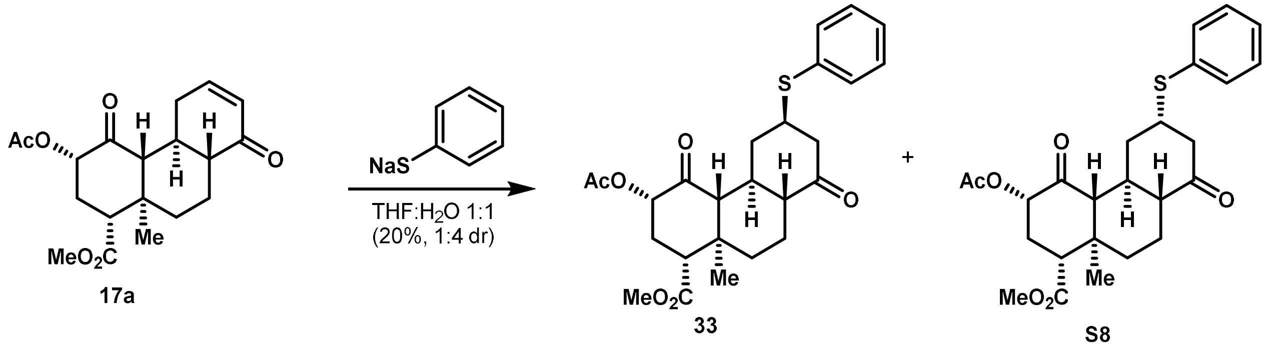

17a (5 mg, 0.014 mmol, 1 equiv.) was dissolved in of H_2O (100 µL) and THF (100 µL) in a 20 mL scintilation vial. To this solution was added sodium thiolate (3 mg, 0.023 mmol, 1.6 equiv.). The solution was stirred at 22 ºC for 1 hour at which point it was checked by TLC. If starting material was still present, an additional 3 mg of sodium thiolate was added and the reaction mixture was stirred for an additional hour. The reaction was then diluted with EtOAc and H_2O (1 mL each) and the layers separated. The aqueous layer was then extracted three times with EtOAc (3 x 1 mL) and the combined organic layer washed once with 1 M NaOH (1 mL). The organic layer was dried with Na_2SO_4 filtered and concentrated. Crude NMR indicated a 1:4 mixture of **33** : **S8** (Note 1). The crude mixture was purified on preparatory TLC eluting with 60% EtOAc in hexanes. Both diastereomers could be removed from the PTLC however **S8** slowly decomposed via reversion back to the starting enone through an E2 elimination (observed by NMR). Therefore, only the equitorial addition product **33** was fully characterized and evaluated for κOR activity. **33** was isolated as a white solid (1.3 mg, 0.0028 mmol, 20% yield).

Note 1: The diastereoselectivity likely proceeds through equilibration to the equitorial thiophenol, longer reactions times could have favored the **33** more.

^{1}H NMR (600 MHz, $CDCl_3$) δ 7.42 – 7.37 (m, 2H), 7.34 – 7.26 (m, 3H), 5.19 (dd, *J* = 12.2, 7.6 Hz, 1H), 3.70 (s, 3H), 3.31 (tt, *J* = 12.6, 4.1 Hz, 1H), 2.76 (dd, *J* = 13.3, 4.0 Hz, 1H), 2.68 (ddd, *J* = 13.7, 4.5, 2.0 Hz, 1H), 2.50 (d, *J* = 12.8 Hz, 1H), 2.40 – 2.23 (m, 4H), 2.17 (s, 3H), 2.01 – 1.92 (m, 2H), 1.78 – 1.67 (m, 2H), 1.50 – 1.41 (m, 2H), 1.27 – 1.19 (m, 1H), 0.81 (s, 3H).

^{13}C NMR (151 MHz, $CDCl_3$) δ 208.12, 202.17, 171.82, 170.03, 133.56, 132.65, 129.20, 128.03, 75.17, 60.66, 52.63, 52.22, 52.03, 48.61, 44.84, 42.80, 37.33, 36.78, 35.88, 31.72, 20.77, 19.69, 13.72.

R_f: 0.56, 60% EtOAc in hexanes (UV, orange – anisaldehyde)

$[\alpha]_D^{27.1}$ = -1.0 (c = 0.1, EtOAc)

HRMS: [M+H]+ calcd. 459.1841, found 459.1824

Table S2. Optimization of cycloadditions and Birch reductions to access **4**

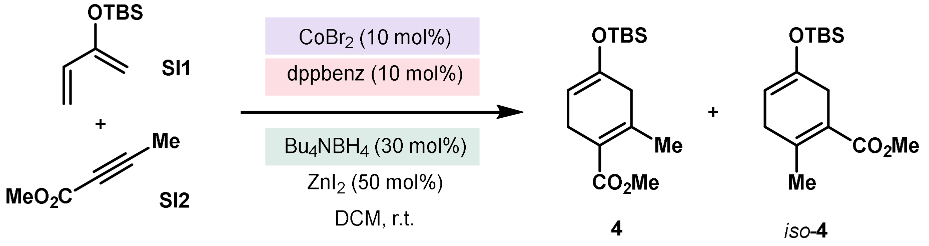

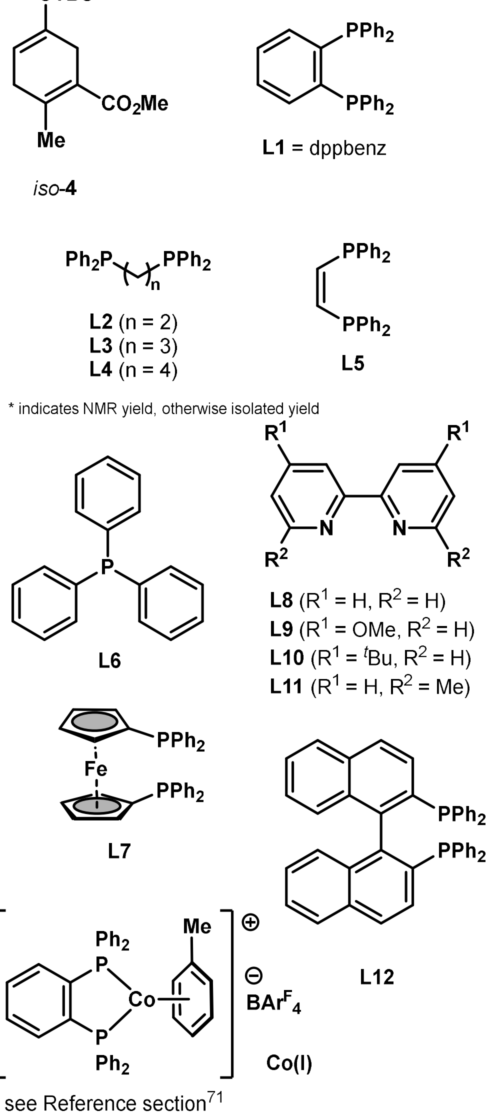

deviation from standard conditions	result (**4**/*iso*-**4**) [%]
none	59/12
**Co(I)** and DCM only	decomposition
$Co(acac)_2$	irreproducible on scale
**L2**	33/39*
**L3**	2/1*
**L4**	0/0*
**L5**	60/10*
**L6**[a]	0/0
**L7**[a]	0/0
**L8**[a]	0/0
**L9**[a]	0/0
**L10**[a]	45/1*
**L11**[a]	0/0
**L12**[a]	0/0
**L1** loading = 20 mol% not 10 mol%	28/5*
Zn	irreproducible on scale
diene-OTIPS instead of diene-OTBS	0/0
$[Rh(COD)Cl]_2$ + $AgBF_4$ instead of [Co]	0/0
140 °C neat instead of [Co]	0/0
$AlMe_3$ in PhMe instead of [Co]	0/0

[a] examined with Zn as the reductant

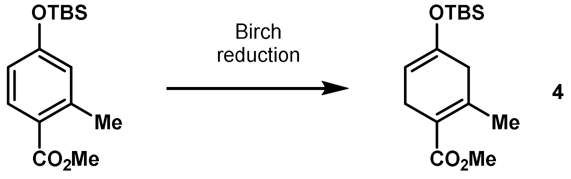

conditions	result
Na or Li, NH_3, THF[72]	decomposition
Na, 15-crown-5, ^{i}PrOH[28]	decomposition
LiBr, TPPA, DMU, THF, 10 mA, Mg(+)/Galvanized steel(–)[27]	decomposition
Li, $H_2N(CH_2)_2NH_2$, ^{t}BuOH, THF[26]	decomposition

see Reference section[72, 26, 27, 28,]

Table S3. Optimization of protection/acetylation of (+)-**6**

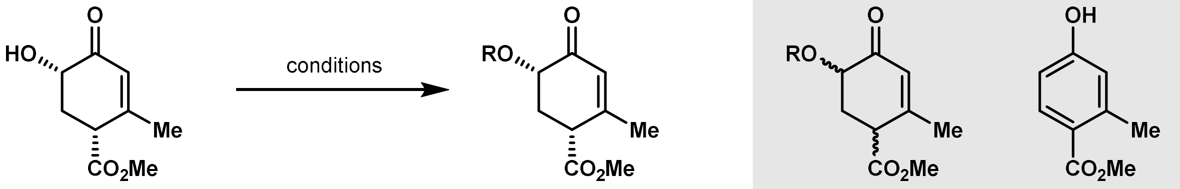

(+)-**6** (+)-**3** (R = Ac)

commonly observed products

R	conditions	result [%]
SiR_3	R_3Si-X (X = Cl, Br, OTf etc.), base	epimerization or aromatization
	TBSCl, Li_2S, MeCN	trace product
Me	MeI, base	aromatization
	MeI, AgOTf, DCM	poor conversion
allyl	[Pd], allyl bromide, base	aromatization
MOM	MOMCl, DIPEA, DCM	epimerization
	$CH(OMe)_2$, P_2O_5	aromatization
	**A**, AgOTf, NaOAc[73]	**60**
Bn	BnBr, NaH	aromatization
	BnCl, [Ni] cat. or [Cu] cat.	0
	BnBr, Ag_2O, DCM/hexanes[74]	**41**
	**B**, MgO[75]	0
	**C**, $BF_3{\cdot}Et_2O$ or H^+[76]	complex mixture
	**D**, Ph_3PAuCl, AgOTf, PhCl[77]	11
^{t}Bu	Boc_2O, $Mg(ClO_4)_2$ cat.[78]	**99**
Bz	BzCl, DMAP, MeCN	epimerization
	BzCl, CuO cat.	complex mixture
Ac	AcCl, DMAP, MeCN	epimerization
	Isopropenyl acetate, Otera's catalyst[79]	35
	Ac_2O, $Cu(OTf)_2$ cat., neat[37]	**99**

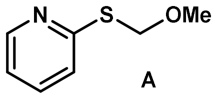

A

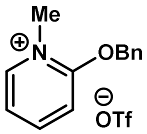

B

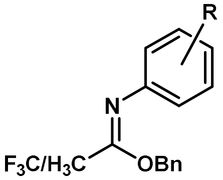

C

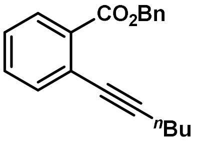

D

See Reference section[73-79, 37]

Table S4. Optimization of conjugate addition to access silyl enol ether **7**

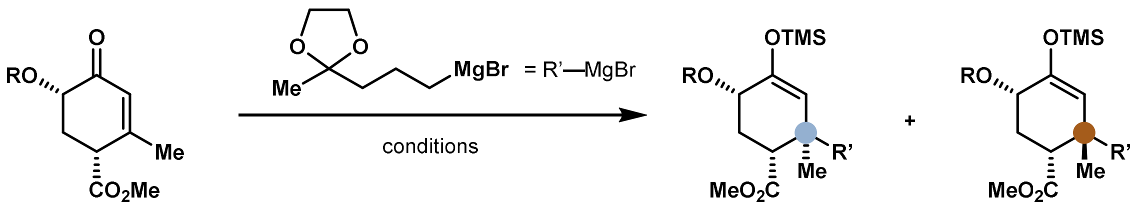

(+)-**3** (R = Ac) **7** (R = Ac) ***epi*-7** (R = Ac)

R	conditions	result (**7**/***epi*-7**) [%]
H	CuTC, TMSOTf, THF	trace product
Bn	CuTC, TMSOTf, THF	55/9
^{t}Bu	CuTC, TMSOTf, THF	trace product
MOM	CuTC, TMSOTf, THF	trace product
Ac	CuTC, TMSOTf, THF	dr = 1:1
	CuTC, TMSOTf, THF/PhMe	dr = 1:1
	CuTC, TMSOTf, THF/DMPU	trace product
	CuTC, TMSOTf, THF/Et_2O, –40 °C	decomposition
	CuTC, TMSOTf, THF/Et_2O, –60 °C, poor stirring	trace product
	CuTC, TMSOTf, THF/Et_2O, –60 °C; dilution→H_2O	decomposition
	CuTC, TMSOTf, THF/Et_2O, –60 °C; H_2O→dilution	**63/10**

Table S5. Optimization of Reformatsky reaction

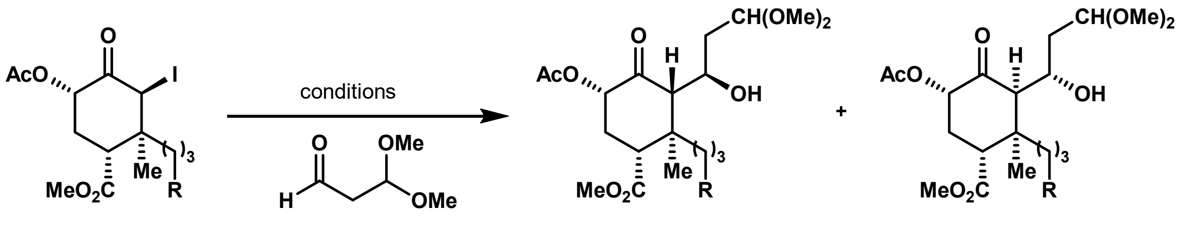

conditions	result (**14**/**13**) [%]
**SmI_2, THF, –78 °C**	**58/12**
SmI_2 (20 mol%), Mg (2 equiv.), THF, –78 °C	decomposition
**Et_2Zn, THF, 0 °C**	**8/69**
Et_2Zn, THF, –40 °C	0/0
Et_2Zn, $RhCl(PPh_3)_2$ (cat.), THF, 0 °C	decomposition
Et_2Zn, $NiCl_2(PPh_3)_2$ (cat.), THF, 0 °C	decomposition
Et_2Zn, $Ni(acac)_2$ + PPh_3 (cat.), THF, 0°C	4/39*
Et_2Zn, DMPU/HMPA, THF, 0 °C	0/0
Et_2Zn, $ZnBr_2$, 0°C	0/0
Et_2Zn, BOX ligands, THF, 0 °C	poor conversion
Et_2Zn, Et_2O or PhMe, 0°C	poor conversion
Et_2Zn, DCM, 0 °C	0/0
EtZnI, THF, 0 °C	0/0
^{i}PrMgCl·LiCl, toluene, –78 °C → 0 °C	0/0
Et_3B, toluene, –78 °C	0/0
In, TMSCl, THF, r.t	0/0
Li-naphthalenide, THF, –78 °C	0/0
Cp_2TiCl_2, Mn, THF, r.t	0/0

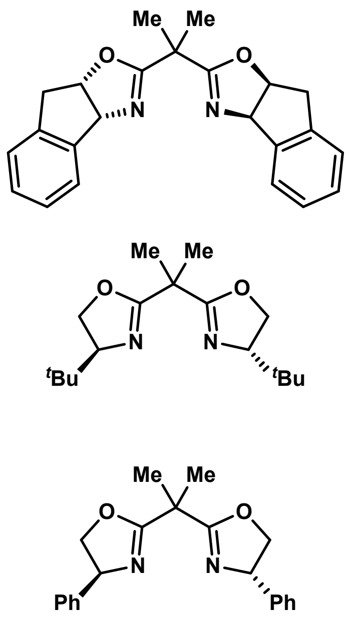

BOX ligands

Table S6. Optimization of deketalization of **14**

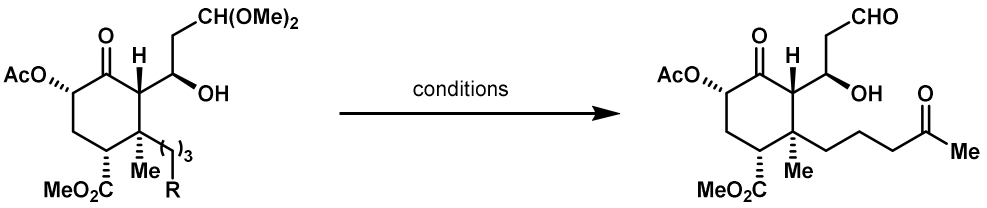

14 **15a**

conditions	result [%]
**$PdCl_2(MeCN)_2$ cat., acetone then activated charcoal**	**75**
$PdCl_2(MeCN)_2$ cat., acetone only	partial decomposition
PTSA, acetone	decomposition upon work-up
PTSA, PhMe/$PhCF_3$/PhF/PhCl, △	decomposition
1M HCl aq., THF	decomposition upon work-up
Amberlyst 15, acetone	irreproducible on scale
$Yb(OTf)_3$, MeCN	poor conversion
$LiBF_4$, MeCN	decomposition
TMSOTf, DCM	decomposition
(*S*)-TRIP, PhCl	ketal group intact

Table S7. Attempted optimization of amine/acid catalyzed Robinson annulation

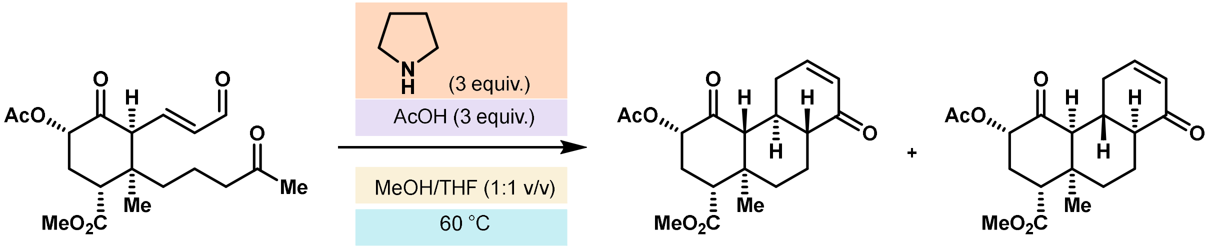

2b **17a** (19%) **17d**

deviation	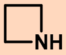	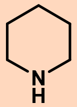	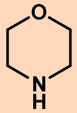	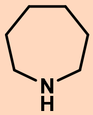	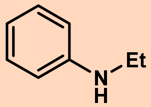	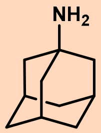	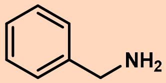
result (**a**/**b**) [%]	8/8	0/0	0/0	0/0	0/0	0/0	0/0

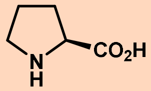	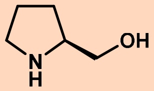	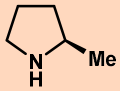	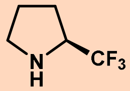	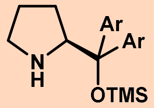 (Ar = C_6H_5 and 3,5-$(CF_3)_2C_6H_3$)	Et_3N
0/0	0/0	0/0	0/0	0/0	0/0

TFA	PTSA	HCl	BzOH
0/0	0/0	0/0	0/0

THF only	MeOH only
trace	11/0

25 °C	90 °C
0	decomposition

Table S8. Optimization of arylation of **17a**

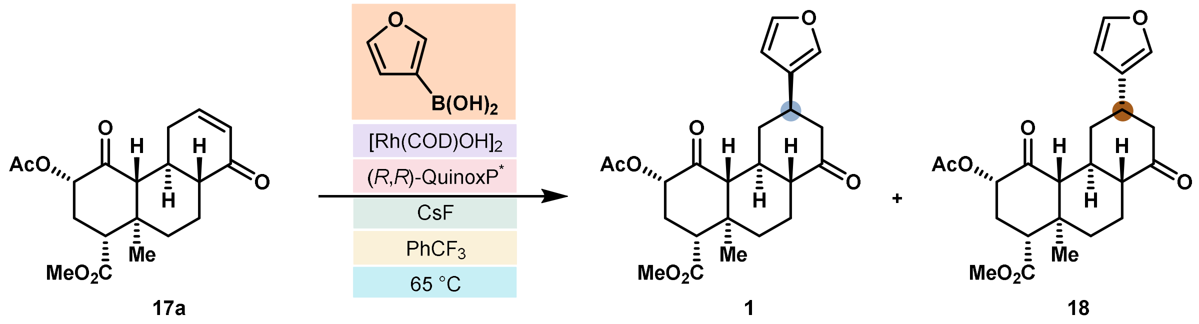

deviation	result (**1**/**18**) [%]
**none**	**51/12**
(***R***) or (***S***)-**L2**	0/0
(***R***) or (***S***)-**L3**	0/0
(***R***)-**L4**	0/0
(***R***)-**L5**	0/0
(***R***)-**L6**	0/0
(***S***)-**L7**	0/87
(***R***)-**L8**	0/84
(***R***)-**L9**	0/0
(***R***)-**L10**	0/0
(***R***)-**L11**	0/0
(***S***)-**L12**	0/0
(***R***) - **L13**	0/25
(***S***)-**L13**	0/41 (isolated)
(***R,R***)-**L14**	0/trace
(***R***)-**L15**	0/trace
(***R,R***)-**L16**	0/trace
(***R,R,S,S***)-**L17**	0/trace
(***R***)-**L18**	0/0
(***R,R***)-**L19**	0/17
(***S,S***)-**L20**	0/0

*Ligands screened with toluene, $B(OH)_3$, at 80 °C

deviation	result (**1**/**18**) [%]
$[Rh(COD)(OMe)]_2$	poor conversion
$[Rh(COD)Cl]_2$	incorrect d.r.
$[Rh(COD)(MeCN)_2](BF_4)$	incorrect d.r.
$[Rh(COE)Cl]_2$	0/0
$[Rh(C_2H_4)_2Cl]_2$	0/0
m-Xylene	poor conversion
PhMe	poor conversion
MeOH	0/0
1,4-dioxane	0/0
Furanyl-3-BF_3K	poor conversion
Furanyl-3-Bpin/Bneop	0/0
Furanyl-3-$B[(OCH_2)_2CCH_3]K$	0/0
$B(OH)_3$	45/15
$^{n}Bu_4NF/KF_2$/phthalimide	0/0
80 °C	poor conversion
100 °C	trace
CuBr·DMS, TMSCl, furanyl-3-MgBr	exclusively **18**

Table continues on next page

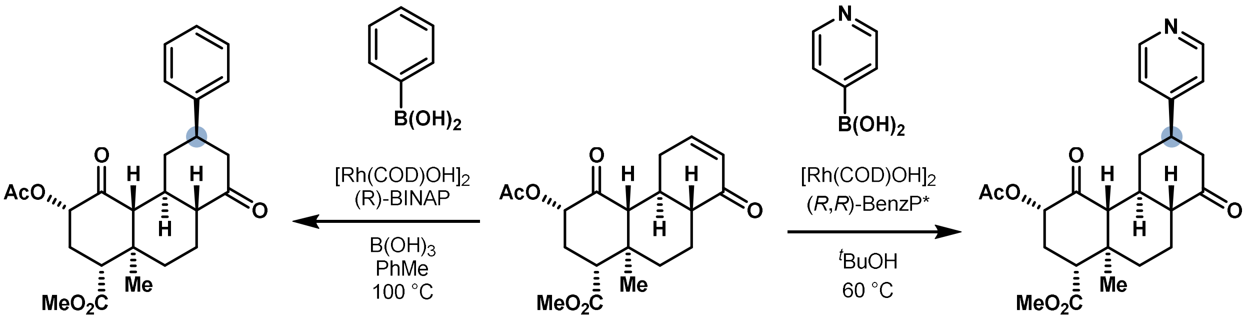

24a **17a** **21**

deviation	result (**24a**/***epi*-24a**) [%]
**none**	**39/4**
same conditions for **1**	0/0
same conditions for **21**	0/0
80 °C instead of 100 °C (without ligand)	0/59

deviation	result (**21**/***epi*-21**) [%]
**none**	**47/<1**[33]
same conditions for **1**	0/0
same conditions for **24a**	0/0
EtOH instead of ^{t}BuOH	decomposition

see Reference section[80] for use of alcoholic solvents

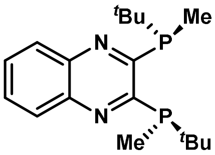

(***R,R***)-**L1**, QuinoxP*

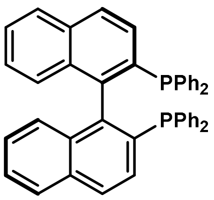

(***R***)-**L2**, BINAP

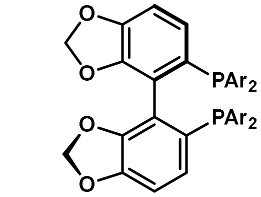

(***R***)-**L3**, SEGPHOS (Ar = C_6H_5)
(***R***)-**L4**, DTBM-SEGPHOS
(Ar = 3,5-(^{t}Bu)$_2$-4-(OMe)C_6H_2)

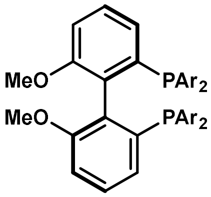

(***R***)-**L5**, MeO-BIPHEP (Ar = C_6H_5)
(***R***)-**L6**, DTBM-MeO-BIPHEP
(Ar = 3,5-(^{t}Bu)$_2$-4-(OMe)C_6H_2)

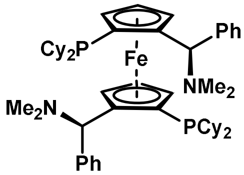

(***S,S***)-**L7**, MandyPhos
(Ar = 4-(OCH_3)-3,5-(CH_3)$_2C_6H_2$)

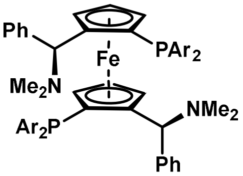

(***S,S***)-**L8**, MandyPhos
(Ar = 3,5-(CF_3)$_2C_6H_3$)

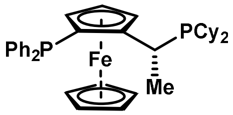

(***R***)-**L9**, JosiPhos

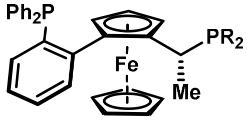

(***R***)-**L10**, WalPhos (R = 3,5-(CF_3)$_2C_6H_3$)
(***R***)-**L11**, WalPhos (R = Cy)

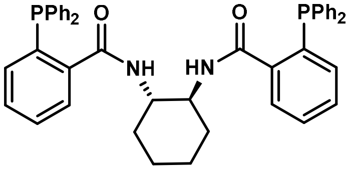

(***S,S***)-**L12**, DACH-phenyl Trost ligand

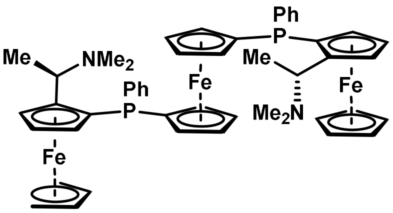

(***R***)-**L13**, TriferPhos

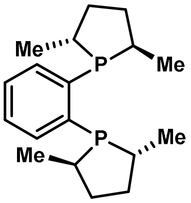

(***R,R***)-**L14**, Me-DuPhos

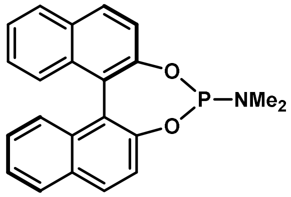

(***R***)-**L15**, MonoPhos

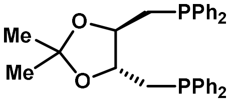

(***S,S***)-**L16**, DIOP

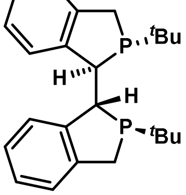

(***R,R,S,S***)-**L17**, DuanPhos

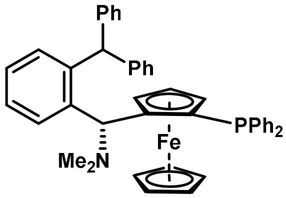

(***R***)-**L18**, TaniaPhos

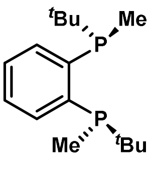

(***R,R***)-BenzP*

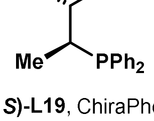

(***S, S***)-**L19**, ChiraPhos

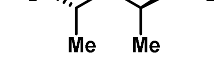

(***S,S***)-**L20**, BDPP

References

1. Atanasov, A. G. et al. Natural products in drug discovery: advances and opportunities. *Nat. Rev. Drug Discov.* **2021**, *20*, 200–216
2. Roth, B. L.; Baner, K.; Westkaemper, R.; Siebert, D.; Rice, K. C.; Steinberg, S.; Ernsberger, P.; Rothman, R. B. Salvinorin A: a potent naturally occurring nonnitrogenous κ opioid selective agonist. *Proc. Natl. Acad. Sci. USA* **2002**, *99*, 11934–11939
3. Hopkins, A.; Keserü, G.; Leeson, P.; Rees, D. C.; Reynolds, C. H. The role of ligand efficiency metrics in drug discovery. *Nat Rev Drug Discov.* **2014**, *13*, 105–121
4. J. M. Hooker, Y. Xu, W. Schiffer, C. Shea, P. Carter, J. S. Fowler, Pharmacokinetics of the potent hallucinogen, salvinorin A in primates parallels the rapid onset and short duration of effects in humans. *Neuroimage*, **2008**, *41*, 1044–1050
5. Roach, J. J.; Shenvi, R. A. A review of salvinorin analogs and their kappa-opioid receptor activity. *Bioorg. Med. Chem. Lett.* **2018**, 28, 1436–1445
6. Bohn, L. M.; Aubé, J. Seeking (and finding) biased ligands of the kappa opioid receptor. *ACS Med. Chem. Lett.* **2017**, *8*, 694–700
7. Ho, J.-H.; Stahl, E. L.; Schmid, C. L.; Aubé, J.; Bohn, L. M. G protein signaling–biased agonism at the κ-opioid receptor is maintained in striatal neurons. *Sci. Signal.* **2018**, *11*, eaar4309
8. Teksin, Z. S.; Lee, I. J.; Nemieboka, N. N.; Othman, A. A.; Upreti, V. V.; Hassan, H. E.; Syed, S. S.; Prisinzano, T. E.; Eddington, N. D. Evaluation of the transport, in vitro metabolism and pharmacokinetics of Salvinorin A, a potent hallucinogen. *Eur. J. Pharm. Biopharm.* **2009**, *72*, 471–477
9. Hooker, J. M.; Munro, T. A.; Béguin, C.; Alexoff, D.; Shea, C.; Xu, Y.; Cohen, B. M. Salvinorin A and derivatives: protection from metabolism does not prolong short-term, whole-brain residence. *Neuropharmacology*, **2009**, *57*, 386–391
10. Beguin, C.; Potter, D. N.; DiNieri, J. A.; Munro, T. A.; Richards, M. R.; Paine, T. A.; Berry, L.; Zhao, Z.; Roth, B. L.; Xu, W.; Liu-Chen, L. Y. N-methylacetamide analog of salvinorin A: A highly potent and selective κ-opioid receptor agonist with oral efficacy. *J. Pharm. Exper. Ther.* **2008**, *324*, 188–195
11. Prisinzano, T. E.; Rothman, R. B. Salvinorin A analogs as probes in opioid pharmacology. *Chem. Rev.* **2008**, *108*, 1732–1743
12. White, K. L.; Robinson, J. E.; Zhu, H.; DiBerto, J. F.; Polepally, P. R.; Zjawiony, J. K.; Nichols, D. E.; Malanga, C. J.; Roth, B. L. The G protein–biased κ-opioid receptor agonist RB-64 is analgesic with a unique spectrum of activities in vivo. *J. Pharmacol. Exp. Ther.* **2015**, *352*, 98–109
13. Paton, K. F.; Biggerstaff, A.; Kaska, S.; Crowley, R. S.; La Flamme, A. C.; Prisinzano, T. E.; Kivell, B. M.; Evaluation of biased and balanced salvinorin A analogs in preclinical models of pain. *Front Neurosci.* **2020**, *14*, 1–13
14. Scheerer, J. R.; Lawrence, J. F.; Wang, G. C.; Evans, D. A. Asymmetric synthesis of salvinorin A, a potent κ opioid receptor agonist. *J. Am. Chem. Soc.* **2007**, *129*, 8968–8969
15. Nozawa, M.; Suka, Y.; Hoshi, T.; Suzuki, T.; Hagiwara, H. Total synthesis of the hallucinogenic neoclerodane diterpenoid salvinorin A. *Org. Lett.* **2008**, *10*, 1365–1368
16. Hagiwara, H.; Suka, Y.; Nojima, T.; Hoshi, T.; Suzuki, T. Second-generation synthesis of salvinorin A. *Tetrahedron*, **2009**, *65*, 4820–4825
17. Line, N. J.; Burns, A. C.; Butler, S. C.; Casbohm, J.; Forsyth, C. J. Total Synthesis of (−)-Salvinorin A. *Chem. Eur. J.* **2016**, *22*, 17983–17986
18. Wang, Y.; Metz, P. Total Synthesis of the Neoclerodane Diterpene Salvinorin A via an Intramolecular Diels–Alder Strategy. *Org. Lett.* **2018**, *20*, 3418–3421
19. Halang, M.; Maier, M. E. Formal Total Synthesis of Salvinorin A. *ChemistryOpen*, **2022**, *11*, e202200015

20. Zimdars, P.; Wang, Y.; Metz, P. A Protecting-Group-Free Synthesis of (−)-Salvinorin A. *Chem. Eur. J.* **2021**, *27*, 7968–7973
21. Bergman, Y. E.; Mulder, R.; Perlmutter, P. Total synthesis of 20-norsalvinorin A. 1. Preparation of a key intermediate. *J. Org. Chem.* **2009**, *74*, 2589–2591
22. Hill, S. J.; Brion, A. U. C. M.; Shenvi, R. A. Chemical syntheses of the salvinorin chemotype of KOR agonist. *Nat. Prod. Rep.* **2020**, *37*, 1478–1496
23. Sherwood, A. M.; Williamson, S. E.; Crowley, R. S.; Abbott, L. M.; Day, V. W.; Prisinzano, T. E. Modular Approach to pseudo-Neoclerodanes as Designer κ-Opioid Ligands. *Org. Lett.* **2017**, *19*, 5414–5417
24. Roach, J. J.; Sasano, Y.; Schmid, C. L.; Zaidi, S.; Katrich, V.; Stevens, R. C.; Bohn, L. M.; Shenvi, R. A. Dynamic strategic bond analysis yields a ten-step synthesis of 20-nor-Salvinorin A, a potent κ-OR agonist. *ACS Cent. Sci.* **2017**, *3*, 1329–1336
25. Hirasawa, S.; Cho, M.; Brust, T. F.; Roach, J. J.; Bohn, L. M.; Shenvi, R. A. O6C-20-nor-salvinorin A is a stable and potent KOR agonist. *Bioorg. Med. Chem. Lett.* **2018**, *28*, 2770–2722
26. Burrows, J.; Kamo, S.; Koide, K.; Scalable Birch reduction with lithium and ethylenediamine in tetrahydrofuran. *Science*, **2021**, *374*, 741-746
27. Peters, B. K.; Rodriguez, K. X.; Reisberg, S. H.; Bell, S. B. et al. Scalable and safe synthetic organic electroreduction inspired by Li-ion battery chemistry. *Science*, **2019**, *363*, 838–845
28. Lei, P.; Ding, Y.; Zhang, X.; Adijiang, A.; Li, H.; Ling, Y.; An, J. A practical and chemoselective ammonia-free Birch reduction. *Org. Lett.* **2018**, *20*, 3439–3442
29. Hilt, G.; Janikowski, J.; Hess, W. meta-Directing Cobalt-Catalyzed Diels–Alder Reactions. *Angew. Chem. Int. Ed.* **2006**, *45*, 5204 –5206
30. Hilt, G.; du Mesnil, F.-X.; An improved cobalt catalyst for homo Diels–Alder reactions of acyclic 1,3-dienes with alkynes. *Tetrahedron Lett.* **2000**, *41*, 6757–6761
31. Erver, F.; Kuttner, J. R.; Hilt, G. Multidirectional Cobalt-Catalyzed Diels–Alder/1,4-Hydrovinylation Sequences. *J. Org. Chem.* **2012**, *77*, 8375–8385
32. Mörschel, P.; Janikowski, J.; Hilt, G. Frenking, G. Ligand-Tuned Regioselectivity of a Cobalt-Catalyzed Diels−Alder Reaction. A Theoretical Study. *J. Am. Chem. Soc.* **2008**, *130*, 8952–8966
33. Raebiger, J. W.; Miedaner, A.; Curtis, C. J.; Miller, S. M.; Anderson, O.P.; DuBois, D.L. Using ligand bite angles to control the hydricity of palladium diphosphine complexes. *J. Am. Chem. Soc.* **2004**, *126*, 5502–5514
34. Jacobsen, E. N.; Zhang, W.; Muci, A. R.; Ecker, J. R.; Ding, L. Highly enantioselective epoxidation catalysts derived from 1, 2-diaminocyclohexane. *J. Am. Chem. Soc.* **1991**, *113*, 7063–7064
35. Adam, W.; Fell, R. T.; Stegmann, V. R.; Saha-Möller, C. R. Synthesis of Optically Active α-Hydroxy Carbonyl Compounds by the Catalytic, Enantioselective Oxidation of Silyl Enol Ethers and Ketene Acetals with (Salen)manganese(III) Complexes. *J. Org. Chem.* **1998**, *120*, 708–714
36. Wang, Z. W.; Tu, Y.; Frohn, M.; Zhang, J. R.; Shi, Y. An efficient catalytic asymmetric epoxidation method. *J. Am. Chem. Soc.* **1997**, *119*, 11224–11235
37. Saravanan, P.; Singh, V. K. An efficient method for acylation reactions. *Tetrahedron Lett.* **1999**, *40*, 2611–2614
38. Lingham, A. R.; Hügel, H. M.; Rook, T. J. Studies towards the synthesis of salvinorin A. *Aust. J. Chem.* **2006**, *59*, 340–348
39. Rodríguez-Fernández, M.; Yan, X.; Collados, J. F.; White, P. B.; Harutyunyan, S. R. Lewis acid enabled copper-catalyzed asymmetric synthesis of chiral β-substituted amides. *J. Am. Chem. Soc.* **2017**, *139*, 14224–14231
40. Horeischi, F.; Biber, N.; Plietker, B. The Total Syntheses of Guttiferone A and 6-epi-Guttiferone A. *J. Am. Chem. Soc.* **2014**, *136*, 4026–4030
41. Baker, M. A.; Demoret, R. M.; Ohtawa, M.; Shenvi, R. A. Concise asymmetric synthesis of (−)-bilobalide. *Nature*, **2019**, *575*, 643–646

42. Matsuo, J.; Murakami, M. The Mukaiyama aldol reaction: 40 years of continuous development. *Angew. Chem. Int. Ed.* **2013**, *52*, 9109–9118
43. Sinast, M.; Zuccolo, M.; Wischnat, J.; Sube, T.; Hasnik, F.; Baro, A.; Dallavalle, S.; Laschat, S. Samarium iodide-promoted asymmetric reformatsky reaction of 3-(2-haloacyl)-2-oxazolidinones with enals. *J. Org. Chem.* **2019**, *84*, 10050–10064
44. Lipshutz, B. H.; Pollart, D.; Monforte, J.; Kotsuki, H. Pd (II)-catalyzed acetal/ketal hydrolysis/exchange reactions. *Tetrahedron Lett.* **1985**, *26*, 705–708
45. Rothermal, K.; Melikian, M.; Hioe, J.; Greindl, J.; Gramüller, J.; Žabka, M.; Sorgenfrei, N.; Hausler, T.; Morana, F.; Gschwind, R. M. Internal acidity scale and reactivity evaluation of chiral phosphoric acids with different 3, 3′-substituents in Brønsted acid catalysis. *Chem. Sci.* **2019**, *10*, 10025–10034
46. Yang, C.; Xue, X.-S.; Jin, J.-L.; Li, X.; Cheng, J. P. Theoretical study on the acidities of chiral phosphoric acids in dimethyl sulfoxide: hints for organocatalysis. *J. Org. Chem.* **2013**, *78*, 7076–7085
47. Monaco, M. R.; Poladura, B.; Diaz de Los Bernardos, M.; Leutzsch, M.; Goddard, R.; List, B. Activation of carboxylic acids in asymmetric organocatalysis. *Angew. Chem. Int. Ed.*; **2014**, *53*, 7063-7067
48. Kötzner, L.; Webber, M. J.; Martínez, A.; De Fusco, C.; List, B. Asymmetric catalysis on the nanoscale: the organocatalytic approach to helicenes. *Angew. Chem. Int. Ed.* **2014**, *53*, 5202–5205
49. Christ, P.; Lindsay, A. G.; Vormittag, S. S.; Neudörfl, J. M.; Berkessel, A.; O'Donoghue, A. C. pKa Values of Chiral Brønsted Acid Catalysts: Phosphoric Acids/Amides, Sulfonyl/Sulfuryl Imides, and Perfluorinated TADDOLs (TEFDDOLs). *Chem. Eur. J.* **2011**, *17*, 8524–8528
50. Borovika, A.; Tang, P.-I.; Klapman, S.; Nagorny, P. Thiophosphoramide-based cooperative catalysts for Brønsted acid promoted ionic Diels–Alder reactions. *Angew. Chem. Int. Ed.* **2013**, *52*, 13424–13428
51. Choi, P. J.; Petterson, K. A.; Roberts, J. D. Ionization equilibria of dicarboxylic acids in dimethyl sulfoxide as studied by NMR. *J. Phys. Org. Chem.* **2002**, *15*, 278-286
52. Phipps, R. J.; Hamilton, G. L.; Toste, F. D. The progression of chiral anions from concepts to applications in asymmetric catalysis. *Nature Chem.* **2012**, *4*, 603–614
53. Evans, D. A.; Clark, J. S.; Metternich, R.; Novack, V. J.; Sheppard, G. S. Diastereoselective aldol reactions using. beta.-keto imide derived enolates. A versatile approach to the assemblage of polypropionate systems. *J. Am. Chem. Soc.* **1990**, *112*, 866
54. Li, N.; Chen, X.-H.; Zhou, S.-M.; Luo, S.-W.; Song, J.; Ren, L.; Gong, L.-Z. Asymmetric amplification in phosphoric acid catalyzed reactions. *Angew. Chem. Int. Ed.* **2010**, *49*, 6378–6381
55. Lovell, K. M.; Vasiljevik, T.; Araya, J. J.; Lozama, A.; Prevatt-Smith, K. M.; Day, V. W.; Dersch, C. M.; Rothman, R. B.; Butelman, E. R.; Kreek, M. J.; Prisinzano, T. E. Semisynthetic neoclerodanes as kappa opioid receptor probes. *Bioorg. Med. Chem.* **2012**, *20*, 3100–3110
56. Munro, T. A.; Duncan, K. K.; Xu, W.; Wang, Y.; Liu-Chen, L.-Y.; Carlezon Jr, W. A.; Cohen, B. M.; Béguin, C. Standard protecting groups create potent and selective κ opioids: Salvinorin B alkoxymethyl ethers. *Bioorg. Med. Chem.* **2008**, *16*, 1279–1286
57. Yan, F.; Mosier, P. D.; Westkaemper, R. B.; Stewart, J.; Zjawiony, J. K.; Vortherms, T. A.; Sheffler, D. J.; Roth, B. L. Identification of the molecular mechanisms by which the diterpenoid salvinorin A binds to κ-opioid receptors. *Biochemistry*, **2005**, *44*, 8643–8651
58. Nobeli, I.; Price, S. L.; Lommerse, J. P. M.; Taylor, R.; Hydrogen bonding properties of oxygen and nitrogen acceptors in aromatic heterocycles. *J. Comp. Chem.* **1997**, *18*, 2060-2074
59. Kumar, K.; Woo, S. M.; Siu, T.; Cortopassi, W. A.; Duarte, F.; Paton, R. S. Cation–π interactions in protein–ligand binding: Theory and data-mining reveal different roles for lysine and arginine. *Chem. Sci.* **2018**, *9*, 2655–2665 (2018).

60. Pang, Z.; Schafroth, M. A.; Ogasawara, D.; Wang, Y.; Nudell, V.; Lal, N. K.; Yang, D.; Wang, K. et al. In situ identification of cellular drug targets in mammalian tissue. *Cell*, **2022**, *185*, 1793–1805.e17
61. Lin, P.-X.; Li, J.-H.; Chen, S.-H.; Cheng, H.-C.; McKetin, R. Quantitative determination of salvinorin A, a natural hallucinogen with abuse liability, in Internet-available Salvia divinorum and endemic species of Salvia in Taiwan. *J. Food Drug Anal.* **2014**, *22*, 370–378
62. Groer, C. E.; Tidgewell, K.; Moyer, R. A.; Harding, W. W.; Rothman, R. B.; Prisinzano, T. E.; Bohn, L. M. An opioid agonist that does not induce μ-opioid receptor—arrestin interactions or receptor internalization. *Mol. Pharm.* **2007**, *71*, 549–557
63. Schmid, C. L.; Kennedy, N. M.; Ross, N. C.; Lovell, K. M.; Yue, Z.; Morgenweck, J.; Cameron, M. D.; Bannister, T. D.; Bohn, L. M. Bias Factor and Therapeutic Window Correlate to Predict Safer Opioid Analgesics. *Cell* **2017**, *171*, 1165-1175
64. Vega, M. M.; Crain, D. M.; Konkol, L. C.; Thomson, R. J. Enantioselective Synthesis of Metacycloprodigiosin via the 'Wasserman Pyrrole.' *Tetrahedron Lett.* **2015**, *56*, 3228–3230
65. Moriarty, R.M.; Vaid, R.K.; Ravikumar, V.T.; Hopins, T.E.; Farid, P. Hypervalent iodine oxidation of 5-substituted and 4,5-disubstituted pyrazol-3(2H)-ones: A facile synthesis of methyl-2-alkynoates and methyl 2,3-alkadienoates. *Tetrahedron* **1989**, *45*, 1605-1610
66. Narayanappa, A.; Hurem, D.; McNulty, J. Regioselective Ylide Formation on Acetal-Functionalized Trialkyl Phosphonium Salts: Extending the Scope of Carbonyl Homologation. *Synlett* **2017**, *28*, 2961-2965
67. Bi, L.; Zhang, Y.; Zhao, M.; Wang, C.; Chan, P.; Tok, J.; Peng, S. Novel synthesis and anti-inflammatory activities of 2,5-disubstituted-dioxacycloalkanes. *Bioorg. Med. Chem.* **2005**, *13*, 5640-5646
68. Zhao, M-X.; Shi, Y. Practical synthesis of an L-fructose-derived ketone catalyst for asymmetric epoxidation of olefins. *J. Org. Chem.* **2006**, *71*, 5377-5379
69. Jamison, T. F.; Ikeuchi, Y. Process for preparation of 1,2:4,5-bis-O-(1-methylethylidene)-β-L-erythro-2,3-hexodiulo-2,6-pyranose Patent WO 2011/109276 Al, September 9, 2011
70. Speck, K.; Wildermuth, R.; Magauer, T. Convergent Assembly of the Tetracyclic Meroterpenoid (−)-Cyclosmenospongine by a Non-Biomimetic Polyene Cyclization. *Angew. Chem.* **2006**, *128*, 14337-14341
71. Whitehurst, W. G.; Kim, J.; Koenig, S. G.; Chirik, P. J. Three-Component Coupling of Arenes, Ethylene, and Alkynes Catalyzed by a Cationic Bis(phosphine) Cobalt Complex: Intercepting Metallacyclopentenes for C–H Functionalization. *J. Am. Chem. Soc.* **2022**, *144*, 4530–4540 (2022).
72. Birch, A. J. *J. Chem. Soc.* **1944**, 430-436
73. Marcune, B. F.; Karady, S.; Dolling, U. H.; Novak, T. J. Methoxymethylation of Alcohols, Phenols, and Avermectin Aglycones Using MOM-2-pyridylsulfide. *J. Org. Chem.* **1999**, *64*, 2446–2449
74. Wang, L.; Hashidoko, Y.; Hashimoto, M. Cosolvent-Promoted O-Benzylation with Silver(I) Oxide: Synthesis of 1′-Benzylated Sucrose Derivatives, Mechanistic Studies, and Scope Investigation. *J. Org. Chem.* **2016**, *81*, 4464–4474
75. Poon, K.W.C.; House, S.E.; Dudley, G.B. A Bench-Stable Organic Salt for the Benzylation of Alcohols. *Synlett* **2005**, *20*, 3142-3144
76. Yasunori, O.; Mamiko, O.; Masafumi, B.; Hidetoshi, Y. Benzyl N-Phenyl-2,2,2-trifluoroacetimidate: A New and Stable Reagent for O-Benzylation. *Chem. Lett.* **2007**, *36*, 992-993
77. Asao, N.; Aikawa, H.; Tago, S.; Umetsu, K. Gold-Catalyzed Etherification and Friedel−Crafts Alkylation Using ortho-Alkynylbenzoic Acid Alkyl Ester as an Efficient Alkylating Agent. *Org. Lett.* **2007**, *9*, 4299–4302

78. Bartoli, G.; Bosco, M.; Locatelli, M.; Marcantoni, E.; Melchiorre, P.; Sambri, L. Unusual and Unexpected Reactivity of t-Butyl Dicarbonate (Boc_2O) with Alcohols in the Presence of Magnesium Perchlorate. A New and General Route to t-Butyl Ethers. *Org. Lett.* **2005**, *7*, 427–430
79. Otera, J.; Danoh, N.; Nozaki, H. Novel template effects of distannoxane catalysts in highly efficient transesterification and esterification. *J. Org. Chem.* **1991**, *56*, 5307–5311
80. Ye, B.; Yao, J.; Wu, C.; Zhu, H.; Yao, W.; Jin, L.; Dou, X. Rhodium-Catalyzed Asymmetric Conjugate Pyridylation with Pyridylboronic Acids. *ACS Catal.* **2022**, *12*, 2434–2440
81. Lingham, A. Studies Toward the Synthesis of Salvinorin A. Royal Melbourne Institute of Technology, Melbourne, Australia (2007).

NMR Characterization:

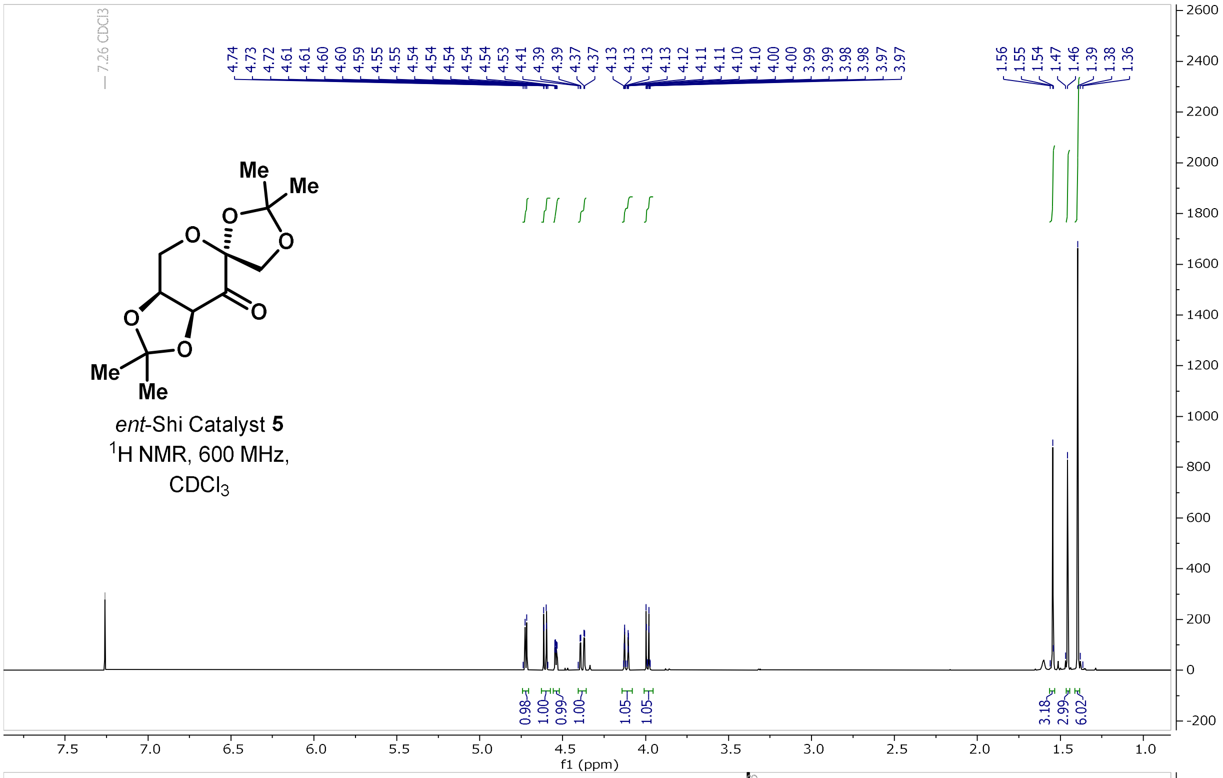

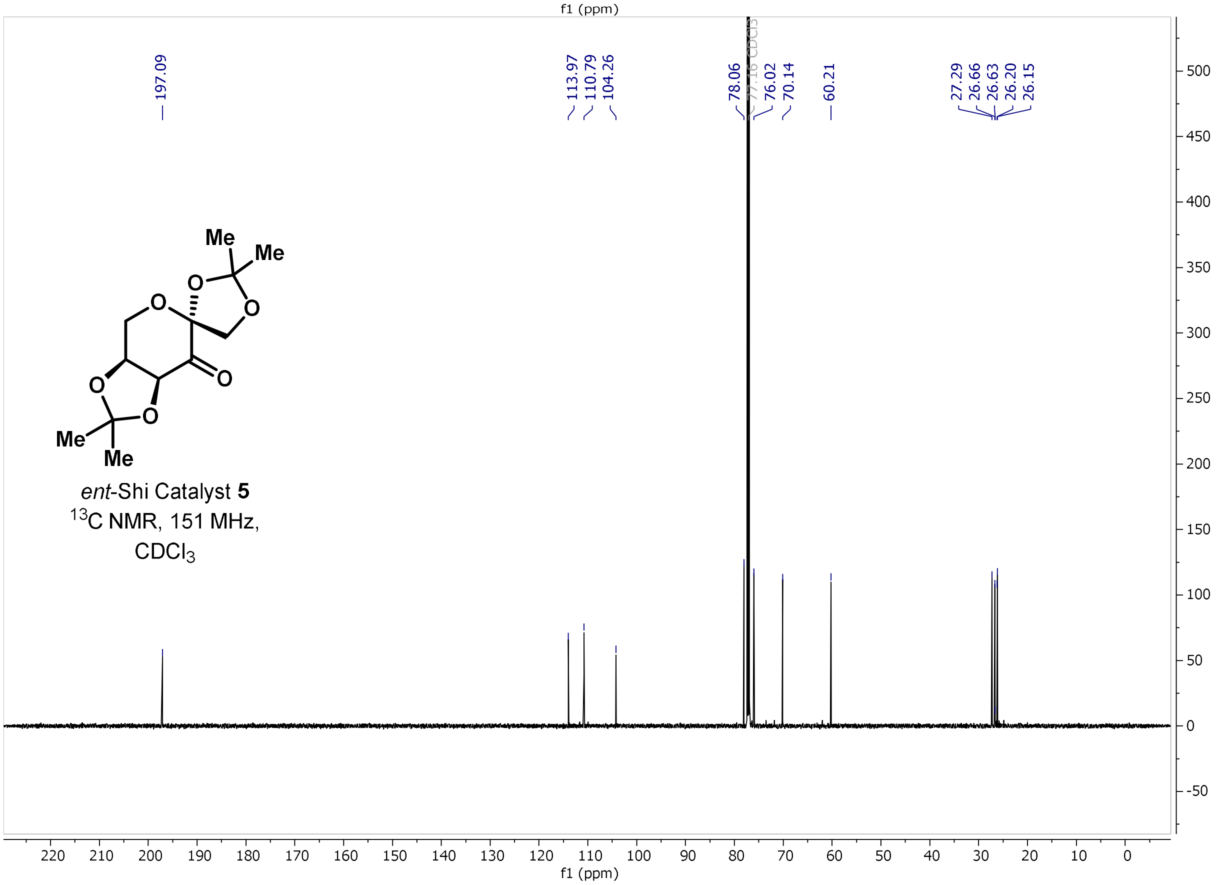

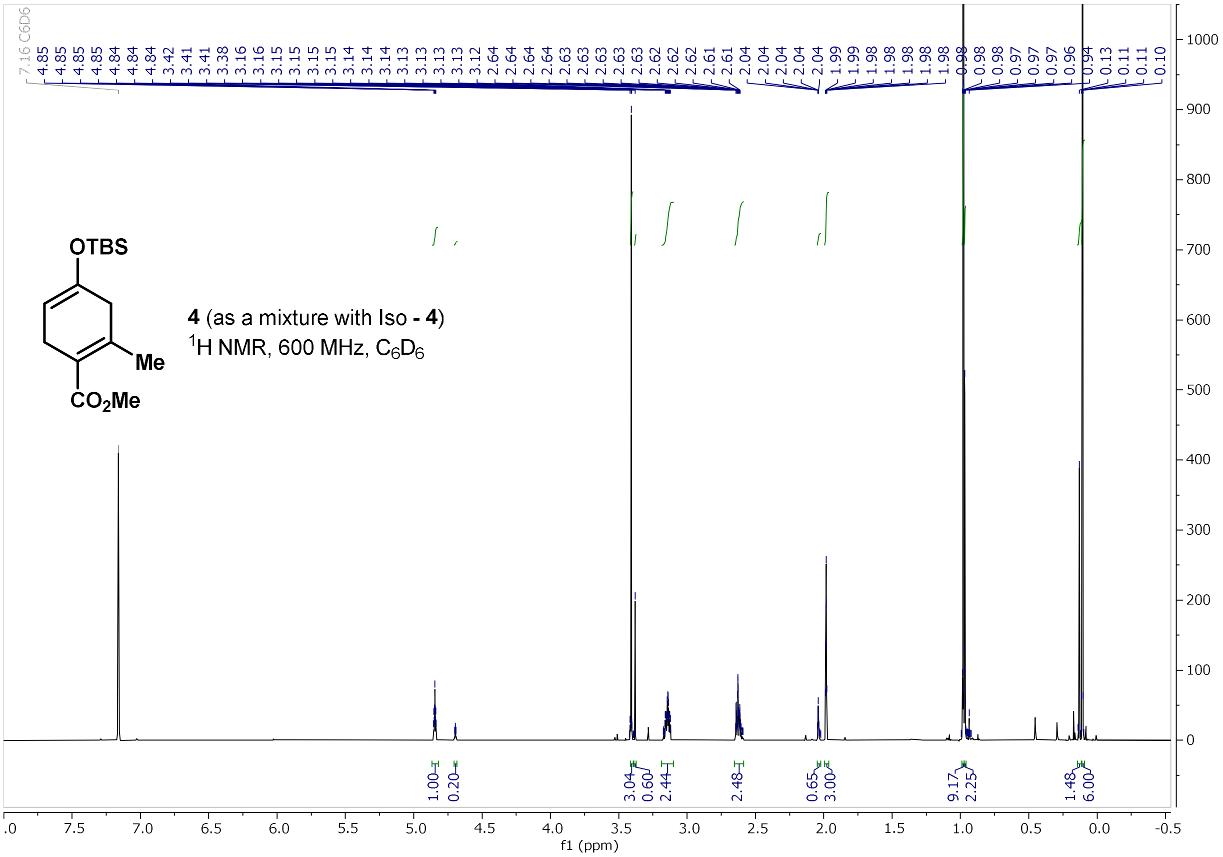
4 (as a mixture with Iso - 4)
^{1}H NMR, 600 MHz, C_6D_6

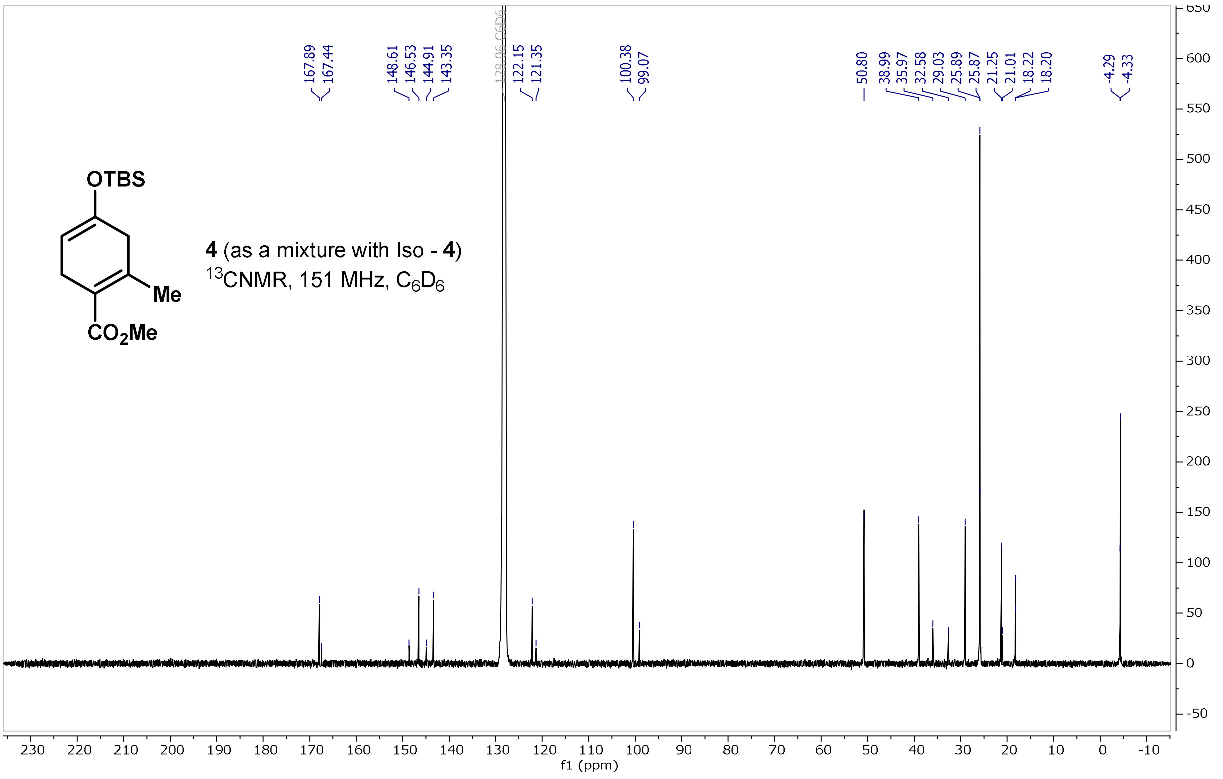
4 (as a mixture with Iso - 4)
^{13}CNMR, 151 MHz, C_6D_6

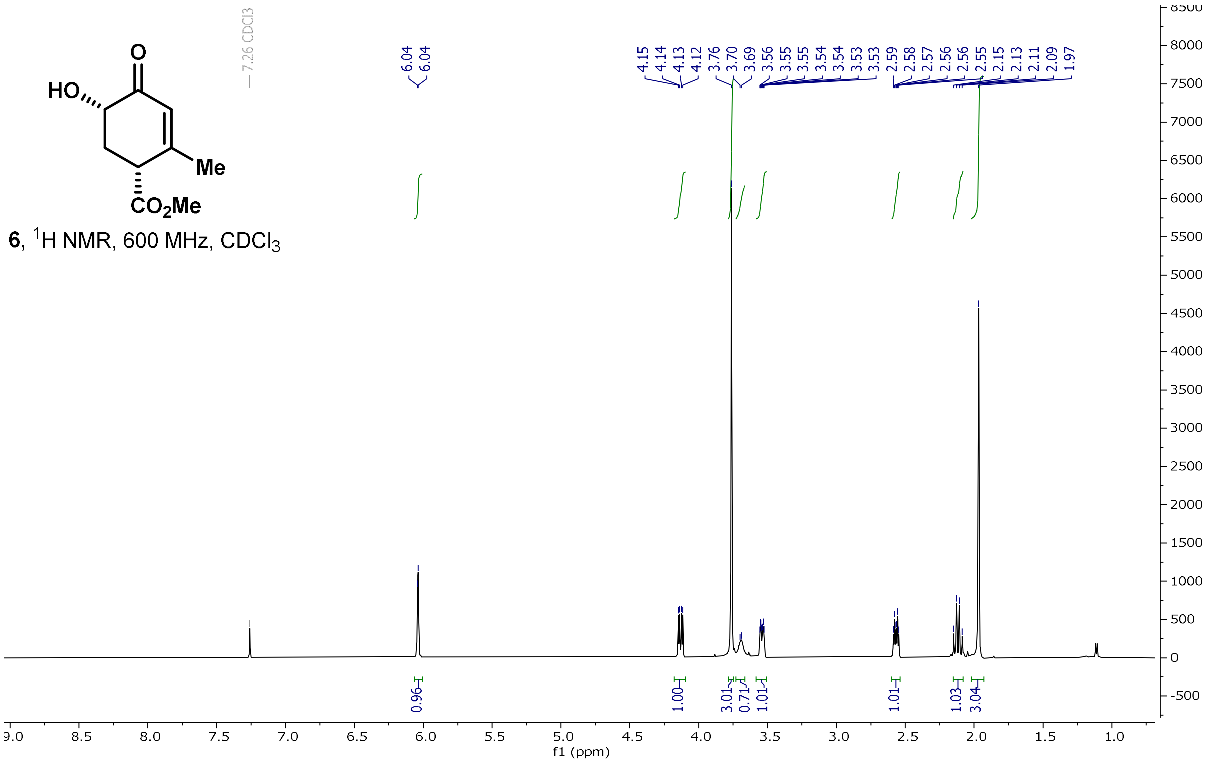

6, ¹H NMR, 600 MHz, CDCl3
7.26 CDCl3
6.04
6.04
4.15
4.14
4.13
4.12
3.76
3.70
3.69
3.56
3.55
3.55
3.54
3.54
3.53
3.53
2.59
2.58
2.57
2.56
2.56
2.55
2.15
2.13
2.11
2.09
1.97
0.96
1.00
3.01
0.71
1.01
1.01
1.03
3.04
f1 (ppm)

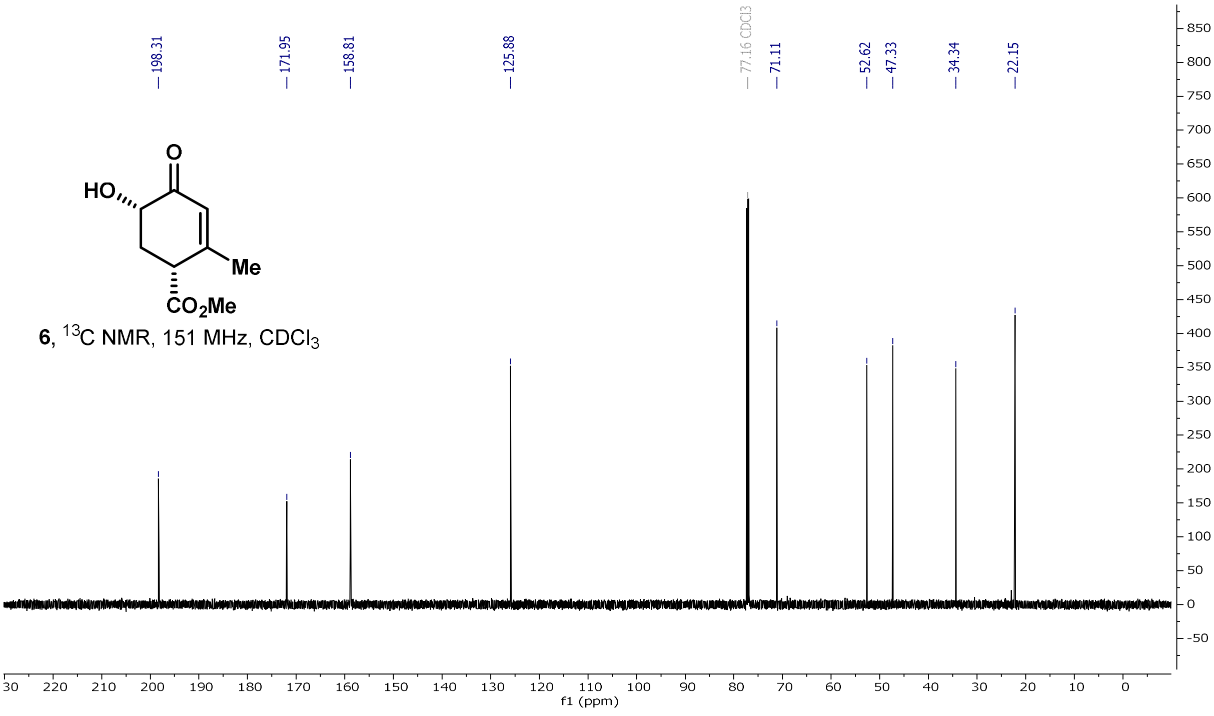

6, ¹³C NMR, 151 MHz, CDCl3
198.31
171.95
158.81
125.88
77.16 CDCl3
71.11
52.62
47.33
34.34
22.15
f1 (ppm)

Crude Shi-Epoxidation (prior to recrystallization, (+)-6), with 1,3,5 Trimethoxy benzene as an internal standard (0.4 equiv. / mmol **4**), ^{1}H NMR, 600 MHz, $CDCl_3$

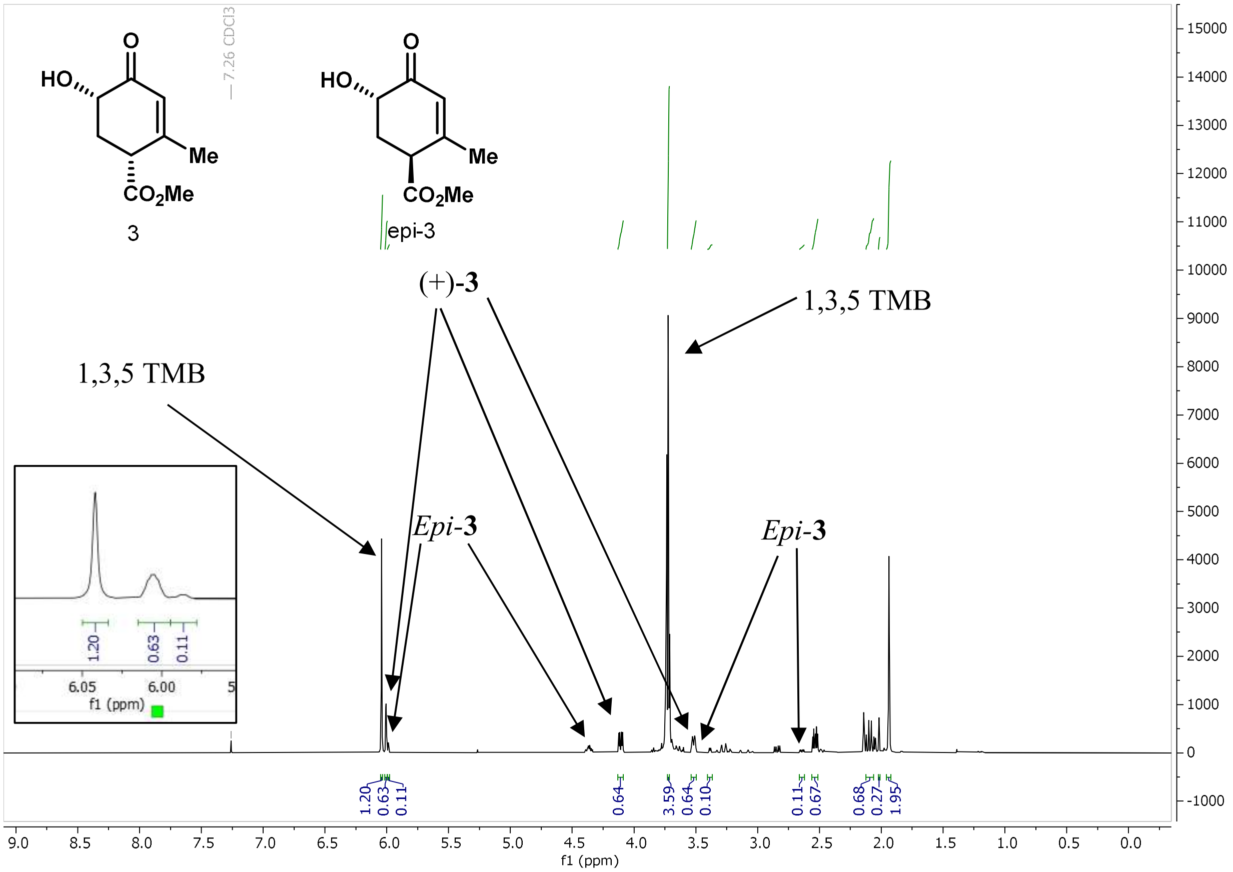

Epi – **3** peaks are assigned from previously reported spectra[81]

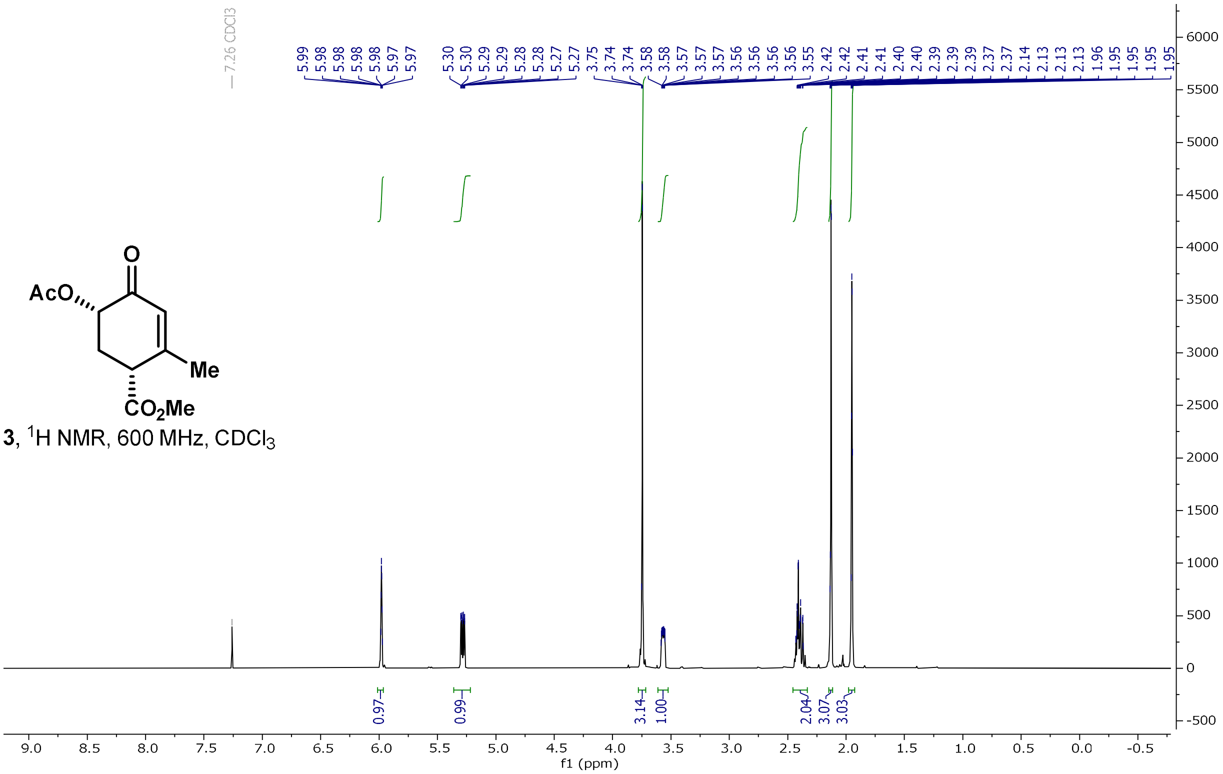
3, ^{1}H NMR, 600 MHz, CDCl$_3$
AcO
Me
CO$_2$Me
7.26 CDCl3
5.99 5.98 5.98 5.98 5.98 5.97 5.97
5.30 5.30 5.29 5.29 5.28 5.28 5.27 5.27
3.75 3.74 3.74 3.58 3.58 3.57 3.57 3.57 3.56 3.56 3.56 3.56 3.55
2.42 2.42 2.41 2.41 2.40 2.40 2.39 2.39 2.39 2.37 2.37 2.14 2.13 2.13 2.13 1.96 1.95 1.95 1.95 1.95
0.97 0.99 3.14 1.00 2.04 3.07 3.03
f1 (ppm)

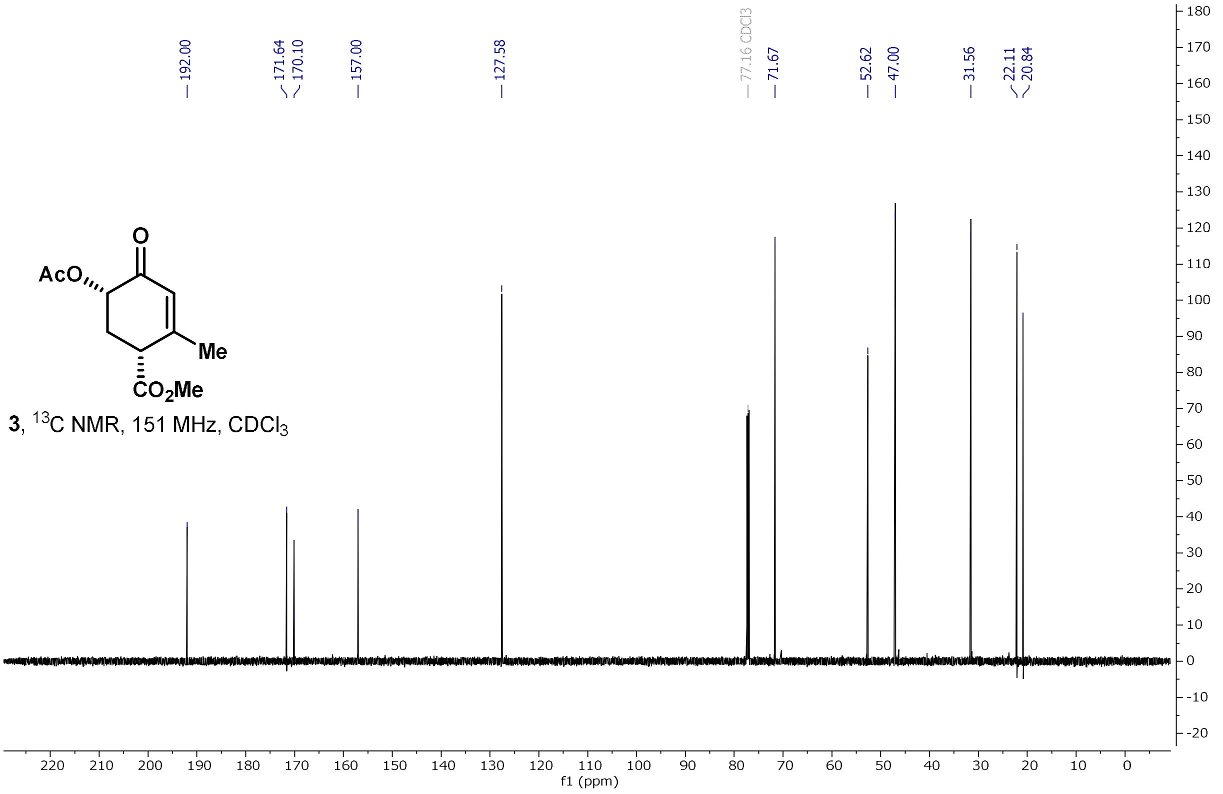
3, ^{13}C NMR, 151 MHz, CDCl$_3$
AcO
Me
CO$_2$Me
192.00
171.64 170.10
157.00
127.58
77.16 CDCl3
71.67
52.62
47.00
31.56
22.11 20.84
f1 (ppm)

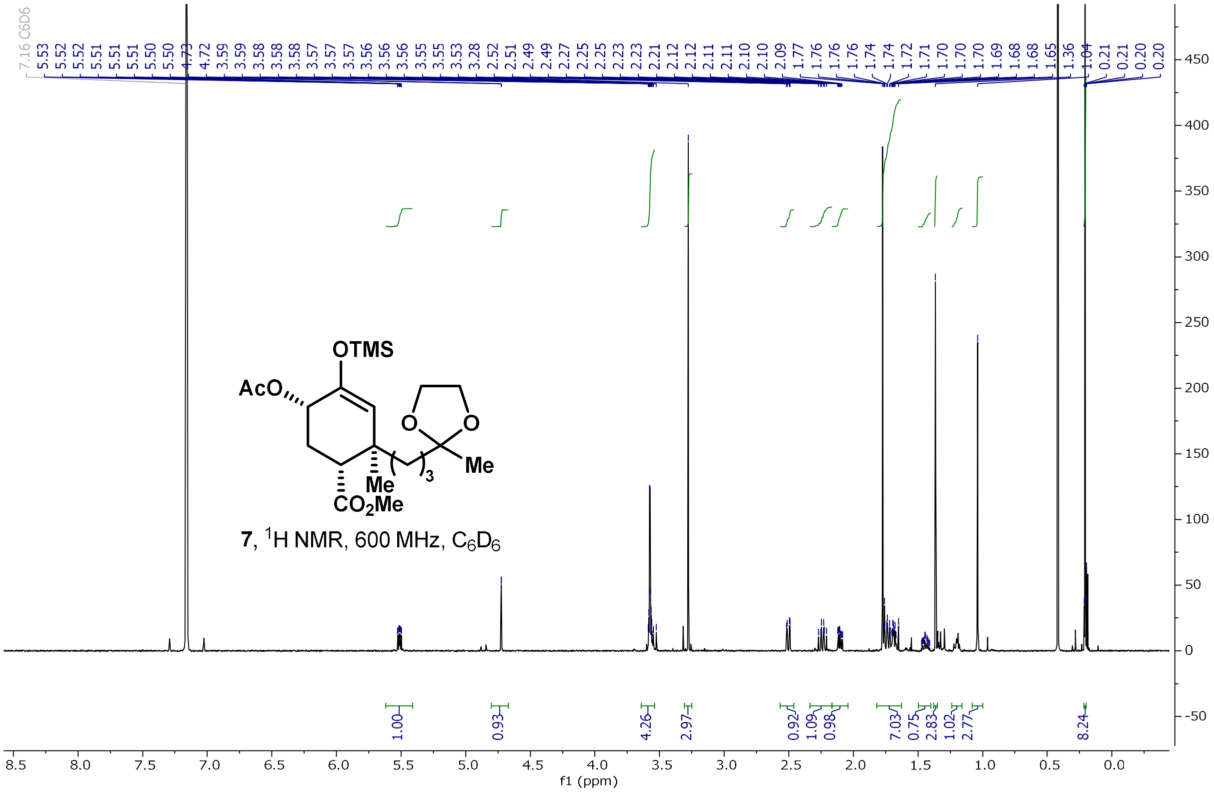
7.16 C6D6
5.53 5.52 5.52 5.51 5.51 5.51 5.50 5.50 4.73 4.72 3.59 3.59 3.58 3.58 3.58 3.57 3.57 3.57 3.56 3.56 3.56 3.55 3.55 3.53 3.28 2.52 2.51 2.49 2.49 2.27 2.25 2.25 2.23 2.23 2.21 2.12 2.12 2.11 2.11 2.10 2.10 2.09 1.77 1.76 1.76 1.76 1.74 1.72 1.71 1.70 1.70 1.70 1.69 1.68 1.68 1.65 1.36 1.04 0.21 0.21 0.20 0.20
OTMS
AcO
O
O
Me
Me
3
CO2Me
7, 1H NMR, 600 MHz, C6D6
1.00 0.93 4.26 2.97 0.92 1.09 0.98 7.03 0.75 2.83 1.02 2.77 8.24
f1 (ppm)

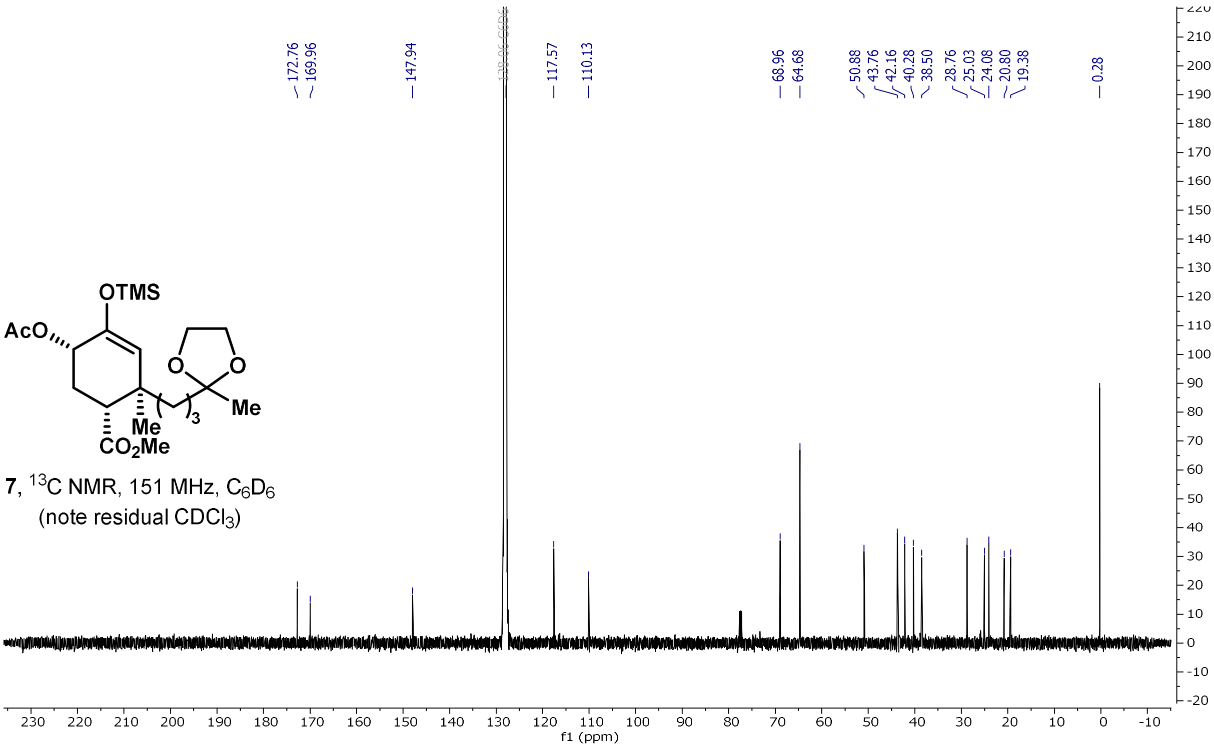
128.06 C6D6
172.76 169.96 147.94 117.57 110.13 68.96 64.68 50.88 43.76 42.16 40.28 38.50 28.76 25.03 24.08 20.80 19.38 0.28
OTMS
AcO
O
O
Me
Me
3
CO2Me
7, 13C NMR, 151 MHz, C6D6
(note residual CDCl3)
f1 (ppm)

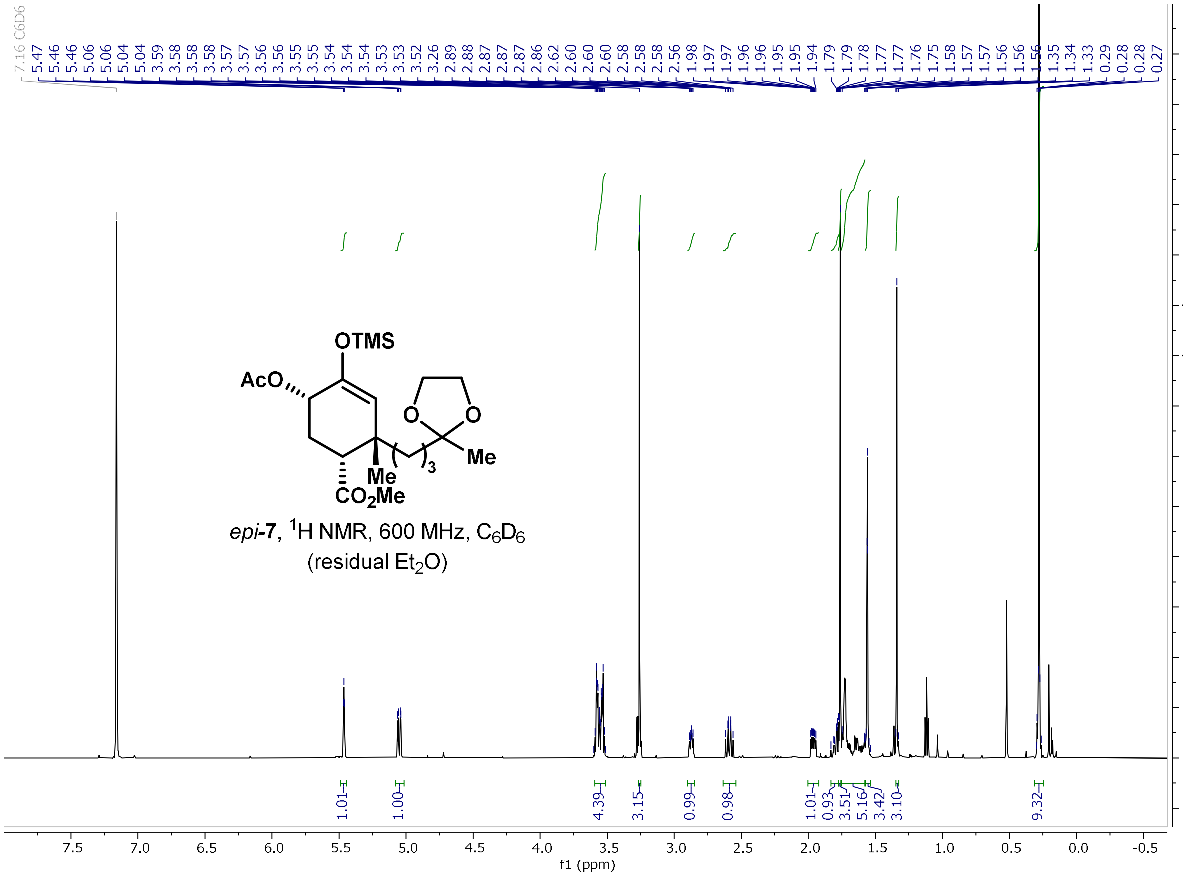
7.16 C6D6
5.47 5.46 5.06 5.06 5.04 5.04 3.59 3.58 3.58 3.58 3.57 3.57 3.56 3.56 3.55 3.55 3.54 3.54 3.54 3.53 3.53 3.52 3.26 2.89 2.88 2.87 2.87 2.87 2.86 2.62 2.60 2.60 2.60 2.58 2.58 2.58 2.56 1.98 1.97 1.97 1.96 1.96 1.95 1.95 1.94 1.79 1.79 1.78 1.77 1.77 1.76 1.75 1.58 1.57 1.57 1.56 1.56 1.56 1.35 1.34 1.33 0.29 0.28 0.28 0.27
OTMS
AcO
O
O
Me
Me
3
CO2Me
epi-7, 1H NMR, 600 MHz, C6D6
(residual Et2O)
1.01 1.00 4.39 3.15 0.99 0.98 1.01 0.93 3.51 5.16 3.42 3.10 9.32
f1 (ppm)

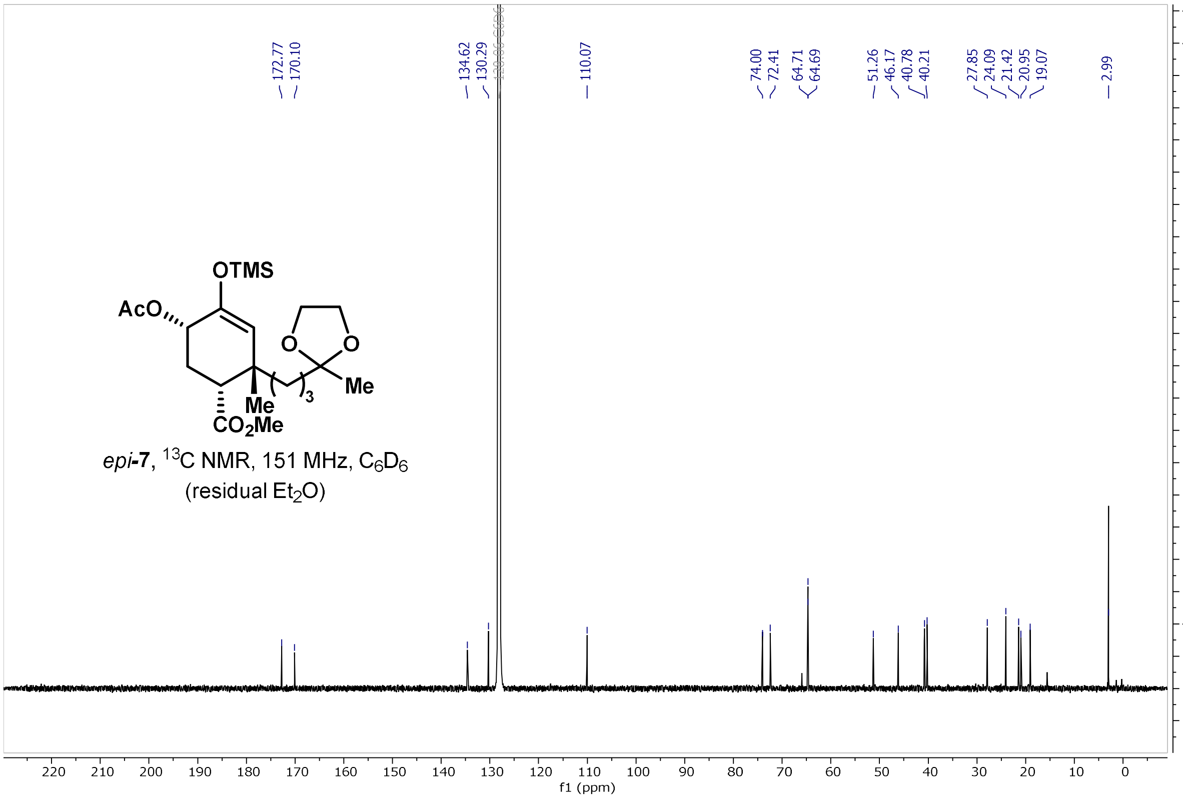
172.77 170.10 134.62 130.29 110.07 74.00 72.41 64.71 64.69 51.26 46.17 40.78 40.21 27.85 24.09 21.42 20.95 19.07 2.99
OTMS
AcO
O
O
Me
Me
3
CO2Me
epi-7, 13C NMR, 151 MHz, C6D6
(residual Et2O)
f1 (ppm)

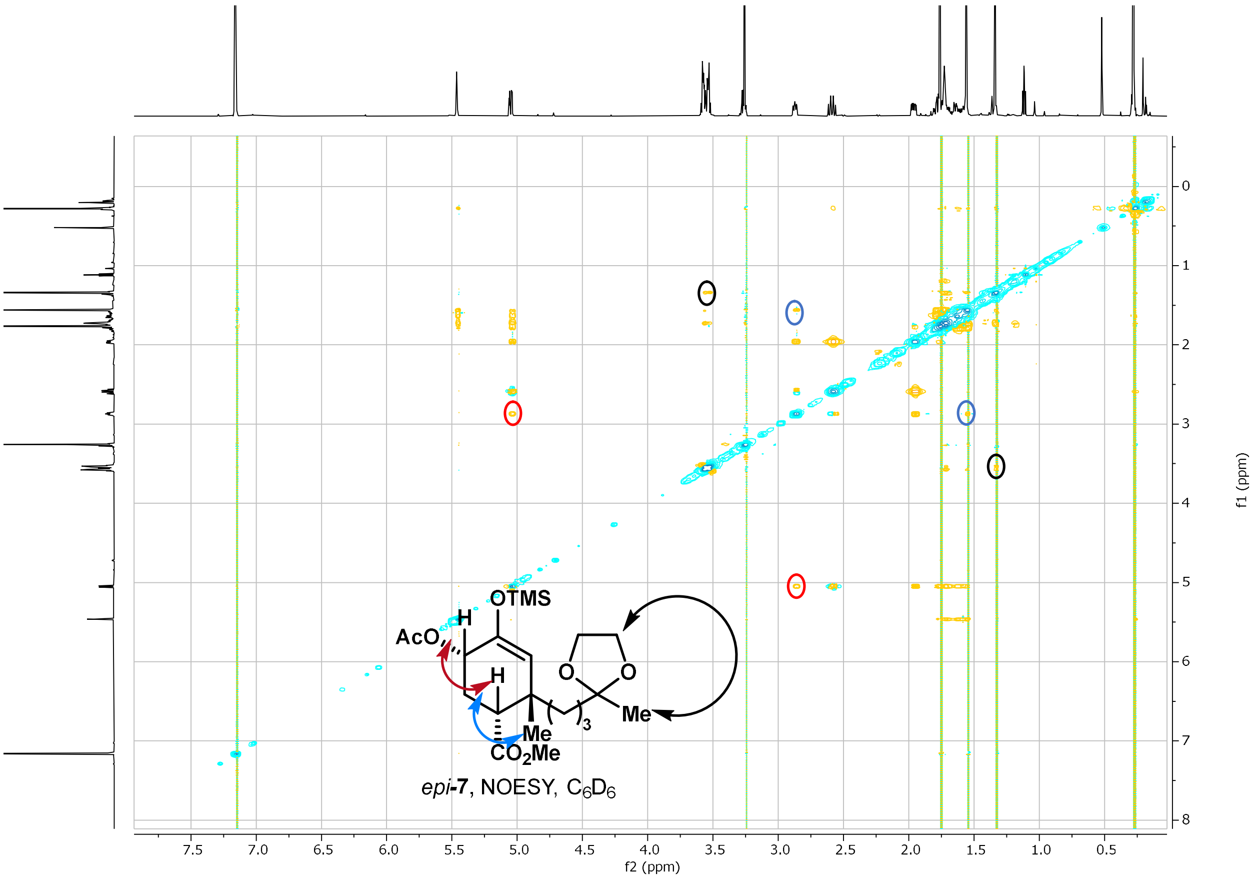
OTMS
AcO
H
H
O
O
Me
Me
3
CO2Me
epi-7, NOESY, C6D6
f2 (ppm)
f1 (ppm)

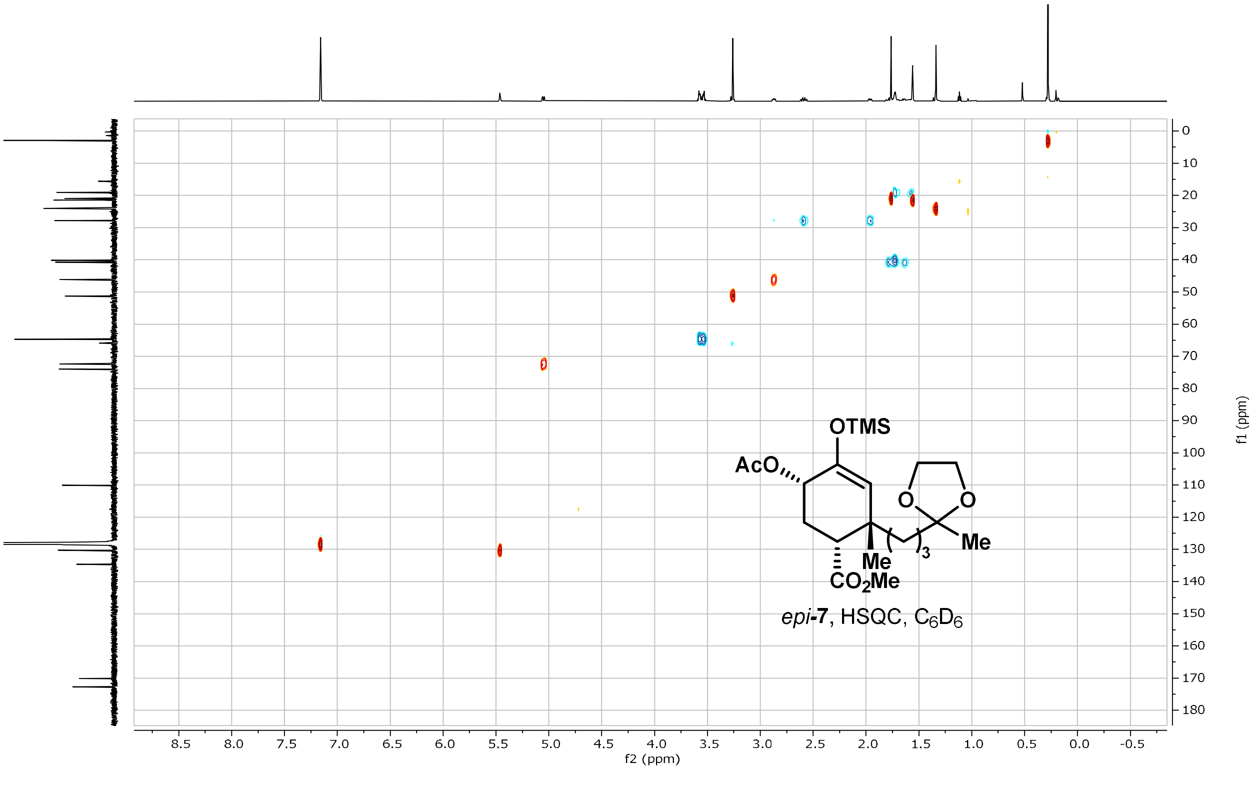
OTMS
AcO
O
O
Me
Me
3
CO2Me
epi-7, HSQC, C6D6
f2 (ppm)
f1 (ppm)

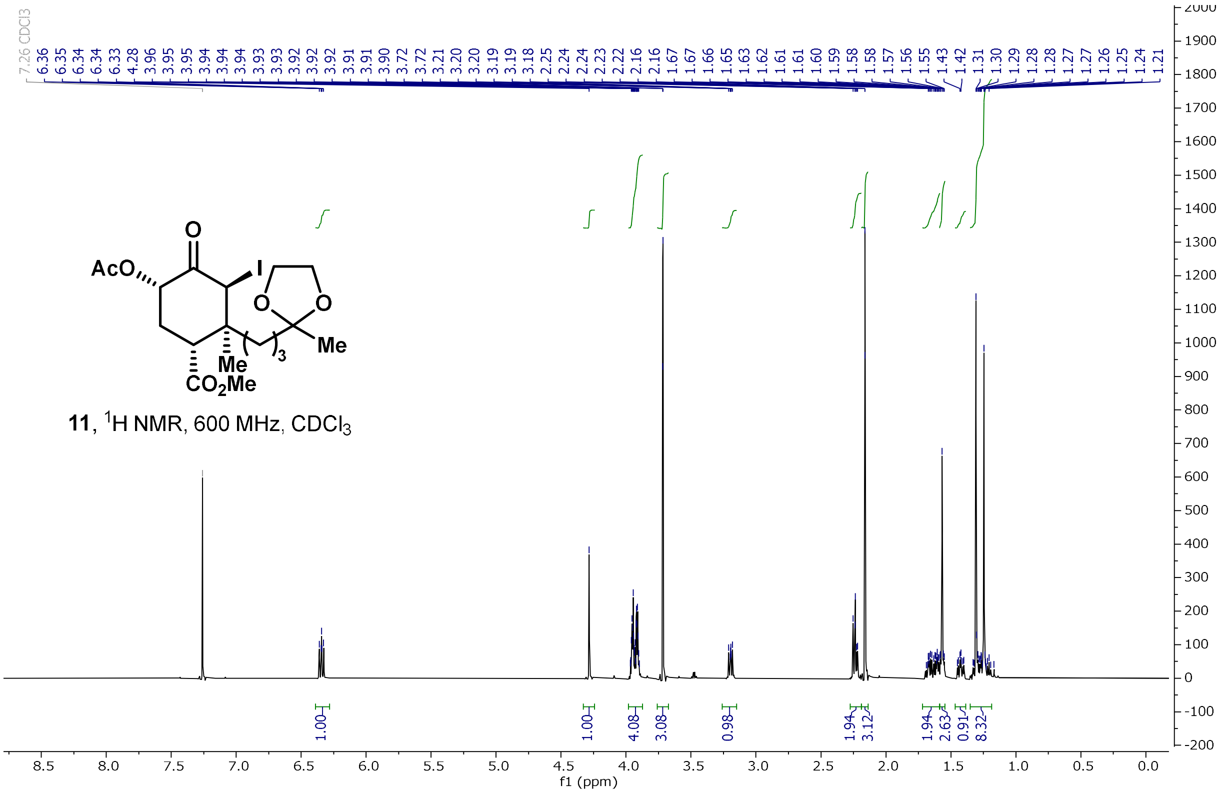

11, ^{1}H NMR, 600 MHz, $CDCl_3$

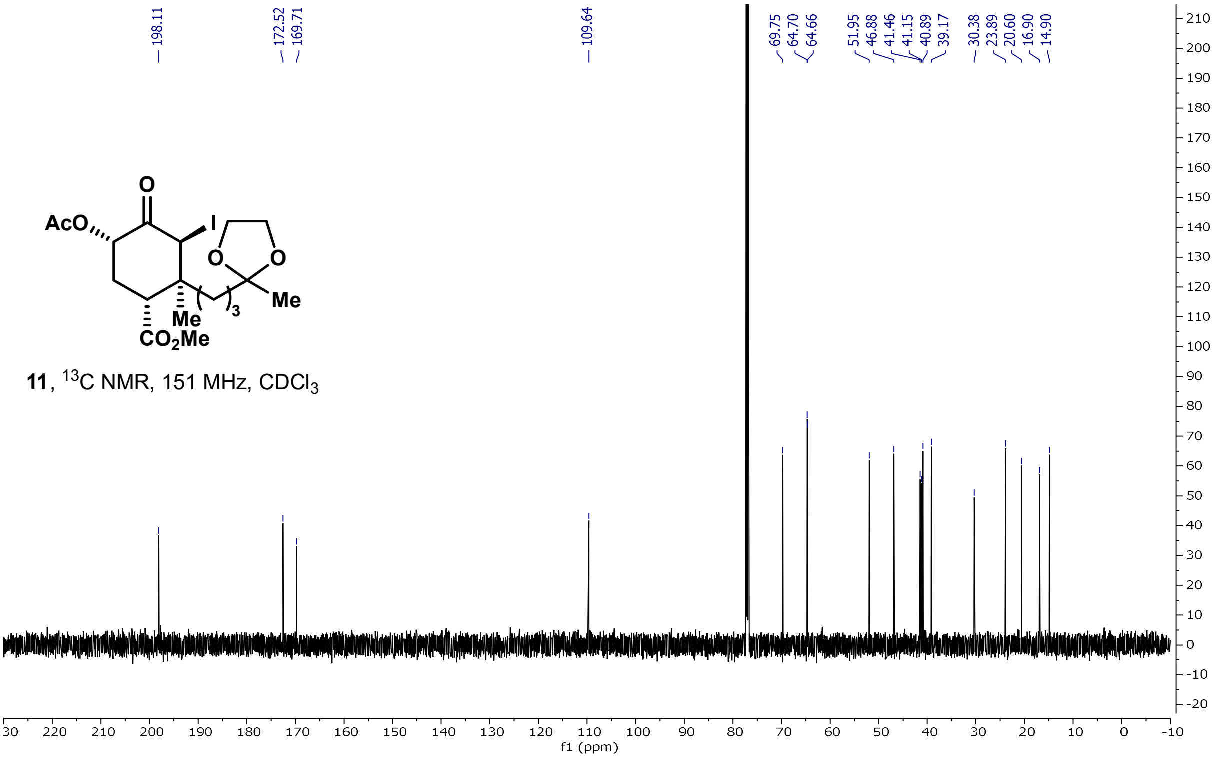

11, ^{13}C NMR, 151 MHz, $CDCl_3$

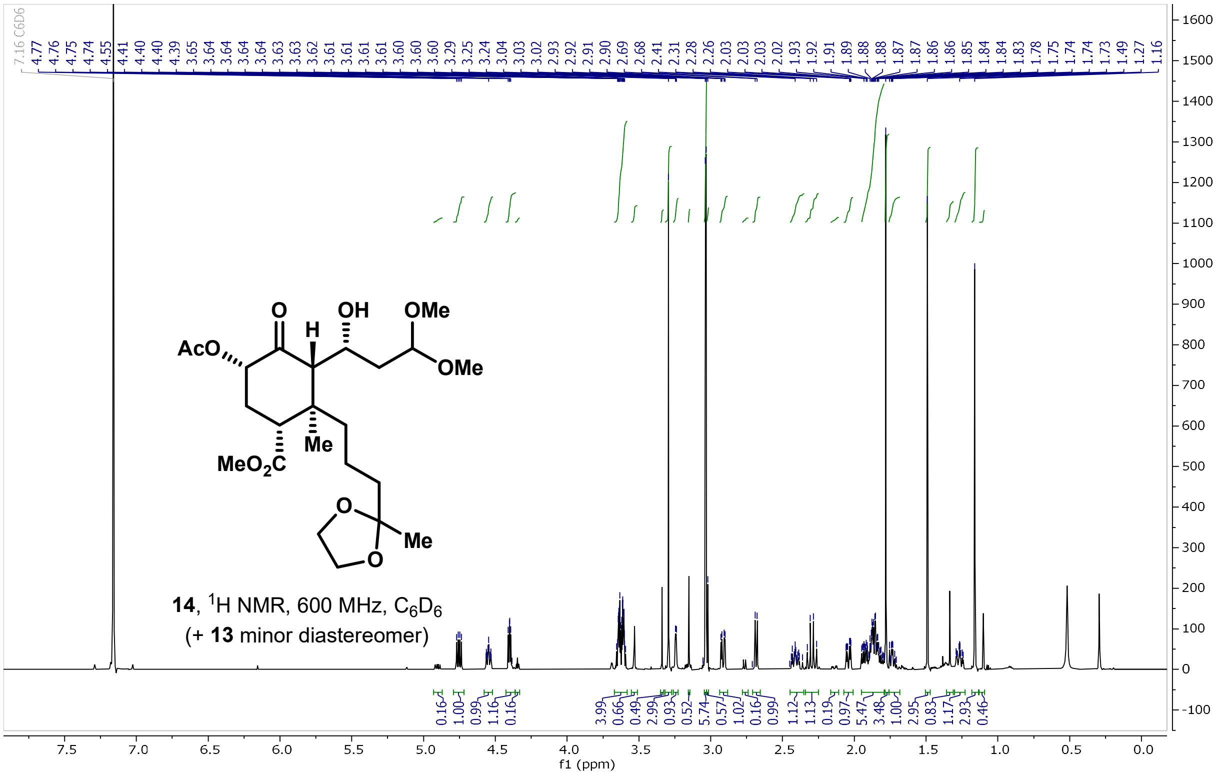

14, ^{1}H NMR, 600 MHz, C_6D_6
(+ **13** minor diastereomer)

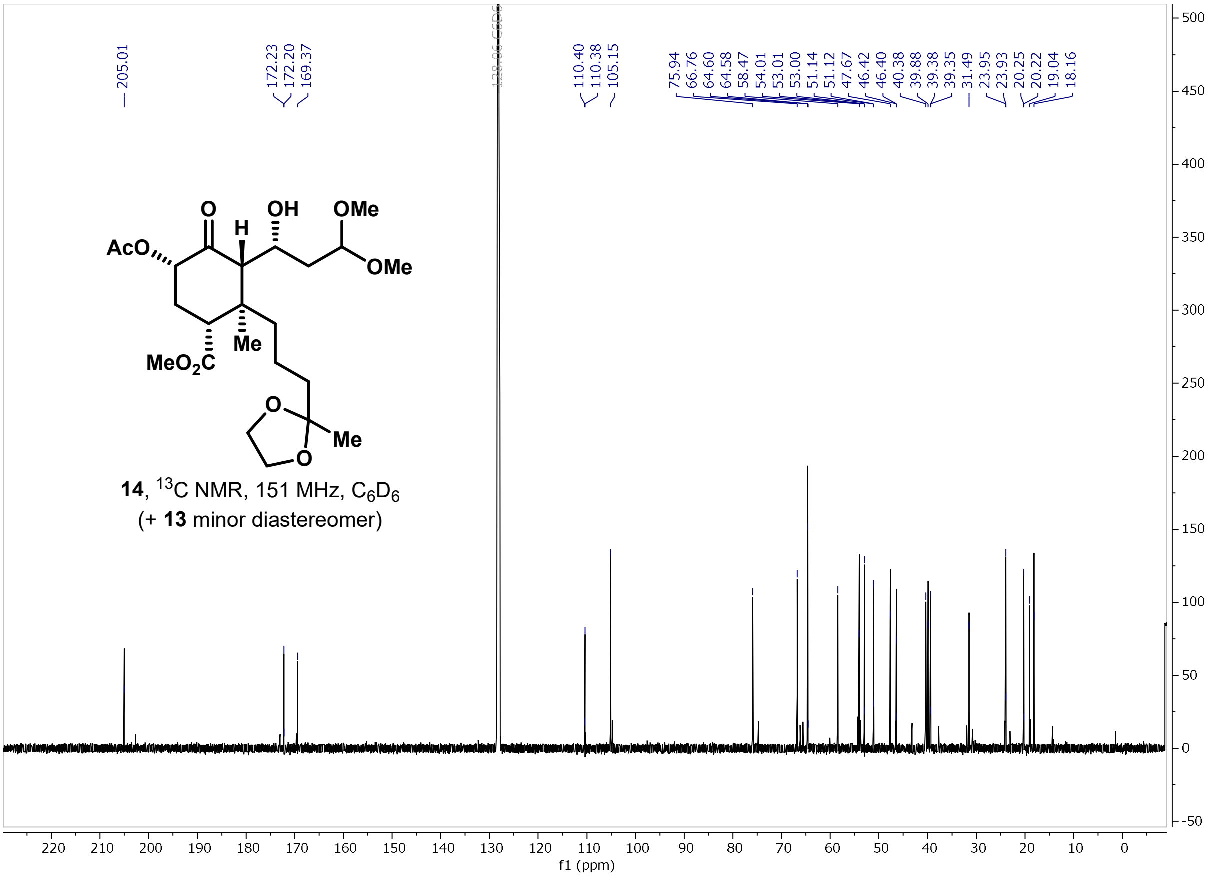

14, ^{13}C NMR, 151 MHz, C_6D_6
(+ **13** minor diastereomer)

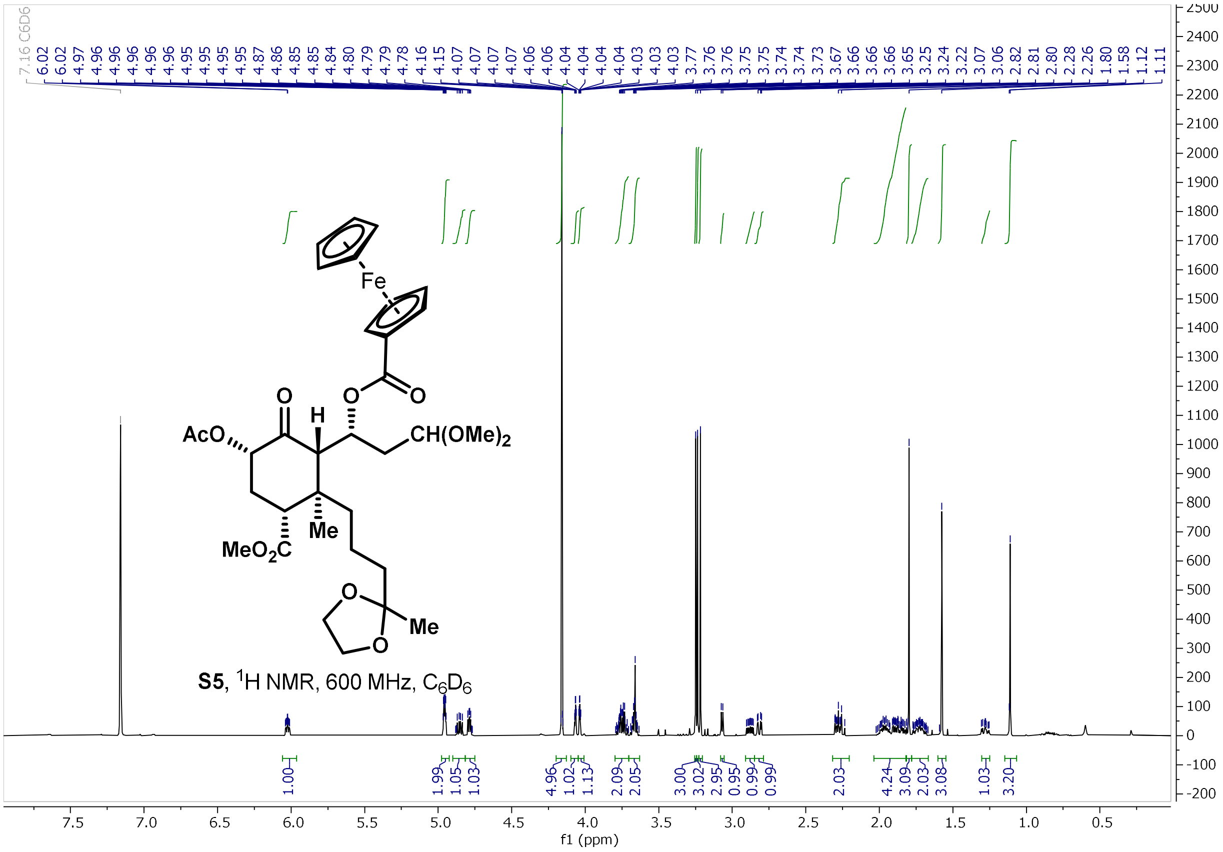

S5, ^{1}H NMR, 600 MHz, C_6D_6

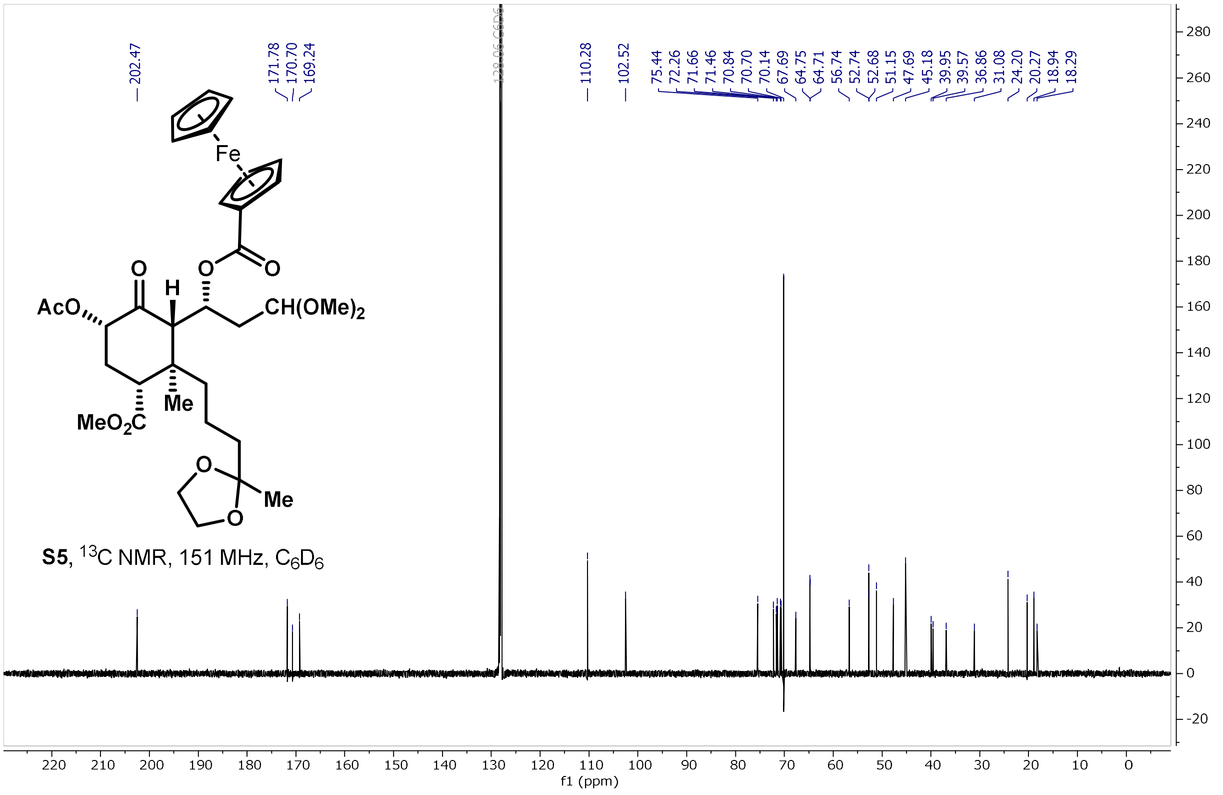

S5, ^{13}C NMR, 151 MHz, C_6D_6

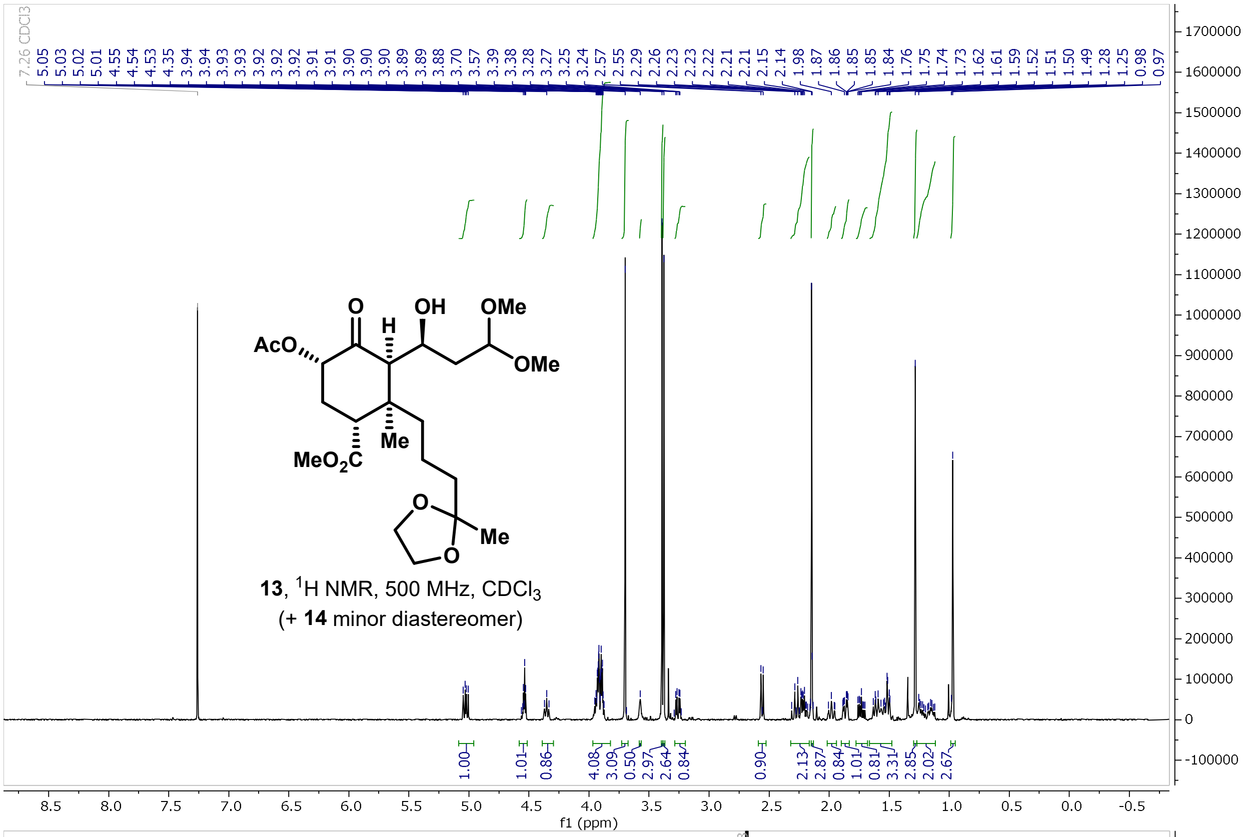

13, ^{1}H NMR, 500 MHz, $CDCl_3$
(+ **14** minor diastereomer)

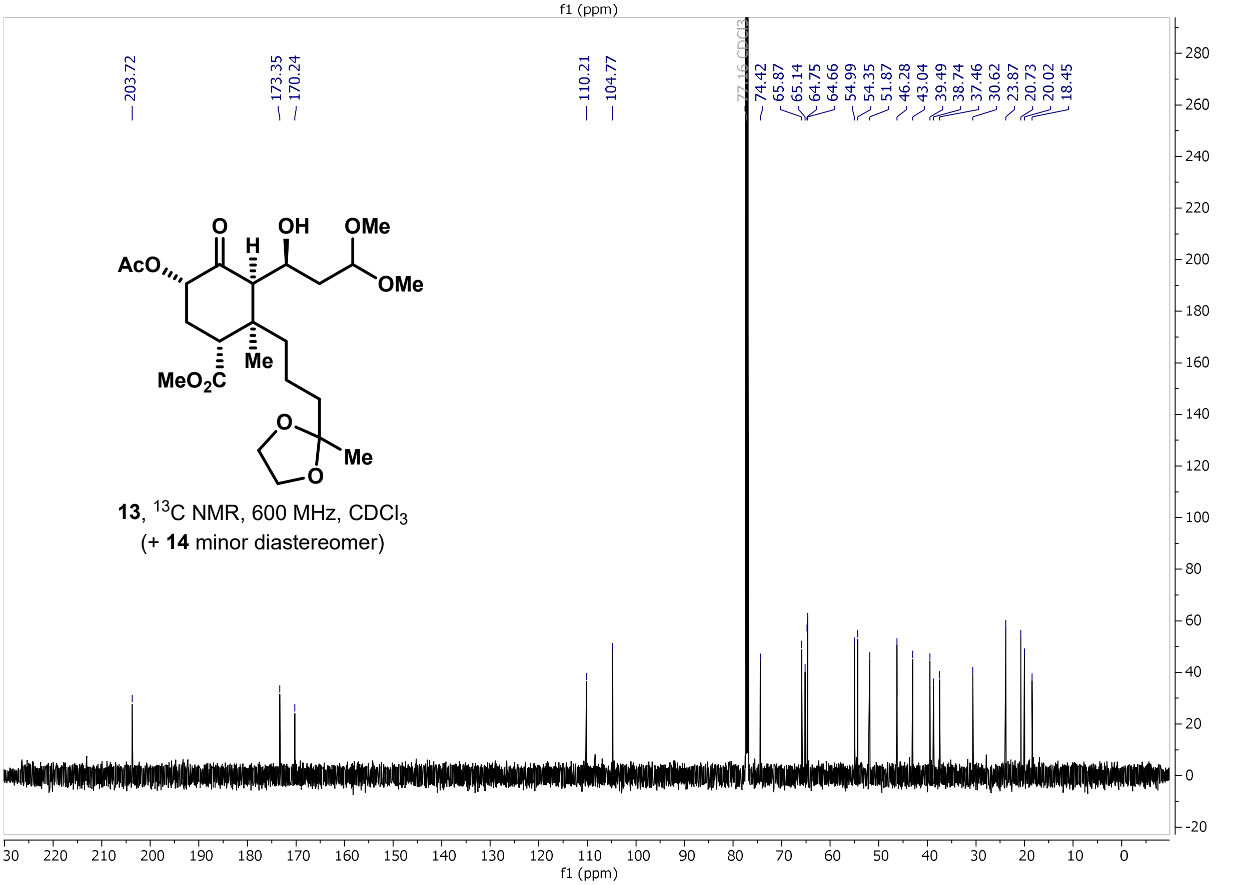

13, ^{13}C NMR, 600 MHz, $CDCl_3$
(+ **14** minor diastereomer)

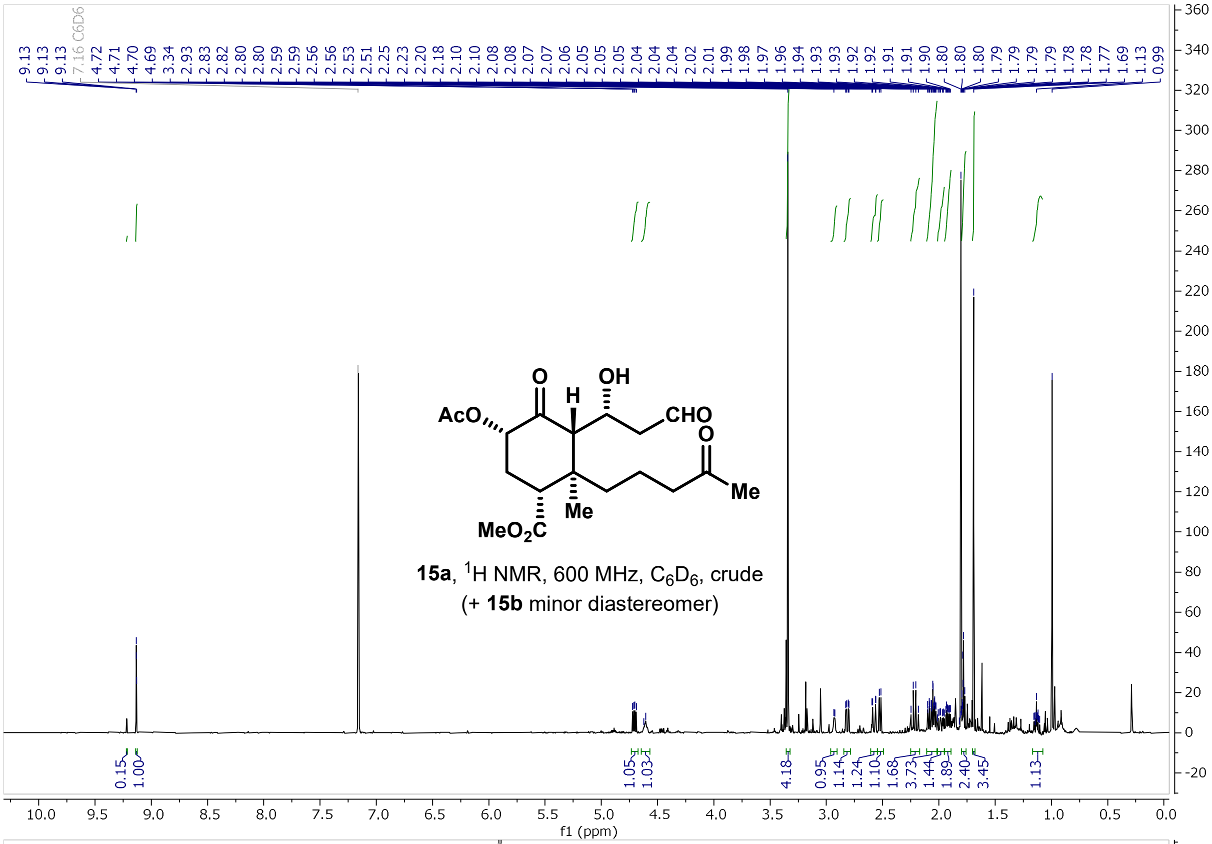

15a, ^1H NMR, 600 MHz, C_6D_6, crude
(+ **15b** minor diastereomer)

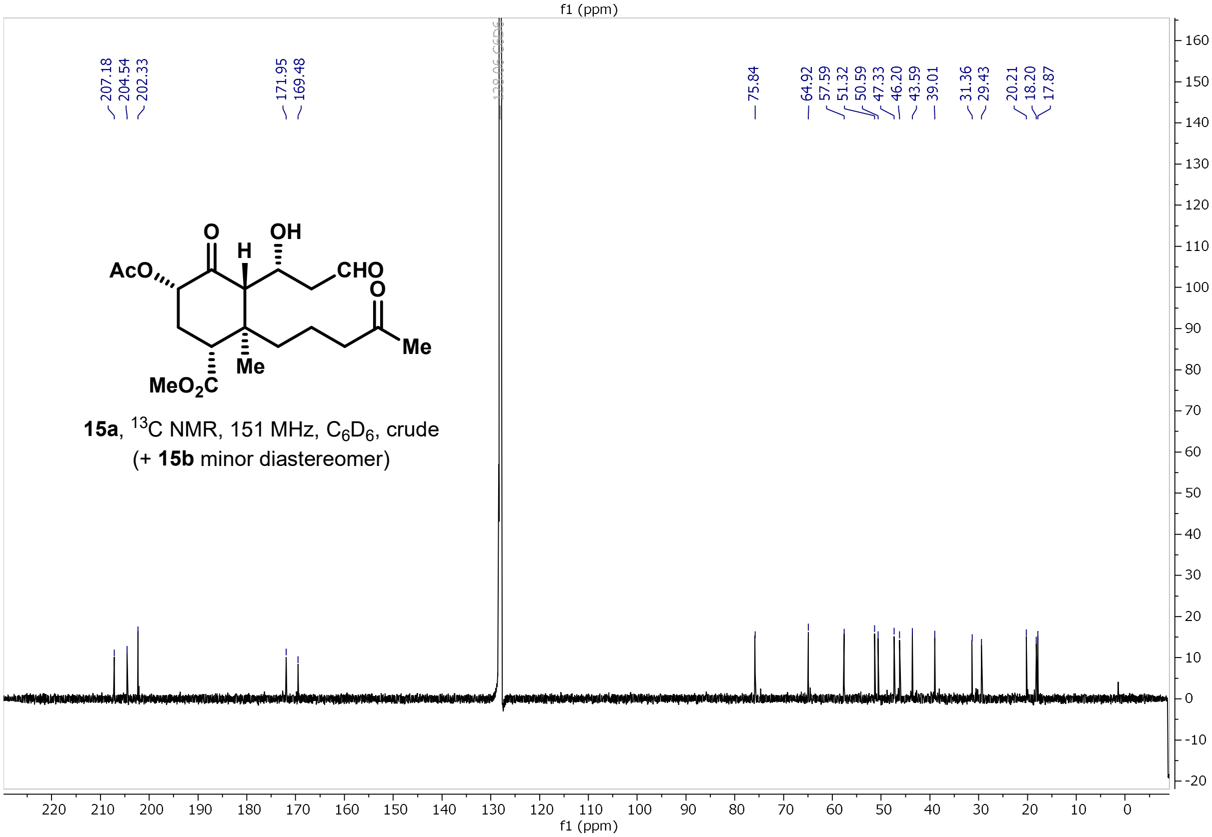

15a, ^{13}C NMR, 151 MHz, C_6D_6, crude
(+ **15b** minor diastereomer)

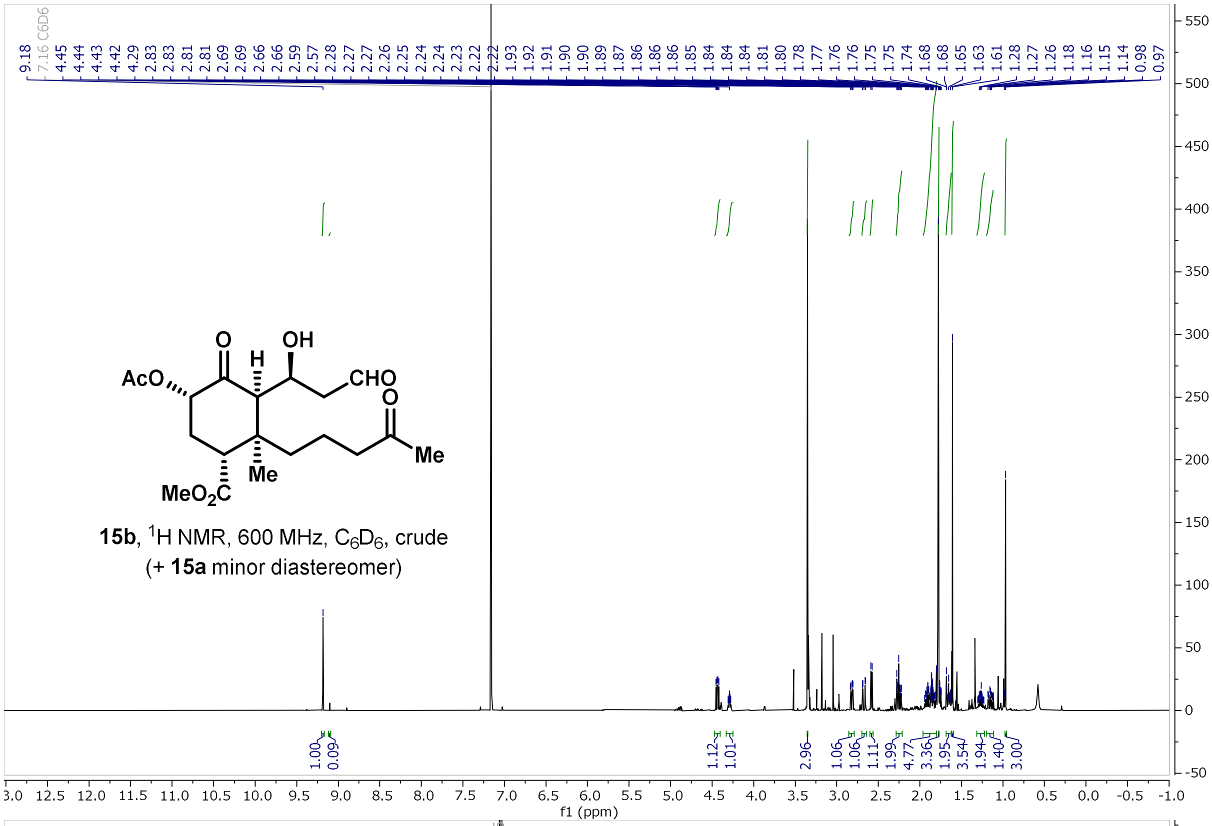
15b, ^{1}H NMR, 600 MHz, C_6D_6, crude
(+ 15a minor diastereomer)
AcO
O
H
OH
CHO
O
Me
Me
MeO_2C
f1 (ppm)

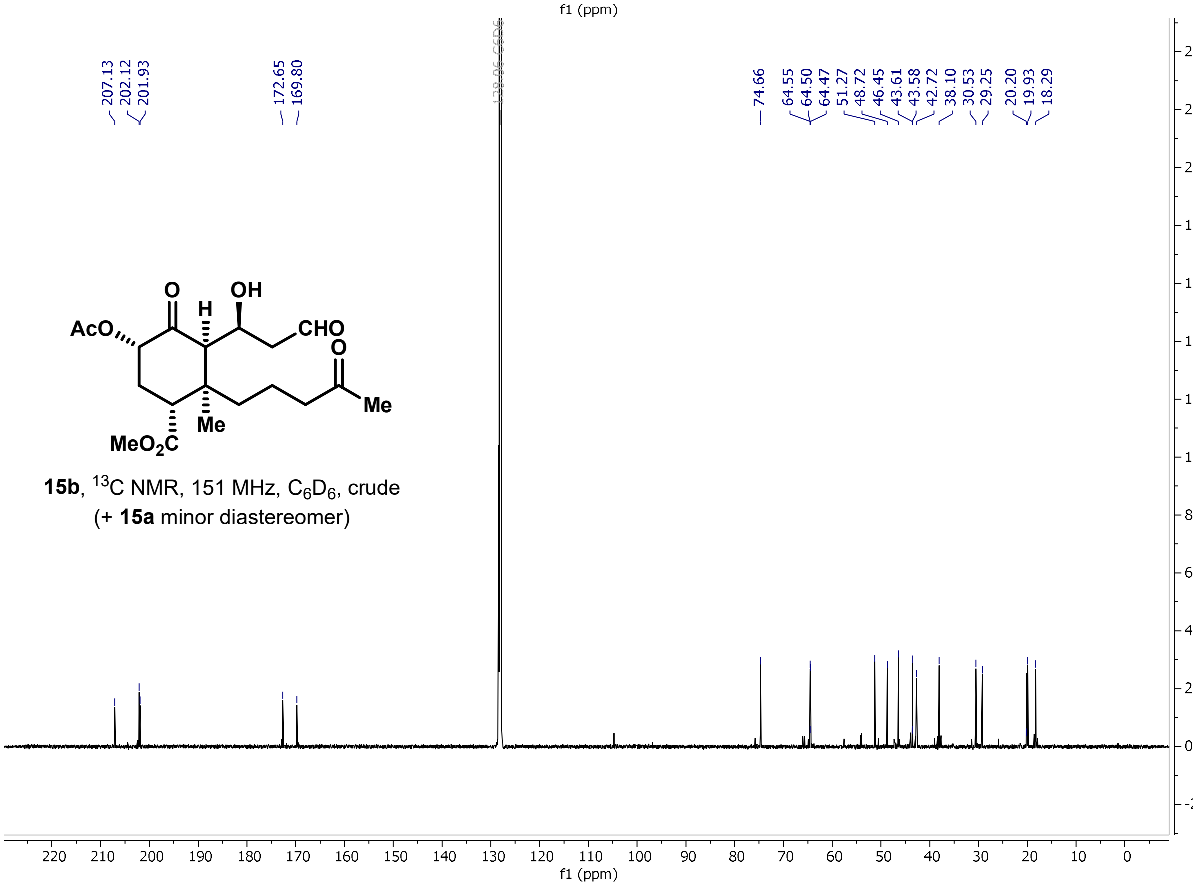
15b, ^{13}C NMR, 151 MHz, C_6D_6, crude
(+ 15a minor diastereomer)
AcO
O
H
OH
CHO
O
Me
Me
MeO_2C
207.13
202.12
201.93
172.65
169.80
74.66
64.55
64.50
64.47
51.27
48.72
46.45
43.61
43.58
42.72
38.10
30.53
29.25
20.20
19.93
18.29
f1 (ppm)

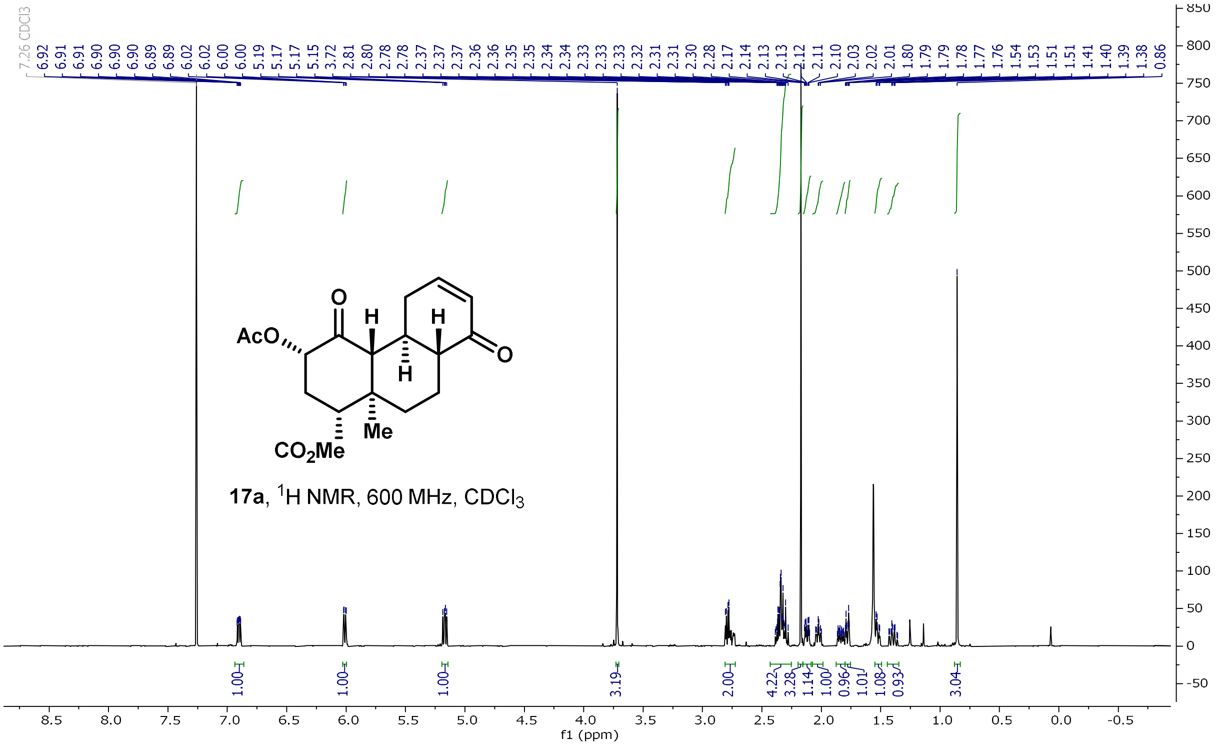

17a, ^{1}H NMR, 600 MHz, $CDCl_3$

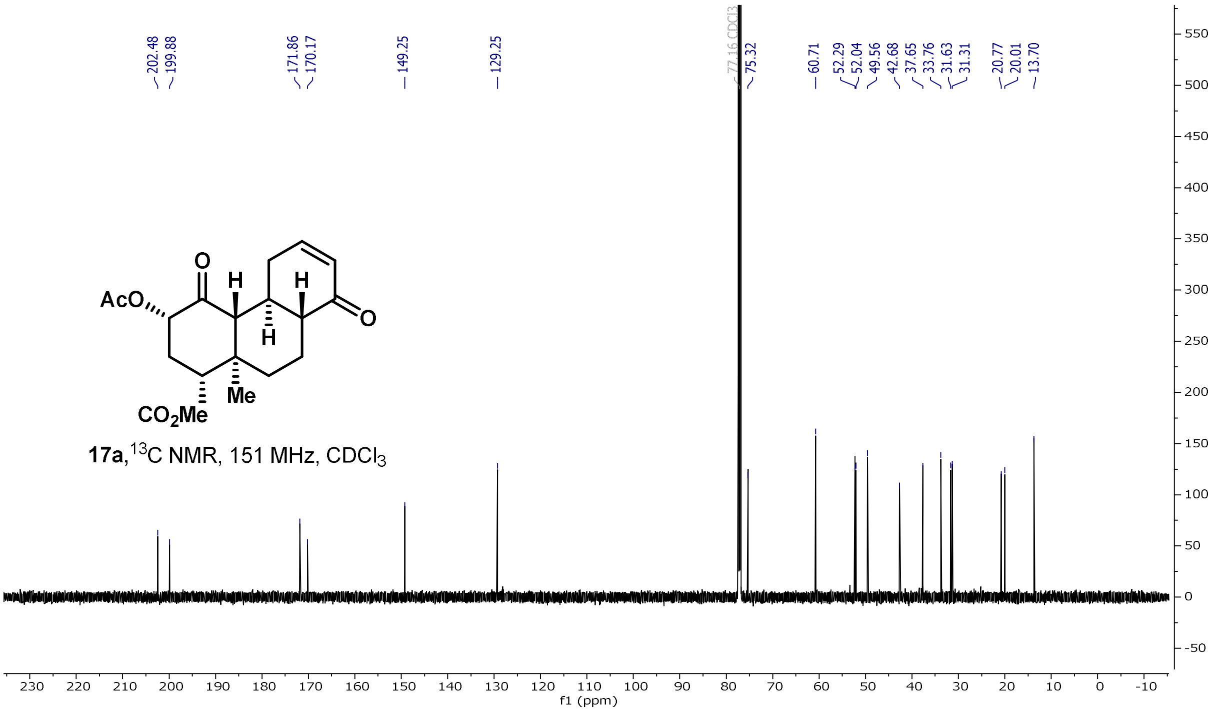

17a, ^{13}C NMR, 151 MHz, $CDCl_3$

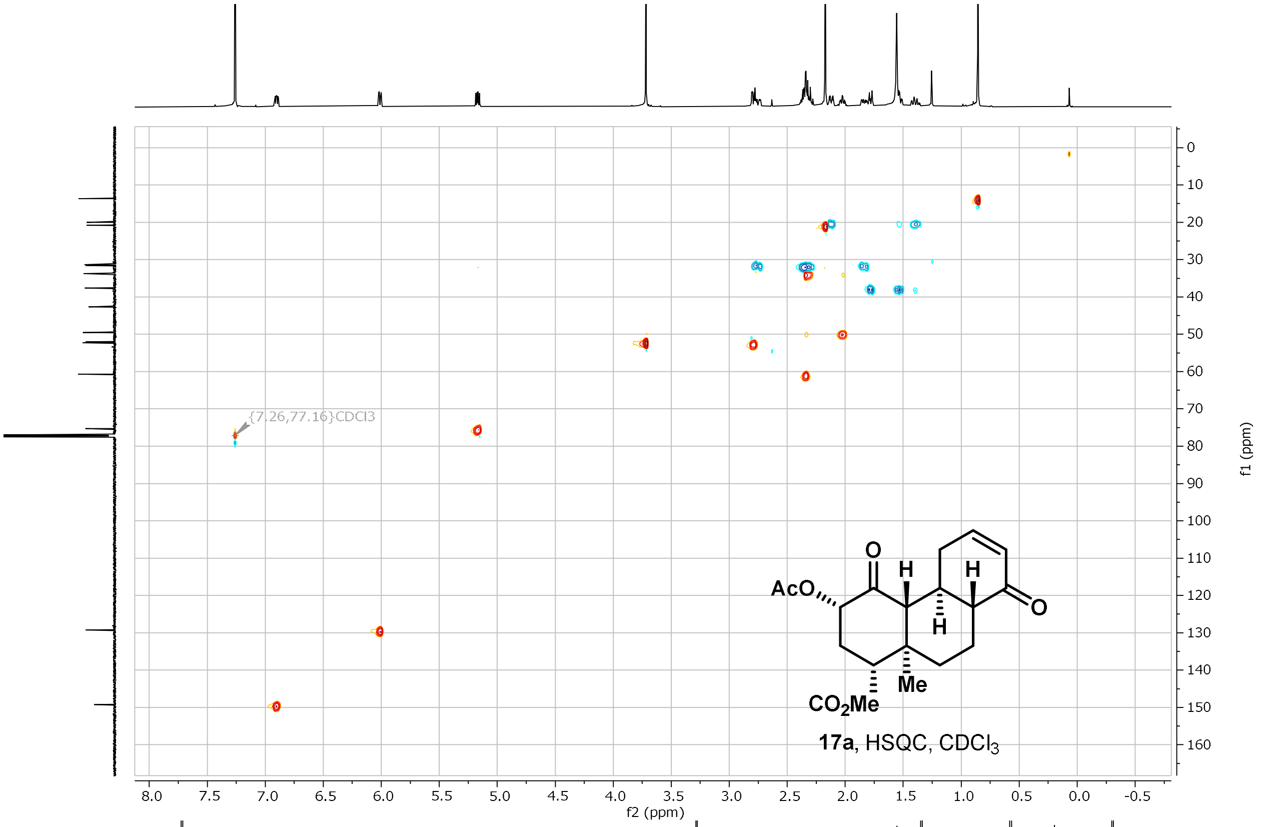

17a, HSQC, $CDCl_3$

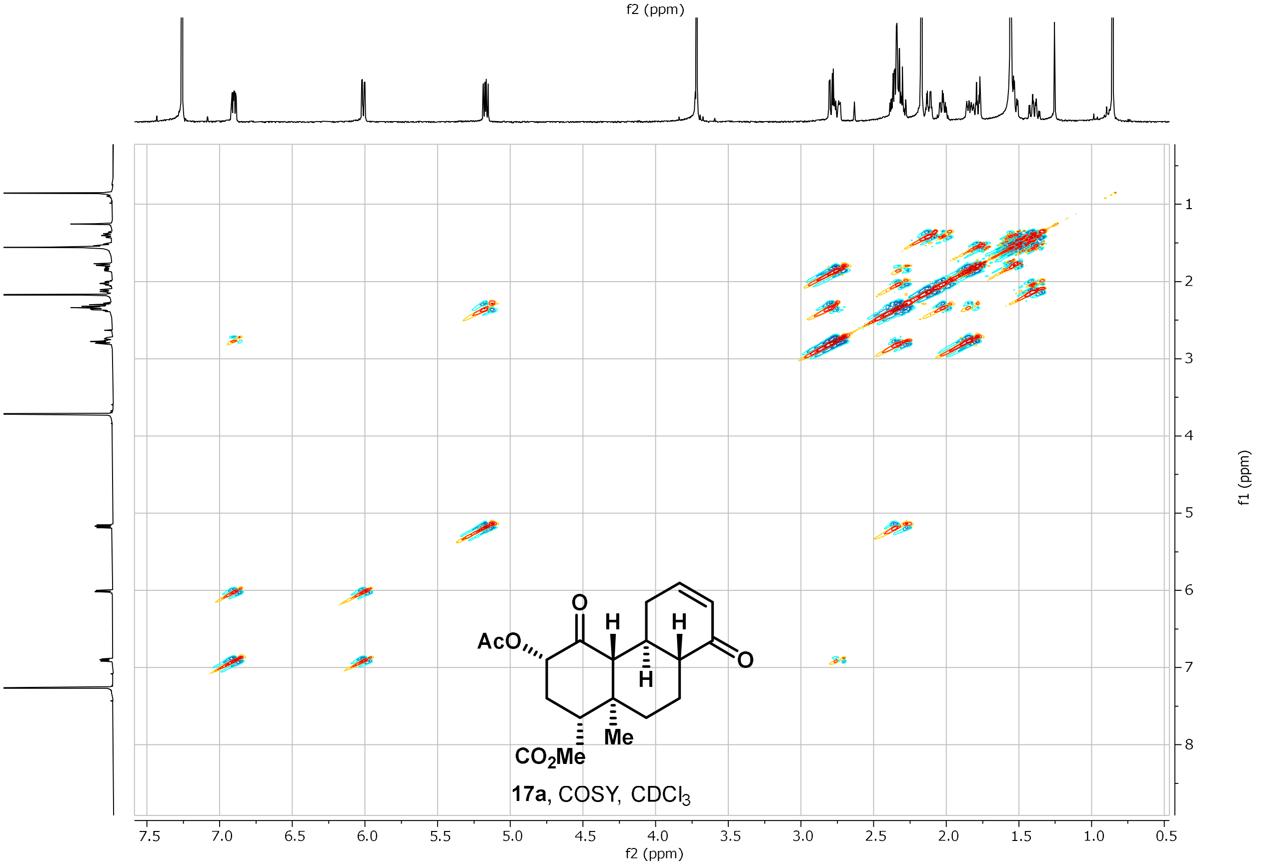

17a, COSY, $CDCl_3$

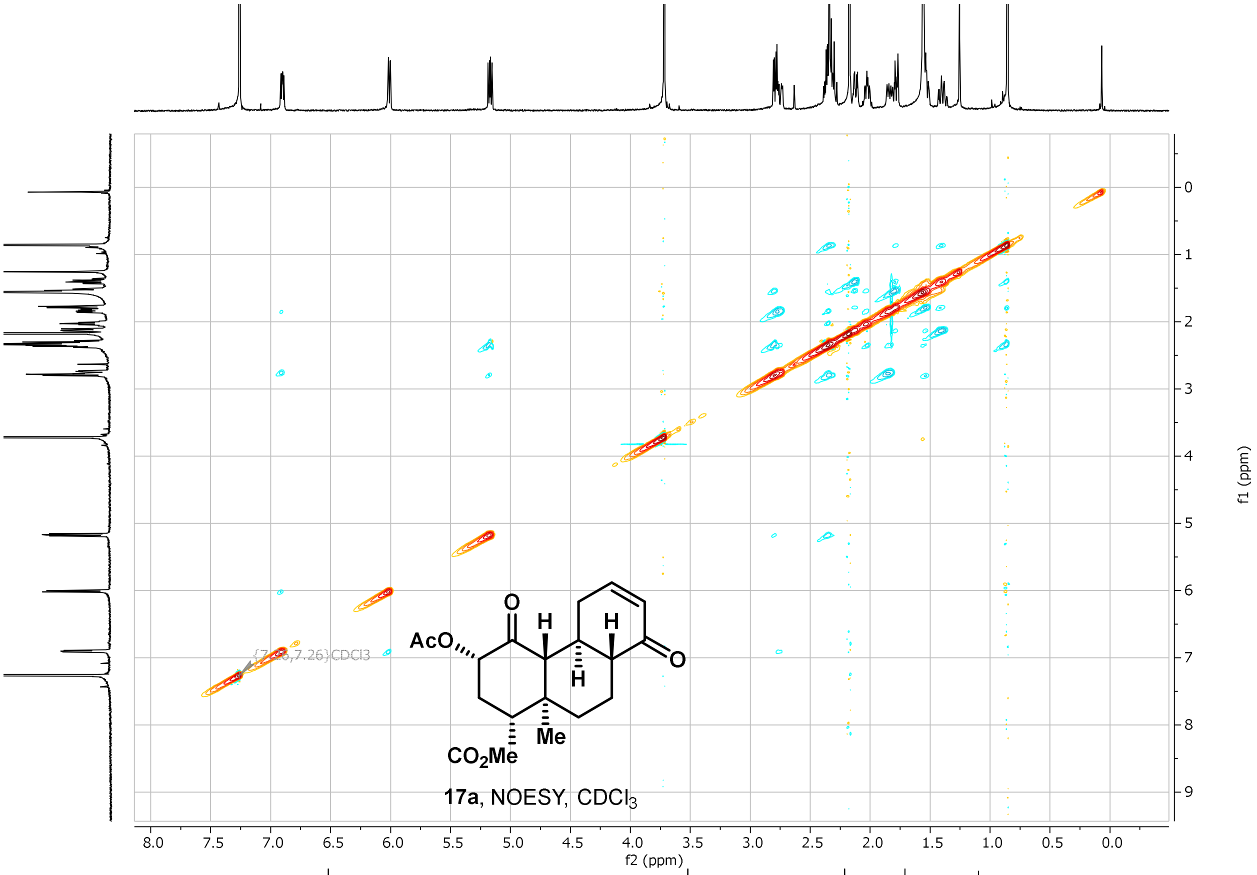
AcO
O
H
H
H
O
CO2Me
Me
17a, NOESY, CDCl3
f2 (ppm)
f1 (ppm)
{7.26,7.26} CDCl3

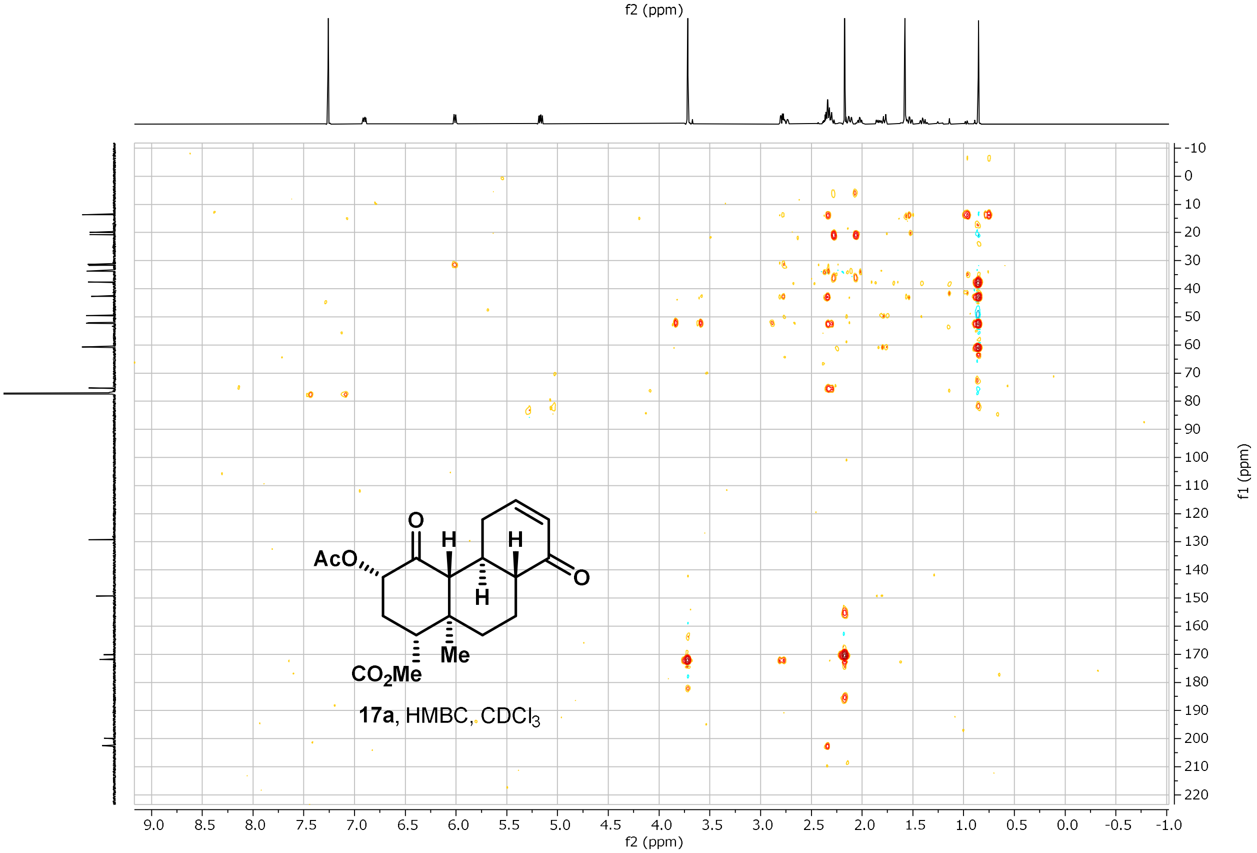
AcO
O
H
H
H
O
CO2Me
Me
17a, HMBC, CDCl3
f2 (ppm)
f1 (ppm)

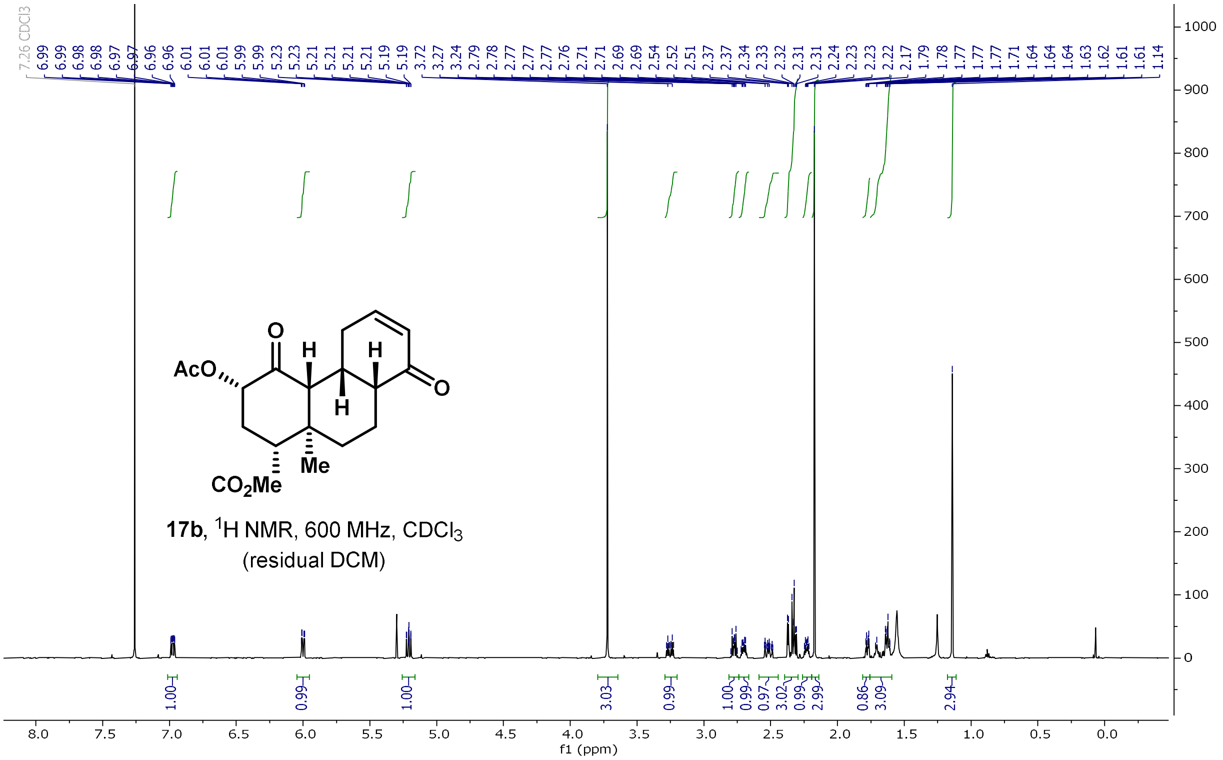

17b, ^{1}H NMR, 600 MHz, $CDCl_3$
(residual DCM)

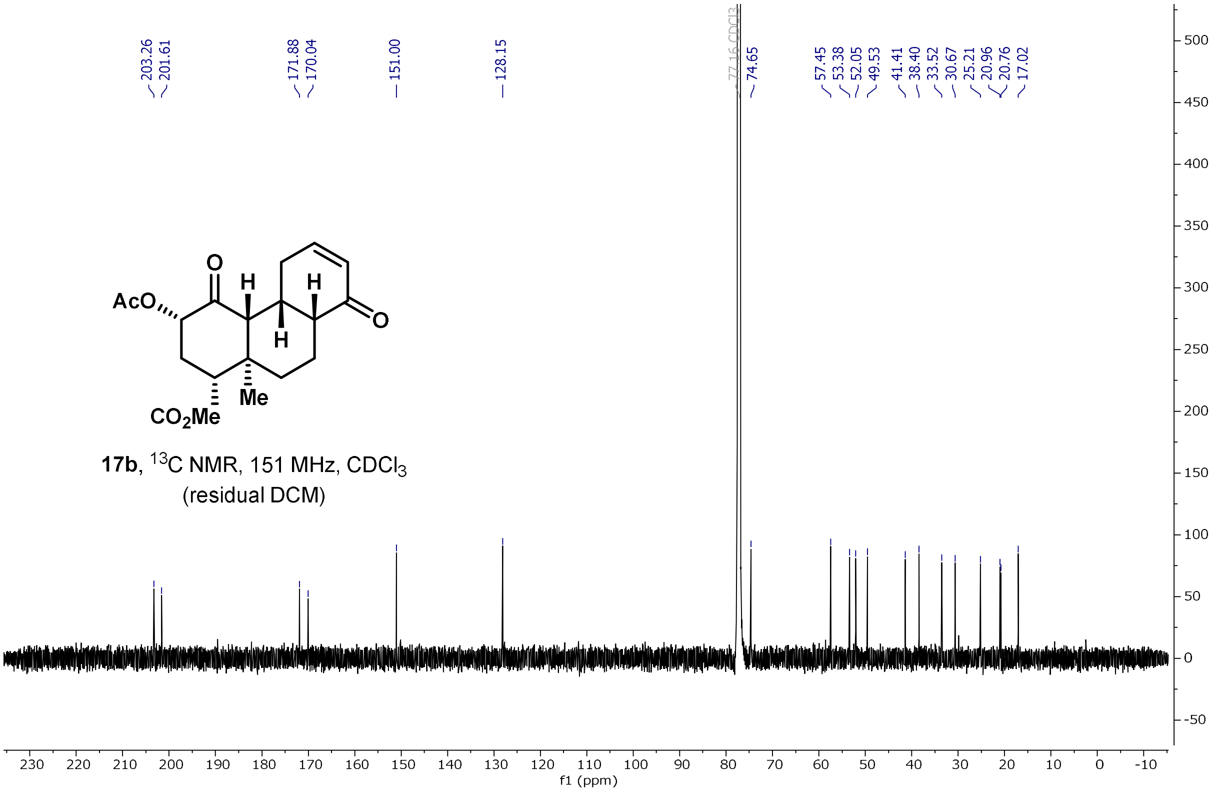

17b, ^{13}C NMR, 151 MHz, $CDCl_3$
(residual DCM)

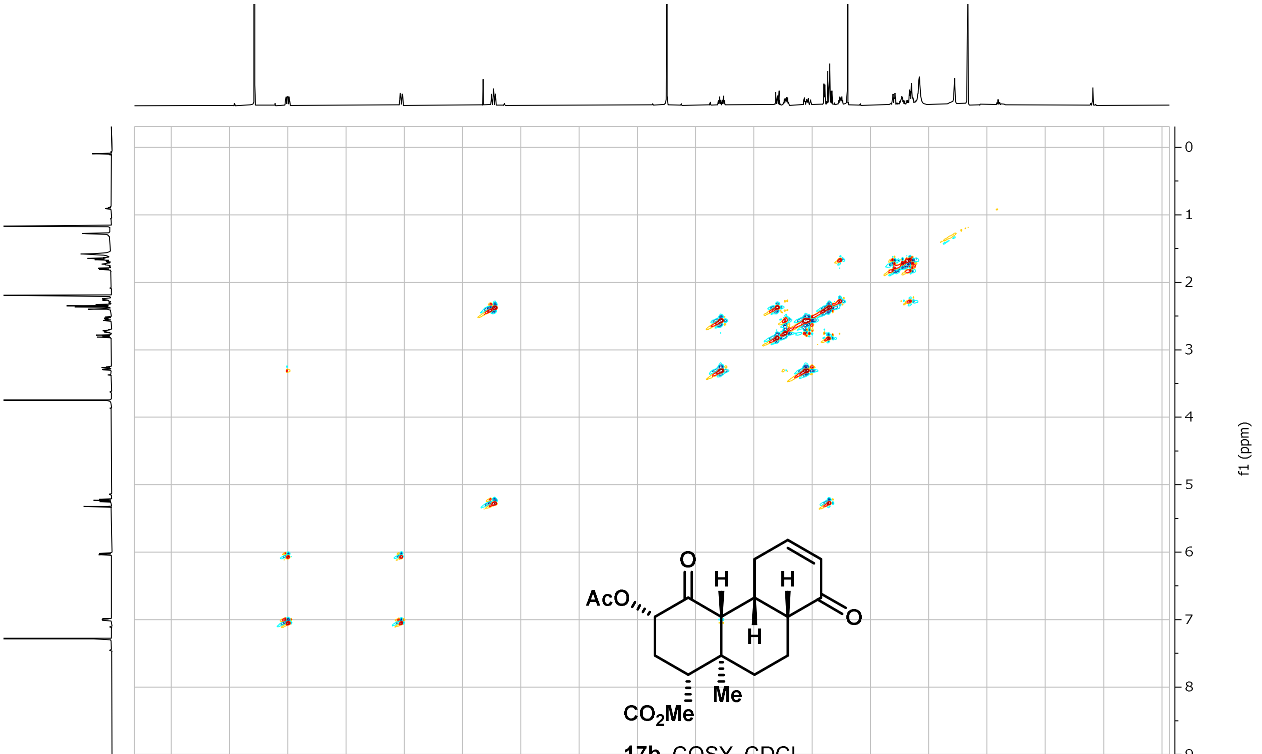

17b, COSY, $CDCl_3$

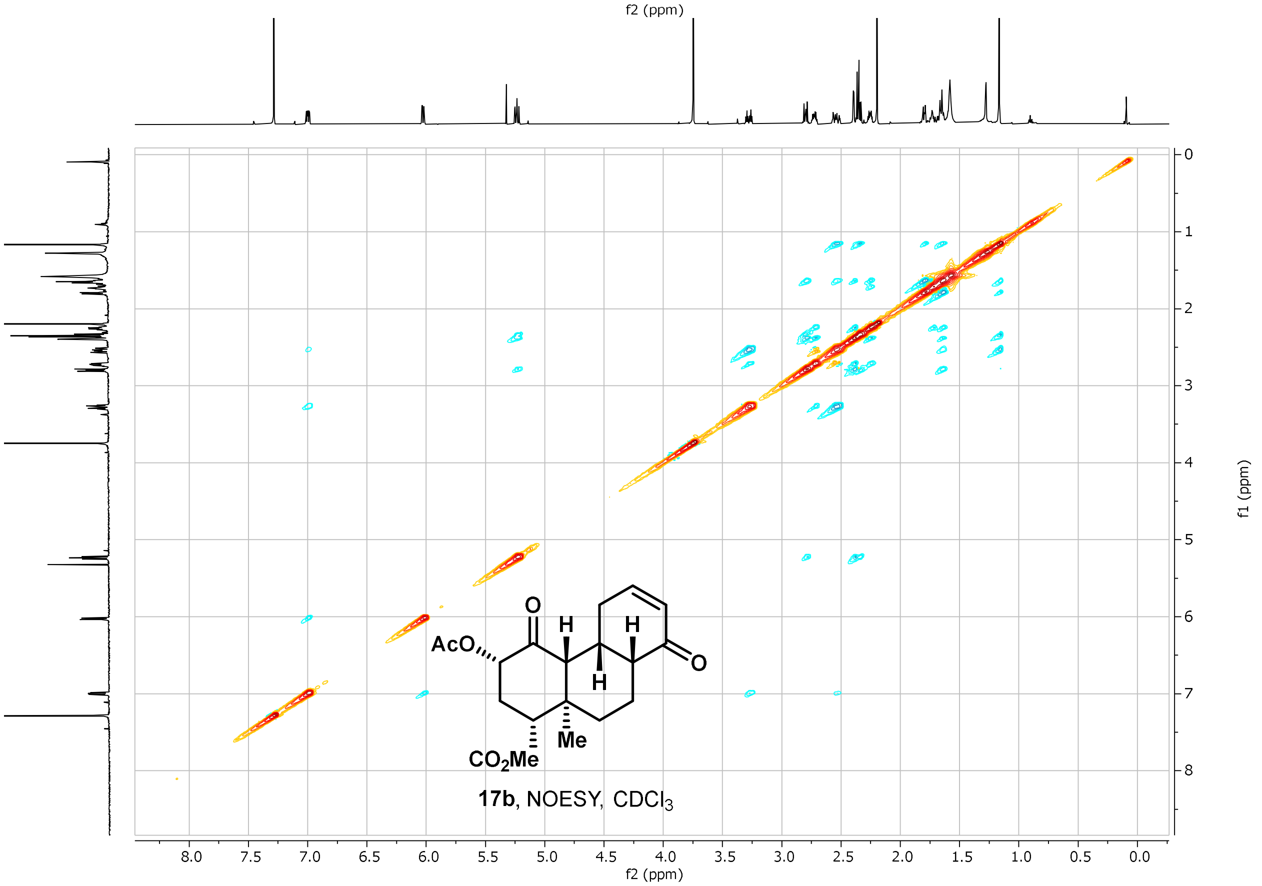

17b, NOESY, $CDCl_3$

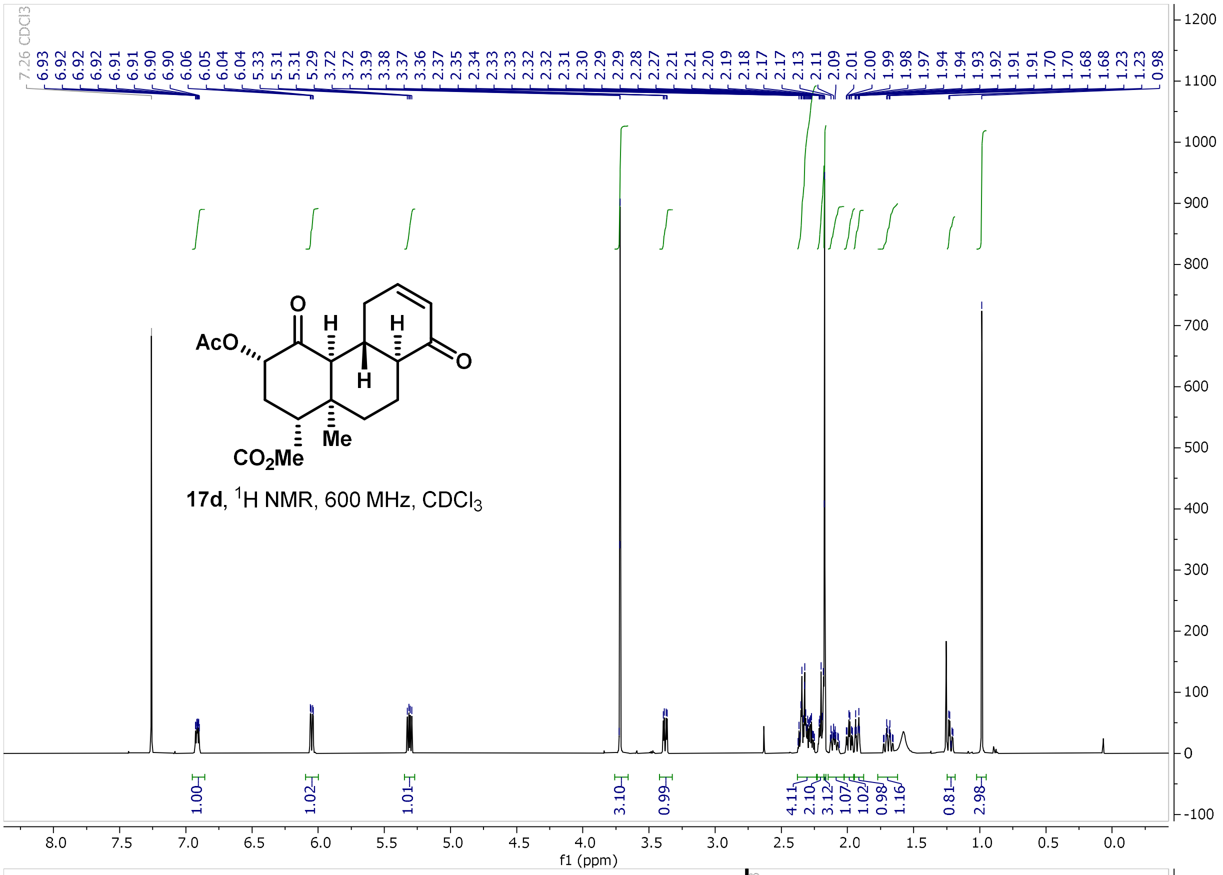
17d, ^{1}H NMR, 600 MHz, $CDCl_3$
AcO
CO2Me
Me
f1 (ppm)

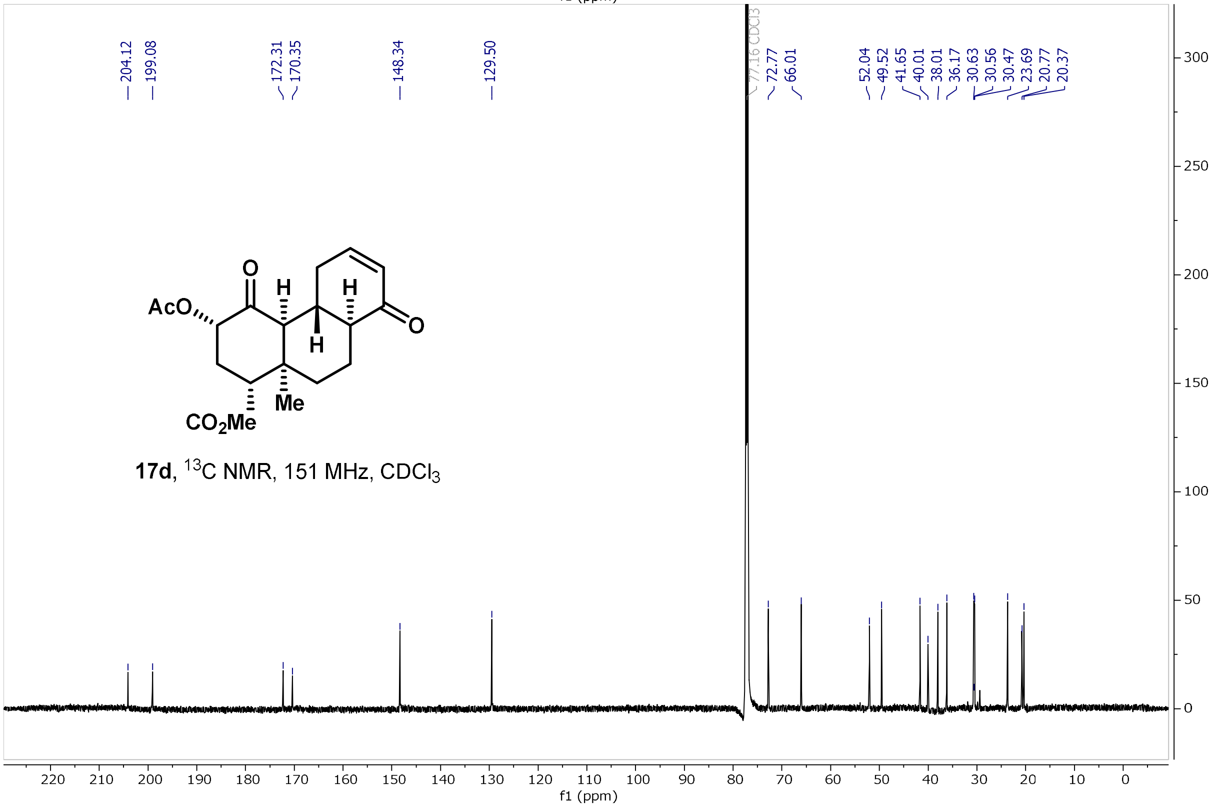
17d, ^{13}C NMR, 151 MHz, $CDCl_3$
AcO
CO2Me
Me
204.12
199.08
172.31
170.35
148.34
129.50
77.16 CDCl3
72.77
66.01
52.04
49.52
41.65
40.01
38.01
36.17
30.63
30.56
30.47
23.69
20.77
20.37
f1 (ppm)

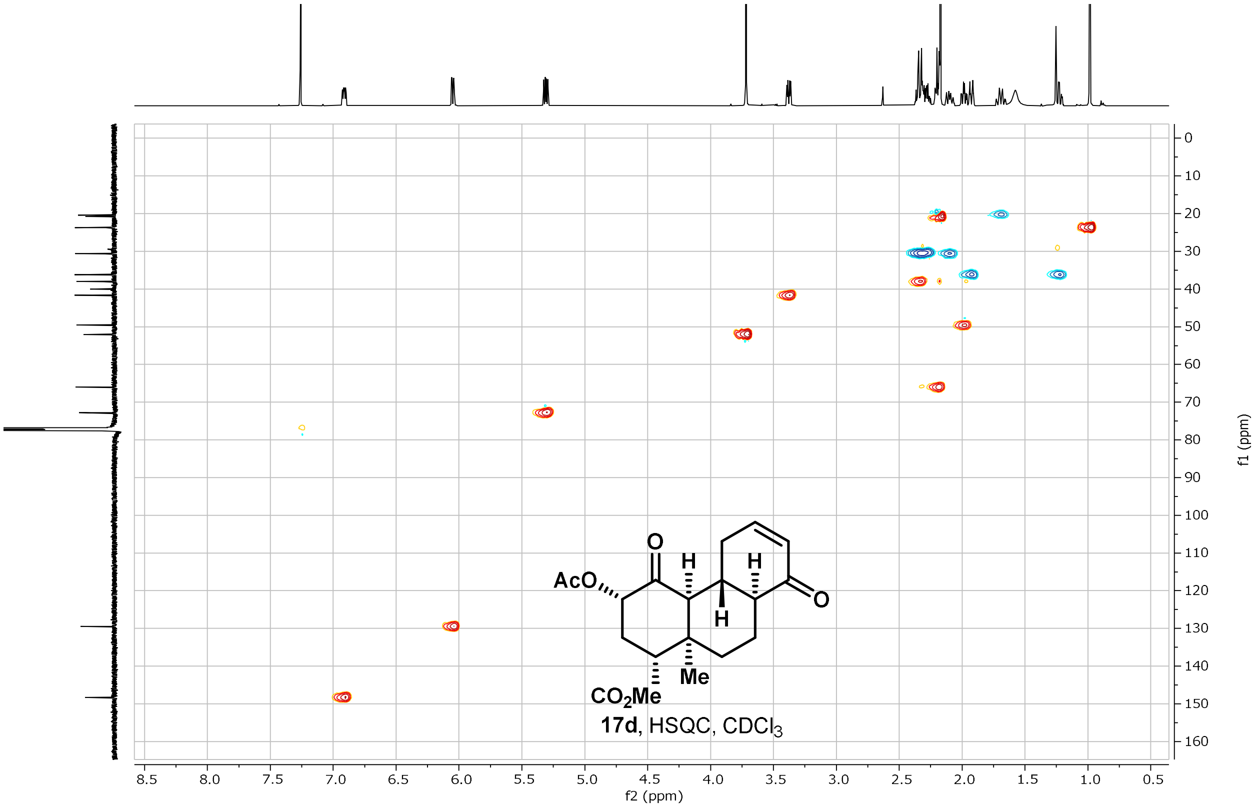
AcO
O
H
H
H
O
Me
CO2Me
17d, HSQC, CDCl3
f2 (ppm)
f1 (ppm)

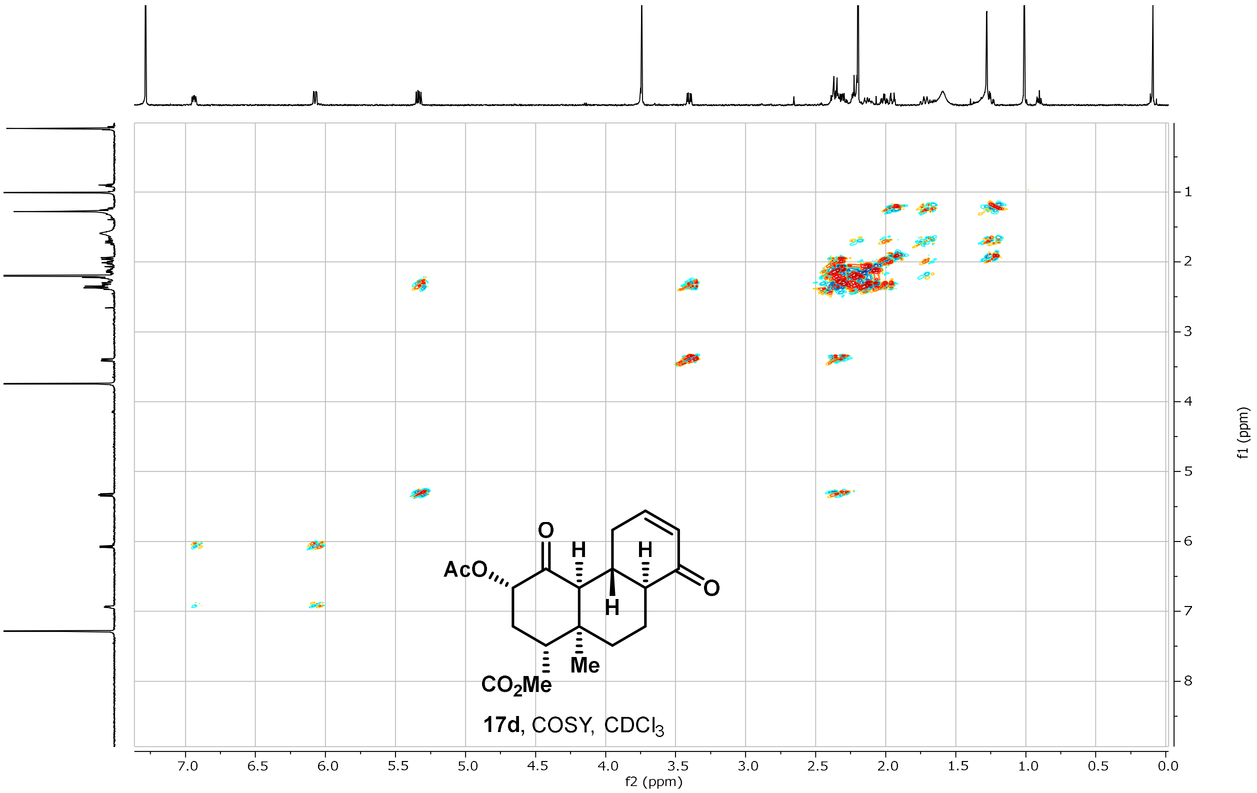
AcO
O
H
H
H
O
Me
CO2Me
17d, COSY, CDCl3
f2 (ppm)
f1 (ppm)

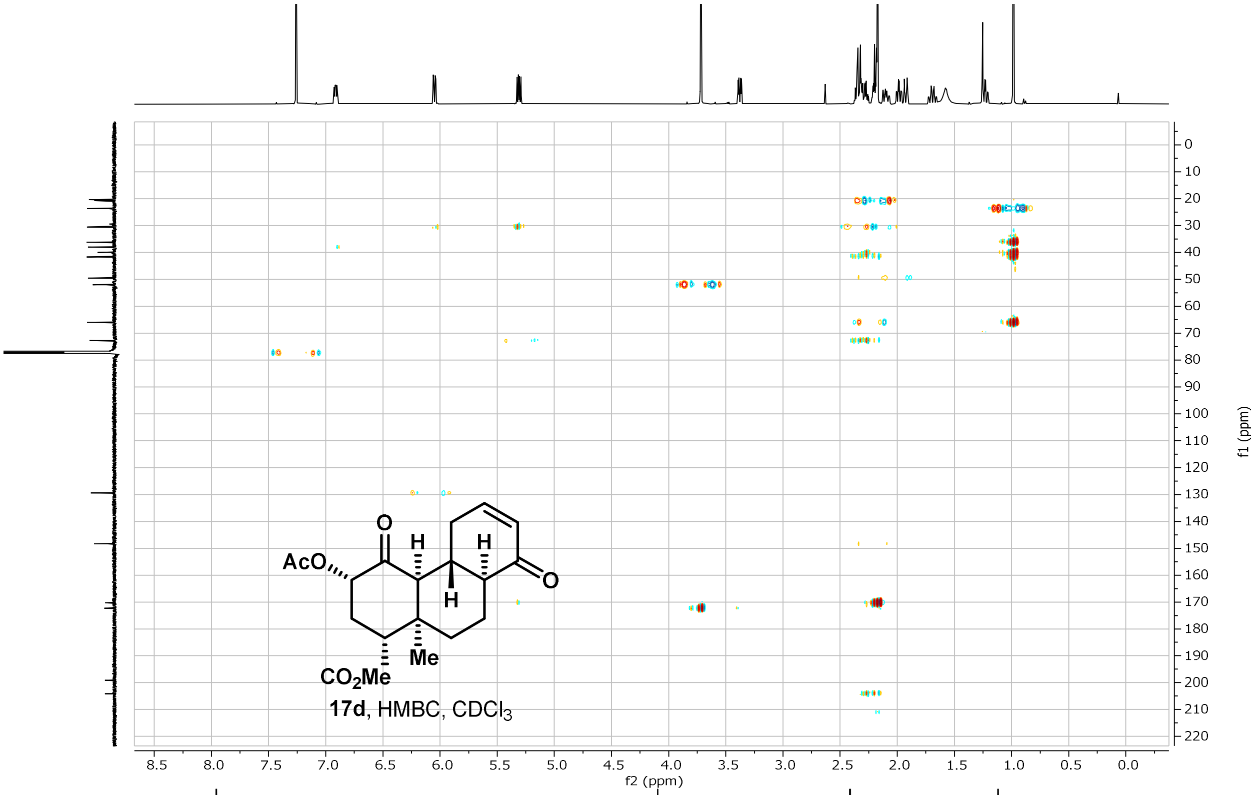
AcO
O
H
H
H
O
Me
CO_2Me
17d, HMBC, $CDCl_3$
f1 (ppm)
f2 (ppm)

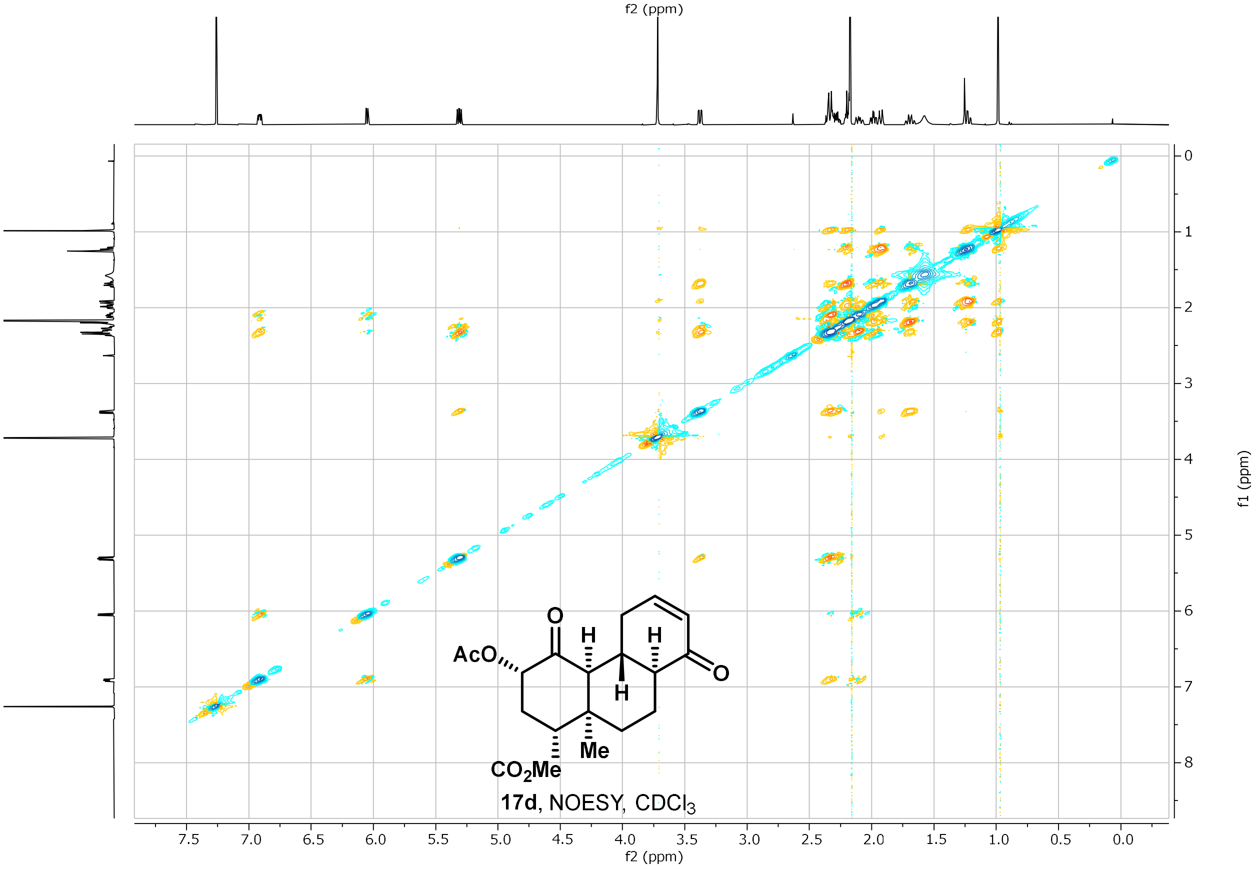
AcO
O
H
H
H
O
Me
CO_2Me
17d, NOESY, $CDCl_3$
f1 (ppm)
f2 (ppm)

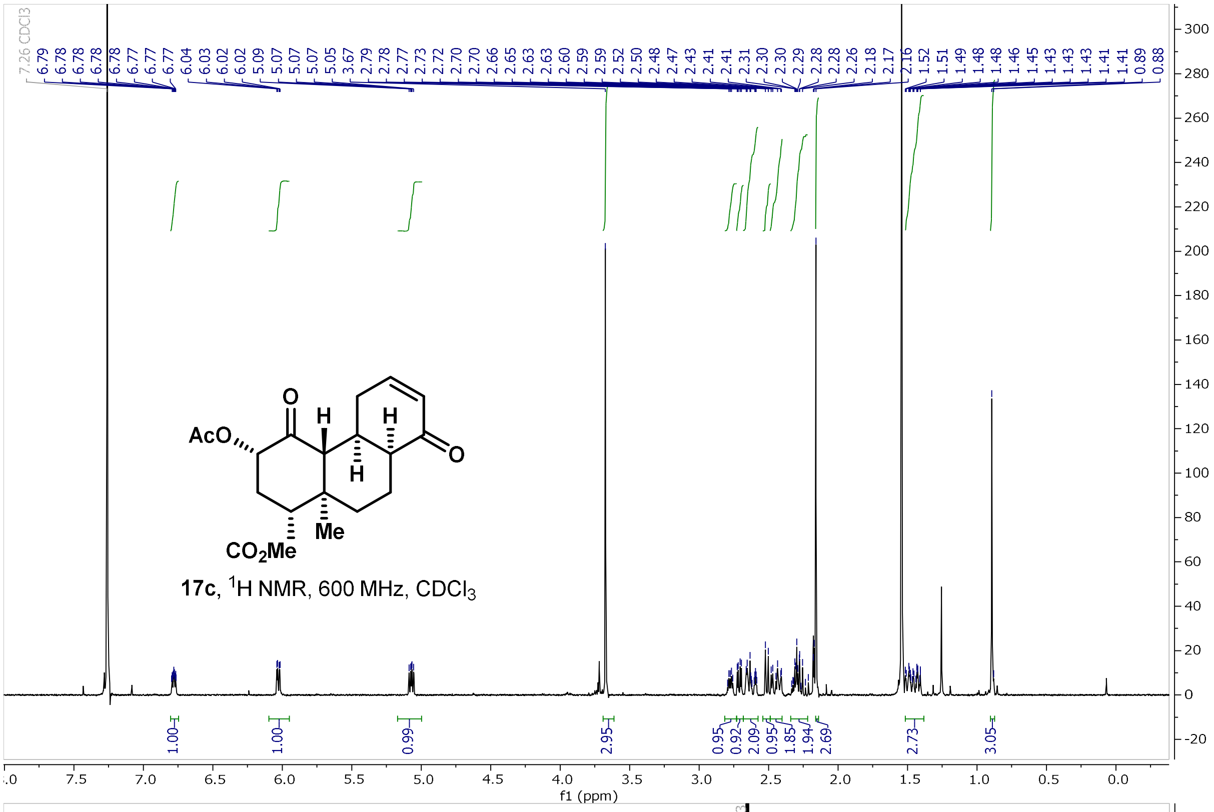
17c, 1H NMR, 600 MHz, CDCl3
7.26 CDCl3
6.79 6.78 6.78 6.78 6.78 6.77 6.77 6.77 6.04 6.03 6.02 6.02 5.09 5.07 5.07 5.07 5.05 3.67 2.79 2.78 2.77 2.73 2.72 2.70 2.70 2.66 2.65 2.63 2.63 2.60 2.59 2.59 2.52 2.50 2.48 2.47 2.43 2.41 2.41 2.31 2.30 2.30 2.29 2.28 2.28 2.26 2.18 2.17 2.16 1.52 1.51 1.49 1.48 1.48 1.46 1.45 1.43 1.43 1.43 1.41 1.41 0.89 0.88
1.00 1.00 0.99 2.95 0.95 0.92 2.09 0.95 0.95 1.85 1.94 2.69 2.73 3.05
f1 (ppm)

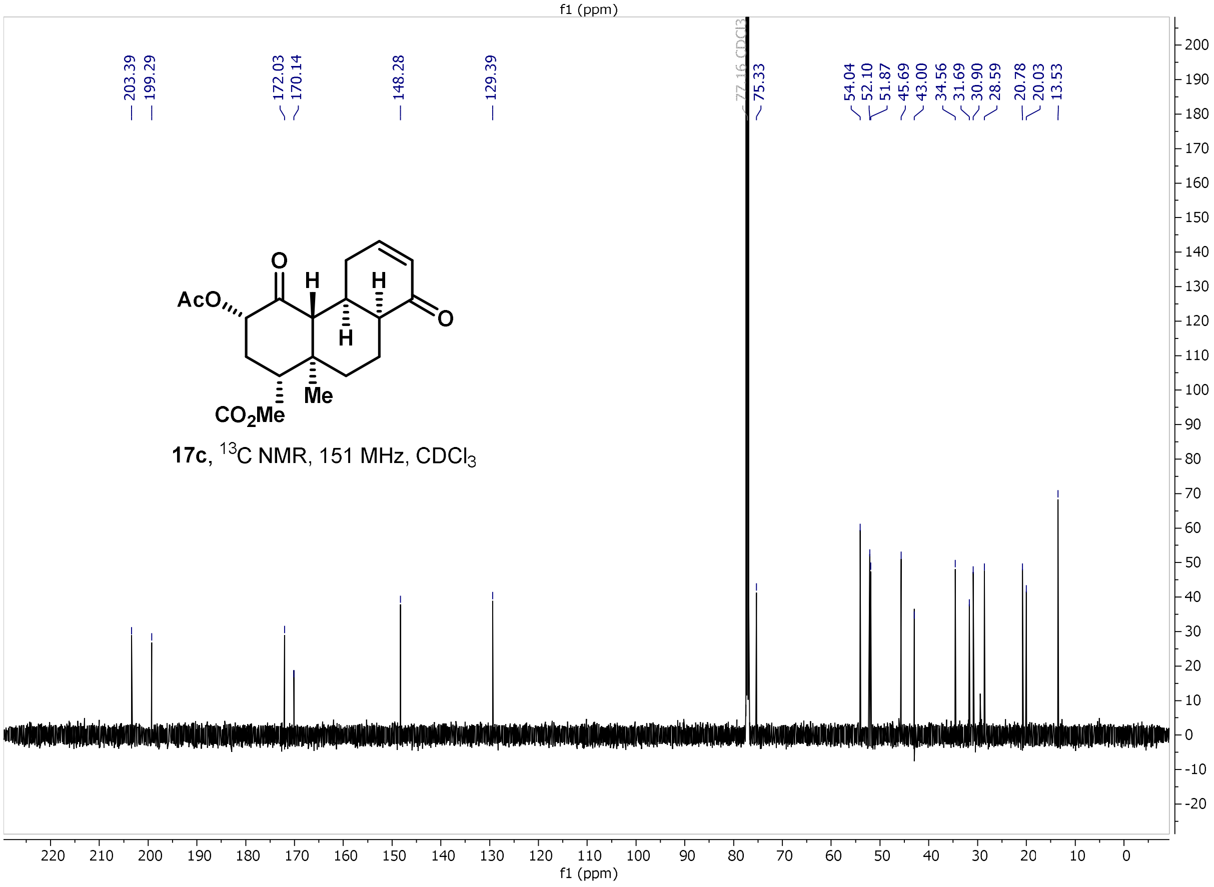
17c, 13C NMR, 151 MHz, CDCl3
203.39 199.29 172.03 170.14 148.28 129.39 77.16 CDCl3 75.33 54.04 52.10 51.87 45.69 43.00 34.56 31.69 30.90 28.59 20.78 20.03 13.53
f1 (ppm)

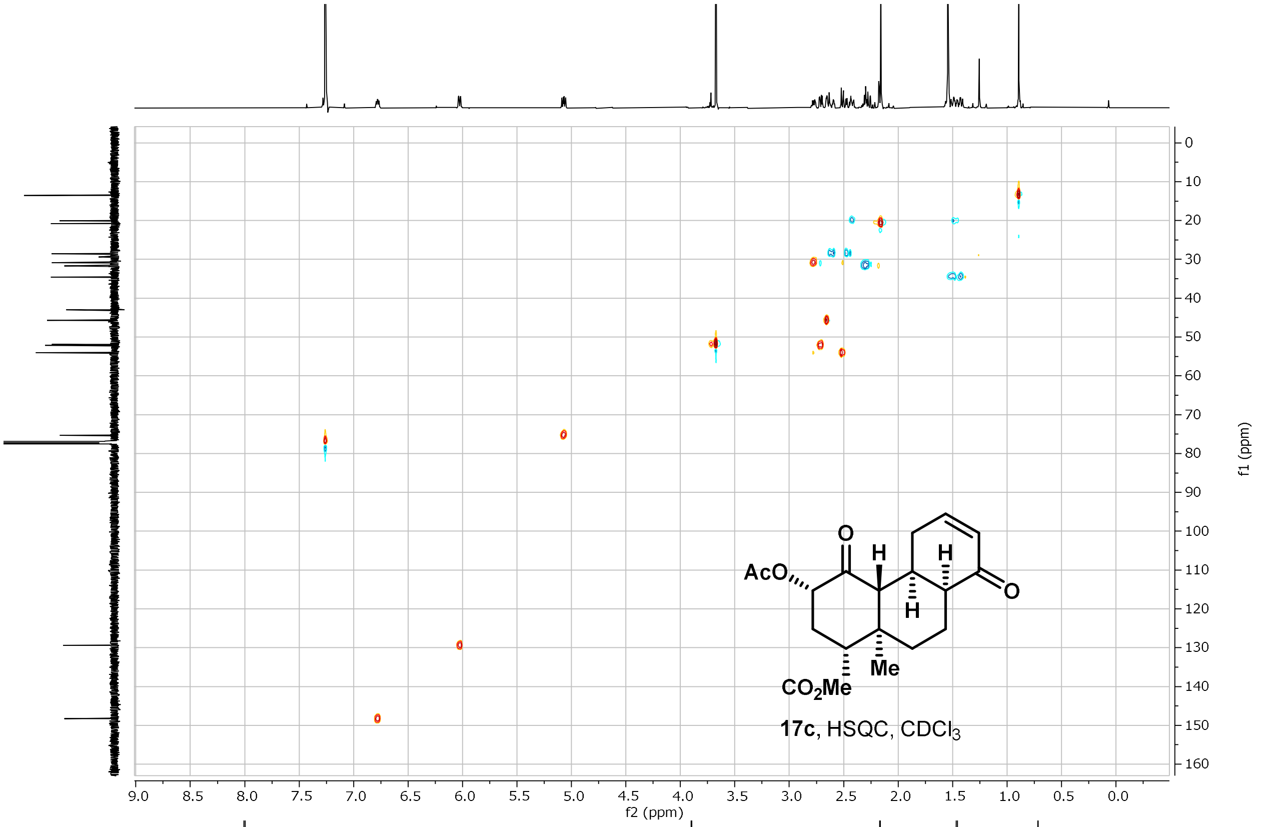
AcO
H
H
H
O
O
Me
CO_2Me
17c, HSQC, $CDCl_3$
f1 (ppm)
f2 (ppm)

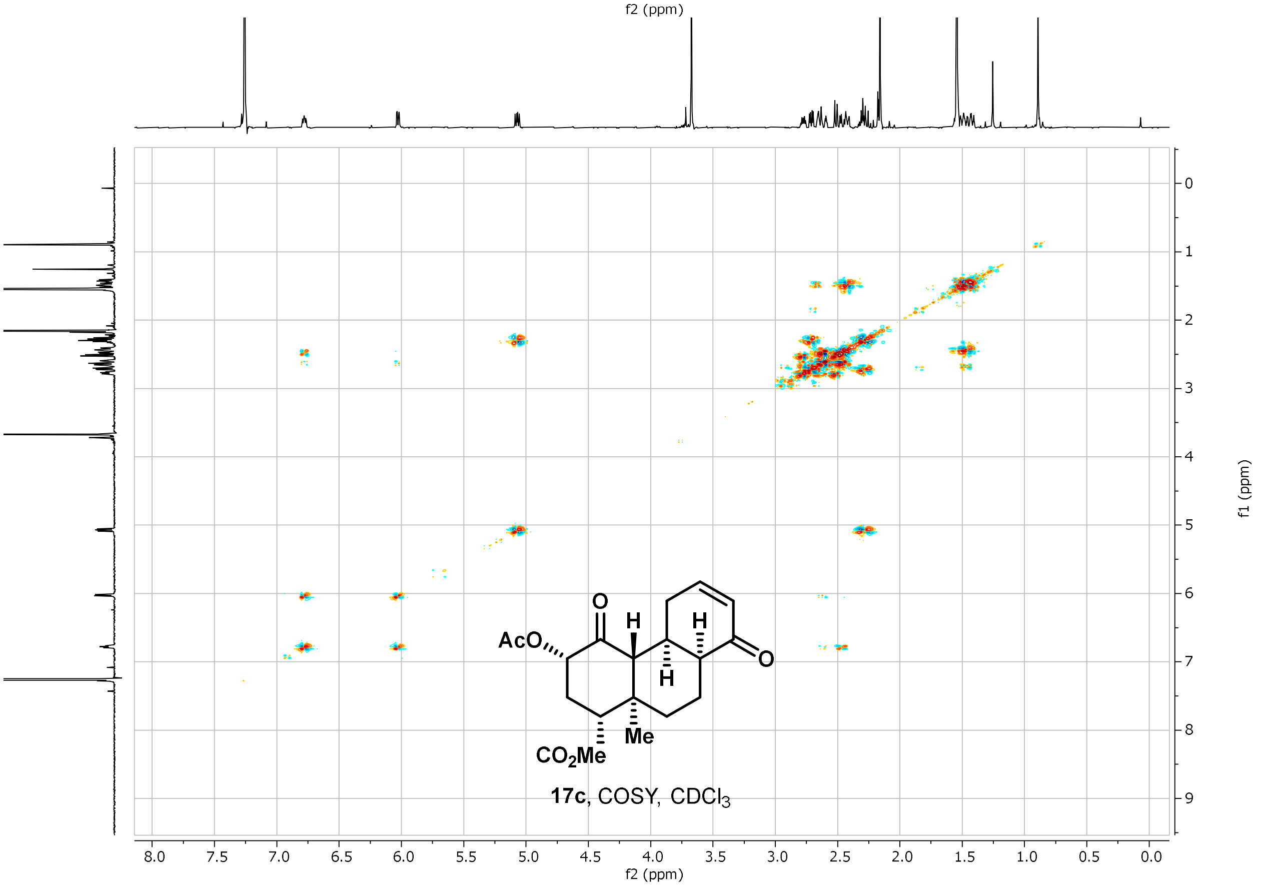
AcO
H
H
H
O
O
Me
CO_2Me
17c, COSY, $CDCl_3$
f1 (ppm)
f2 (ppm)

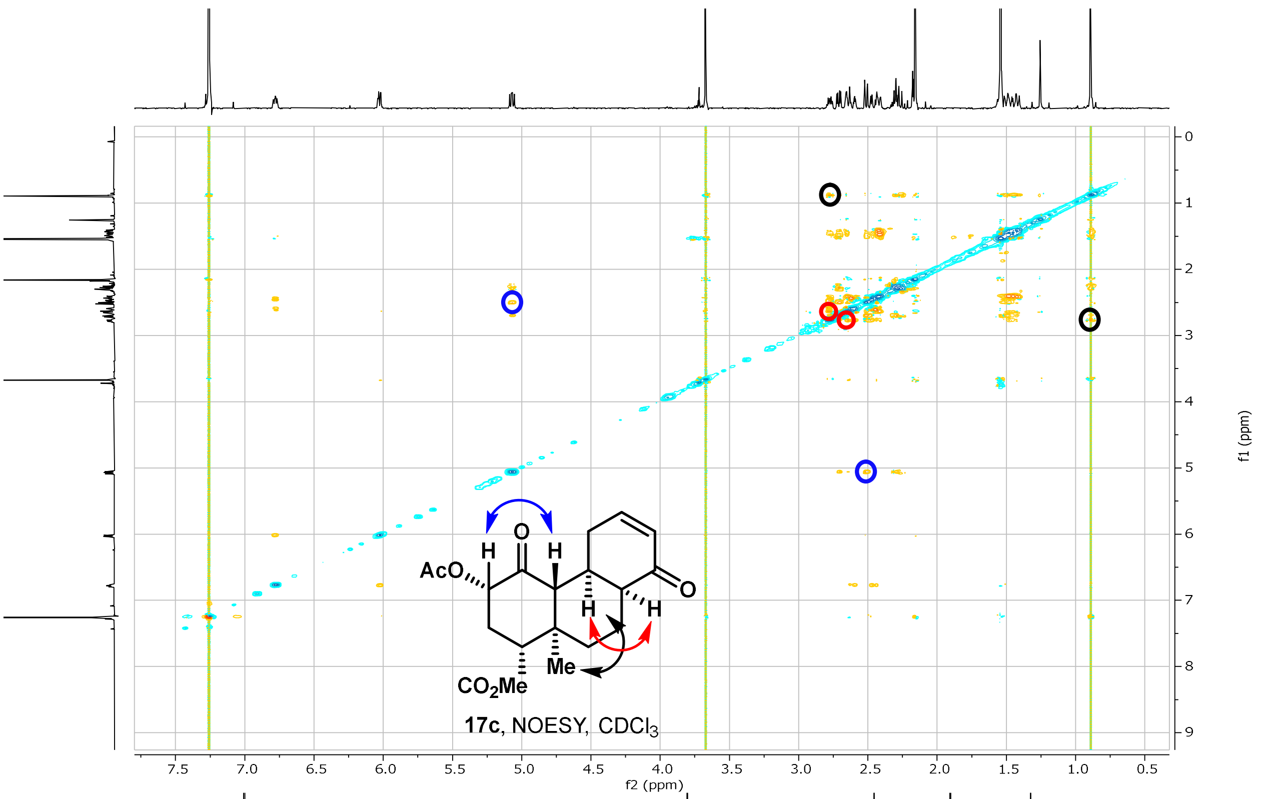

17c, NOESY, CDCl$_3$

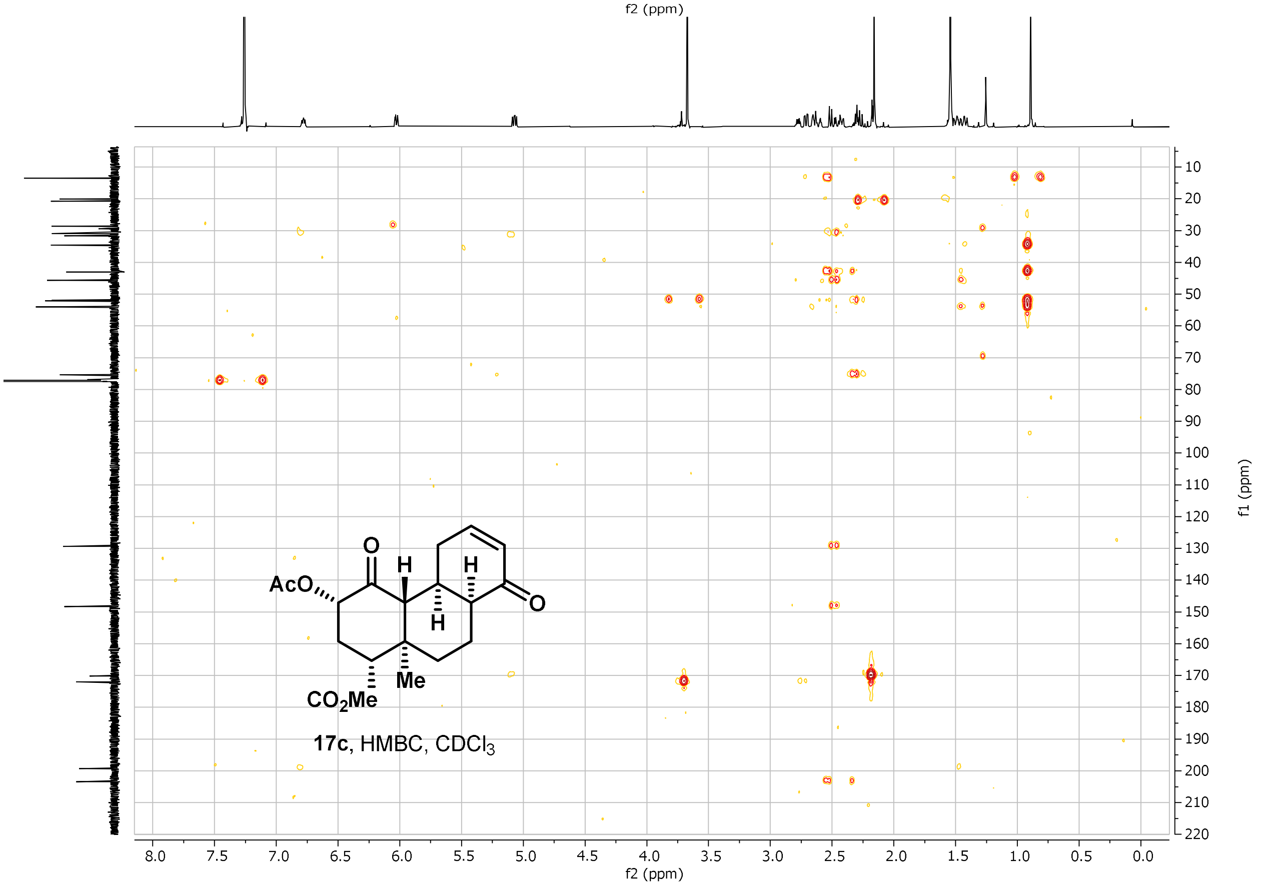

17c, HMBC, CDCl$_3$

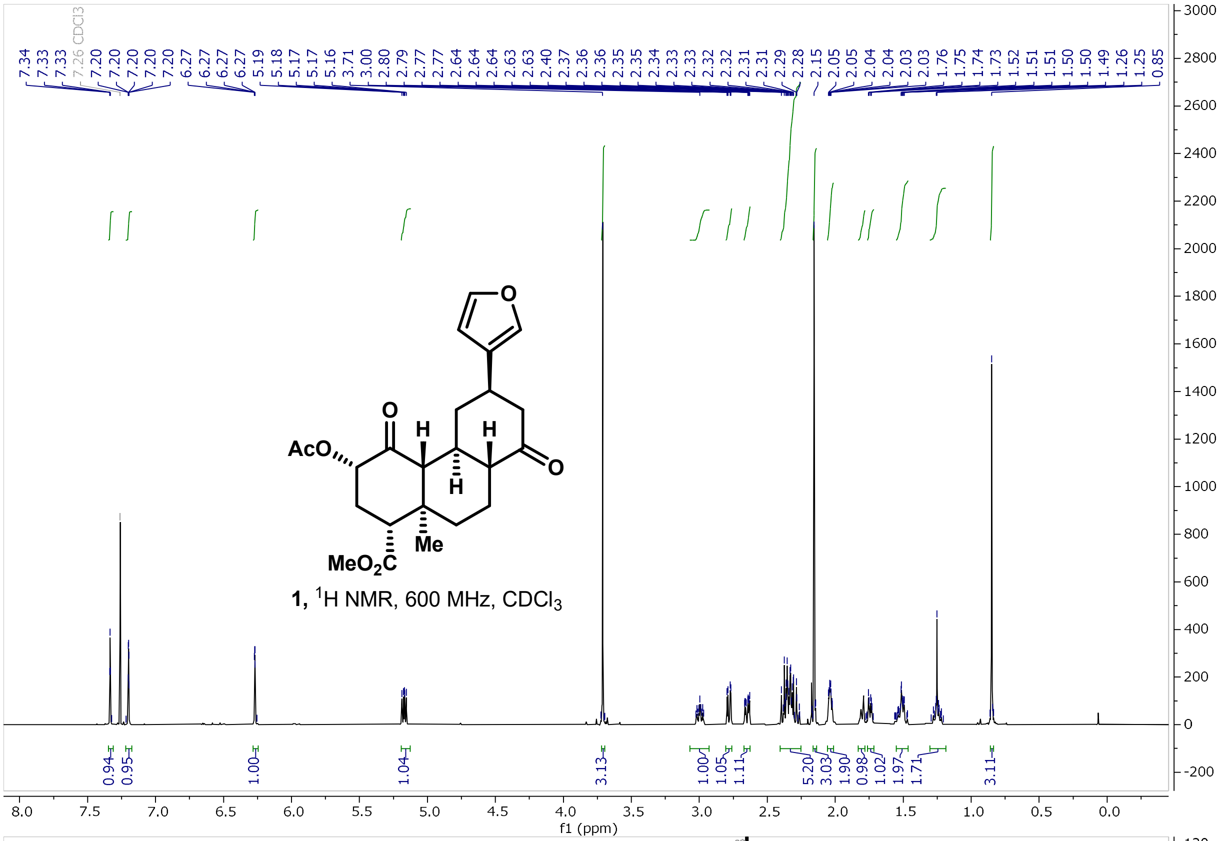

1, ¹H NMR, 600 MHz, CDCl3
f1 (ppm)

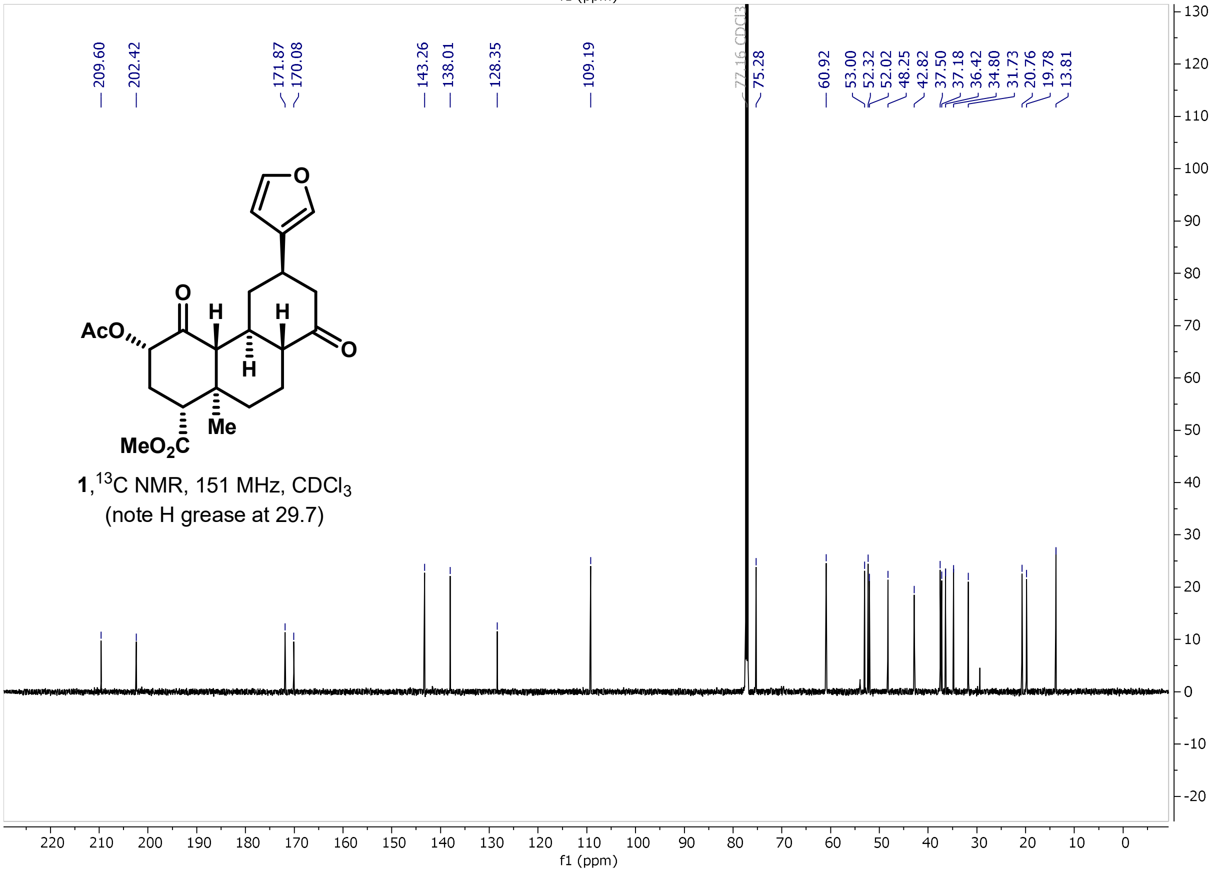

1,¹³C NMR, 151 MHz, CDCl3
(note H grease at 29.7)
f1 (ppm)

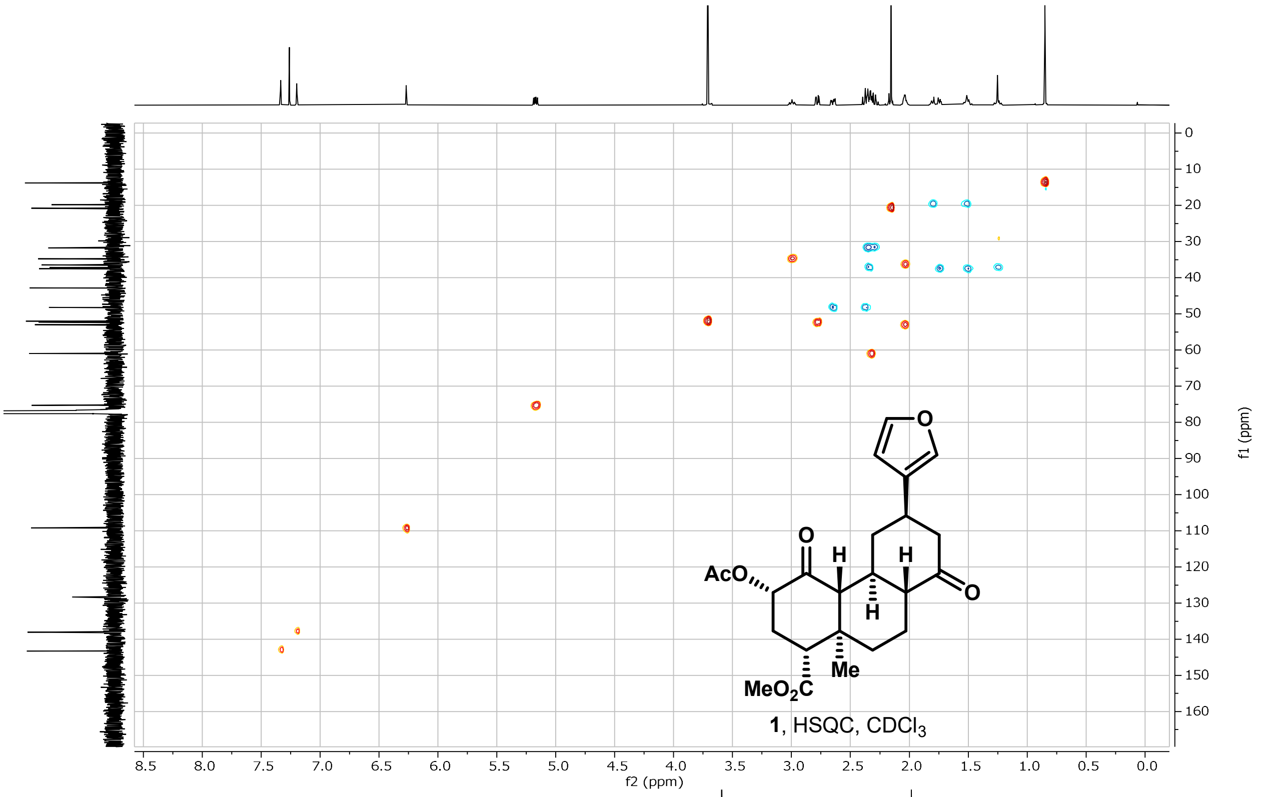

AcO
O
H
H
H
O
O
Me
MeO2C
1, HSQC, CDCl3
f1 (ppm)
f2 (ppm)

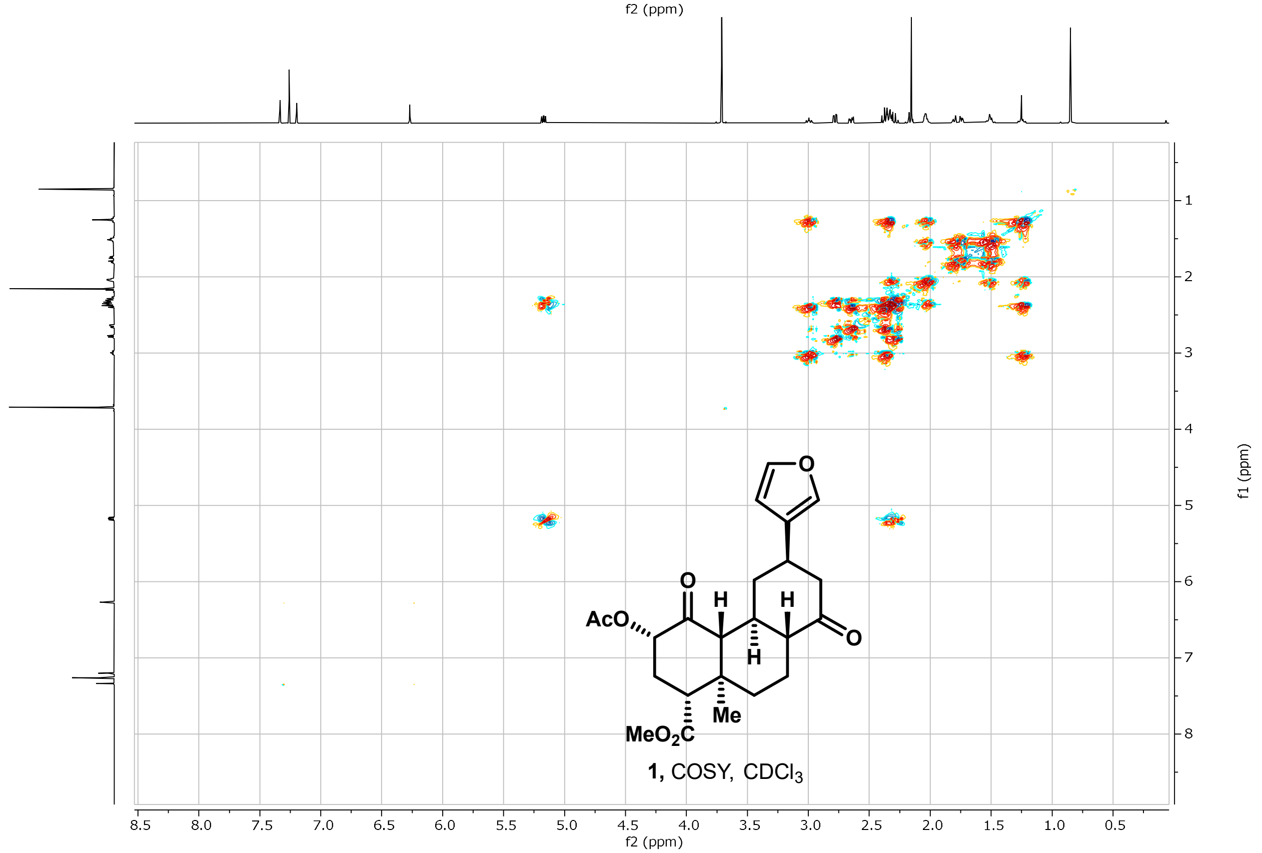

AcO
O
H
H
H
O
O
Me
MeO2C
1, COSY, CDCl3
f1 (ppm)
f2 (ppm)

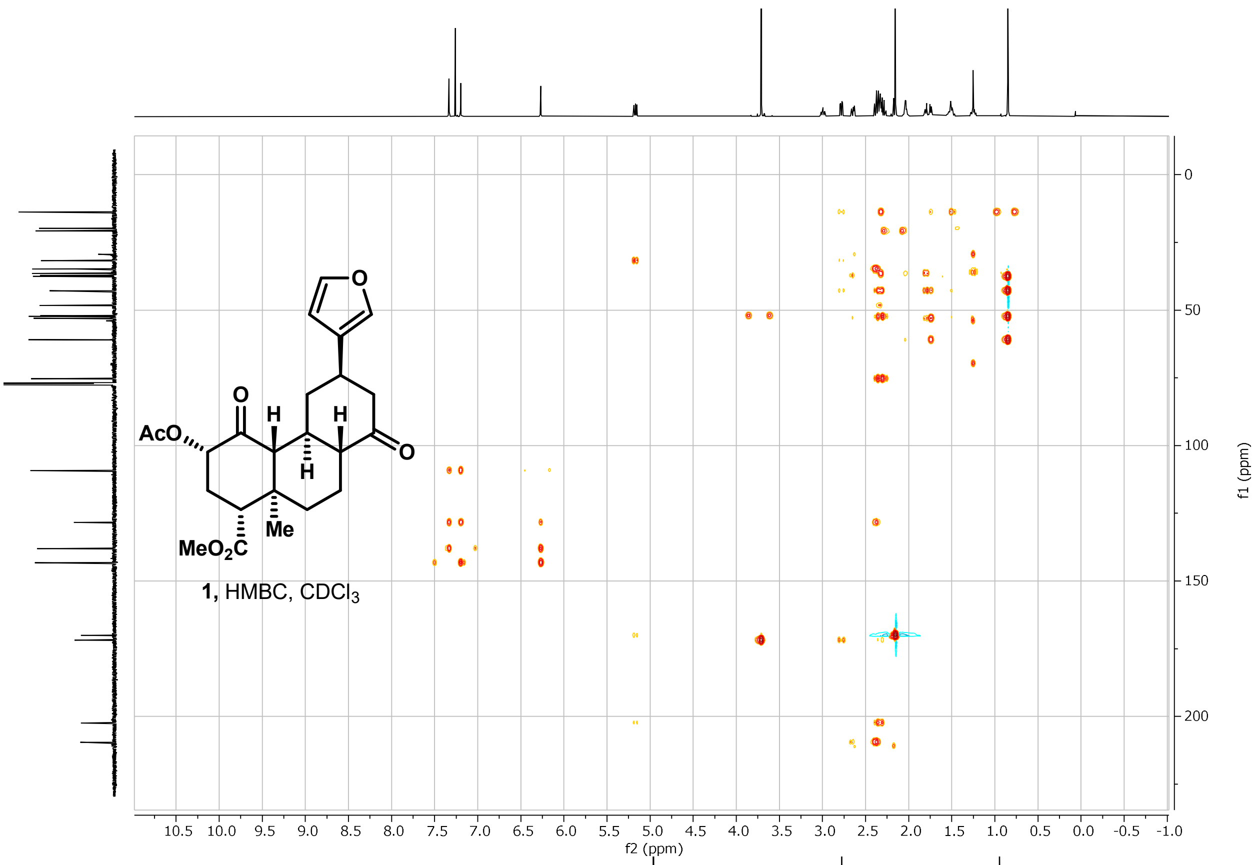

AcO
O
H
H
H
O
O
Me
MeO2C
1, HMBC, CDCl3
f2 (ppm)
f1 (ppm)

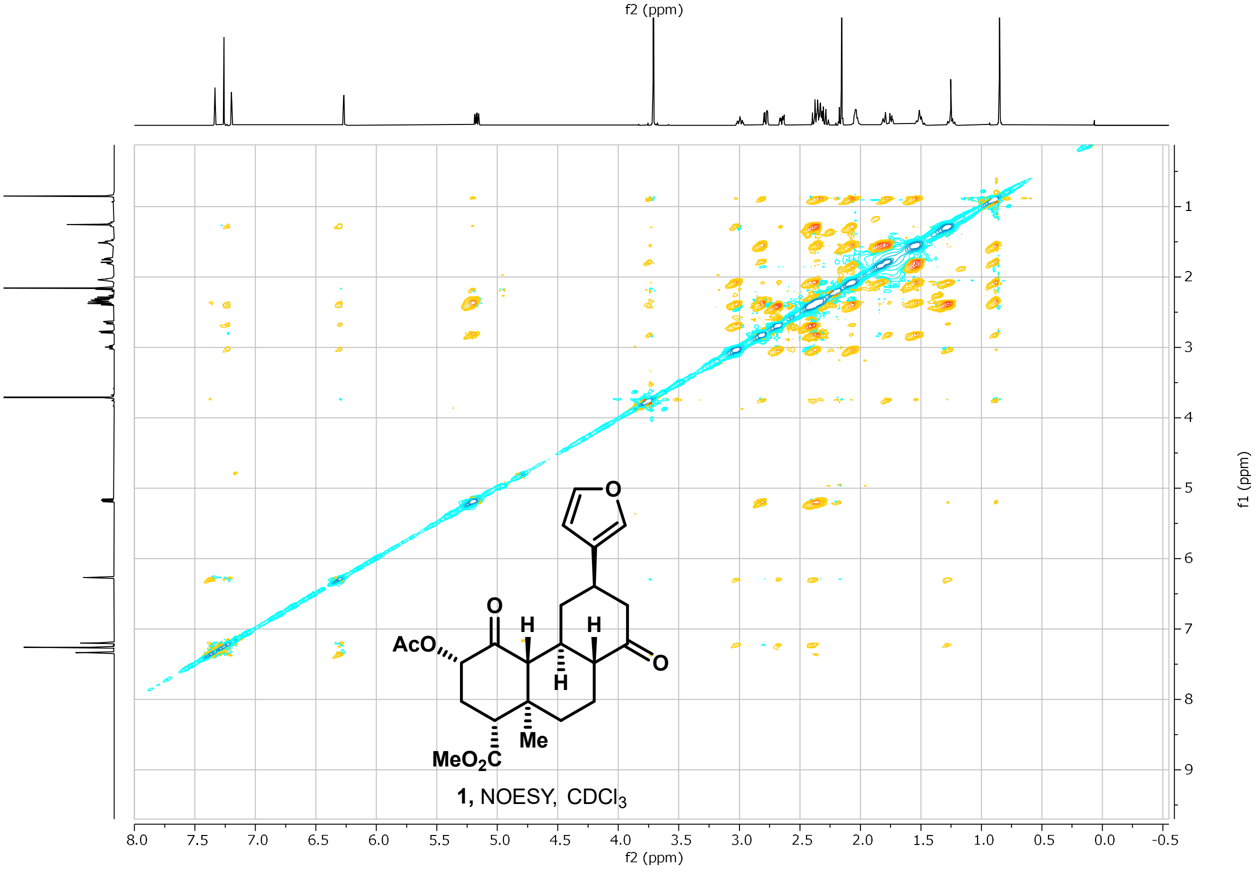

AcO
O
H
H
H
O
O
Me
MeO2C
1, NOESY, CDCl3
f2 (ppm)
f1 (ppm)

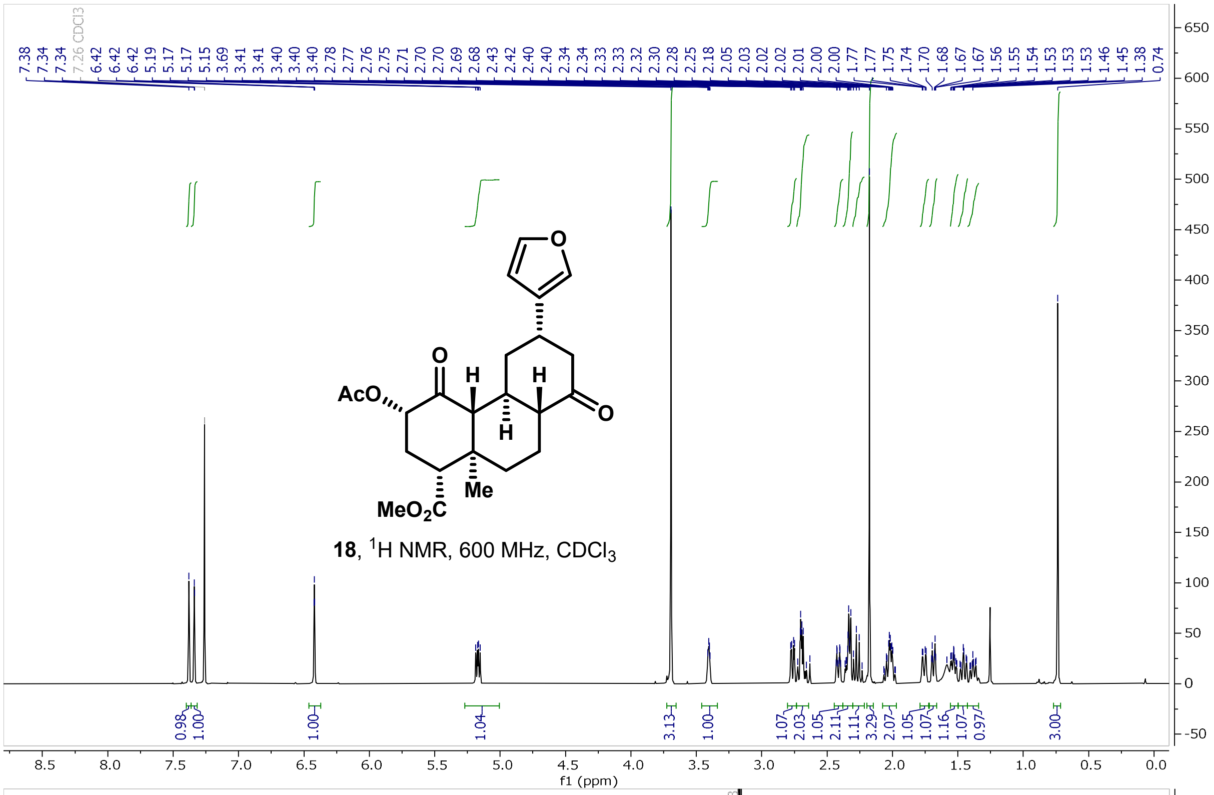

18, ^{1}H NMR, 600 MHz, $CDCl_3$

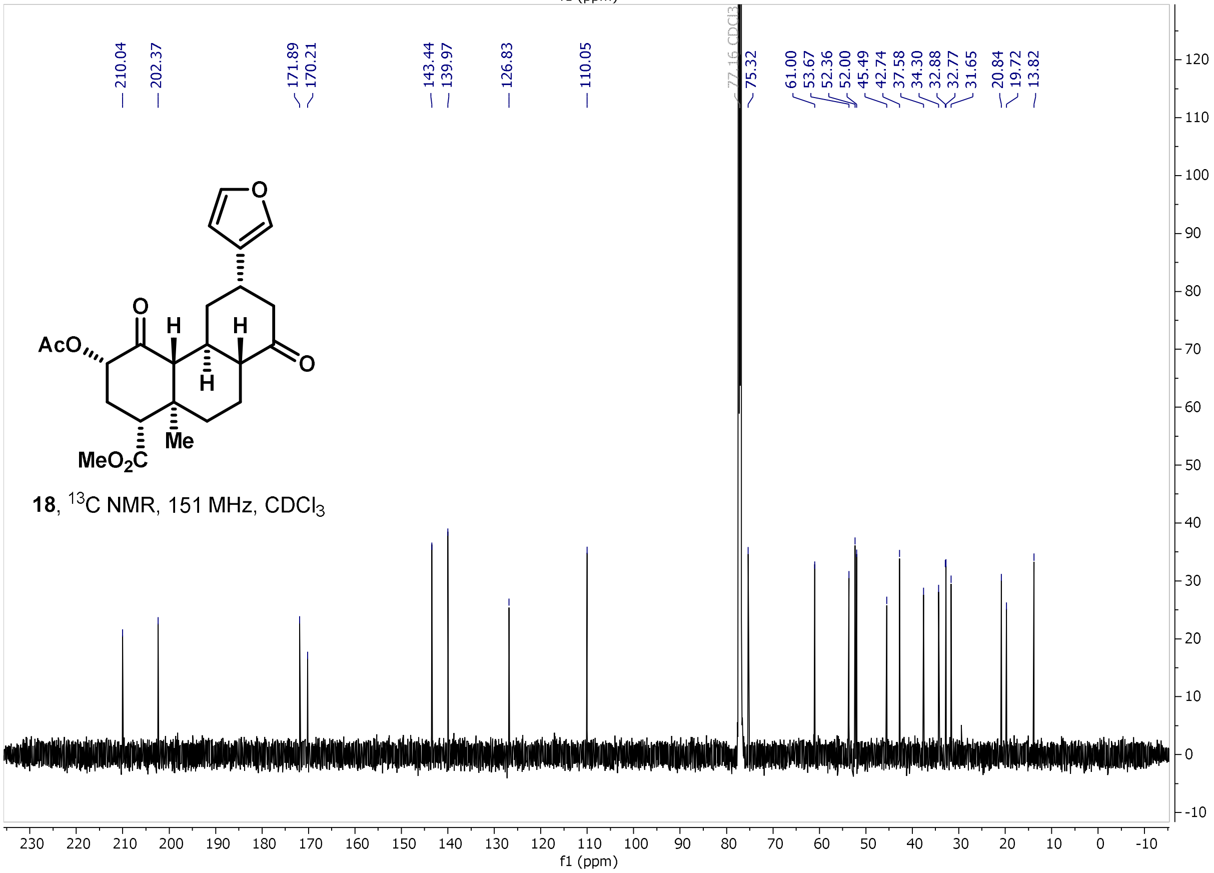

18, ^{13}C NMR, 151 MHz, $CDCl_3$

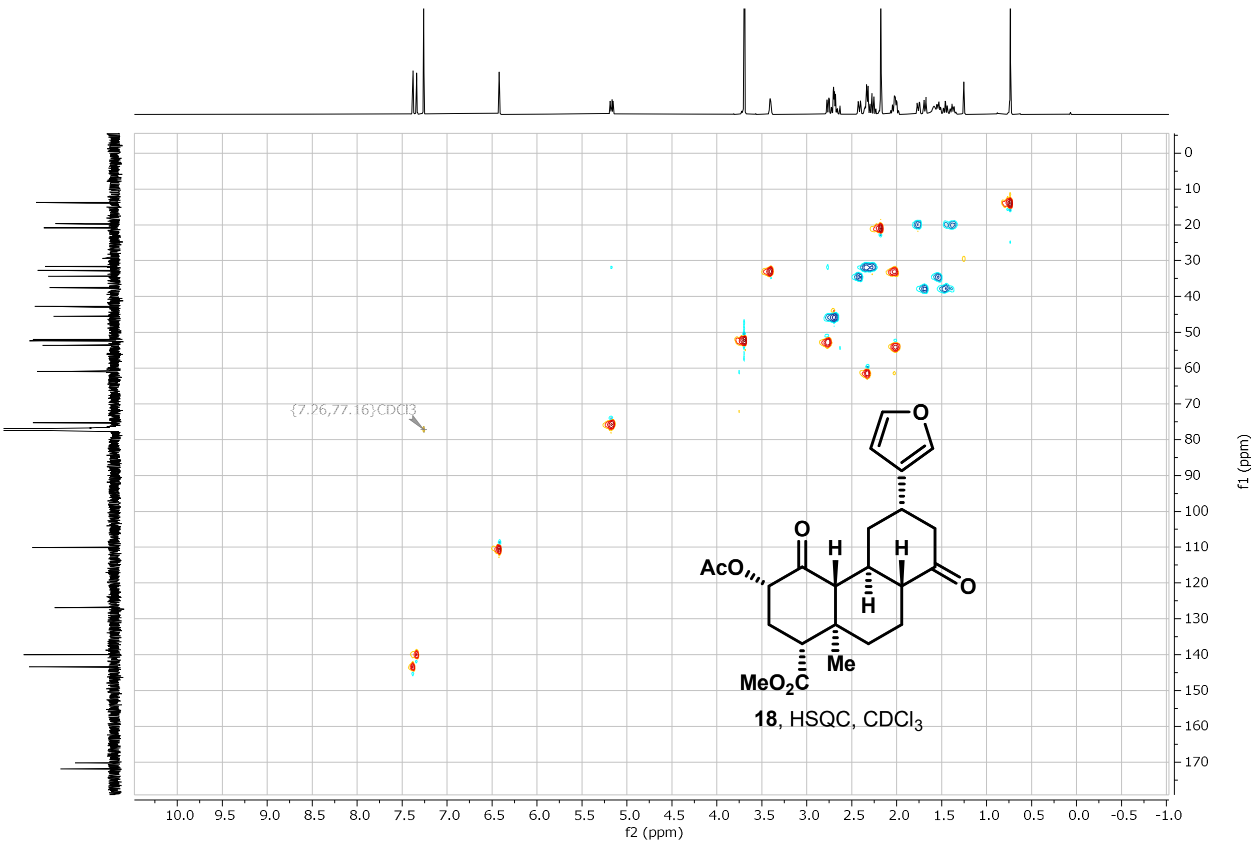
{7.26,77.16}CDCl3
AcO
O
H
H
H
O
O
Me
MeO2C
18, HSQC, CDCl3
f1 (ppm)
f2 (ppm)

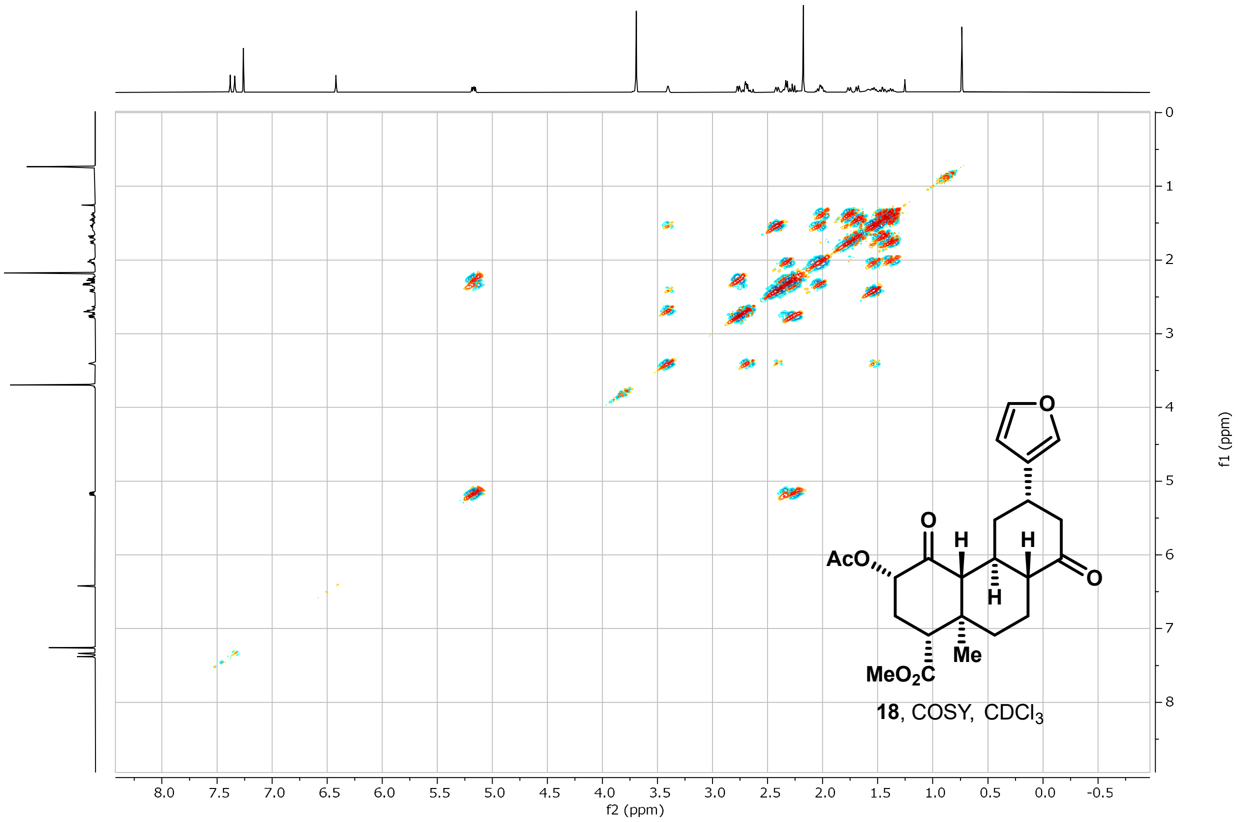
AcO
O
H
H
H
O
O
Me
MeO2C
18, COSY, CDCl3
f1 (ppm)
f2 (ppm)

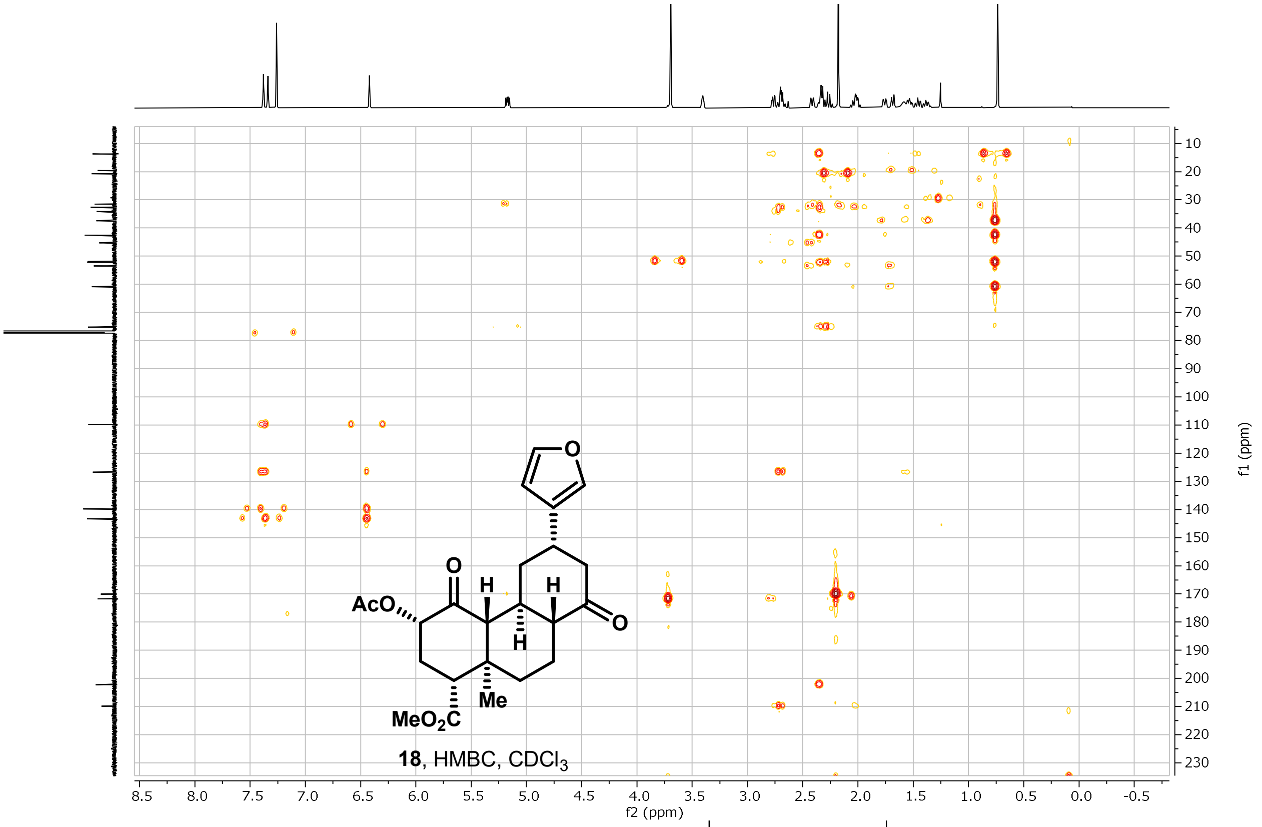

18, HMBC, $CDCl_3$

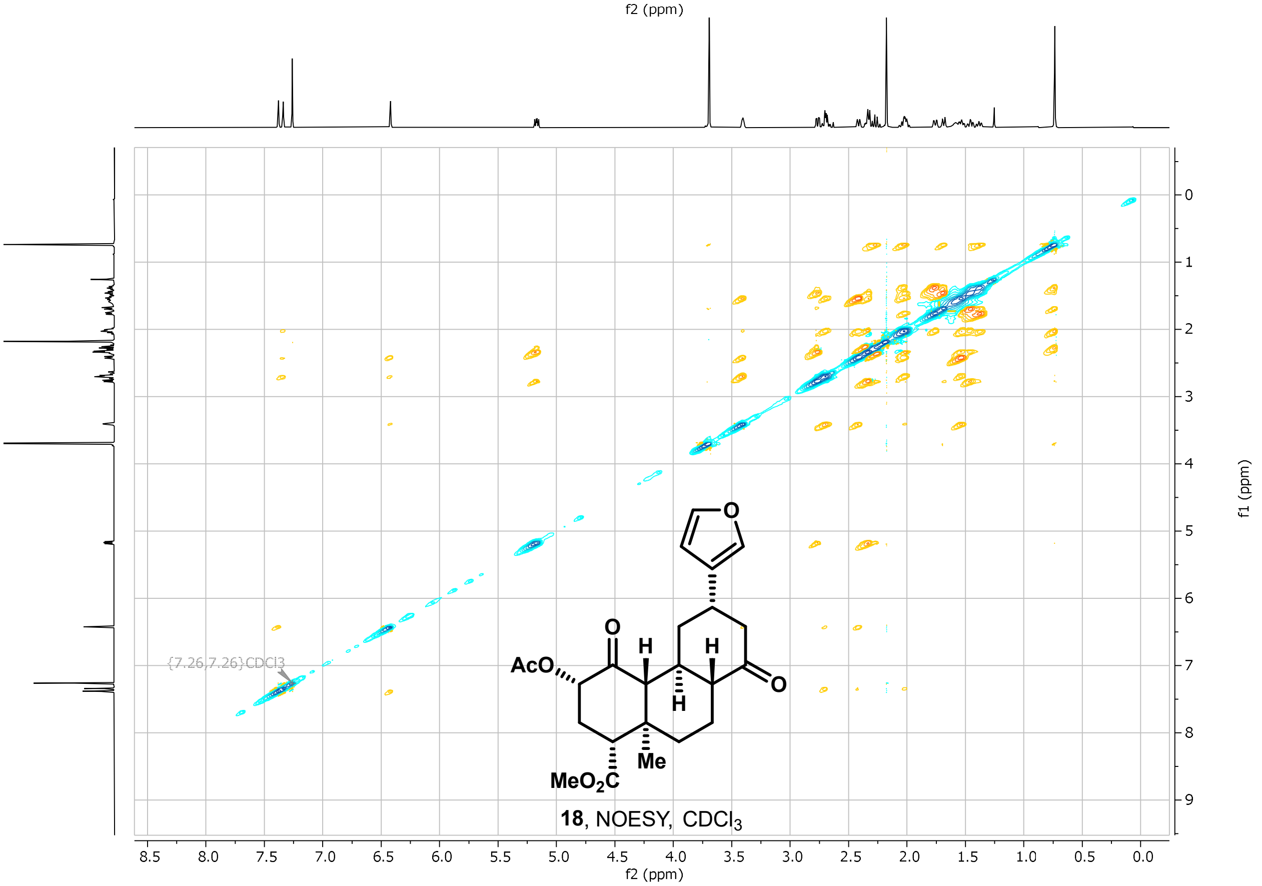

18, NOESY, $CDCl_3$

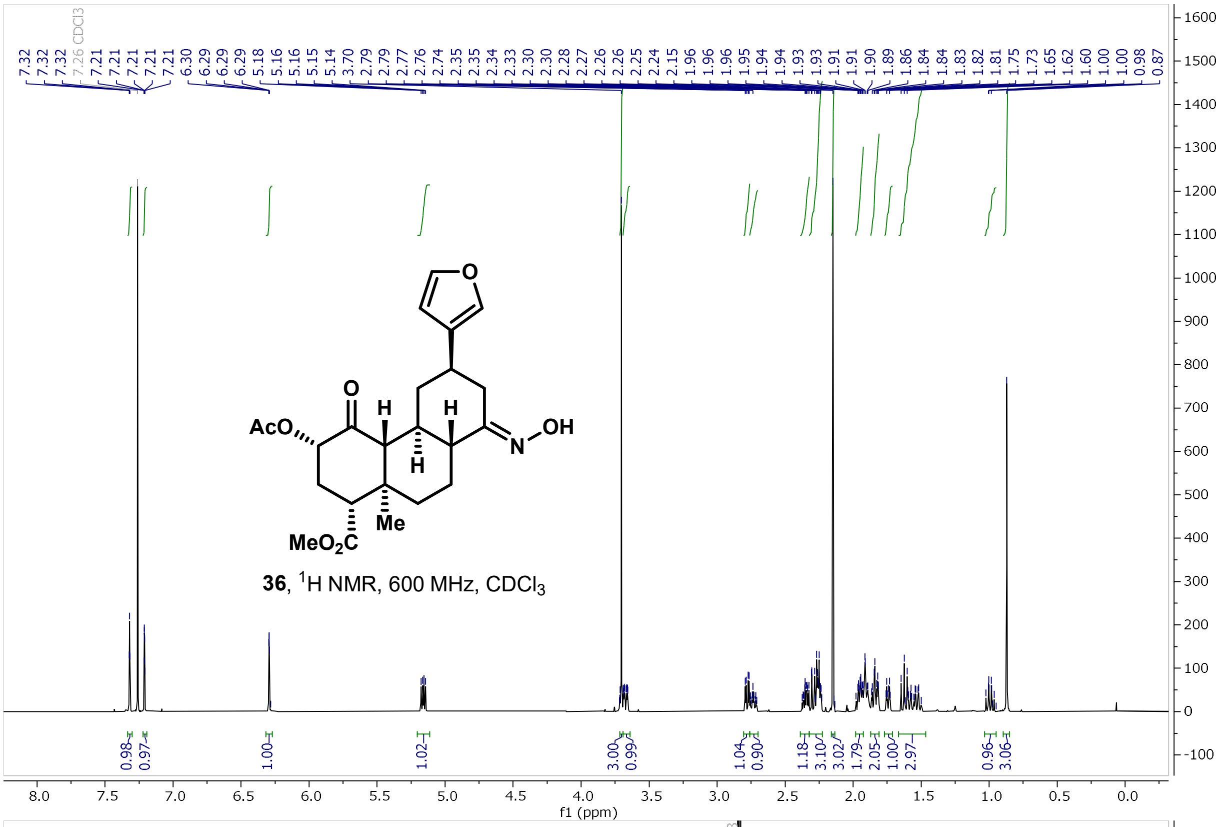
36, ¹H NMR, 600 MHz, CDCl3
f1 (ppm)

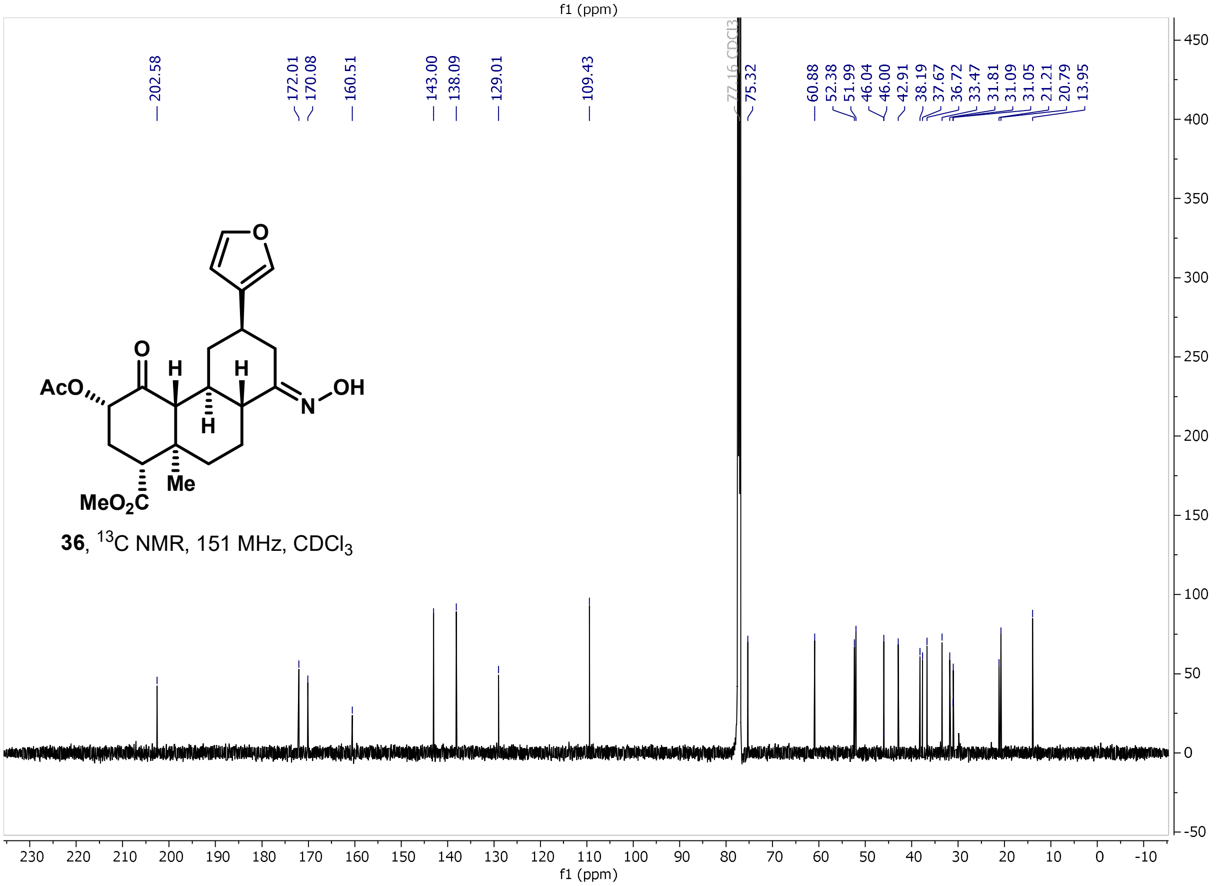
36, ¹³C NMR, 151 MHz, CDCl3
202.58
172.01
170.08
160.51
143.00
138.09
129.01
109.43
75.32
60.88
52.38
51.99
46.04
46.00
42.91
38.19
37.67
36.72
33.47
31.81
31.09
31.05
21.21
20.79
13.95
f1 (ppm)

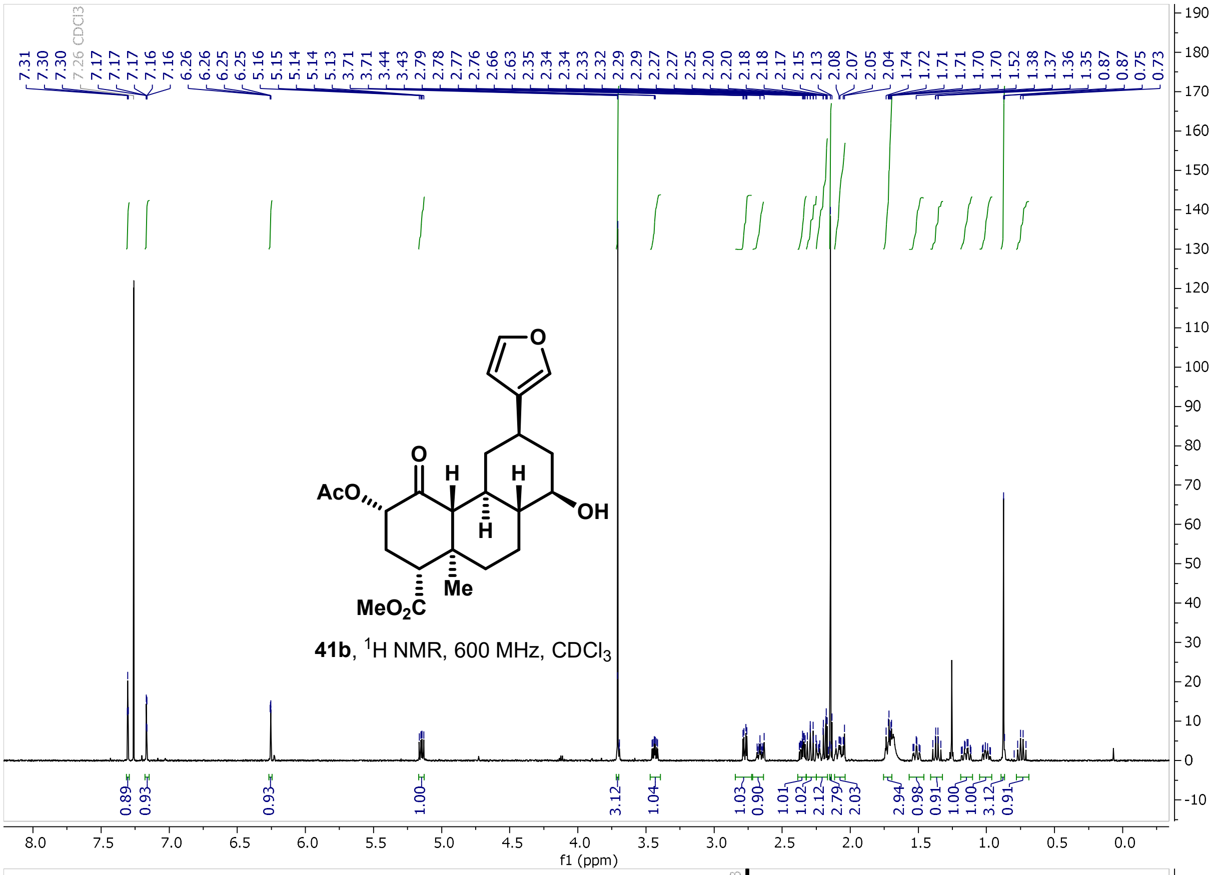
41b, 1H NMR, 600 MHz, CDCl3

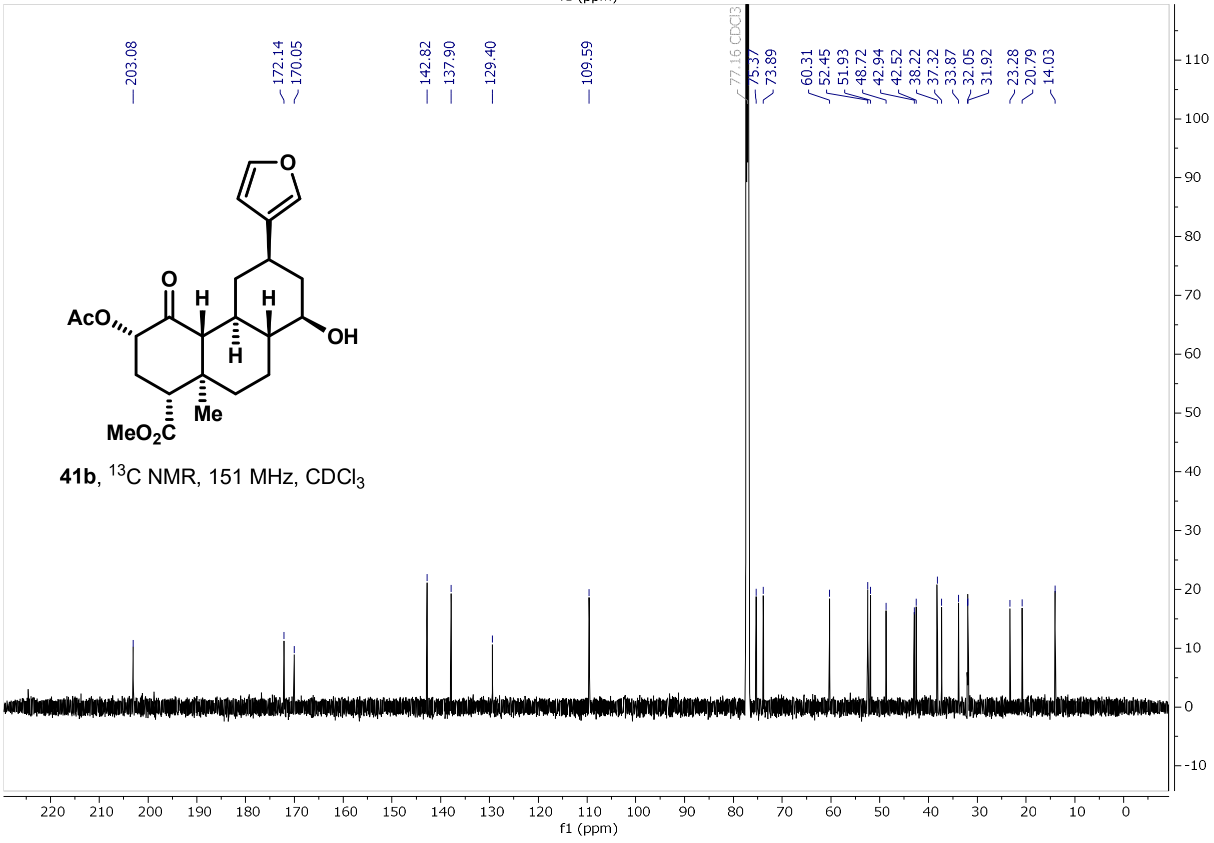
41b, 13C NMR, 151 MHz, CDCl3

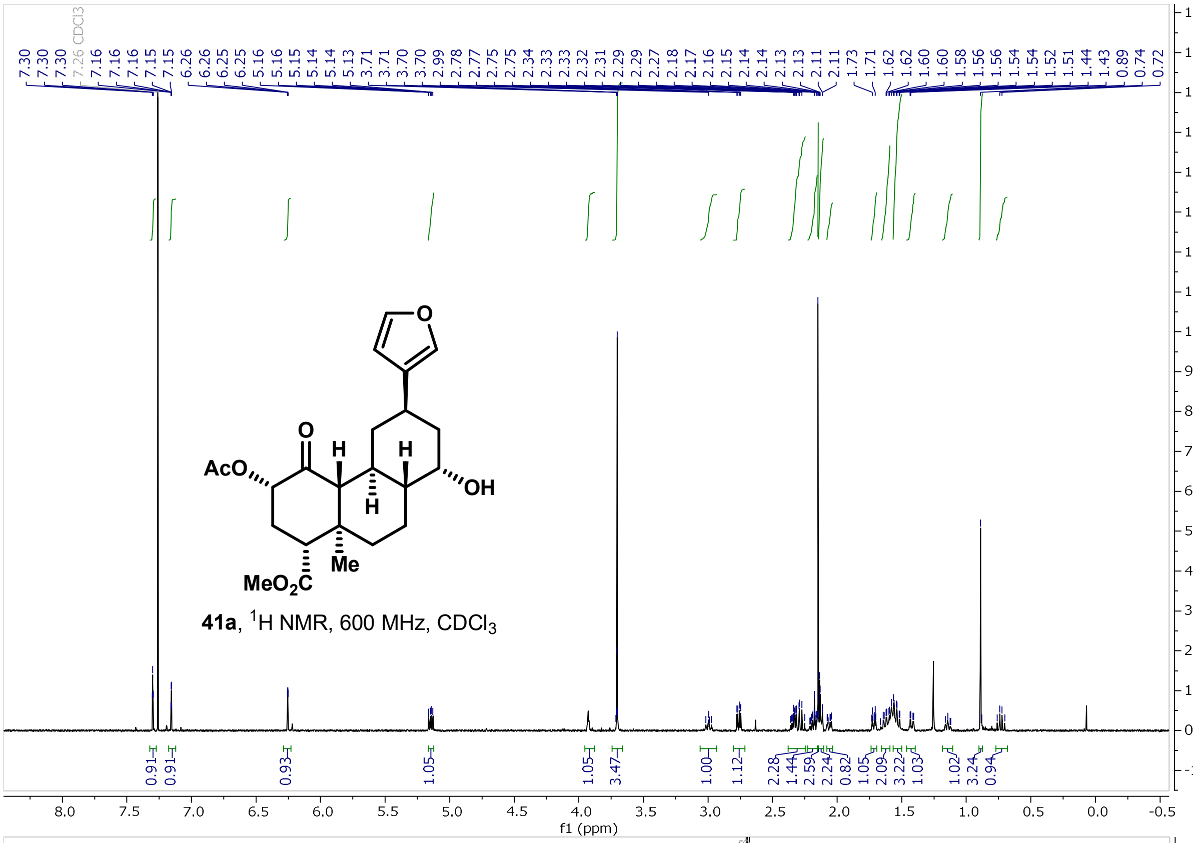

41a, ^{1}H NMR, 600 MHz, $CDCl_3$

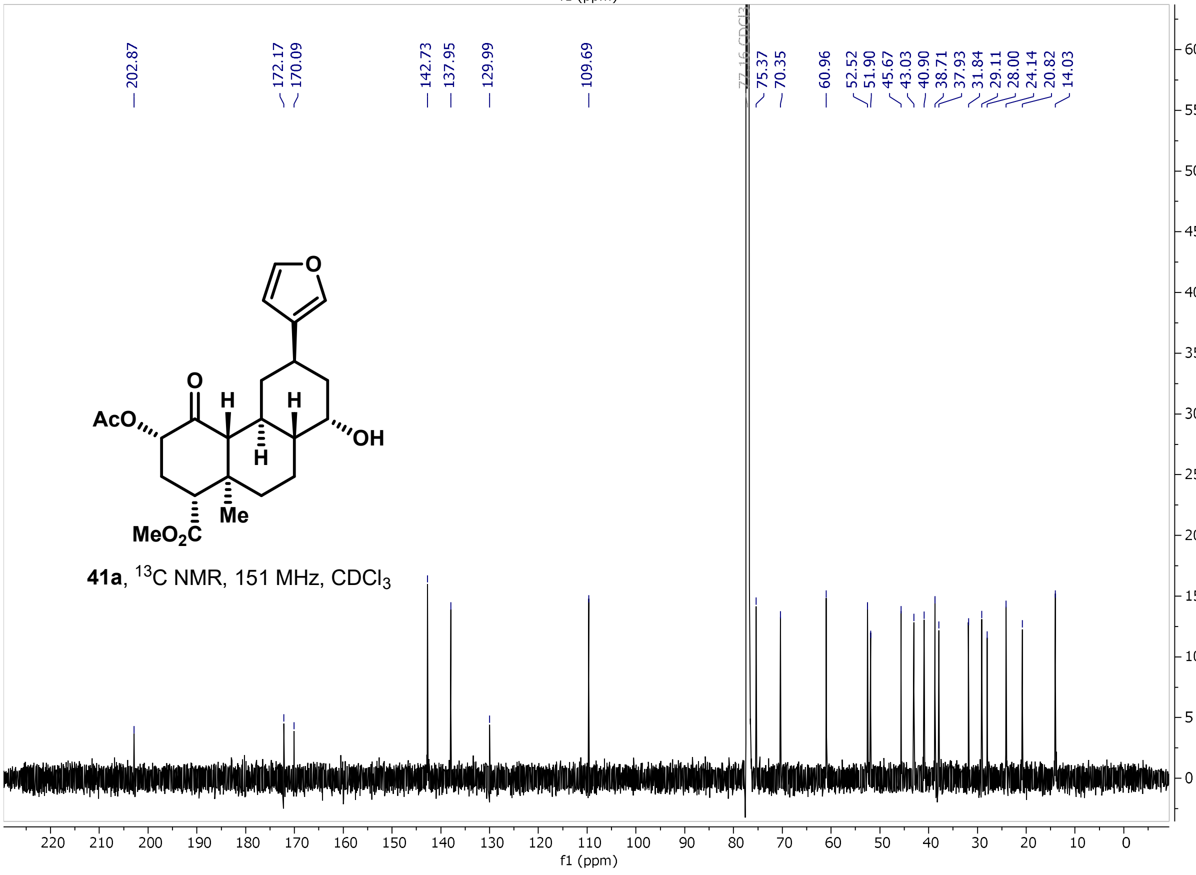

41a, ^{13}C NMR, 151 MHz, $CDCl_3$

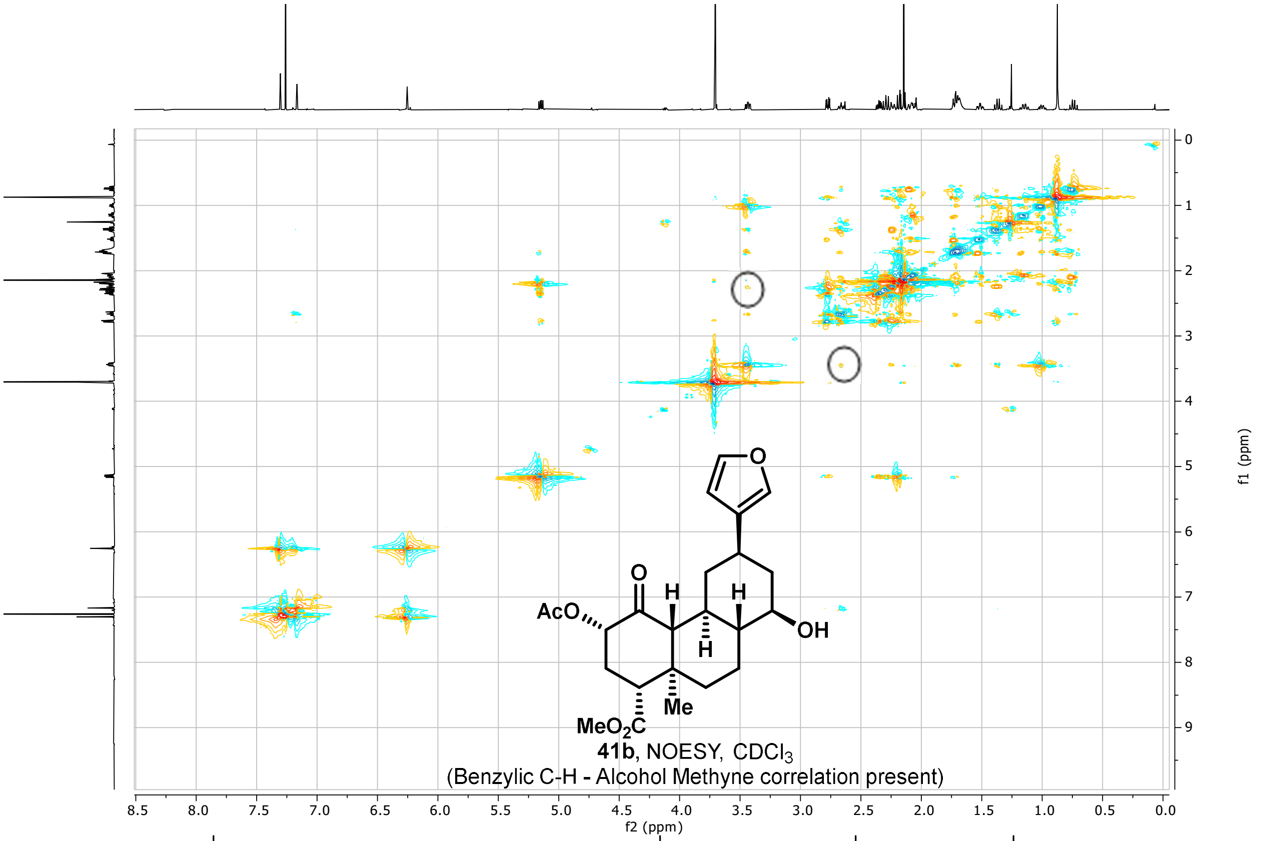

41b, NOESY, $CDCl_3$
(Benzylic C-H - Alcohol Methyne correlation present)

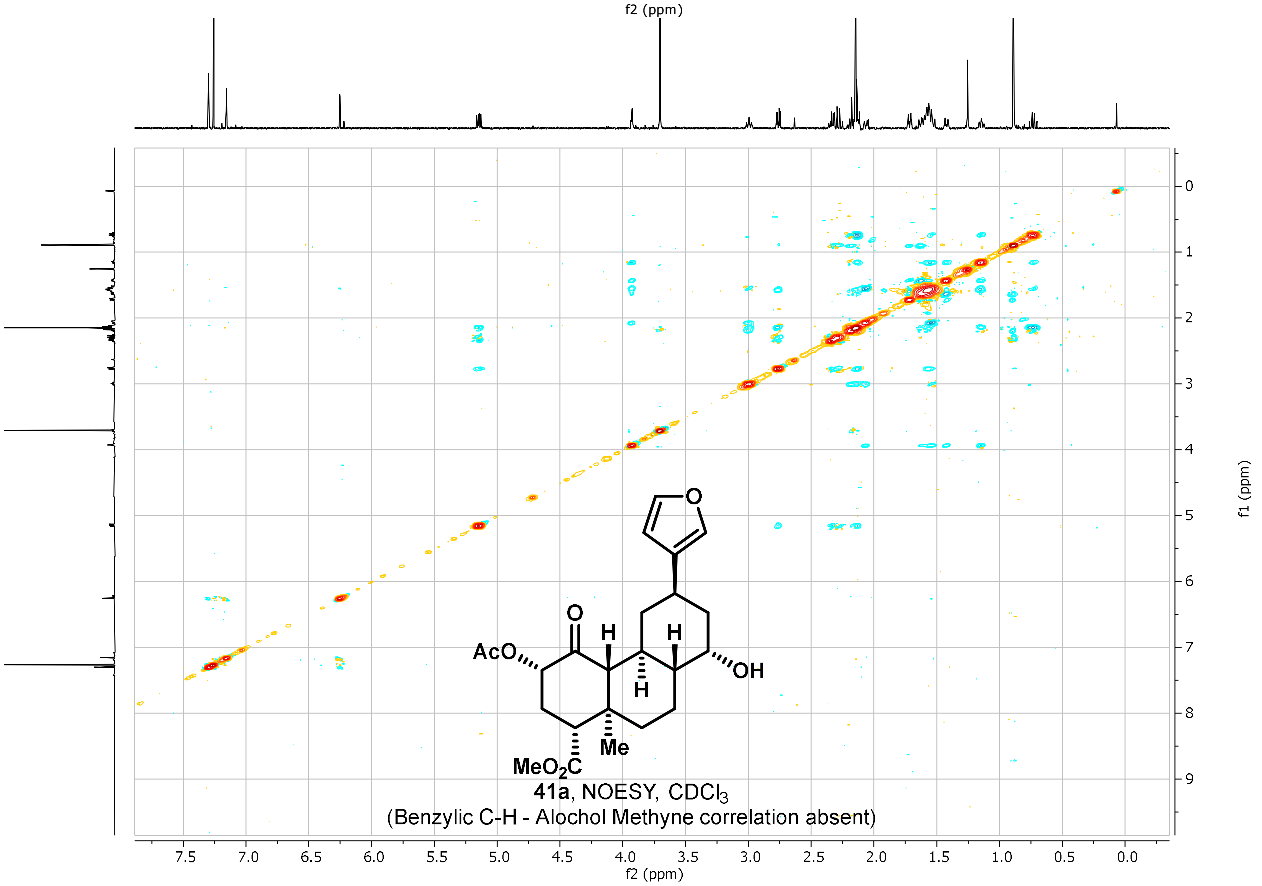

41a, NOESY, $CDCl_3$
(Benzylic C-H - Alochol Methyne correlation absent)

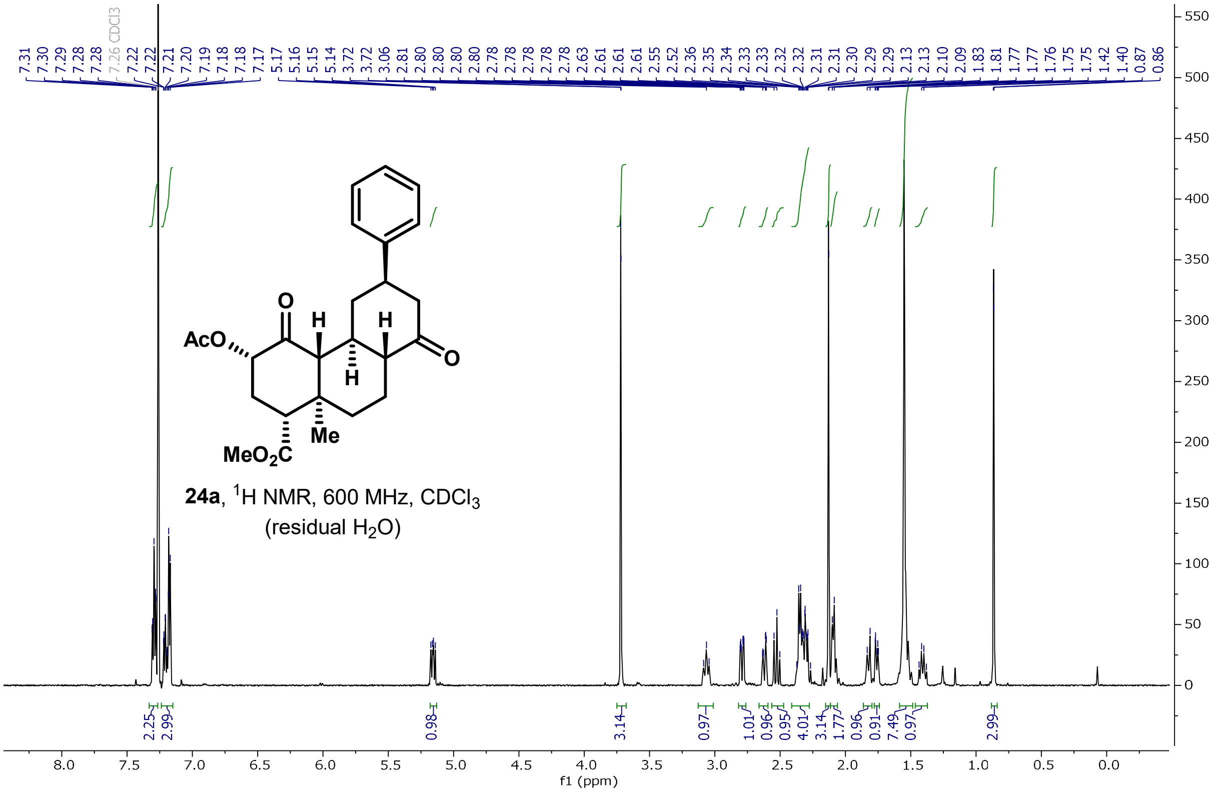

24a, ^{1}H NMR, 600 MHz, $CDCl_3$
(residual H_2O)

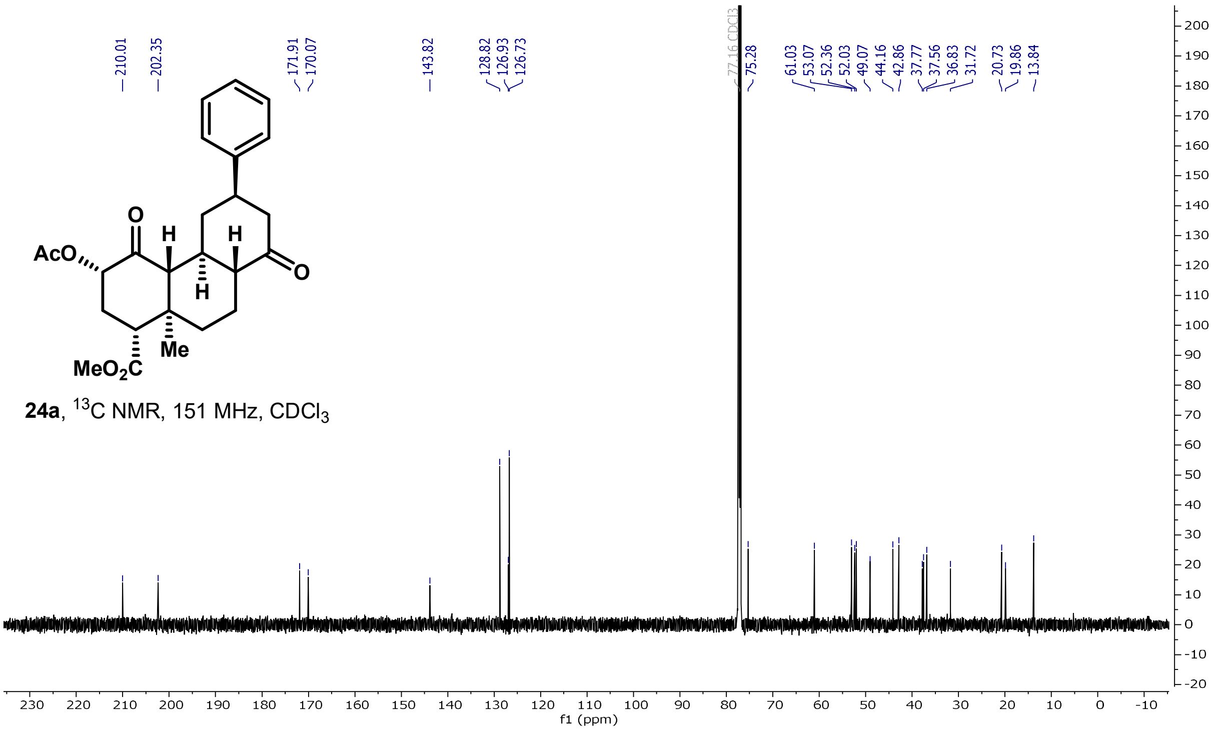

24a, ^{13}C NMR, 151 MHz, $CDCl_3$

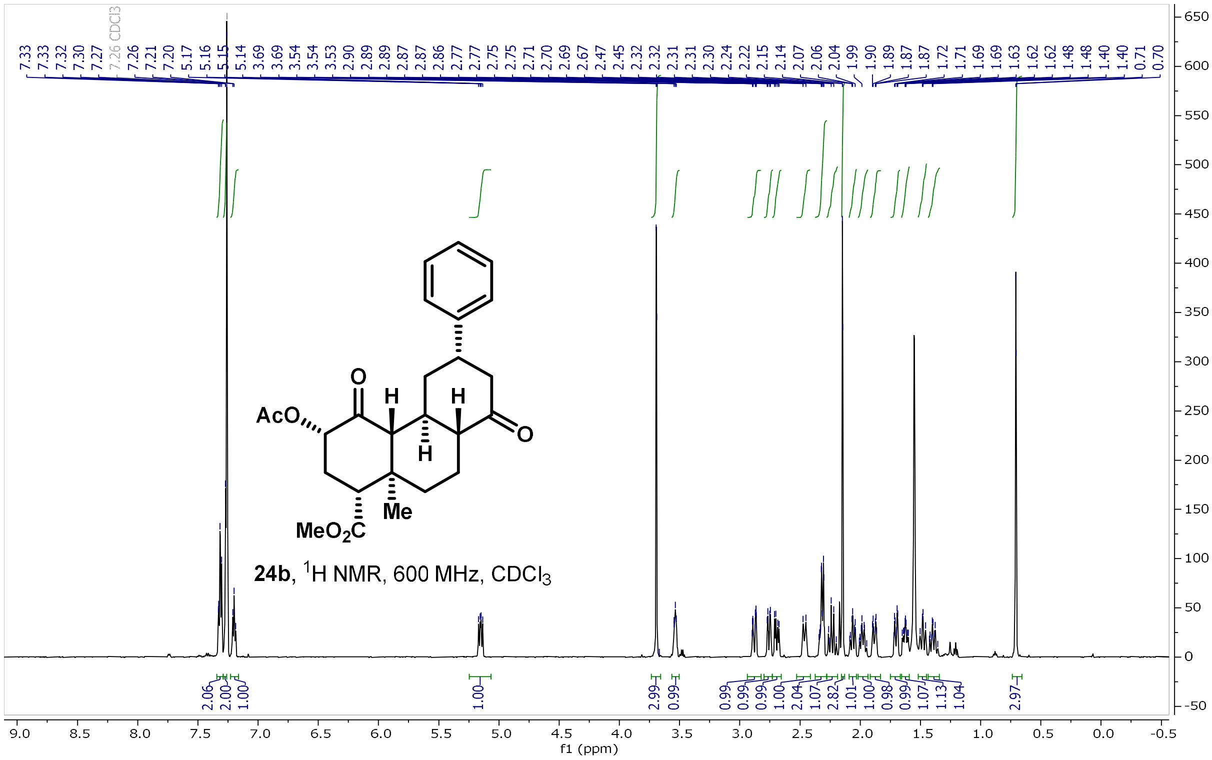
7.33 7.33 7.32 7.30 7.27 7.26 CDCl3 7.26 7.21 7.20 5.17 5.16 5.15 5.14 3.69 3.69 3.54 3.54 3.53 2.90 2.89 2.89 2.87 2.87 2.86 2.77 2.77 2.75 2.75 2.71 2.70 2.69 2.67 2.47 2.45 2.32 2.32 2.31 2.31 2.30 2.24 2.22 2.15 2.14 2.07 2.06 2.04 1.99 1.90 1.89 1.87 1.87 1.72 1.71 1.69 1.69 1.63 1.62 1.48 1.48 1.40 1.40 0.71 0.70
2.06 2.00 1.00 1.00 2.99 0.99 0.99 0.99 0.99 1.00 2.04 1.07 2.82 1.01 1.00 0.98 0.99 1.07 1.13 1.04 2.97
AcO
O
H
H
H
O
Me
MeO2C
f1 (ppm)
24b, 1H NMR, 600 MHz, CDCl3

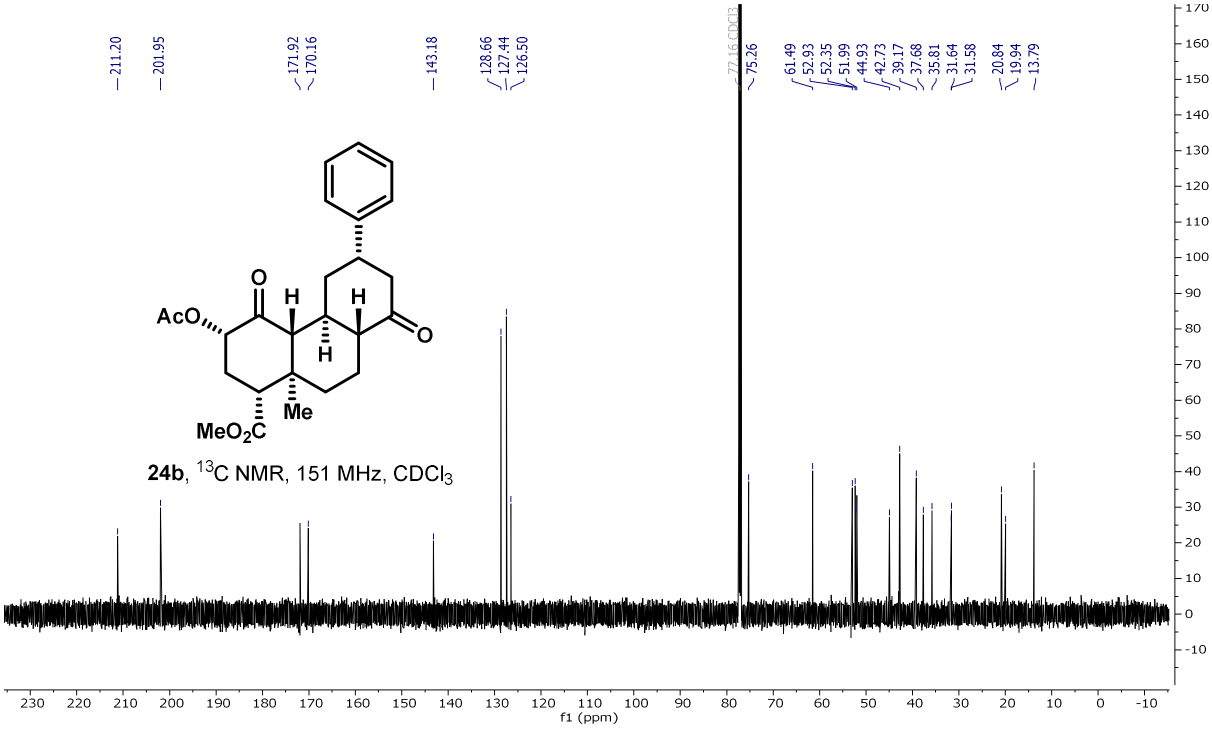
211.20 201.95 171.92 170.16 143.18 128.66 127.44 126.50 77.16 CDCl3 75.26 61.49 52.93 52.35 51.99 44.93 42.73 39.17 37.68 35.81 31.64 31.58 20.84 19.94 13.79
AcO
O
H
H
H
O
Me
MeO2C
f1 (ppm)
24b, 13C NMR, 151 MHz, CDCl3

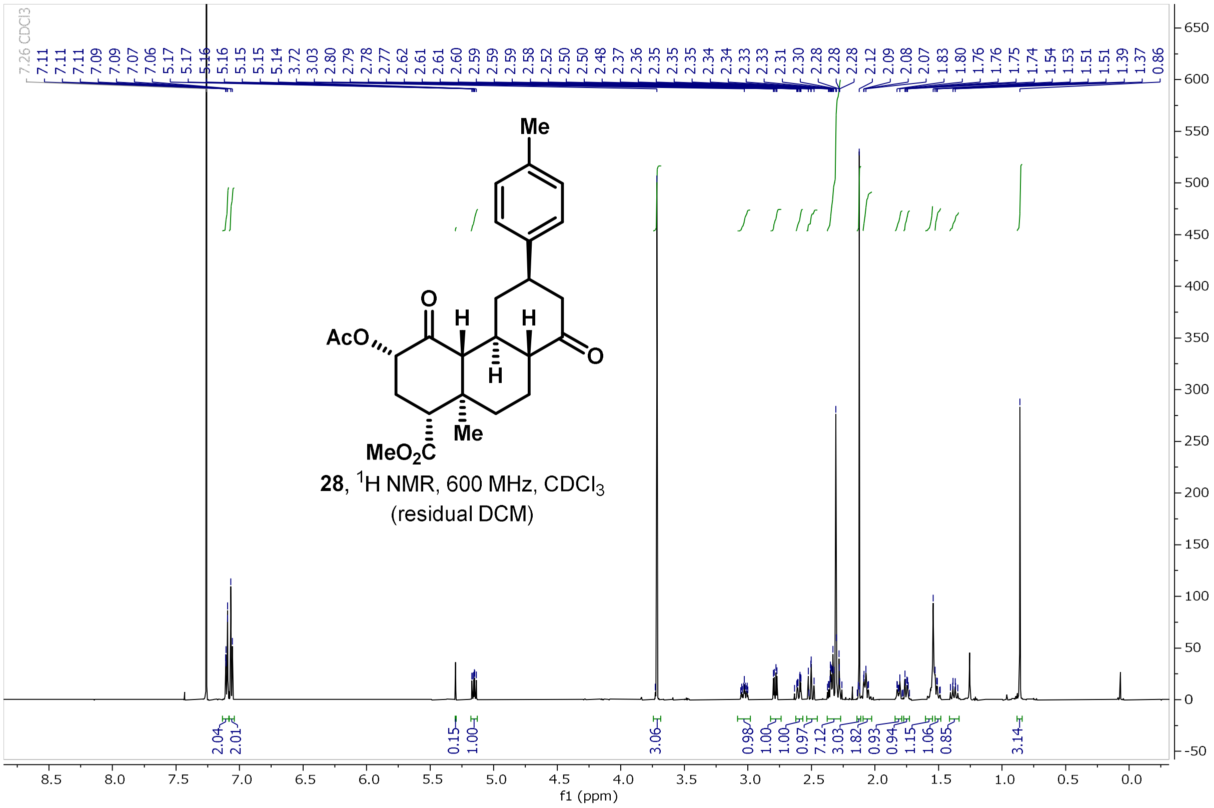
28, 1H NMR, 600 MHz, CDCl3
(residual DCM)
AcO
MeO2C
Me
H
O
f1 (ppm)

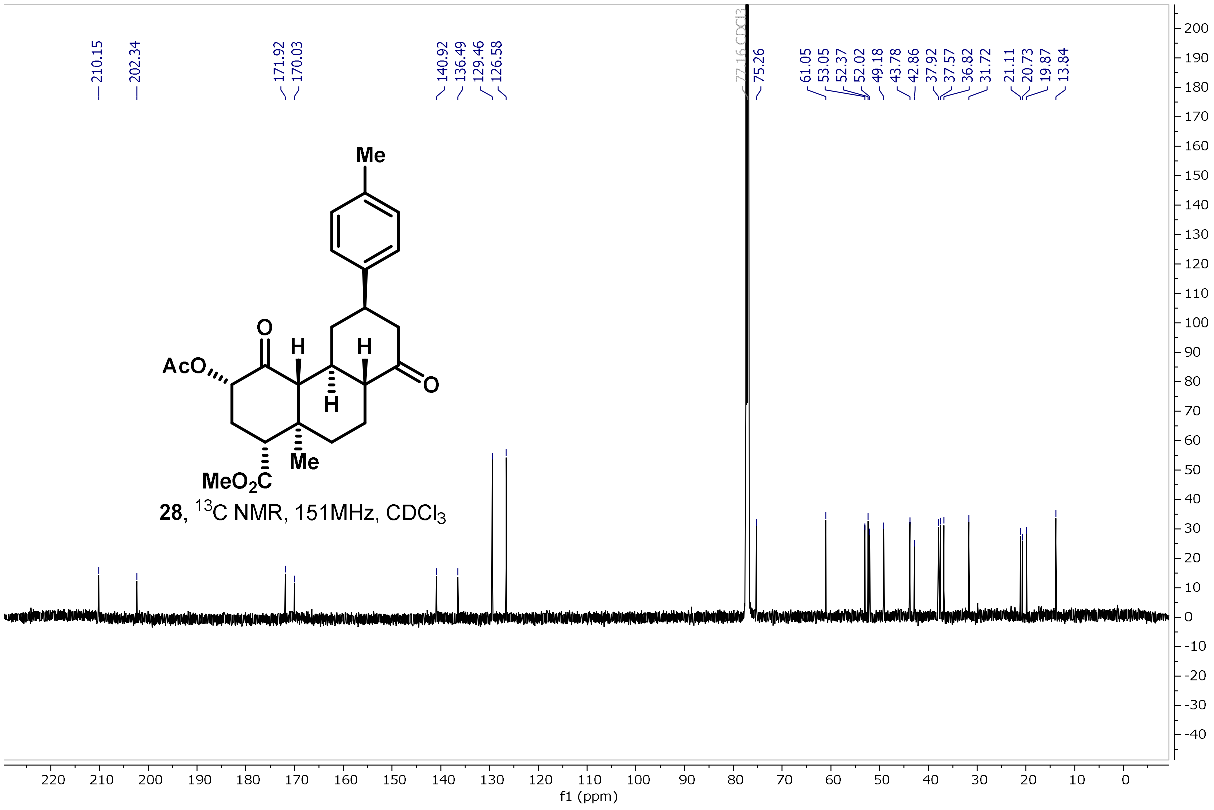
28, 13C NMR, 151MHz, CDCl3
AcO
MeO2C
Me
H
O
f1 (ppm)

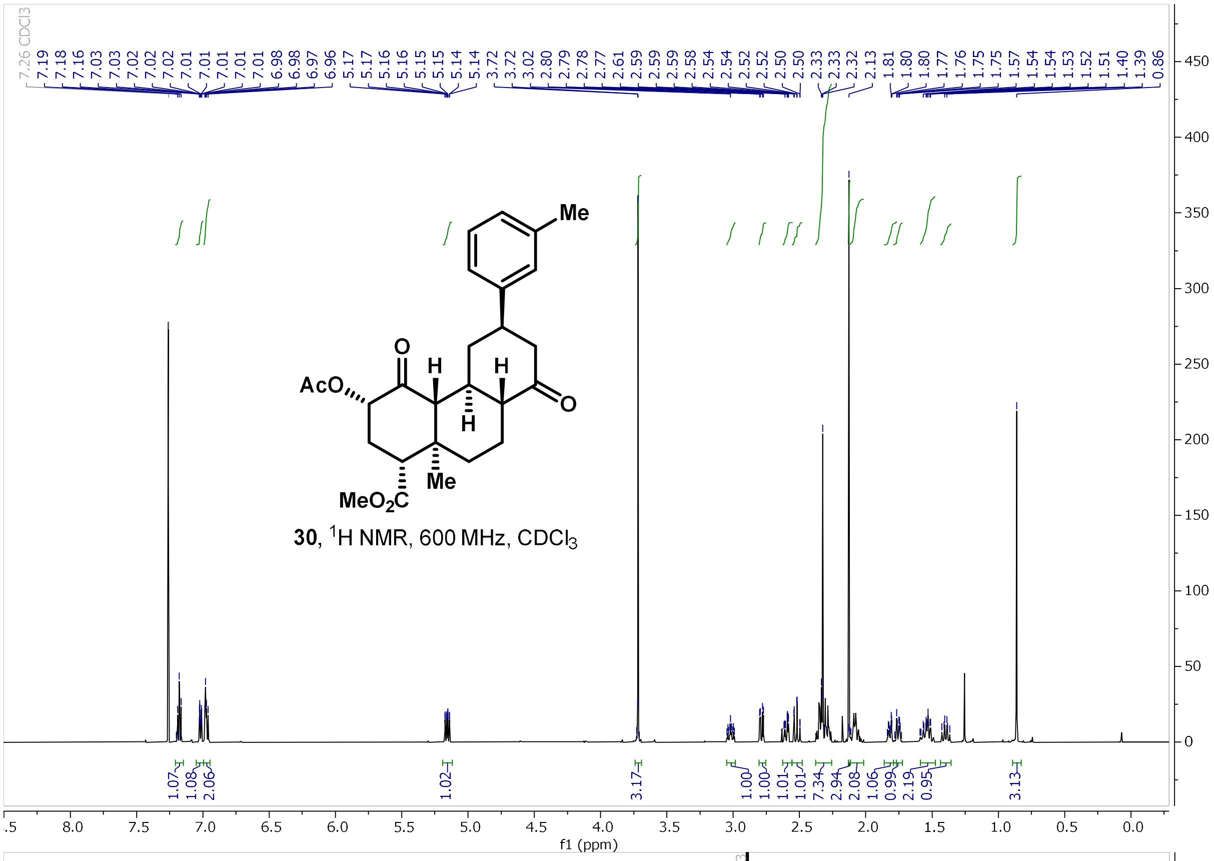

30, ^{1}H NMR, 600 MHz, $CDCl_3$

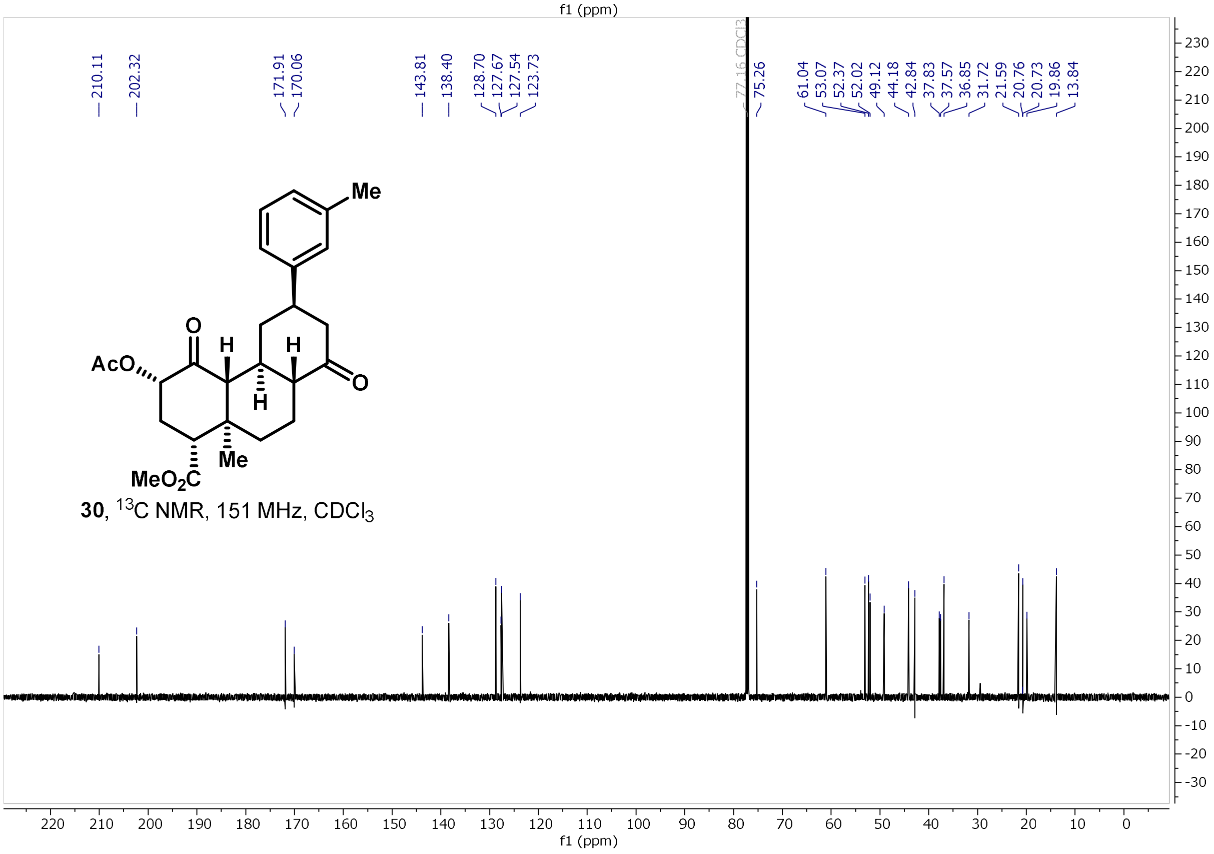

30, ^{13}C NMR, 151 MHz, $CDCl_3$

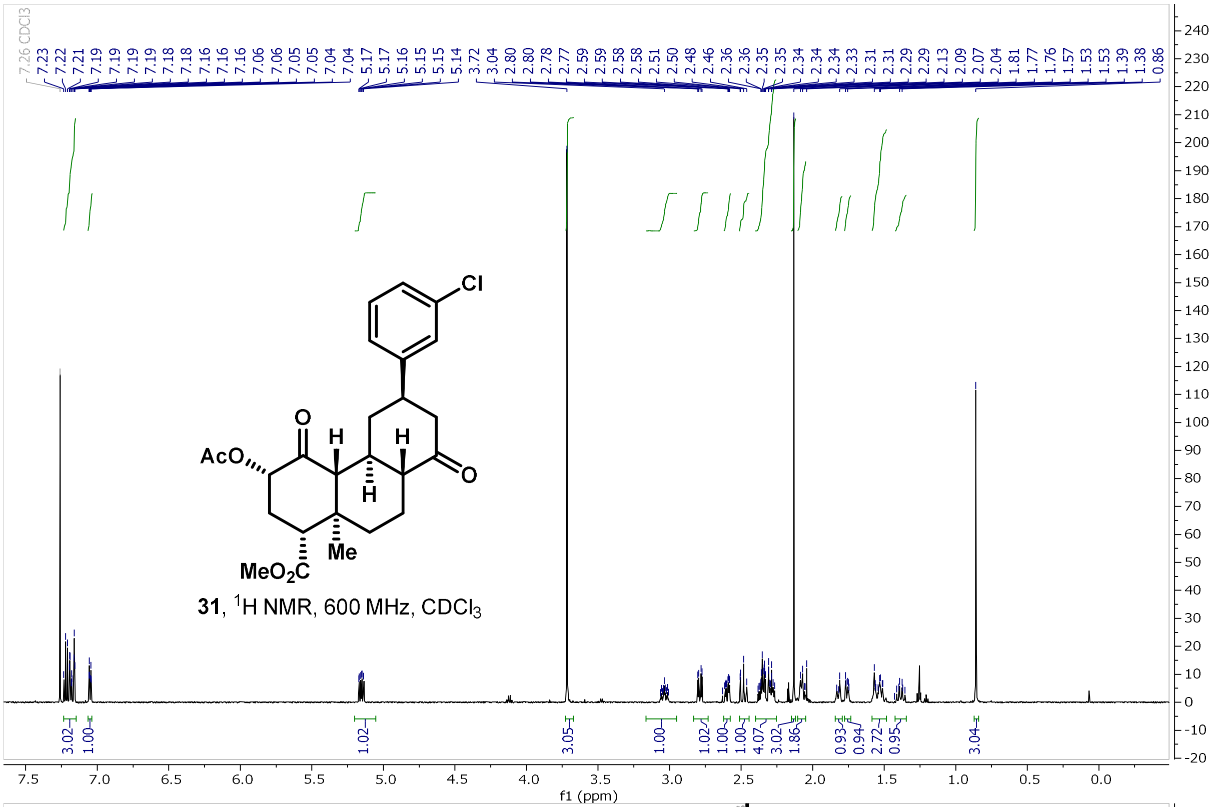
AcO
MeO2C
Me
Cl
31, 1H NMR, 600 MHz, CDCl3
f1 (ppm)

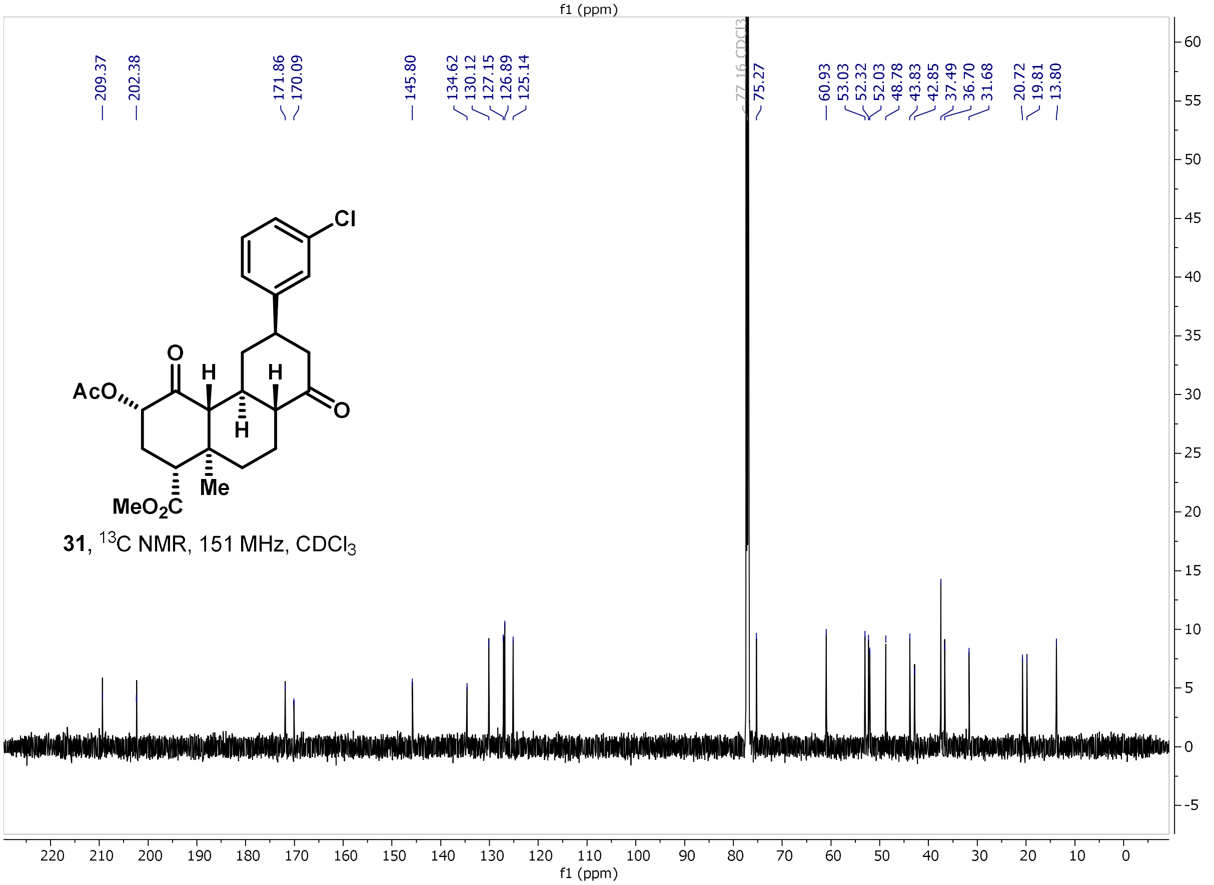
AcO
MeO2C
Me
Cl
31, 13C NMR, 151 MHz, CDCl3
209.37
202.38
171.86
170.09
145.80
134.62
130.12
127.15
126.89
125.14
75.27
60.93
53.03
52.32
52.03
48.78
43.83
42.85
37.49
36.70
31.68
20.72
19.81
13.80
f1 (ppm)

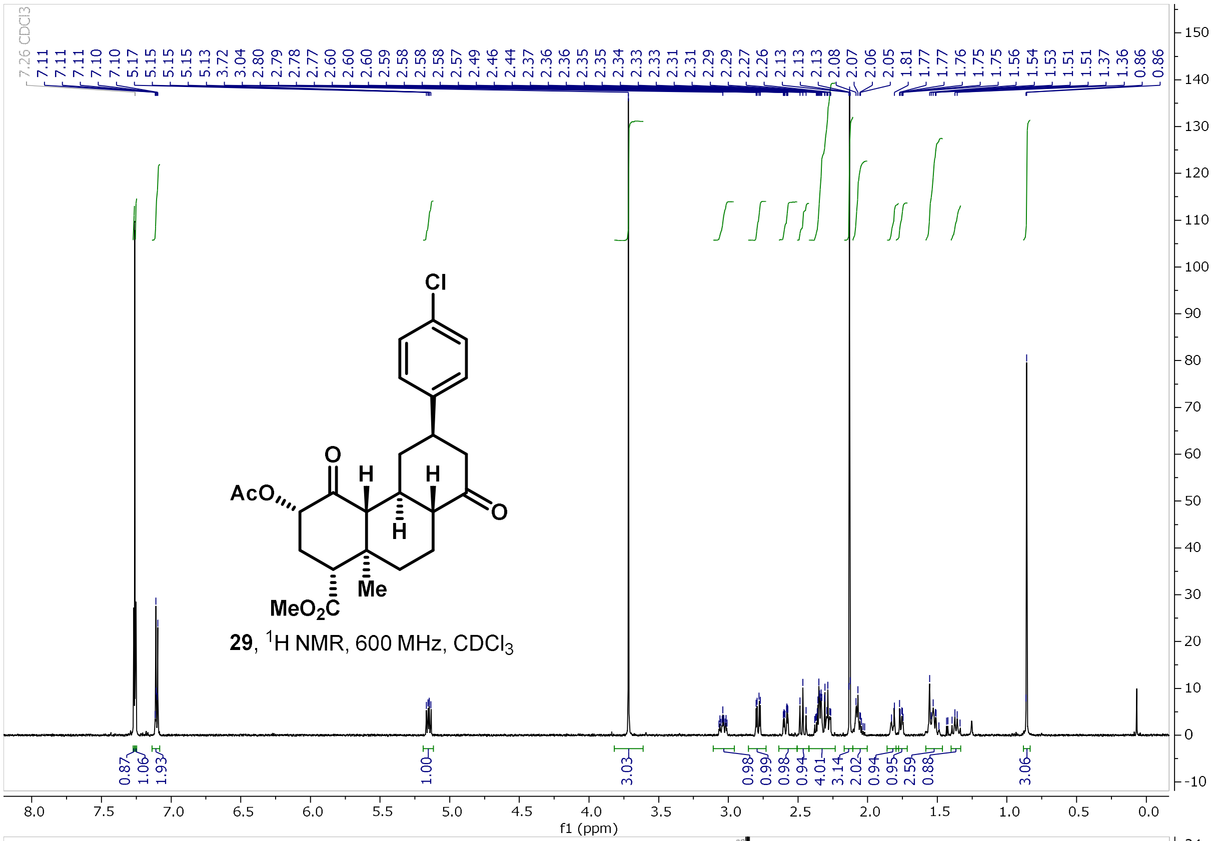

7.26 CDCl3
7.11
7.11
7.11
7.10
7.10
5.17
5.15
5.15
5.15
5.13
3.72
3.04
2.80
2.79
2.78
2.77
2.60
2.60
2.60
2.59
2.58
2.58
2.58
2.57
2.49
2.46
2.44
2.37
2.36
2.36
2.35
2.35
2.34
2.33
2.33
2.33
2.31
2.31
2.29
2.29
2.27
2.26
2.13
2.13
2.13
2.08
2.07
2.06
2.05
1.81
1.77
1.77
1.76
1.75
1.75
1.56
1.54
1.53
1.51
1.51
1.37
1.36
0.86
0.86
Cl
AcO
O
H
H
H
O
Me
MeO2C
29, 1H NMR, 600 MHz, CDCl3
0.87
1.06
1.93
1.00
3.03
0.98
0.99
0.98
0.94
4.01
3.14
2.02
0.94
0.95
2.59
0.88
3.06
f1 (ppm)

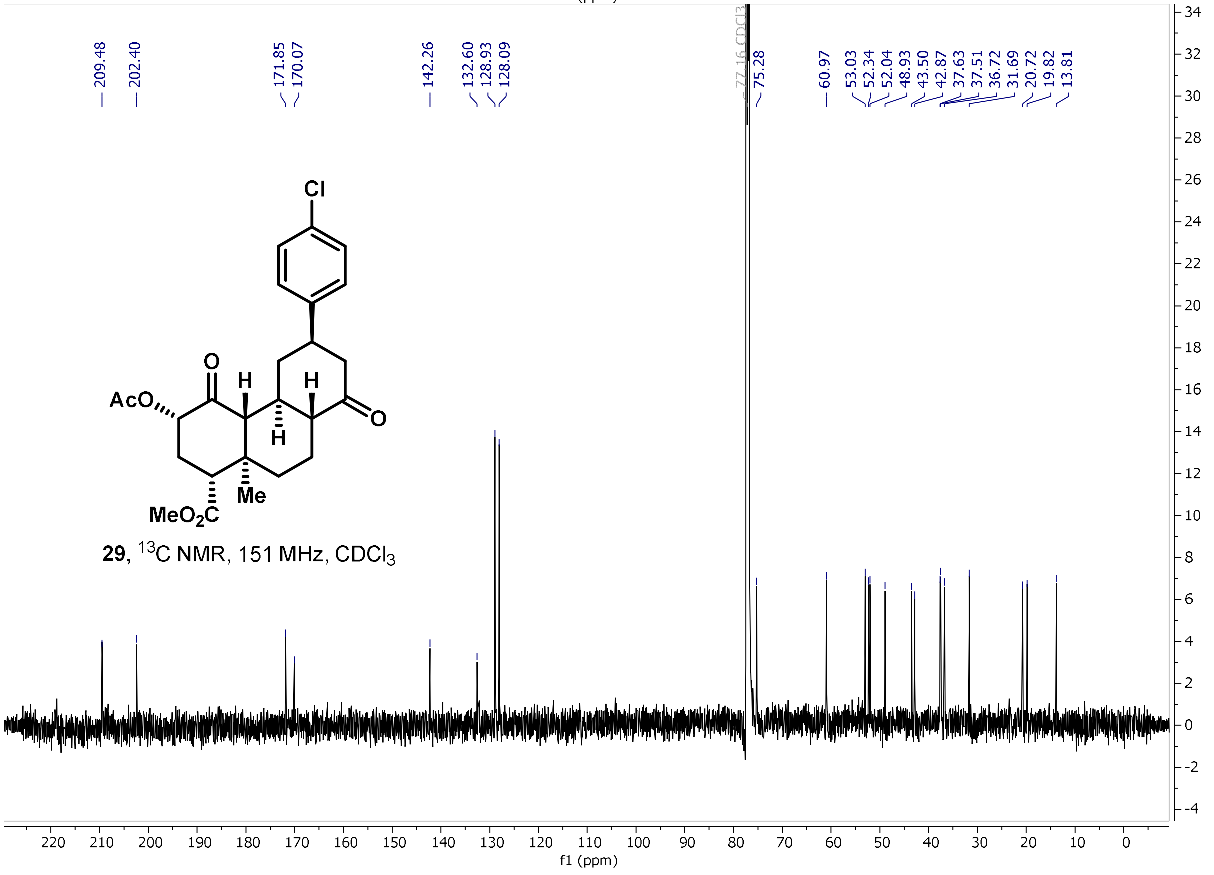

209.48
202.40
171.85
170.07
142.26
132.60
128.93
128.09
77.16 CDCl3
75.28
60.97
53.03
52.34
52.04
48.93
43.50
42.87
37.63
37.51
36.72
31.69
20.72
19.82
13.81
Cl
AcO
O
H
H
H
O
Me
MeO2C
29, 13C NMR, 151 MHz, CDCl3
f1 (ppm)

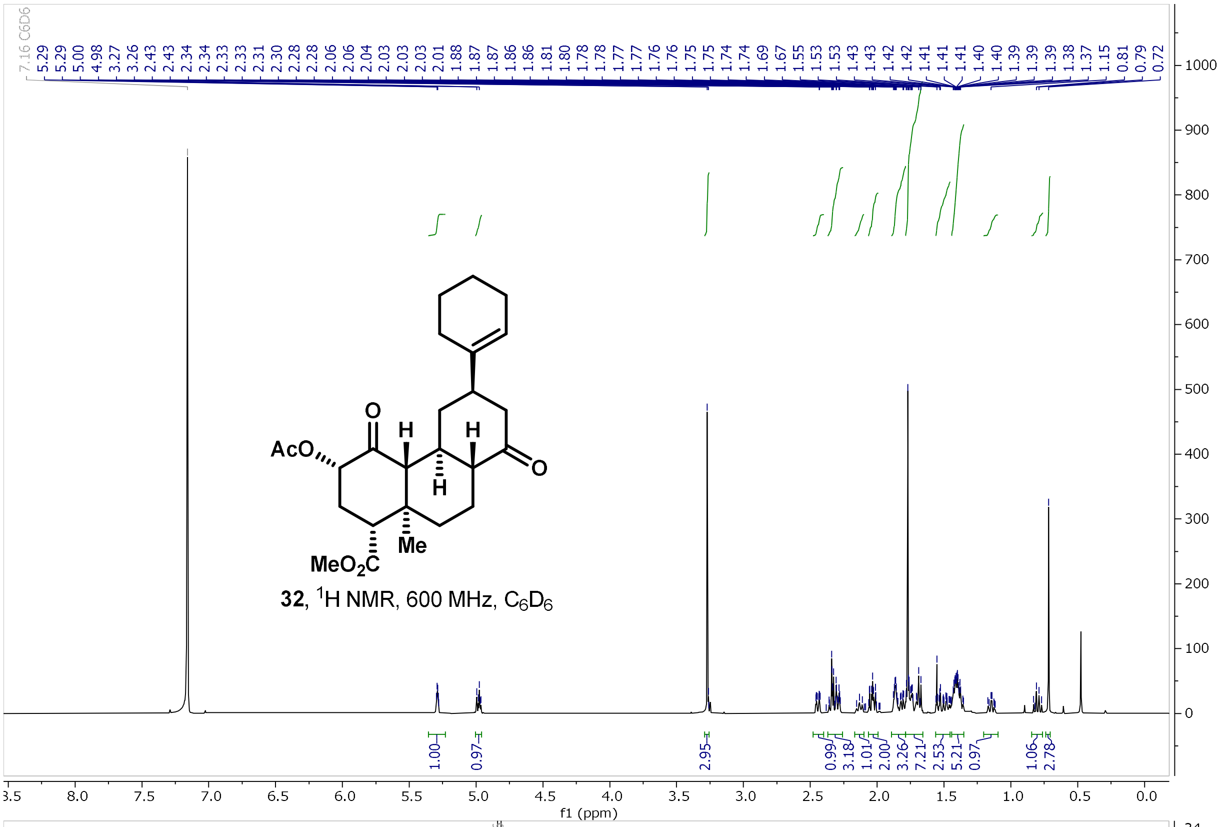

32, ^{1}H NMR, 600 MHz, C_6D_6

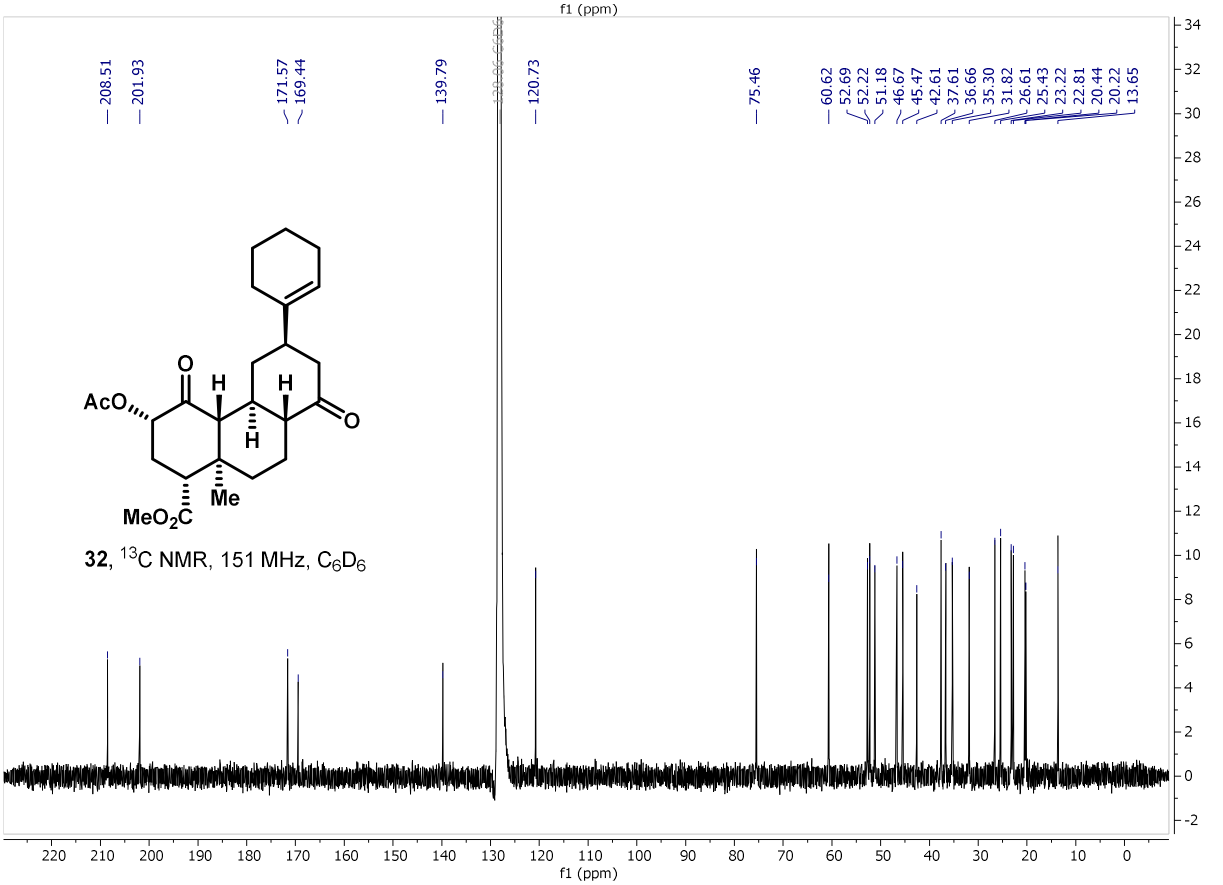

32, ^{13}C NMR, 151 MHz, C_6D_6

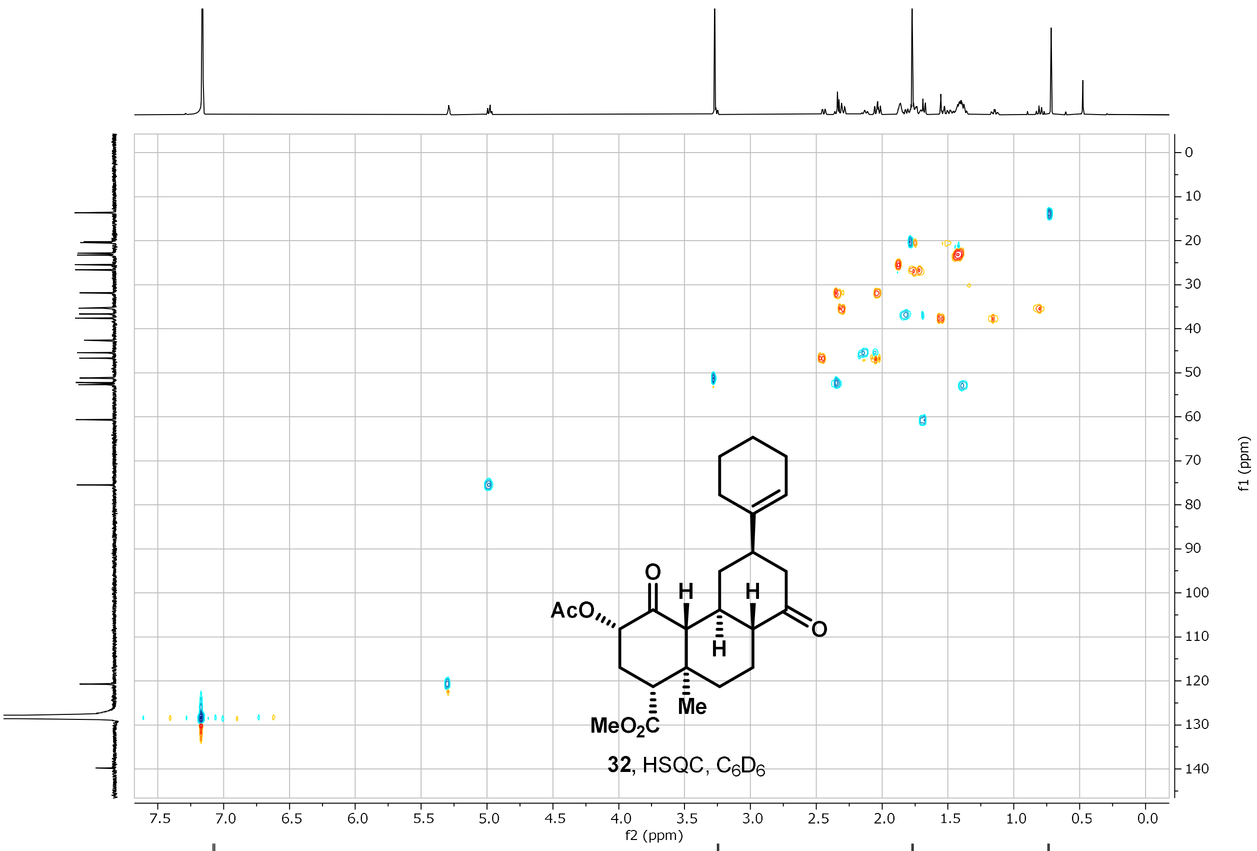

32, HSQC, C_6D_6

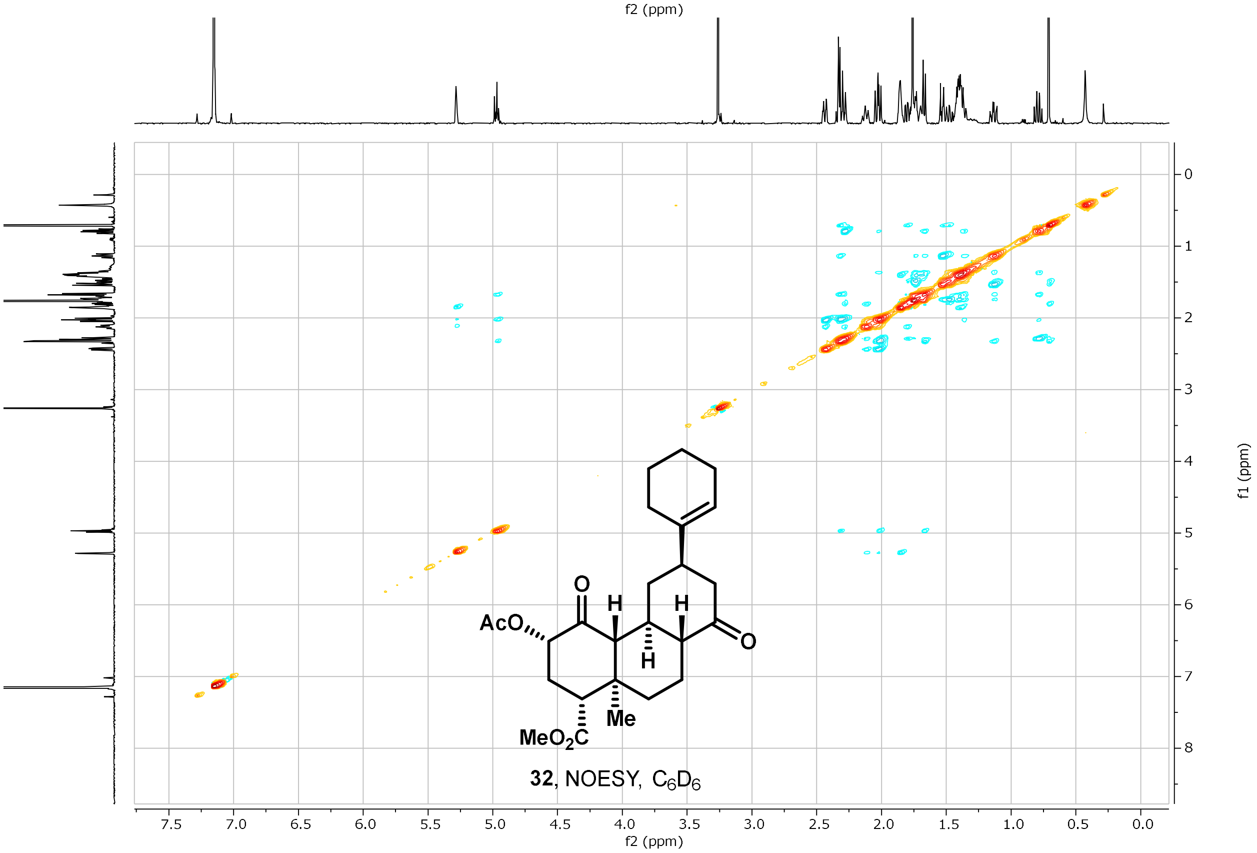

32, NOESY, C_6D_6

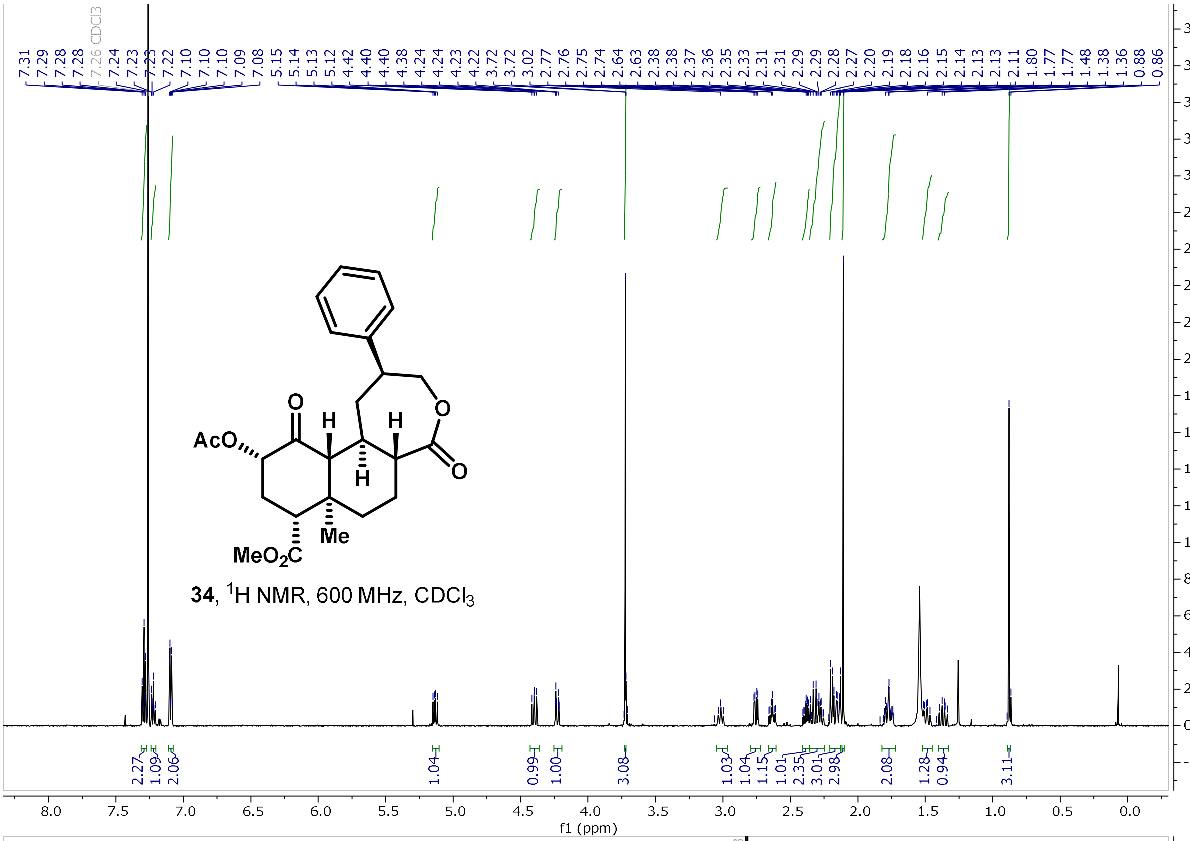
7.31 7.29 7.28 7.28 7.26 CDCl3 7.24 7.23 7.23 7.22 7.10 7.10 7.10 7.09 7.08 5.15 5.14 5.13 5.12 4.42 4.40 4.40 4.38 4.24 4.24 4.23 4.22 3.72 3.72 3.02 2.77 2.76 2.75 2.74 2.64 2.63 2.38 2.38 2.37 2.36 2.35 2.33 2.31 2.29 2.29 2.28 2.27 2.20 2.19 2.18 2.16 2.15 2.14 2.13 2.13 2.11 1.80 1.77 1.77 1.48 1.38 1.36 0.88 0.86
2.27 1.09 2.06 1.04 0.99 1.00 3.08 1.03 1.04 1.15 1.01 2.35 3.01 2.98 2.08 1.28 0.94 3.11
AcO
H
H
H
O
O
O
Me
MeO2C
34, 1H NMR, 600 MHz, CDCl3
f1 (ppm)

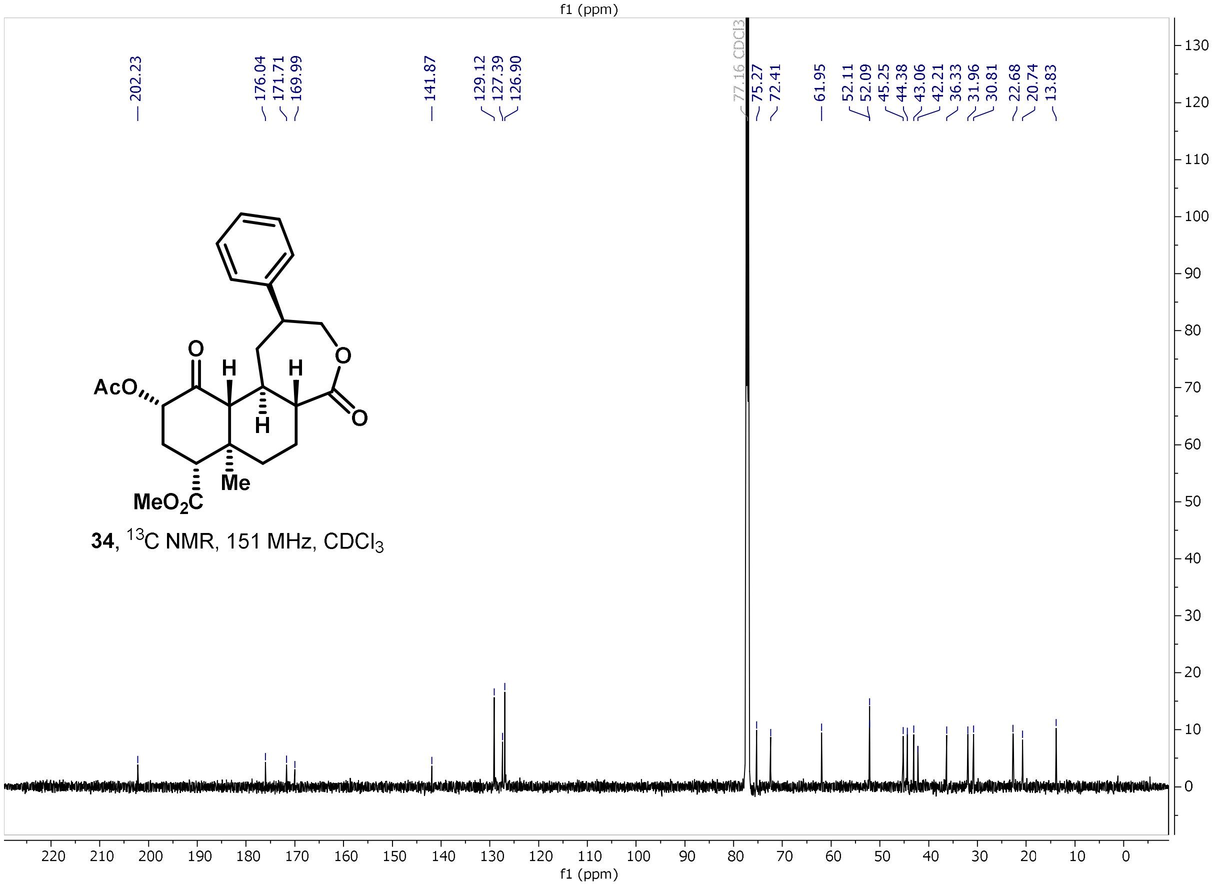
202.23 176.04 171.71 169.99 141.87 129.12 127.39 126.90 77.16 CDCl3 75.27 72.41 61.95 52.11 52.09 45.25 44.38 43.06 42.21 36.33 31.96 30.81 22.68 20.74 13.83
AcO
H
H
H
O
O
O
Me
MeO2C
34, 13C NMR, 151 MHz, CDCl3
f1 (ppm)

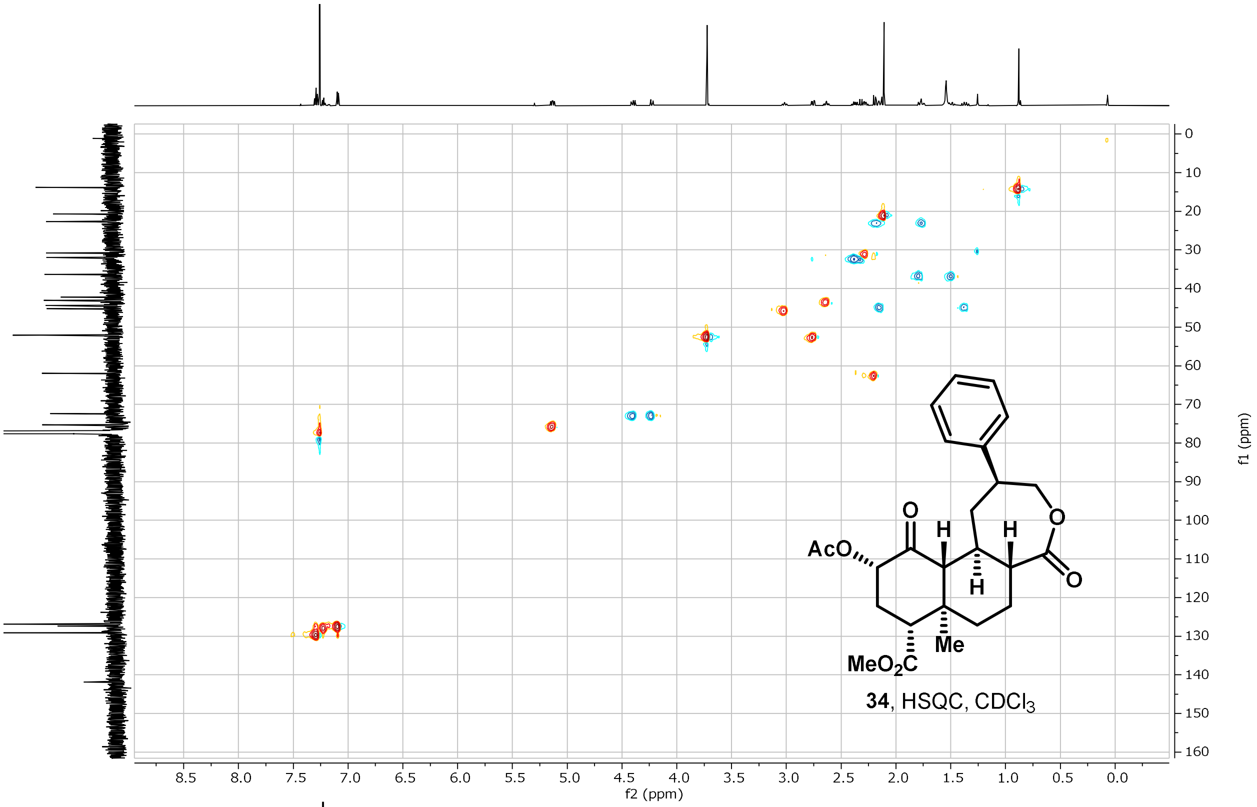
AcO
MeO2C
Me
34, HSQC, CDCl3
f2 (ppm)
f1 (ppm)

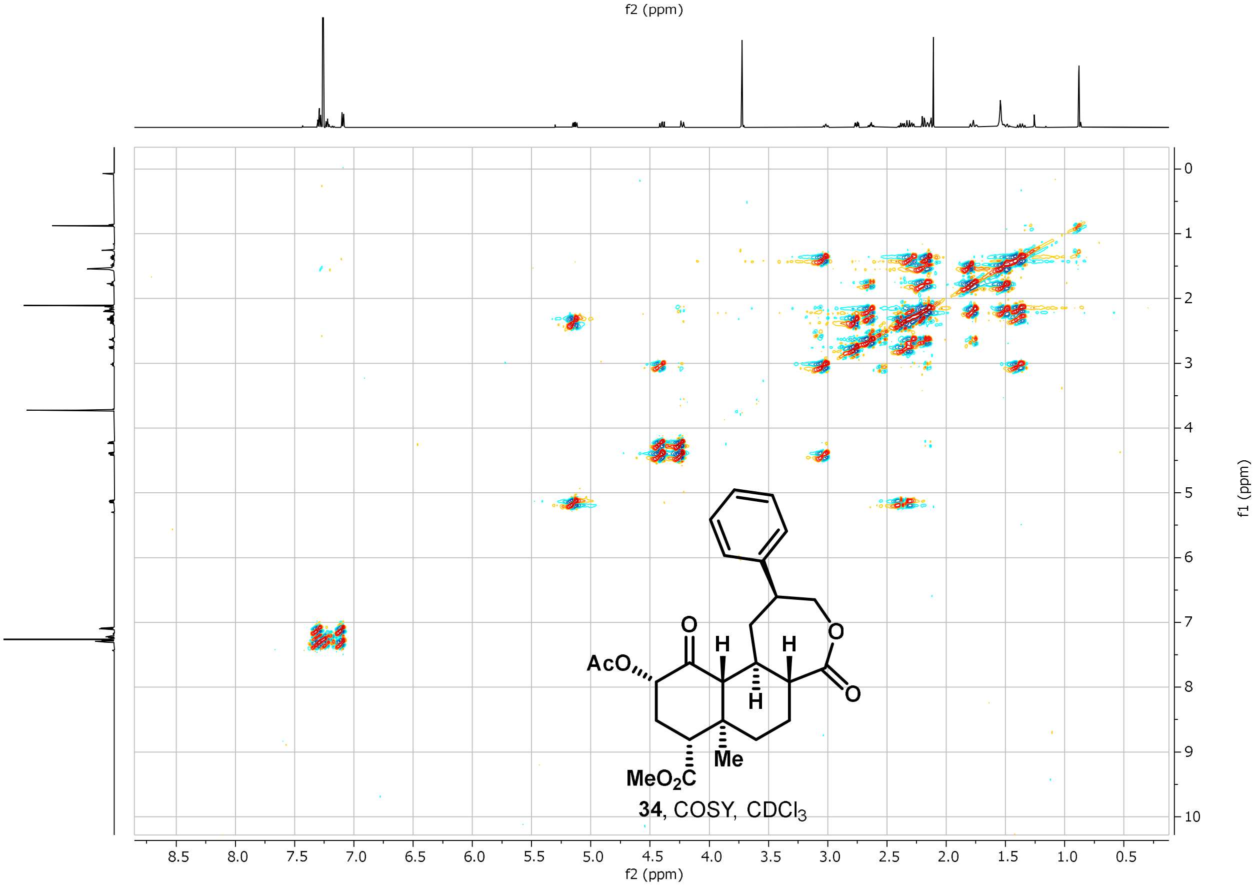
AcO
MeO2C
Me
34, COSY, CDCl3
f2 (ppm)
f1 (ppm)

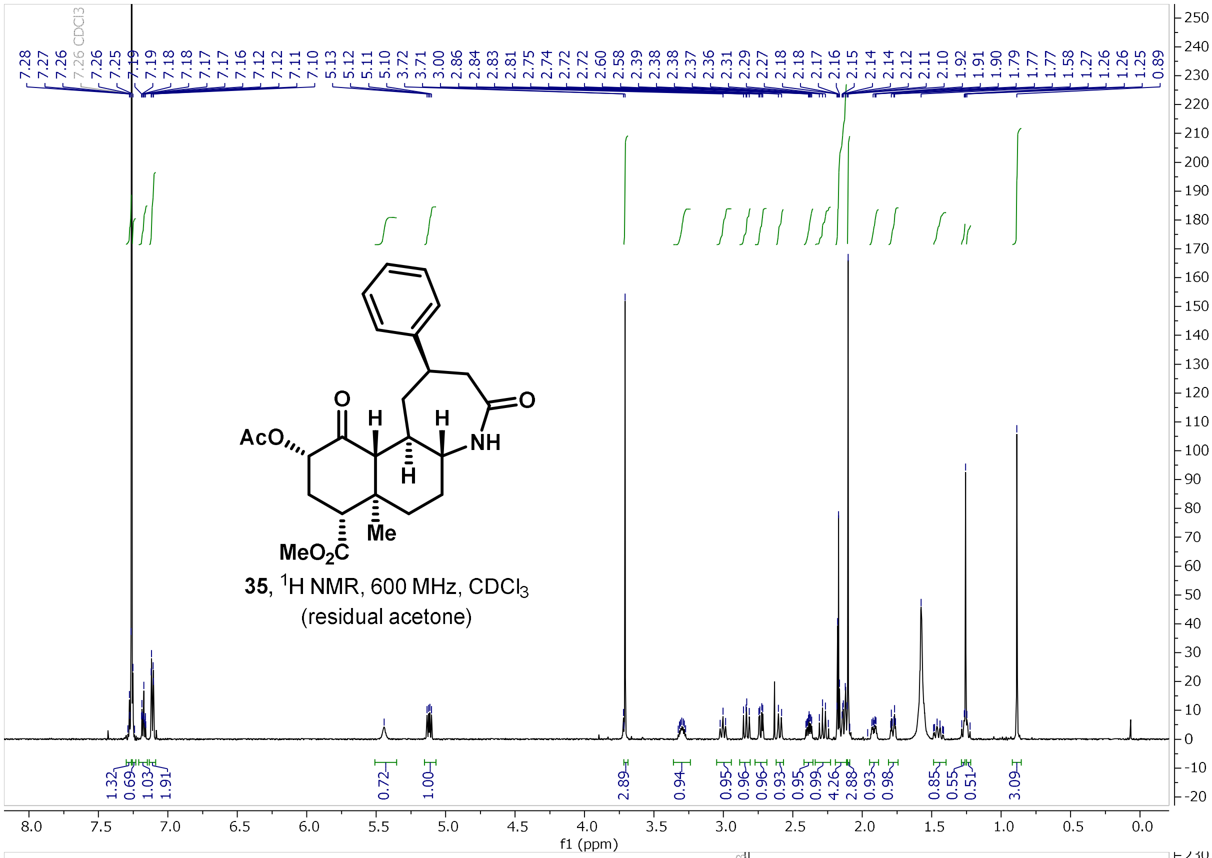

35, ^{1}H NMR, 600 MHz, $CDCl_3$
(residual acetone)

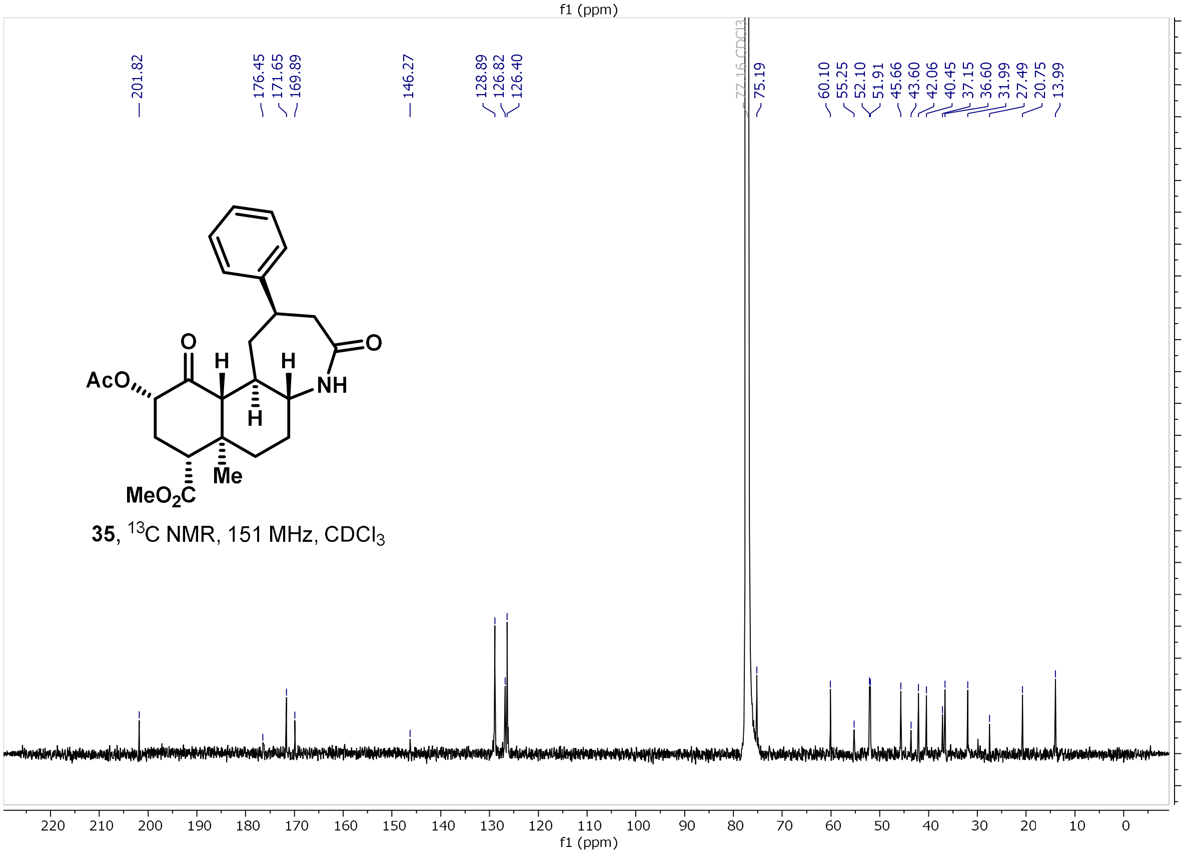

35, ^{13}C NMR, 151 MHz, $CDCl_3$

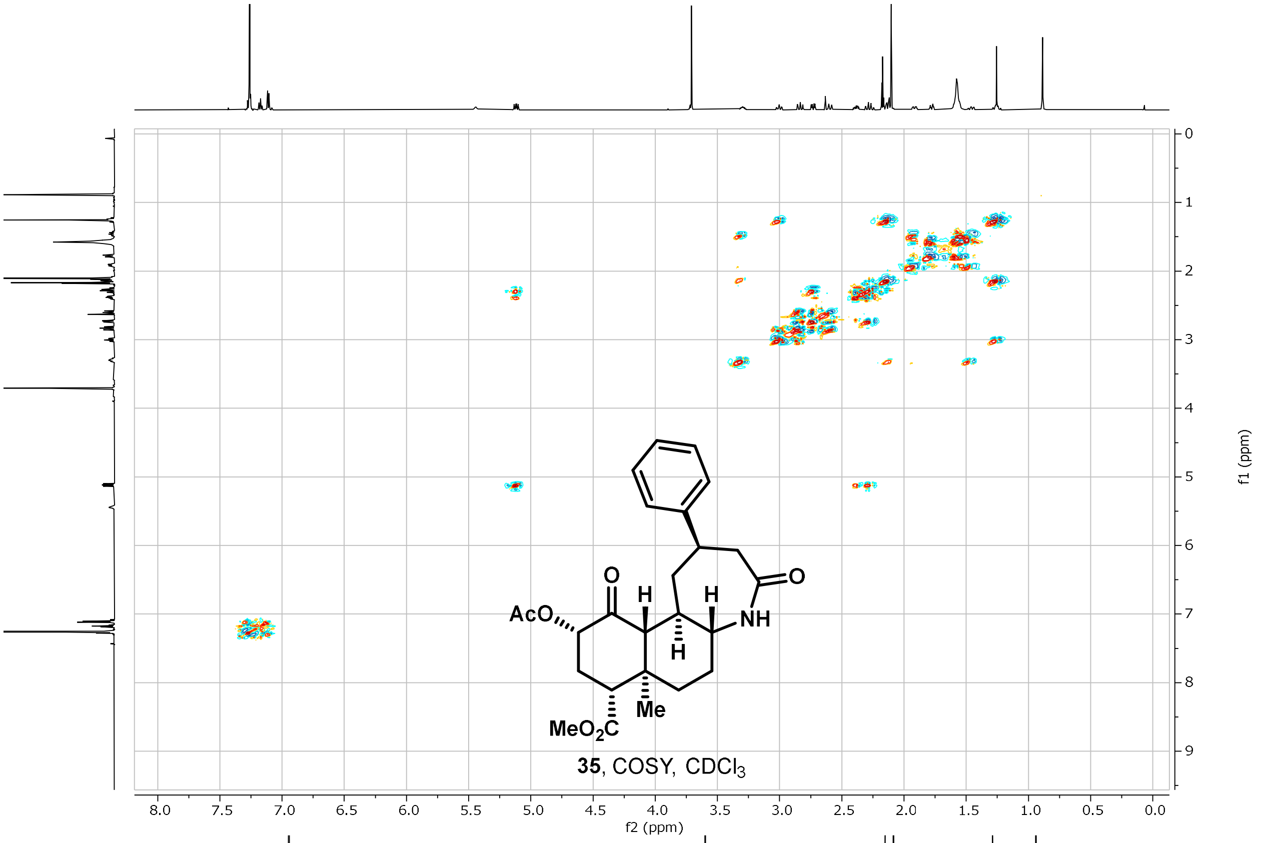
f1 (ppm)
f2 (ppm)
AcO
O
H
H
H
NH
O
Me
MeO2C
35, COSY, CDCl3

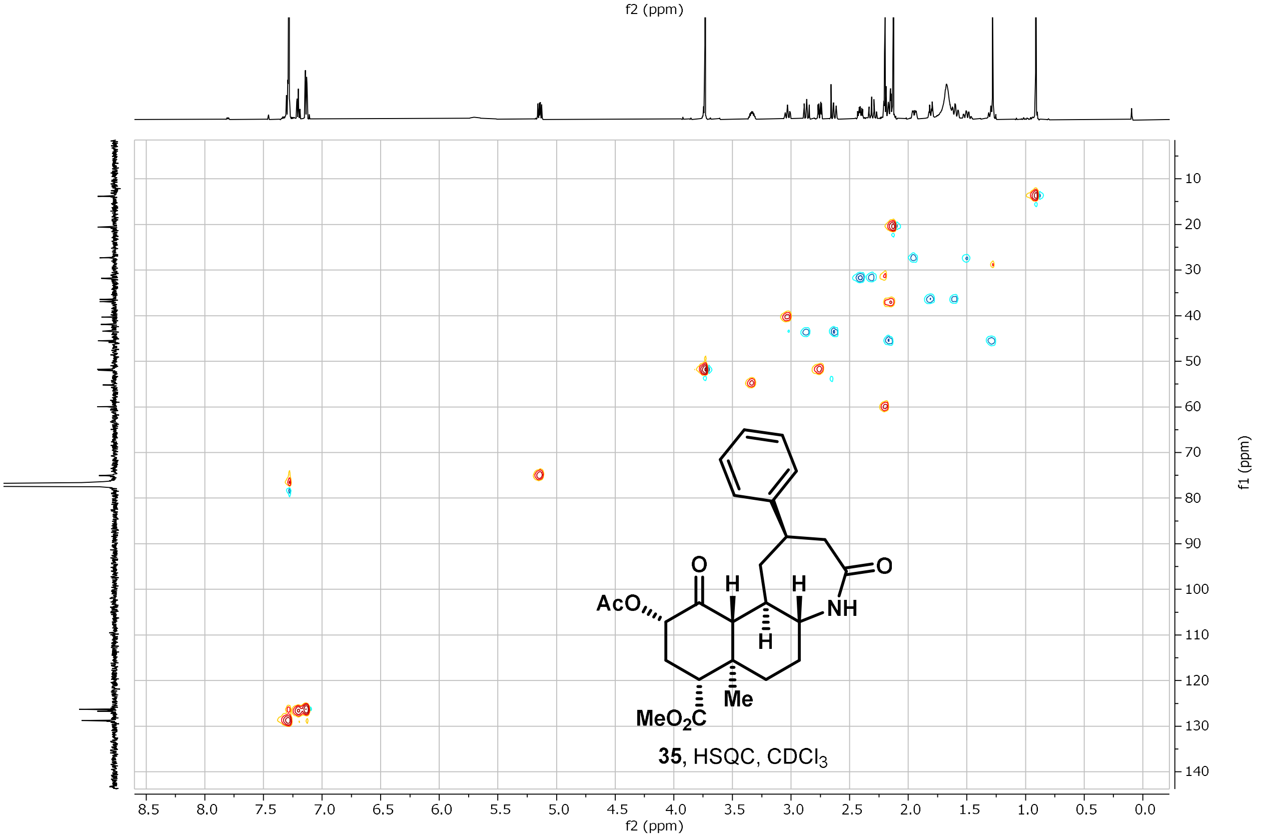
f1 (ppm)
f2 (ppm)
AcO
O
H
H
H
NH
O
Me
MeO2C
35, HSQC, CDCl3

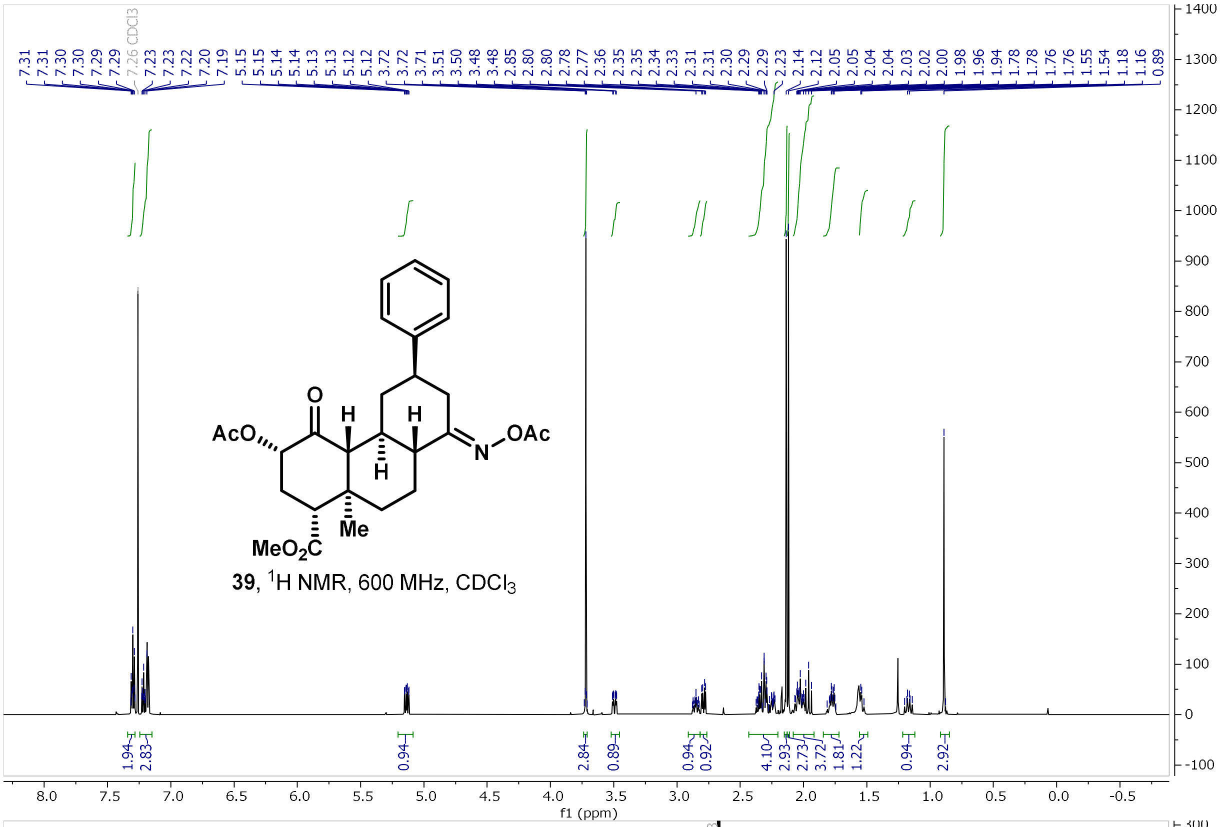

39, ^{1}H NMR, 600 MHz, $CDCl_3$

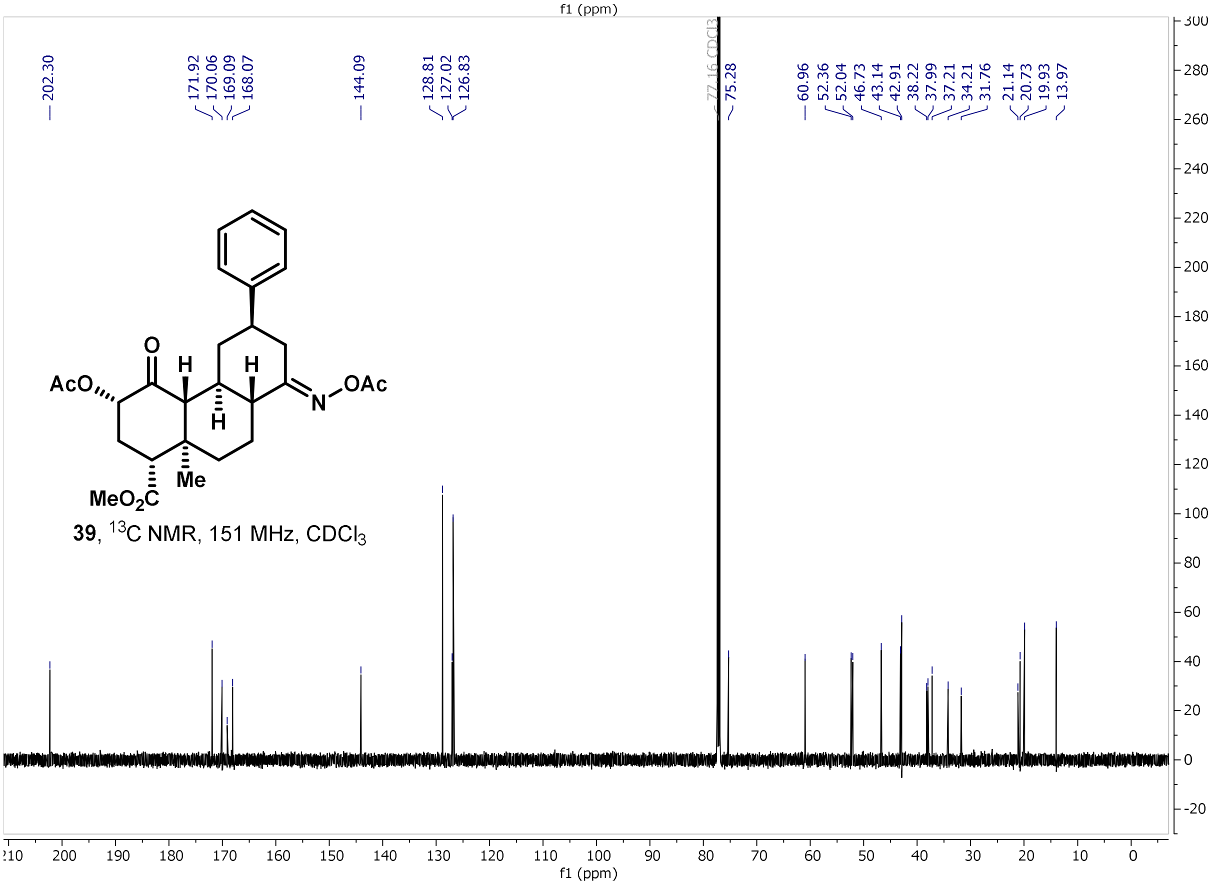

39, ^{13}C NMR, 151 MHz, $CDCl_3$

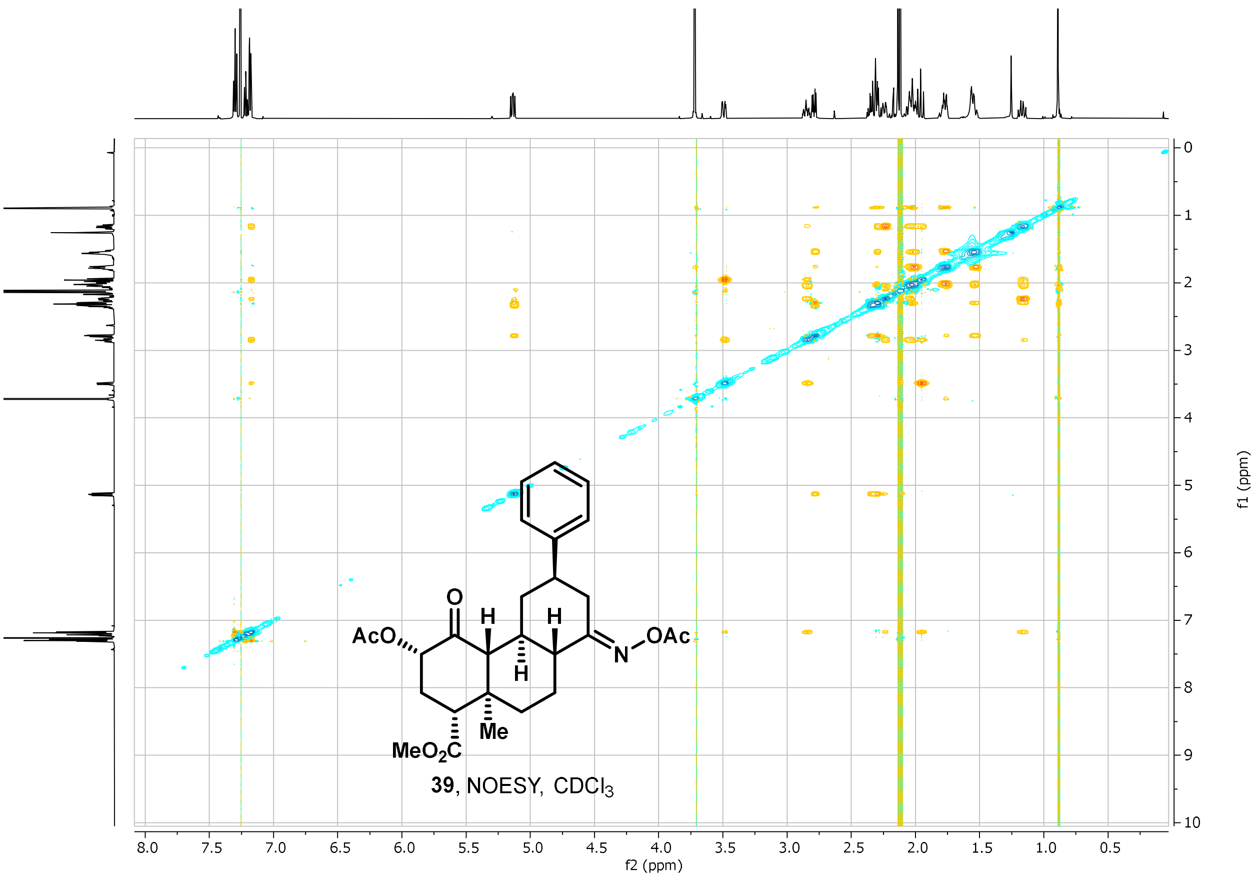
AcO
O
H
H
H
N
OAc
MeO2C
Me
39, NOESY, CDCl3
f2 (ppm)
f1 (ppm)

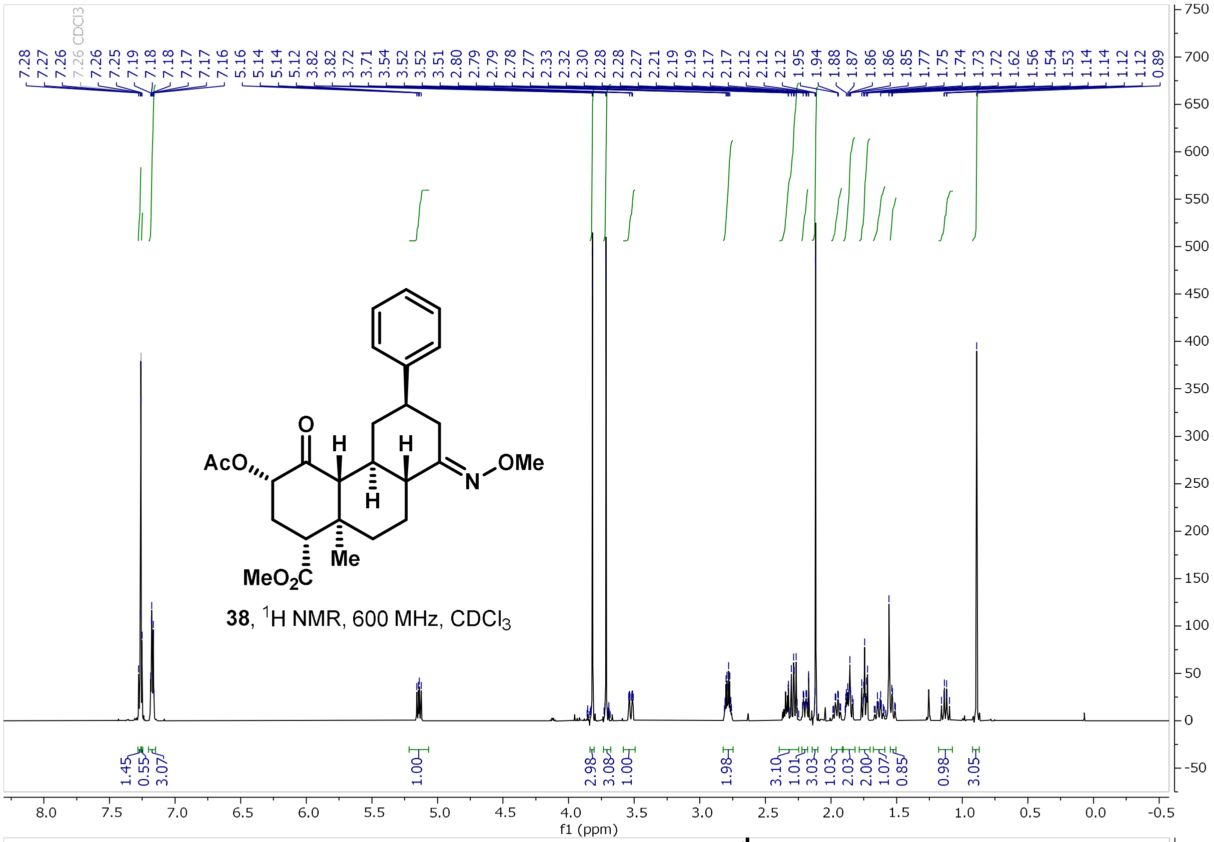

38, ^{1}H NMR, 600 MHz, $CDCl_3$

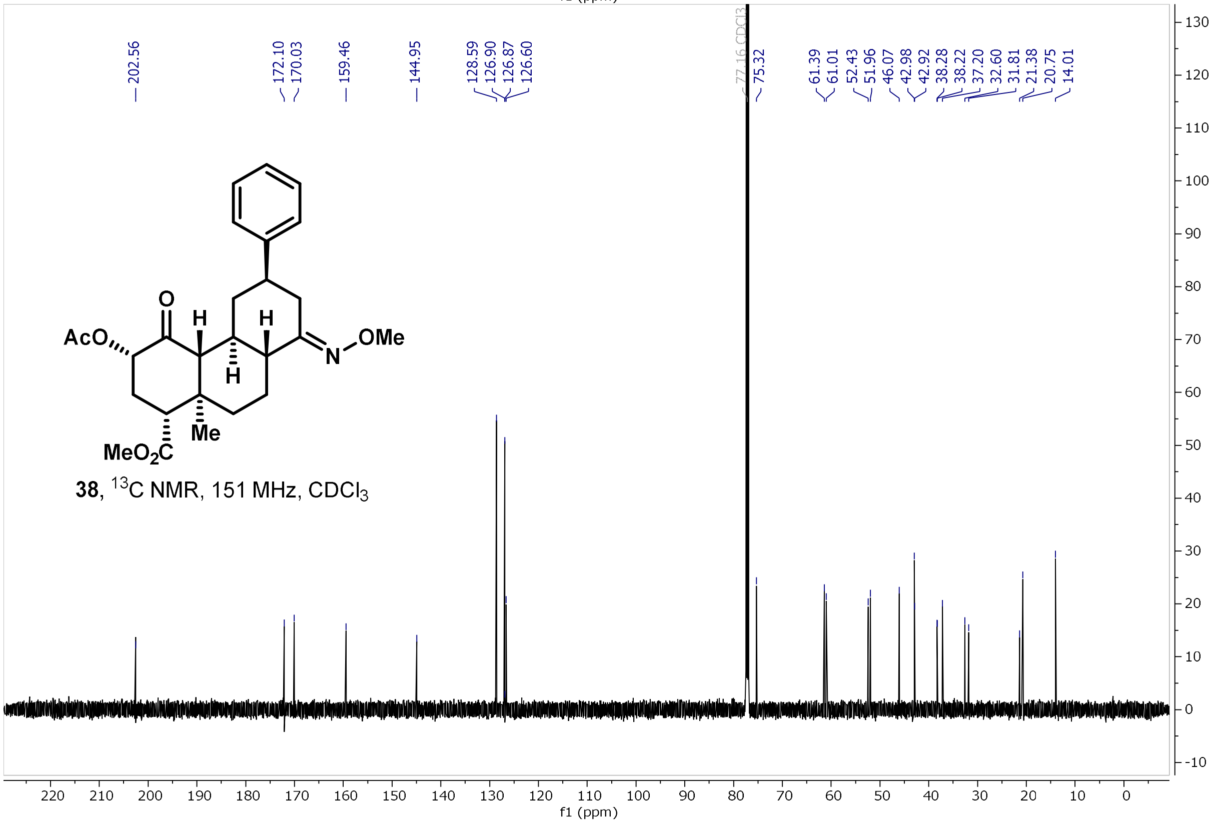

38, ^{13}C NMR, 151 MHz, $CDCl_3$

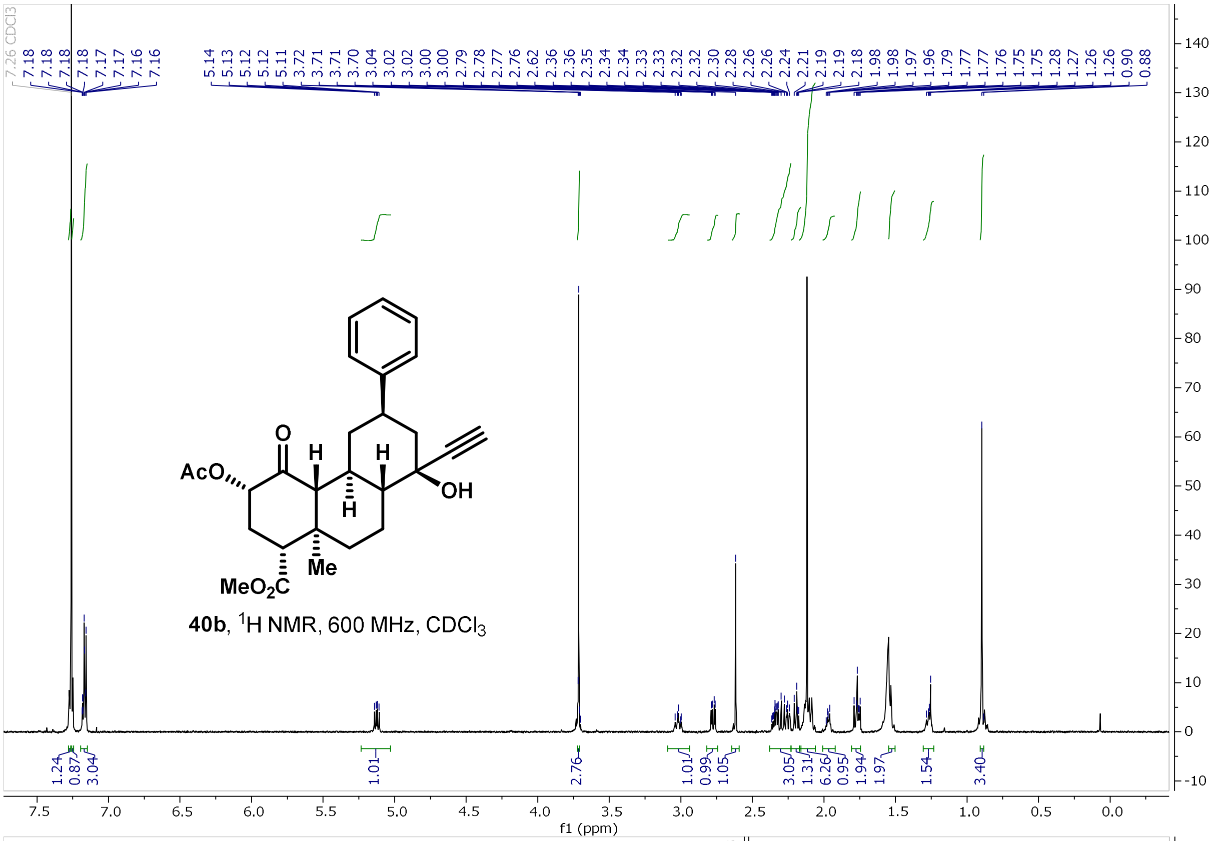

40b, ^{1}H NMR, 600 MHz, $CDCl_3$

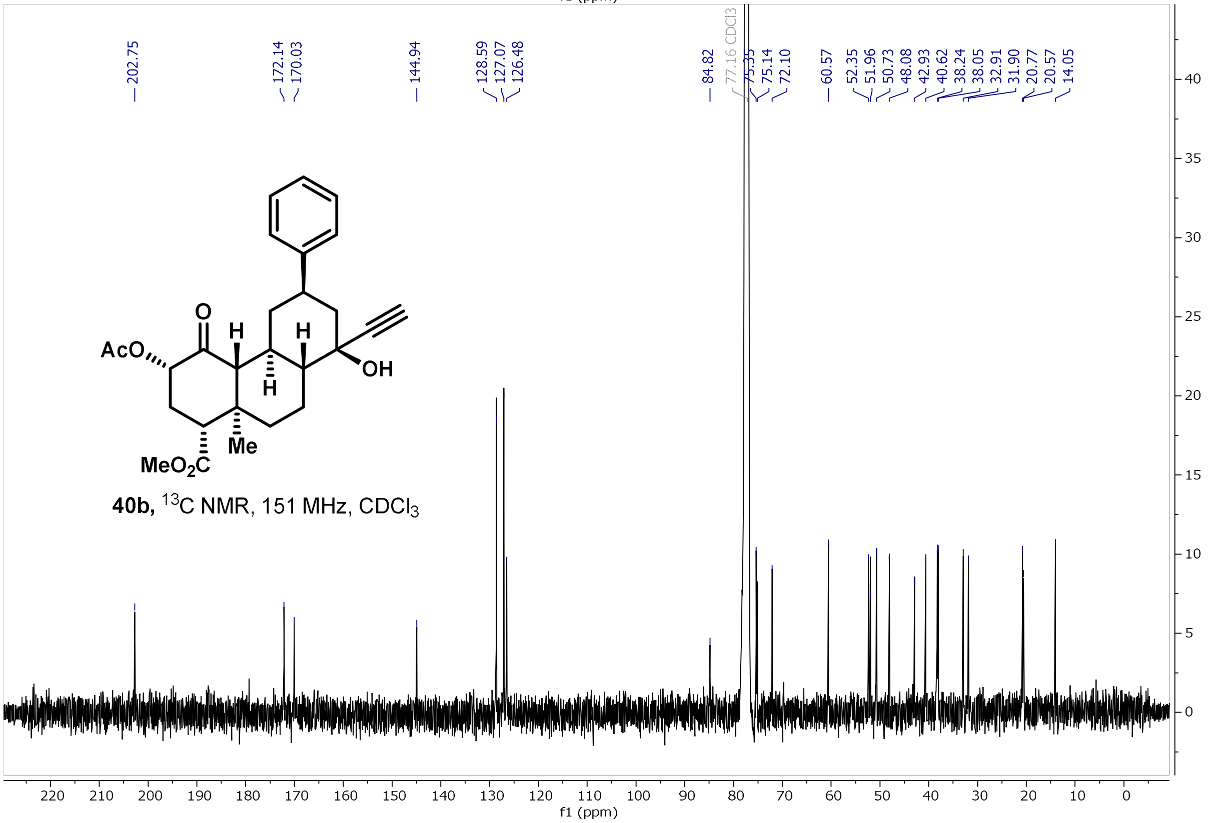

40b, ^{13}C NMR, 151 MHz, $CDCl_3$

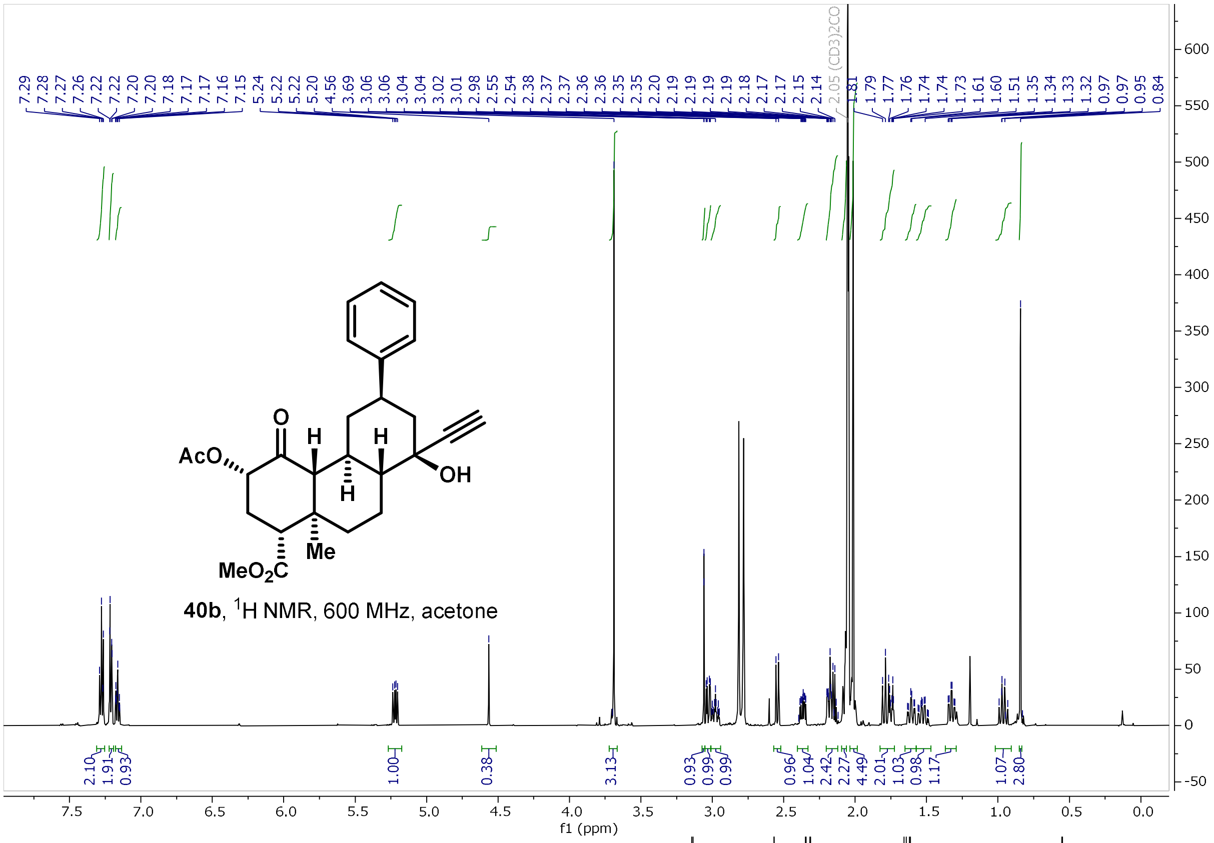

40b, ^{1}H NMR, 600 MHz, acetone

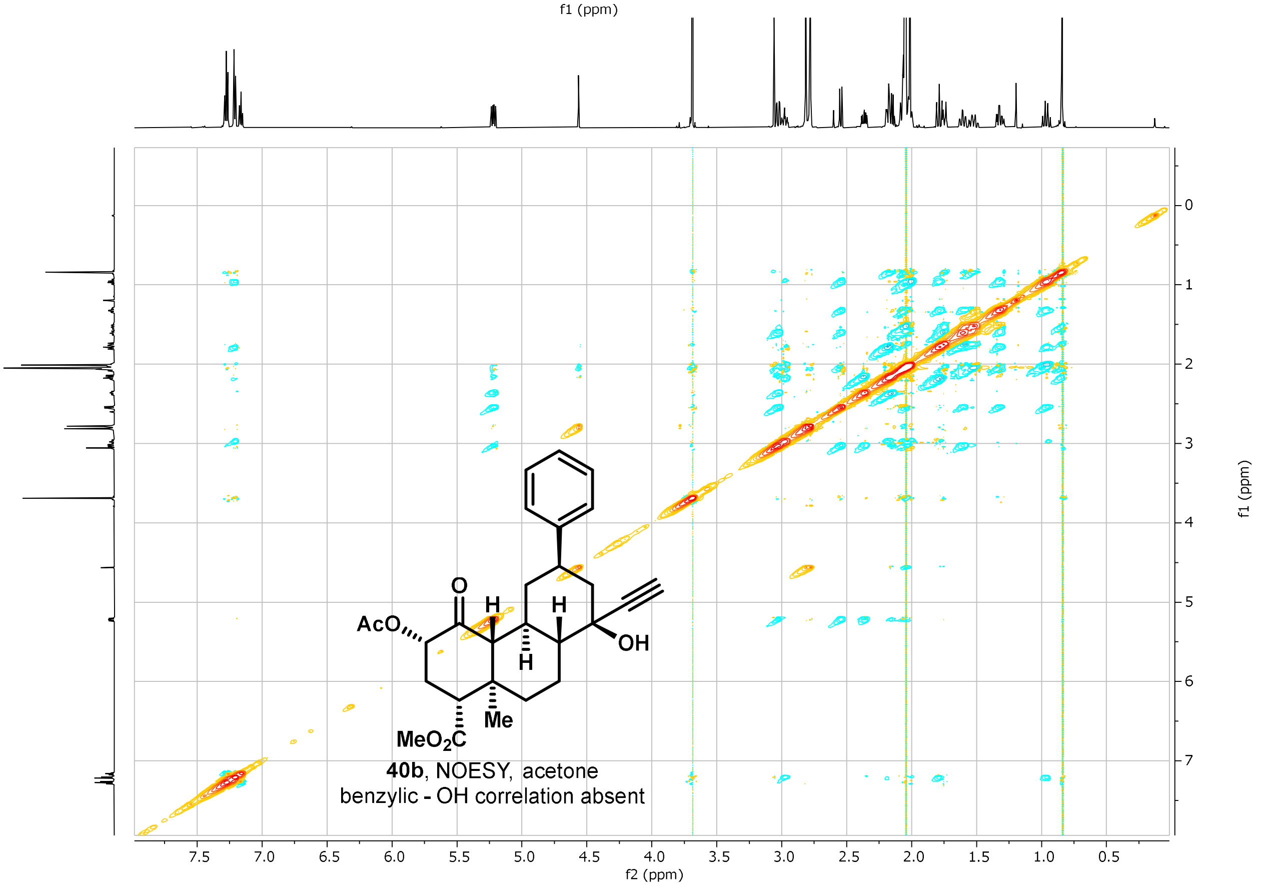

40b, NOESY, acetone
benzylic - OH correlation absent

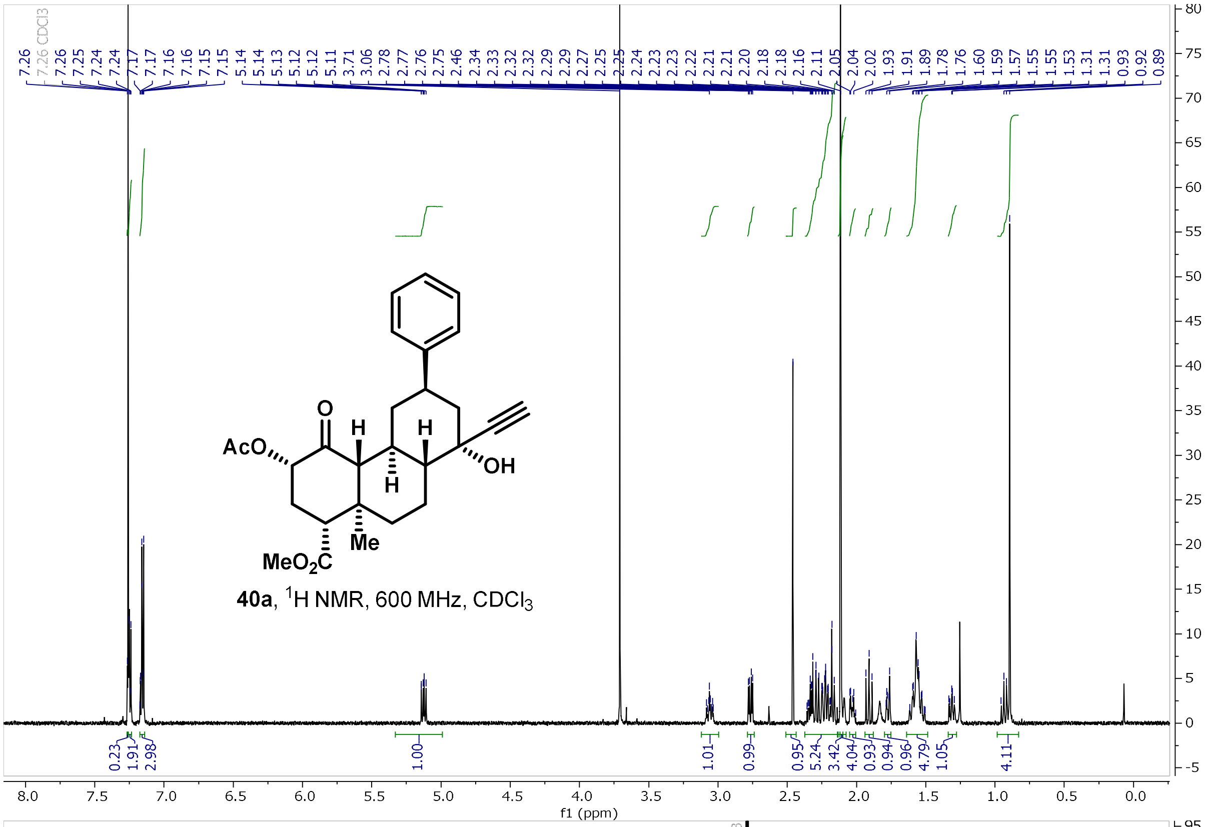

40a, ^{1}H NMR, 600 MHz, $CDCl_3$

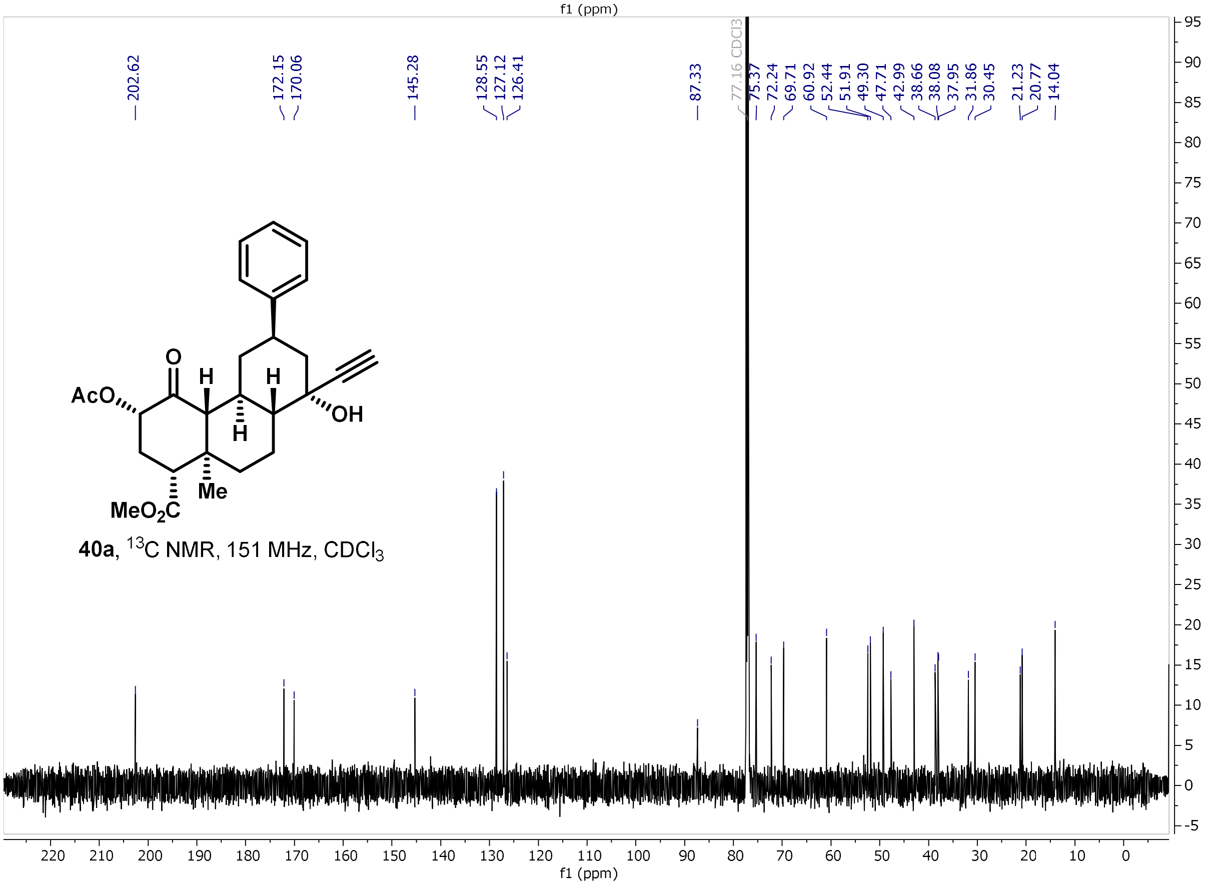

40a, ^{13}C NMR, 151 MHz, $CDCl_3$

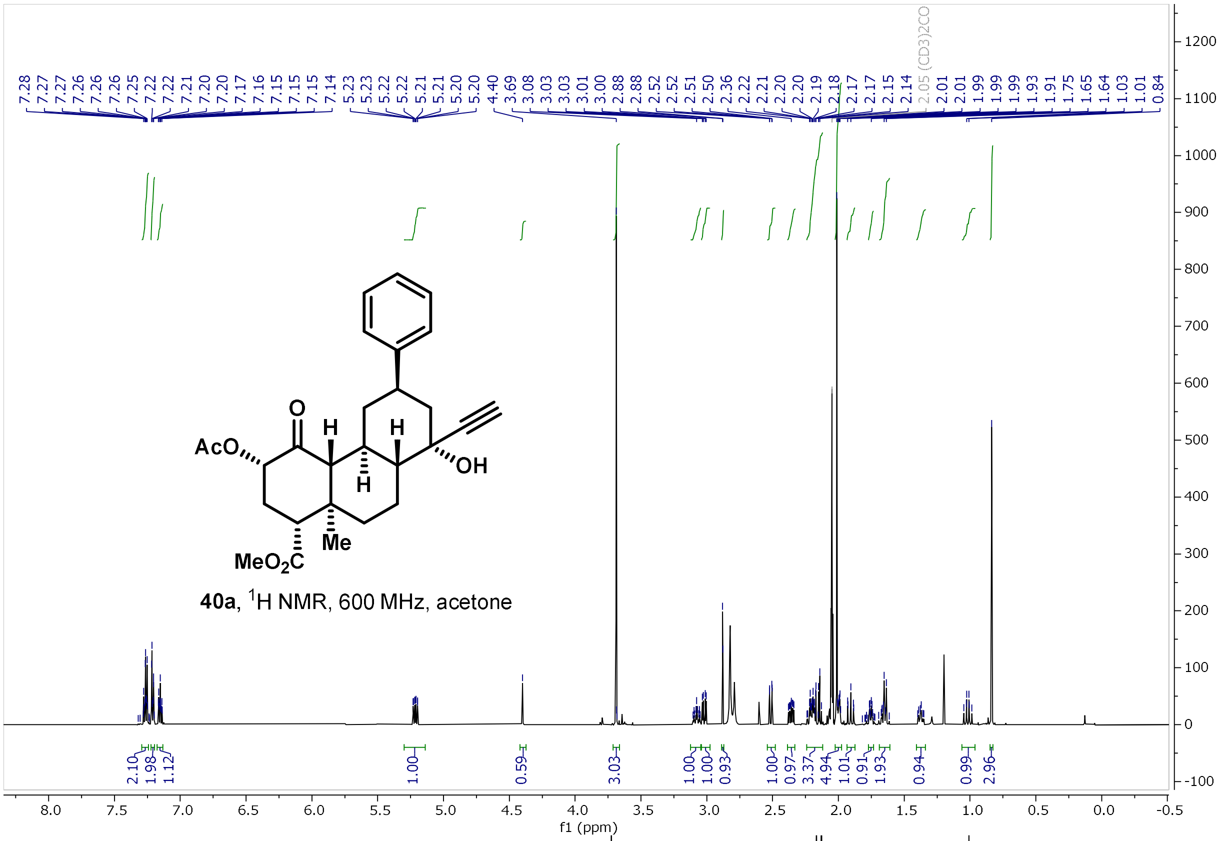

40a, ^{1}H NMR, 600 MHz, acetone

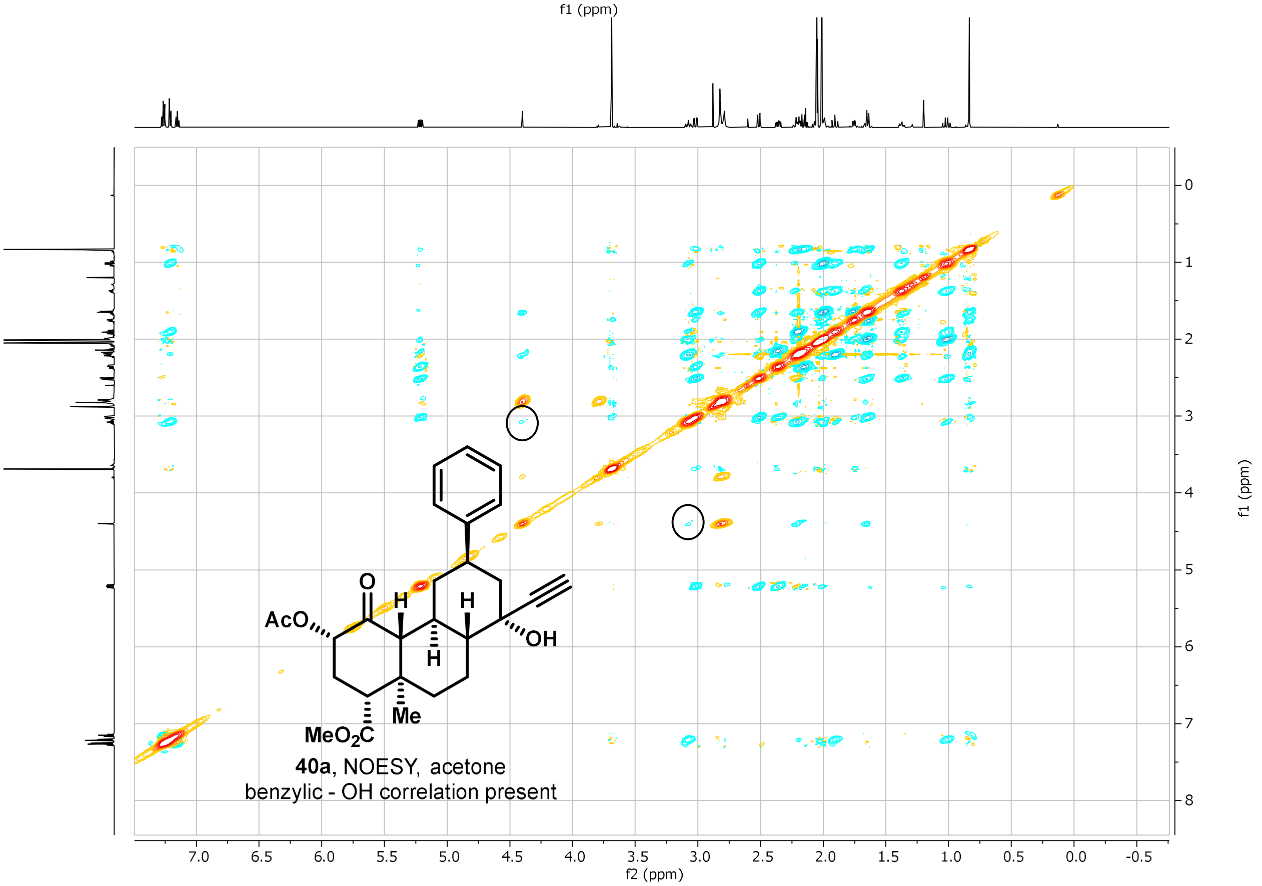

40a, NOESY, acetone
benzylic - OH correlation present

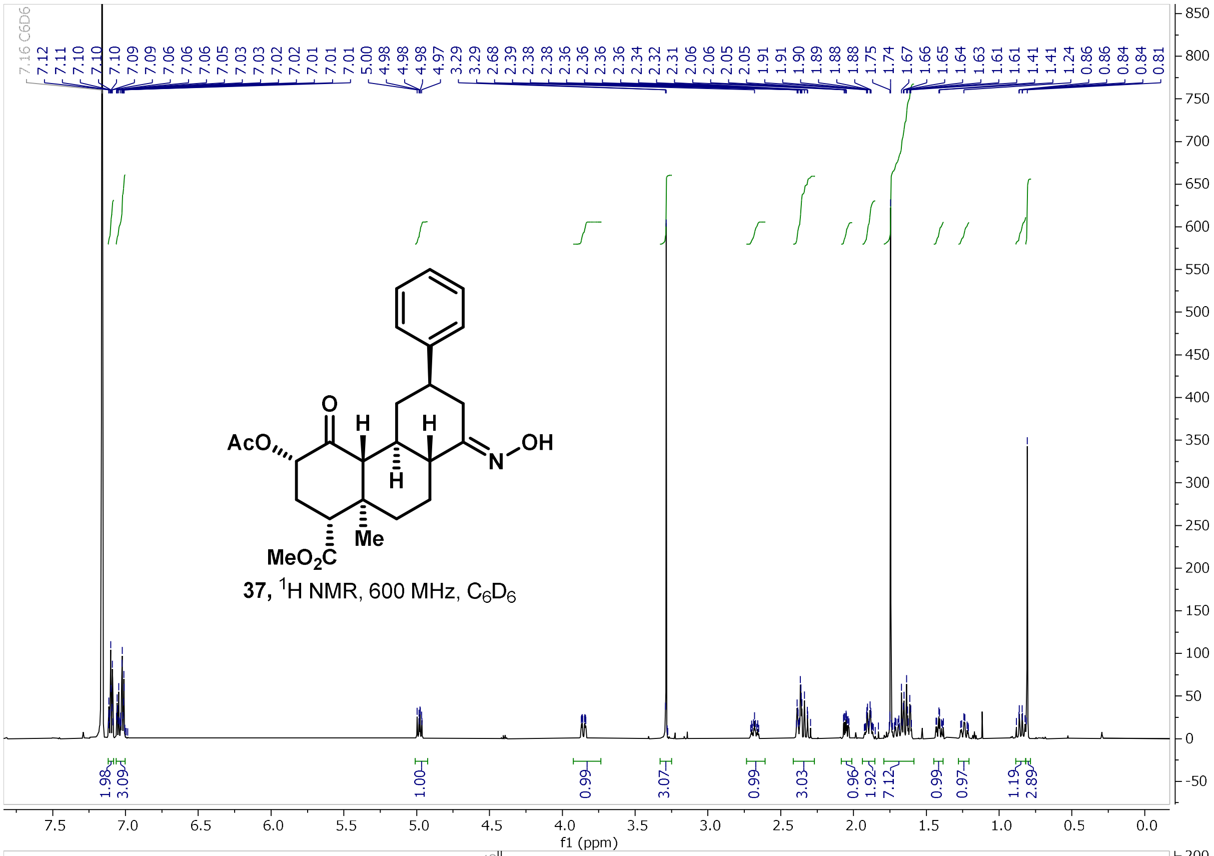

37, ^{1}H NMR, 600 MHz, C_6D_6

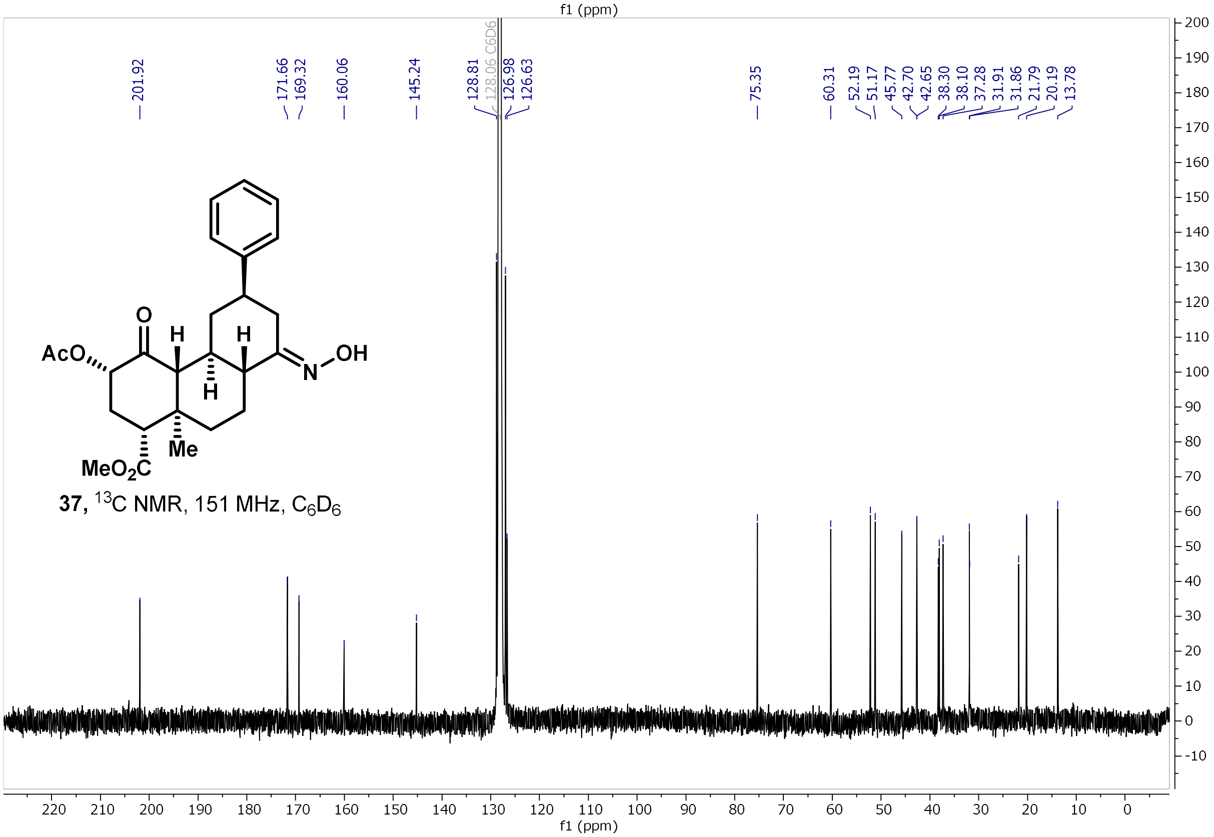

37, ^{13}C NMR, 151 MHz, C_6D_6

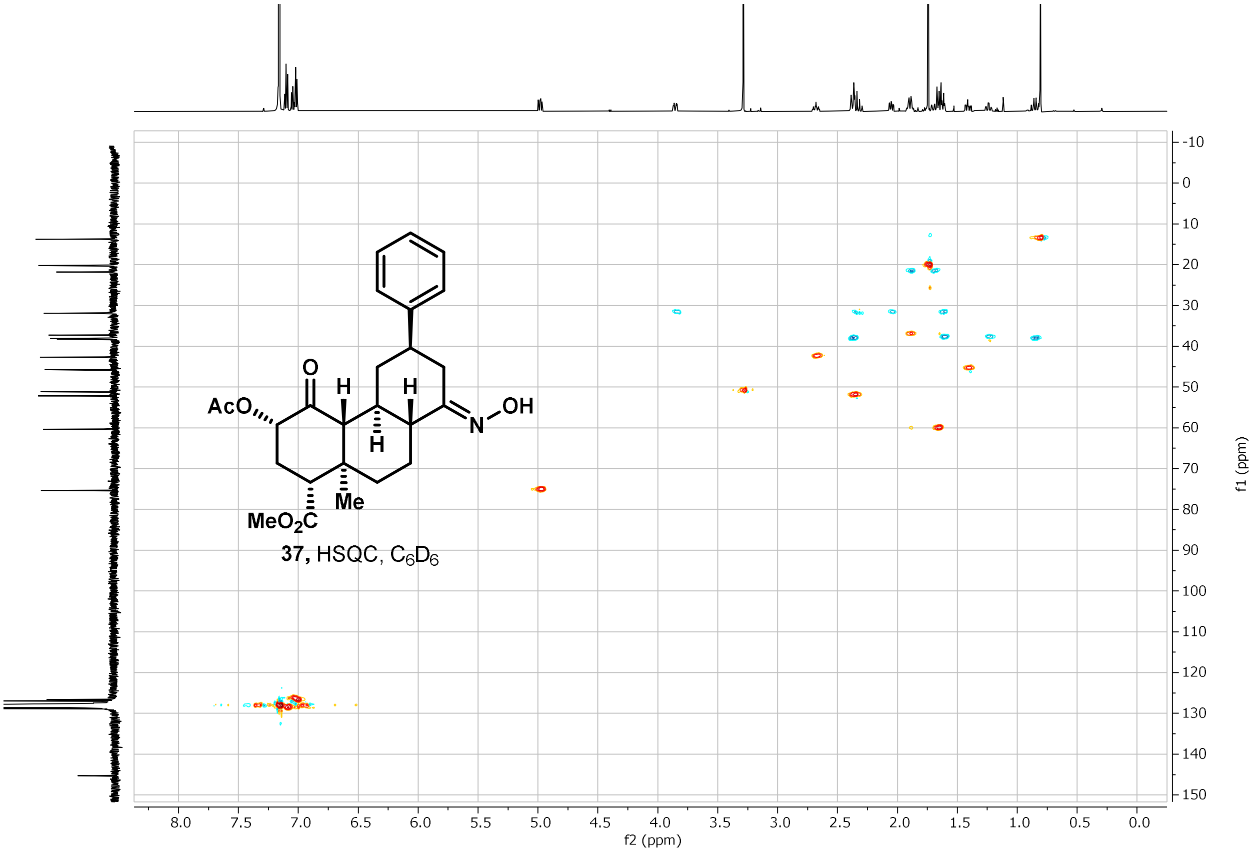
AcO
O
H
H
H
N
OH
Me
MeO2C
37, HSQC, C6D6
f2 (ppm)
f1 (ppm)

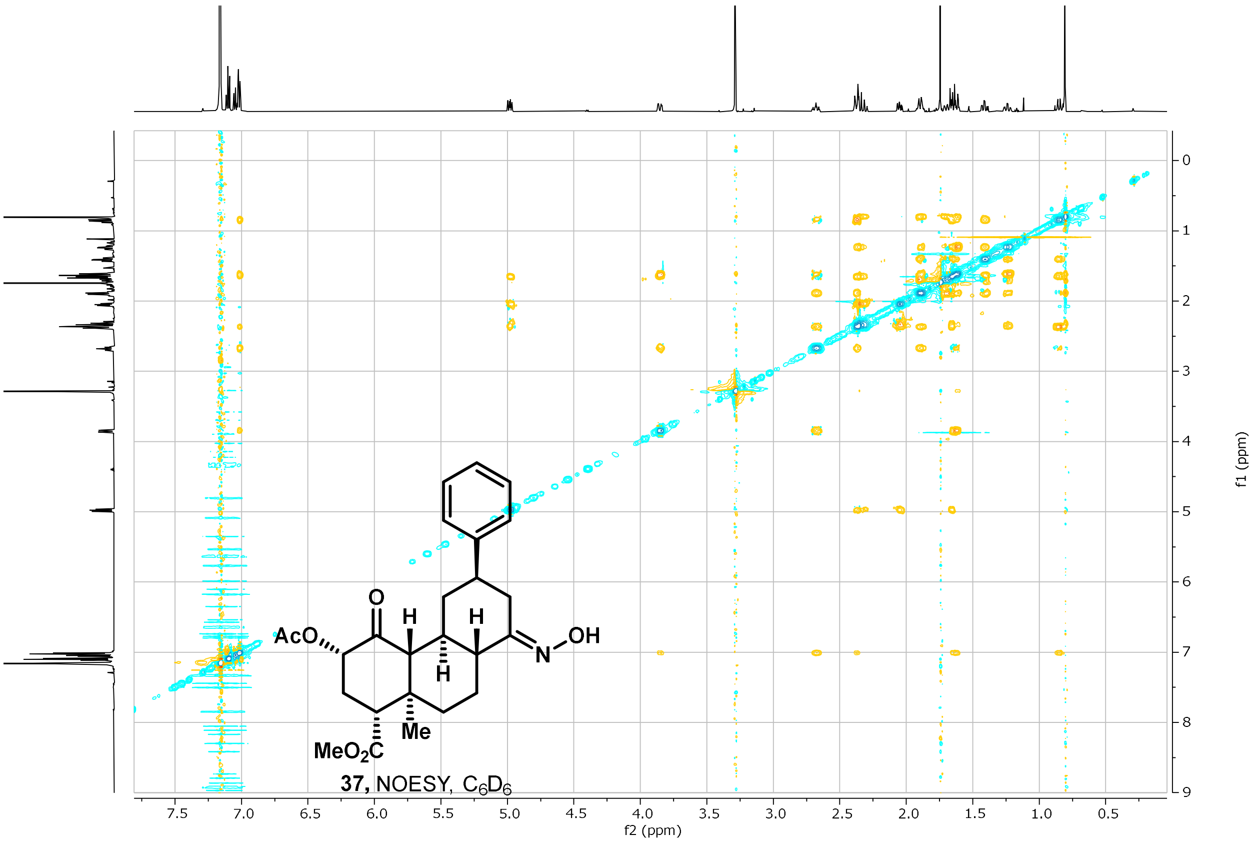
AcO
O
H
H
H
N
OH
Me
MeO2C
37, NOESY, C6D6
f2 (ppm)
f1 (ppm)

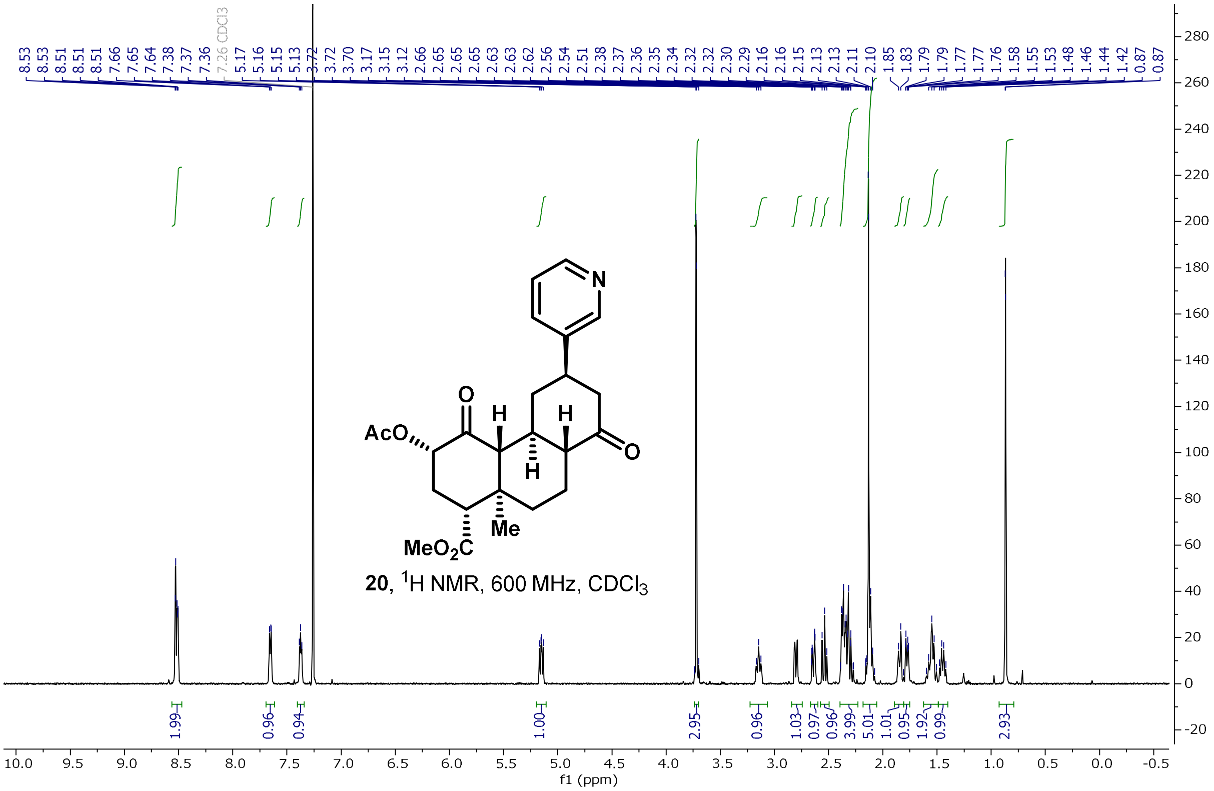
20, ^{1}H NMR, 600 MHz, CDCl$_3$

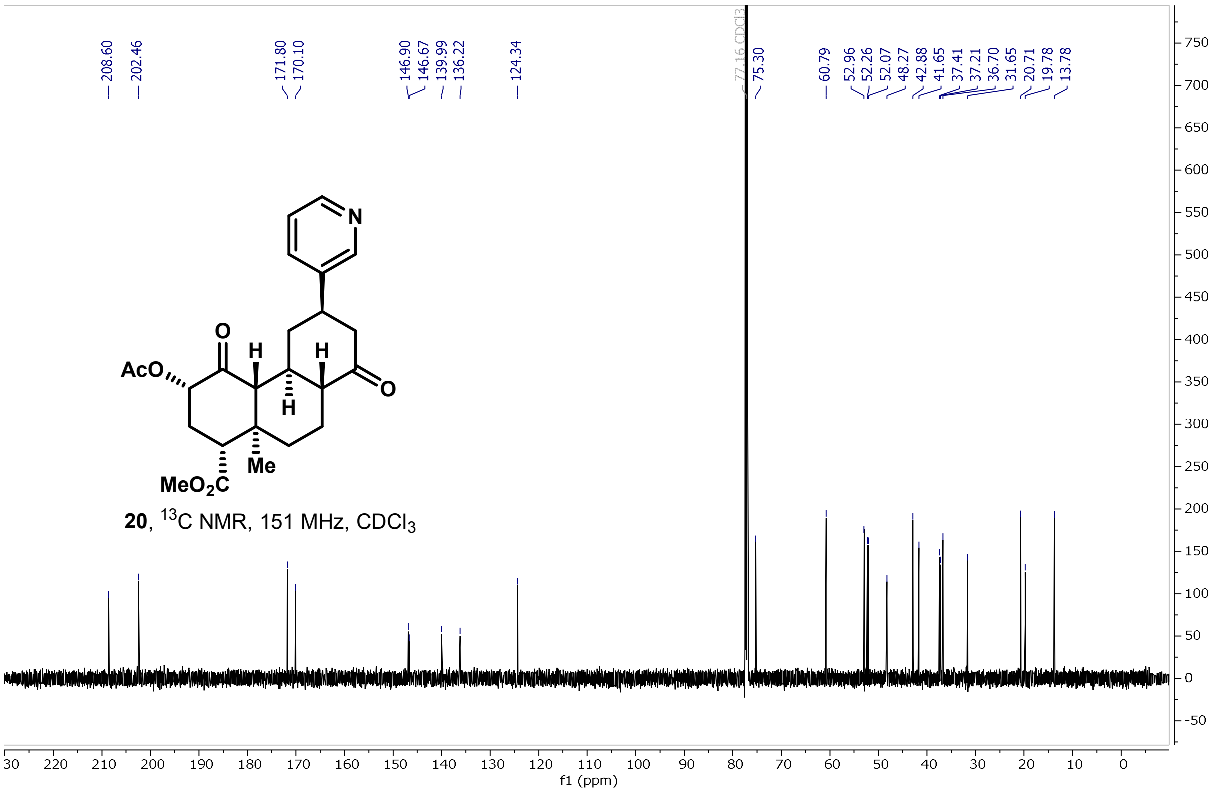
20, ^{13}C NMR, 151 MHz, CDCl$_3$

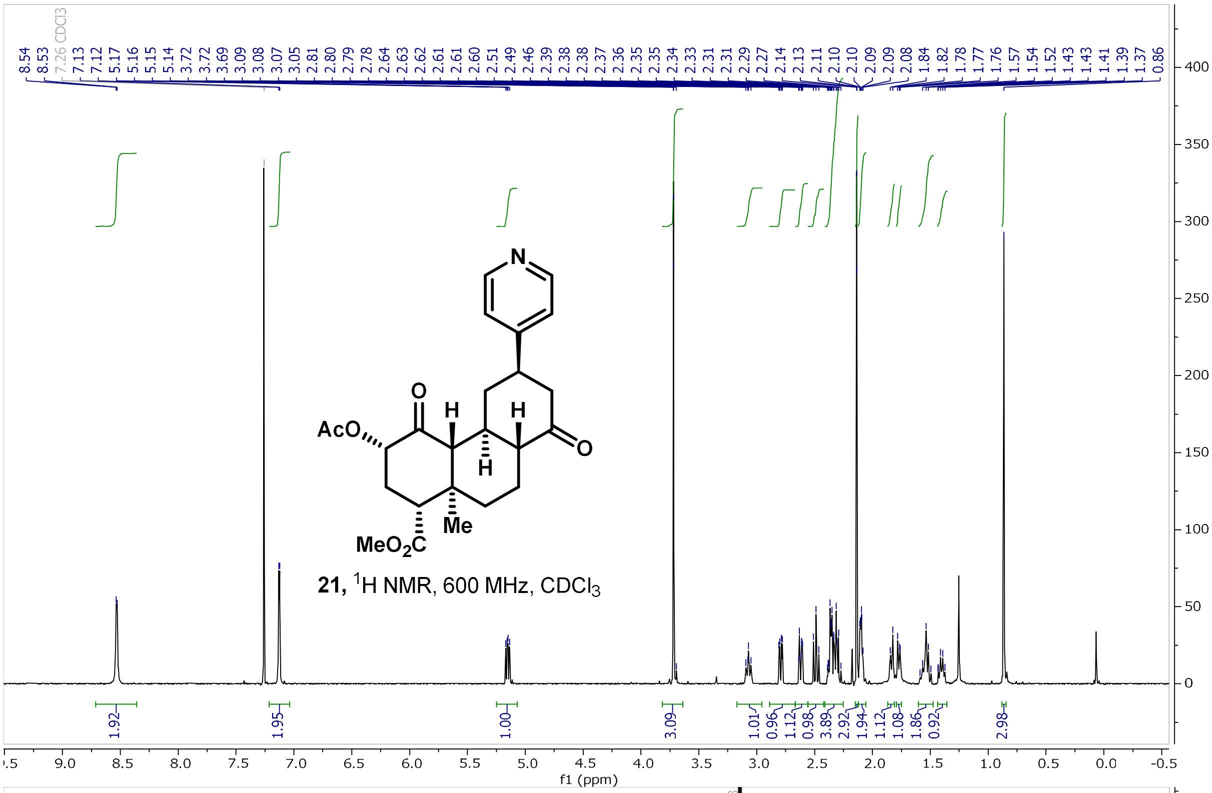

21, ^{1}H NMR, 600 MHz, $CDCl_3$

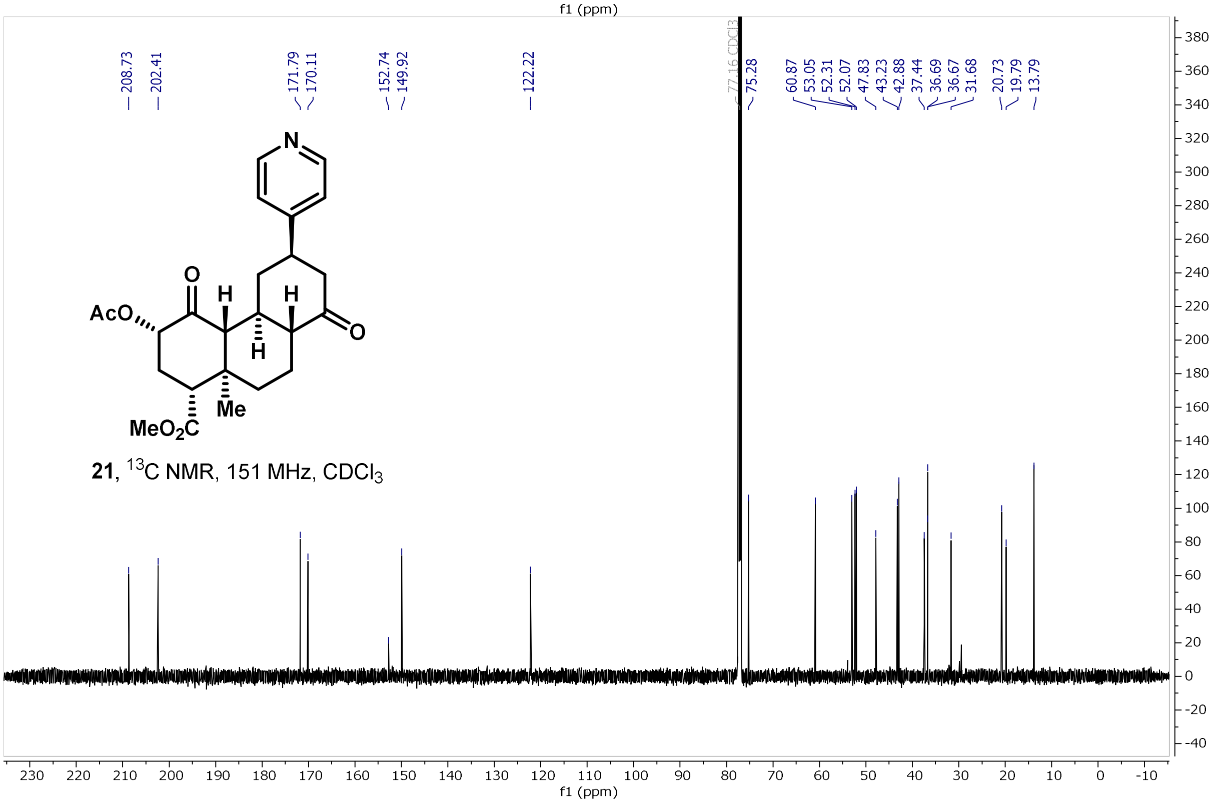

21, ^{13}C NMR, 151 MHz, $CDCl_3$

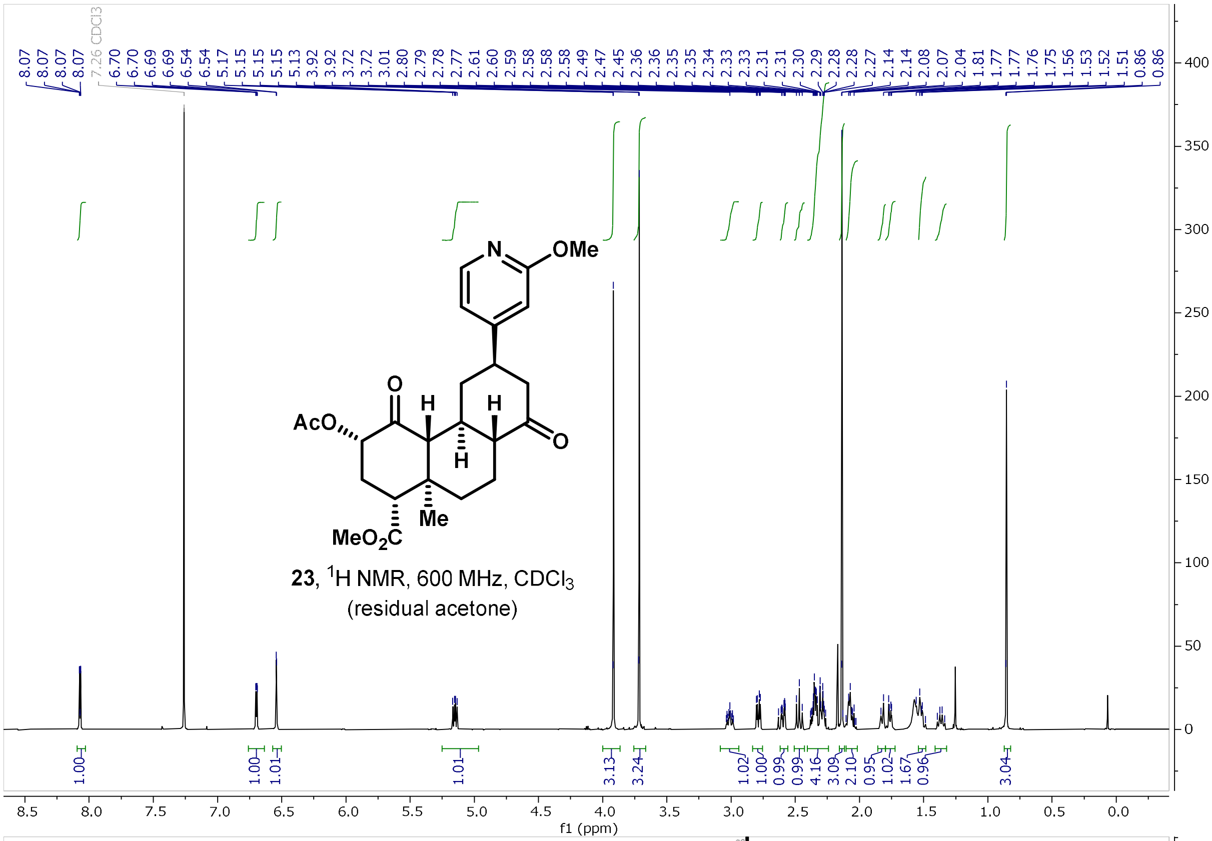

23, ^{1}H NMR, 600 MHz, $CDCl_3$
(residual acetone)

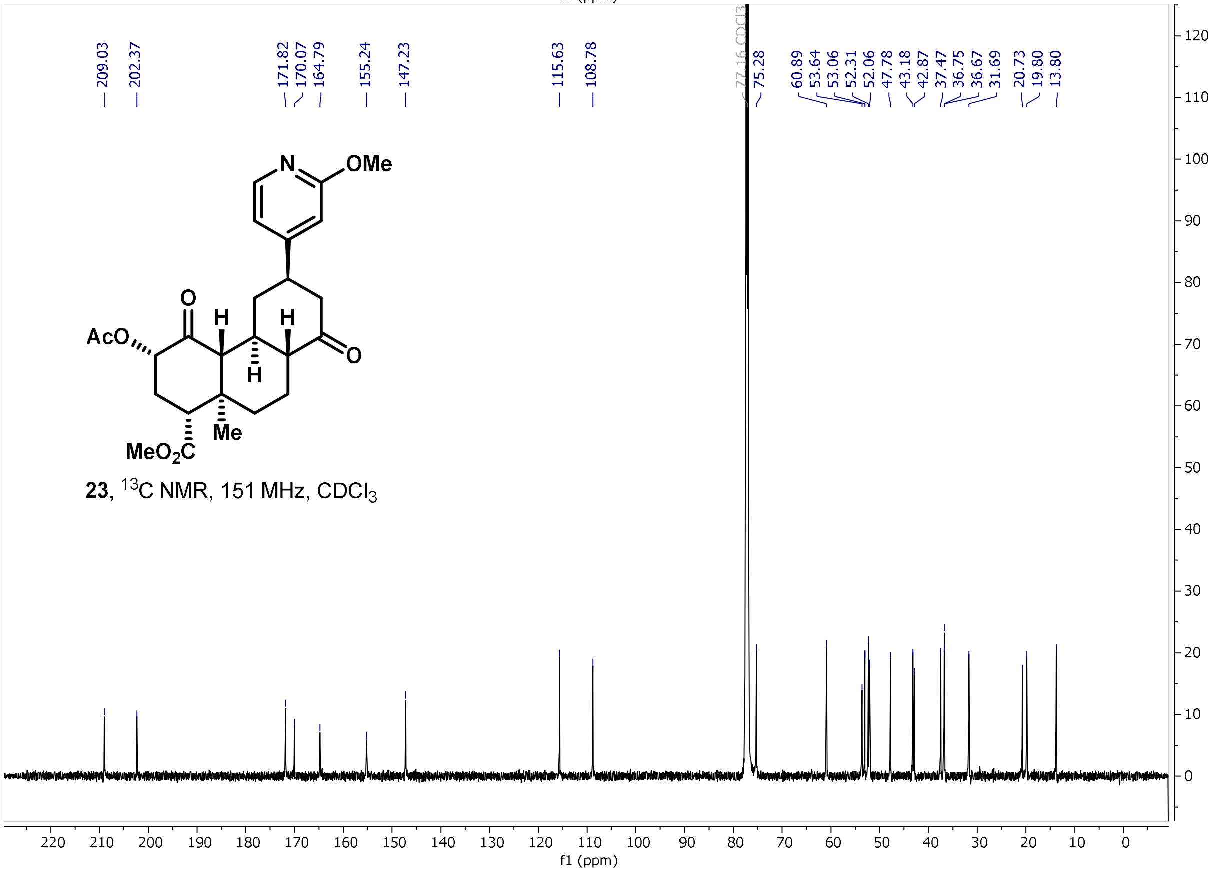

23, ^{13}C NMR, 151 MHz, $CDCl_3$

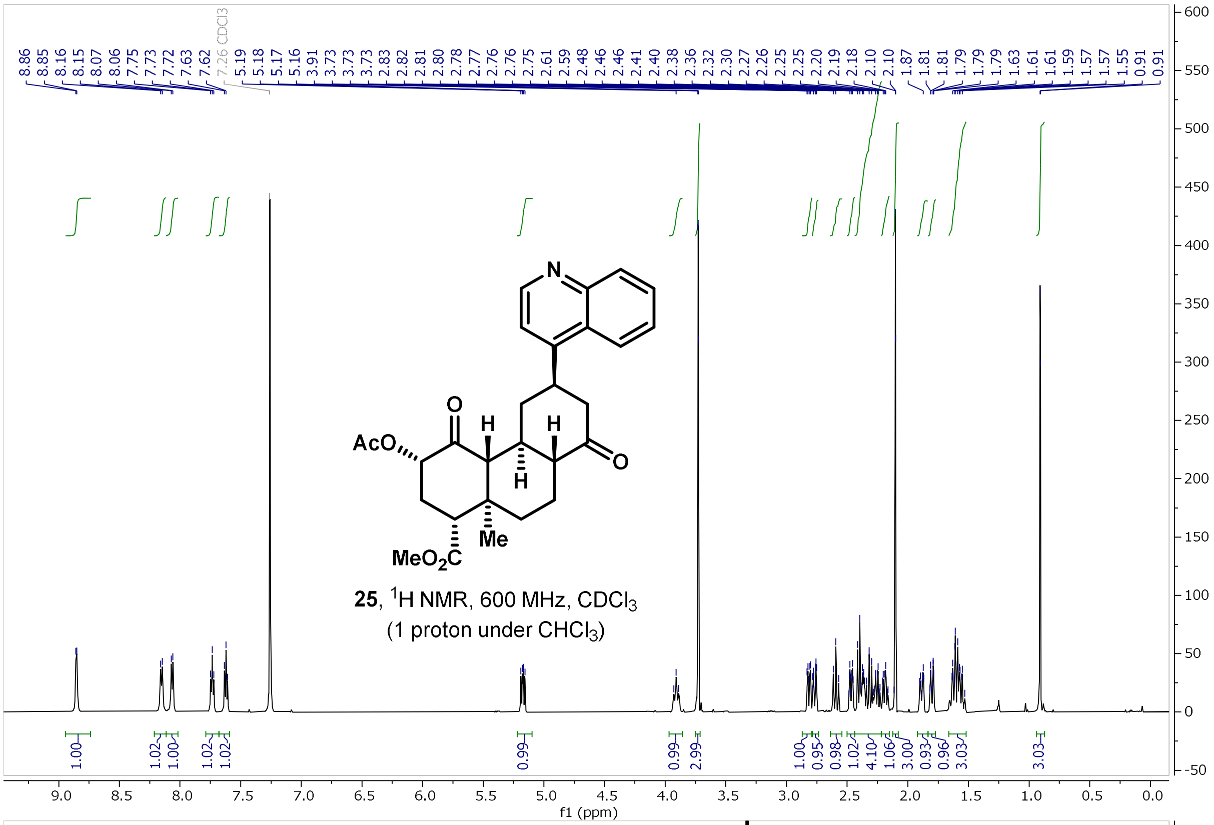

25, ^{1}H NMR, 600 MHz, $CDCl_3$
(1 proton under $CHCl_3$)

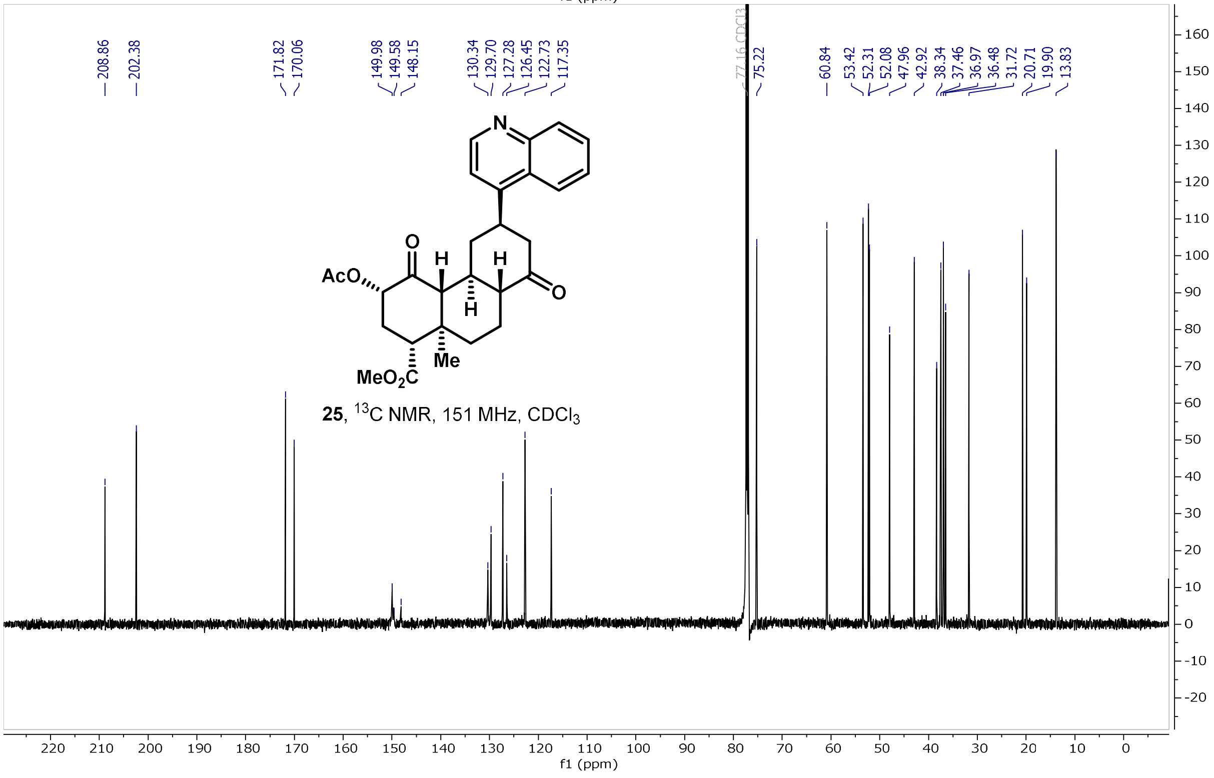

25, ^{13}C NMR, 151 MHz, $CDCl_3$

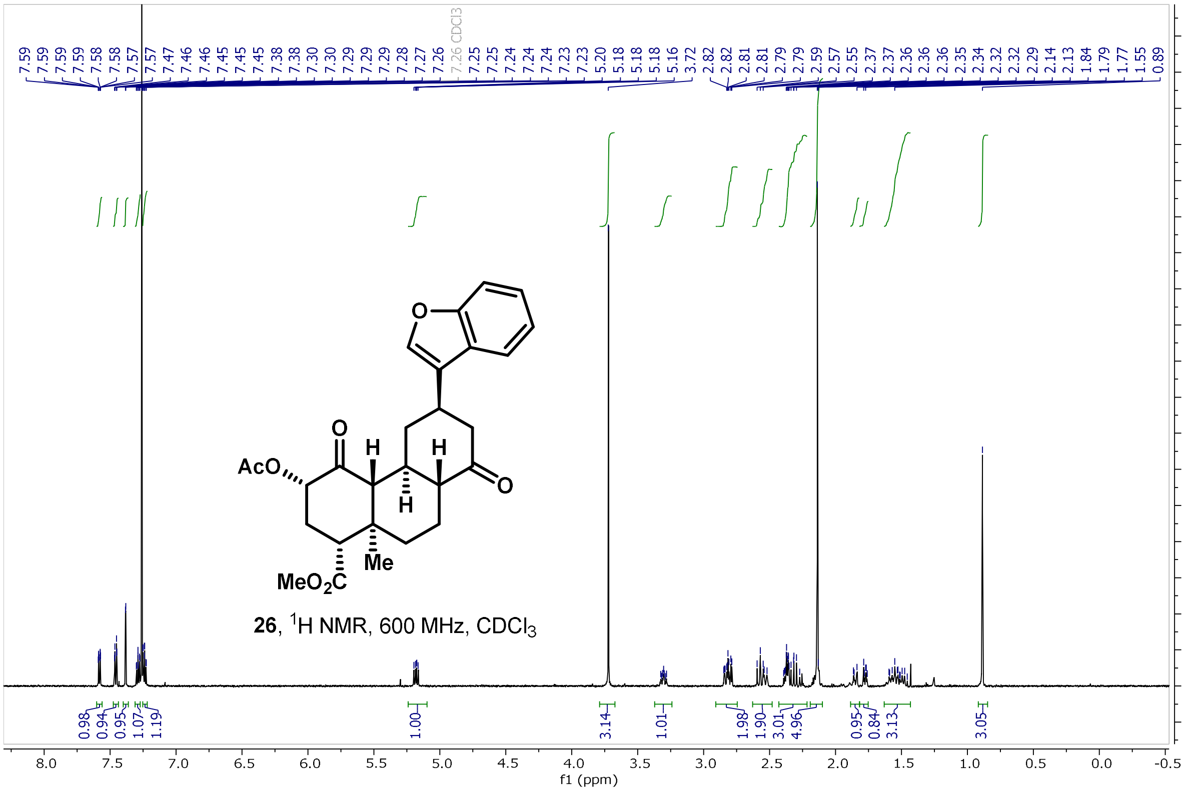

26, ¹H NMR, 600 MHz, CDCl3
AcO
MeO2C
Me
f1 (ppm)

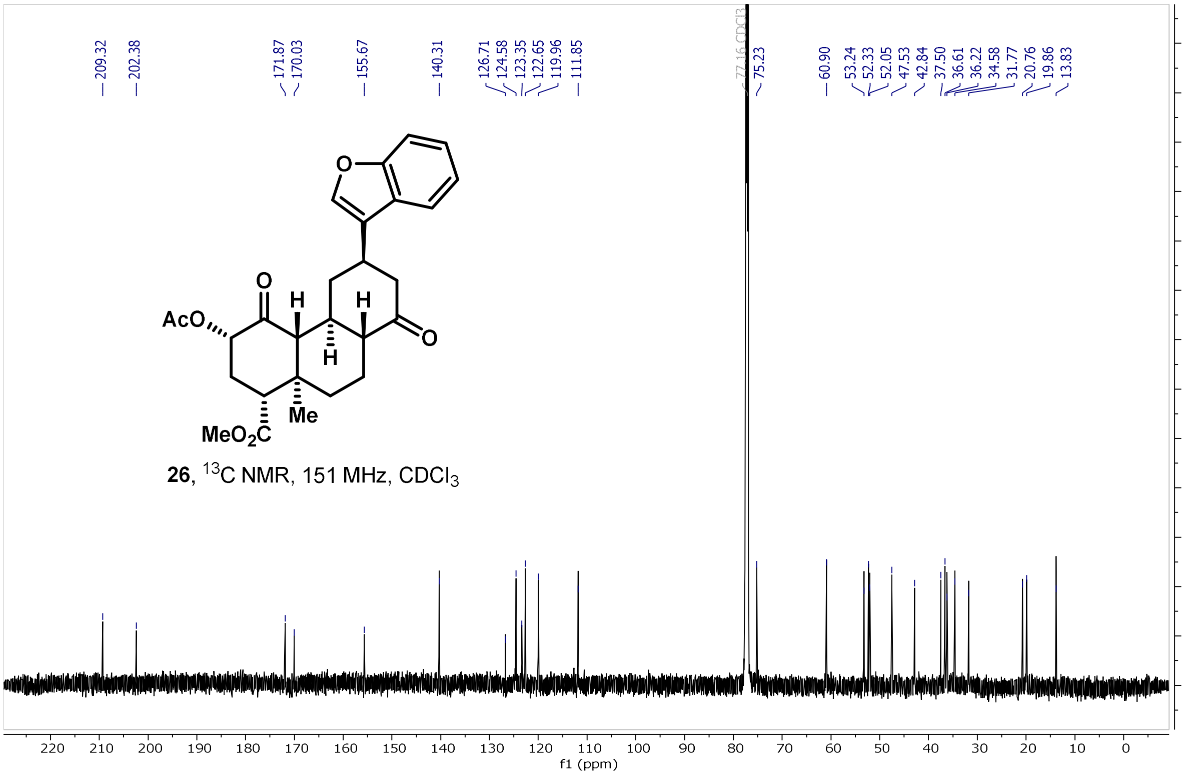

26, ¹³C NMR, 151 MHz, CDCl3
209.32
202.38
171.87
170.03
155.67
140.31
126.71
124.58
123.35
122.65
119.96
111.85
77.16 CDCl3
75.23
60.90
53.24
52.33
52.05
47.53
42.84
37.50
36.61
36.22
34.58
31.77
20.76
19.86
13.83
f1 (ppm)

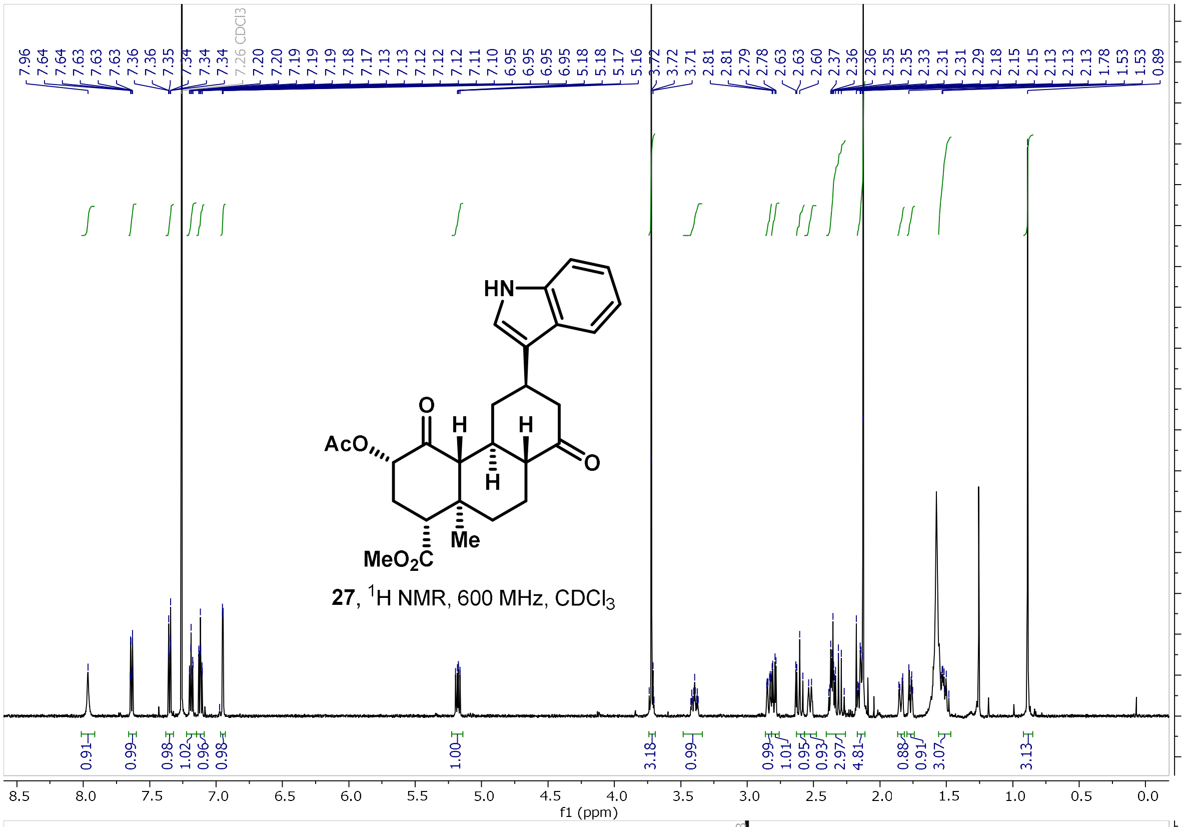
7.96 7.64 7.63 7.63 7.63 7.36 7.36 7.35 7.34 7.34 7.34 7.26 CDCl3 7.20 7.20 7.19 7.19 7.19 7.18 7.17 7.13 7.13 7.12 7.12 7.12 7.11 7.10 6.95 6.95 6.95 6.95 5.18 5.18 5.17 5.16 3.72 3.72 3.71 2.81 2.81 2.79 2.78 2.63 2.63 2.60 2.37 2.36 2.36 2.35 2.35 2.33 2.31 2.31 2.29 2.18 2.15 2.15 2.13 2.13 2.13 1.78 1.53 1.53 0.89
HN
AcO
O
H
H
H
O
Me
MeO2C
27, 1H NMR, 600 MHz, CDCl3
0.91 0.99 0.98 1.02 0.96 0.98 1.00 3.18 0.99 0.99 1.01 0.95 0.93 2.97 4.81 0.88 0.91 3.07 3.13
f1 (ppm)

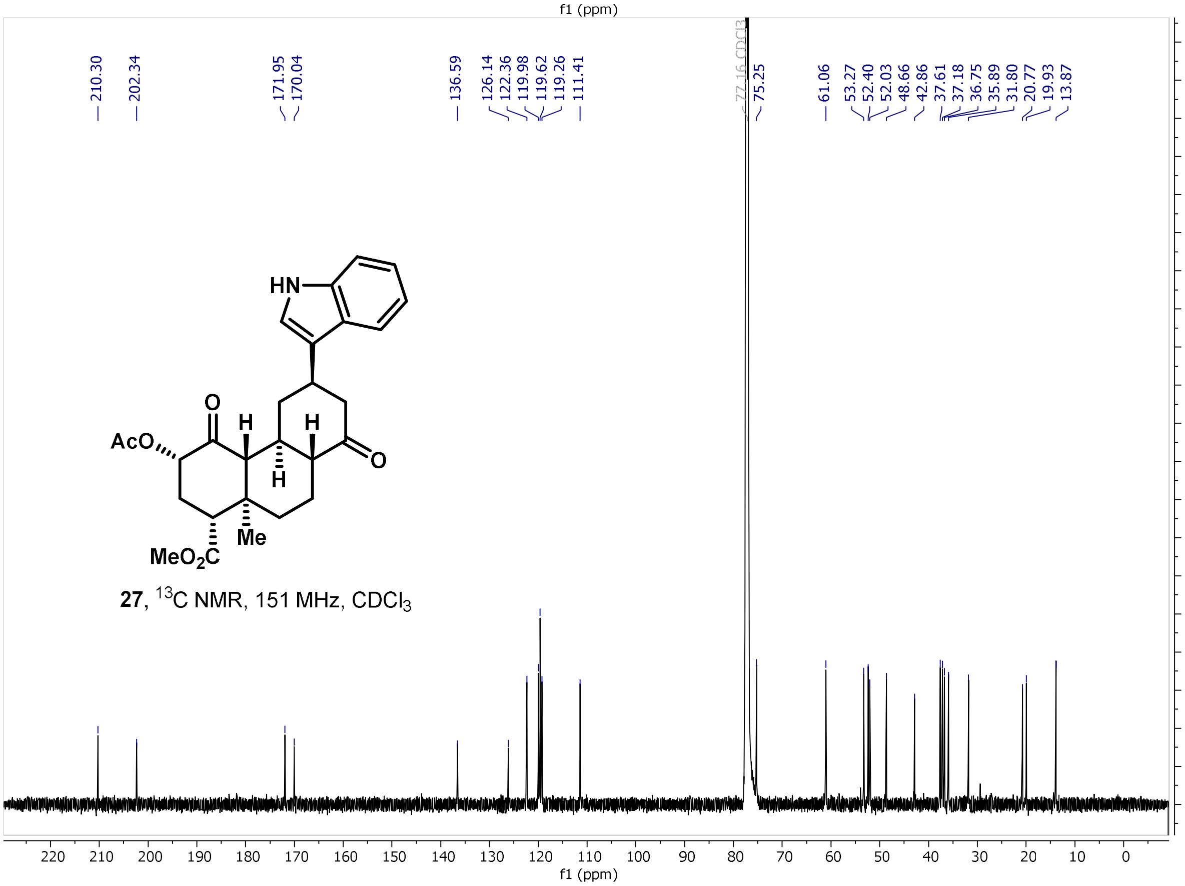
210.30 202.34 171.95 170.04 136.59 126.14 122.36 119.98 119.62 119.26 111.41 77.16 CDCl3 75.25 61.06 53.27 52.40 52.03 48.66 42.86 37.61 37.18 36.75 35.89 31.80 20.77 19.93 13.87
HN
AcO
O
H
H
H
O
Me
MeO2C
27, 13C NMR, 151 MHz, CDCl3
f1 (ppm)

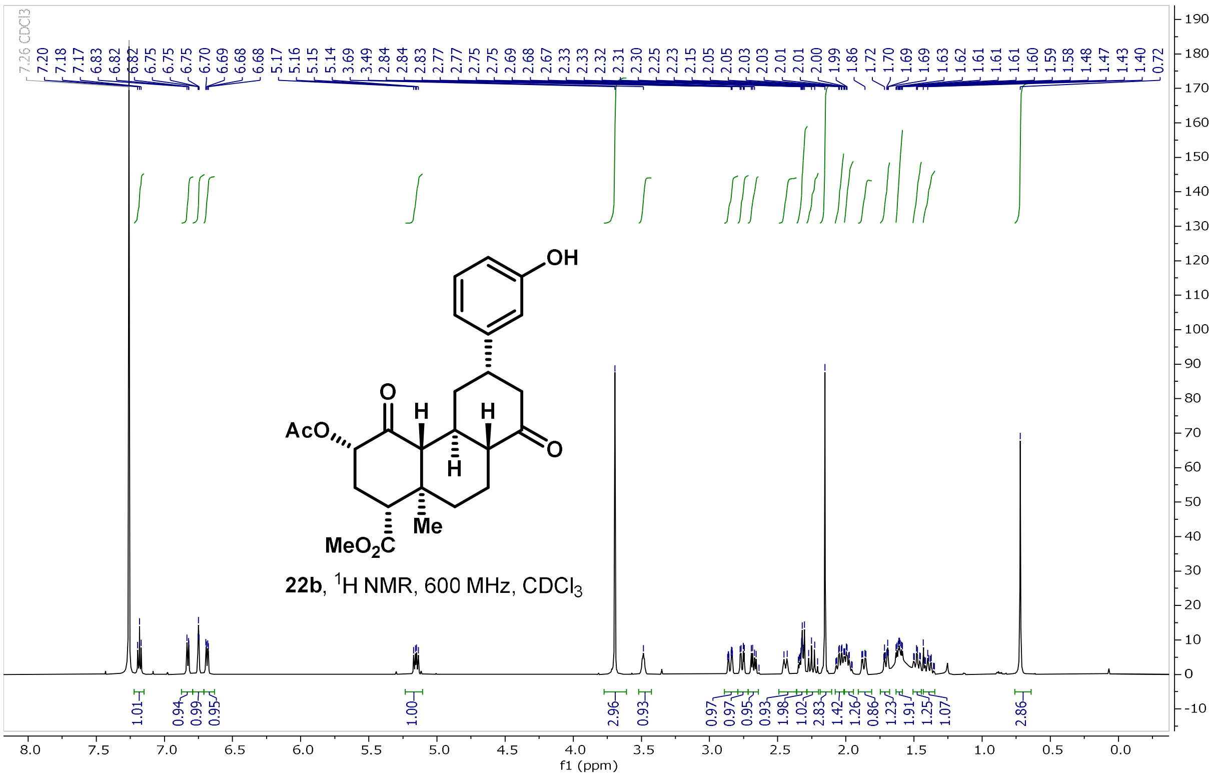

22b, ^{1}H NMR, 600 MHz, $CDCl_3$

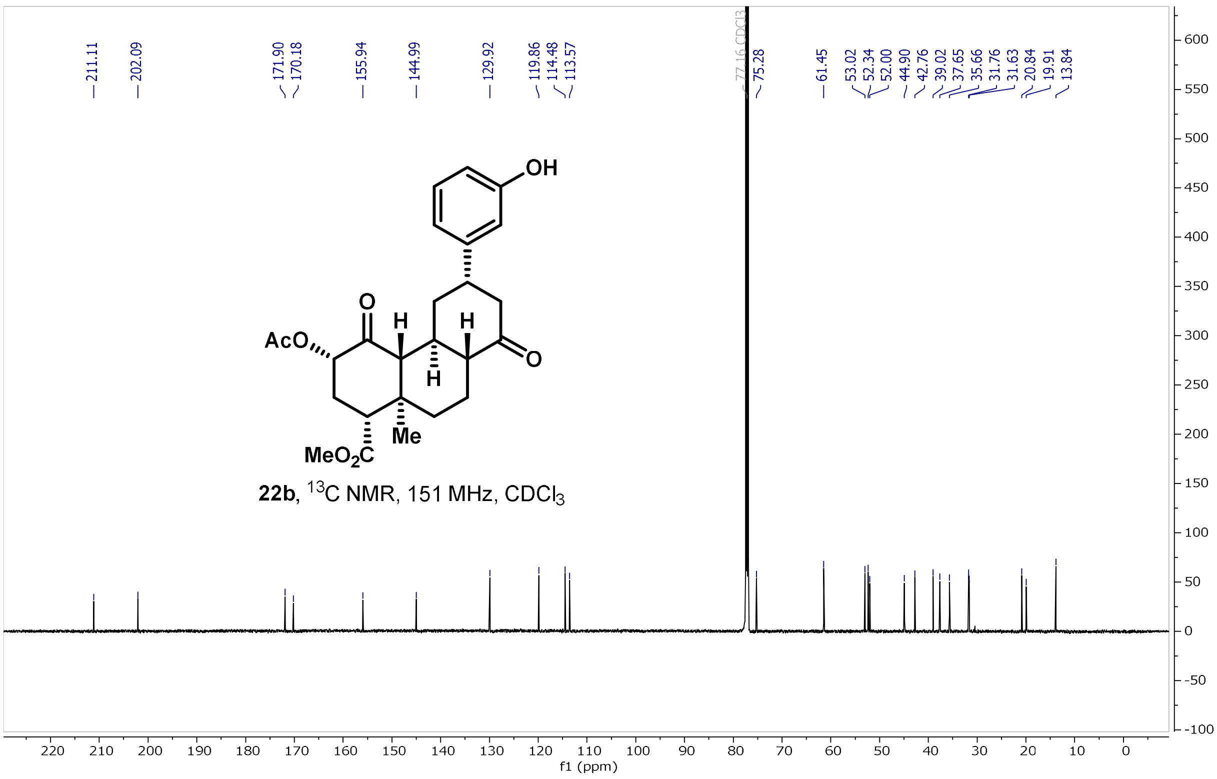

22b, ^{13}C NMR, 151 MHz, $CDCl_3$

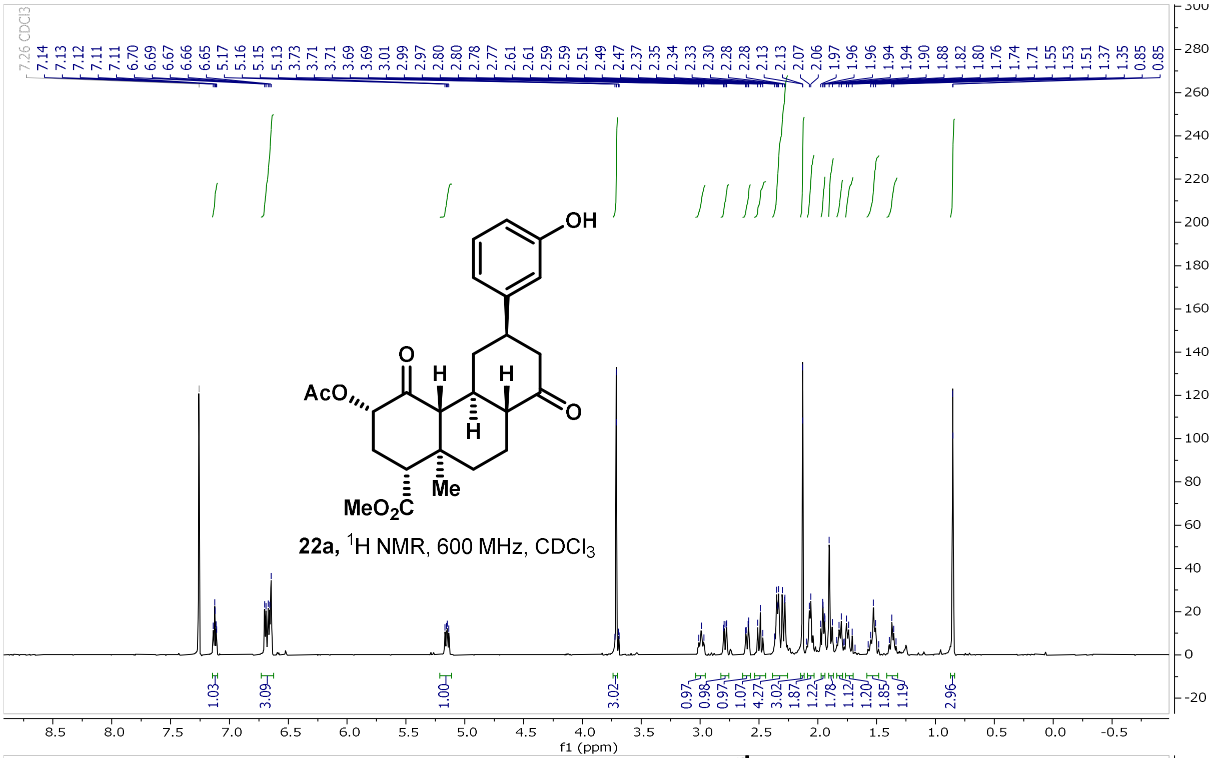
22a, ¹H NMR, 600 MHz, CDCl₃
f1 (ppm)

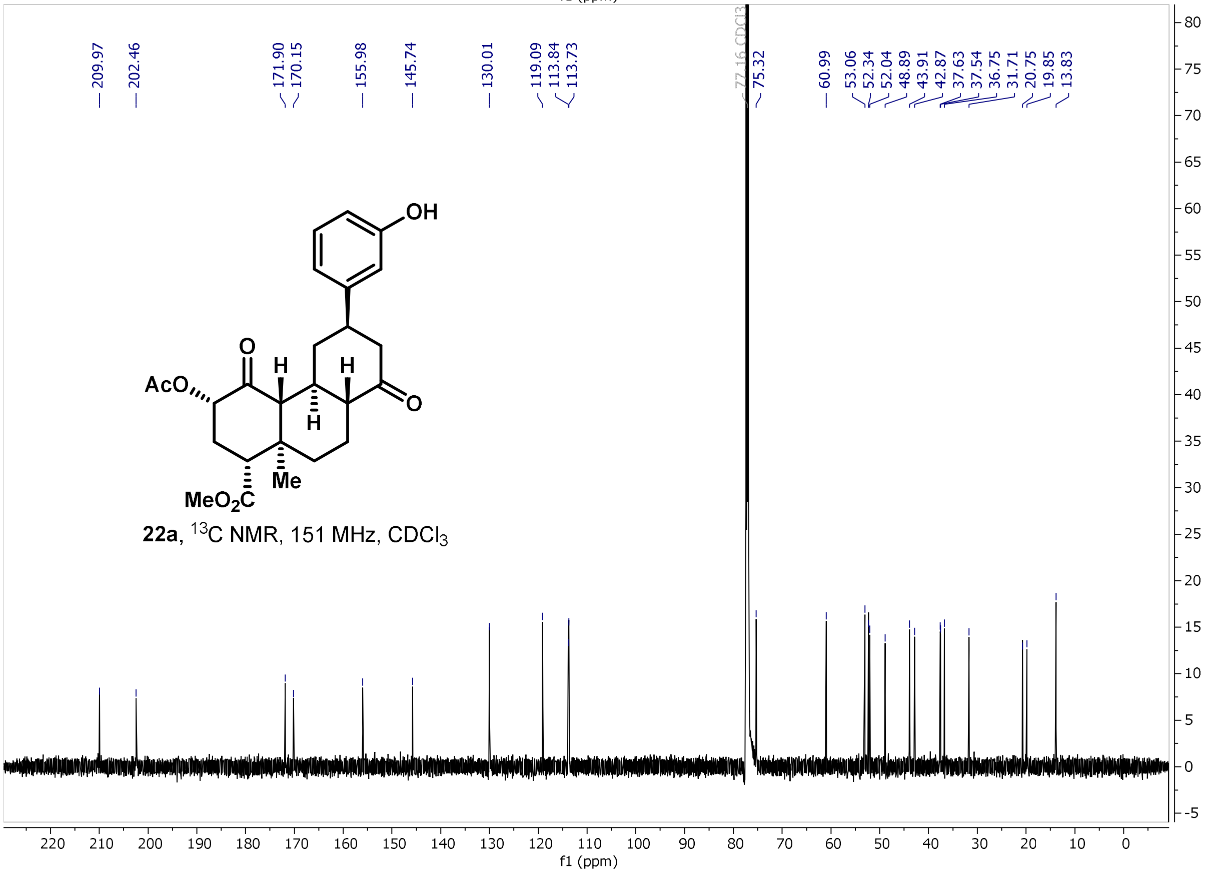
22a, ¹³C NMR, 151 MHz, CDCl₃
f1 (ppm)

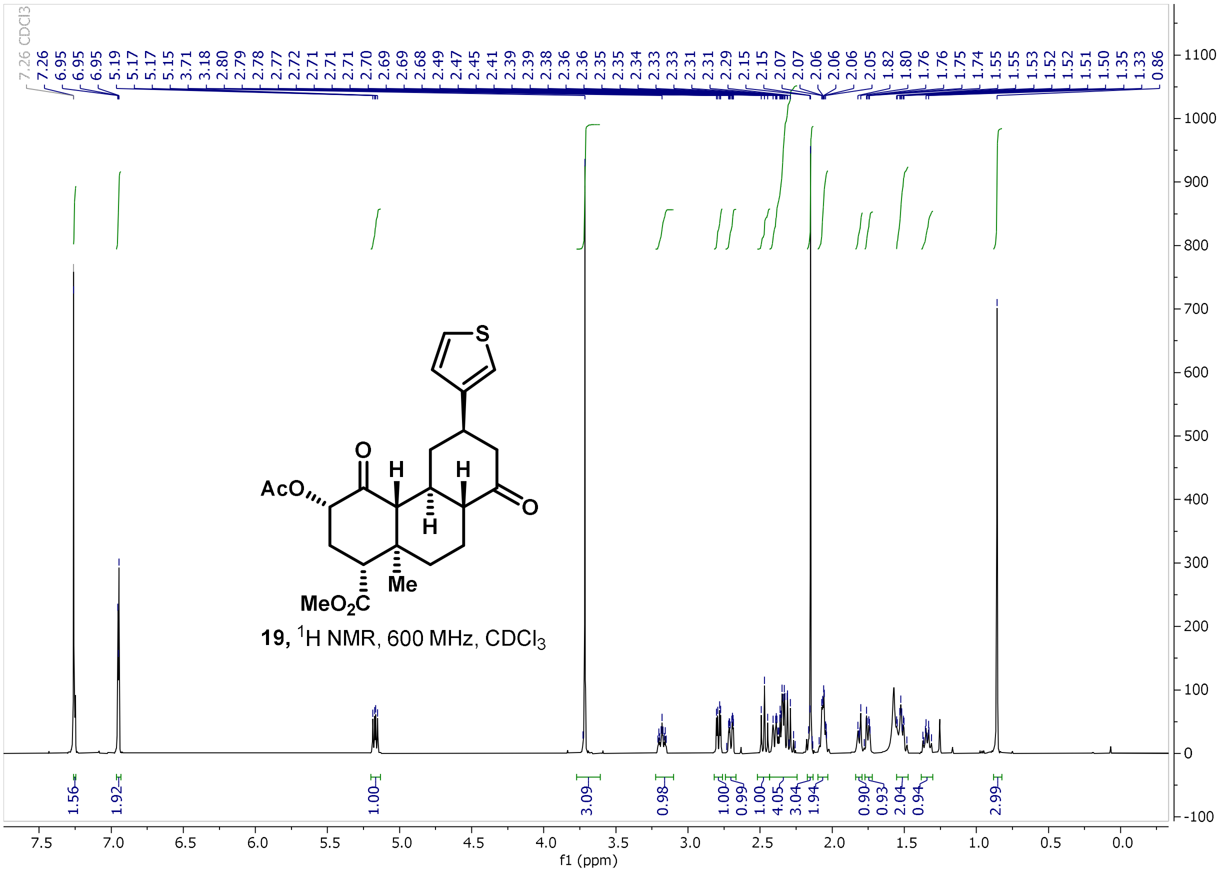
19, ^{1}H NMR, 600 MHz, $CDCl_3$
f1 (ppm)

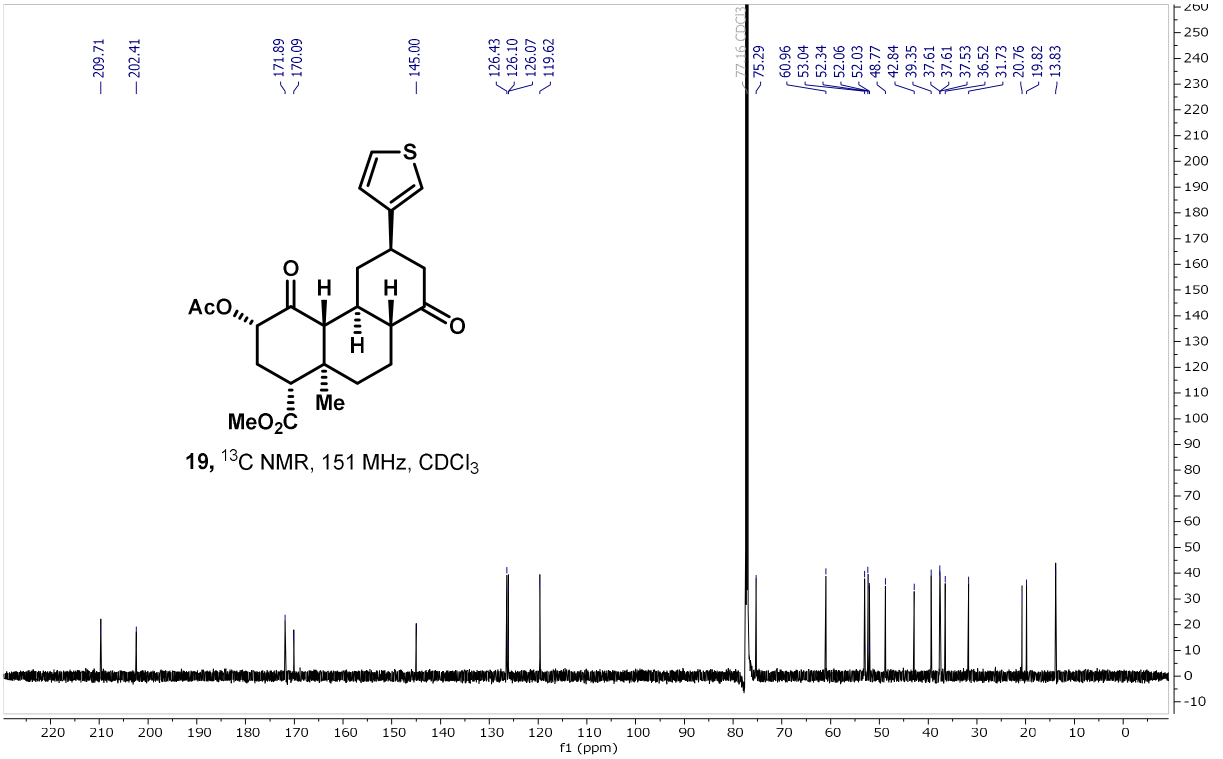
19, ^{13}C NMR, 151 MHz, $CDCl_3$
f1 (ppm)

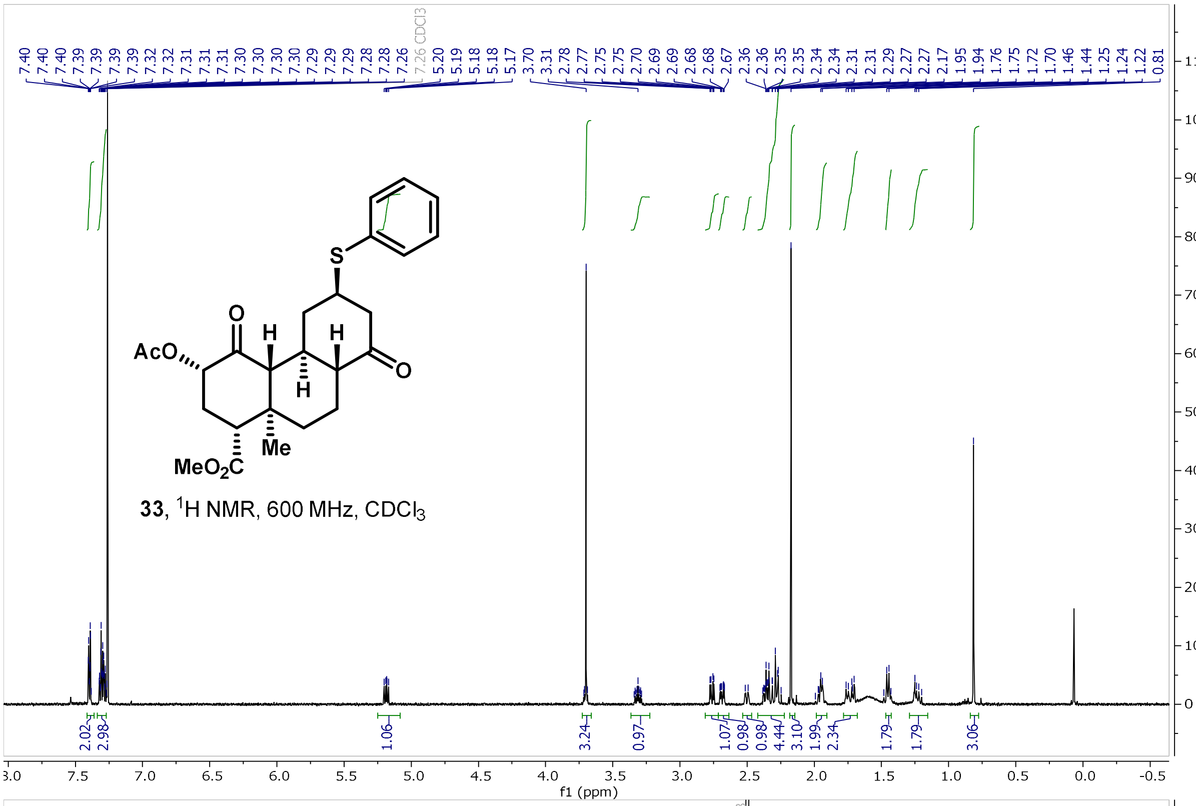
33, 1H NMR, 600 MHz, CDCl3
AcO
MeO2C
Me
H
S
O
7.40 7.40 7.40 7.39 7.39 7.39 7.39 7.32 7.32 7.31 7.31 7.31 7.30 7.30 7.30 7.30 7.29 7.29 7.29 7.28 7.28 7.26 7.26 CDCl3 5.20 5.19 5.18 5.18 5.17 3.70 3.31 2.78 2.77 2.75 2.75 2.70 2.69 2.69 2.68 2.68 2.67 2.36 2.36 2.35 2.35 2.34 2.34 2.31 2.31 2.29 2.27 2.27 2.17 2.17 1.95 1.94 1.76 1.75 1.72 1.70 1.46 1.44 1.25 1.24 1.22 0.81
2.02 2.98 1.06 3.24 0.97 1.07 0.98 0.98 4.44 3.10 1.99 2.34 1.79 1.79 3.06
f1 (ppm)

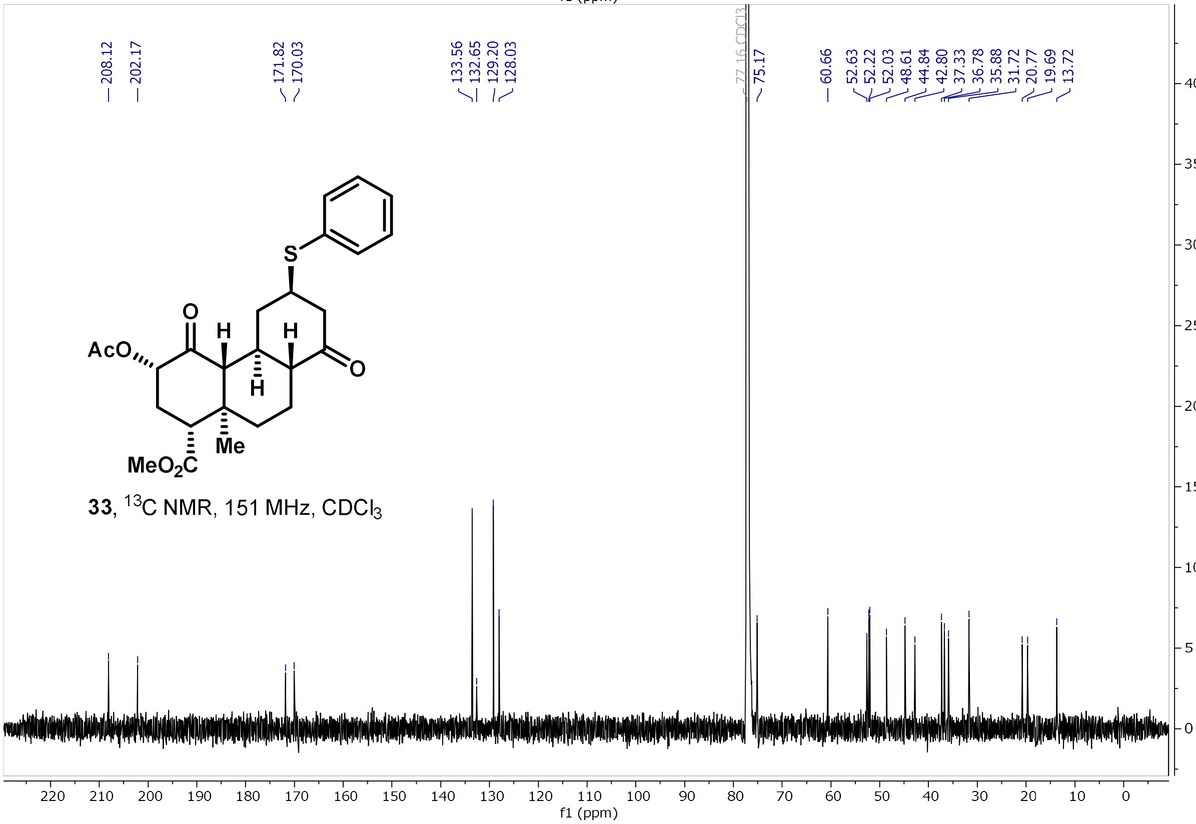
33, 13C NMR, 151 MHz, CDCl3
AcO
MeO2C
Me
H
S
O
208.12 202.17 171.82 170.03 133.56 132.65 129.20 128.03 77.16 CDCl3 75.17 60.66 52.63 52.22 52.03 48.61 44.84 42.80 37.33 36.78 35.88 31.72 20.77 19.69 13.72
f1 (ppm)

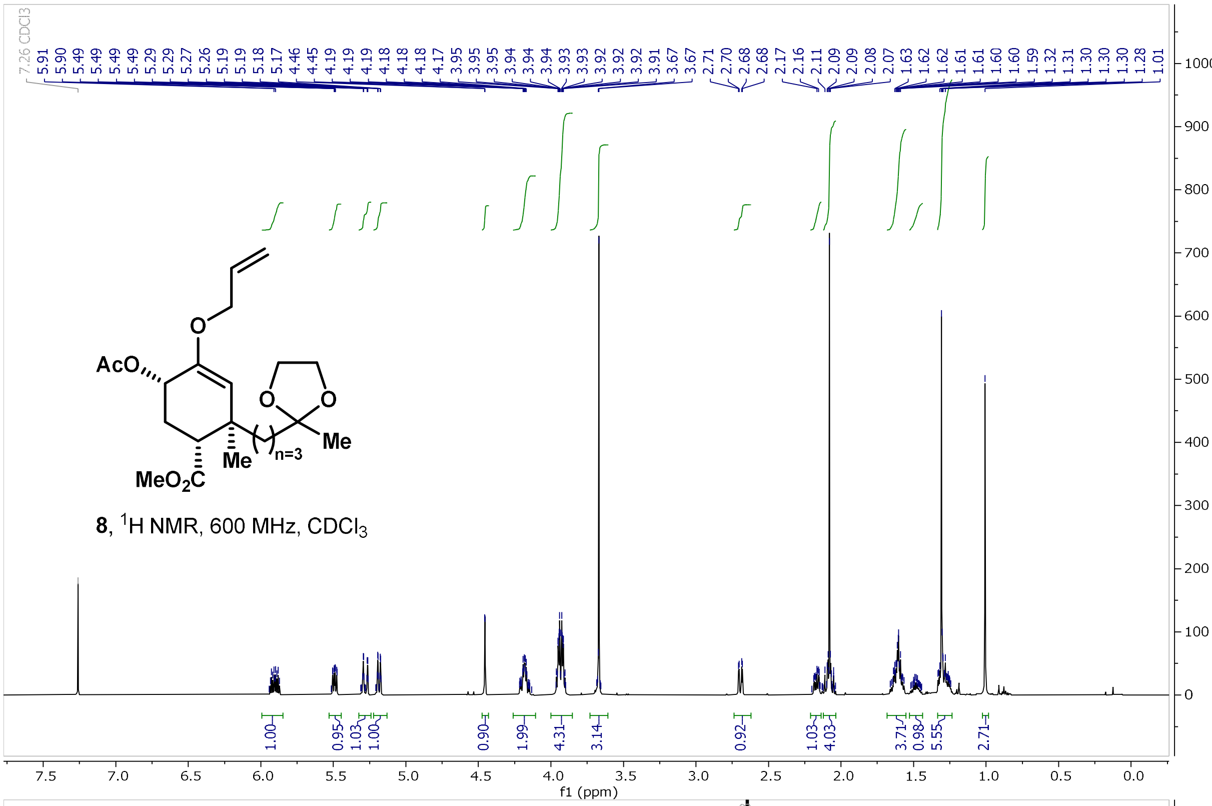
AcO
O
O
O
Me
Me
MeO2C
n=3
8, 1H NMR, 600 MHz, CDCl3
f1 (ppm)

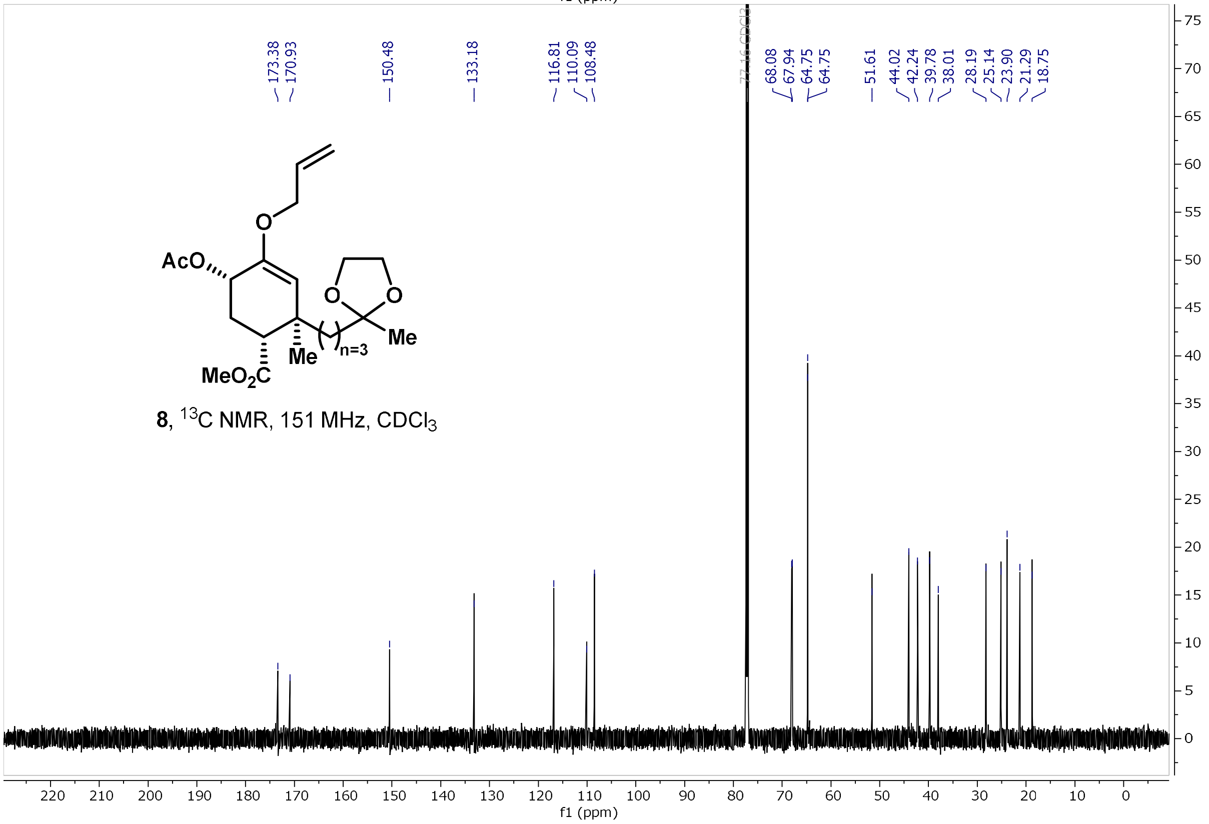
173.38
170.93
150.48
133.18
116.81
110.09
108.48
68.08
67.94
64.75
64.75
51.61
44.02
42.24
39.78
38.01
28.19
25.14
23.90
21.29
18.75
AcO
Me
Me
MeO2C
n=3
8, 13C NMR, 151 MHz, CDCl3
f1 (ppm)

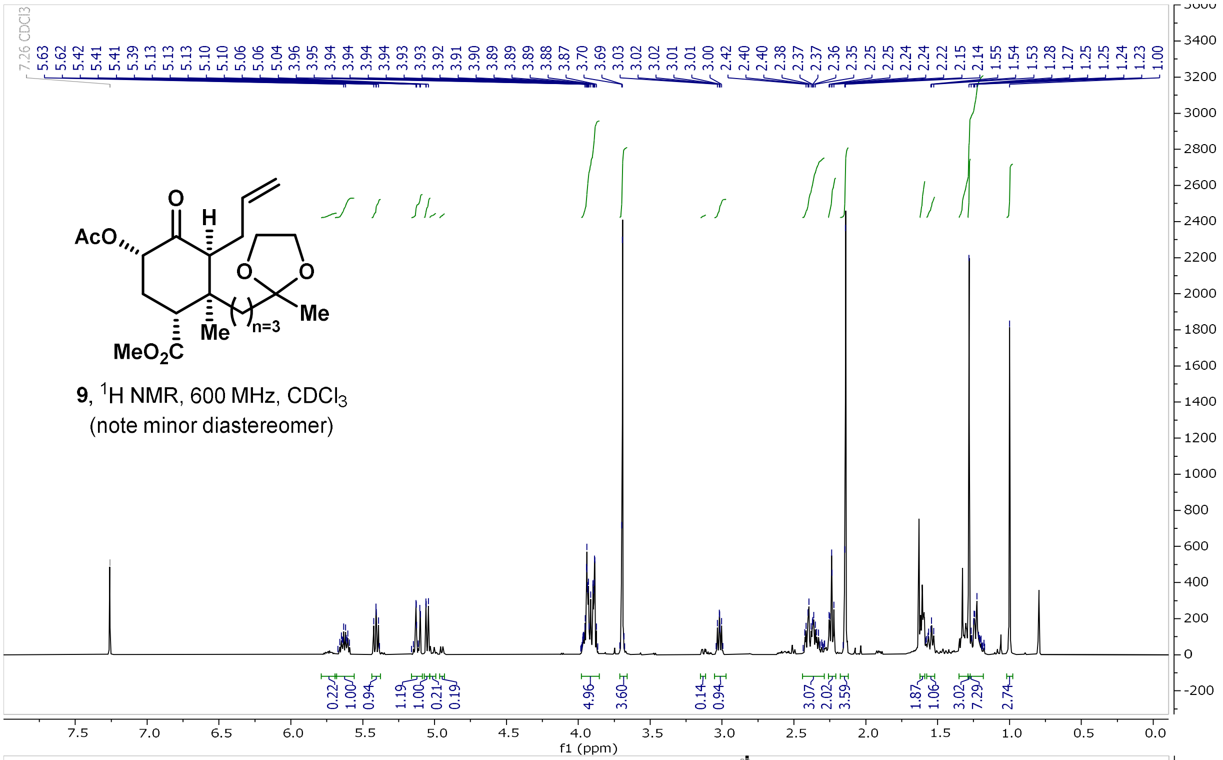

AcO
O
H
O
O
Me
Me
n=3
MeO2C
9, 1H NMR, 600 MHz, CDCl3
(note minor diastereomer)
f1 (ppm)

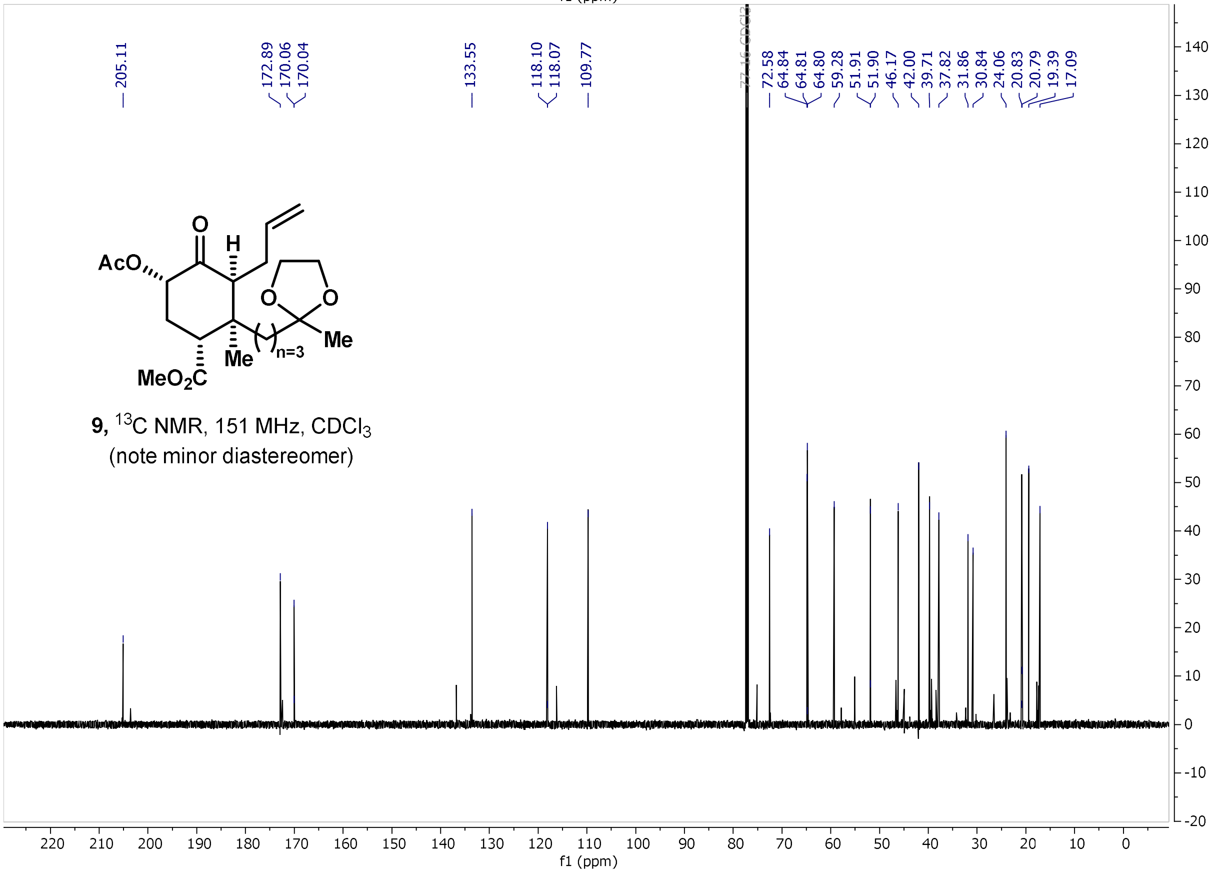

205.11
172.89
170.06
170.04
133.55
118.10
118.07
109.77
72.58
64.84
64.81
64.80
59.28
51.91
51.90
46.17
42.00
39.71
37.82
31.86
30.84
24.06
20.83
20.79
19.39
17.09
AcO
O
H
O
O
Me
Me
n=3
MeO2C
9, 13C NMR, 151 MHz, CDCl3
(note minor diastereomer)
f1 (ppm)

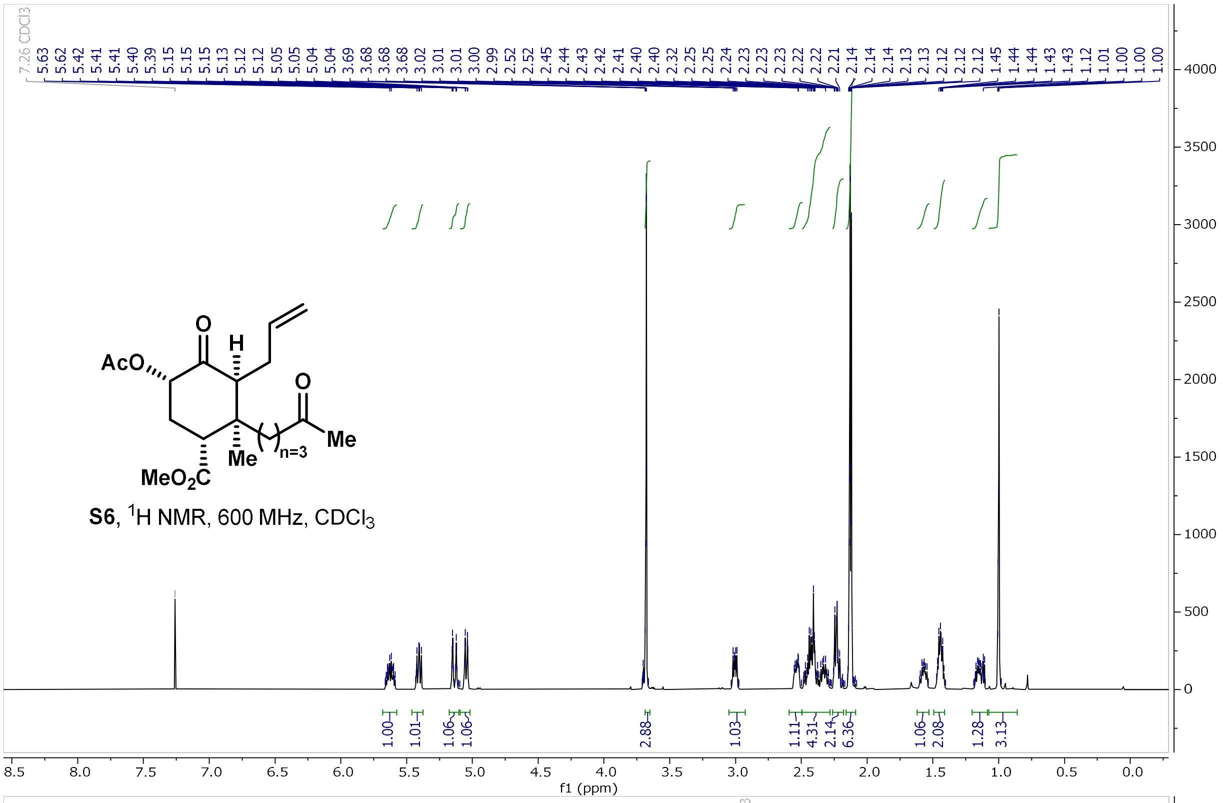
S6, ^{1}H NMR, 600 MHz, $CDCl_3$
f1 (ppm)

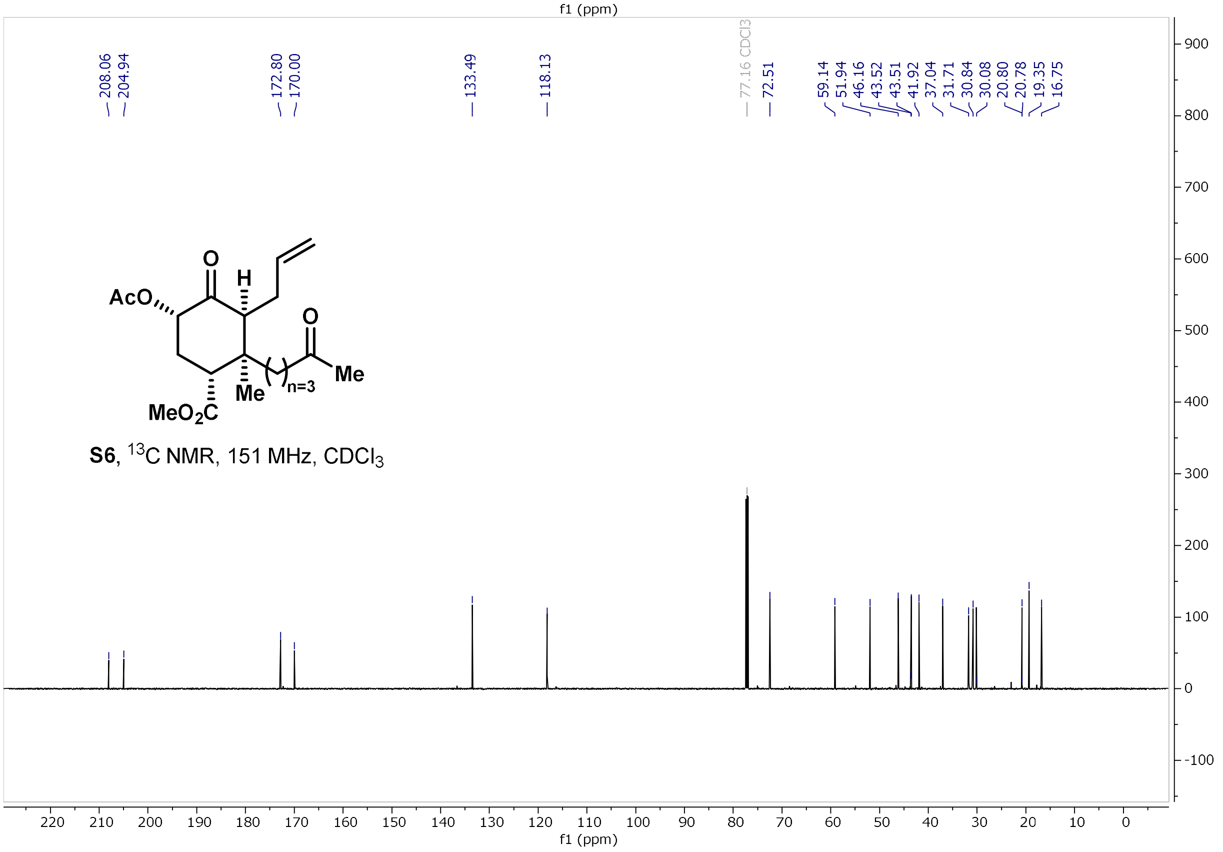
S6, ^{13}C NMR, 151 MHz, $CDCl_3$
f1 (ppm)

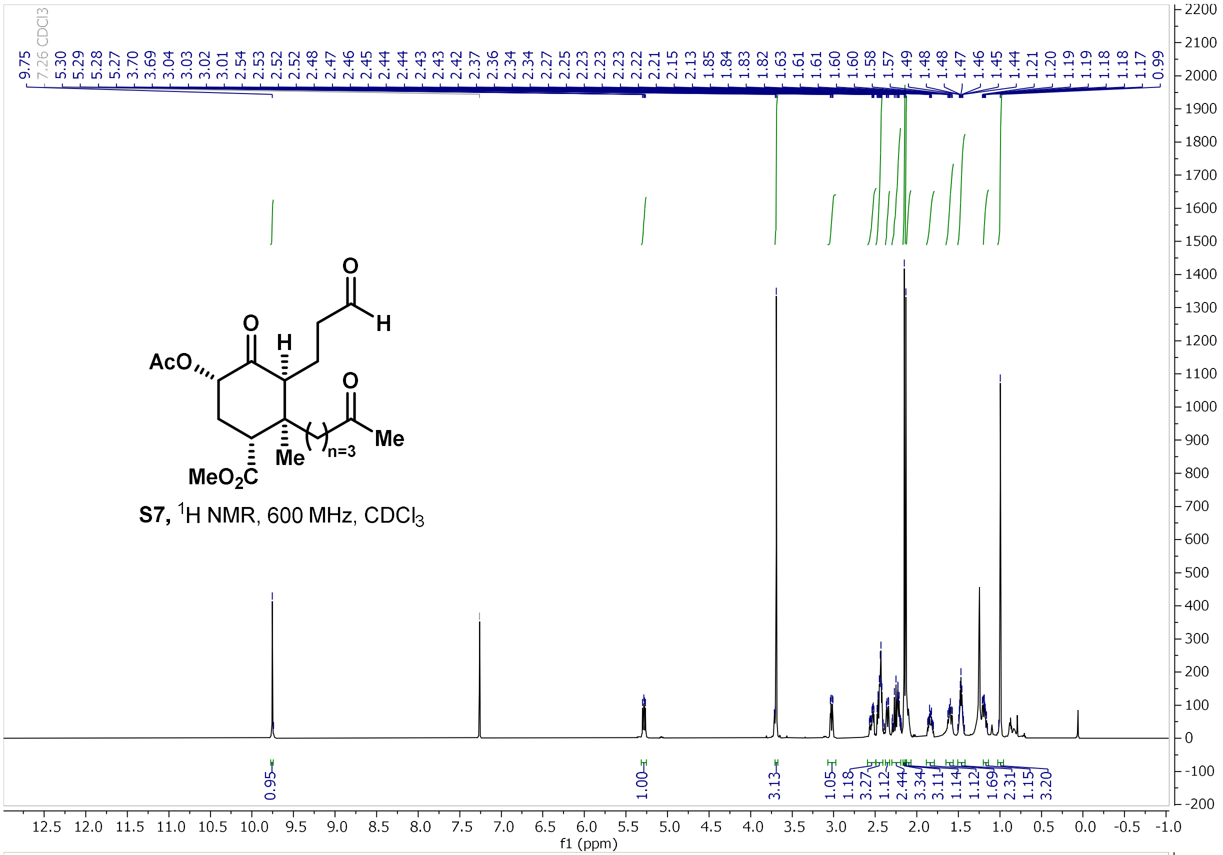

S7, 1H NMR, 600 MHz, $CDCl_3$

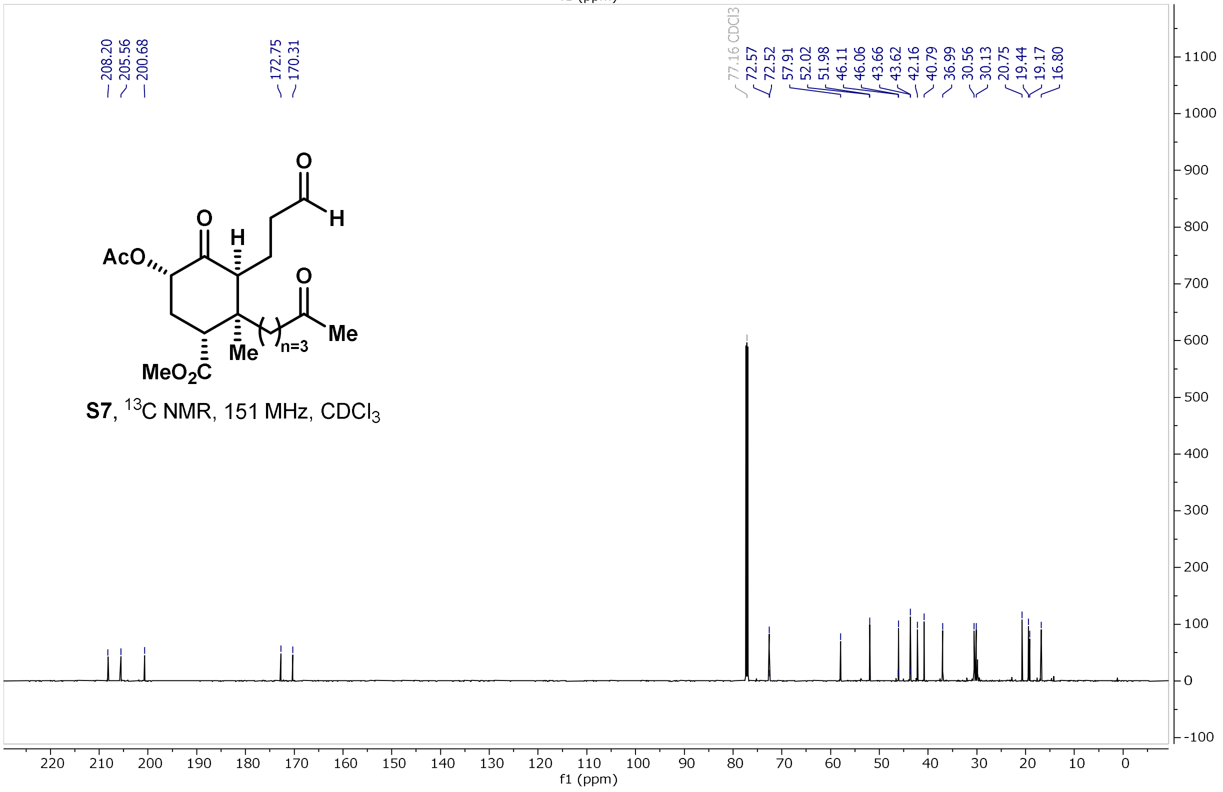

S7, 13C NMR, 151 MHz, $CDCl_3$

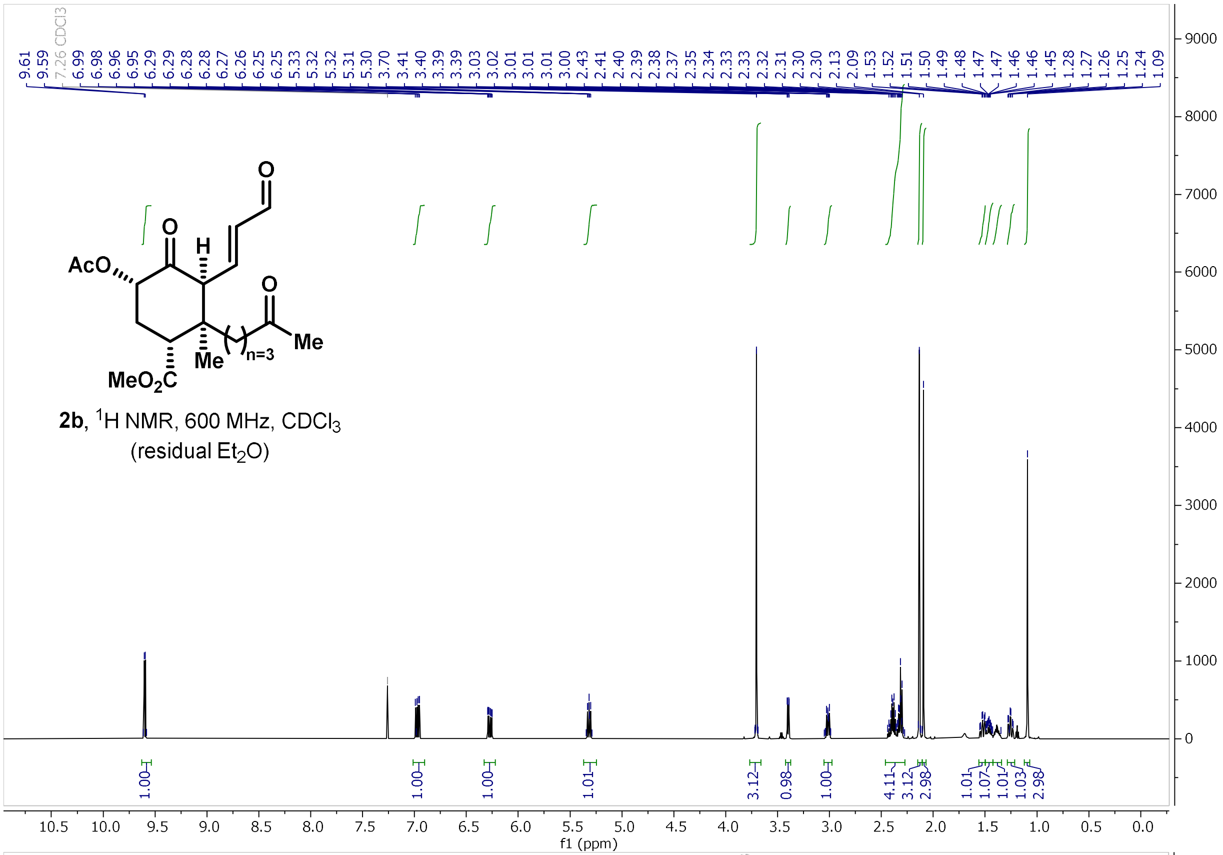
9.61
9.59
7.26 CDCl3
6.99
6.98
6.96
6.95
6.29
6.29
6.28
6.28
6.27
6.26
6.25
6.25
5.33
5.32
5.32
5.31
5.30
3.70
3.41
3.40
3.39
3.39
3.03
3.02
3.01
3.01
3.01
3.00
2.43
2.41
2.40
2.39
2.38
2.37
2.35
2.34
2.33
2.33
2.32
2.31
2.30
2.30
2.13
2.09
1.53
1.52
1.51
1.50
1.49
1.48
1.47
1.47
1.46
1.46
1.45
1.28
1.27
1.26
1.25
1.24
1.09
AcO
O
H
O
O
Me
Me
n=3
MeO2C
2b, 1H NMR, 600 MHz, CDCl3
(residual Et2O)
1.00
1.00
1.00
1.01
3.12
0.98
1.00
4.11
3.12
2.98
1.01
1.07
1.01
1.03
2.98
f1 (ppm)

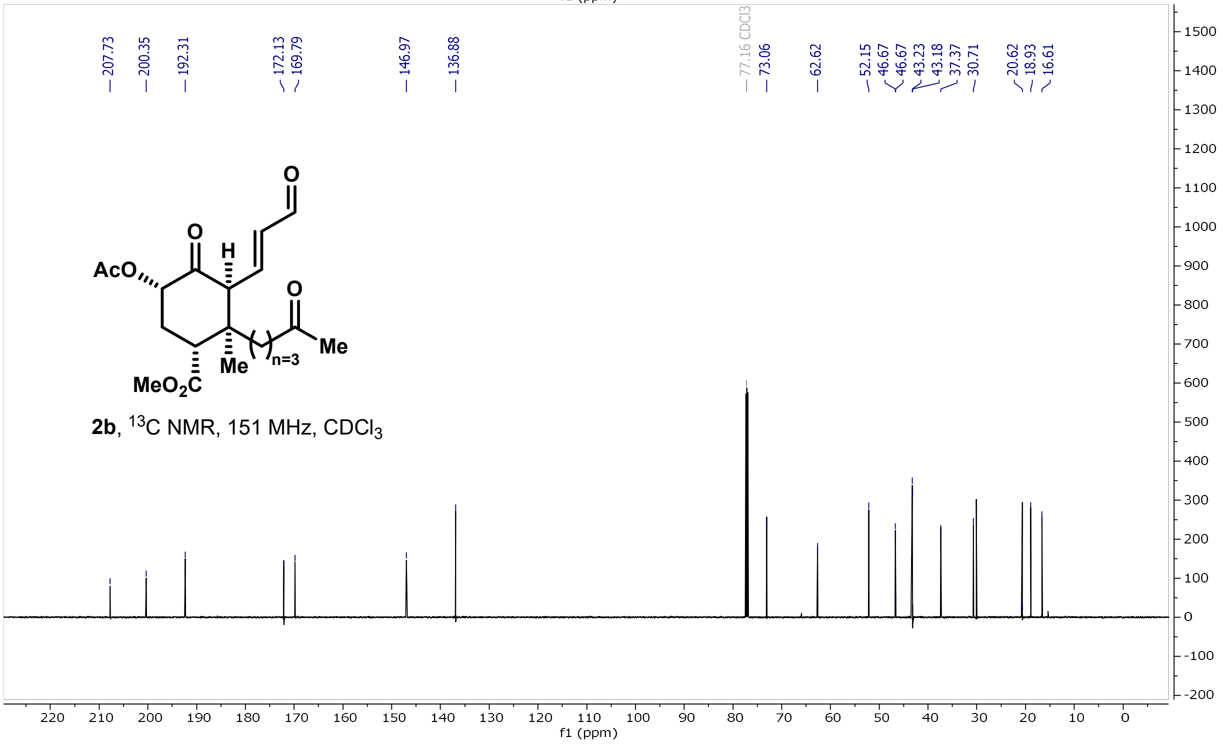
207.73
200.35
192.31
172.13
169.79
146.97
136.88
77.16 CDCl3
73.06
62.62
52.15
46.67
46.67
43.23
43.18
37.37
30.71
20.62
18.93
16.61
AcO
O
H
O
O
Me
Me
n=3
MeO2C
2b, 13C NMR, 151 MHz, CDCl3
f1 (ppm)

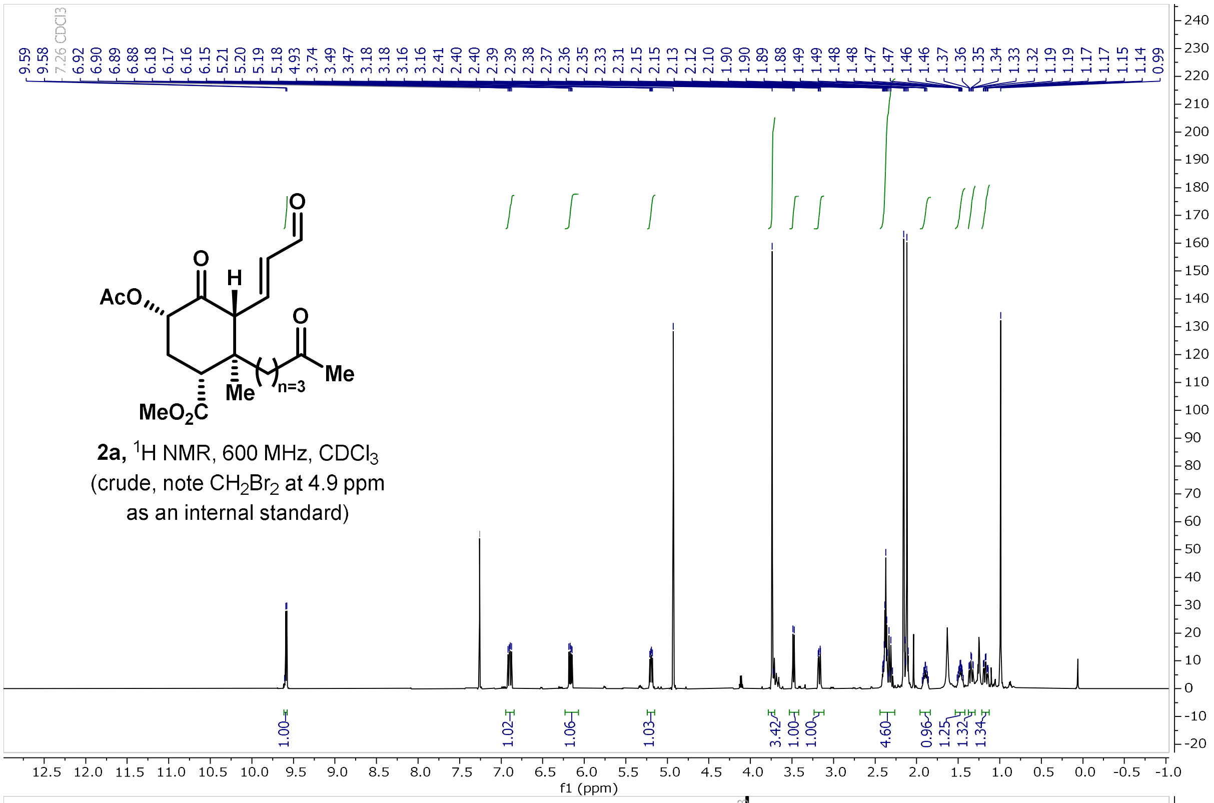
9.59
9.58
7.26 CDCl3
6.92
6.90
6.89
6.88
6.18
6.17
6.16
6.15
5.21
5.20
5.19
5.18
4.93
3.74
3.49
3.47
3.18
3.18
3.16
3.16
2.41
2.40
2.40
2.39
2.38
2.37
2.36
2.35
2.33
2.31
2.15
2.15
2.13
2.12
2.10
1.90
1.90
1.89
1.88
1.49
1.49
1.48
1.47
1.47
1.46
1.46
1.37
1.36
1.35
1.34
1.33
1.32
1.19
1.19
1.17
1.17
1.15
1.14
0.99
AcO
O
H
O
O
Me
Me
n=3
MeO2C
2a, 1H NMR, 600 MHz, CDCl3
(crude, note CH2Br2 at 4.9 ppm
as an internal standard)
1.00
1.02
1.06
1.03
3.42
1.00
1.00
4.60
0.96
1.25
1.32
1.34
f1 (ppm)

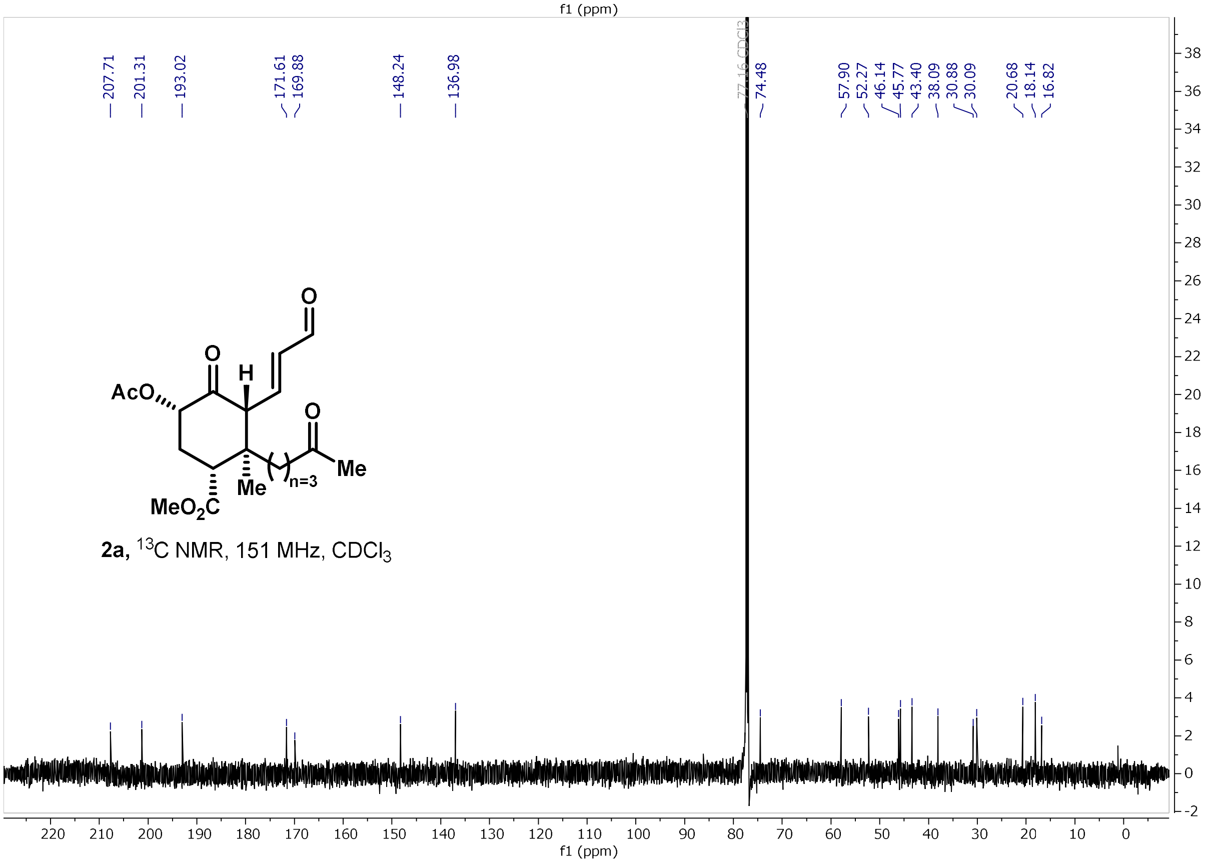
207.71
201.31
193.02
171.61
169.88
148.24
136.98
77.16 CDCl3
74.48
57.90
52.27
46.14
45.77
43.40
38.09
30.88
30.09
20.68
18.14
16.82
AcO
O
H
O
O
Me
Me
n=3
MeO2C
2a, 13C NMR, 151 MHz, CDCl3
f1 (ppm)

X-ray crystallographic data

All X-ray structures were of enantiopure material which confirmed absolute stereochemistry.

**Compound**	**Deposition Number**
Hydroxy Hagemann's Ester **3**	2202073
Acetate **6**	2195935
Iodide **11**	2211512
Ferrocene Carboxylate **S5**	2241886
Et_2Zn Reformatsky Adduct **13**	2245372
Enone **17d**	2250953
Enone **17b**	2217612
'O6C' **1**	2249326
Analog **24a**	2263493
Analog **24b**	2268905
Analog **32**	2267254
Deketalized C-allyl **S6**	2127086
Enal **2b**	2166577

X-ray Structure of Hydroxyl **3** (CCDC2202073)

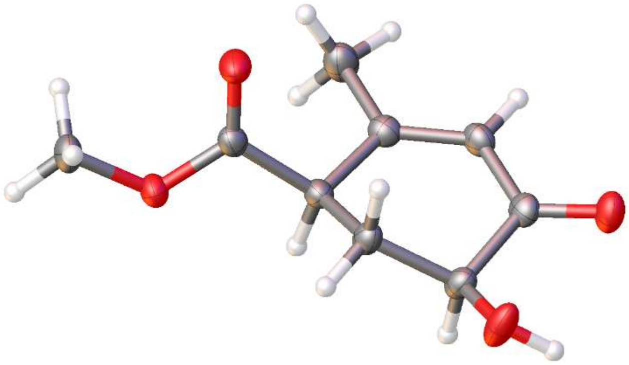

Experimental Summary

The single crystal X-ray diffraction studies were carried out on a Bruker Smart APEX II CCD diffractometer equipped with Cu K_α radiation (λ = 1.54178 Å).

Crystals of the subject compound were used as received. A 0.2 x 0.12 x 0.08 mm piece of a crystal was mounted on a Cryoloop with Paratone oil. Data were collected in a nitrogen gas stream at 100(2) K using ϕ and ω scans. Crystal-to-detector distance was 40 mm and exposure time was 1, 1.5, 2, 2.5, or 3 seconds depending on the 2θ range per frame using a scan width of 1.5°. Data collection was 99.9 % complete to 67.679° in θ. A total of 6594 reflections were collected covering the indices, −6<=h<=6, −8<=k<=8, −13<=l<=13. 1679 reflections were found to be symmetry independent, with a R_{int} of 0.0230. Indexing and unit cell refinement indicated a **P**rimitive, **Monoclinic** lattice. The space group was found to be ***P*2₁**. The data were integrated using the Bruker SAINT Software program and scaled using the SADABS software program. Solution by direct methods (SHELXT) produced a complete phasing model consistent with the proposed structure.

All nonhydrogen atoms were refined anisotropically by full-matrix least-squares (SHELXL-2014). All carbon bonded hydrogen atoms were placed using a riding model. Their positions were constrained relative to their parent atom using the appropriate HFIX command in SHELXL-2014. Crystallographic data are summarized in Table S9.1.

Notes: Absolute stereochemistry was conclusively assigned (Absolute Structure Parameter = 0.04(4)). There is one copy of the compound in the asymmetric unit. The chemical formula for the compound is: $C_9H_{12}O_4$

Table S9.1. Crystal data and structure refinement for Shenvi207.

Identification code	shenvi207
Empirical formula	C9 H12 O4
Formula weight	184.19
Temperature	100.0 K
Wavelength	1.54178 Å
Crystal system	Monoclinic
Space group	P 1 21 1
Unit cell dimensions	a = 5.4573(2) Å α = 90°.
	b = 7.1662(2) Å β = 90.9110(10)°.
	c = 11.4633(3) Å γ= 90°.
Volume	448.25(2) Å3
Z	2
Density (calculated)	1.365 Mg/m^3
Absorption coefficient	0.907 mm^{-1}
F(000)	196
Crystal size	0.2 x 0.12 x 0.08 mm^3
Theta range for data collection	3.856 to 70.146°.
Index ranges	-6<=h<=6, -8<=k<=8, -13<=l<=13
Reflections collected	6594
Independent reflections	1679 [R(int) = 0.0230]
Completeness to theta = 67.679°	99.9 %
Absorption correction	Semi-empirical from equivalents
Max. and min. transmission	0.5219 and 0.4314
Refinement method	Full-matrix least-squares on F^2
Data / restraints / parameters	1679 / 1 / 124
Goodness-of-fit on F^2	1.073
Final R indices [I>2sigma(I)]	R1 = 0.0251, wR2 = 0.0669
R indices (all data)	R1 = 0.0253, wR2 = 0.0670
Absolute structure parameter	0.04(4)
Largest diff. peak and hole	0.178 and -0.115 e.Å^{-3}

Table S9.2. Atomic coordinates (x 10^4) and equivalent isotropic displacement parameters (Å^{2}x 10^3) for Shenvi207. U(eq) is defined as one third of the trace of the orthogonalized U^{ij} tensor.

	x	y	z	U(eq)
O(1)	9834(2)	4688(2)	382(1)	32(1)
O(2)	8737(3)	1560(2)	1640(1)	36(1)
O(3)	4735(2)	6374(2)	4592(1)	32(1)
O(4)	1359(2)	4662(2)	4247(1)	26(1)
C(1)	3549(4)	8428(3)	2166(2)	32(1)
C(2)	4922(3)	6643(3)	2005(1)	24(1)
C(3)	6747(3)	6509(3)	1238(1)	25(1)
C(4)	8033(3)	4764(3)	1006(1)	24(1)
C(5)	6999(3)	3002(3)	1544(1)	25(1)
C(6)	6049(3)	3429(3)	2754(1)	24(1)
C(7)	4094(3)	4964(2)	2695(1)	22(1)
C(8)	3465(3)	5452(2)	3945(2)	22(1)
C(9)	674(3)	4843(3)	5456(2)	31(1)

Table S9.3. Bond lengths [Å] and angles [°] for Shenvi207.

O(1)-C(4)	1.226(2)
O(2)-H(2)	0.87(3)
O(2)-C(5)	1.406(2)
O(3)-C(8)	1.204(2)
O(4)-C(8)	1.332(2)
O(4)-C(9)	1.4466(19)
C(1)-H(1A)	0.9800
C(1)-H(1B)	0.9800
C(1)-H(1C)	0.9800
C(1)-C(2)	1.495(3)
C(2)-C(3)	1.342(2)
C(2)-C(7)	1.513(2)
C(3)-H(3)	0.9500
C(3)-C(4)	1.461(3)
C(4)-C(5)	1.518(2)
C(5)-H(5)	1.0000
C(5)-C(6)	1.519(2)
C(6)-H(6A)	0.9900
C(6)-H(6B)	0.9900
C(6)-C(7)	1.533(2)
C(7)-H(7)	1.0000
C(7)-C(8)	1.519(2)
C(9)-H(9A)	0.9800
C(9)-H(9B)	0.9800
C(9)-H(9C)	0.9800
C(5)-O(2)-H(2)	107(2)
C(8)-O(4)-C(9)	116.61(13)
H(1A)-C(1)-H(1B)	109.5
H(1A)-C(1)-H(1C)	109.5
H(1B)-C(1)-H(1C)	109.5
C(2)-C(1)-H(1A)	109.5
C(2)-C(1)-H(1B)	109.5
C(2)-C(1)-H(1C)	109.5
C(1)-C(2)-C(7)	117.49(14)
C(3)-C(2)-C(1)	121.33(17)
C(3)-C(2)-C(7)	121.11(16)
C(2)-C(3)-H(3)	118.5
C(2)-C(3)-C(4)	122.95(17)
C(4)-C(3)-H(3)	118.5
O(1)-C(4)-C(3)	122.41(16)
O(1)-C(4)-C(5)	120.49(17)
C(3)-C(4)-C(5)	117.08(13)
O(2)-C(5)-C(4)	112.83(15)
O(2)-C(5)-H(5)	108.6
O(2)-C(5)-C(6)	108.41(14)
C(4)-C(5)-H(5)	108.6
C(4)-C(5)-C(6)	109.77(15)
C(6)-C(5)-H(5)	108.6
C(5)-C(6)-H(6A)	109.5
C(5)-C(6)-H(6B)	109.5
C(5)-C(6)-C(7)	110.57(13)
H(6A)-C(6)-H(6B)	108.1
C(7)-C(6)-H(6A)	109.5
C(7)-C(6)-H(6B)	109.5
C(2)-C(7)-C(6)	112.34(14)
C(2)-C(7)-H(7)	108.3
C(2)-C(7)-C(8)	112.57(15)
C(6)-C(7)-H(7)	108.3
C(8)-C(7)-C(6)	106.93(13)
C(8)-C(7)-H(7)	108.3
O(3)-C(8)-O(4)	124.30(16)
O(3)-C(8)-C(7)	124.79(15)
O(4)-C(8)-C(7)	110.86(14)
O(4)-C(9)-H(9A)	109.5
O(4)-C(9)-H(9B)	109.5
O(4)-C(9)-H(9C)	109.5
H(9A)-C(9)-H(9B)	109.5
H(9A)-C(9)-H(9C)	109.5
H(9B)-C(9)-H(9C)	109.5

Symmetry transformations used to generate equivalent atoms:

Table S9.4. Anisotropic displacement parameters ($Å^2$x 10^3) for Shenvi207. The anisotropic displacement factor exponent takes the form: $-2\pi^2$[h2 a*2U^{11} + ... + 2 h k a* b* U^{12}]

	U11	U22	U33	U23	U13	U12
O(1)	35(1)	34(1)	27(1)	2(1)	12(1)	2(1)
O(2)	54(1)	28(1)	28(1)	2(1)	16(1)	13(1)
O(3)	29(1)	41(1)	28(1)	-10(1)	6(1)	-11(1)
O(4)	24(1)	30(1)	26(1)	-3(1)	7(1)	-6(1)

C(1)	32(1)	26(1)	38(1)	4(1)	7(1)	3(1)
C(2)	24(1)	24(1)	23(1)	1(1)	-2(1)	-1(1)
C(3)	28(1)	24(1)	23(1)	5(1)	2(1)	-4(1)
C(4)	27(1)	28(1)	17(1)	2(1)	1(1)	-1(1)
C(5)	33(1)	21(1)	21(1)	-1(1)	5(1)	1(1)
C(6)	30(1)	22(1)	20(1)	1(1)	6(1)	1(1)
C(7)	23(1)	22(1)	21(1)	-1(1)	3(1)	-4(1)
C(8)	20(1)	20(1)	26(1)	1(1)	3(1)	0(1)
C(9)	30(1)	35(1)	28(1)	-1(1)	11(1)	-4(1)

Table S9.5. Hydrogen coordinates (x 10^4) and isotropic displacement parameters ($Å^2$x 10^3) for Shenvi207.

	x	y	z	U(eq)	
H(2)	9240(50)		1330(50)		940(30)61(8)
H(1A)	1794	8206	2034	48	
H(1B)	4127	9357	1606	48	
H(1C)	3824	8889	2962	48	
H(3)	7224	7599	828	30	
H(5)	5599	2557	1044	30	
H(6A)	7425	3837	3266	29	
H(6B)	5337	2284	3094	29	
H(7)	2595	4439	2304	26	
H(9A)	-967	4321	5560	46	
H(9B)	672	6165	5674	46	
H(9C)	1854	4167	5951	46	

X-ray Structure of Acetate **6** (CCDC2195935)

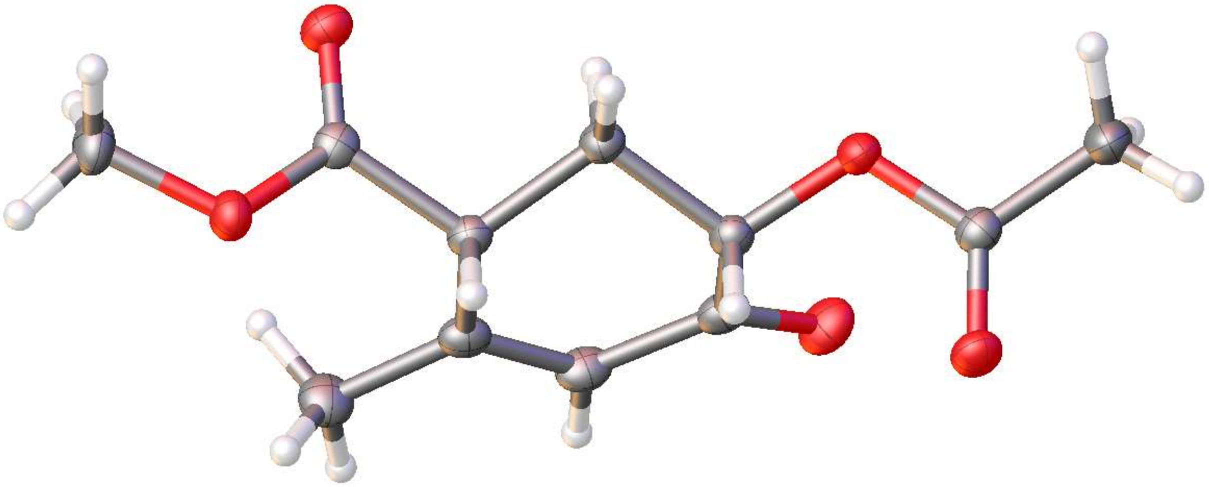

Experimental Summary

The single crystal X-ray diffraction studies were carried out on a Bruker SMART Pt135 CCD diffractometer equipped with Cu K_{α} radiation (λ = 1.54178 Å).

Crystals of the subject compound were used as received. A 0.2 x 0.2 x 0.2 mm piece of a crystal was mounted on a Cryoloop with Paratone oil. Data were collected in a nitrogen gas stream at 100(2) K using ϕ and ω scans. Crystal-to-detector distance was 45 mm and exposure time was 1 or 5 seconds depending on the 2θ range per frame using a scan width of 1.25°. Data collection was 99.8 % complete to 67.679° in θ. A total of 7357 reflections were collected covering the indices, −7<=h<=5, −9<=k<=11, −22<=l<=20. 2157 reflections were found to be symmetry independent, with a R_{int} of 0.0384. Indexing and unit cell refinement indicated a **P**rimitive, **Orthorhombic** lattice. The space group was found to be $\boldsymbol{P2_12_12_1}$. The data were integrated using the Bruker SAINT Software program and scaled using the SADABS software program. Solution by direct methods (SHELXT) produced a complete phasing model consistent with the proposed structure.

All nonhydrogen atoms were refined anisotropically by full-matrix least-squares (SHELXL-2014). All carbon bonded hydrogen atoms were placed using a riding model. Their positions were constrained relative to their parent atom using the appropriate HFIX command in SHELXL-2014. Crystallographic data are summarized in Table S10.1.

Notes: Absolute stereochemistry was conclusively assigned (Absolute Structure Parameter = 0.04(7)). There is one copy of the compound in the asymmetric unit. The chemical formula for the compound is: $C_{11}H_{14}O_5$

Table S10.1. Crystal data and structure refinement for Shenvi287.

Identification code	shenvi287_0m_a
Empirical formula	C11 H14 O5
Formula weight	226.22
Temperature	100.15 K
Wavelength	1.54178 Å
Crystal system	Orthorhombic
Space group	$P2_12_12_1$
Unit cell dimensions	a = 6.2196(3) Å α= 90°.
	b = 9.8099(5) Å β = 90°.
	c = 18.4317(9) Å γ = 90°.
Volume	1124.59(10) Å3
Z	4
Density (calculated)	1.336 Mg/m^3
Absorption coefficient	0.896 mm^{-1}
F(000)	480
Crystal size	0.21 x 0.17 x 0.17 mm^3
Theta range for data collection	4.798 to 70.745°.
Index ranges	-7<=h<=5, -9<=k<=11, -22<=l<=20
Reflections collected	7357
Independent reflections	2157 [R(int) = 0.0384]
Completeness to theta = 67.679°	99.8 %
Absorption correction	Semi-empirical from equivalents
Max. and min. transmission	0.5220 and 0.4456
Refinement method	Full-matrix least-squares on F^2
Data / restraints / parameters	2157 / 0 / 149
Goodness-of-fit on F^2	1.117
Final R indices [I>2sigma(I)]	R1 = 0.0275, wR2 = 0.0698
R indices (all data)	R1 = 0.0280, wR2 = 0.0702
Absolute structure parameter	0.04(7)
Extinction coefficient	0.0048(9)
Largest diff. peak and hole	0.217 and -0.156 e.$Å^{-3}$

Table S10.2. Atomic coordinates (x 10^4) and equivalent isotropic displacement parameters (Å^{2}x 10^3) for Shenvi287. U(eq) is defined as one third of the trace of the orthogonalized U^{ij} tensor.

	x	y	z	U(eq)
O(1)	3122(2)	2788(1)	1495(1)	25(1)
O(2)	2016(2)	3729(1)	2550(1)	21(1)
O(3)	3131(2)	1166(1)	2896(1)	28(1)
O(4)	6059(2)	5422(1)	4833(1)	23(1)
O(5)	9467(2)	5458(1)	4417(1)	22(1)
C(1)	-642(3)	3258(2)	1671(1)	30(1)
C(2)	1686(3)	3214(2)	1871(1)	20(1)
C(3)	4148(3)	3526(2)	2825(1)	19(1)
C(4)	4390(3)	2055(2)	3080(1)	21(1)
C(5)	6242(3)	1793(2)	3547(1)	23(1)
C(6)	7446(3)	2784(2)	3847(1)	21(1)
C(7)	9337(3)	2436(2)	4313(1)	28(1)
C(8)	6926(3)	4287(2)	3704(1)	18(1)
C(9)	7381(3)	5132(2)	4380(1)	19(1)
C(10)	10146(3)	6142(2)	5078(1)	27(1)
C(11)	4600(3)	4499(2)	3451(1)	19(1)

—

Table S10.3. Bond lengths [Å] and angles [°] for Shenvi287.

O(1)-C(2)	1.206(2)
O(2)-C(2)	1.364(2)
O(2)-C(3)	1.433(2)
O(3)-C(4)	1.220(2)
O(4)-C(9)	1.206(2)
O(5)-C(9)	1.338(2)
O(5)-C(10)	1.454(2)
C(1)-H(1A)	0.9800
C(1)-H(1B)	0.9800
C(1)-H(1C)	0.9800
C(1)-C(2)	1.495(2)
C(3)-H(3)	1.0000
C(3)-C(4)	1.526(2)
C(3)-C(11)	1.523(2)
C(4)-C(5)	1.461(2)
C(5)-H(5)	0.9500
C(5)-C(6)	1.346(2)
C(6)-C(7)	1.496(3)
C(6)-C(8)	1.532(2)
C(7)-H(7A)	0.9800
C(7)-H(7B)	0.9800
C(7)-H(7C)	0.9800
C(8)-H(8)	1.0000
C(8)-C(9)	1.524(2)
C(8)-C(11)	1.534(2)
C(10)-H(10A)	0.9800
C(10)-H(10B)	0.9800
C(10)-H(10C)	0.9800
C(11)-H(11A)	0.9900
C(11)-H(11B)	0.9900
C(2)-O(2)-C(3)	114.37(12)
C(9)-O(5)-C(10)	115.74(14)
H(1A)-C(1)-H(1B)	109.5
H(1A)-C(1)-H(1C)	109.5
H(1B)-C(1)-H(1C)	109.5
C(2)-C(1)-H(1A)	109.5
C(2)-C(1)-H(1B)	109.5
C(2)-C(1)-H(1C)	109.5
O(1)-C(2)-O(2)	122.99(16)
O(1)-C(2)-C(1)	125.82(15)
O(2)-C(2)-C(1)	111.19(14)
O(2)-C(3)-H(3)	108.9
O(2)-C(3)-C(4)	109.38(13)
O(2)-C(3)-C(11)	110.61(12)
C(4)-C(3)-H(3)	108.9
C(11)-C(3)-H(3)	108.9
C(11)-C(3)-C(4)	109.98(13)
O(3)-C(4)-C(3)	121.82(16)
O(3)-C(4)-C(5)	122.97(15)

C(5)-C(4)-C(3)	115.21(14)
C(4)-C(5)-H(5)	118.2
C(6)-C(5)-C(4)	123.61(14)
C(6)-C(5)-H(5)	118.2
C(5)-C(6)-C(7)	120.53(15)
C(5)-C(6)-C(8)	120.47(15)
C(7)-C(6)-C(8)	118.99(14)
C(6)-C(7)-H(7A)	109.5
C(6)-C(7)-H(7B)	109.5
C(6)-C(7)-H(7C)	109.5
H(7A)-C(7)-H(7B)	109.5
H(7A)-C(7)-H(7C)	109.5
H(7B)-C(7)-H(7C)	109.5
C(6)-C(8)-H(8)	107.9
C(6)-C(8)-C(11)	112.46(13)
C(9)-C(8)-C(6)	110.09(12)
C(9)-C(8)-H(8)	107.9
C(9)-C(8)-C(11)	110.49(13)
C(11)-C(8)-H(8)	107.9
O(4)-C(9)-O(5)	124.75(15)
O(4)-C(9)-C(8)	124.61(15)
O(5)-C(9)-C(8)	110.58(14)
O(5)-C(10)-H(10A)	109.5
O(5)-C(10)-H(10B)	109.5
O(5)-C(10)-H(10C)	109.5
H(10A)-C(10)-H(10B)	109.5
H(10A)-C(10)-H(10C)	109.5
H(10B)-C(10)-H(10C)	109.5
C(3)-C(11)-C(8)	108.62(13)
C(3)-C(11)-H(11A)	110.0
C(3)-C(11)-H(11B)	110.0
C(8)-C(11)-H(11A)	110.0
C(8)-C(11)-H(11B)	110.0
H(11A)-C(11)-H(11B)	108.3

Symmetry transformations used to generate equivalent atoms:

Table S10.4. Anisotropic displacement parameters ($Å^2$x 10^3) for Shenvi287. The anisotropic displacement factor exponent takes the form: $-2\pi^2$[h2 a*2U11 + ... + 2 h k a* b* U12]

	U11	U22	U33	U23	U13	U12
O(1)	25(1)	29(1)	20(1)	-3(1)	4(1)	0(1)
O(2)	20(1)	22(1)	21(1)	-5(1)	0(1)	1(1)
O(3)	36(1)	20(1)	29(1)	-3(1)	2(1)	-8(1)
O(4)	27(1)	21(1)	21(1)	-1(1)	3(1)	-2(1)
O(5)	22(1)	22(1)	24(1)	-2(1)	-3(1)	-3(1)
C(1)	25(1)	35(1)	30(1)	-12(1)	-4(1)	6(1)
C(2)	23(1)	16(1)	21(1)	0(1)	0(1)	-1(1)
C(3)	18(1)	18(1)	19(1)	-1(1)	2(1)	-1(1)
C(4)	26(1)	17(1)	19(1)	-1(1)	6(1)	-2(1)
C(5)	31(1)	15(1)	24(1)	2(1)	4(1)	3(1)
C(6)	24(1)	19(1)	20(1)	2(1)	5(1)	3(1)
C(7)	31(1)	22(1)	33(1)	3(1)	-4(1)	5(1)
C(8)	19(1)	16(1)	19(1)	1(1)	2(1)	-1(1)
C(9)	23(1)	14(1)	19(1)	4(1)	0(1)	0(1)
C(10)	30(1)	25(1)	28(1)	-3(1)	-9(1)	-4(1)
C(11)	21(1)	16(1)	21(1)	-1(1)	1(1)	0(1)

Table S10.5. Hydrogen coordinates (x 104) and isotropic displacement parameters ($Å^2$x 10^3) for Shenvi287.

	x	y	z	U(eq)
H(1A)	-1396	2499	1905	45
H(1B)	-790	3182	1144	45
H(1C)	-1267	4123	1835	45
H(3)	5205	3703	2428	22
H(5)	6617	872	3645	28
H(7A)	10634	2859	4112	43
H(7B)	9523	1444	4326	43
H(7C)	9094	2776	4806	43
H(8)	7901	4615	3309	21
H(10A)	9690	7098	5063	41
H(10B)	11715	6097	5119	41
H(10C)	9491	5692	5498	41
H(11A)	3595	4317	3856	23
H(11B)	4394	5453	3290	23

X-ray Structure of Iodide **11** (CCDC2211512)

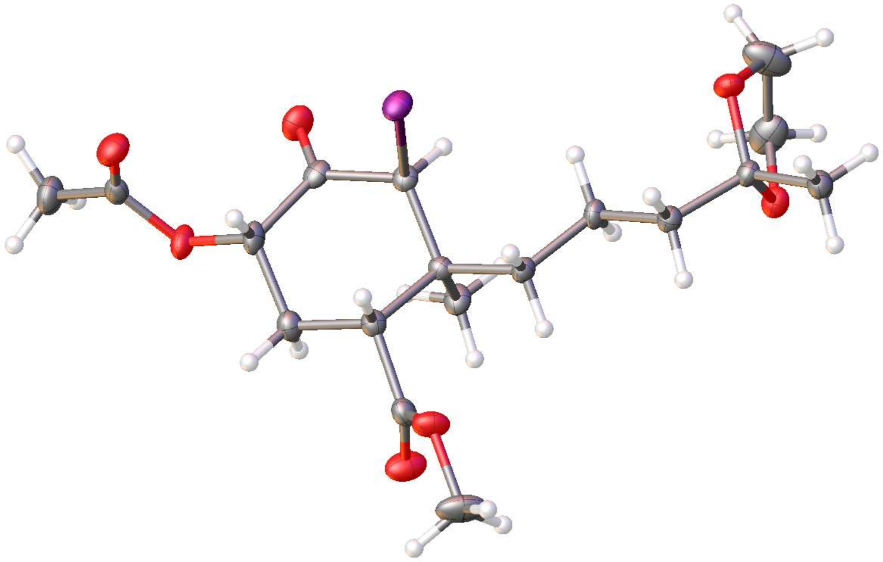

Experimental Summary

The single crystal X-ray diffraction studies were carried out on a Bruker D8-Venture 3-circle diffractometer equipped with a Photon3 detector and Mo K_{α} radiation (λ = 0.7107 Å).

Crystals of the subject compound were used as received. A 0.2 x 0.2 x 0.18 mm piece of a crystal was mounted on a Cryoloop with Paratone oil. Data were collected in a nitrogen gas stream at 100(2) K using ω scans. Crystal-to-detector distance was 50 mm and exposure time was 1 second depending on the 2θ range per frame using a scan width of 0.70°. Data collection was 100 % complete to 25.242° in θ. A total of 23894 reflections were collected covering the indices, −12<=h<=12, −14<=k<=13, −20<=l<=21. 4213 reflections were found to be symmetry independent, with a R_{int} of 0.0404. Indexing and unit cell refinement indicated a **P**rimitive, **Orthorhombic** lattice. The space group was found to be $\boldsymbol{P2_12_12_1}$. The data were integrated using the Bruker SAINT Software program and scaled using the SADABS software program. Solution by direct methods (SHELXT) produced a complete phasing model consistent with the proposed structure.

All nonhydrogen atoms were refined anisotropically by full-matrix least-squares (SHELXL-2014). All carbon bonded hydrogen atoms were placed using a riding model. Their positions were constrained relative to their parent atom using the appropriate HFIX command in SHELXL-2014. Crystallographic data are summarized in Table S11.1.

Notes: Absolute stereochemistry was conclusively assigned (Absolute Structure Parameter = -0.028(9)). There is one copy of the compound in the asymmetric unit. The chemical formula for the compound is: $C_{18}H_{27}IO_7$

Table S11.1. Crystal data and structure refinement for Shenvi288.

Identification code	shenvi288_0m_a	
Empirical formula	C18 H27 I O7	
Formula weight	482.29	
Temperature	100 K	
Wavelength	0.71073 Å	
Crystal system	Orthorhombic	
Space group	$P2_12_12_1$	
Unit cell dimensions	a = 10.2399(5) Å	α= 90°.
	b = 11.8478(6) Å	β= 90°.
	c = 16.9563(6) Å	γ = 90°.
Volume	2057.14(16) Å3	
Z	4	
Density (calculated)	1.557 Mg/m^3	
Absorption coefficient	1.591 mm^{-1}	
F(000)	976	
Crystal size	0.2 x 0.2 x 0.18 mm^3	
Theta range for data collection	2.629 to 26.403°.	
Index ranges	-12<=h<=12, -14<=k<=13, -20<=l<=21	
Reflections collected	23894	
Independent reflections	4213 [R(int) = 0.0404]	
Completeness to theta = 25.242°	99.6 %	
Absorption correction	Semi-empirical from equivalents	
Max. and min. transmission	0.4908 and 0.4347	
Refinement method	Full-matrix least-squares on F^2	
Data / restraints / parameters	4213 / 0 / 239	
Goodness-of-fit on F^2	1.050	
Final R indices [I>2sigma(I)]	R1 = 0.0197, wR2 = 0.0418	
R indices (all data)	R1 = 0.0223, wR2 = 0.0426	
Absolute structure parameter	-0.028(9)	
Largest diff. peak and hole	0.367 and -0.337 e.$Å^{-3}$	

Table S11.2. Atomic coordinates (x 104) and equivalent isotropic displacement parameters (Å^{2}x 10^3) for Shenvi288. U(eq) is defined as one third of the trace of the orthogonalized U^{ij} tensor.

	x	y	z	U(eq)
I(1)	1501(1)	9063(1)	7448(1)	24(1)
O(1)	-286(3)	7169(2)	9703(2)	30(1)
O(2)	1116(2)	5862(2)	9254(1)	26(1)
O(3)	5951(2)	6173(2)	8432(2)	34(1)
O(4)	6082(2)	8056(2)	8422(1)	26(1)
O(5)	4947(2)	7661(2)	4341(1)	22(1)
O(6)	3356(2)	9014(2)	4489(1)	25(1)
O(7)	238(2)	6239(2)	7792(1)	28(1)
C(1)	-625(4)	5218(3)	10043(2)	33(1)
C(2)	44(3)	6197(3)	9658(2)	21(1)
C(3)	1783(3)	6714(3)	8801(2)	21(1)
C(4)	3232(3)	6482(3)	8848(2)	22(1)
C(5)	3979(3)	7259(3)	8278(2)	19(1)
C(6)	5427(3)	7075(3)	8388(2)	21(1)
C(7)	7480(4)	7947(3)	8525(3)	41(1)
C(8)	3555(3)	7075(2)	7406(2)	16(1)
C(9)	4359(3)	7860(3)	6868(2)	18(1)
C(10)	4010(3)	7869(3)	5996(2)	20(1)
C(11)	4840(3)	8740(3)	5562(2)	19(1)
C(12)	4686(4)	8760(3)	4675(2)	18(1)
C(13)	5587(4)	9620(3)	4291(2)	23(1)
C(14)	2974(4)	8259(3)	3878(3)	44(1)
C(15)	3783(4)	7242(4)	3998(2)	38(1)
C(16)	3741(3)	5833(3)	7144(2)	22(1)
C(17)	2073(3)	7300(2)	7344(2)	17(1)
C(18)	1253(3)	6699(3)	7962(2)	19(1)

Table S11.3. Bond lengths [Å] and angles [°] for Shenvi288.

I(1)-C(17)	2.177(3)
O(1)-C(2)	1.203(5)
O(2)-C(2)	1.353(4)
O(2)-C(3)	1.441(4)
O(3)-C(6)	1.198(4)
O(4)-C(6)	1.344(4)
O(4)-C(7)	1.448(4)
O(5)-C(12)	1.444(4)
O(5)-C(15)	1.416(4)
O(6)-C(12)	1.430(4)
O(6)-C(14)	1.423(5)
O(7)-C(18)	1.208(4)
C(1)-H(1A)	0.9800
C(1)-H(1B)	0.9800
C(1)-H(1C)	0.9800
C(1)-C(2)	1.497(5)
C(3)-H(3)	1.0000
C(3)-C(4)	1.511(5)
C(3)-C(18)	1.523(5)
C(4)-H(4A)	0.9900
C(4)-H(4B)	0.9900
C(4)-C(5)	1.538(4)
C(5)-H(5)	1.0000
C(5)-C(6)	1.510(5)
C(5)-C(8)	1.558(4)
C(7)-H(7A)	0.9800
C(7)-H(7B)	0.9800
C(7)-H(7C)	0.9800
C(8)-C(9)	1.541(4)
C(8)-C(16)	1.548(4)
C(8)-C(17)	1.545(4)
C(9)-H(9A)	0.9900
C(9)-H(9B)	0.9900
C(9)-C(10)	1.520(4)
C(10)-H(10A)	0.9900
C(10)-H(10B)	0.9900
C(10)-C(11)	1.526(4)
C(11)-H(11A)	0.9900
C(11)-H(11B)	0.9900
C(11)-C(12)	1.512(4)
C(12)-C(13)	1.521(5)
C(13)-H(13A)	0.9800
C(13)-H(13B)	0.9800
C(13)-H(13C)	0.9800
C(14)-H(14A)	0.9900
C(14)-H(14B)	0.9900
C(14)-C(15)	1.476(6)
C(15)-H(15A)	0.9900
C(15)-H(15B)	0.9900
C(16)-H(16A)	0.9800
C(16)-H(16B)	0.9800
C(16)-H(16C)	0.9800
C(17)-H(17)	1.0000
C(17)-C(18)	1.520(4)

C(2)-O(2)-C(3)	116.7(3)
C(6)-O(4)-C(7)	114.9(3)
C(15)-O(5)-C(12)	108.7(3)
C(14)-O(6)-C(12)	106.9(3)
H(1A)-C(1)-H(1B)	109.5
H(1A)-C(1)-H(1C)	109.5
H(1B)-C(1)-H(1C)	109.5
C(2)-C(1)-H(1A)	109.5
C(2)-C(1)-H(1B)	109.5
C(2)-C(1)-H(1C)	109.5
O(1)-C(2)-O(2)	122.8(3)
O(1)-C(2)-C(1)	125.8(3)
O(2)-C(2)-C(1)	111.4(3)
O(2)-C(3)-H(3)	108.9
O(2)-C(3)-C(4)	108.1(3)
O(2)-C(3)-C(18)	108.7(3)
C(4)-C(3)-H(3)	108.9
C(4)-C(3)-C(18)	113.4(3)
C(18)-C(3)-H(3)	108.9
C(3)-C(4)-H(4A)	109.6
C(3)-C(4)-H(4B)	109.6
C(3)-C(4)-C(5)	110.3(3)
H(4A)-C(4)-H(4B)	108.1
C(5)-C(4)-H(4A)	109.6
C(5)-C(4)-H(4B)	109.6
C(4)-C(5)-H(5)	108.0
C(4)-C(5)-C(8)	112.0(3)
C(6)-C(5)-C(4)	108.9(3)
C(6)-C(5)-H(5)	108.0
C(6)-C(5)-C(8)	111.8(3)
C(8)-C(5)-H(5)	108.0
O(3)-C(6)-O(4)	123.0(3)
O(3)-C(6)-C(5)	125.2(3)
O(4)-C(6)-C(5)	111.7(3)
O(4)-C(7)-H(7A)	109.5
O(4)-C(7)-H(7B)	109.5
O(4)-C(7)-H(7C)	109.5
H(7A)-C(7)-H(7B)	109.5
H(7A)-C(7)-H(7C)	109.5
H(7B)-C(7)-H(7C)	109.5
C(9)-C(8)-C(5)	109.2(2)
C(9)-C(8)-C(16)	109.8(2)
C(9)-C(8)-C(17)	112.4(2)

C(16)-C(8)-C(5)	111.7(2)	H(13A)-C(13)-H(13B)	109.5
C(17)-C(8)-C(5)	108.3(2)	H(13A)-C(13)-H(13C)	109.5
C(17)-C(8)-C(16)	105.4(2)	H(13B)-C(13)-H(13C)	109.5
C(8)-C(9)-H(9A)	108.0	O(6)-C(14)-H(14A)	110.8
C(8)-C(9)-H(9B)	108.0	O(6)-C(14)-H(14B)	110.8
H(9A)-C(9)-H(9B)	107.3	O(6)-C(14)-C(15)	105.0(3)
C(10)-C(9)-C(8)	117.0(3)	H(14A)-C(14)-H(14B)	108.8
C(10)-C(9)-H(9A)	108.0	C(15)-C(14)-H(14A)	110.8
C(10)-C(9)-H(9B)	108.0	C(15)-C(14)-H(14B)	110.8
C(9)-C(10)-H(10A)	109.6	O(5)-C(15)-C(14)	104.0(3)
C(9)-C(10)-H(10B)	109.6	O(5)-C(15)-H(15A)	110.9
C(9)-C(10)-C(11)	110.1(3)	O(5)-C(15)-H(15B)	110.9
H(10A)-C(10)-H(10B)	108.2	C(14)-C(15)-H(15A)	110.9
C(11)-C(10)-H(10A)	109.6	C(14)-C(15)-H(15B)	110.9
C(11)-C(10)-H(10B)	109.6	H(15A)-C(15)-H(15B)	109.0
C(10)-C(11)-H(11A)	108.4	C(8)-C(16)-H(16A)	109.5
C(10)-C(11)-H(11B)	108.4	C(8)-C(16)-H(16B)	109.5
H(11A)-C(11)-H(11B)	107.4	C(8)-C(16)-H(16C)	109.5
C(12)-C(11)-C(10)	115.6(3)	H(16A)-C(16)-H(16B)	109.5
C(12)-C(11)-H(11A)	108.4	H(16A)-C(16)-H(16C)	109.5
C(12)-C(11)-H(11B)	108.4	H(16B)-C(16)-H(16C)	109.5
O(5)-C(12)-C(11)	110.9(3)	I(1)-C(17)-H(17)	107.6
O(5)-C(12)-C(13)	108.9(3)	C(8)-C(17)-I(1)	115.11(18)
O(6)-C(12)-O(5)	106.2(3)	C(8)-C(17)-H(17)	107.6
O(6)-C(12)-C(11)	108.8(3)	C(18)-C(17)-I(1)	104.17(19)
O(6)-C(12)-C(13)	110.0(3)	C(18)-C(17)-C(8)	114.5(2)
C(11)-C(12)-C(13)	111.9(3)	C(18)-C(17)-H(17)	107.6
C(12)-C(13)-H(13A)	109.5	O(7)-C(18)-C(3)	122.3(3)
C(12)-C(13)-H(13B)	109.5	O(7)-C(18)-C(17)	121.4(3)
C(12)-C(13)-H(13C)	109.5	C(17)-C(18)-C(3)	116.3(3)

Symmetry transformations used to generate equivalent atoms:

Table S11.4. Anisotropic displacement parameters ($Å^2$x 10^3) for Shenvi288. The anisotropic displacement factor exponent takes the form: $-2\pi^2$[h2 a*2U11 + ... + 2 h k a* b* U12]

	U11	U22	U33	U23	U13	U12
I(1)	24(1)	16(1)	31(1)	1(1)	6(1)	4(1)
O(1)	26(2)	30(2)	35(2)	-1(1)	6(1)	4(1)
O(2)	28(1)	22(1)	27(1)	5(1)	12(1)	1(1)
O(3)	26(1)	22(1)	54(2)	7(1)	-2(1)	1(1)
O(4)	20(1)	23(1)	36(1)	1(1)	-9(1)	-3(1)
O(5)	23(1)	20(1)	23(1)	-4(1)	2(1)	-3(1)
O(6)	17(1)	32(1)	26(1)	1(1)	-2(1)	2(1)
O(7)	25(1)	26(1)	33(1)	-2(1)	4(1)	-8(1)
C(1)	33(2)	36(2)	31(2)	6(2)	12(2)	-4(2)
C(2)	18(2)	30(2)	15(2)	1(1)	-2(1)	-4(2)
C(3)	26(2)	18(2)	20(2)	2(1)	8(1)	-4(1)
C(4)	25(2)	22(2)	20(2)	2(1)	2(1)	-3(1)
C(5)	21(2)	16(2)	19(2)	0(1)	1(1)	-1(1)

C(6)	24(2)	22(2)	18(2)	4(1)	0(1)	-2(2)
C(7)	24(2)	30(2)	69(3)	0(2)	-18(2)	-3(2)
C(8)	18(1)	15(1)	16(1)	0(1)	-1(2)	0(1)
C(9)	16(2)	18(2)	19(2)	1(1)	3(1)	-1(1)
C(10)	21(2)	19(2)	18(2)	2(1)	6(1)	-3(1)
C(11)	18(2)	19(2)	19(2)	-1(1)	1(1)	-1(1)
C(12)	17(2)	19(2)	18(2)	0(1)	4(1)	1(1)
C(13)	24(2)	25(2)	20(2)	2(1)	1(1)	-2(2)
C(14)	46(3)	35(2)	50(3)	6(2)	-24(2)	-11(2)
C(15)	32(2)	42(2)	41(2)	-16(2)	-1(2)	-8(2)
C(16)	27(2)	16(2)	23(2)	-2(1)	4(1)	2(1)
C(17)	20(2)	12(1)	19(2)	-2(1)	2(1)	1(1)
C(18)	19(2)	14(2)	26(2)	-2(1)	5(1)	2(1)

Table S11.5. Hydrogen coordinates (x 10^4) and isotropic displacement parameters ($Å^2$x 10^3) for Shenvi288.

	x	y	z	U(eq)
H(1A)	-581	4559	9696	50
H(1B)	-1541	5410	10143	50
H(1C)	-191	5045	10544	50
H(3)	1603	7470	9040	26
H(4A)	3404	5683	8710	27
H(4B)	3542	6611	9394	27
H(5)	3777	8058	8424	22
H(7A)	7828	7423	8131	62
H(7B)	7664	7656	9055	62
H(7C)	7893	8687	8461	62
H(9A)	4273	8639	7071	21
H(9B)	5290	7645	6917	21
H(10A)	3073	8056	5932	23
H(10B)	4162	7112	5768	23
H(11A)	4621	9498	5769	23
H(11B)	5770	8595	5686	23
H(13A)	6497	9419	4400	34
H(13B)	5403	10372	4506	34
H(13C)	5439	9625	3720	34
H(14A)	3142	8595	3353	52
H(14B)	2034	8073	3920	52
H(15A)	3344	6702	4356	46
H(15B)	3966	6862	3490	46
H(16A)	3171	5344	7459	33
H(16B)	4653	5611	7224	33
H(16C)	3516	5759	6585	33
H(17)	1778	7028	6814	21

X-ray Structure of Ferrocene Carboxylate **S5** (CCDC2241886)

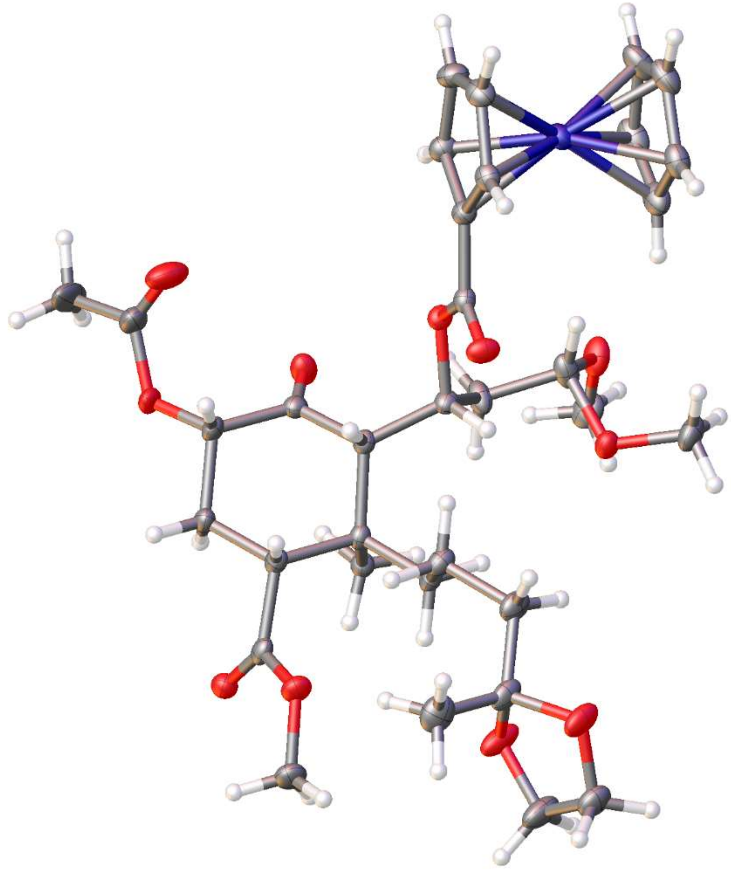

Experimental Summary

The single crystal X-ray diffraction studies were carried out on a Bruker D8-Venture 3-circle diffractometer equipped with a Photon3 detector and Mo K_α radiation (λ = 0.7107 Å).

Crystals of the subject compound were used as received. A 0.22 x 0.08 x 0.035 mm piece of a crystal was mounted on a Cryoloop with Paratone oil. Data were collected in a nitrogen gas stream at 100(2) K using ω scans. Crystal-to-detector distance was 50 mm and exposure time was 1 or 4 seconds per frame using a scan width of 0.70°. Data collection was 99.8 % complete to 25.242° in θ. A total of 38780 reflections were collected covering the indices, −13<=h<=12, −14<=k<=14, −17<=l<=17. 6613 reflections were found to be symmetry independent, with a R_{int} of 0.0309. Indexing and unit cell refinement indicated a **P**rimitive, **Monoclinic** lattice. The space group was found to be $\boldsymbol{P2_1}$. The data were integrated using the Bruker SAINT Software program and scaled using the SADABS software program. Solution by direct methods (SHELXT) produced a complete phasing model consistent with the proposed structure.

All nonhydrogen atoms were refined anisotropically by full-matrix least-squares (SHELXL-2014). All carbon bonded hydrogen atoms were placed using a riding model. Their positions were constrained relative to their parent atom using the appropriate HFIX command in SHELXL-2014. Crystallographic data are summarized in Table S12.1.

Notes: Absolute stereochemistry was conclusively assigned (Absolute Structure Parameter = 0.021(4)). There is one copy of the compound in the asymmetric unit. There is some positional disorder in the compound. The chemical formula for the compound is: $C_{34}H_{46}FeO_{11}$

Table S12.1. Crystal data and structure refinement for Shenvi295.

Identification code	shenvi295	
Empirical formula	C34 H46 Fe O11	
Formula weight	686.56	
Temperature	100.15 K	
Wavelength	0.71073 Å	
Crystal system	Monoclinic	
Space group	P 1 21 1	
Unit cell dimensions	a = 10.4370(3) Å	α= 90°.
	b = 11.2575(4) Å	β= 95.6906(11)°.
	c = 13.8833(4) Å	γ = 90°.
Volume	1623.17(9) Å3	
Z	2	
Density (calculated)	1.405 Mg/m^3	
Absorption coefficient	0.526 mm^{-1}	
F(000)	728	
Crystal size	0.22 x 0.08 x 0.035 mm^3	
Theta range for data collection	2.668 to 26.372°.	
Index ranges	-13<=h<=12, -14<=k<=14, -17<=l<=17	
Reflections collected	38780	
Independent reflections	6613 [R(int) = 0.0309]	
Completeness to theta = 25.242°	99.8 %	
Absorption correction	Semi-empirical from equivalents	
Max. and min. transmission	0.7454 and 0.7010	
Refinement method	Full-matrix least-squares on F^2	
Data / restraints / parameters	6613 / 2 / 451	
Goodness-of-fit on F^2	1.045	
Final R indices [I>2sigma(I)]	R1 = 0.0234, wR2 = 0.0527	
R indices (all data)	R1 = 0.0246, wR2 = 0.0532	
Absolute structure parameter	0.021(4)	
Largest diff. peak and hole	0.206 and -0.195 e.Å^{-3}	

Table S12.2. Atomic coordinates (x 10^4) and equivalent isotropic displacement parameters ($Å^2$x 10^3) for Shenvi295. U(eq) is defined as one third of the trace of the orthogonalized U^{ij} tensor.

	x	y	z	U(eq)
Fe(1)	4160(1)	1505(1)	1238(1)	21(1)
O(8)	4191(1)	4697(1)	2164(1)	18(1)
O(3)	4044(2)	9928(2)	5016(1)	24(1)
O(5)	7339(2)	8162(2)	3131(1)	28(1)
O(4)	3218(2)	8419(2)	5806(1)	26(1)
O(7)	5434(2)	7234(2)	1897(1)	27(1)
O(9)	3528(2)	3561(1)	3362(1)	25(1)
O(11)	1446(2)	5778(2)	-9(1)	32(1)
O(2)	62(2)	7361(2)	5464(1)	35(1)
O(6)	8206(2)	6423(2)	2736(2)	46(1)
O(1)	-720(2)	5636(2)	6008(2)	34(1)
C(14)	5332(2)	7071(2)	2745(2)	19(1)
C(10)	3898(2)	8875(2)	5128(2)	20(1)
O(10)	742(2)	5567(2)	1472(2)	23(1)
C(13)	4181(2)	6479(2)	3150(1)	17(1)
C(20)	4117(2)	3666(2)	2660(2)	18(1)
C(31)	2822(2)	6214(2)	1459(2)	25(1)
C(32)	1858(2)	5397(2)	924(2)	27(1)
C(21)	4862(2)	2707(2)	2259(2)	19(1)
C(22)	4951(2)	1525(3)	2645(1)	20(1)
C(15)	6325(2)	7524(2)	3535(2)	21(1)
C(28)	2409(2)	673(2)	1186(2)	28(1)
C(8)	3448(2)	7478(2)	3679(2)	18(1)
C(27)	3239(3)	59(2)	610(2)	30(1)
C(19)	3371(2)	5684(2)	2424(2)	19(1)
C(7)	2205(2)	6992(2)	4059(2)	19(1)
C(9)	4451(2)	7908(2)	4532(2)	19(1)
C(24)	6128(2)	1611(3)	1332(2)	27(1)
C(23)	5730(2)	857(2)	2073(2)	25(1)
C(25)	5592(2)	2750(2)	1441(2)	22(1)
C(16)	5693(2)	8402(2)	4176(2)	21(1)
C(17)	8228(2)	7487(3)	2729(2)	37(1)
C(12)	3049(2)	8540(2)	3015(2)	19(1)
C(11)	2580(2)	9289(2)	6365(2)	31(1)
C(6)	2344(2)	5934(2)	4753(2)	23(1)
C(29)	2267(2)	1848(2)	816(2)	30(1)
C(5)	1039(2)	5510(2)	5065(2)	27(1)
C(26)	3597(3)	843(2)	-116(2)	32(1)
C(30)	2999(3)	1949(2)	12(2)	32(1)
C(1)	-1600(2)	6532(3)	6237(2)	32(1)
C(3)	457(2)	6227(2)	5830(2)	27(1)
C(4)	1324(2)	6337(3)	6762(2)	37(1)
C(2)	-1072(3)	7662(3)	5860(2)	40(1)
C(18)	9203(3)	8249(3)	2298(3)	63(1)
C(34)	1028(4)	6928(3)	-231(3)	32(1)
C(33)	-290(4)	4754(4)	1205(3)	32(1)

O(10A)	1214(5)	4486(5)	1370(4)	21(2)
C(33A)	136(9)	5041(9)	1730(8)	30(2)
C(34A)	936(8)	5100(8)	-648(6)	29(2)

Table S12.3. Bond lengths [Å] and angles [°] for Shenvi295.

Fe(1)-C(21)	2.042(2)
Fe(1)-C(22)	2.0448(19)
Fe(1)-C(28)	2.049(2)
Fe(1)-C(27)	2.041(2)
Fe(1)-C(24)	2.048(2)
Fe(1)-C(23)	2.046(2)
Fe(1)-C(25)	2.048(2)
Fe(1)-C(29)	2.042(2)
Fe(1)-C(26)	2.052(2)
Fe(1)-C(30)	2.050(2)
O(8)-C(20)	1.356(3)
O(8)-C(19)	1.469(3)
O(3)-C(10)	1.208(3)
O(5)-C(15)	1.437(3)
O(5)-C(17)	1.362(3)
O(4)-C(10)	1.336(3)
O(4)-C(11)	1.452(3)
O(7)-C(14)	1.206(3)
O(9)-C(20)	1.209(3)
O(11)-C(32)	1.391(3)
O(11)-C(34)	1.391(4)
O(11)-C(34A)	1.249(8)
O(2)-C(3)	1.420(3)
O(2)-C(2)	1.395(3)
O(6)-C(17)	1.198(4)
O(1)-C(1)	1.421(3)
O(1)-C(3)	1.440(3)
C(14)-C(13)	1.529(3)
C(14)-C(15)	1.520(3)
C(10)-C(9)	1.517(3)
O(10)-C(32)	1.465(3)
O(10)-C(33)	1.434(5)
C(13)-H(13)	1.0000
C(13)-C(8)	1.581(3)
C(13)-C(19)	1.537(3)
C(20)-C(21)	1.472(3)
C(31)-H(31A)	0.9900
C(31)-H(31B)	0.9900
C(31)-C(32)	1.503(3)
C(31)-C(19)	1.526(3)
C(32)-H(32)	1.0000
C(32)-H(32A)	1.0000
C(32)-O(10A)	1.403(5)
C(21)-C(22)	1.434(4)
C(21)-C(25)	1.430(3)
C(22)-H(22)	0.9500
C(22)-C(23)	1.409(3)
C(15)-H(15)	1.0000
C(15)-C(16)	1.524(3)
C(28)-H(28)	0.9500
C(28)-C(27)	1.415(4)
C(28)-C(29)	1.422(4)
C(8)-C(7)	1.548(3)
C(8)-C(9)	1.576(3)
C(8)-C(12)	1.541(3)
C(27)-H(27)	0.9500
C(27)-C(26)	1.418(4)
C(19)-H(19)	1.0000
C(7)-H(7A)	0.9900
C(7)-H(7B)	0.9900
C(7)-C(6)	1.529(3)
C(9)-H(9)	1.0000
C(9)-C(16)	1.536(3)
C(24)-H(24)	0.9500
C(24)-C(23)	1.427(3)
C(24)-C(25)	1.414(4)
C(23)-H(23)	0.9500
C(25)-H(25)	0.9500
C(16)-H(16A)	0.9900
C(16)-H(16B)	0.9900
C(17)-C(18)	1.500(4)
C(12)-H(12A)	0.9800
C(12)-H(12B)	0.9800
C(12)-H(12C)	0.9800
C(11)-H(11A)	0.9800
C(11)-H(11B)	0.9800
C(11)-H(11C)	0.9800
C(6)-H(6A)	0.9900
C(6)-H(6B)	0.9900
C(6)-C(5)	1.545(3)
C(29)-H(29)	0.9500
C(29)-C(30)	1.419(4)
C(5)-H(5A)	0.9900
C(5)-H(5B)	0.9900
C(5)-C(3)	1.508(3)
C(26)-H(26)	0.9500
C(26)-C(30)	1.411(4)
C(30)-H(30)	0.9500
C(1)-H(1A)	0.9900
C(1)-H(1B)	0.9900
C(1)-C(2)	1.501(4)
C(3)-C(4)	1.509(3)
C(4)-H(4A)	0.9800
C(4)-H(4B)	0.9800
C(4)-H(4C)	0.9800
C(2)-H(2A)	0.9900
C(2)-H(2B)	0.9900
C(18)-H(18A)	0.9800

C(18)-H(18B)	0.9800
C(18)-H(18C)	0.9800
C(34)-H(34A)	0.9800
C(34)-H(34B)	0.9800
C(34)-H(34C)	0.9800
C(33)-H(33A)	0.9800
C(33)-H(33B)	0.9800
C(33)-H(33C)	0.9800
O(10A)-C(33A)	1.420(10)
C(33A)-H(33D)	0.9800
C(33A)-H(33E)	0.9800
C(33A)-H(33F)	0.9800
C(34A)-H(34D)	0.9800
C(34A)-H(34E)	0.9800
C(34A)-H(34F)	0.9800

C(21)-Fe(1)-C(22)	41.08(10)
C(21)-Fe(1)-C(28)	125.76(9)
C(21)-Fe(1)-C(24)	68.22(9)
C(21)-Fe(1)-C(23)	68.35(9)
C(21)-Fe(1)-C(25)	40.92(8)
C(21)-Fe(1)-C(26)	157.90(9)
C(21)-Fe(1)-C(30)	123.80(9)
C(22)-Fe(1)-C(28)	108.23(9)
C(22)-Fe(1)-C(24)	68.35(8)
C(22)-Fe(1)-C(23)	40.31(9)
C(22)-Fe(1)-C(25)	68.94(9)
C(22)-Fe(1)-C(26)	158.59(10)
C(22)-Fe(1)-C(30)	160.00(10)
C(28)-Fe(1)-C(26)	68.16(10)
C(28)-Fe(1)-C(30)	68.30(10)
C(27)-Fe(1)-C(21)	161.03(9)
C(27)-Fe(1)-C(22)	123.03(10)
C(27)-Fe(1)-C(28)	40.48(10)
C(27)-Fe(1)-C(24)	120.05(11)
C(27)-Fe(1)-C(23)	105.92(10)
C(27)-Fe(1)-C(25)	155.68(10)
C(27)-Fe(1)-C(29)	68.11(10)
C(27)-Fe(1)-C(26)	40.53(10)
C(27)-Fe(1)-C(30)	68.01(10)
C(24)-Fe(1)-C(28)	156.09(11)
C(24)-Fe(1)-C(26)	105.93(10)
C(24)-Fe(1)-C(30)	123.05(10)
C(23)-Fe(1)-C(28)	121.21(10)
C(23)-Fe(1)-C(24)	40.80(10)
C(23)-Fe(1)-C(25)	68.52(9)
C(23)-Fe(1)-C(26)	122.04(11)
C(23)-Fe(1)-C(30)	158.70(10)
C(25)-Fe(1)-C(28)	162.55(10)
C(25)-Fe(1)-C(24)	40.38(10)
C(25)-Fe(1)-C(26)	120.92(10)
C(25)-Fe(1)-C(30)	108.11(10)
C(29)-Fe(1)-C(21)	110.08(9)
C(29)-Fe(1)-C(22)	123.99(9)
C(29)-Fe(1)-C(28)	40.68(10)
C(29)-Fe(1)-C(24)	160.61(10)
C(29)-Fe(1)-C(23)	158.11(10)
C(29)-Fe(1)-C(25)	125.60(10)
C(29)-Fe(1)-C(26)	67.98(11)
C(29)-Fe(1)-C(30)	40.58(11)
C(30)-Fe(1)-C(26)	40.23(10)
C(20)-O(8)-C(19)	117.27(16)
C(17)-O(5)-C(15)	116.0(2)
C(10)-O(4)-C(11)	114.95(19)
C(34)-O(11)-C(32)	123.7(2)
C(34A)-O(11)-C(32)	123.0(5)
C(2)-O(2)-C(3)	107.82(19)
C(1)-O(1)-C(3)	106.99(19)
O(7)-C(14)-C(13)	124.99(19)
O(7)-C(14)-C(15)	122.2(2)
C(15)-C(14)-C(13)	112.67(18)
O(3)-C(10)-O(4)	123.4(2)
O(3)-C(10)-C(9)	125.1(2)
O(4)-C(10)-C(9)	111.5(2)
C(33)-O(10)-C(32)	113.4(2)
C(14)-C(13)-H(13)	105.7
C(14)-C(13)-C(8)	107.15(19)
C(14)-C(13)-C(19)	114.44(16)
C(8)-C(13)-H(13)	105.7
C(19)-C(13)-H(13)	105.7
C(19)-C(13)-C(8)	117.29(16)
O(8)-C(20)-C(21)	112.16(18)
O(9)-C(20)-O(8)	123.6(2)
O(9)-C(20)-C(21)	124.2(2)
H(31A)-C(31)-H(31B)	108.0
C(32)-C(31)-H(31A)	109.3
C(32)-C(31)-H(31B)	109.3
C(32)-C(31)-C(19)	111.66(19)
C(19)-C(31)-H(31A)	109.3
C(19)-C(31)-H(31B)	109.3
O(11)-C(32)-O(10)	104.8(2)
O(11)-C(32)-C(31)	113.62(19)
O(11)-C(32)-H(32)	112.2
O(11)-C(32)-H(32A)	93.9
O(11)-C(32)-O(10A)	121.2(3)
O(10)-C(32)-C(31)	101.0(2)
O(10)-C(32)-H(32)	112.2
C(31)-C(32)-H(32)	112.2
C(31)-C(32)-H(32A)	93.9
O(10A)-C(32)-C(31)	123.8(3)
O(10A)-C(32)-H(32A)	93.9
C(20)-C(21)-Fe(1)	125.63(15)

C(22)-C(21)-Fe(1)	69.56(12)
C(22)-C(21)-C(20)	123.82(19)
C(25)-C(21)-Fe(1)	69.75(13)
C(25)-C(21)-C(20)	128.2(2)
C(25)-C(21)-C(22)	107.98(19)
Fe(1)-C(22)-H(22)	126.2
C(21)-C(22)-Fe(1)	69.37(12)
C(21)-C(22)-H(22)	126.1
C(23)-C(22)-Fe(1)	69.88(12)
C(23)-C(22)-C(21)	107.73(18)
C(23)-C(22)-H(22)	126.1
O(5)-C(15)-C(14)	111.23(18)
O(5)-C(15)-H(15)	109.9
O(5)-C(15)-C(16)	106.33(18)
C(14)-C(15)-H(15)	109.9
C(14)-C(15)-C(16)	109.55(17)
C(16)-C(15)-H(15)	109.9
Fe(1)-C(28)-H(28)	126.4
C(27)-C(28)-Fe(1)	69.46(14)
C(27)-C(28)-H(28)	126.3
C(27)-C(28)-C(29)	107.4(2)
C(29)-C(28)-Fe(1)	69.38(15)
C(29)-C(28)-H(28)	126.3
C(7)-C(8)-C(13)	111.56(18)
C(7)-C(8)-C(9)	111.69(17)
C(9)-C(8)-C(13)	104.54(16)
C(12)-C(8)-C(13)	112.85(17)
C(12)-C(8)-C(7)	106.95(17)
C(12)-C(8)-C(9)	109.32(17)
Fe(1)-C(27)-H(27)	125.6
C(28)-C(27)-Fe(1)	70.05(14)
C(28)-C(27)-H(27)	125.8
C(28)-C(27)-C(26)	108.4(2)
C(26)-C(27)-Fe(1)	70.16(14)
C(26)-C(27)-H(27)	125.8
O(8)-C(19)-C(13)	107.80(16)
O(8)-C(19)-C(31)	104.85(17)
O(8)-C(19)-H(19)	108.3
C(13)-C(19)-H(19)	108.3
C(31)-C(19)-C(13)	118.87(19)
C(31)-C(19)-H(19)	108.3
C(8)-C(7)-H(7A)	108.0
C(8)-C(7)-H(7B)	108.0
H(7A)-C(7)-H(7B)	107.2
C(6)-C(7)-C(8)	117.33(18)
C(6)-C(7)-H(7A)	108.0
C(6)-C(7)-H(7B)	108.0
C(10)-C(9)-C(8)	111.70(17)
C(10)-C(9)-H(9)	108.3
C(10)-C(9)-C(16)	107.13(18)
C(8)-C(9)-H(9)	108.3

C(16)-C(9)-C(8)	112.86(17)
C(16)-C(9)-H(9)	108.3
Fe(1)-C(24)-H(24)	126.5
C(23)-C(24)-Fe(1)	69.51(13)
C(23)-C(24)-H(24)	125.8
C(25)-C(24)-Fe(1)	69.81(13)
C(25)-C(24)-H(24)	125.8
C(25)-C(24)-C(23)	108.43(19)
Fe(1)-C(23)-H(23)	126.2
C(22)-C(23)-Fe(1)	69.82(12)
C(22)-C(23)-C(24)	108.3(2)
C(22)-C(23)-H(23)	125.9
C(24)-C(23)-Fe(1)	69.69(13)
C(24)-C(23)-H(23)	125.9
Fe(1)-C(25)-H(25)	126.2
C(21)-C(25)-Fe(1)	69.33(13)
C(21)-C(25)-H(25)	126.2
C(24)-C(25)-Fe(1)	69.81(14)
C(24)-C(25)-C(21)	107.6(2)
C(24)-C(25)-H(25)	126.2
C(15)-C(16)-C(9)	112.38(18)
C(15)-C(16)-H(16A)	109.1
C(15)-C(16)-H(16B)	109.1
C(9)-C(16)-H(16A)	109.1
C(9)-C(16)-H(16B)	109.1
H(16A)-C(16)-H(16B)	107.9
O(5)-C(17)-C(18)	111.2(3)
O(6)-C(17)-O(5)	122.7(2)
O(6)-C(17)-C(18)	126.1(2)
C(8)-C(12)-H(12A)	109.5
C(8)-C(12)-H(12B)	109.5
C(8)-C(12)-H(12C)	109.5
H(12A)-C(12)-H(12B)	109.5
H(12A)-C(12)-H(12C)	109.5
H(12B)-C(12)-H(12C)	109.5
O(4)-C(11)-H(11A)	109.5
O(4)-C(11)-H(11B)	109.5
O(4)-C(11)-H(11C)	109.5
H(11A)-C(11)-H(11B)	109.5
H(11A)-C(11)-H(11C)	109.5
H(11B)-C(11)-H(11C)	109.5
C(7)-C(6)-H(6A)	109.0
C(7)-C(6)-H(6B)	109.0
C(7)-C(6)-C(5)	112.72(19)
H(6A)-C(6)-H(6B)	107.8
C(5)-C(6)-H(6A)	109.0
C(5)-C(6)-H(6B)	109.0
Fe(1)-C(29)-H(29)	125.7
C(28)-C(29)-Fe(1)	69.94(14)
C(28)-C(29)-H(29)	125.9
C(30)-C(29)-Fe(1)	70.03(15)

C(30)-C(29)-C(28)	108.2(2)
C(30)-C(29)-H(29)	125.9
C(6)-C(5)-H(5A)	108.0
C(6)-C(5)-H(5B)	108.0
H(5A)-C(5)-H(5B)	107.2
C(3)-C(5)-C(6)	117.4(2)
C(3)-C(5)-H(5A)	108.0
C(3)-C(5)-H(5B)	108.0
Fe(1)-C(26)-H(26)	126.5
C(27)-C(26)-Fe(1)	69.32(14)
C(27)-C(26)-H(26)	126.0
C(30)-C(26)-Fe(1)	69.80(15)
C(30)-C(26)-C(27)	108.0(2)
C(30)-C(26)-H(26)	126.0
Fe(1)-C(30)-H(30)	126.2
C(29)-C(30)-Fe(1)	69.39(14)
C(29)-C(30)-H(30)	126.0
C(26)-C(30)-Fe(1)	69.97(15)
C(26)-C(30)-C(29)	108.0(2)
C(26)-C(30)-H(30)	126.0
O(1)-C(1)-H(1A)	110.8
O(1)-C(1)-H(1B)	110.8
O(1)-C(1)-C(2)	104.85(18)
H(1A)-C(1)-H(1B)	108.9
C(2)-C(1)-H(1A)	110.8
C(2)-C(1)-H(1B)	110.8
O(2)-C(3)-O(1)	104.71(18)
O(2)-C(3)-C(5)	110.65(19)
O(2)-C(3)-C(4)	111.1(2)
O(1)-C(3)-C(5)	106.54(19)
O(1)-C(3)-C(4)	109.81(19)
C(5)-C(3)-C(4)	113.5(2)
C(3)-C(4)-H(4A)	109.5
C(3)-C(4)-H(4B)	109.5
C(3)-C(4)-H(4C)	109.5
H(4A)-C(4)-H(4B)	109.5
H(4A)-C(4)-H(4C)	109.5
H(4B)-C(4)-H(4C)	109.5
O(2)-C(2)-C(1)	106.6(2)
O(2)-C(2)-H(2A)	110.4
O(2)-C(2)-H(2B)	110.4
C(1)-C(2)-H(2A)	110.4
C(1)-C(2)-H(2B)	110.4
H(2A)-C(2)-H(2B)	108.6
C(17)-C(18)-H(18A)	109.5
C(17)-C(18)-H(18B)	109.5
C(17)-C(18)-H(18C)	109.5
H(18A)-C(18)-H(18B)	109.5
H(18A)-C(18)-H(18C)	109.5
H(18B)-C(18)-H(18C)	109.5
O(11)-C(34)-H(34A)	109.5
O(11)-C(34)-H(34B)	109.5
O(11)-C(34)-H(34C)	109.5
H(34A)-C(34)-H(34B)	109.5
H(34A)-C(34)-H(34C)	109.5
H(34B)-C(34)-H(34C)	109.5
O(10)-C(33)-H(33A)	109.5
O(10)-C(33)-H(33B)	109.5
O(10)-C(33)-H(33C)	109.5
H(33A)-C(33)-H(33B)	109.5
H(33A)-C(33)-H(33C)	109.5
H(33B)-C(33)-H(33C)	109.5
C(32)-O(10A)-C(33A)	105.3(6)
O(10A)-C(33A)-H(33D)	109.5
O(10A)-C(33A)-H(33E)	109.5
O(10A)-C(33A)-H(33F)	109.5
H(33D)-C(33A)-H(33E)	109.5
H(33D)-C(33A)-H(33F)	109.5
H(33E)-C(33A)-H(33F)	109.5
O(11)-C(34A)-H(34D)	109.5
O(11)-C(34A)-H(34E)	109.5
O(11)-C(34A)-H(34F)	109.5
H(34D)-C(34A)-H(34E)	109.5
H(34D)-C(34A)-H(34F)	109.5
H(34E)-C(34A)-H(34F)	109.5

Symmetry transformations used to generate equivalent atoms:

Table S12.4. Anisotropic displacement parameters (Å^{2}x 10^3) for Shenvi295. The anisotropic displacement factor exponent takes the form: $-2\pi^2$[h2 a*2U11 + ... + 2 h k a* b* U12]

	U11	U22	U33	U23	U13	U12
Fe(1)	27(1)	17(1)	17(1)	1(1)	0(1)	2(1)
O(8)	18(1)	16(1)	21(1)	3(1)	4(1)	-1(1)
O(3)	22(1)	26(1)	23(1)	-5(1)	4(1)	-1(1)
O(5)	16(1)	32(1)	38(1)	-13(1)	11(1)	-9(1)
O(4)	23(1)	32(1)	24(1)	-2(1)	10(1)	-1(1)

O(7)	27(1)	33(1)	22(1)	-3(1)	6(1)	-10(1)
O(9)	28(1)	22(1)	26(1)	4(1)	13(1)	2(1)
O(11)	25(1)	37(1)	32(1)	9(1)	-8(1)	-5(1)
O(2)	25(1)	29(1)	56(1)	16(1)	20(1)	6(1)
O(6)	41(1)	40(1)	64(1)	-5(1)	29(1)	5(1)
O(1)	22(1)	30(1)	52(1)	11(1)	17(1)	-1(1)
C(14)	18(1)	17(1)	22(1)	-2(1)	4(1)	0(1)
C(10)	13(1)	30(1)	18(1)	0(1)	0(1)	-1(1)
O(10)	18(1)	29(1)	21(1)	-2(1)	4(1)	-4(1)
C(13)	15(1)	18(1)	17(1)	1(1)	0(1)	0(1)
C(20)	16(1)	18(1)	20(1)	2(1)	-1(1)	-1(1)
C(31)	28(1)	18(1)	28(1)	2(1)	-10(1)	-2(1)
C(32)	31(1)	31(1)	18(1)	0(1)	2(1)	-13(1)
C(21)	18(1)	20(1)	18(1)	1(1)	2(1)	1(1)
C(22)	21(1)	21(1)	18(1)	2(1)	-2(1)	0(1)
C(15)	14(1)	26(1)	25(1)	-3(1)	4(1)	-3(1)
C(28)	30(1)	27(1)	25(1)	2(1)	-5(1)	-7(1)
C(8)	15(1)	21(1)	18(1)	1(1)	1(1)	-2(1)
C(27)	41(2)	21(1)	25(1)	-1(1)	-7(1)	-5(1)
C(19)	17(1)	15(1)	24(1)	1(1)	2(1)	1(1)
C(7)	13(1)	23(1)	22(1)	0(1)	2(1)	0(1)
C(9)	15(1)	23(1)	18(1)	1(1)	1(1)	0(1)
C(24)	26(1)	29(1)	26(1)	-3(1)	5(1)	6(1)
C(23)	27(1)	22(1)	25(1)	1(1)	-2(1)	4(1)
C(25)	23(1)	24(1)	21(1)	2(1)	4(1)	0(1)
C(16)	15(1)	27(1)	22(1)	-6(1)	2(1)	-3(1)
C(17)	22(1)	46(2)	44(2)	-19(1)	13(1)	-6(1)
C(12)	18(1)	18(1)	21(1)	0(1)	1(1)	0(1)
C(11)	27(1)	37(1)	30(1)	-7(1)	13(1)	-1(1)
C(6)	19(1)	24(1)	28(1)	5(1)	6(1)	1(1)
C(29)	28(1)	28(1)	32(1)	-1(1)	-9(1)	1(1)
C(5)	22(1)	26(1)	35(1)	6(1)	10(1)	-2(1)
C(26)	48(2)	26(1)	21(1)	-3(1)	-3(1)	-7(1)
C(30)	43(2)	27(1)	23(1)	6(1)	-10(1)	-3(1)
C(1)	24(1)	36(1)	37(1)	5(2)	13(1)	0(1)
C(3)	19(1)	24(1)	38(1)	10(1)	10(1)	-1(1)
C(4)	34(1)	44(2)	34(1)	9(1)	10(1)	-4(1)
C(2)	24(1)	34(2)	64(2)	7(1)	20(1)	3(1)
C(18)	37(2)	58(2)	101(3)	-42(2)	45(2)	-24(2)
C(34)	27(2)	40(2)	26(2)	9(2)	-3(1)	4(2)
C(33)	17(2)	36(2)	43(2)	4(2)	4(2)	-5(2)
O(10A)	17(3)	22(3)	26(3)	2(2)	6(2)	-5(2)
C(33A)	12(4)	34(5)	45(6)	-2(5)	3(4)	-2(4)
C(34A)	29(4)	36(5)	21(4)	-8(4)	-4(3)	-4(4)

Table S12.5. Hydrogen coordinates (x 10^4) and isotropic displacement parameters ($Å^2$x 10^3) for Shenvi295.

	x	y	z	U(eq)
H(13)	4555	5935	3674	21
H(31A)	2403	6982	1576	30
H(31B)	3533	6369	1054	30
H(32)	2159	4553	938	32
H(32A)	2530	4854	706	32
H(22)	4557	1244	3189	24
H(15)	6695	6843	3933	26
H(28)	2019	357	1720	33
H(27)	3510	-743	696	36
H(19)	2643	5348	2754	22
H(7A)	1795	7649	4389	23
H(7B)	1605	6760	3493	23
H(9)	4683	7214	4964	23
H(24)	6662	1385	847	32
H(23)	5952	43	2165	30
H(25)	5698	3423	1043	27
H(16A)	5490	9143	3807	26
H(16B)	6307	8603	4742	26
H(12A)	2356	8296	2527	28
H(12B)	3791	8808	2693	28
H(12C)	2746	9190	3404	28
H(11A)	2011	8881	6779	46
H(11B)	2070	9828	5925	46
H(11C)	3227	9746	6769	46
H(6A)	2916	6161	5336	28
H(6B)	2757	5269	4434	28
H(29)	1767	2461	1065	36
H(5A)	411	5489	4482	33
H(5B)	1149	4684	5304	33
H(26)	4142	657	-603	38
H(30)	3074	2638	-372	38
H(1A)	-1644	6580	6945	38
H(1B)	-2473	6368	5918	38
H(4A)	859	6738	7249	55
H(4B)	2089	6801	6648	55
H(4C)	1586	5544	6996	55
H(2A)	-1698	8018	5359	47
H(2B)	-893	8243	6391	47
H(18A)	9745	8647	2818	94
H(18B)	8759	8846	1873	94
H(18C)	9742	7752	1922	94
H(34A)	1747	7483	-96	47
H(34B)	334	7138	164	47
H(34C)	709	6974	-918	47
H(33A)	-996	4901	1605	48
H(33B)	19	3937	1306	48

H(33C)	-599	4868	522	48
H(33D)	-359	4447	2053	46
H(33E)	-411	5396	1191	46
H(33F)	435	5662	2193	46
H(34D)	315	4583	-369	44
H(34E)	1602	4613	-906	44
H(34F)	491	5572	-1172	44

X-ray Structure of Et_2Zn Reformatsky Adduct **13** (CCDC2245372)

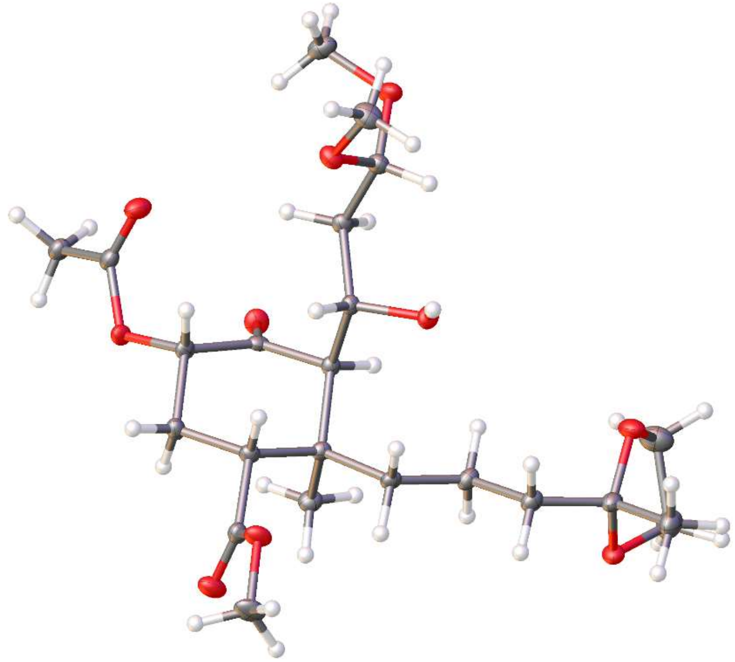

Experimental Summary

The single crystal X-ray diffraction studies were carried out on a Bruker X8 APEX II diffractometer equipped with Cu K_α radiation (λ = 1.54178). Crystals of the subject compound were used as received (grown from MTBE/$CHCl_3$)

. A 0.220 x 0.120 x 0.080 mm colorless crystal was mounted on a Cryoloop with Paratone oil.

Data were collected in a nitrogen gas stream at 100(2) K using ϕ and ω scans. Crystal-to-detector distance was 45 mm using exposure time 1.0, 2.0 and 4.0 seconds (depending on the 2 θ position) with a scan width of 1.50°. Data collection was 98.8 complete to 67.500° in θ. A total of 22709 reflections were collected. 4382 reflections were found to be symmetry independent, with a R_{int} of 0.0287. Indexing and unit cell refinement indicated a **Primitive Monoclinic** lattice. The space group was found to be ***P*2₁.** The data were integrated using the Bruker SAINT Software program and scaled using the SADABS software program. Solution by direct methods (SHELXT) produced a complete phasing model consistent with the proposed structure.

All nonhydrogen atoms were refined anisotropically by full-matrix least-squares (SHELXL-2014). All carbon bonded hydrogen atoms were placed using a riding model. Their positions were constrained relative to their parent atom using the appropriate HFIX command in SHELXL-2014.

Notes:

Absolute stereochemistry conclusive

Table S13.1. Crystal data and structure refinement for Shenvi296.

Report date	2023-02-28
Identification code	shenvi296
Empirical formula	C23 H38 O10
Molecular formula	C23 H38 O10
Formula weight	474.53
Temperature	100.00 K
Wavelength	1.54178 Å
Crystal system	Monoclinic
Space group	P 1 21 1
Unit cell dimensions	a = 12.7467(5) Å α= 90°.
	b = 6.4300(3) Å β= 105.8720(10)°.
	c = 15.4016(7) Å γ = 90°.
Volume	1214.21(9) Å3
Z	2
Density (calculated)	1.298 Mg/m^3
Absorption coefficient	0.845 mm^{-1}
F(000)	512
Crystal size	0.22 x 0.12 x 0.08 mm^3
Crystal color, habit	colorless trapezoid
Theta range for data collection	2.983 to 68.262°.
Index ranges	-15<=h<=15, -7<=k<=7, -18<=l<=18
Reflections collected	22709
Independent reflections	4382 [R(int) = 0.0287]
Completeness to theta = 67.500°	98.8 %
Absorption correction	Semi-empirical from equivalents
Max. and min. transmission	0.7533 and 0.6235
Refinement method	Full-matrix least-squares on F^2
Data / restraints / parameters	4382 / 1 / 306
Goodness-of-fit on F^2	1.031
Final R indices [I>2sigma(I)]	R1 = 0.0294, wR2 = 0.0763
R indices (all data)	R1 = 0.0294, wR2 = 0.0763
Absolute structure parameter	0.07(13)
Extinction coefficient	0.0023(6)
Largest diff. peak and hole	0.197 and -0.149 e.Å^{-3}

Table S13.2. Atomic coordinates (x 10^4) and equivalent isotropic displacement parameters ($Å^2$x 10^3) for Shenvi296. U(eq) is defined as one third of the trace of the orthogonalized U^{ij} tensor.

	x	y	z	U(eq)
O(1)	5780(1)	1452(2)	2456(1)	19(1)
O(2)	4703(1)	1861(2)	3679(1)	16(1)
O(3)	3307(1)	2463(2)	2449(1)	21(1)
O(4)	7415(1)	9133(2)	5096(1)	19(1)
O(5)	7973(1)	5959(2)	5635(1)	22(1)
O(6)	10936(1)	7276(2)	2299(1)	18(1)
O(7)	9528(1)	8182(3)	1094(1)	22(1)
O(8)	6483(1)	7885(2)	1650(1)	17(1)
O(9)	3699(1)	8519(2)	1010(1)	18(1)
O(10)	3693(1)	6413(2)	-232(1)	19(1)
C(1)	5849(1)	3157(3)	2792(1)	13(1)
C(2)	5221(2)	3700(3)	3476(1)	14(1)
C(3)	5971(2)	4546(3)	4350(1)	15(1)
C(4)	6706(2)	6275(3)	4152(1)	13(1)
C(5)	7381(2)	5551(3)	3497(1)	12(1)
C(6)	6547(2)	4895(3)	2587(1)	12(1)
C(7)	7439(2)	7055(3)	5042(1)	15(1)
C(8)	8091(2)	3638(3)	3867(1)	17(1)
C(9)	8126(2)	7350(3)	3378(1)	14(1)
C(10)	8828(2)	6974(3)	2729(1)	16(1)
C(11)	9410(2)	8979(3)	2610(1)	17(1)
C(12)	10137(2)	8844(3)	1978(1)	17(1)
C(13)	10659(2)	10932(4)	1898(2)	23(1)
C(14)	5809(2)	6616(3)	2032(1)	12(1)
C(15)	4901(2)	5649(3)	1268(1)	14(1)
C(16)	4327(2)	7278(3)	590(1)	16(1)
C(17)	3786(2)	1286(3)	3030(1)	15(1)
C(18)	3489(2)	-929(3)	3133(1)	17(1)
C(19)	8107(2)	10022(4)	5918(1)	24(1)
C(20)	10062(2)	6444(4)	837(2)	29(1)
C(21)	11174(2)	6417(4)	1520(1)	22(1)
C(22)	3319(2)	10379(3)	515(2)	24(1)
C(23)	2690(2)	5486(4)	-190(2)	23(1)

Table S13.3. Bond lengths [Å] and angles [°] for Shenvi296.

O(1)-C(1)	1.206(2)
O(2)-C(2)	1.431(2)
O(2)-C(17)	1.366(2)
O(3)-C(17)	1.202(3)
O(4)-C(7)	1.340(3)
O(4)-C(19)	1.448(2)
O(5)-C(7)	1.206(3)
O(6)-C(12)	1.422(2)
O(6)-C(21)	1.427(3)
O(7)-C(12)	1.435(3)
O(7)-C(20)	1.419(3)
O(8)-H(8)	0.8400
O(8)-C(14)	1.423(2)
O(9)-C(16)	1.407(2)
O(9)-C(22)	1.430(2)
O(10)-C(16)	1.416(2)
O(10)-C(23)	1.428(3)
C(1)-C(2)	1.526(2)
C(1)-C(6)	1.515(3)
C(2)-H(2)	1.0000
C(2)-C(3)	1.523(3)
C(3)-H(3A)	0.9900
C(3)-H(3B)	0.9900
C(3)-C(4)	1.537(2)
C(4)-H(4)	1.0000
C(4)-C(5)	1.565(2)
C(4)-C(7)	1.517(3)
C(5)-C(6)	1.568(2)
C(5)-C(8)	1.541(3)
C(5)-C(9)	1.539(2)
C(6)-H(6)	1.0000
C(6)-C(14)	1.549(3)
C(8)-H(8A)	0.9800
C(8)-H(8B)	0.9800
C(8)-H(8C)	0.9800
C(9)-H(9A)	0.9900
C(9)-H(9B)	0.9900
C(9)-C(10)	1.533(2)
C(10)-H(10A)	0.9900
C(10)-H(10B)	0.9900
C(10)-C(11)	1.523(3)
C(11)-H(11A)	0.9900
C(11)-H(11B)	0.9900
C(11)-C(12)	1.520(3)
C(12)-C(13)	1.518(3)
C(13)-H(13A)	0.9800
C(13)-H(13B)	0.9800
C(13)-H(13C)	0.9800
C(14)-H(14)	1.0000
C(14)-C(15)	1.538(3)
C(15)-H(15A)	0.9900
C(15)-H(15B)	0.9900
C(15)-C(16)	1.519(3)
C(16)-H(16)	1.0000
C(17)-C(18)	1.493(3)
C(18)-H(18A)	0.9800
C(18)-H(18B)	0.9800
C(18)-H(18C)	0.9800
C(19)-H(19A)	0.9800
C(19)-H(19B)	0.9800
C(19)-H(19C)	0.9800
C(20)-H(20A)	0.9900
C(20)-H(20B)	0.9900
C(20)-C(21)	1.517(3)
C(21)-H(21A)	0.9900
C(21)-H(21B)	0.9900
C(22)-H(22A)	0.9800
C(22)-H(22B)	0.9800
C(22)-H(22C)	0.9800
C(23)-H(23A)	0.9800
C(23)-H(23B)	0.9800
C(23)-H(23C)	0.9800

C(17)-O(2)-C(2)	114.67(14)
C(7)-O(4)-C(19)	115.41(16)
C(12)-O(6)-C(21)	106.16(15)
C(20)-O(7)-C(12)	108.74(15)
C(14)-O(8)-H(8)	109.5
C(16)-O(9)-C(22)	112.39(15)
C(16)-O(10)-C(23)	115.05(15)
O(1)-C(1)-C(2)	120.88(17)
O(1)-C(1)-C(6)	124.03(17)
C(6)-C(1)-C(2)	115.09(16)
O(2)-C(2)-C(1)	108.85(15)
O(2)-C(2)-H(2)	109.2
O(2)-C(2)-C(3)	108.47(14)
C(1)-C(2)-H(2)	109.2
C(3)-C(2)-C(1)	111.76(15)
C(3)-C(2)-H(2)	109.2
C(2)-C(3)-H(3A)	109.5
C(2)-C(3)-H(3B)	109.5
C(2)-C(3)-C(4)	110.59(15)

H(3A)-C(3)-H(3B)	108.1
C(4)-C(3)-H(3A)	109.5
C(4)-C(3)-H(3B)	109.5
C(3)-C(4)-H(4)	107.9
C(3)-C(4)-C(5)	112.64(15)
C(5)-C(4)-H(4)	107.9
C(7)-C(4)-C(3)	108.51(15)
C(7)-C(4)-H(4)	107.9
C(7)-C(4)-C(5)	111.72(15)
C(4)-C(5)-C(6)	107.36(14)
C(8)-C(5)-C(4)	111.70(15)
C(8)-C(5)-C(6)	107.62(15)
C(9)-C(5)-C(4)	108.55(14)
C(9)-C(5)-C(6)	112.70(14)
C(9)-C(5)-C(8)	108.95(15)
C(1)-C(6)-C(5)	107.73(14)
C(1)-C(6)-H(6)	107.1
C(1)-C(6)-C(14)	109.88(15)
C(5)-C(6)-H(6)	107.1
C(14)-C(6)-C(5)	117.54(15)
C(14)-C(6)-H(6)	107.1
O(4)-C(7)-C(4)	111.53(16)
O(5)-C(7)-O(4)	123.62(18)
O(5)-C(7)-C(4)	124.85(18)
C(5)-C(8)-H(8A)	109.5
C(5)-C(8)-H(8B)	109.5
C(5)-C(8)-H(8C)	109.5
H(8A)-C(8)-H(8B)	109.5
H(8A)-C(8)-H(8C)	109.5
H(8B)-C(8)-H(8C)	109.5
C(5)-C(9)-H(9A)	108.1
C(5)-C(9)-H(9B)	108.1
H(9A)-C(9)-H(9B)	107.3
C(10)-C(9)-C(5)	116.60(16)
C(10)-C(9)-H(9A)	108.1
C(10)-C(9)-H(9B)	108.1
C(9)-C(10)-H(10A)	109.8
C(9)-C(10)-H(10B)	109.8
H(10A)-C(10)-H(10B)	108.2
C(11)-C(10)-C(9)	109.57(16)
C(11)-C(10)-H(10A)	109.8
C(11)-C(10)-H(10B)	109.8
C(10)-C(11)-H(11A)	108.4
C(10)-C(11)-H(11B)	108.4
H(11A)-C(11)-H(11B)	107.5
C(12)-C(11)-C(10)	115.52(16)
C(12)-C(11)-H(11A)	108.4
C(12)-C(11)-H(11B)	108.4
O(6)-C(12)-O(7)	105.69(16)
O(6)-C(12)-C(11)	108.77(16)
O(6)-C(12)-C(13)	111.54(15)
O(7)-C(12)-C(11)	110.96(15)
O(7)-C(12)-C(13)	108.73(16)
C(13)-C(12)-C(11)	111.04(17)
C(12)-C(13)-H(13A)	109.5
C(12)-C(13)-H(13B)	109.5
C(12)-C(13)-H(13C)	109.5
H(13A)-C(13)-H(13B)	109.5
H(13A)-C(13)-H(13C)	109.5
H(13B)-C(13)-H(13C)	109.5
O(8)-C(14)-C(6)	107.06(14)
O(8)-C(14)-H(14)	110.0
O(8)-C(14)-C(15)	109.23(15)
C(6)-C(14)-H(14)	110.0
C(15)-C(14)-C(6)	110.44(15)
C(15)-C(14)-H(14)	110.0
C(14)-C(15)-H(15A)	109.3
C(14)-C(15)-H(15B)	109.3
H(15A)-C(15)-H(15B)	108.0
C(16)-C(15)-C(14)	111.53(16)
C(16)-C(15)-H(15A)	109.3
C(16)-C(15)-H(15B)	109.3
O(9)-C(16)-O(10)	112.12(15)
O(9)-C(16)-C(15)	107.59(15)
O(9)-C(16)-H(16)	107.9
O(10)-C(16)-C(15)	113.16(16)
O(10)-C(16)-H(16)	107.9
C(15)-C(16)-H(16)	107.9
O(2)-C(17)-C(18)	111.81(16)
O(3)-C(17)-O(2)	122.21(18)
O(3)-C(17)-C(18)	125.98(18)
C(17)-C(18)-H(18A)	109.5
C(17)-C(18)-H(18B)	109.5
C(17)-C(18)-H(18C)	109.5
H(18A)-C(18)-H(18B)	109.5
H(18A)-C(18)-H(18C)	109.5
H(18B)-C(18)-H(18C)	109.5
O(4)-C(19)-H(19A)	109.5
O(4)-C(19)-H(19B)	109.5
O(4)-C(19)-H(19C)	109.5
H(19A)-C(19)-H(19B)	109.5
H(19A)-C(19)-H(19C)	109.5
H(19B)-C(19)-H(19C)	109.5
O(7)-C(20)-H(20A)	110.9
O(7)-C(20)-H(20B)	110.9
O(7)-C(20)-C(21)	104.14(17)

H(20A)-C(20)-H(20B)	108.9	O(9)-C(22)-H(22C)	109.5
C(21)-C(20)-H(20A)	110.9	H(22A)-C(22)-H(22B)	109.5
C(21)-C(20)-H(20B)	110.9	H(22A)-C(22)-H(22C)	109.5
O(6)-C(21)-C(20)	101.85(16)	H(22B)-C(22)-H(22C)	109.5
O(6)-C(21)-H(21A)	111.4	O(10)-C(23)-H(23A)	109.5
O(6)-C(21)-H(21B)	111.4	O(10)-C(23)-H(23B)	109.5
C(20)-C(21)-H(21A)	111.4	O(10)-C(23)-H(23C)	109.5
C(20)-C(21)-H(21B)	111.4	H(23A)-C(23)-H(23B)	109.5
H(21A)-C(21)-H(21B)	109.3	H(23A)-C(23)-H(23C)	109.5
O(9)-C(22)-H(22A)	109.5	H(23B)-C(23)-H(23C)	109.5
O(9)-C(22)-H(22B)	109.5		

Table S13.4. Anisotropic displacement parameters ($Å^2$x 10^3) for Shenvi296. The anisotropic displacement factor exponent takes the form: $-2\pi^2$[h2 a*2U11 + ... + 2 h k a* b* U12]

	U11	U22	U33	U23	U13	U12
O(1)	24(1)	11(1)	20(1)	-4(1)	7(1)	-4(1)
O(2)	17(1)	15(1)	15(1)	1(1)	3(1)	-7(1)
O(3)	16(1)	21(1)	24(1)	3(1)	0(1)	-2(1)
O(4)	24(1)	15(1)	14(1)	-4(1)	-1(1)	-4(1)
O(5)	27(1)	19(1)	15(1)	0(1)	-2(1)	-4(1)
O(6)	14(1)	22(1)	17(1)	4(1)	3(1)	2(1)
O(7)	18(1)	29(1)	16(1)	0(1)	0(1)	2(1)
O(8)	17(1)	12(1)	21(1)	4(1)	5(1)	-2(1)
O(9)	22(1)	15(1)	15(1)	1(1)	5(1)	1(1)
O(10)	17(1)	26(1)	14(1)	-4(1)	2(1)	-1(1)
C(1)	13(1)	11(1)	11(1)	2(1)	-1(1)	0(1)
C(2)	15(1)	11(1)	14(1)	0(1)	3(1)	-4(1)
C(3)	18(1)	16(1)	13(1)	-1(1)	5(1)	-4(1)
C(4)	14(1)	12(1)	13(1)	-2(1)	2(1)	-2(1)
C(5)	13(1)	10(1)	14(1)	-1(1)	2(1)	-1(1)
C(6)	14(1)	10(1)	13(1)	-2(1)	4(1)	-2(1)
C(7)	16(1)	15(1)	14(1)	-1(1)	5(1)	-4(1)
C(8)	16(1)	14(1)	20(1)	1(1)	3(1)	1(1)
C(9)	12(1)	13(1)	15(1)	-2(1)	2(1)	-3(1)
C(10)	13(1)	15(1)	21(1)	0(1)	5(1)	-2(1)
C(11)	15(1)	15(1)	20(1)	0(1)	4(1)	-3(1)
C(12)	13(1)	20(1)	17(1)	4(1)	2(1)	-1(1)
C(13)	20(1)	23(1)	27(1)	6(1)	7(1)	-3(1)
C(14)	14(1)	10(1)	13(1)	0(1)	4(1)	-2(1)
C(15)	16(1)	13(1)	13(1)	-2(1)	3(1)	-3(1)
C(16)	16(1)	17(1)	13(1)	-1(1)	3(1)	-1(1)
C(17)	13(1)	18(1)	16(1)	-4(1)	6(1)	-3(1)
C(18)	16(1)	18(1)	18(1)	-2(1)	4(1)	-5(1)
C(19)	31(1)	19(1)	17(1)	-6(1)	-2(1)	-7(1)
C(20)	32(1)	26(1)	25(1)	-4(1)	1(1)	3(1)
C(21)	22(1)	21(1)	25(1)	1(1)	9(1)	1(1)
C(22)	30(1)	17(1)	23(1)	2(1)	4(1)	4(1)
C(23)	17(1)	24(1)	25(1)	-7(1)	2(1)	-2(1)

Table S13.5. Hydrogen coordinates (x 10^4) and isotropic displacement parameters ($Å^2$x 10^3) for Shenvi296.

	x	y	z	U(eq)
H(8)	6292	9134	1657	25
H(2)	4653	4764	3208	16
H(3A)	6430	3405	4685	19
H(3B)	5528	5105	4734	19
H(4)	6227	7452	3858	16
H(6)	6979	4295	2194	15
H(8A)	7628	2514	3983	26
H(8B)	8634	4011	4430	26
H(8C)	8464	3173	3423	26
H(9A)	7663	8585	3165	17
H(9B)	8618	7695	3979	17
H(10A)	9372	5873	2973	19
H(10B)	8362	6503	2137	19
H(11A)	8853	10066	2381	20
H(11B)	9860	9436	3210	20
H(13A)	11077	11402	2498	35
H(13B)	10089	11951	1635	35
H(13C)	11148	10790	1509	35
H(14)	5483	7469	2434	15
H(15A)	5220	4576	955	17
H(15B)	4362	4960	1529	17
H(16)	4898	8184	450	19
H(18A)	3588	-1745	2624	26
H(18B)	2725	-1007	3145	26
H(18C)	3958	-1488	3698	26
H(19A)	8105	11541	5865	36
H(19B)	8853	9501	6017	36
H(19C)	7831	9626	6430	36
H(20A)	10132	6617	217	35
H(20B)	9658	5143	867	35
H(21A)	11462	4984	1635	26
H(21B)	11704	7287	1319	26
H(22A)	2909	10018	-103	36
H(22B)	3943	11253	500	36
H(22C)	2843	11139	808	36
H(23A)	2453	4474	-680	34
H(23B)	2132	6567	-252	34
H(23C)	2795	4780	392	34

Table S13.6. Hydrogen bonds for Shenvi296 [Å and °].

D-H...A	d(D-H)	d(H...A)	d(D...A)	<(DHA)
O(8)-H(8)...O(1)#1	0.84	2.14	2.8657(19)	143.7

Symmetry transformations used to generate equivalent atoms:
#1 x,y+1,z

X-ray Structure of Enone **17d** (CCDC2250953)

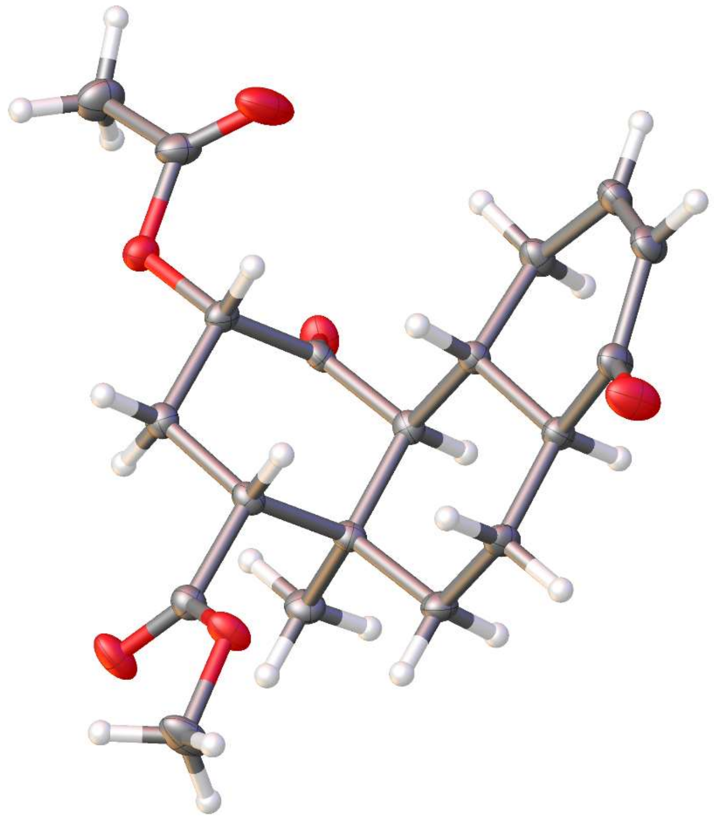

Experimental Summary

The single crystal X-ray diffraction studies were carried out on a Bruker SMART Pt 135 diffractometer equipped with Cu K_{α} radiation (λ = 1.54178). Crystals of the subject compound were used as received (grown from MTBE/Pentane). A 0.200 x 0.180 x 0.150 mm colorless crystal was mounted on a Cryoloop with Paratone oil.

Data were collected in a nitrogen gas stream at 100(2) K using ϕ and ω scans. Crystal-to-detector distance was 45 mm using exposure time 1.0, 2.0 and 4.0 second (depending on the 2 θ position) with a scan width of 1.40°. Data collection was 100.0% complete to 67.679° in θ. A total of 19914 reflections were collected. 3483 reflections were found to be symmetry independent, with a R_{int} of 0.0473. Indexing and unit cell refinement indicated an **Orthorhombic** lattice. The space group was found to be ***P*** $\mathbf{2_12_12_1}$**.**
The data were integrated using the Bruker SAINT Software program and scaled using the SADABS software program. Solution by direct methods (SHELXT) produced a complete phasing model consistent with the proposed structure.

All nonhydrogen atoms were refined anisotropically by full-matrix least-squares (SHELXL-2014). All carbon bonded hydrogen atoms were placed using a riding model. Their positions were constrained relative to their parent atom using the appropriate HFIX command in SHELXL-2014.

Notes: Excellent data and refinement.
Absolute structure parameter 0.03(7) conclusive

Table S14.1. Crystal data and structure refinement for Shenvi299.

Report date	2023-03-21
Identification code	Shenvi299

Empirical formula	C19 H24 O6
Molecular formula	C19 H24 O6
Formula weight	348.38
Temperature	100.15 K
Wavelength	1.54178 Å
Crystal system	Orthorhombic
Space group	$P2_12_12_1$
Unit cell dimensions	a = 9.6309(7) Å α = 90°.
	b = 13.0401(10) Å β = 90°.
	c = 14.5282(10) Å γ = 90°.
Volume	1824.6(2) Å3
Z	4
Density (calculated)	1.268 Mg/m^3
Absorption coefficient	0.778 mm^{-1}
F(000)	744
Crystal size	0.2 x 0.18 x 0.15 mm^3
Crystal color, habit	colorless irregular
Theta range for data collection	4.556 to 70.543°.
Index ranges	-11<=h<=11, -15<=k<=15, -17<=l<=17
Reflections collected	19914
Independent reflections	3483 [R(int) = 0.0473]
Completeness to theta = 67.679°	100.0 %
Absorption correction	Semi-empirical from equivalents
Max. and min. transmission	0.7533 and 0.6546
Refinement method	Full-matrix least-squares on F^2
Data / restraints / parameters	3483 / 0 / 229
Goodness-of-fit on F^2	1.045
Final R indices [I>2sigma(I)]	R1 = 0.0307, wR2 = 0.0817
R indices (all data)	R1 = 0.0309, wR2 = 0.0820
Absolute structure parameter	0.03(7)
Largest diff. peak and hole	0.301 and -0.149 e.Å^{-3}

Table S14.2. Atomic coordinates (x 10^4) and equivalent isotropic displacement parameters ($Å^2$x 10^3) for Shenvi299. U(eq) is defined as one third of the trace of the orthogonalized U^{ij} tensor.

	x	y	z	U(eq)
O(1)	10030(1)	7065(1)	7366(1)	33(1)
O(2)	3703(1)	4568(1)	6480(1)	24(1)
O(3)	4193(1)	4532(1)	4677(1)	20(1)
O(4)	5602(2)	3289(1)	5214(1)	41(1)
O(5)	3739(1)	8477(1)	4929(1)	28(1)
O(6)	5961(1)	8876(1)	5230(1)	23(1)
C(1)	9195(2)	6365(1)	7432(1)	21(1)
C(2)	9652(2)	5281(1)	7491(1)	23(1)
C(3)	8758(2)	4504(1)	7427(1)	22(1)
C(4)	7227(2)	4657(1)	7262(1)	20(1)
C(5)	6904(2)	5727(1)	6884(1)	15(1)
C(6)	5319(2)	5914(1)	6850(1)	16(1)
C(7)	4593(2)	5156(1)	6224(1)	16(1)
C(8)	4977(2)	5262(1)	5206(1)	17(1)
C(9)	4606(2)	6343(1)	4876(1)	18(1)
C(10)	5292(2)	7156(1)	5499(1)	16(1)
C(11)	4901(2)	7017(1)	6544(1)	15(1)
C(12)	5673(2)	7785(1)	7164(1)	18(1)
C(13)	7252(2)	7642(1)	7177(1)	18(1)
C(14)	7635(2)	6552(1)	7469(1)	17(1)
C(15)	4886(2)	8221(1)	5180(1)	18(1)
C(16)	3326(2)	7143(1)	6694(1)	21(1)
C(17)	4574(2)	3536(1)	4802(1)	25(1)
C(18)	3545(2)	2819(2)	4382(2)	31(1)
C(19)	5656(2)	9941(1)	5014(2)	29(1)

Table S14.3. Bond lengths [Å] and angles [°] for Shenvi299.

O(1)-C(1)	1.221(2)
O(2)-C(7)	1.210(2)
O(3)-C(8)	1.438(2)
O(3)-C(17)	1.363(2)
O(4)-C(17)	1.201(3)
O(5)-C(15)	1.211(2)
O(6)-C(15)	1.345(2)
O(6)-C(19)	1.454(2)
C(1)-C(2)	1.483(2)
C(1)-C(14)	1.522(2)
C(2)-H(2)	0.9500
C(2)-C(3)	1.333(3)
C(3)-H(3)	0.9500
C(3)-C(4)	1.507(2)
C(4)-H(4A)	0.9900
C(4)-H(4B)	0.9900
C(4)-C(5)	1.532(2)
C(5)-H(5)	1.0000
C(5)-C(6)	1.546(2)
C(5)-C(14)	1.542(2)
C(6)-H(6)	1.0000
C(6)-C(7)	1.514(2)
C(6)-C(11)	1.558(2)
C(7)-C(8)	1.531(2)
C(8)-H(8)	1.0000
C(8)-C(9)	1.531(2)
C(9)-H(9A)	0.9900
C(9)-H(9B)	0.9900
C(9)-C(10)	1.542(2)
C(10)-H(10)	1.0000
C(10)-C(11)	1.574(2)
C(10)-C(15)	1.515(2)
C(11)-C(12)	1.539(2)
C(11)-C(16)	1.542(2)
C(12)-H(12A)	0.9900
C(12)-H(12B)	0.9900
C(12)-C(13)	1.533(2)
C(13)-H(13A)	0.9900
C(13)-H(13B)	0.9900
C(13)-C(14)	1.530(2)
C(14)-H(14)	1.0000
C(16)-H(16A)	0.9800
C(16)-H(16B)	0.9800
C(16)-H(16C)	0.9800
C(17)-C(18)	1.492(3)
C(18)-H(18A)	0.9800
C(18)-H(18B)	0.9800
C(18)-H(18C)	0.9800
C(19)-H(19A)	0.9800
C(19)-H(19B)	0.9800
C(19)-H(19C)	0.9800
C(17)-O(3)-C(8)	114.71(13)
C(15)-O(6)-C(19)	116.07(13)
O(1)-C(1)-C(2)	121.42(16)
O(1)-C(1)-C(14)	122.23(16)
C(2)-C(1)-C(14)	116.35(15)
C(1)-C(2)-H(2)	119.0
C(3)-C(2)-C(1)	121.91(16)
C(3)-C(2)-H(2)	119.0
C(2)-C(3)-H(3)	118.6
C(2)-C(3)-C(4)	122.84(16)
C(4)-C(3)-H(3)	118.6
C(3)-C(4)-H(4A)	109.2
C(3)-C(4)-H(4B)	109.2
C(3)-C(4)-C(5)	112.12(14)
H(4A)-C(4)-H(4B)	107.9
C(5)-C(4)-H(4A)	109.2
C(5)-C(4)-H(4B)	109.2
C(4)-C(5)-H(5)	108.2
C(4)-C(5)-C(6)	110.83(13)
C(4)-C(5)-C(14)	110.16(13)
C(6)-C(5)-H(5)	108.2
C(14)-C(5)-H(5)	108.2
C(14)-C(5)-C(6)	111.02(13)
C(5)-C(6)-H(6)	107.5
C(5)-C(6)-C(11)	114.18(13)
C(7)-C(6)-C(5)	111.85(13)
C(7)-C(6)-H(6)	107.5
C(7)-C(6)-C(11)	108.15(13)
C(11)-C(6)-H(6)	107.5
O(2)-C(7)-C(6)	123.85(15)
O(2)-C(7)-C(8)	121.72(15)
C(6)-C(7)-C(8)	114.19(13)
O(3)-C(8)-C(7)	109.30(13)
O(3)-C(8)-H(8)	109.9
O(3)-C(8)-C(9)	108.63(13)
C(7)-C(8)-H(8)	109.9
C(7)-C(8)-C(9)	109.20(13)
C(9)-C(8)-H(8)	109.9
C(8)-C(9)-H(9A)	109.6
C(8)-C(9)-H(9B)	109.6

C(8)-C(9)-C(10)	110.43(13)	C(1)-C(14)-C(13)	112.15(14)
H(9A)-C(9)-H(9B)	108.1	C(1)-C(14)-H(14)	107.7
C(10)-C(9)-H(9A)	109.6	C(5)-C(14)-H(14)	107.7
C(10)-C(9)-H(9B)	109.6	C(13)-C(14)-C(5)	112.64(13)
C(9)-C(10)-H(10)	108.2	C(13)-C(14)-H(14)	107.7
C(9)-C(10)-C(11)	112.60(13)	O(5)-C(15)-O(6)	122.96(15)
C(11)-C(10)-H(10)	108.2	O(5)-C(15)-C(10)	125.50(15)
C(15)-C(10)-C(9)	109.86(13)	O(6)-C(15)-C(10)	111.53(14)
C(15)-C(10)-H(10)	108.2	C(11)-C(16)-H(16A)	109.5
C(15)-C(10)-C(11)	109.79(13)	C(11)-C(16)-H(16B)	109.5
C(6)-C(11)-C(10)	108.66(13)	C(11)-C(16)-H(16C)	109.5
C(12)-C(11)-C(6)	107.96(13)	H(16A)-C(16)-H(16B)	109.5
C(12)-C(11)-C(10)	112.01(13)	H(16A)-C(16)-H(16C)	109.5
C(12)-C(11)-C(16)	108.80(13)	H(16B)-C(16)-H(16C)	109.5
C(16)-C(11)-C(6)	108.20(13)	O(3)-C(17)-C(18)	111.29(16)
C(16)-C(11)-C(10)	111.08(13)	O(4)-C(17)-O(3)	122.98(17)
C(11)-C(12)-H(12A)	108.7	O(4)-C(17)-C(18)	125.72(18)
C(11)-C(12)-H(12B)	108.7	C(17)-C(18)-H(18A)	109.5
H(12A)-C(12)-H(12B)	107.6	C(17)-C(18)-H(18B)	109.5
C(13)-C(12)-C(11)	114.03(14)	C(17)-C(18)-H(18C)	109.5
C(13)-C(12)-H(12A)	108.7	H(18A)-C(18)-H(18B)	109.5
C(13)-C(12)-H(12B)	108.7	H(18A)-C(18)-H(18C)	109.5
C(12)-C(13)-H(13A)	109.5	H(18B)-C(18)-H(18C)	109.5
C(12)-C(13)-H(13B)	109.5	O(6)-C(19)-H(19A)	109.5
H(13A)-C(13)-H(13B)	108.1	O(6)-C(19)-H(19B)	109.5
C(14)-C(13)-C(12)	110.81(14)	O(6)-C(19)-H(19C)	109.5
C(14)-C(13)-H(13A)	109.5	H(19A)-C(19)-H(19B)	109.5
C(14)-C(13)-H(13B)	109.5	H(19A)-C(19)-H(19C)	109.5
C(1)-C(14)-C(5)	108.63(13)	H(19B)-C(19)-H(19C)	109.5

X-ray Structure of Enone **17b (**CCDC2217612)

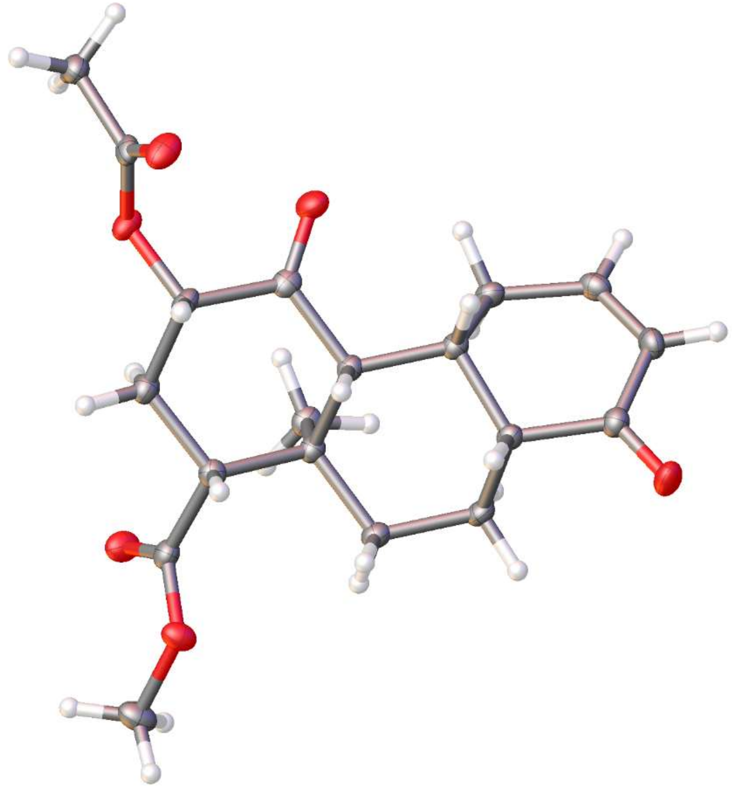

Experimental Summary

The single crystal X-ray diffraction studies were carried out on a Bruker SMART Pt135 CCD diffractometer equipped with Cu K_α radiation (λ = 1.54178 Å).

Crystals of the subject compound were used as received. A 0.3 x 0.12 x 0.04 mm piece of a crystal was mounted on a Cryoloop with Paratone oil. Data were collected in a nitrogen gas stream at 100(2) K using ϕ and ω scans. Crystal-to-detector distance was 45 mm and exposure time was 5, 10, 15, or 20 seconds depending on the 2θ range per frame using a scan width of 1.25°. Data collection was 100 % complete to 67.679° in θ. A total of 14263 reflections were collected covering the indices, −10<=h<=10, −7<=k<=7, −17<=l<=18. 3223 reflections were found to be symmetry independent, with a R_{int} of 0.0391. Indexing and unit cell refinement indicated a **P**rimitive, **Monoclinic** lattice. The space group was found to be $\boldsymbol{P2_1}$. The data were integrated using the Bruker SAINT Software program and scaled using the SADABS software program. Solution by direct methods (SHELXT) produced a complete phasing model consistent with the proposed structure.

All nonhydrogen atoms were refined anisotropically by full-matrix least-squares (SHELXL-2014). All carbon bonded hydrogen atoms were placed using a riding model. Their positions were constrained relative to their parent atom using the appropriate HFIX command in SHELXL-2014. Crystallographic data are summarized in Table S15.1.

Notes: Absolute stereochemistry was conclusively assigned (Absolute Structure Parameter = 0.09(9)). There is one copy of the compound in the asymmetric unit. The chemical formula for the compound is: $C_{19}H_{24}O_6$

Table S15.1. Crystal data and structure refinement for Shenvi289.

Identification code	shenvi289_0m_a
Empirical formula	C19 H24 O6
Formula weight	348.38
Temperature	100.15 K
Wavelength	1.54178 Å
Crystal system	Monoclinic
Space group	P 1 21 1
Unit cell dimensions	a = 8.8051(3) Å α= 90°.
	b = 6.5374(3) Å β= 96.846(2)°.
	c = 14.8753(6) Å γ = 90°.
Volume	850.15(6) Å3
Z	2
Density (calculated)	1.361 Mg/m^3
Absorption coefficient	0.835 mm^{-1}
F(000)	372
Crystal size	0.3 x 0.12 x 0.04 mm^3
Theta range for data collection	2.992 to 70.144°.
Index ranges	-10<=h<=10, -7<=k<=7, -17<=l<=18
Reflections collected	14263
Independent reflections	3223 [R(int) = 0.0391]
Completeness to theta = 67.679°	100.0 %
Absorption correction	Semi-empirical from equivalents
Max. and min. transmission	0.5220 and 0.4129
Refinement method	Full-matrix least-squares on F^2
Data / restraints / parameters	3223 / 1 / 229
Goodness-of-fit on F^2	1.061
Final R indices [I>2sigma(I)]	R1 = 0.0284, wR2 = 0.0694
R indices (all data)	R1 = 0.0300, wR2 = 0.0704
Absolute structure parameter	0.09(9)
Largest diff. peak and hole	0.249 and -0.147 e.Å^{-3}

Table S15.2. Atomic coordinates (x 10^4) and equivalent isotropic displacement parameters ($Å^2$x 10^3) for Shenvi289. U(eq) is defined as one third of the trace of the orthogonalized U^{ij} tensor.

	x	y	z	U(eq)
O(1)	1644(2)	8463(2)	593(1)	27(1)
O(2)	10731(2)	6752(2)	2739(1)	24(1)
O(3)	9578(2)	9786(2)	2846(1)	22(1)
O(4)	7745(2)	2137(2)	4576(1)	20(1)
O(5)	5785(2)	2217(3)	5422(1)	27(1)
O(6)	5365(2)	1858(2)	3279(1)	24(1)
C(1)	4056(2)	3170(3)	1407(1)	19(1)
C(2)	2638(2)	3202(3)	739(1)	21(1)
C(3)	1826(2)	4887(4)	510(1)	21(1)
C(4)	2321(2)	6895(3)	857(1)	19(1)
C(5)	3766(2)	7022(3)	1531(1)	16(1)
C(6)	5140(2)	7668(3)	1044(1)	18(1)
C(7)	6565(2)	8012(3)	1728(1)	17(1)
C(8)	7021(2)	6128(3)	2322(1)	16(1)
C(9)	7762(2)	4490(3)	1767(1)	19(1)
C(10)	8158(2)	6829(3)	3161(1)	17(1)
C(11)	9625(2)	7738(3)	2891(1)	18(1)
C(12)	10943(2)	10743(4)	2582(2)	26(1)
C(13)	8597(2)	5042(3)	3816(1)	19(1)
C(14)	7205(2)	4007(3)	4133(1)	18(1)
C(15)	6858(2)	1335(3)	5173(1)	18(1)
C(16)	7411(2)	-735(3)	5491(1)	21(1)
C(17)	5971(2)	3524(3)	3351(1)	17(1)
C(18)	5562(2)	5330(3)	2727(1)	15(1)
C(19)	4108(2)	5014(3)	2054(1)	16(1)

Table S15.3. Bond lengths [Å] and angles [°] for Shenvi289.

O(1)-C(4)	1.227(3)
O(2)-C(11)	1.210(3)
O(3)-C(11)	1.341(3)
O(3)-C(12)	1.450(2)
O(4)-C(14)	1.442(2)
O(4)-C(15)	1.357(2)
O(5)-C(15)	1.202(2)
O(6)-C(17)	1.212(3)
C(1)-H(1A)	0.9900
C(1)-H(1B)	0.9900
C(1)-C(2)	1.501(3)
C(1)-C(19)	1.539(3)
C(2)-H(2)	0.9500
C(2)-C(3)	1.336(3)
C(3)-H(3)	0.9500
C(3)-C(4)	1.458(3)
C(4)-C(5)	1.525(3)
C(5)-H(5)	1.0000
C(5)-C(6)	1.541(3)
C(5)-C(19)	1.537(3)
C(6)-H(6A)	0.9900
C(6)-H(6B)	0.9900
C(6)-C(7)	1.535(3)
C(7)-H(7A)	0.9900
C(7)-H(7B)	0.9900
C(7)-C(8)	1.541(3)
C(8)-C(9)	1.543(3)
C(8)-C(10)	1.572(3)
C(8)-C(18)	1.572(2)
C(9)-H(9A)	0.9800
C(9)-H(9B)	0.9800
C(9)-H(9C)	0.9800
C(10)-H(10)	1.0000
C(10)-C(11)	1.519(3)
C(10)-C(13)	1.541(3)
C(12)-H(12A)	0.9800
C(12)-H(12B)	0.9800
C(12)-H(12C)	0.9800
C(13)-H(13A)	0.9900
C(13)-H(13B)	0.9900
C(13)-C(14)	1.523(3)
C(14)-H(14)	1.0000
C(14)-C(17)	1.528(3)
C(15)-C(16)	1.496(3)
C(16)-H(16A)	0.9800
C(16)-H(16B)	0.9800
C(16)-H(16C)	0.9800
C(17)-C(18)	1.518(3)
C(18)-H(18)	1.0000
C(18)-C(19)	1.543(3)
C(19)-H(19)	1.0000

C(11)-O(3)-C(12)	114.97(16)
C(15)-O(4)-C(14)	116.25(15)
H(1A)-C(1)-H(1B)	108.0
C(2)-C(1)-H(1A)	109.4
C(2)-C(1)-H(1B)	109.4
C(2)-C(1)-C(19)	111.33(17)
C(19)-C(1)-H(1A)	109.4
C(19)-C(1)-H(1B)	109.4
C(1)-C(2)-H(2)	118.0
C(3)-C(2)-C(1)	123.91(19)
C(3)-C(2)-H(2)	118.0
C(2)-C(3)-H(3)	119.2
C(2)-C(3)-C(4)	121.61(18)
C(4)-C(3)-H(3)	119.2
O(1)-C(4)-C(3)	121.69(17)
O(1)-C(4)-C(5)	120.05(19)
C(3)-C(4)-C(5)	118.19(18)
C(4)-C(5)-H(5)	107.7
C(4)-C(5)-C(6)	110.23(14)
C(4)-C(5)-C(19)	112.56(16)
C(6)-C(5)-H(5)	107.7
C(19)-C(5)-H(5)	107.7
C(19)-C(5)-C(6)	110.77(15)
C(5)-C(6)-H(6A)	109.5
C(5)-C(6)-H(6B)	109.5
H(6A)-C(6)-H(6B)	108.1
C(7)-C(6)-C(5)	110.74(14)
C(7)-C(6)-H(6A)	109.5
C(7)-C(6)-H(6B)	109.5
C(6)-C(7)-H(7A)	108.9
C(6)-C(7)-H(7B)	108.9
C(6)-C(7)-C(8)	113.55(16)
H(7A)-C(7)-H(7B)	107.7
C(8)-C(7)-H(7A)	108.9
C(8)-C(7)-H(7B)	108.9
C(7)-C(8)-C(9)	110.23(15)
C(7)-C(8)-C(10)	108.67(16)
C(7)-C(8)-C(18)	108.46(15)

C(9)-C(8)-C(10)	110.90(15)	O(4)-C(14)-C(13)	106.50(16)
C(9)-C(8)-C(18)	113.06(16)	O(4)-C(14)-H(14)	109.3
C(18)-C(8)-C(10)	105.33(14)	O(4)-C(14)-C(17)	109.79(17)
C(8)-C(9)-H(9A)	109.5	C(13)-C(14)-H(14)	109.3
C(8)-C(9)-H(9B)	109.5	C(13)-C(14)-C(17)	112.46(15)
C(8)-C(9)-H(9C)	109.5	C(17)-C(14)-H(14)	109.3
H(9A)-C(9)-H(9B)	109.5	O(4)-C(15)-C(16)	111.42(17)
H(9A)-C(9)-H(9C)	109.5	O(5)-C(15)-O(4)	123.48(19)
H(9B)-C(9)-H(9C)	109.5	O(5)-C(15)-C(16)	125.08(18)
C(8)-C(10)-H(10)	108.2	C(15)-C(16)-H(16A)	109.5
C(11)-C(10)-C(8)	112.64(14)	C(15)-C(16)-H(16B)	109.5
C(11)-C(10)-H(10)	108.2	C(15)-C(16)-H(16C)	109.5
C(11)-C(10)-C(13)	107.88(15)	H(16A)-C(16)-H(16B)	109.5
C(13)-C(10)-C(8)	111.67(17)	H(16A)-C(16)-H(16C)	109.5
C(13)-C(10)-H(10)	108.2	H(16B)-C(16)-H(16C)	109.5
O(2)-C(11)-O(3)	122.89(19)	O(6)-C(17)-C(14)	121.25(18)
O(2)-C(11)-C(10)	124.69(19)	O(6)-C(17)-C(18)	125.13(17)
O(3)-C(11)-C(10)	112.42(17)	C(18)-C(17)-C(14)	113.57(17)
O(3)-C(12)-H(12A)	109.5	C(8)-C(18)-H(18)	104.8
O(3)-C(12)-H(12B)	109.5	C(17)-C(18)-C(8)	110.34(15)
O(3)-C(12)-H(12C)	109.5	C(17)-C(18)-H(18)	104.8
H(12A)-C(12)-H(12B)	109.5	C(17)-C(18)-C(19)	114.25(16)
H(12A)-C(12)-H(12C)	109.5	C(19)-C(18)-C(8)	116.62(15)
H(12B)-C(12)-H(12C)	109.5	C(19)-C(18)-H(18)	104.8
C(10)-C(13)-H(13A)	109.1	C(1)-C(19)-C(18)	118.10(16)
C(10)-C(13)-H(13B)	109.1	C(1)-C(19)-H(19)	106.2
H(13A)-C(13)-H(13B)	107.8	C(5)-C(19)-C(1)	111.32(14)
C(14)-C(13)-C(10)	112.46(16)	C(5)-C(19)-C(18)	108.15(16)
C(14)-C(13)-H(13A)	109.1	C(5)-C(19)-H(19)	106.2
C(14)-C(13)-H(13B)	109.1	C(18)-C(19)-H(19)	106.2

Symmetry transformations used to generate equivalent atoms:

Table S15.4. Anisotropic displacement parameters ($Å^2$x 10^3) for Shenvi289. The anisotropic displacement factor exponent takes the form: $-2\pi^2$[h2 a*2U11 + ... + 2 h k a* b* U12]

	U11	U22	U33	U23	U13	U12
O(1)	28(1)	22(1)	29(1)	4(1)	-8(1)	4(1)
O(2)	19(1)	23(1)	30(1)	4(1)	7(1)	3(1)
O(3)	19(1)	19(1)	28(1)	-2(1)	7(1)	-2(1)
O(4)	19(1)	24(1)	18(1)	7(1)	5(1)	5(1)
O(5)	25(1)	34(1)	23(1)	8(1)	10(1)	7(1)
O(6)	29(1)	22(1)	20(1)	5(1)	2(1)	-2(1)
C(1)	23(1)	15(1)	18(1)	1(1)	3(1)	-1(1)
C(2)	25(1)	20(1)	18(1)	-3(1)	4(1)	-5(1)
C(3)	20(1)	25(1)	17(1)	-1(1)	-2(1)	-4(1)
C(4)	20(1)	21(1)	16(1)	1(1)	1(1)	1(1)
C(5)	18(1)	14(1)	14(1)	-1(1)	1(1)	0(1)
C(6)	22(1)	16(1)	14(1)	3(1)	2(1)	1(1)
C(7)	20(1)	16(1)	16(1)	2(1)	4(1)	0(1)
C(8)	16(1)	17(1)	13(1)	0(1)	3(1)	1(1)
C(9)	19(1)	21(1)	17(1)	-1(1)	5(1)	3(1)
C(10)	17(1)	18(1)	15(1)	-1(1)	4(1)	1(1)
C(11)	19(1)	20(1)	14(1)	1(1)	1(1)	2(1)
C(12)	22(1)	24(1)	35(1)	-3(1)	10(1)	-5(1)
C(13)	17(1)	23(1)	17(1)	2(1)	2(1)	2(1)
C(14)	19(1)	21(1)	15(1)	6(1)	4(1)	5(1)
C(15)	16(1)	26(1)	11(1)	2(1)	0(1)	-1(1)
C(16)	20(1)	23(1)	18(1)	3(1)	0(1)	-2(1)
C(17)	18(1)	21(1)	15(1)	1(1)	7(1)	2(1)
C(18)	16(1)	16(1)	13(1)	0(1)	3(1)	1(1)
C(19)	17(1)	17(1)	14(1)	1(1)	3(1)	1(1)

Table S15.5. Hydrogen coordinates (x 10^4) and isotropic displacement parameters ($Å^2$x 10^3) for Shenvi289.

	x	y	z	U(eq)
H(1A)	4968	3194	1078	22
H(1B)	4083	1889	1763	22
H(2)	2298	1947	461	25
H(3)	902	4775	112	25
H(5)	3602	8111	1982	19
H(6A)	5354	6592	608	21
H(6B)	4888	8945	701	21
H(7A)	6372	9175	2125	21
H(7B)	7432	8390	1395	21
H(9A)	7164	4350	1171	28
H(9B)	7783	3177	2087	28
H(9C)	8809	4904	1692	28
H(10)	7638	7899	3495	20
H(12A)	11129	10237	1984	40
H(12B)	11821	10408	3026	40
H(12C)	10803	12229	2556	40
H(13A)	9186	4018	3510	23
H(13B)	9265	5561	4350	23
H(14)	6766	4912	4579	22
H(16A)	8231	-1178	5144	31
H(16B)	6563	-1714	5402	31
H(16C)	7801	-671	6136	31
H(18)	5292	6452	3137	18
H(19)	3250	4815	2429	19

X-ray Structure of O6C **1** (CCDC2249326)

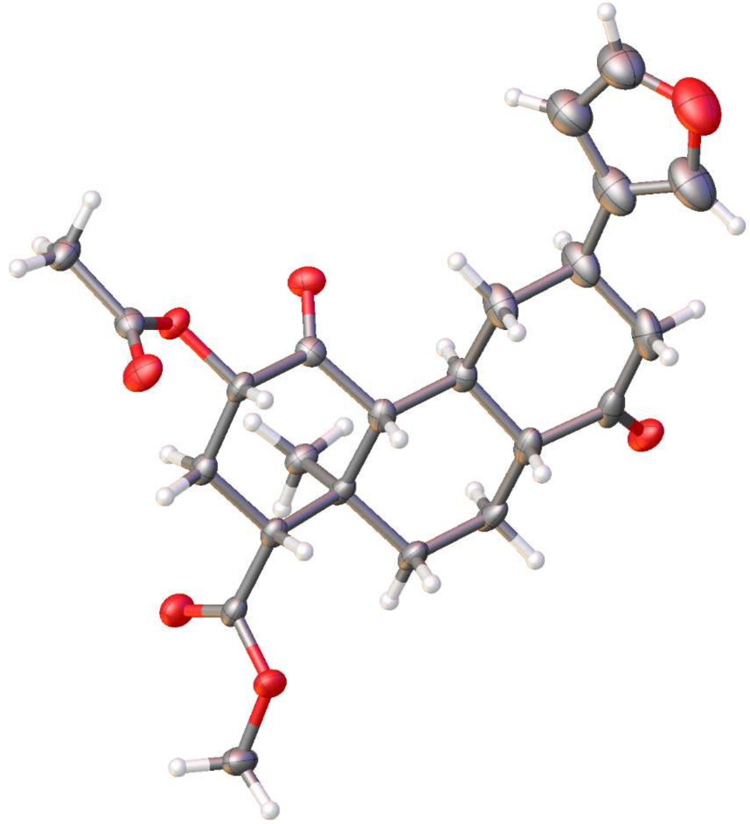

Experimental Summary

The single crystal X-ray diffraction studies were carried out on a Bruker Microstar APEX II CCD diffractometer equipped with Cu K_α radiation (λ = 1.54178 Å).

Crystals of the subject compound were used as received. A 0.2 x 0.16 x 0.06 mm piece of a crystal was mounted on a Cryoloop with Paratone oil. Data were collected in a nitrogen gas stream at 100(2) K using ϕ and ω scans. Crystal-to-detector distance was 40 mm and exposure time was 5, 10, 15, or 20 seconds depending on the 2θ range per frame using a scan width of 1.25°. Data collection was 99.9 % complete to 67.679° in θ. A total of 19144 reflections were collected covering the indices, −38<=h<=38, −7<=k<=7, −13<=l<=13. 3900 reflections were found to be symmetry independent, with a R_{int} of 0.0388. Indexing and unit cell refinement indicated a **C**-Centered, **Monoclinic** lattice. The space group was found to be ***C*2**. The data were integrated using the Bruker SAINT Software program and scaled using the SADABS software program. Solution by direct methods (SHELXT) produced a complete phasing model consistent with the proposed structure.

All nonhydrogen atoms were refined anisotropically by full-matrix least-squares (SHELXL-2014). All carbon bonded hydrogen atoms were placed using a riding model. Their positions were constrained relative to their parent atom using the appropriate HFIX command in SHELXL-2014. Crystallographic data are summarized in Table S16.1.

Notes: Absolute stereochemistry was conclusively assigned (Absolute Structure Parameter = 0.01(10)). There is one copy of the compound in the asymmetric unit. There was unusual density near the furan ring

that could not be explained. It is possible that the furan is missing in ~15% of the molecules. The chemical formula for the compound is: $C_{23}H_{28}O_7$

Table S16.1. Crystal data and structure refinement for Shenvi297.

Identification code	shenvi297_0m_a
Empirical formula	C23 H28 O7
Formula weight	416.45
Temperature	100.15 K
Wavelength	1.54178 Å
Crystal system	Monoclinic
Space group	C 1 2 1
Unit cell dimensions	a = 31.983(4) Å α= 90°.
	b = 5.9244(7) Å β = 103.574(6)°.
	c = 11.4947(13) Å γ = 90°.
Volume	2117.2(4) Å3
Z	4
Density (calculated)	1.307 Mg/m3
Absorption coefficient	0.796 mm-1
F(000)	888
Crystal size	0.2 x 0.16 x 0.06 mm3
Theta range for data collection	2.843 to 68.924°.
Index ranges	-38<=h<=38, -7<=k<=7, -13<=l<=13
Reflections collected	19144
Independent reflections	3900 [R(int) = 0.0388]
Completeness to theta = 67.679°	99.9 %
Absorption correction	Semi-empirical from equivalents
Max. and min. transmission	0.5215 and 0.4405
Refinement method	Full-matrix least-squares on F^2
Data / restraints / parameters	3900 / 1 / 274
Goodness-of-fit on F^2	1.063
Final R indices [I>2sigma(I)]	R1 = 0.0528, wR2 = 0.1465
R indices (all data)	R1 = 0.0562, wR2 = 0.1494
Absolute structure parameter	0.01(10)
Largest diff. peak and hole	0.714 and -0.391 e.Å-3

Table S16.2. Atomic coordinates (x 10^4) and equivalent isotropic displacement parameters ($Å^2$x 10^3) for Shenvi297. U(eq) is defined as one third of the trace of the orthogonalized U^{ij} tensor.

	x	y	z	U(eq)
O(2)	2413(1)	2660(5)	4594(3)	37(1)
O(3)	4452(1)	4103(5)	9953(2)	33(1)
O(4)	4427(1)	594(4)	9219(2)	27(1)
O(5)	4883(1)	8457(4)	6517(2)	25(1)
O(6)	5471(1)	6484(5)	6418(3)	35(1)
O(7)	4046(1)	8672(4)	5548(2)	28(1)
C(5)	3022(1)	5673(10)	2912(4)	42(1)
C(6)	2754(1)	3570(9)	3008(4)	43(1)
C(7)	2745(1)	3011(7)	4287(4)	32(1)
C(8)	3180(1)	2906(6)	5169(4)	29(1)
C(9)	3154(1)	2489(6)	6447(4)	30(1)
C(10)	3607(1)	2183(6)	7242(4)	26(1)
C(11)	3903(1)	4216(5)	7217(3)	21(1)
C(12)	3748(1)	6235(6)	7840(3)	25(1)
C(13)	4378(1)	3572(6)	7825(3)	21(1)
C(14)	4424(1)	2845(6)	9112(3)	22(1)
C(15)	4445(1)	-252(7)	10404(3)	33(1)
C(16)	4692(1)	5518(6)	7774(3)	23(1)
C(17)	4649(1)	6378(6)	6498(3)	22(1)
C(18)	5299(1)	8264(7)	6469(3)	25(1)
C(19)	5504(1)	10532(7)	6467(3)	31(1)
C(20)	4180(1)	6823(6)	5900(3)	22(1)
C(21)	3900(1)	4756(6)	5881(3)	23(1)
C(22)	3450(1)	5011(6)	5050(3)	25(1)
C(23)	3477(1)	5376(9)	3747(4)	39(1)
O(1)	2991(1)	6147(10)	-346(3)	71(1)
C(2)	3203(2)	8052(12)	78(5)	59(1)
C(1)	2895(2)	5024(13)	628(4)	62(2)
C(3)	3240(2)	8231(12)	1267(5)	56(1)
C(4)	3046(1)	6253(11)	1646(4)	51(1)

Table S16.3. Bond lengths [Å] and angles [°] for Shenvi297.

O(2)-C(7)	1.215(5)
O(3)-C(14)	1.208(5)
O(4)-C(14)	1.339(4)
O(4)-C(15)	1.439(4)
O(5)-C(17)	1.439(4)
O(5)-C(18)	1.350(4)
O(6)-C(18)	1.197(5)
O(7)-C(20)	1.211(5)
C(5)-H(5)	1.0000
C(5)-C(6)	1.531(7)
C(5)-C(23)	1.555(5)
C(5)-C(4)	1.514(6)
C(6)-H(6A)	0.9900
C(6)-H(6B)	0.9900
C(6)-C(7)	1.513(6)
C(7)-C(8)	1.517(5)
C(8)-H(8)	1.0000
C(8)-C(9)	1.511(6)
C(8)-C(22)	1.542(5)
C(9)-H(9A)	0.9900
C(9)-H(9B)	0.9900
C(9)-C(10)	1.531(5)
C(10)-H(10A)	0.9900
C(10)-H(10B)	0.9900
C(10)-C(11)	1.537(4)
C(11)-C(12)	1.535(5)
C(11)-C(13)	1.564(4)
C(11)-C(21)	1.567(5)
C(12)-H(12A)	0.9800
C(12)-H(12B)	0.9800
C(12)-H(12C)	0.9800
C(13)-H(13)	1.0000
C(13)-C(14)	1.514(5)
C(13)-C(16)	1.539(5)
C(15)-H(15A)	0.9800
C(15)-H(15B)	0.9800
C(15)-H(15C)	0.9800
C(16)-H(16A)	0.9900
C(16)-H(16B)	0.9900
C(16)-C(17)	1.528(5)
C(17)-H(17)	1.0000
C(17)-C(20)	1.518(4)
C(18)-C(19)	1.495(5)
C(19)-H(19A)	0.9800
C(19)-H(19B)	0.9800
C(19)-H(19C)	0.9800
C(20)-C(21)	1.513(5)
C(21)-H(21)	1.0000
C(21)-C(22)	1.536(4)
C(22)-H(22)	1.0000
C(22)-C(23)	1.536(5)
C(23)-H(23A)	0.9900
C(23)-H(23B)	0.9900
O(1)-C(2)	1.349(8)
O(1)-C(1)	1.396(7)
C(2)-H(2)	0.9500
C(2)-C(3)	1.348(7)
C(1)-H(1)	0.9500
C(1)-C(4)	1.367(8)
C(3)-H(3)	0.9500
C(3)-C(4)	1.440(9)
C(14)-O(4)-C(15)	115.6(3)
C(18)-O(5)-C(17)	116.2(3)
C(6)-C(5)-H(5)	107.3
C(6)-C(5)-C(23)	108.8(4)
C(23)-C(5)-H(5)	107.3
C(4)-C(5)-H(5)	107.3
C(4)-C(5)-C(6)	114.2(4)
C(4)-C(5)-C(23)	111.5(3)
C(5)-C(6)-H(6A)	109.1
C(5)-C(6)-H(6B)	109.1
H(6A)-C(6)-H(6B)	107.8
C(7)-C(6)-C(5)	112.7(3)
C(7)-C(6)-H(6A)	109.1
C(7)-C(6)-H(6B)	109.1
O(2)-C(7)-C(6)	122.4(3)
O(2)-C(7)-C(8)	121.7(4)
C(6)-C(7)-C(8)	115.9(3)
C(7)-C(8)-H(8)	106.5
C(7)-C(8)-C(22)	110.7(3)
C(9)-C(8)-C(7)	113.9(3)
C(9)-C(8)-H(8)	106.5
C(9)-C(8)-C(22)	112.2(3)
C(22)-C(8)-H(8)	106.5
C(8)-C(9)-H(9A)	109.7
C(8)-C(9)-H(9B)	109.7
C(8)-C(9)-C(10)	109.9(3)
H(9A)-C(9)-H(9B)	108.2
C(10)-C(9)-H(9A)	109.7

C(10)-C(9)-H(9B)	109.7
C(9)-C(10)-H(10A)	108.9
C(9)-C(10)-H(10B)	108.9
C(9)-C(10)-C(11)	113.4(3)
H(10A)-C(10)-H(10B)	107.7
C(11)-C(10)-H(10A)	108.9
C(11)-C(10)-H(10B)	108.9
C(10)-C(11)-C(13)	109.8(3)
C(10)-C(11)-C(21)	108.5(3)
C(12)-C(11)-C(10)	109.5(3)
C(12)-C(11)-C(13)	111.6(3)
C(12)-C(11)-C(21)	111.7(3)
C(13)-C(11)-C(21)	105.6(2)
C(11)-C(12)-H(12A)	109.5
C(11)-C(12)-H(12B)	109.5
C(11)-C(12)-H(12C)	109.5
H(12A)-C(12)-H(12B)	109.5
H(12A)-C(12)-H(12C)	109.5
H(12B)-C(12)-H(12C)	109.5
C(11)-C(13)-H(13)	107.8
C(14)-C(13)-C(11)	111.6(3)
C(14)-C(13)-H(13)	107.8
C(14)-C(13)-C(16)	109.6(3)
C(16)-C(13)-C(11)	112.0(3)
C(16)-C(13)-H(13)	107.8
O(3)-C(14)-O(4)	122.9(3)
O(3)-C(14)-C(13)	125.4(3)
O(4)-C(14)-C(13)	111.8(3)
O(4)-C(15)-H(15A)	109.5
O(4)-C(15)-H(15B)	109.5
O(4)-C(15)-H(15C)	109.5
H(15A)-C(15)-H(15B)	109.5
H(15A)-C(15)-H(15C)	109.5
H(15B)-C(15)-H(15C)	109.5
C(13)-C(16)-H(16A)	109.2
C(13)-C(16)-H(16B)	109.2
H(16A)-C(16)-H(16B)	107.9
C(17)-C(16)-C(13)	111.9(3)
C(17)-C(16)-H(16A)	109.2
C(17)-C(16)-H(16B)	109.2
O(5)-C(17)-C(16)	109.9(3)
O(5)-C(17)-H(17)	109.4
O(5)-C(17)-C(20)	108.3(3)
C(16)-C(17)-H(17)	109.4
C(20)-C(17)-C(16)	110.5(3)
C(20)-C(17)-H(17)	109.4
O(5)-C(18)-C(19)	111.2(3)
O(6)-C(18)-O(5)	123.0(3)
O(6)-C(18)-C(19)	125.8(3)
C(18)-C(19)-H(19A)	109.5
C(18)-C(19)-H(19B)	109.5
C(18)-C(19)-H(19C)	109.5
H(19A)-C(19)-H(19B)	109.5
H(19A)-C(19)-H(19C)	109.5
H(19B)-C(19)-H(19C)	109.5
O(7)-C(20)-C(17)	122.8(3)
O(7)-C(20)-C(21)	124.5(3)
C(21)-C(20)-C(17)	112.6(3)
C(11)-C(21)-H(21)	107.4
C(20)-C(21)-C(11)	106.5(3)
C(20)-C(21)-H(21)	107.4
C(20)-C(21)-C(22)	113.2(3)
C(22)-C(21)-C(11)	114.7(3)
C(22)-C(21)-H(21)	107.4
C(8)-C(22)-H(22)	108.4
C(21)-C(22)-C(8)	109.2(3)
C(21)-C(22)-H(22)	108.4
C(23)-C(22)-C(8)	111.2(3)
C(23)-C(22)-C(21)	111.2(3)
C(23)-C(22)-H(22)	108.4
C(5)-C(23)-H(23A)	109.4
C(5)-C(23)-H(23B)	109.4
C(22)-C(23)-C(5)	111.1(3)
C(22)-C(23)-H(23A)	109.4
C(22)-C(23)-H(23B)	109.4
H(23A)-C(23)-H(23B)	108.0
C(2)-O(1)-C(1)	107.2(4)
O(1)-C(2)-H(2)	124.8
C(3)-C(2)-O(1)	110.4(5)
C(3)-C(2)-H(2)	124.8
O(1)-C(1)-H(1)	125.3
C(4)-C(1)-O(1)	109.4(6)
C(4)-C(1)-H(1)	125.3
C(2)-C(3)-H(3)	126.2
C(2)-C(3)-C(4)	107.6(6)
C(4)-C(3)-H(3)	126.2
C(1)-C(4)-C(5)	127.9(6)
C(1)-C(4)-C(3)	105.3(5)
C(3)-C(4)-C(5)	126.8(5)

Symmetry transformations used to generate equivalent atoms:

Table S16.4. Anisotropic displacement parameters ($Å^2$x 10^3) for Shenvi297. The anisotropic displacement factor exponent takes the form: $-2\pi^2$[h2 a*2U11 + ... + 2 h k a* b* U12]

	U11	U22	U33	U23	U13	U12
O(2)	22(1)	29(2)	56(2)	11(1)	3(1)	-4(1)
O(3)	43(2)	24(1)	30(1)	-4(1)	9(1)	-3(1)
O(4)	34(1)	18(1)	28(1)	1(1)	6(1)	1(1)
O(5)	23(1)	21(1)	33(1)	-2(1)	11(1)	-4(1)
O(6)	22(1)	32(2)	55(2)	3(1)	16(1)	0(1)
O(7)	26(1)	26(1)	36(1)	6(1)	12(1)	5(1)
C(5)	22(2)	70(3)	35(2)	2(2)	7(2)	4(2)
C(6)	22(2)	64(3)	41(2)	-16(2)	1(2)	1(2)
C(7)	20(2)	21(2)	49(2)	-11(2)	1(2)	1(1)
C(8)	18(2)	23(2)	42(2)	-6(2)	2(2)	2(1)
C(9)	17(2)	19(2)	52(2)	4(2)	6(2)	-3(1)
C(10)	22(2)	16(2)	40(2)	2(1)	5(2)	-2(1)
C(11)	17(1)	13(2)	32(2)	-1(1)	7(1)	0(1)
C(12)	23(2)	21(2)	32(2)	-1(1)	12(1)	0(1)
C(13)	18(2)	16(2)	28(2)	-3(1)	5(1)	1(1)
C(14)	18(2)	18(2)	30(2)	-2(1)	4(1)	-2(1)
C(15)	43(2)	25(2)	30(2)	5(2)	8(2)	-2(2)
C(16)	17(1)	22(2)	31(2)	-2(1)	6(1)	-1(1)
C(17)	20(2)	18(2)	30(2)	-3(1)	7(1)	-4(1)
C(18)	22(2)	32(2)	23(2)	1(1)	8(1)	-4(2)
C(19)	36(2)	30(2)	31(2)	-4(2)	13(2)	-10(2)
C(20)	20(2)	26(2)	21(2)	-1(1)	9(1)	3(1)
C(21)	16(2)	22(2)	29(2)	-5(1)	5(1)	3(1)
C(22)	17(2)	28(2)	30(2)	-2(1)	6(1)	2(1)
C(23)	21(2)	63(3)	33(2)	-4(2)	6(2)	1(2)
O(1)	60(2)	109(4)	44(2)	-3(2)	15(2)	-3(2)
C(2)	56(3)	79(4)	44(3)	7(3)	16(2)	-4(3)
C(1)	54(3)	97(5)	36(3)	-3(3)	12(2)	7(3)
C(3)	48(3)	73(4)	46(3)	5(3)	10(2)	2(3)
C(4)	29(2)	84(4)	41(2)	3(3)	9(2)	5(2)

Table S16.5. Hydrogen coordinates (x 10^4) and isotropic displacement parameters ($Å^2$x 10^3) for Shenvi297.

	x	y	z	U(eq)
H(5)	2882	6977	3224	51
H(6A)	2455	3815	2539	52
H(6B)	2873	2269	2653	52
H(8)	3335	1584	4929	34
H(9A)	2981	1117	6486	36
H(9B)	3011	3782	6738	36
H(10A)	3739	822	6978	32
H(10B)	3585	1926	8077	32
H(12A)	3465	6719	7376	37
H(12B)	3953	7483	7895	37
H(12C)	3727	5793	8646	37
H(13)	4461	2265	7377	25
H(15A)	4440	-1905	10387	49
H(15B)	4197	308	10679	49
H(15C)	4711	267	10950	49
H(16A)	4636	6774	8284	27
H(16B)	4991	4991	8098	27
H(17)	4767	5224	6026	26
H(19A)	5443	11121	5648	47
H(19B)	5816	10393	6776	47
H(19C)	5388	11568	6977	47
H(21)	4046	3472	5570	27
H(22)	3309	6360	5313	30
H(23A)	3621	4065	3476	47
H(23B)	3652	6736	3697	47
H(2)	3312	9118	-394	71
H(1)	2748	3623	588	75
H(3)	3370	9442	1764	67

X-ray Structure of analog **24a (**CCDC 2263493)

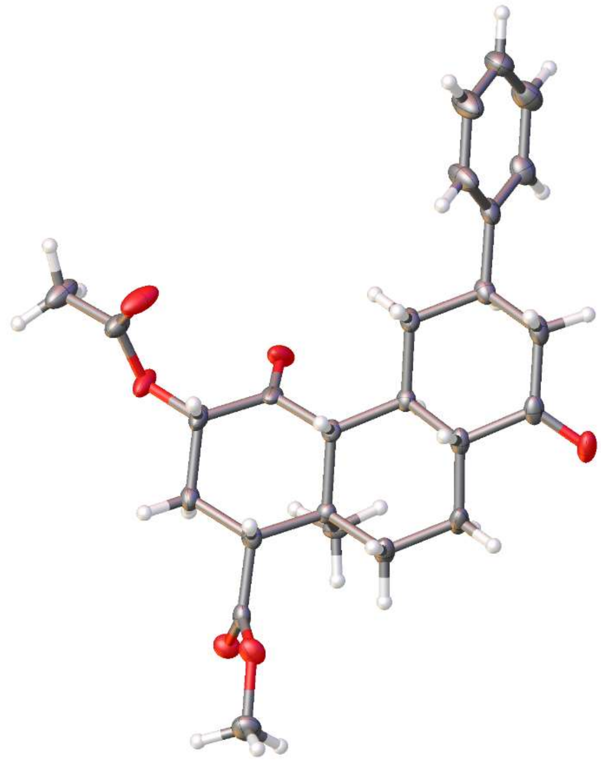

Experimental Summary

The single crystal X-ray diffraction studies were carried out on a Bruker Smart APEX II CCD diffractometer equipped with Cu K_α radiation (λ = 1.54178 Å).

Crystals of the subject compound were used as received. A 0.2 x 0.18 x 0.15 mm piece of a crystal was mounted on a Cryoloop with Paratone oil. Data were collected in a nitrogen gas stream at 100(2) K using ϕ and ω scans. Crystal-to-detector distance was 40 mm and exposure time was 5, 10, 15, or 20 seconds depending on the 2θ range per frame using a scan width of 1.25°. Data collection was 100 % complete to 67.679° in θ. A total of 25059 reflections were collected covering the indices, −37<=h<=37, −7<=k<=7, −15<=l<=15. 4221 reflections were found to be symmetry independent, with a R_{int} of 0.0248. Indexing and unit cell refinement indicated a **P**rimitive, **Monoclinic** lattice. The space group was found to be ***C*2**. The data were integrated using the Bruker SAINT Software program and scaled using the SADABS software program. Solution by direct methods (SHELXT) produced a complete phasing model consistent with the proposed structure.

All nonhydrogen atoms were refined anisotropically by full-matrix least-squares (SHELXL-2014). All carbon bonded hydrogen atoms were placed using a riding model. Their positions were constrained relative to their parent atom using the appropriate HFIX command in SHELXL-2014. Crystallographic data are summarized in Table S17.1.

Notes: Great data! Absolute stereochemistry was conclusively assigned (Absolute Structure Parameter = 0.07(7)). There is one copy of the compound in the asymmetric unit. There is some minor positional disorder on the ester. The chemical formula for the compound is: $C_{25}H_{30}O_6$

Table S17.1. Crystal data and structure refinement for Shenvi303.

Identification code	shenvi303_0m_a	
Empirical formula	C25 H30 O6	
Formula weight	426.49	
Temperature	100.15 K	
Wavelength	1.54178 Å	
Crystal system	Monoclinic	
Space group	C 1 2 1	
Unit cell dimensions	a = 30.7768(8) Å	α= 90°.
	b = 5.9625(2) Å	β= 106.0350(10)°.
	c = 12.6590(3) Å	γ = 90°.
Volume	2232.63(11) $Å^3$	
Z	4	
Density (calculated)	1.269 Mg/m^3	
Absorption coefficient	0.733 mm^{-1}	
F(000)	912	
Crystal size	0.2 x 0.18 x 0.15 mm^3	
Theta range for data collection	2.988 to 70.023°.	
Index ranges	-37<=h<=37, -7<=k<=7, -15<=l<=15	
Reflections collected	25059	
Independent reflections	4221 [R(int) = 0.0248]	
Completeness to theta = 67.679°	100.0 %	
Absorption correction	Semi-empirical from equivalents	
Max. and min. transmission	0.5220 and 0.4428	
Refinement method	Full-matrix least-squares on F^2	
Data / restraints / parameters	4221 / 1 / 312	
Goodness-of-fit on F^2	1.068	
Final R indices [I>2sigma(I)]	R1 = 0.0259, wR2 = 0.0658	
R indices (all data)	R1 = 0.0264, wR2 = 0.0662	

Absolute structure parameter	0.07(7)
Largest diff. peak and hole	0.139 and -0.153 e.Å^{-3}

Table S17.2. Atomic coordinates (x 10^4) and equivalent isotropic displacement parameters ($Å^2$x 10^3) for Shenvi303. U(eq) is defined as one third of the trace of the orthogonalized U^{ij} tensor.

	x	y	z	U(eq)
O(2)	4975(1)	8193(2)	3033(1)	32(1)
O(3)	5616(1)	4127(2)	168(1)	24(1)
O(4)	5577(1)	624(2)	773(1)	25(1)
O(5)	7574(1)	2253(2)	5575(1)	30(1)
O(6)	5850(1)	8621(2)	4175(1)	33(1)
C(3)	5256(1)	6239(3)	3126(1)	25(1)
C(4)	5261(1)	5493(3)	1977(1)	22(1)
C(5)	5598(1)	3558(3)	2048(1)	19(1)
C(6)	5597(1)	2863(3)	897(1)	19(1)
C(7)	5593(1)	-189(3)	-293(1)	30(1)
C(8)	6086(1)	4161(2)	2758(1)	17(1)
C(9)	6286(1)	6133(3)	2269(1)	20(1)
C(10)	6388(1)	2089(3)	2839(1)	24(1)
C(11)	6847(1)	2321(3)	3696(1)	26(1)
C(12)	6779(1)	2860(3)	4815(1)	22(1)
C(13)	7221(1)	2888(3)	5723(1)	23(1)
C(14)	7191(1)	3661(3)	6834(1)	27(1)
C(15)	6921(1)	5847(3)	6770(1)	22(1)
C(16)	6892(1)	6569(3)	7899(1)	24(1)
C(17)	7153(1)	8329(3)	8445(1)	29(1)
C(18)	7125(1)	9026(3)	9478(2)	36(1)
C(19)	6836(1)	7951(4)	9971(2)	38(1)
C(20)	6579(1)	6168(4)	9443(2)	42(1)
C(21)	6608(1)	5475(4)	8416(1)	35(1)
C(22)	6456(1)	5565(3)	5941(1)	21(1)
C(23)	6504(1)	5036(3)	4792(1)	18(1)

C(24)	6044(1)	4755(3)	3934(1)	18(1)
C(25)	5734(1)	6768(3)	3818(1)	21(1)
O(1)	4749(1)	6810(5)	4463(2)	44(1)
C(1)	4440(2)	10240(13)	3579(5)	38(2)
C(2)	4719(1)	8154(6)	3742(4)	26(1)
O(1A)	4416(1)	6208(5)	3112(3)	38(1)
C(1A)	4327(2)	10219(13)	3130(6)	35(1)
C(2A)	4572(1)	8046(7)	3100(4)	28(1)

Table S17.3. Bond lengths [Å] and angles [°] for Shenvi303.

O(2)-C(3)	1.437(2)	C(11)-H(11A)	0.9900
O(2)-C(2)	1.348(4)	C(11)-H(11B)	0.9900
O(2)-C(2A)	1.269(4)	C(11)-C(12)	1.522(2)
O(3)-C(6)	1.206(2)	C(12)-H(12)	1.0000
O(4)-C(6)	1.344(2)	C(12)-C(13)	1.519(2)
O(4)-C(7)	1.448(2)	C(12)-C(23)	1.545(2)
O(5)-C(13)	1.213(2)	C(13)-C(14)	1.507(2)
O(6)-C(25)	1.210(2)	C(14)-H(14A)	0.9900
C(3)-H(3)	1.0000	C(14)-H(14B)	0.9900
C(3)-C(4)	1.525(2)	C(14)-C(15)	1.536(2)
C(3)-C(25)	1.522(2)	C(15)-H(15)	1.0000
C(4)-H(4A)	0.9900	C(15)-C(16)	1.518(2)
C(4)-H(4B)	0.9900	C(15)-C(22)	1.534(2)
C(4)-C(5)	1.535(2)	C(16)-C(17)	1.385(3)
C(5)-H(5)	1.0000	C(16)-C(21)	1.393(2)
C(5)-C(6)	1.514(2)	C(17)-H(17)	0.9500
C(5)-C(8)	1.5643(19)	C(17)-C(18)	1.398(3)
C(7)-H(7A)	0.9800	C(18)-H(18)	0.9500
C(7)-H(7B)	0.9800	C(18)-C(19)	1.376(3)
C(7)-H(7C)	0.9800	C(19)-H(19)	0.9500
C(8)-C(9)	1.536(2)	C(19)-C(20)	1.383(3)
C(8)-C(10)	1.534(2)	C(20)-H(20)	0.9500
C(8)-C(24)	1.5695(19)	C(20)-C(21)	1.390(3)
C(9)-H(9A)	0.9800	C(21)-H(21)	0.9500
C(9)-H(9B)	0.9800	C(22)-H(22A)	0.9900
C(9)-H(9C)	0.9800	C(22)-H(22B)	0.9900
C(10)-H(10A)	0.9900	C(22)-C(23)	1.534(2)
C(10)-H(10B)	0.9900	C(23)-H(23)	1.0000
C(10)-C(11)	1.529(2)	C(23)-C(24)	1.5370(19)

C(24)-H(24)	1.0000
C(24)-C(25)	1.516(2)
O(1)-O(1)#1	1.756(6)
O(1)-C(2)	1.198(5)
C(1)-H(1A)	0.9800
C(1)-H(1B)	0.9800
C(1)-H(1C)	0.9800
C(1)-C(2)	1.494(8)
O(1A)-C(2A)	1.198(5)
C(1A)-H(1AA)	0.9800
C(1A)-H(1AB)	0.9800
C(1A)-H(1AC)	0.9800
C(1A)-C(2A)	1.505(8)

C(2)-O(2)-C(3)	112.4(2)
C(2A)-O(2)-C(3)	121.1(2)
C(6)-O(4)-C(7)	115.35(13)
O(2)-C(3)-H(3)	109.2
O(2)-C(3)-C(4)	108.86(12)
O(2)-C(3)-C(25)	110.06(15)
C(4)-C(3)-H(3)	109.2
C(25)-C(3)-H(3)	109.2
C(25)-C(3)-C(4)	110.29(12)
C(3)-C(4)-H(4A)	109.6
C(3)-C(4)-H(4B)	109.6
C(3)-C(4)-C(5)	110.34(12)
H(4A)-C(4)-H(4B)	108.1
C(5)-C(4)-H(4A)	109.6
C(5)-C(4)-H(4B)	109.6
C(4)-C(5)-H(5)	107.9
C(4)-C(5)-C(8)	112.66(12)
C(6)-C(5)-C(4)	109.14(12)
C(6)-C(5)-H(5)	107.9
C(6)-C(5)-C(8)	111.18(12)
C(8)-C(5)-H(5)	107.9
O(3)-C(6)-O(4)	122.88(15)
O(3)-C(6)-C(5)	125.32(14)
O(4)-C(6)-C(5)	111.79(13)
O(4)-C(7)-H(7A)	109.5
O(4)-C(7)-H(7B)	109.5
O(4)-C(7)-H(7C)	109.5
H(7A)-C(7)-H(7B)	109.5
H(7A)-C(7)-H(7C)	109.5
H(7B)-C(7)-H(7C)	109.5
C(5)-C(8)-C(24)	106.30(11)
C(9)-C(8)-C(5)	111.93(12)
C(9)-C(8)-C(24)	110.92(12)
C(10)-C(8)-C(5)	108.81(12)
C(10)-C(8)-C(9)	109.81(12)
C(10)-C(8)-C(24)	108.97(12)
C(8)-C(9)-H(9A)	109.5
C(8)-C(9)-H(9B)	109.5
C(8)-C(9)-H(9C)	109.5
H(9A)-C(9)-H(9B)	109.5
H(9A)-C(9)-H(9C)	109.5
H(9B)-C(9)-H(9C)	109.5
C(8)-C(10)-H(10A)	108.9
C(8)-C(10)-H(10B)	108.9
H(10A)-C(10)-H(10B)	107.7
C(11)-C(10)-C(8)	113.51(13)
C(11)-C(10)-H(10A)	108.9
C(11)-C(10)-H(10B)	108.9

C(10)-C(11)-H(11A)	109.7	C(16)-C(17)-C(18)	120.99(17)
C(10)-C(11)-H(11B)	109.7	C(18)-C(17)-H(17)	119.5
H(11A)-C(11)-H(11B)	108.2	C(17)-C(18)-H(18)	120.0
C(12)-C(11)-C(10)	110.04(14)	C(19)-C(18)-C(17)	120.04(18)
C(12)-C(11)-H(11A)	109.7	C(19)-C(18)-H(18)	120.0
C(12)-C(11)-H(11B)	109.7	C(18)-C(19)-H(19)	120.2
C(11)-C(12)-H(12)	106.6	C(18)-C(19)-C(20)	119.69(17)
C(11)-C(12)-C(23)	112.05(13)	C(20)-C(19)-H(19)	120.2
C(13)-C(12)-C(11)	112.52(13)	C(19)-C(20)-H(20)	119.9
C(13)-C(12)-H(12)	106.6	C(19)-C(20)-C(21)	120.23(19)
C(13)-C(12)-C(23)	112.06(13)	C(21)-C(20)-H(20)	119.9
C(23)-C(12)-H(12)	106.6	C(16)-C(21)-H(21)	119.6
O(5)-C(13)-C(12)	122.15(15)	C(20)-C(21)-C(16)	120.79(19)
O(5)-C(13)-C(14)	121.83(14)	C(20)-C(21)-H(21)	119.6
C(14)-C(13)-C(12)	115.97(13)	C(15)-C(22)-H(22A)	109.5
C(13)-C(14)-H(14A)	109.1	C(15)-C(22)-H(22B)	109.5
C(13)-C(14)-H(14B)	109.1	C(15)-C(22)-C(23)	110.76(12)
C(13)-C(14)-C(15)	112.44(13)	H(22A)-C(22)-H(22B)	108.1
H(14A)-C(14)-H(14B)	107.8	C(23)-C(22)-H(22A)	109.5
C(15)-C(14)-H(14A)	109.1	C(23)-C(22)-H(22B)	109.5
C(15)-C(14)-H(14B)	109.1	C(12)-C(23)-H(23)	108.5
C(14)-C(15)-H(15)	107.7	C(22)-C(23)-C(12)	110.59(12)
C(16)-C(15)-C(14)	111.24(13)	C(22)-C(23)-H(23)	108.5
C(16)-C(15)-H(15)	107.7	C(22)-C(23)-C(24)	112.37(11)
C(16)-C(15)-C(22)	112.93(13)	C(24)-C(23)-C(12)	108.30(12)
C(22)-C(15)-C(14)	109.41(13)	C(24)-C(23)-H(23)	108.5
C(22)-C(15)-H(15)	107.7	C(8)-C(24)-H(24)	107.0
C(17)-C(16)-C(15)	120.57(15)	C(23)-C(24)-C(8)	113.27(11)
C(17)-C(16)-C(21)	118.25(16)	C(23)-C(24)-H(24)	107.0
C(21)-C(16)-C(15)	121.18(15)	C(25)-C(24)-C(8)	107.49(11)
C(16)-C(17)-H(17)	119.5	C(25)-C(24)-C(23)	114.52(13)

C(25)-C(24)-H(24)	107.0	O(1)-C(2)-O(2)	125.7(3)
O(6)-C(25)-C(3)	122.95(15)	O(1)-C(2)-C(1)	125.6(4)
O(6)-C(25)-C(24)	124.95(14)	H(1AA)-C(1A)-H(1AB)	109.5
C(24)-C(25)-C(3)	111.98(14)	H(1AA)-C(1A)-H(1AC)	109.5
C(2)-O(1)-O(1)#1	117.1(3)	H(1AB)-C(1A)-H(1AC)	109.5
H(1A)-C(1)-H(1B)	109.5	C(2A)-C(1A)-H(1AA)	109.5
H(1A)-C(1)-H(1C)	109.5	C(2A)-C(1A)-H(1AB)	109.5
H(1B)-C(1)-H(1C)	109.5	C(2A)-C(1A)-H(1AC)	109.5
C(2)-C(1)-H(1A)	109.5	O(2)-C(2A)-C(1A)	116.6(4)
C(2)-C(1)-H(1B)	109.5	O(1A)-C(2A)-O(2)	117.8(3)
C(2)-C(1)-H(1C)	109.5	O(1A)-C(2A)-C(1A)	125.6(4)
O(2)-C(2)-C(1)	108.3(4)		

Symmetry transformations used to generate equivalent atoms:

#1 -x+1,y,-z+1

Table S17.4. Anisotropic displacement parameters ($Å^2$x 10^3) for Shenvi303. The anisotropic displacement factor exponent takes the form: $-2\pi^2[h^2 a^{*2}U^{11} + ... + 2 h k a^* b^* U^{12}]$

	U^{11}	U^{22}	U^{33}	U^{23}	U^{13}	U^{12}
O(2)	27(1)	49(1)	26(1)	15(1)	14(1)	21(1)
O(3)	30(1)	22(1)	21(1)	2(1)	6(1)	1(1)
O(4)	29(1)	18(1)	24(1)	-1(1)	2(1)	-3(1)
O(5)	17(1)	27(1)	41(1)	-6(1)	-1(1)	5(1)
O(6)	41(1)	33(1)	20(1)	-7(1)	0(1)	19(1)
C(3)	19(1)	37(1)	22(1)	9(1)	9(1)	11(1)
C(4)	17(1)	28(1)	21(1)	5(1)	4(1)	4(1)
C(5)	16(1)	19(1)	21(1)	3(1)	4(1)	-2(1)
C(6)	13(1)	19(1)	24(1)	1(1)	1(1)	0(1)
C(7)	35(1)	24(1)	30(1)	-7(1)	7(1)	1(1)
C(8)	15(1)	16(1)	19(1)	-1(1)	4(1)	-1(1)
C(9)	23(1)	21(1)	19(1)	-4(1)	9(1)	-5(1)
C(10)	19(1)	19(1)	31(1)	-6(1)	1(1)	2(1)
C(11)	17(1)	24(1)	33(1)	-9(1)	1(1)	5(1)
C(12)	15(1)	18(1)	28(1)	0(1)	1(1)	0(1)
C(13)	18(1)	16(1)	32(1)	2(1)	1(1)	2(1)
C(14)	19(1)	32(1)	26(1)	6(1)	-1(1)	2(1)
C(15)	18(1)	26(1)	20(1)	4(1)	3(1)	-3(1)
C(16)	19(1)	31(1)	20(1)	4(1)	0(1)	2(1)
C(17)	29(1)	28(1)	26(1)	6(1)	2(1)	0(1)
C(18)	42(1)	30(1)	31(1)	-4(1)	1(1)	-1(1)
C(19)	36(1)	53(1)	23(1)	-6(1)	4(1)	2(1)
C(20)	33(1)	69(2)	25(1)	-2(1)	9(1)	-13(1)
C(21)	30(1)	52(1)	22(1)	-5(1)	4(1)	-17(1)
C(22)	16(1)	28(1)	18(1)	4(1)	4(1)	1(1)
C(23)	14(1)	19(1)	20(1)	2(1)	4(1)	0(1)

C(24)	15(1)	21(1)	17(1)	4(1)	5(1)	0(1)
C(25)	21(1)	33(1)	11(1)	3(1)	8(1)	8(1)
O(1)	62(2)	43(2)	40(2)	19(1)	36(1)	28(2)
C(1)	46(4)	39(3)	38(3)	16(3)	27(3)	22(3)
C(2)	27(2)	28(2)	26(2)	3(2)	13(2)	9(2)
O(1A)	18(1)	37(2)	62(2)	3(1)	14(1)	-1(1)
C(1A)	34(3)	40(3)	36(3)	6(3)	16(3)	19(2)
C(2A)	18(2)	41(2)	24(2)	2(2)	6(2)	6(2)

Table S17.5. Hydrogen coordinates (x 10^4) and isotropic displacement parameters ($Å^2$x 10^3) for Shenvi303.

	x	y	z	U(eq)
H(3)	5128	5007	3485	30
H(4A)	5349	6771	1580	26
H(4B)	4955	4999	1560	26
H(5)	5489	2254	2402	23
H(7A)	5328	358	-858	45
H(7B)	5593	-1832	-292	45
H(7C)	5869	358	-451	45
H(9A)	6075	7398	2139	30
H(9B)	6339	5672	1571	30
H(9C)	6573	6589	2783	30
H(10A)	6229	770	3026	29
H(10B)	6439	1808	2111	29
H(11A)	7019	905	3739	31
H(11B)	7023	3533	3474	31
H(12)	6594	1612	4992	26
H(14A)	7500	3892	7320	32
H(14B)	7046	2474	7165	32
H(15)	7086	7046	6486	26
H(17)	7355	9074	8113	35
H(18)	7304	10244	9840	44
H(19)	6815	8429	10670	46
H(20)	6381	5415	9783	51
H(21)	6431	4241	8063	42
H(22A)	6279	6962	5911	25
H(22B)	6290	4334	6182	25
H(23)	6670	6300	4558	21

H(24)	5888	3467	4179	21
H(1A)	4630	11534	3531	57
H(1B)	4312	10439	4201	57
H(1C)	4194	10115	2898	57
H(1AA)	4341	10588	3893	53
H(1AB)	4010	10064	2701	53
H(1AC)	4470	11419	2817	53

X-ray Structure of analog **24b (**CCDC 2268905)

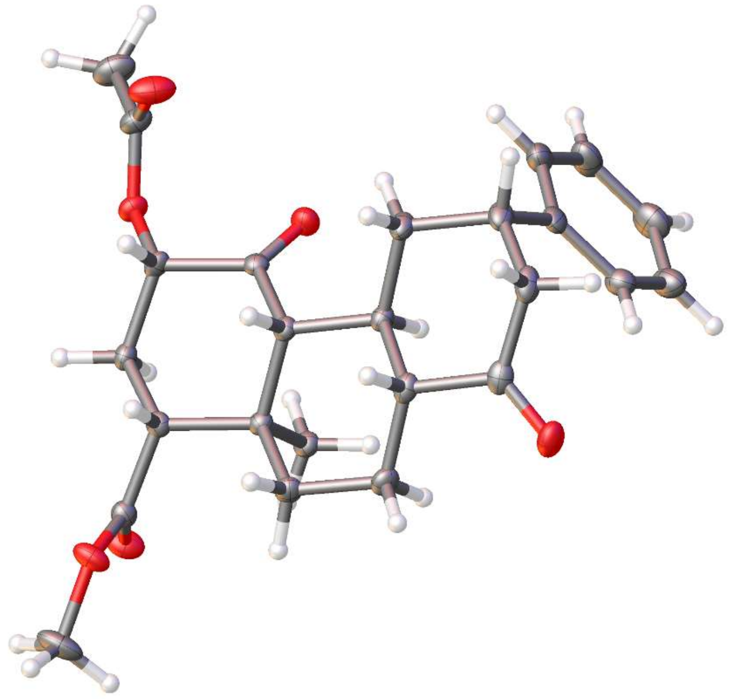

Experimental Summary

The single crystal X-ray diffraction studies were carried out on a Bruker SMART Pt 135 diffractometer equipped with Cu K_α radiation (λ = 1.54178). Crystals of the subject compound were used as received (grown from CDCl3/Pentane). A 0.200 x 0.200 x 0.170 mm colorless crystal was mounted on a Cryoloop with Paratone oil.

Data were collected in a nitrogen gas stream at 100(2) K using ϕ and ϖ scans. Crystal-to-detector distance was 45 mm using exposure time 1.0, 2.0 and 4.0 second (depending on the 2θ position) with a scan width of 1.50°. Data collection was 100.0 % complete to 67.679° in θ.

A total of 21563 reflections were collected. 4315 reflections were found to be symmetry independent, with a R_{int} of 0.0495. Indexing and unit cell refinement indicated an **Orthorhombic** lattice. The space group was found to be $\boldsymbol{P\ 2_12_12_1}$**.** The data were integrated using the Bruker SAINT Software program and scaled using the SADABS software program. Solution by direct methods (SHELXT) produced a complete phasing model consistent with the proposed structure.

All nonhydrogen atoms were refined anisotropically by full-matrix least-squares (SHELXL-2014). All carbon bonded hydrogen atoms were placed using a riding model. Their positions were constrained relative to their parent atom using the appropriate HFIX command in SHELXL-2014.

Absolute structure parameter -0.05(8) conclusive

Table S18.1. Crystal data and structure refinement for Shenvi305.

Report date	2023-06-09	
Identification code	shenvi305	
Empirical formula	C25 H30 O6	
Molecular formula	C25 H30 O6	
Formula weight	426.49	
Temperature	100.15 K	
Wavelength	1.54178 Å	
Crystal system	Orthorhombic	
Space group	$P2_12_12_1$	
Unit cell dimensions	a = 9.9970(5) Å	α= 90°.
	b = 11.1325(6) Å	β= 90°.
	c = 20.4030(10) Å	γ = 90°.
Volume	2270.7(2) $Å^3$	
Z	4	
Density (calculated)	1.248 Mg/m^3	
Absorption coefficient	0.721 mm^{-1}	
F(000)	912	
Crystal size	0.2 x 0.2 x 0.17 mm^3	
Crystal color, habit	colorless block	
Theta range for data collection	4.334 to 70.359°.	
Index ranges	-12<=h<=12, -13<=k<=13, -24<=l<=24	
Reflections collected	21563	
Independent reflections	4315 [R(int) = 0.0495]	
Completeness to theta = 67.679°	100.0 %	
Absorption correction	Semi-empirical from equivalents	
Max. and min. transmission	0.7533 and 0.6412	
Refinement method	Full-matrix least-squares on F^2	
Data / restraints / parameters	4315 / 0 / 284	
Goodness-of-fit on F^2	1.071	
Final R indices [I>2sigma(I)]	R1 = 0.0358, wR2 = 0.0926	
R indices (all data)	R1 = 0.0362, wR2 = 0.0932	
Absolute structure parameter	-0.05(8)	
Extinction coefficient	0.0033(5)	
Largest diff. peak and hole	0.256 and -0.184 e.$Å^{-3}$	

Table S18.2. Atomic coordinates (x 10^4) and equivalent isotropic displacement parameters ($Å^2$x 10^3) for Shenvi305. U(eq) is defined as one third of the trace of the orthogonalized U^{ij} tensor.

	x	y	z	U(eq)
O(1)	8080(1)	6250(1)	6583(1)	22(1)
O(2)	8893(1)	6290(1)	7847(1)	19(1)
O(3)	10532(2)	5206(1)	7372(1)	35(1)
O(4)	3785(2)	3982(1)	8491(1)	25(1)
O(5)	3945(2)	5994(1)	8453(1)	26(1)
O(6)	3869(2)	3618(1)	4826(1)	27(1)
C(1)	7589(2)	5491(2)	6934(1)	16(1)
C(2)	7942(2)	5386(2)	7661(1)	17(1)
C(3)	6698(2)	5591(2)	8078(1)	17(1)
C(4)	5544(2)	4780(2)	7856(1)	16(1)
C(5)	5199(2)	4955(2)	7113(1)	15(1)
C(6)	6498(2)	4616(2)	6727(1)	15(1)
C(7)	6298(2)	4555(2)	5979(1)	16(1)
C(8)	7579(2)	4155(2)	5624(1)	21(1)
C(9)	7370(2)	4183(2)	4874(1)	22(1)
C(10)	6227(2)	3316(2)	4694(1)	24(1)
C(11)	4962(2)	3551(2)	5084(1)	21(1)
C(12)	5145(2)	3675(2)	5821(1)	18(1)
C(13)	3850(2)	3993(2)	6176(1)	20(1)
C(14)	4077(2)	4078(2)	6919(1)	19(1)
C(15)	4778(2)	6255(2)	6963(1)	17(1)
C(16)	4347(2)	5010(2)	8295(1)	18(1)
C(17)	7173(2)	5469(2)	4631(1)	21(1)
C(18)	8183(2)	6316(2)	4738(1)	28(1)
C(19)	8047(2)	7497(2)	4529(1)	33(1)
C(20)	6904(2)	7862(2)	4199(1)	33(1)
C(21)	5894(2)	7032(2)	4078(1)	29(1)
C(22)	6031(2)	5845(2)	4296(1)	23(1)
C(23)	10171(2)	6099(2)	7650(1)	23(1)
C(24)	11041(2)	7137(2)	7847(1)	32(1)
C(25)	2599(2)	4099(2)	8892(1)	36(1)

Table S18.3. Bond lengths [Å] and angles [°] for Shenvi305.

O(1)-C(1)	1.211(2)	C(19)-H(19)	0.9500
O(2)-C(2)	1.436(2)	C(19)-C(20)	1.387(4)
O(2)-C(23)	1.357(2)	C(20)-H(20)	0.9500
O(3)-C(23)	1.201(3)	C(20)-C(21)	1.391(3)
O(4)-C(16)	1.337(2)	C(21)-H(21)	0.9500
O(4)-C(25)	1.446(2)	C(21)-C(22)	1.400(3)
O(5)-C(16)	1.210(2)	C(22)-H(22)	0.9500
O(6)-C(11)	1.216(3)	C(23)-C(24)	1.501(3)
C(1)-C(2)	1.529(2)	C(24)-H(24B)	0.9800
C(1)-C(6)	1.522(2)	C(24)-H(24A)	0.9800
C(2)-H(2)	1.0000	C(24)-H(24C)	0.9800
C(2)-C(3)	1.524(2)	C(25)-H(25A)	0.9800
C(3)-H(3A)	0.9900	C(25)-H(25B)	0.9800
C(3)-H(3B)	0.9900	C(25)-H(25C)	0.9800
C(3)-C(4)	1.534(2)		
C(4)-H(4)	1.0000	C(23)-O(2)-C(2)	115.83(14)
C(4)-C(5)	1.566(2)	C(16)-O(4)-C(25)	115.86(15)
C(4)-C(16)	1.516(3)	O(1)-C(1)-C(2)	122.25(16)
C(5)-C(6)	1.565(2)	O(1)-C(1)-C(6)	124.87(16)
C(5)-C(14)	1.539(2)	C(6)-C(1)-C(2)	112.72(14)
C(5)-C(15)	1.538(2)	O(2)-C(2)-C(1)	110.82(14)
C(6)-H(6)	1.0000	O(2)-C(2)-H(2)	109.7
C(6)-C(7)	1.540(2)	O(2)-C(2)-C(3)	106.74(14)
C(7)-H(7)	1.0000	C(1)-C(2)-H(2)	109.7
C(7)-C(8)	1.536(2)	C(3)-C(2)-C(1)	110.00(14)
C(7)-C(12)	1.547(2)	C(3)-C(2)-H(2)	109.7
C(8)-H(8A)	0.9900	C(2)-C(3)-H(3A)	109.4
C(8)-H(8B)	0.9900	C(2)-C(3)-H(3B)	109.4
C(8)-C(9)	1.545(2)	C(2)-C(3)-C(4)	111.12(14)
C(9)-H(9)	1.0000	H(3A)-C(3)-H(3B)	108.0
C(9)-C(10)	1.540(3)	C(4)-C(3)-H(3A)	109.4
C(9)-C(17)	1.528(3)	C(4)-C(3)-H(3B)	109.4
C(10)-H(10A)	0.9900	C(3)-C(4)-H(4)	107.9
C(10)-H(10B)	0.9900	C(3)-C(4)-C(5)	112.26(14)
C(10)-C(11)	1.517(3)	C(5)-C(4)-H(4)	107.9
C(11)-C(12)	1.520(2)	C(16)-C(4)-C(3)	108.60(14)
C(12)-H(12)	1.0000	C(16)-C(4)-H(4)	107.9
C(12)-C(13)	1.526(3)	C(16)-C(4)-C(5)	112.17(14)
C(13)-H(13A)	0.9900	C(6)-C(5)-C(4)	105.96(13)
C(13)-H(13B)	0.9900	C(14)-C(5)-C(4)	109.29(14)
C(13)-C(14)	1.535(2)	C(14)-C(5)-C(6)	108.80(13)
C(14)-H(14A)	0.9900	C(15)-C(5)-C(4)	111.67(14)
C(14)-H(14B)	0.9900	C(15)-C(5)-C(6)	110.77(14)
C(15)-H(15A)	0.9800	C(15)-C(5)-C(14)	110.23(14)
C(15)-H(15B)	0.9800	C(1)-C(6)-C(5)	107.43(13)
C(15)-H(15C)	0.9800	C(1)-C(6)-H(6)	107.3
C(17)-C(18)	1.398(3)	C(1)-C(6)-C(7)	113.35(14)
C(17)-C(22)	1.395(3)	C(5)-C(6)-H(6)	107.3
C(18)-H(18)	0.9500	C(7)-C(6)-C(5)	113.68(14)
C(18)-C(19)	1.389(3)	C(7)-C(6)-H(6)	107.3

C(6)-C(7)-H(7)	108.6	H(14A)-C(14)-H(14B)	107.7
C(6)-C(7)-C(12)	109.31(14)	C(5)-C(15)-H(15A)	109.5
C(8)-C(7)-C(6)	111.80(14)	C(5)-C(15)-H(15B)	109.5
C(8)-C(7)-H(7)	108.6	C(5)-C(15)-H(15C)	109.5
C(8)-C(7)-C(12)	109.77(15)	H(15A)-C(15)-H(15B)	109.5
C(12)-C(7)-H(7)	108.6	H(15A)-C(15)-H(15C)	109.5
C(7)-C(8)-H(8A)	109.6	H(15B)-C(15)-H(15C)	109.5
C(7)-C(8)-H(8B)	109.6	O(4)-C(16)-C(4)	111.33(15)
C(7)-C(8)-C(9)	110.38(15)	O(5)-C(16)-O(4)	123.75(17)
H(8A)-C(8)-H(8B)	108.1	O(5)-C(16)-C(4)	124.93(16)
C(9)-C(8)-H(8A)	109.6	C(18)-C(17)-C(9)	119.22(18)
C(9)-C(8)-H(8B)	109.6	C(22)-C(17)-C(9)	123.06(18)
C(8)-C(9)-H(9)	107.4	C(22)-C(17)-C(18)	117.71(19)
C(10)-C(9)-C(8)	108.88(16)	C(17)-C(18)-H(18)	119.3
C(10)-C(9)-H(9)	107.4	C(19)-C(18)-C(17)	121.3(2)
C(17)-C(9)-C(8)	111.00(15)	C(19)-C(18)-H(18)	119.3
C(17)-C(9)-H(9)	107.4	C(18)-C(19)-H(19)	119.8
C(17)-C(9)-C(10)	114.49(16)	C(20)-C(19)-C(18)	120.5(2)
C(9)-C(10)-H(10A)	109.1	C(20)-C(19)-H(19)	119.8
C(9)-C(10)-H(10B)	109.1	C(19)-C(20)-H(20)	120.4
H(10A)-C(10)-H(10B)	107.8	C(19)-C(20)-C(21)	119.3(2)
C(11)-C(10)-C(9)	112.64(15)	C(21)-C(20)-H(20)	120.4
C(11)-C(10)-H(10A)	109.1	C(20)-C(21)-H(21)	120.0
C(11)-C(10)-H(10B)	109.1	C(20)-C(21)-C(22)	120.0(2)
O(6)-C(11)-C(10)	122.15(17)	C(22)-C(21)-H(21)	120.0
O(6)-C(11)-C(12)	122.10(18)	C(17)-C(22)-C(21)	121.2(2)
C(10)-C(11)-C(12)	115.74(17)	C(17)-C(22)-H(22)	119.4
C(7)-C(12)-H(12)	106.7	C(21)-C(22)-H(22)	119.4
C(11)-C(12)-C(7)	110.68(15)	O(2)-C(23)-C(24)	110.20(17)
C(11)-C(12)-H(12)	106.7	O(3)-C(23)-O(2)	123.60(18)
C(11)-C(12)-C(13)	112.88(16)	O(3)-C(23)-C(24)	126.18(19)
C(13)-C(12)-C(7)	112.71(14)	C(23)-C(24)-H(24B)	109.5
C(13)-C(12)-H(12)	106.7	C(23)-C(24)-H(24A)	109.5
C(12)-C(13)-H(13A)	109.4	C(23)-C(24)-H(24C)	109.5
C(12)-C(13)-H(13B)	109.4	H(24B)-C(24)-H(24A)	109.5
C(12)-C(13)-C(14)	110.99(15)	H(24B)-C(24)-H(24C)	109.5
H(13A)-C(13)-H(13B)	108.0	H(24A)-C(24)-H(24C)	109.5
C(14)-C(13)-H(13A)	109.4	O(4)-C(25)-H(25A)	109.5
C(14)-C(13)-H(13B)	109.4	O(4)-C(25)-H(25B)	109.5
C(5)-C(14)-H(14A)	108.8	O(4)-C(25)-H(25C)	109.5
C(5)-C(14)-H(14B)	108.8	H(25A)-C(25)-H(25B)	109.5
C(13)-C(14)-C(5)	113.63(14)	H(25A)-C(25)-H(25C)	109.5
C(13)-C(14)-H(14A)	108.8	H(25B)-C(25)-H(25C)	109.5
C(13)-C(14)-H(14B)	108.8		

Table S18.4. Anisotropic displacement parameters ($Å^2$x 10^3) for Shenvi305. The anisotropic displacement factor exponent takes the form: $-2\pi^2[h^2 a^{*2}U^{11} + ... + 2 h k a^* b^* U^{12}]$

	U^{11}	U^{22}	U^{33}	U^{23}	U^{13}	U^{12}
O(1)	21(1)	25(1)	21(1)	3(1)	1(1)	-6(1)
O(2)	16(1)	18(1)	22(1)	-2(1)	-3(1)	-3(1)
O(3)	20(1)	31(1)	53(1)	-12(1)	4(1)	0(1)
O(4)	26(1)	20(1)	28(1)	0(1)	12(1)	-3(1)
O(5)	27(1)	20(1)	31(1)	-6(1)	9(1)	0(1)
O(6)	30(1)	28(1)	22(1)	2(1)	-9(1)	-7(1)
C(1)	13(1)	17(1)	17(1)	-1(1)	1(1)	3(1)
C(2)	15(1)	16(1)	19(1)	0(1)	-5(1)	-2(1)
C(3)	18(1)	18(1)	15(1)	0(1)	-3(1)	0(1)
C(4)	18(1)	13(1)	16(1)	1(1)	0(1)	0(1)
C(5)	14(1)	14(1)	16(1)	0(1)	-1(1)	0(1)
C(6)	16(1)	13(1)	15(1)	0(1)	-1(1)	2(1)
C(7)	16(1)	16(1)	15(1)	-1(1)	-3(1)	1(1)
C(8)	20(1)	26(1)	17(1)	-1(1)	-1(1)	7(1)
C(9)	23(1)	27(1)	15(1)	-3(1)	1(1)	8(1)
C(10)	35(1)	20(1)	17(1)	-3(1)	-4(1)	6(1)
C(11)	29(1)	13(1)	20(1)	0(1)	-5(1)	-2(1)
C(12)	22(1)	13(1)	18(1)	0(1)	-5(1)	1(1)
C(13)	18(1)	21(1)	22(1)	-1(1)	-5(1)	-5(1)
C(14)	17(1)	19(1)	20(1)	0(1)	-1(1)	-4(1)
C(15)	17(1)	16(1)	18(1)	1(1)	-1(1)	2(1)
C(16)	19(1)	18(1)	17(1)	-1(1)	-1(1)	-3(1)
C(17)	21(1)	27(1)	13(1)	-1(1)	4(1)	2(1)
C(18)	24(1)	43(1)	17(1)	3(1)	0(1)	-5(1)
C(19)	36(1)	39(1)	25(1)	5(1)	1(1)	-16(1)
C(20)	38(1)	27(1)	34(1)	7(1)	7(1)	-5(1)
C(21)	24(1)	30(1)	34(1)	10(1)	4(1)	1(1)
C(22)	20(1)	25(1)	25(1)	2(1)	3(1)	0(1)
C(23)	16(1)	24(1)	29(1)	0(1)	-4(1)	-1(1)
C(24)	22(1)	27(1)	49(1)	-2(1)	-5(1)	-6(1)
C(25)	34(1)	32(1)	41(1)	-1(1)	23(1)	-6(1)

X-ray Structure of analog **32** (CCDC 2267254)

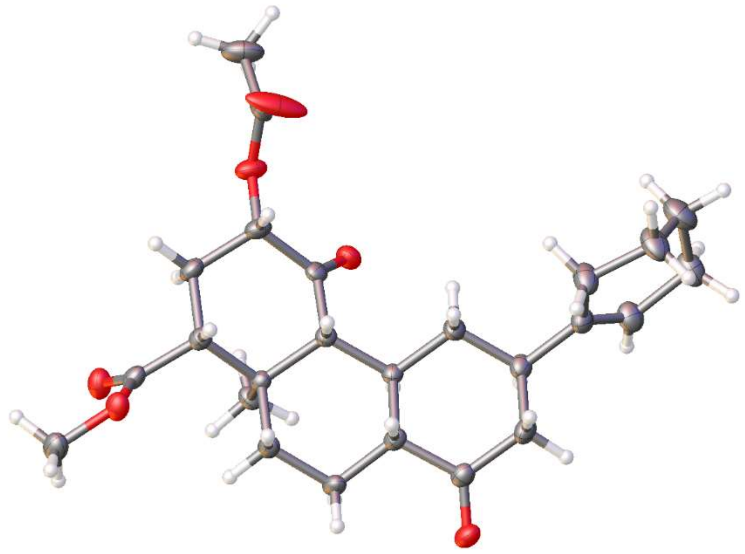

Experimental Summary

The single crystal X-ray diffraction studies were carried out on a Bruker SMART Pt135 CCD diffractometer equipped with Cu K_{α} radiation (λ = 1.54178 Å).

Crystals of the subject compound were used as received. A 0.27 x 0.09 x 0.04 mm piece of a crystal was mounted on a Cryoloop with Paratone oil. Data were collected in a nitrogen gas stream at 100(2) K using ϕ and ω scans. Crystal-to-detector distance was 45 mm and exposure time was 5, 10, 15, or 30 seconds depending on the 2θ range per frame using a scan width of 1.25°. Data collection was 98.3 % complete to 67.679° in θ. A total of 20228 reflections were collected covering the indices, −38<=h<=38, −7<=k<=7, −15<=l<=15. 4286 reflections were found to be symmetry independent, with a R_{int} of 0.0440. Indexing and unit cell refinement indicated a **C**-centered, **Orthorhombic** lattice. The space group was found to be ***C*2**. The data were integrated using the Bruker SAINT Software program and scaled using the SADABS software program. Solution by direct methods (SHELXT) produced a complete phasing model consistent with the proposed structure.

All nonhydrogen atoms were refined anisotropically by full-matrix least-squares (SHELXL-2014). All carbon bonded hydrogen atoms were placed using a riding model. Their positions were constrained relative to their parent atom using the appropriate HFIX command in SHELXL-2014. Crystallographic data are summarized in Table S19.1.

Notes: Absolute stereochemistry could not be conclusively assigned (Absolute Structure Parameter = 0.23(12)). However, the stereochemistry could be established based on the synthesis. There is a minor positional disorder on one of the esters. There is one copy of the compound in the asymmetric unit. The chemical formula for the compound is: $C_{25}H_{34}O_6$

Table S19.1. Crystal data and structure refinement for Shenvi304.

Identification code	shenvi304_0m_a	
Empirical formula	C25 H34 O6	
Formula weight	430.52	
Temperature	100.15 K	
Wavelength	1.54178 Å	
Crystal system	Monoclinic	
Space group	C 1 2 1	
Unit cell dimensions	a = 31.806(3) Å	□= 90°.
	b = 5.9304(5) Å	□= 106.701(5)°.
	c = 12.6356(11) Å	□ = 90°.
Volume	2282.8(3) $Å^3$	
Z	4	
Density (calculated)	1.253 Mg/m^3	
Absorption coefficient	0.717 mm^{-1}	
F(000)	928	
Crystal size	0.27 x 0.09 x 0.04 mm^3	
Theta range for data collection	2.901 to 71.598°.	
Index ranges	-38<=h<=38, -7<=k<=7, -15<=l<=15	
Reflections collected	20228	
Independent reflections	4286 [R(int) = 0.0440]	
Completeness to theta = 67.679°	98.3 %	
Absorption correction	Semi-empirical from equivalents	
Max. and min. transmission	0.5220 and 0.4276	
Refinement method	Full-matrix least-squares on F^2	
Data / restraints / parameters	4286 / 4 / 302	
Goodness-of-fit on F^2	1.067	
Final R indices [I>2sigma(I)]	R1 = 0.0369, wR2 = 0.0988	
R indices (all data)	R1 = 0.0390, wR2 = 0.1025	
Absolute structure parameter	0.23(12)	
Largest diff. peak and hole	0.236 and -0.171 e.$Å^{-3}$	

Table S19.2. Atomic coordinates (x 10^4) and equivalent isotropic displacement parameters (Å^{2}x 10^3) for Shenvi304. U(eq) is defined as one third of the trace of the orthogonalized U^{ij} tensor.

	x	y	z	U(eq)
O(6)	5891(1)	8532(3)	4278(1)	29(1)
O(5)	7576(1)	2342(3)	5496(2)	30(1)
O(3)	5562(1)	4156(3)	182(1)	32(1)
O(4)	5561(1)	597(3)	785(1)	29(1)
O(2)	5032(1)	8169(3)	3216(2)	33(1)
C(6)	5571(1)	2849(4)	924(2)	25(1)
C(12)	6793(1)	2749(4)	4792(2)	23(1)
C(23)	6520(1)	4904(4)	4822(2)	21(1)
C(8)	6079(1)	4101(4)	2777(2)	20(1)
C(9)	6254(1)	6122(4)	2268(2)	23(1)
C(25)	5768(1)	6673(4)	3914(2)	23(1)
C(24)	6066(1)	4638(4)	3987(2)	20(1)
C(13)	7232(1)	2794(4)	5682(2)	25(1)
C(11)	6836(1)	2306(4)	3634(2)	26(1)
C(22)	6487(1)	5294(4)	5998(2)	25(1)
C(16)	6906(1)	6120(5)	7982(2)	29(1)
C(15)	6939(1)	5525(4)	6834(2)	26(1)
C(4)	5271(1)	5412(4)	2091(2)	27(1)
C(10)	6380(1)	2045(4)	2818(2)	24(1)
C(3)	5295(1)	6170(4)	3268(2)	27(1)
C(5)	5598(1)	3484(4)	2110(2)	24(1)
C(14)	7211(1)	3372(5)	6837(2)	29(1)
C(7)	5550(1)	-166(5)	-315(2)	34(1)
C(21)	6598(1)	4725(6)	8429(2)	39(1)
C(17)	7137(1)	7820(5)	8560(2)	34(1)
C(20)	6650(1)	5154(6)	9661(2)	43(1)
C(18)	7103(1)	8599(5)	9674(2)	41(1)
C(19)	6696(1)	7670(6)	9907(3)	48(1)
C(2A)	4710(5)	7990(20)	3709(10)	28(3)
O(1A)	4640(6)	6349(19)	4183(15)	79(5)
C(2)	4632(4)	7960(17)	3334(15)	38(3)
O(1)	4486(2)	6177(10)	3395(18)	87(6)
C(1)	4425(1)	10150(6)	3447(3)	51(1)

Table S19.3. Bond lengths [Å] and angles [°] for Shenvi304.

O(6)-C(25)	1.215(3)	C(7)-H(7B)	0.9800
O(5)-C(13)	1.214(3)	C(7)-H(7C)	0.9800
O(3)-C(6)	1.210(3)	C(21)-H(21A)	0.9900
O(4)-C(6)	1.346(3)	C(21)-H(21B)	0.9900
O(4)-C(7)	1.453(3)	C(21)-C(20)	1.540(4)
O(2)-C(3)	1.441(3)	C(17)-H(17)	0.9500
O(2)-C(2A)	1.346(11)	C(17)-C(18)	1.515(4)
O(2)-C(2)	1.327(10)	C(20)-H(20A)	0.9900
C(6)-C(5)	1.523(3)	C(20)-H(20B)	0.9900
C(12)-H(12)	1.0000	C(20)-C(19)	1.522(5)
C(12)-C(23)	1.552(3)	C(18)-H(18A)	0.9900
C(12)-C(13)	1.520(3)	C(18)-H(18B)	0.9900
C(12)-C(11)	1.531(3)	C(18)-C(19)	1.511(5)
C(23)-H(23)	1.0000	C(19)-H(19A)	0.9900
C(23)-C(24)	1.532(3)	C(19)-H(19B)	0.9900
C(23)-C(22)	1.537(3)	C(2A)-O(1A)	1.195(12)
C(8)-C(9)	1.537(3)	C(2A)-C(1)	1.551(11)
C(8)-C(24)	1.574(3)	C(2)-O(1)	1.167(11)
C(8)-C(10)	1.541(3)	C(2)-C(1)	1.482(10)
C(8)-C(5)	1.563(3)	C(1)-H(1AA)	0.9800
C(9)-H(9A)	0.9800	C(1)-H(1AB)	0.9800
C(9)-H(9B)	0.9800	C(1)-H(1AC)	0.9800
C(9)-H(9C)	0.9800	C(1)-H(1BD)	0.9800
C(25)-C(24)	1.522(3)	C(1)-H(1BE)	0.9800
C(25)-C(3)	1.521(3)	C(1)-H(1BF)	0.9800
C(24)-H(24)	1.0000		
C(13)-C(14)	1.519(3)	C(6)-O(4)-C(7)	115.2(2)
C(11)-H(11A)	0.9900	C(2A)-O(2)-C(3)	114.9(5)
C(11)-H(11B)	0.9900	C(2)-O(2)-C(3)	118.6(5)
C(11)-C(10)	1.526(3)	O(3)-C(6)-O(4)	122.7(2)
C(22)-H(22A)	0.9900	O(3)-C(6)-C(5)	125.8(2)
C(22)-H(22B)	0.9900	O(4)-C(6)-C(5)	111.5(2)
C(22)-C(15)	1.526(3)	C(23)-C(12)-H(12)	106.6
C(16)-C(15)	1.526(3)	C(13)-C(12)-H(12)	106.6
C(16)-C(21)	1.507(4)	C(13)-C(12)-C(23)	111.64(19)
C(16)-C(17)	1.335(4)	C(13)-C(12)-C(11)	113.28(19)
C(15)-H(15)	1.0000	C(11)-C(12)-H(12)	106.6
C(15)-C(14)	1.542(4)	C(11)-C(12)-C(23)	111.66(19)
C(4)-H(4A)	0.9900	C(12)-C(23)-H(23)	108.8
C(4)-H(4B)	0.9900	C(24)-C(23)-C(12)	108.76(18)
C(4)-C(3)	1.535(3)	C(24)-C(23)-H(23)	108.8
C(4)-C(5)	1.541(3)	C(24)-C(23)-C(22)	111.62(18)
C(10)-H(10A)	0.9900	C(22)-C(23)-C(12)	109.99(19)
C(10)-H(10B)	0.9900	C(22)-C(23)-H(23)	108.8
C(3)-H(3)	1.0000	C(9)-C(8)-C(24)	111.21(18)
C(5)-H(5)	1.0000	C(9)-C(8)-C(10)	109.61(18)
C(14)-H(14A)	0.9900	C(9)-C(8)-C(5)	111.82(19)
C(14)-H(14B)	0.9900	C(10)-C(8)-C(24)	108.45(18)
C(7)-H(7A)	0.9800	C(10)-C(8)-C(5)	109.58(17)

C(5)-C(8)-C(24)	106.06(17)
C(8)-C(9)-H(9A)	109.5
C(8)-C(9)-H(9B)	109.5
C(8)-C(9)-H(9C)	109.5
H(9A)-C(9)-H(9B)	109.5
H(9A)-C(9)-H(9C)	109.5
H(9B)-C(9)-H(9C)	109.5
O(6)-C(25)-C(24)	124.6(2)
O(6)-C(25)-C(3)	122.9(2)
C(3)-C(25)-C(24)	112.3(2)
C(23)-C(24)-C(8)	114.00(17)
C(23)-C(24)-H(24)	107.1
C(8)-C(24)-H(24)	107.1
C(25)-C(24)-C(23)	114.09(19)
C(25)-C(24)-C(8)	106.96(18)
C(25)-C(24)-H(24)	107.1
O(5)-C(13)-C(12)	122.7(2)
O(5)-C(13)-C(14)	121.8(2)
C(14)-C(13)-C(12)	115.5(2)
C(12)-C(11)-H(11A)	109.8
C(12)-C(11)-H(11B)	109.8
H(11A)-C(11)-H(11B)	108.2
C(10)-C(11)-C(12)	109.53(19)
C(10)-C(11)-H(11A)	109.8
C(10)-C(11)-H(11B)	109.8
C(23)-C(22)-H(22A)	109.3
C(23)-C(22)-H(22B)	109.3
H(22A)-C(22)-H(22B)	107.9
C(15)-C(22)-C(23)	111.76(18)
C(15)-C(22)-H(22A)	109.3
C(15)-C(22)-H(22B)	109.3
C(21)-C(16)-C(15)	117.3(2)
C(17)-C(16)-C(15)	121.0(2)
C(17)-C(16)-C(21)	121.7(2)
C(22)-C(15)-C(16)	111.70(19)
C(22)-C(15)-H(15)	107.4
C(22)-C(15)-C(14)	110.1(2)
C(16)-C(15)-H(15)	107.4
C(16)-C(15)-C(14)	112.5(2)
C(14)-C(15)-H(15)	107.4
H(4A)-C(4)-H(4B)	108.0
C(3)-C(4)-H(4A)	109.4
C(3)-C(4)-H(4B)	109.4
C(3)-C(4)-C(5)	110.97(19)
C(5)-C(4)-H(4A)	109.4
C(5)-C(4)-H(4B)	109.4
C(8)-C(10)-H(10A)	108.8
C(8)-C(10)-H(10B)	108.8
C(11)-C(10)-C(8)	113.78(18)
C(11)-C(10)-H(10A)	108.8
C(11)-C(10)-H(10B)	108.8
H(10A)-C(10)-H(10B)	107.7
O(2)-C(3)-C(25)	109.5(2)
O(2)-C(3)-C(4)	109.3(2)
O(2)-C(3)-H(3)	109.3
C(25)-C(3)-C(4)	110.03(18)
C(25)-C(3)-H(3)	109.3
C(4)-C(3)-H(3)	109.3
C(6)-C(5)-C(8)	111.03(18)
C(6)-C(5)-C(4)	108.72(19)
C(6)-C(5)-H(5)	108.1
C(8)-C(5)-H(5)	108.1
C(4)-C(5)-C(8)	112.72(19)
C(4)-C(5)-H(5)	108.1
C(13)-C(14)-C(15)	111.37(19)
C(13)-C(14)-H(14A)	109.4
C(13)-C(14)-H(14B)	109.4
C(15)-C(14)-H(14A)	109.4
C(15)-C(14)-H(14B)	109.4
H(14A)-C(14)-H(14B)	108.0
O(4)-C(7)-H(7A)	109.5
O(4)-C(7)-H(7B)	109.5
O(4)-C(7)-H(7C)	109.5
H(7A)-C(7)-H(7B)	109.5
H(7A)-C(7)-H(7C)	109.5
H(7B)-C(7)-H(7C)	109.5
C(16)-C(21)-H(21A)	108.9
C(16)-C(21)-H(21B)	108.9
C(16)-C(21)-C(20)	113.4(2)
H(21A)-C(21)-H(21B)	107.7
C(20)-C(21)-H(21A)	108.9
C(20)-C(21)-H(21B)	108.9
C(16)-C(17)-H(17)	117.9
C(16)-C(17)-C(18)	124.2(2)
C(18)-C(17)-H(17)	117.9
C(21)-C(20)-H(20A)	109.6
C(21)-C(20)-H(20B)	109.6
H(20A)-C(20)-H(20B)	108.1
C(19)-C(20)-C(21)	110.1(3)
C(19)-C(20)-H(20A)	109.6
C(19)-C(20)-H(20B)	109.6
C(17)-C(18)-H(18A)	109.3
C(17)-C(18)-H(18B)	109.3
H(18A)-C(18)-H(18B)	108.0
C(19)-C(18)-C(17)	111.4(3)
C(19)-C(18)-H(18A)	109.3
C(19)-C(18)-H(18B)	109.3
C(20)-C(19)-H(19A)	109.5
C(20)-C(19)-H(19B)	109.5
C(18)-C(19)-C(20)	110.8(3)
C(18)-C(19)-H(19A)	109.5
C(18)-C(19)-H(19B)	109.5

H(19A)-C(19)-H(19B)	108.1
O(2)-C(2A)-C(1)	108.0(7)
O(1A)-C(2A)-O(2)	125.0(10)
O(1A)-C(2A)-C(1)	126.7(10)
O(2)-C(2)-C(1)	113.3(7)
O(1)-C(2)-O(2)	120.3(9)
O(1)-C(2)-C(1)	126.3(8)
C(2A)-C(1)-H(1AA)	109.5
C(2A)-C(1)-H(1AB)	109.5
C(2A)-C(1)-H(1AC)	109.5
C(2)-C(1)-H(1BD)	109.5
C(2)-C(1)-H(1BE)	109.5
C(2)-C(1)-H(1BF)	109.5
H(1AA)-C(1)-H(1AB)	109.5
H(1AA)-C(1)-H(1AC)	109.5
H(1AB)-C(1)-H(1AC)	109.5
H(1BD)-C(1)-H(1BE)	109.5
H(1BD)-C(1)-H(1BF)	109.5
H(1BE)-C(1)-H(1BF)	109.5

Symmetry transformations used to generate equivalent atoms:

Table S19.4. Anisotropic displacement parameters ($Å^2$x 10^3) for Shenvi304. The anisotropic displacement factor exponent takes the form: -2□$^2[h^2 a^{*2}U^{11} + ... + 2 h k a^* b^* U^{12}]$

	U^{11}	U^{22}	U^{33}	U^{23}	U^{13}	U^{12}
O(6)	32(1)	30(1)	26(1)	-5(1)	9(1)	6(1)
O(5)	21(1)	32(1)	34(1)	-4(1)	3(1)	4(1)
O(3)	38(1)	30(1)	25(1)	0(1)	2(1)	2(1)
O(4)	32(1)	24(1)	28(1)	-3(1)	2(1)	-1(1)
O(2)	25(1)	38(1)	41(1)	7(1)	15(1)	9(1)
C(6)	18(1)	25(1)	28(1)	-1(1)	0(1)	1(1)
C(12)	19(1)	23(1)	24(1)	0(1)	4(1)	0(1)
C(23)	18(1)	26(1)	20(1)	0(1)	6(1)	0(1)
C(8)	17(1)	20(1)	21(1)	-2(1)	4(1)	0(1)
C(9)	27(1)	23(1)	21(1)	-1(1)	10(1)	-2(1)
C(25)	22(1)	32(1)	18(1)	2(1)	10(1)	2(1)
C(24)	19(1)	24(1)	20(1)	1(1)	7(1)	0(1)
C(13)	23(1)	23(1)	26(1)	0(1)	4(1)	1(1)
C(11)	21(1)	26(1)	28(1)	-6(1)	5(1)	5(1)
C(22)	23(1)	35(1)	19(1)	2(1)	8(1)	1(1)
C(16)	26(1)	39(1)	21(1)	3(1)	6(1)	2(1)
C(15)	24(1)	35(1)	20(1)	2(1)	7(1)	-2(1)
C(4)	17(1)	31(1)	30(1)	0(1)	2(1)	2(1)
C(10)	23(1)	22(1)	24(1)	-4(1)	4(1)	2(1)
C(3)	20(1)	30(1)	31(1)	3(1)	9(1)	5(1)
C(5)	20(1)	23(1)	26(1)	0(1)	5(1)	0(1)
C(14)	23(1)	38(1)	22(1)	4(1)	2(1)	2(1)
C(7)	35(1)	31(1)	31(1)	-7(1)	3(1)	4(1)
C(21)	37(2)	55(2)	25(1)	-3(1)	12(1)	-14(1)
C(17)	41(2)	34(1)	27(1)	3(1)	12(1)	-3(1)
C(20)	50(2)	57(2)	30(1)	-4(1)	22(1)	-15(2)
C(18)	57(2)	37(2)	32(1)	-5(1)	16(1)	-5(1)
C(19)	60(2)	55(2)	38(2)	-5(1)	29(2)	0(2)
C(2A)	31(5)	37(4)	17(5)	-4(3)	9(4)	8(3)
O(1A)	118(9)	51(4)	112(9)	38(5)	104(8)	39(5)
C(2)	21(4)	42(4)	50(7)	-14(4)	9(5)	-1(3)
O(1)	35(3)	36(3)	208(15)	-16(5)	65(5)	-7(2)

C(1)	45(2)	44(2)	73(2)	2(2)	34(2)	15(1)

Table S19.5. Hydrogen coordinates (x 10^4) and isotropic displacement parameters ($Å^2$x 10^3) for Shenvi304.

	x	y	z	U(eq)
H(12)	6624	1454	4970	27
H(23)	6671	6227	4602	25
H(9A)	6043	7368	2158	34
H(9B)	6293	5681	1556	34
H(9C)	6536	6606	2767	34
H(24)	5920	3332	4237	24
H(11A)	6992	3577	3405	31
H(11B)	7009	915	3639	31
H(22A)	6315	6681	6009	30
H(22B)	6329	4014	6212	30
H(15)	7093	6799	6585	31
H(4A)	5339	6705	1673	32
H(4B)	4970	4895	1709	32
H(10A)	6238	695	3023	28
H(10B)	6412	1787	2071	28
H(3)	5178	4942	3648	32
H(5)	5506	2146	2470	28
H(14A)	7512	3596	7328	34
H(14B)	7078	2098	7131	34
H(7A)	5270	271	-841	51
H(7B)	5580	-1810	-315	51
H(7C)	5792	528	-533	51
H(21A)	6653	3108	8324	46
H(21B)	6292	5062	7998	46
H(17)	7337	8589	8253	40
H(20A)	6390	4563	9853	52
H(20B)	6912	4352	10118	52
H(18A)	7366	8098	10259	50
H(18B)	7094	10267	9687	50
H(19A)	6434	8469	9446	58
H(19B)	6713	7933	10692	58
H(1AA)	4603	11401	3302	76
H(1AB)	4318	10527	4078	76
H(1AC)	4175	9891	2793	76
H(1BD)	4564	10781	4181	76
H(1BE)	4111	9923	3353	76
H(1BF)	4463	11196	2880	76

X-ray Structure of Deketalized C-allyl **SI 6** (CCDC2127086)

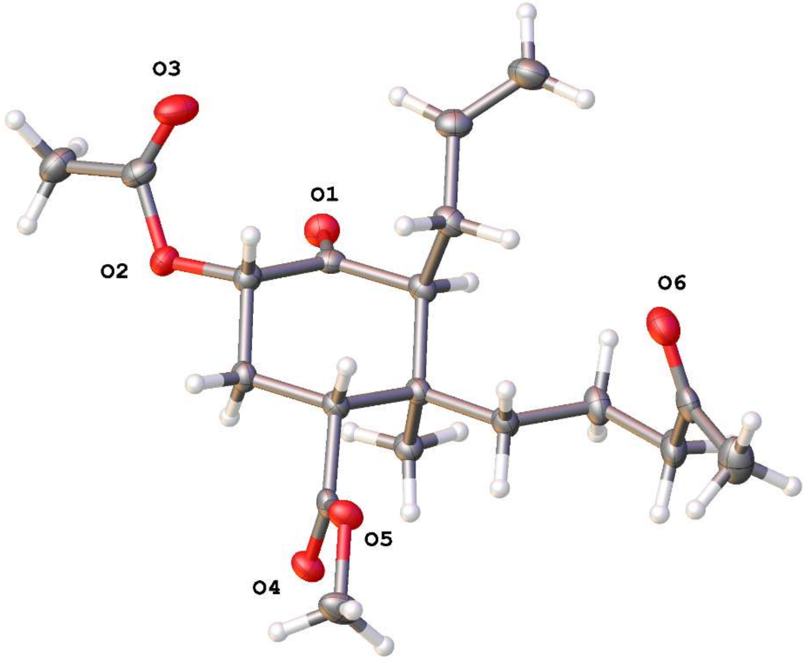

Experimental Summary

The single crystal X-ray diffraction studies were carried out on a Bruker SMART Pt135 CCD diffractometer equipped with Cu K_{α} radiation (λ =1.54178 Å).

Crystals of the subject compound were used as received. A 0.22 x 0.2 x 0.18 mm piece of a colorless crystal was mounted on a Cryoloop with Paratone oil. Data were collected in a nitrogen gas stream at 100(2) K using ϕ and ω scans. Crystal-to-detector distance was 45 mm and exposure time was 1, 2, 3 seconds depending on the 2 θ range per frame using a scan width of 1.6°. Data collection was 100 % complete to 67.684° in θ. A total of 15689 reflections were collected covering the indices, -11<=h<=11, -12<=k<=12, -12<=l<=12. 3884 reflections were found to be symmetry independent, with a R_{int} of 0.0308. Indexing and unit cell refinement indicated a **P**rimitive, **Monoclinic** lattice. The space group was found to be $\boldsymbol{P2_1}$. The data were integrated using the Bruker SAINT Software program and scaled using the SADABS software program. Solution by direct methods (SHELXT) produced a complete phasing model consistent with the proposed structure.

All nonhydrogen atoms were refined anisotropically by full-matrix least-squares (SHELXL-2014). All carbon bonded hydrogen atoms were placed using a riding model. Their positions were constrained relative to their parent atom using the appropriate HFIX command in SHELXL-2014. Crystallographic data are summarized in Table S20.1.

Notes: Absolute stereochemistry was conclusively assigned (Flack = -0.03(4)).

Table S20.1. Crystal data and structure refinement for Shenvi271.

Identification code	shenvi271
Empirical formula	C19 H28 O6
Formula weight	352.41
Temperature	100.15 K
Wavelength	1.54184 Å
Crystal system	Monoclinic
Space group	P 1 21 1
Unit cell dimensions	a = 9.650(2) Å α= 90°.
	b = 10.340(3) Å β= 100.387(12)°.
	c = 10.128(3) Å γ = 90°.
Volume	993.9(4) Å3
Z	2
Density (calculated)	1.178 Mg/m^3
Absorption coefficient	0.714 mm^{-1}
F(000)	380
Crystal size	0.22 x 0.2 x 0.18 mm^3
Theta range for data collection	4.438 to 72.572°.
Index ranges	-11<=h<=11, -12<=k<=12, -12<=l<=12
Reflections collected	15689
Independent reflections	3884 [R(int) = 0.0308]
Completeness to theta = 67.684°	100.0 %
Absorption correction	Semi-empirical from equivalents
Max. and min. transmission	0.7536 and 0.6751
Refinement method	Full-matrix least-squares on F^2
Data / restraints / parameters	3884 / 1 / 230
Goodness-of-fit on F^2	1.077
Final R indices [I>2sigma(I)]	R1 = 0.0277, wR2 = 0.0677
R indices (all data)	R1 = 0.0281, wR2 = 0.0682
Absolute structure parameter	-0.03(4)
Extinction coefficient	n/a
Largest diff. peak and hole	0.262 and -0.143 e.$Å^{-3}$

Table S20.2. Atomic coordinates (x 10^4) and equivalent isotropic displacement parameters ($Å^2$x 10^3) for Shenvi271. U(eq) is defined as one third of the trace of the orthogonalized U^{ij} tensor.

	x	y	z	U(eq)
O(2)	4283(1)	7309(1)	1400(1)	23(1)
O(5)	6087(1)	5024(1)	6661(1)	24(1)
O(6)	1376(1)	2068(1)	6173(1)	32(1)
O(4)	5504(1)	7132(1)	6594(1)	24(1)
O(1)	1582(1)	7124(1)	1685(1)	25(1)
O(3)	3523(2)	5996(1)	-366(1)	37(1)
C(1)	2384(2)	6405(2)	2404(2)	19(1)
C(2)	3948(2)	6335(2)	2311(2)	20(1)
C(6)	1926(2)	5545(2)	3473(2)	19(1)
C(4)	4431(2)	5746(2)	4767(2)	18(1)
C(10)	5380(2)	6064(2)	6093(2)	19(1)
C(8)	3952(2)	7037(2)	69(2)	25(1)
C(3)	4870(2)	6613(2)	3674(2)	20(1)
C(17)	1954(2)	4102(2)	3056(2)	22(1)
C(12)	2557(2)	4961(2)	5990(2)	21(1)
C(5)	2836(2)	5892(2)	4871(2)	18(1)
C(18)	1029(2)	3838(2)	1716(2)	26(1)
C(7)	2444(2)	7291(2)	5205(2)	22(1)
C(15)	1422(2)	2559(2)	7273(2)	26(1)
C(13)	1030(2)	4799(2)	6195(2)	27(1)
C(19)	-95(2)	3084(2)	1539(2)	36(1)
C(16)	1905(2)	1807(2)	8547(2)	40(1)
C(14)	987(2)	3951(2)	7427(2)	30(1)
C(9)	4212(2)	8191(2)	-748(2)	32(1)
C(11)	7004(2)	5274(2)	7938(2)	31(1)

Table S20.3. Bond lengths [Å] and angles [°] for Shenvi271.

O(2)-C(2)	1.4413(19)
O(2)-C(8)	1.358(2)
O(5)-C(10)	1.345(2)
O(5)-C(11)	1.453(2)
O(6)-C(15)	1.218(2)
O(4)-C(10)	1.212(2)
O(1)-C(1)	1.216(2)
O(3)-C(8)	1.207(2)
C(1)-C(2)	1.530(2)
C(1)-C(6)	1.527(2)
C(2)-H(2)	1.0000
C(2)-C(3)	1.529(2)
C(6)-H(6)	1.0000
C(6)-C(17)	1.553(2)
C(6)-C(5)	1.567(2)
C(4)-H(4)	1.0000
C(4)-C(10)	1.519(2)
C(4)-C(3)	1.541(2)
C(4)-C(5)	1.569(2)
C(8)-C(9)	1.499(3)
C(3)-H(3A)	0.9900
C(3)-H(3B)	0.9900
C(17)-H(17A)	0.9900
C(17)-H(17B)	0.9900
C(17)-C(18)	1.508(2)
C(12)-H(12A)	0.9900
C(12)-H(12B)	0.9900
C(12)-C(5)	1.547(2)
C(12)-C(13)	1.533(2)
C(5)-C(7)	1.548(2)
C(18)-H(18)	0.9500
C(18)-C(19)	1.321(3)
C(7)-H(7A)	0.9800
C(7)-H(7B)	0.9800
C(7)-H(7C)	0.9800
C(15)-C(16)	1.507(3)
C(15)-C(14)	1.515(3)
C(13)-H(13A)	0.9900
C(13)-H(13B)	0.9900
C(13)-C(14)	1.532(3)
C(19)-H(19A)	0.9500
C(19)-H(19B)	0.9500
C(16)-H(16A)	0.9800
C(16)-H(16B)	0.9800
C(16)-H(16C)	0.9800

C(14)-H(14A)	0.9900
C(14)-H(14B)	0.9900
C(9)-H(9A)	0.9800
C(9)-H(9B)	0.9800
C(9)-H(9C)	0.9800
C(11)-H(11A)	0.9800
C(11)-H(11B)	0.9800
C(11)-H(11C)	0.9800
C(8)-O(2)-C(2)	116.78(13)
C(10)-O(5)-C(11)	114.76(13)
O(1)-C(1)-C(2)	121.31(14)
O(1)-C(1)-C(6)	123.13(14)
C(6)-C(1)-C(2)	115.54(13)
O(2)-C(2)-C(1)	110.15(12)
O(2)-C(2)-H(2)	109.8
O(2)-C(2)-C(3)	106.57(12)
C(1)-C(2)-H(2)	109.8
C(3)-C(2)-C(1)	110.80(12)
C(3)-C(2)-H(2)	109.8
C(1)-C(6)-H(6)	107.3
C(1)-C(6)-C(17)	110.19(13)
C(1)-C(6)-C(5)	108.68(12)
C(17)-C(6)-H(6)	107.3
C(17)-C(6)-C(5)	115.61(13)
C(5)-C(6)-H(6)	107.3
C(10)-C(4)-H(4)	108.1
C(10)-C(4)-C(3)	108.00(12)
C(10)-C(4)-C(5)	111.20(12)
C(3)-C(4)-H(4)	108.1
C(3)-C(4)-C(5)	113.08(12)
C(5)-C(4)-H(4)	108.1
O(5)-C(10)-C(4)	112.54(13)
O(4)-C(10)-O(5)	123.05(14)
O(4)-C(10)-C(4)	124.41(14)
O(2)-C(8)-C(9)	110.45(15)
O(3)-C(8)-O(2)	123.42(16)
O(3)-C(8)-C(9)	126.12(16)
C(2)-C(3)-C(4)	110.39(12)
C(2)-C(3)-H(3A)	109.6
C(2)-C(3)-H(3B)	109.6
C(4)-C(3)-H(3A)	109.6
C(4)-C(3)-H(3B)	109.6
H(3A)-C(3)-H(3B)	108.1
C(6)-C(17)-H(17A)	109.1

C(6)-C(17)-H(17B)	109.1
H(17A)-C(17)-H(17B)	107.8
C(18)-C(17)-C(6)	112.47(13)
C(18)-C(17)-H(17A)	109.1
C(18)-C(17)-H(17B)	109.1
H(12A)-C(12)-H(12B)	107.2
C(5)-C(12)-H(12A)	107.9
C(5)-C(12)-H(12B)	107.9
C(13)-C(12)-H(12A)	107.9
C(13)-C(12)-H(12B)	107.9
C(13)-C(12)-C(5)	117.73(13)
C(6)-C(5)-C(4)	108.26(12)
C(12)-C(5)-C(6)	112.04(12)
C(12)-C(5)-C(4)	106.96(12)
C(12)-C(5)-C(7)	109.89(12)
C(7)-C(5)-C(6)	107.01(12)
C(7)-C(5)-C(4)	112.74(12)
C(17)-C(18)-H(18)	117.8
C(19)-C(18)-C(17)	124.49(17)
C(19)-C(18)-H(18)	117.8
C(5)-C(7)-H(7A)	109.5
C(5)-C(7)-H(7B)	109.5
C(5)-C(7)-H(7C)	109.5
H(7A)-C(7)-H(7B)	109.5
H(7A)-C(7)-H(7C)	109.5
H(7B)-C(7)-H(7C)	109.5
O(6)-C(15)-C(16)	121.56(18)
O(6)-C(15)-C(14)	121.67(18)
C(16)-C(15)-C(14)	116.77(17)
C(12)-C(13)-H(13A)	109.7
C(12)-C(13)-H(13B)	109.7
H(13A)-C(13)-H(13B)	108.2
C(14)-C(13)-C(12)	109.91(14)
C(14)-C(13)-H(13A)	109.7
C(14)-C(13)-H(13B)	109.7
C(18)-C(19)-H(19A)	120.0
C(18)-C(19)-H(19B)	120.0
H(19A)-C(19)-H(19B)	120.0
C(15)-C(16)-H(16A)	109.5
C(15)-C(16)-H(16B)	109.5
C(15)-C(16)-H(16C)	109.5
H(16A)-C(16)-H(16B)	109.5
H(16A)-C(16)-H(16C)	109.5
H(16B)-C(16)-H(16C)	109.5
C(15)-C(14)-C(13)	114.28(15)
C(15)-C(14)-H(14A)	108.7
C(15)-C(14)-H(14B)	108.7
C(13)-C(14)-H(14A)	108.7
C(13)-C(14)-H(14B)	108.7
H(14A)-C(14)-H(14B)	107.6
C(8)-C(9)-H(9A)	109.5
C(8)-C(9)-H(9B)	109.5
C(8)-C(9)-H(9C)	109.5
H(9A)-C(9)-H(9B)	109.5
H(9A)-C(9)-H(9C)	109.5
H(9B)-C(9)-H(9C)	109.5
O(5)-C(11)-H(11A)	109.5
O(5)-C(11)-H(11B)	109.5
O(5)-C(11)-H(11C)	109.5
H(11A)-C(11)-H(11B)	109.5
H(11A)-C(11)-H(11C)	109.5
H(11B)-C(11)-H(11C)	109.5

Table S20.4. Anisotropic displacement parameters ($Å^2 x 10^3$) for Shenvi271. The anisotropic displacement factor exponent takes the form: $-2\pi^2$[h2 a*2U11 + ... + 2 h k a* b* U12]

	U11	U22	U33	U23	U13	U12
O(2)	25(1)	25(1)	19(1)	1(1)	6(1)	-2(1)
O(5)	23(1)	21(1)	24(1)	1(1)	-2(1)	0(1)
O(6)	30(1)	26(1)	41(1)	-2(1)	9(1)	-6(1)
O(4)	24(1)	19(1)	28(1)	-2(1)	1(1)	-4(1)
O(1)	24(1)	26(1)	26(1)	2(1)	1(1)	4(1)
O(3)	54(1)	31(1)	24(1)	-5(1)	3(1)	-2(1)
C(1)	20(1)	18(1)	18(1)	-4(1)	1(1)	1(1)
C(2)	22(1)	19(1)	20(1)	1(1)	5(1)	1(1)
C(6)	15(1)	18(1)	23(1)	-1(1)	2(1)	0(1)
C(4)	16(1)	16(1)	21(1)	0(1)	2(1)	0(1)
C(10)	15(1)	20(1)	22(1)	2(1)	5(1)	-3(1)
C(8)	27(1)	30(1)	19(1)	-2(1)	5(1)	4(1)
C(3)	16(1)	23(1)	22(1)	1(1)	5(1)	-1(1)
C(17)	18(1)	19(1)	28(1)	-2(1)	1(1)	1(1)
C(12)	18(1)	22(1)	23(1)	1(1)	3(1)	-3(1)
C(5)	15(1)	19(1)	20(1)	-1(1)	4(1)	-2(1)
C(18)	30(1)	22(1)	25(1)	-5(1)	2(1)	2(1)
C(7)	21(1)	20(1)	24(1)	-2(1)	6(1)	0(1)
C(15)	15(1)	29(1)	36(1)	2(1)	7(1)	-6(1)
C(13)	21(1)	24(1)	38(1)	1(1)	11(1)	-2(1)
C(19)	32(1)	41(1)	32(1)	-9(1)	-2(1)	-5(1)
C(16)	27(1)	49(1)	44(1)	14(1)	5(1)	2(1)
C(14)	29(1)	28(1)	36(1)	-2(1)	16(1)	-7(1)
C(9)	41(1)	34(1)	22(1)	3(1)	9(1)	2(1)
C(11)	33(1)	28(1)	28(1)	2(1)	-8(1)	-1(1)

Table S20.5. Hydrogen coordinates (x 10^4) and isotropic displacement parameters ($Å^2$x 10^3) for Shenvi271.

	x	y	z	U(eq)
H(2)	4174	5458	1990	24
H(6)	926	5769	3513	22
H(4)	4601	4825	4540	22
H(3A)	4774	7533	3912	24
H(3B)	5870	6450	3623	24
H(17A)	2935	3851	3011	27
H(17B)	1635	3560	3749	27
H(12A)	2917	4096	5800	25
H(12B)	3123	5260	6849	25
H(18)	1272	4241	946	31
H(7A)	2715	7888	4545	32
H(7B)	2943	7523	6105	32
H(7C)	1425	7348	5178	32
H(13A)	619	5657	6322	32
H(13B)	463	4394	5389	32
H(19A)	-368	2665	2287	43
H(19B)	-630	2961	664	43
H(16A)	1993	890	8331	60
H(16B)	1215	1905	9141	60
H(16C)	2821	2137	8998	60
H(14A)	1616	4333	8209	36
H(14B)	17	3961	7620	36
H(9A)	3318	8636	-1071	48
H(9B)	4625	7910	-1516	48
H(9C)	4863	8783	-190	48
H(11A)	7706	5927	7815	47
H(11B)	7484	4472	8275	47
H(11C)	6442	5591	8586	47

X-ray Structure of Enal **2b** (CCDC2166577)

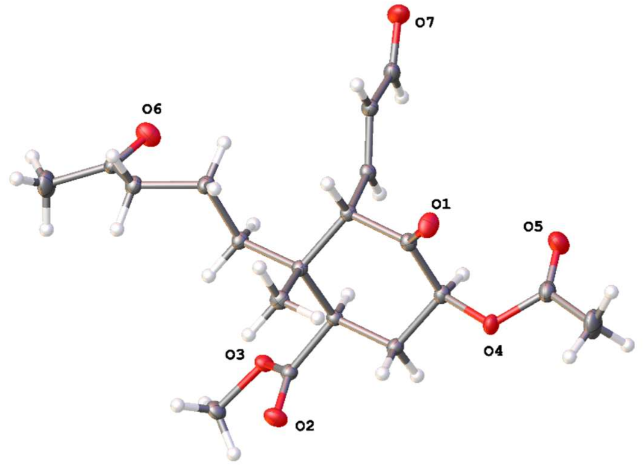

Experimental. Single colourless block-shaped crystals of **Shenvi280** were used as supplied. A suitable crystal with dimensions 0.20 × 0.20 × 0.18 mm^3 was selected and mounted on a Bruker APEX-II CCD diffractometer. The crystal was kept at a steady T = 100.00 K during data collection. The structure was solved with the ShelXT (Sheldrick, 2015) solution program using dual methods and by using Olex2 1.5-alpha (Dolomanov et al., 2009) as the graphical interface. The model was refined with XL (Sheldrick, 2008) using full matrix least squares minimisation on $\boldsymbol{F}^2$.

Crystal Data. $C_{19}H_{26}O_7$, M_r = 366.40, monoclinic, $P2_1$ (No. 4), a = 6.3391(3) Å, b = 15.7216(9) Å, c = 9.5075(5) Å, β = 99.794(2)°, $\alpha = \gamma$ = 90°, V = 933.72(9) Å^3, T = 100.00 K, Z = 2, Z' = 1, μ(MoK$_\alpha$) = 0.099, 79290 reflections measured, 6798 unique (R_{int} = 0.0355) which were used in all calculations. The final wR_2 was 0.0736 (all data) and R_1 was 0.0279 (I≥2 σ(I)).

**Compound**	**Shenvi280**
Formula	$C_{19}H_{26}O_7$
$D_{calc.}$/ g cm^{-3}	1.303
μ/mm^{-1}	0.099
Formula Weight	366.40
Colour	colourless
Shape	block-shaped
Size/mm^3	0.20×0.20×0.18
T/K	100.00
Crystal System	monoclinic
Flack Parameter	-0.01(11)
Hooft Parameter	0.02(10)
Space Group	$P2_1$
a/Å	6.3391(3)
b/Å	15.7216(9)
c/Å	9.5075(5)
α/°	90
β/°	99.794(2)
γ/°	90
V/Å^3	933.72(9)
Z	2
Z'	1
Wavelength/Å	0.71073
Radiation type	MoK□

$\Theta_{min}/^{\circ}$	2.591
$\Theta_{max}/^{\circ}$	32.607
Measured Refl's.	79290
Indep't Refl's	6798
Refl's I≥2 □(I)	6540
R_{int}	0.0355
Parameters	239
Restraints	1
Largest Peak	0.289
Deepest Hole	-0.210
GooF	1.081
wR_2 (all data)	0.0736
wR_2	0.0718
R_1 (all data)	0.0302
R_1	0.0279

Structure Quality Indicators

Reflections: d min (Mo) 2Θ=65.2° 0.66 | I/σ(I) 53.4 | Rint 3.55% | Full 50.5° 99.8

Refinement: Shift 0.000 | Max Peak 0.3 | Min Peak -0.2 | GooF 1.081 | Hooft .02(10)

A colourless block-shaped-shaped crystal with dimensions 0.20 × 0.20 × 0.18 mm^3 was mounted. Data were collected using a Bruker APEX-II CCD diffractometer operating at T = 100.00 K.
Data were measured using ϕ and ω scans with MoK$_\alpha$ radiation. . The maximum resolution that was achieved was Θ = 32.607° (0.66 Å).
The unit cell was refined using SAINT V8.40B on 1765 reflections, 2% of the observed reflections.
Data reduction, scaling and absorption corrections were performed using SAINT V8.40B. The final completeness is 99.80 % out to 32.607° in Θ. SADABS-2016/2 (Bruker, 2016/2) was used for absorption correction. wR_2(int) was 0.0819 before and 0.0505 after correction. The Ratio of minimum to maximum transmission is 0.9634. The λ/2 correction factor is Not present. The absorption coefficient □ of this material is 0.099 mm^{-1} at this wavelength (λ = 0.71073Å) and the minimum and maximum transmissions are 0.718 and 0.746.
The structure was solved and the space group $P2_1$ (# 4) determined by the ShelXT (Sheldrick, 2015) structure solution program using using dual methods and refined by full matrix least squares minimisation on $\boldsymbol{F}^2$ using version 2018/3 of XL (Sheldrick, 2008). All non-hydrogen atoms were refined anisotropically. Hydrogen atom positions were calculated geometrically and refined using the riding model. Hydrogen atom positions were calculated geometrically and refined using the riding model.

There is a single formula unit in the asymmetric unit, which is represented by the reported sum formula. In other words: Z is 2 and Z' is 1. The moiety formula is C19 H26 O7.
The Flack parameter was refined to -0.01(11). Determination of absolute structure using Bayesian statistics on Bijvoet differences using the Olex2 results in 0.02(10). The chiral atoms in this structure are: C2(S), C4(R), C5(S), C6(S). Note: The Flack parameter is used to determine chirality of the crystal studied, the value should be near 0, a value of 1 means that the stereochemistry is wrong and the model should be inverted. A value of 0.5 means that the crystal consists of a racemic mixture of the two enantiomers.
Table S21.1: Fractional Atomic Coordinates (×10^4) and Equivalent Isotropic Displacement Parameters (Å^2×10^3) for **Shenvi280**. U_{eq} is defined as 1/3 of the trace of the orthogonalised U_{ij}.

**Atom**	**x**	**y**	**z**	$\boldsymbol{U_{eq}}$
O3	870.1(12)	6766.0(5)	4803.9(9)	15.24(15)
O2	3897.0(14)	7212.3(6)	4094.5(10)	20.65(17)
O1	9554.8(14)	4884.1(6)	7539.5(11)	22.61(18)

Atom	x	y	z	U_{eq}
O4	9325.3(13)	6460.1(6)	8540.2(9)	16.96(16)
O7	2760.1(18)	2721.8(7)	9523.6(11)	27.0(2)
O5	9497.1(18)	5703.8(7)	10571.1(11)	28.6(2)
O6	-1377.4(15)	3537.9(7)	3803.9(11)	25.4(2)
C9	2997.4(17)	6784.0(7)	4871.1(11)	13.74(18)
C19	2896.0(19)	3424.6(8)	9014.2(12)	18.1(2)
C4	4119.5(16)	6182.6(6)	6009.8(11)	12.59(17)
C17	4400.3(17)	4443.1(7)	7547.4(11)	13.44(18)
C14	1474.9(18)	3836.0(7)	2525.0(12)	16.03(19)
C6	5832.3(16)	4731.5(7)	6531.3(11)	12.57(17)
C7	10288.7(19)	6198.8(8)	9850.7(13)	19.9(2)
C3	6086.6(17)	6635.3(7)	6854.6(12)	15.18(18)
C13	3001.2(17)	4008.1(7)	3917.9(12)	15.60(19)
C1	7774.8(17)	5186.8(7)	7369.9(12)	14.11(18)
C15	-837.1(18)	3710.7(7)	2673.5(12)	16.89(19)
C12	2673.5(16)	4905.7(7)	4501.3(12)	14.23(18)
C2	7335.1(17)	6052.5(7)	7984.5(11)	14.28(18)
C10	-341.7(19)	7270.6(8)	3670.5(13)	18.7(2)
C5	4720.0(16)	5330.8(7)	5316.0(11)	12.13(17)
C11	6322.7(18)	5488.0(7)	4293.5(12)	16.35(19)
C18	4284.6(18)	3637.2(7)	7977.8(11)	15.34(18)
C16	-2461(2)	3817.6(11)	1334.3(15)	30.1(3)
C8	12437(2)	6609.9(11)	10275.3(16)	31.8(3)

Table S21.2: Anisotropic Displacement Parameters (×10[4]) for **Shenvi280**. The anisotropic displacement factor exponent takes the form: $-2□^2[h^2a^{*2} \times U_{11} + ... + 2hka^* \times b^* \times U_{12}]$

Atom	U_{11}	U_{22}	U_{33}	U_{23}	U_{13}	U_{12}
O3	12.3(3)	15.1(3)	17.8(3)	3.7(3)	1.1(3)	1.7(3)
O2	18.3(4)	21.1(4)	23.3(4)	7.7(3)	5.7(3)	0.1(3)
O1	11.9(4)	26.4(4)	27.9(4)	-5.2(4)	-1.5(3)	3.6(3)
O4	14.2(3)	20.3(4)	15.1(3)	-1.2(3)	-1.1(3)	-4.1(3)
O7	37.5(5)	22.9(4)	21.1(4)	2.7(3)	5.9(4)	-10.9(4)
O5	30.3(5)	29.7(5)	22.8(4)	7.3(4)	-4.0(4)	-4.8(4)
O6	19.4(4)	31.7(5)	25.5(4)	6.8(4)	5.5(3)	1.2(4)
C9	13.4(4)	12.4(4)	15.1(4)	0.2(3)	1.5(3)	0.6(3)
C19	18.2(5)	21.0(5)	14.1(4)	0.3(4)	0.2(4)	-4.6(4)
C4	11.9(4)	11.9(4)	13.6(4)	0.7(3)	1.4(3)	0.8(3)
C17	10.8(4)	15.3(4)	13.8(4)	0.4(3)	0.8(3)	0.4(3)
C14	16.9(5)	15.9(5)	15.0(4)	-2.3(3)	1.9(4)	0.0(4)
C6	10.4(4)	13.1(4)	14.1(4)	0.0(3)	1.6(3)	0.7(3)
C7	19.1(5)	20.8(5)	17.8(5)	-2.2(4)	-2.5(4)	0.0(4)
C3	14.3(4)	13.4(4)	17.0(4)	-0.8(3)	0.3(3)	-0.6(3)
C13	13.9(4)	14.3(4)	17.6(4)	-1.8(4)	0.1(4)	1.0(4)
C1	11.9(4)	16.5(4)	13.8(4)	0.5(3)	1.7(3)	0.4(3)
C15	16.1(4)	14.0(4)	19.3(5)	-1.7(4)	-0.7(4)	-0.7(4)
C12	11.7(4)	13.9(4)	16.2(4)	-1.4(3)	0.0(3)	0.8(3)
C2	11.4(4)	16.0(5)	14.9(4)	-1.2(3)	0.7(3)	-1.0(3)
C10	16.8(5)	18.7(5)	18.9(5)	4.5(4)	-1.4(4)	3.6(4)
C5	10.7(4)	12.3(4)	13.3(4)	0.2(3)	1.9(3)	0.5(3)
C11	15.5(5)	18.0(5)	16.6(4)	-0.1(4)	6.0(4)	-0.8(4)
C18	16.5(4)	15.2(4)	14.1(4)	-0.3(3)	1.8(3)	-1.5(4)
C16	23.1(6)	38.5(8)	24.4(6)	0.7(5)	-8.0(5)	-7.5(5)

Atom	U_{11}	U_{22}	U_{33}	U_{23}	U_{13}	U_{12}
C8	23.1(6)	44.2(9)	24.5(6)	-2.9(6)	-6.1(5)	-9.7(6)

Table S21.3: Bond Lengths in Å for **Shenvi280**.

Atom	Atom	Length/Å	Atom	Atom	Length/Å
O3	C9	1.3395(13)	C17	C6	1.5038(15)
O3	C10	1.4488(13)	C17	C18	1.3373(15)
O2	C9	1.2111(14)	C14	C13	1.5255(15)
O1	C1	1.2098(14)	C14	C15	1.5090(16)
O4	C7	1.3547(14)	C6	C1	1.5265(15)
O4	C2	1.4333(13)	C6	C5	1.5629(14)
O7	C19	1.2153(16)	C7	C8	1.4992(18)
O5	C7	1.2015(16)	C3	C2	1.5267(15)
O6	C15	1.2130(15)	C13	C12	1.5432(15)
C9	C4	1.5200(14)	C1	C2	1.5254(16)
C19	C18	1.4672(16)	C15	C16	1.5044(17)
C4	C3	1.5381(15)	C12	C5	1.5452(15)
C4	C5	1.5681(14)	C5	C11	1.5409(15)

Table S21.4: Bond Angles in ° for **Shenvi280**.

Atom	Atom	Atom	Angle/°	Atom	Atom	Atom	Angle/°
C9	O3	C10	115.21(9)	O1	C1	C6	122.43(10)
C7	O4	C2	115.74(9)	O1	C1	C2	121.80(10)
O3	C9	C4	111.48(9)	C2	C1	C6	115.77(9)
O2	C9	O3	123.87(10)	O6	C15	C14	122.50(11)
O2	C9	C4	124.63(10)	O6	C15	C16	121.28(12)
O7	C19	C18	124.48(12)	C16	C15	C14	116.23(11)
C9	C4	C3	108.89(8)	C13	C12	C5	115.01(8)
C9	C4	C5	110.45(8)	O4	C2	C3	108.03(9)
C3	C4	C5	112.43(8)	O4	C2	C1	109.47(9)
C18	C17	C6	123.55(10)	C1	C2	C3	111.87(9)
C15	C14	C13	115.12(9)	C6	C5	C4	108.37(8)
C17	C6	C1	109.10(9)	C12	C5	C4	109.70(8)
C17	C6	C5	114.27(8)	C12	C5	C6	110.15(8)
C1	C6	C5	109.02(8)	C11	C5	C4	111.20(8)
O4	C7	C8	111.33(11)	C11	C5	C6	107.47(8)
O5	C7	O4	123.64(11)	C11	C5	C12	109.91(9)
O5	C7	C8	125.03(12)	C17	C18	C19	119.46(10)
C2	C3	C4	111.53(9)				
C14	C13	C12	111.71(9)				

Table S21.5: Torsion Angles in ° for **Shenvi280**.

Atom	Atom	Atom	Atom	Angle/°
O3	C9	C4	C3	-136.28(9)
O3	C9	C4	C5	99.80(10)
O2	C9	C4	C3	45.38(14)
O2	C9	C4	C5	-78.53(13)
O1	C1	C2	O4	9.03(15)
O1	C1	C2	C3	128.75(12)
O7	C19	C18	C17	-176.23(12)

Atom	Atom	Atom	Atom	Angle/°
C9	C4	C3	C2	-177.95(8)
C9	C4	C5	C6	-179.10(8)
C9	C4	C5	C12	-58.80(11)
C9	C4	C5	C11	62.99(11)
C4	C3	C2	O4	169.69(8)
C4	C3	C2	C1	49.13(12)
C17	C6	C1	O1	109.91(12)
C17	C6	C1	C2	-70.15(12)
C17	C6	C5	C4	65.51(11)
C17	C6	C5	C12	-54.49(11)
C17	C6	C5	C11	-174.22(9)
C14	C13	C12	C5	-147.47(9)
C6	C17	C18	C19	177.74(10)
C6	C1	C2	O4	-170.92(9)
C6	C1	C2	C3	-51.20(12)
C7	O4	C2	C3	157.86(9)
C7	O4	C2	C1	-80.09(12)
C3	C4	C5	C6	59.06(11)
C3	C4	C5	C12	179.35(8)
C3	C4	C5	C11	-58.85(11)
C13	C14	C15	O6	-18.41(16)
C13	C14	C15	C16	161.21(11)
C13	C12	C5	C4	-172.99(9)
C13	C12	C5	C6	-53.79(12)
C13	C12	C5	C11	64.44(12)
C1	C6	C5	C4	-56.83(10)
C1	C6	C5	C12	-176.84(8)
C1	C6	C5	C11	63.43(11)
C15	C14	C13	C12	-71.73(12)
C2	O4	C7	O5	-5.17(18)
C2	O4	C7	C8	175.18(11)
C10	O3	C9	O2	2.98(16)
C10	O3	C9	C4	-175.36(9)
C5	C4	C3	C2	-55.22(11)
C5	C6	C1	O1	-124.69(12)
C5	C6	C1	C2	55.26(12)
C18	C17	C6	C1	-103.58(12)
C18	C17	C6	C5	134.12(11)

Table S21.6: Hydrogen Fractional Atomic Coordinates (×10^4^) and Equivalent Isotropic Displacement Parameters (Å^2×10^3) for **Shenvi280**. U_{eq} is defined as 1/3 of the trace of the orthogonalised U_{ij}.

Atom	x	y	z	U_{eq}
H19	2044.82	3866.51	9307.13	22
H4	3111.22	6047.44	6681.26	15
H17	3530.67	4855.84	7902.91	16
H14A	1560.11	4318.56	1868.01	19
H14B	1969.79	3320.9	2078.05	19
H6	6350.52	4215.38	6078.14	15
H3A	7035.36	6823.29	6188.22	18
H3B	5614.88	7146.76	7323.38	18
H13A	4495.89	3951.15	3754.17	19
H13B	2764.94	3578.61	4636.59	19
H12A	1607.36	4868.73	5148.39	17
H12B	2065.5	5276.6	3691.75	17
H2	6496.29	5970.42	8774.82	17
H10A	-214.89	7019.65	2744.96	28
H10B	220.89	7852.39	3719.1	28
H10C	-1850.74	7281.01	3780.61	28
H11A	5665.14	5858.17	3510.92	25
H11B	6711.37	4943.96	3904.18	25
H11C	7610.78	5762.01	4815.02	25
H18	5094.97	3205.96	7615.45	18
H16A	-3800.7	3537.7	1453.95	45
H16B	-1915.17	3560.09	528.88	45
H16C	-2725.87	4424.71	1147.85	45
H8A	12867.88	6878.78	9438.52	48
H8B	13493.61	6177.55	10658.26	48
H8C	12352.65	7041.92	11006.7	48
